# Comparison of DNA Methylation Changes Between the Gestation Period and the After-Delivery State: A Pilot Study of 10 Women

**DOI:** 10.3389/fnut.2022.829915

**Published:** 2022-05-04

**Authors:** Ming-Wei Lin, Mong-Hsun Tsai, Ching-Yu Shih, Yi-Yun Tai, Chien-Nan Lee, Shin-Yu Lin

**Affiliations:** ^1^Department of Obstetrics and Gynecology, National Taiwan University Hospital, Taipei, Taiwan; ^2^Bioinformatics and Biostatistics Core Lab, Centers of Genomic and Precision Medicine, National Taiwan University, Taipei, Taiwan; ^3^Institute of Biotechnology, National Taiwan University, Taipei, Taiwan; ^4^Department of Medical Genetics, National Taiwan University Hospital, Taipei, Taiwan

**Keywords:** DNA methylation, gestational adaptation, pregnancy, pathway analysis, after-pregnancy status

## Abstract

**Background:**

Gestational adaptation occurs soon after fertilization and continues throughout pregnancy, whereas women return to a pre-pregnancy state after delivery and lactation. However, little is known about the role of DNA methylation in fine-tuning maternal physiology. Understanding the changes in DNA methylation during pregnancy is the first step in clarifying the association of diet, nutrition, and thromboembolism with the changes in DNA methylation. In this study, we investigated whether and how the DNA methylation pattern changes in the three trimesters and after delivery in ten uncomplicated pregnancies.

**Results:**

DNA methylation was measured using a Human MethylationEPIC BeadChip. There were 14,018 cytosine-guanine dinucleotide (CpG) sites with statistically significant changes in DNA methylation over the four time periods (*p* < 0.001). Overall, DNA methylation after delivery was higher than that of the three trimesters (*p* < 0.001), with the protein ubiquitination pathway being the top canonical pathway involved. We classified the CpG sites into nine groups according to the changes in the three trimesters and found that 38.37% of CpG sites had DNA methylation changes during pregnancy, especially between the first and second trimesters.

**Conclusion:**

DNA methylation pattern changes between trimesters, indicating possible involvement in maternal adaptation to pregnancy. Meanwhile, DNA methylation patterns during pregnancy and in the postpartum period were different, implying that puerperium repair may also function through DNA methylation mechanisms.

## Introduction

Gestational adaptation occurs soon after fertilization and continues throughout pregnancy, while women return to a pre-pregnancy state after delivery and lactation. Almost every organ system undergoes important physiological alterations and adaptations that support fetal development and help the mother and fetus survive the demands of childbirth (1). For example, plasma volume and total red cell mass are controlled by different mechanisms, and pregnancy provides the most dramatic example of how this can occur (2). Moreover, the maintenance of pregnancy relies on finely tuned immune adaptations (3). These immunological changes increase fetal survival and permit accommodation of the semi-allogeneic fetal graft (4). Since physiological adaptations during pregnancy are crucial for the developing fetus and the demands of childbirth, understanding these changes is important to recognize pathological deviations in obstetric complications and to optimize the outcomes for both the mother and her baby.

There is growing evidence that epigenetic mechanisms (DNA methylation, histone modification, and non-coding RNAs) are responsible for tissue-specific gene expression during growth and development and that these mechanisms underlie developmental plasticity (5). Such tuning of phenotypes has potential adaptive value and Darwinian fitness advantage because it attempts to produce a phenotype optimized for current circumstances or the predicted future environment (6). Maternal caloric malnutrition or an imbalanced diet with low protein can lead to changes in DNA methylation and fetal programming, making the offspring more resilient to future stress (7). However, the nuances of changes in DNA methylation and the transcriptional control of gene expression are not completely understood. An increasing number of studies are investigating obstetric complications such as gestational diabetes, preterm birth, and preeclampsia through epigenetic mechanism (8–13). Nevertheless, most studies have focused on epigenetic changes in the placenta or cord blood instead of maternal blood. In addition, little is known about how DNA methylation changes during gestation. Variation in DNA methylation before and throughout pregnancy was first described by Pauwels et al. (14). They noted that the mean global DNA methylation percentage was significantly higher before pregnancy than during pregnancy. Chen et al. later investigated methylation dynamics in women from preadolescence to late pregnancy (15). Nevertheless, the present study used different female cohorts to show the methylation dynamics before and after pregnancy. Several studies have investigated epigenetic markers in the maternal peripheral blood for obstetric complications (16–21). However, the effect of gestational age on DNA methylation profiles remains unknown. Pregnancy DNA methylation profiling in a healthy population may provide a basis for improving our understanding of the development of pregnancy complications. Therefore, our aim was to study DNA methylation changes in different trimesters and its status after delivery and to describe the DNA methylation patterns in different physiological pathways.

## Materials and Methods

Ten women with uncomplicated pregnancies were enrolled. The inclusion criteria were pregnant women who visited our hospital before the 14th week of gestation and were willing to participate in the study. The exclusion criteria were pregnant women with underlying medical conditions, including but not limited to any infection symptoms or signs, autoimmune disorders, abnormal thyroid function, asthma, urticaria, thalassemia carrier, chronic hypertension, diabetes mellitus, gestational diabetes mellitus, gestational hypertension, preeclampsia, fetal anomaly, intrauterine growth retardation, small or large for gestational age, polyhydramnios, oligohydramnios, and placental insufficiency. Women who agreed to participate and provided written informed consent were included in this study. Peripheral blood sampling was conducted to analyze DNA methylation between 10 and 14 weeks of gestation (average at the 10th week), 24–28 weeks of gestation (average at the 25th week), 38–40 weeks of gestation (average at the 38th week), and to observe the status of methylation after delivery and lactation (average at 10 months after delivery and 4 months after stopping breastfeeding). This study was approved by the Research Ethics Committee of National Taiwan University Hospital (NTUH-REC No. 201907038RINC).

### Isolation of Genomic DNA, Bisulfite Conversion, DNA Amplification, Fragmentation, and Hybridization of Illumina Infinium® Methylation Array

Genomic DNA was extracted using a QIAamp DNA Micro Kit (Qiagen). Genomic DNA was isolated using proteinase K-phenol-chloroform extraction following standard protocols with 0.5% sodium dodecyl sulfate and 200 μg/mL proteinase K. Normalized DNA concentrations of 50 ng/μL and total genomic DNA (500 ng) were used for DNA bisulfite conversion using the EZ DNA Methylation™ kit (Zymo Research). Bisulfite-treated DNA (200 ng) was used in the Infinium MethylationEPIC assay. First, the bisulfite-treated DNA was amplified by DNA polymerase during the incubation step in the Illumina hybridization oven for 20–24 h at 37°C. The amplified DNA product was fragmented into 300–600 base pairs. After alcohol precipitation and resuspension of the fragmented DNA, the BeadChip was prepared for hybridization in the capillary flow-through chamber (Illumina Human MethylationEPIC BeadChip Array, Illumina) Each array accommodated eight samples, and the plate layout is listed in Supplementary Table 1. The amplified and fragmented DNA samples were annealed to locus-specific 50-mers during the hybridization step at 48°C for 16 h in an Illumina Hybridization Oven. After hybridization, allele specificity was determined using an enzymatic single-base extension. The products were then fluorescently stained with biotin-ddNTPs or dinitrophenol-ddNTPs. The intensity of the bead fluorescence was measured using Illumina HiScan (iScan Control Software v.3.3.28), and the results were analyzed using GenomeStudio Software v2011.1 for methylation profiles.

### Statistical Analysis

Before data analysis, we used data from the first trimester for normalization for each woman. Principal component analysis (PCA) was also performed to exclude batch effects (Supplementary Figure 1). Differential DNA methylation analysis was conducted using R software (version 3.6.3). Methylation levels (β values) were obtained from the microarray data, where the β value is the ratio of the methylated probe intensity to the overall intensity. The β values were transformed into the corresponding M values by log transformation, and differential methylation analysis was conducted (22). One-way ANOVA was used to identify differentially methylated cytosine-guanine dinucleotide (CpG) sites during the four time periods. CpG sites with probe SNPs within 10 base pairs (bp) were deleted. We also removed CpG probes with *p* > 0.01 in one sample. Only probes with a significant difference in the average β value over the four time periods (*p* < 0.001) were selected for further analysis (*n* = 14,018). Average delta-beta values indicating differentially methylated CpG sites were used with different cut-offs to classify the data into nine categories from the three trimesters. The details are presented in Table 1. We considered a difference in methylation > 2%—that is, at least one CpG site within the designated region must have a mean difference in methylation of >2% between samples among the three trimesters (Δβ = 0.02)—to be of biological interest. Furthermore, gene ontology and pathway analyses were conducted using Ingenuity Pathway Analysis (IPA® Qiagen, Redwood City, CA) on (1) the top 1,000 significant differentially methylated CpG sites over the four groups (time periods) (*p* < 0.001) and (2) the significant findings (*p* < 0.001) from the nine groups. The heat map was produced using the pheatmap package (version 1.0.12) in R.

**Table 1:** The nine groups with different methylation patterns in the three trimesters.

**Group**	**β value of 1st trimester– β value of 2nd trimester**	**β value of 2nd trimester –β value of 3rd trimester**	***N* (%) of CpG sites**	**Illustration**
1	< -0.02	< -0.02	43 (0.31%)	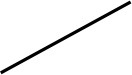
2	>0.02	>0.02	39 (0.28%)	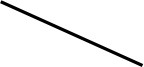
3	< -0.02	>0.02	9 (0.06%)	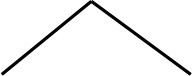
4	>0.02	< -0.02	117 (0.83%)	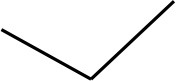
5	Between −0.02 and 0.02	< -0.02	513 (3.66%)	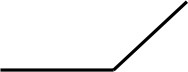
6	Between −0.02 and 0.02	>0.02	48 (0.34%)	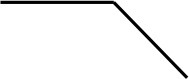
7	< -0.02	Between −0.02 and 0.02	995 (7.10%)	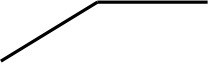
8	>0.02	Between −0.02 and 0.02	3,173 (22.64%)	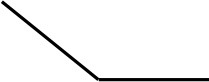
9	Between −0.02 and 0.02	Between −0.02 and 0.02	8,639 (61.63%)	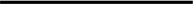

*These groups are classified according to the differences of the average β values between the first and the second trimesters, as well as that between the second and the third trimesters*.

## Results

**Figure 1:**
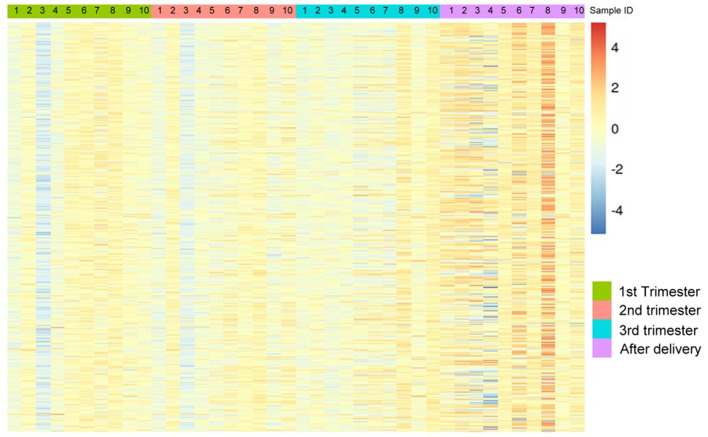
The heat map visualization of differentially methylated CpG sites. Top 1,000 differentially methylated CpG sites of 10 women within the 1st trimester, 2nd trimester, 3rd trimester and the after-delivery status are shown by using M value (*p* < 0.001). A scale is shown on the right, in which red and blue correspond to a higher and a lower methylation status, respectively.

**Figure 2:**
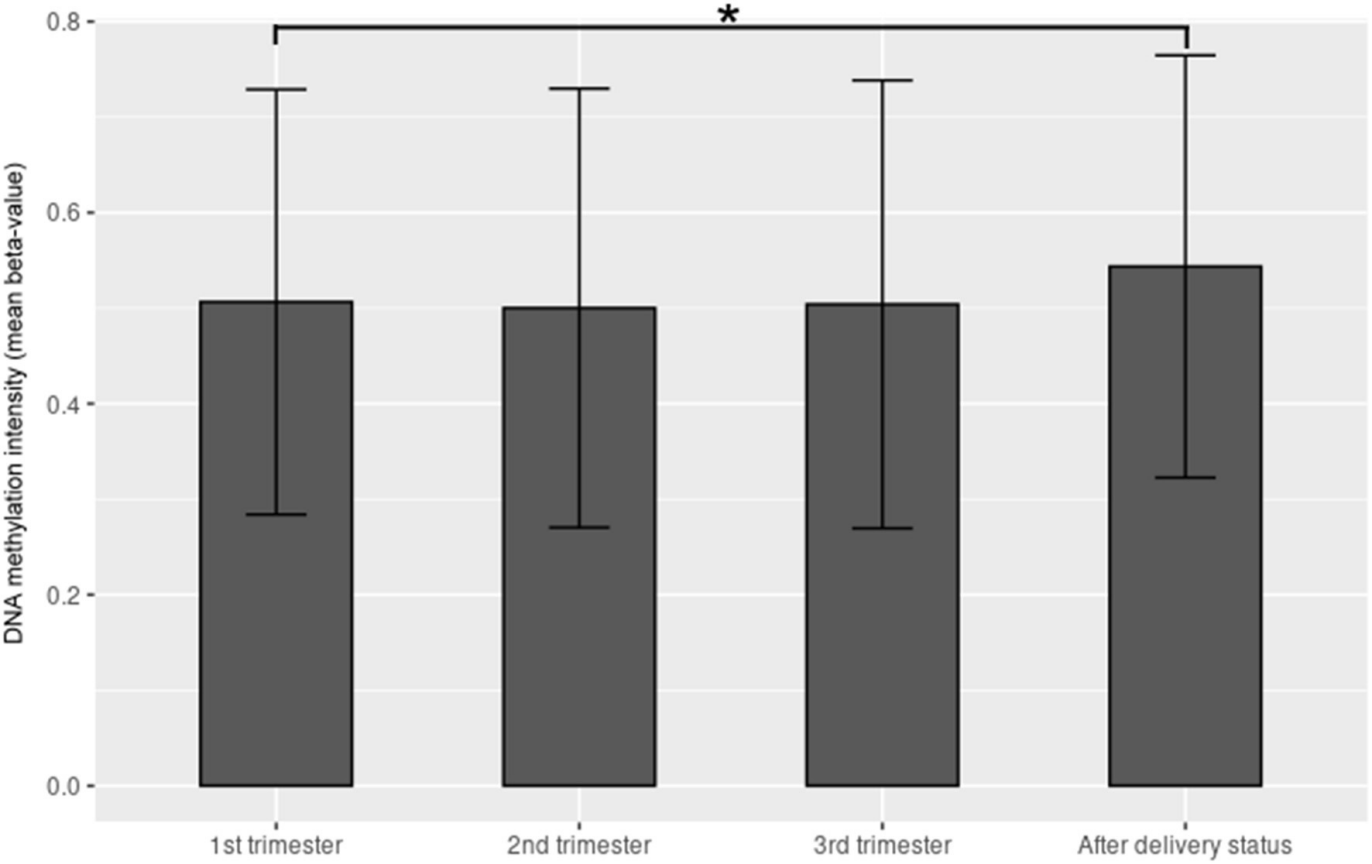
The mean β value of the top 1,000 CpG sites with significant methylation differences within the 1st trimester, 2nd trimester, 3rd trimester and after-delivery status (*p* < 0.001). Y-axis denotes DNA methylation intensity shown by mean β value; X-axis denotes biosampling occasions. *p < 0.001.

The demographic characteristics are shown in Table 2. Among the 865,918 CpG sites, 14,018 showed statistically significant differences in methylation between the four time periods (*P* < 0.001). The heat map illustrates the top 1,000 CpG sites with significant methylation differences during the four periods (Figure 1). The mean DNA methylation level was higher after delivery than in the three trimesters (*p* < 0.001) (Figure 2).

**Table 2:** Clinical characteristics in the pregnant women included in this study.

**Baseline characteristics**	**All**
*N*	10
Age (years)	33.2 (4.5)
Nulliparous (*N*, %)	6 (60%)
Gestational age at the FPV (weeks)	10 (2.5)
Family history of DM (*N*, %)	5 (50%)
Pre-pregnancy BW (Kg)	53 (4.2)
Pre-pregnancy BMI (kg/m^2^)	21.5 (2.2)
SBP (mmHg)	105.4 (9.6)
DBP (mmHg)	61.1 (9.1)
**Laboratory test results at the first prenatal visit**	
Hb (g/dL)	12.6 (1.2)
WBC (K/μL)	7.9 (1.4)
TC (mg/dL)	184 (36.3)
LDL-C (mg/dL)	99 (26.1)
HDL-C (mg/dL)	73.1 (11.3)
TG (mg/dL)	102.6 (46.2)
FPG (mg/dL)	81.2 (4.1)
HbA1c (%)	5.2 (0.3)
GWG at 24–28 gestational weeks (kg)	3.7 (3.4)
**Laboratory test results at 24–28 gestational weeks**	
Hb (g/dL)	11.2 (1)
WBC (K/μL)	8.4 (1.3)
**Glucose level during OGTT at 24–28 gestational weeks**	
FPG during OGTT (mg/dL)	76.7 (3.4)
1hPG (mg/dL)	122.4 (19.9)
2hPG (mg/dL)	105.5 (23.3)
GWG at deliver (kg)	6.9 (4.5)
**Laboratory test results at deliver**	
Hb (g/dL)	11.5 (0.8)
WBC (K/μL)	9.4 (2.2)
Gestational age at deliver	38.9 (1.5)
Birth weight (g)	2961.3 (347.1)

*Mean (standard deviations) or N (%) were shown*.

*BMI, body mass index; BW, body weight; DBP, diastolic blood pressure; DM, diabetes mellitus; FPG, fasting plasma glucose; FPV, first prenatal visit; GWG, gestational weight gain, compared to the body weight before pregnancy; Hb, hemoglobin; HbA1c, hemoglobin A1c; HbA1c, glycosylated hemoglobin; HDL-C, HDL cholesterol; LDL-C, LDL cholesterol; OGTT, oral glucose tolerance tests; FPG during OGTT, fasting plasma glucose during oral glucose tolerance tests; SBP, systolic blood pressure; TC, total cholesterol; TG, triglycerides; WBC, white blood cell; 1hPG, 1-hour plasma glucose during oral glucose tolerance tests; 2hPG, 2-hour plasma glucose during oral glucose tolerance tests*.

IPA of the 14,018 CpG sites revealed that the top five canonical pathways were the “protein ubiquitination pathway”, followed by “hypoxia signaling in the cardiovascular system,” “inhibition of ARE-mediated mRNA degradation pathway,” “tumor necrosis factor receptor (TNFR)2 signaling,” and “phagosome maturation” (Table 3). The top five physiological systems were “embryonic development,” followed by “hematologic system development and function,” “humoral immune response,” “immune cell trafficking,” and “organ morphology.”

**Table 3:** The ingenuity pathway analysis for the top 1,000 CpG sites with statistically significant mean β value difference within the 1st trimester, 2nd trimester, 3rd trimester, and after-delivery status.

**Name**	***P*-value**	**Genes**
**The top five canonical pathways**		
Protein ubiquitination pathway	2.41E-11	UBE2D1,UBE2E1,UBE2F,UBE2H,UBE2J2, UBE2K,UBE2L6,UBE2O,UBE2Q2,UBE2V1, UBE3A,UCHL3,USP1,USP12,USP22,USP3, USP36,USP4,USP40,USP42,USP45,USP48, USP54,USP8,ZBTB12
Hypoxia signaling in the cardiovascular system	1.41E-06	UBE2D1,UBE2E1,UBE2F,UBE2H,UBE2J2, UBE2K,UBE2L6,UBE2O,UBE2Q2,UBE2V1
Inhibition of ARE-mediated mRNA degradation pathway	5.57E-04	TNF,TNFRSF1A,TNFRSF1B,TNFSF10, TNFSF13,YWHAG,YWHAQ,ZFP36,ZFP36L1
TNFR2 signaling	9.18E-04	TNF,TNFAIP3,TNFRSF1B,TRAF1
Phagosome maturation	1.43E-03	TUBA1B,TUBB,VAMP2,VPS28, VPS37B,VTI1A,YKT6,ZBTB12

**Figure 3:**
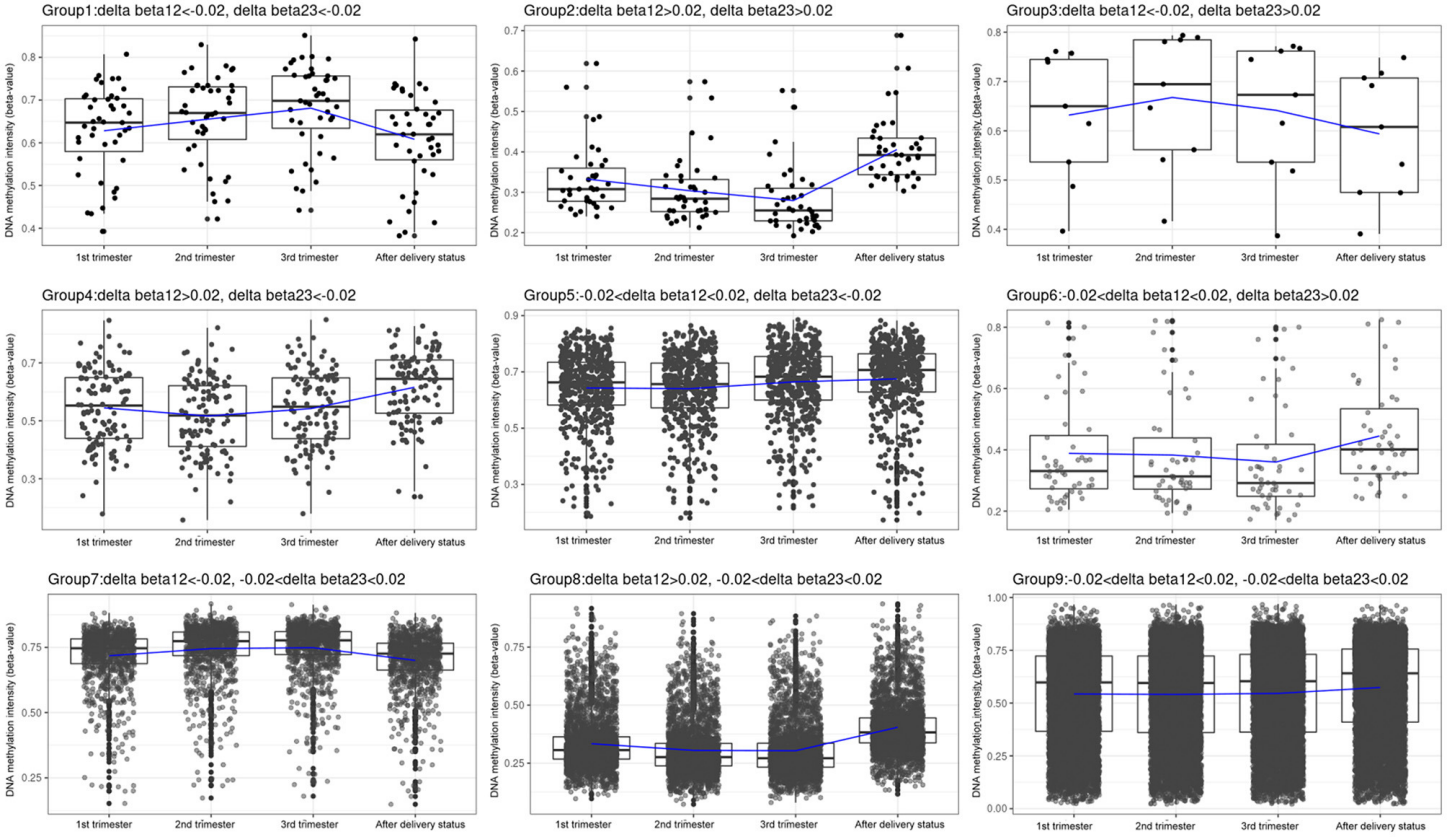
Box plots of the DNA methylation intensity in the 9 groups, classified by average delta-beta values with different cut-offs from 3 trimesters. Group 1: The β values went up with time. Group 2: The β values went down with time. Group 3: The β values rose and then fell. Group 4: The β values decreased before increasing. Group 5: The β values remained the same in the first two trimesters and then increased in the third trimester. Group 6: The β values remained the same in the first two trimesters and then decreased in the third trimester. Group 7: The β values rose from the first trimester to the second trimester and remained the same between the second and third trimesters. Group 8: The β values dropped in the second trimester and remained the same between the second and third trimesters. Group 9: The β values remained roughly the same over the three trimesters. Y-axis denotes DNA methylation intensity shown by mean β value; X-axis denotes biosampling occasions. Each box contains the middle 50% of the data, with the upper edge (hinge) of the box indicating the 75th percentile and the lower one indicating the 25th percentile and the interquartile range (IQR). The line in the box represents median values. The upper and lower ends of the vertical lines (“whiskers”) indicate the upper quartile +1.5 IQR and lower quartile −1.5 IQR, respectively. The individual values outside this range are marked by circles.

We classified the 14,018 CpG sites into nine groups according to changes in the three trimesters (Table 1; Figure 3; Supplementary Tables 2–10). Group 1: The β values increased with time (*N* = 43, 0.31%). Group 2: The β values decreased with time (*N* = 39, 0.28%). Group 3: The β values rose and then fell (*N* = 9, 0.06%). Group 4: The β values decreased before increasing (*N* = 117, 0.83%). Group 5: The β values remained the same in the first two trimesters and then increased in the third trimester (*N* = 513, 3.66%). Group 6: The β values remained the same in the first two trimesters and then decreased in the third trimester (*N* = 48, 0.34%). Group 7: The β values increased from the first trimester to the second trimester and remained the same between the second and third trimesters (*N* = 995, 7.10%). Group 8: The β values dropped in the second trimester and remained the same between the second and third trimesters (*N* = 3,173, 22.64%). Group 9: The β values remained approximately the same over the three trimesters (*N* = 8,639, 61.63%). IPA was performed in Groups 1 and 2 and in Groups 4, 5, 6, 7, 8, and 9 (Tables 4, 5).

**Table 4:** The ingenuity pathway analysis of each group for the top 5 physiological system development and function within 3 trimesters and after delivery.

**Name**	***P*-value range**
**Group 1 and 2 (82 CpG sites)**	
Hematological system development and function	4.60E-02–8.11E-04
Nervous system development and function	9.69E-03–8.11E-04
Tissue development	4.60E-02–8.11E-04
Tissue morphology	2.25E-02–8.11E-04
Organismal functions	1.86E-02–1.86E-02
**Group 4 (117 CpG sites)**	
Cardiovascular system development and function	2.96E-02–8.03E-04
Cell-mediated immune response	1.50E-03–1.50E-03
Hematologic system development and function	7.47E-03–1.50E-03
Immune cell trafficking	1.50E-03–1.50E-03
Lymphoid tissue structure and development	1.50E-03–1.50E-03
**Group 5 (513 CpG sites)**	
Nervous system development and function	6.67E-03–4.36E-04
Embryonic development	1.33E-02–2.79E-03
Hair and skin development and function	2.79E-03–2.79E-03
Humoral immune response	1.33E-02–4.99E-03
Renal and urological system development and function	1.33E-02–4.99E-03
**Group 6 (48 CpG sites)**	
Hematological system development and function	4.22E-02–4.40E-05
Humoral immune response	3.52E-02–4.40E-05
Immune cell trafficking	4.22E-02–4.40E-05
Tissue morphology	8.19E-03–9.67E-04
Embryonic development	3.94E-02–2.42E-03
**Group 7 (995 CpG sites)**	
Hematological system development and function	1.46E-03–1.17E-07
Lymphoid tissue structure and development	1.46E-03–1.17E-07
Hematopoiesis	1.02E-03–8.06E-07
Tissue development	1.30E-03–8.06E-07
Embryonic development	7.41E-04–1.28E-06
**Group 8 (3,173 CpG sites)**	
Hematological system development and function	6.74E-03–2.49E-06
Immune cell trafficking	6.74E-03–2.49E-06
Tissue development	4.12E-03–1.28E-05
Tissue morphology	7.12E-04–9.28E-05
Hematopoiesis	3.84E-03–1.62E-04
**Group 9 (8,639 CpG sites)**	
Tissue development	6.04E-04–1.21E-07
Hair and skin development and function	2.88E-05–2.88E-05
Hematological system development and function	6.04E-04–8.76E-05
Organismal development	6.04E-04–1.49E-04
Connective tissue development and function	2.01E-04–1.98E-04

**Table 5:** The ingenuity pathway analysis of each group for the top 5 canonical pathways within 3 trimesters and after delivery.

**Name**	***P*-value**	**Genes**
**Group 1 and 2 (82 CpG sites)**		
Insulin secreting signaling pathway	1.17E-03	ITPR1,PRKAG2,VTI1A
Netrin signaling	1.53E-03	ITPR1,PRKAG2
GRCR-mediated integration of enteroendocrine signaling exemplified by an L cell	1.53E-03	ITPR1,PRKAG2
Synaptogenesis signaling pathway	1.82E-03	ITPR1,PRKAG2,VTI1A
Neuropathic pain signaling in dorsal horn neurons	2.92E-03	ITPR1,PRKAG2
**Group 4 (117 CpG sites)**		
Maturity onset diabetes of young (MODY) signaling	3.53E-03	CACNA1A,OGDH
2-ketoglutarate dehydrogenase complex	5.98E-03	OGDH
CDK5 signaling	1.12E-02	CACNA1A,PPP2R2A
Cardiac-adrenergic signaling	1.86E-02	CACNA1A,PPP2R2A
Dopamine-DARPP32 feedback in cAMP signaling	2.36E-02	CACNA1A,PPP2R2A
**Group 5 (513 CpG sites)**		
Nitric oxide signaling in the cardiovascular system	3.99E-05	AKT2,ATP2A3,GUCY2D,ITPR1,MAPK1,PRKCZ
Apelin cardiomyocyte signaling pathway	5.04E-05	AKT2,ATP2A3,ITPR1,MAPK1,PRKCZ,SLC9A8
Phospholipase C signaling	5.51E-05	AHNAK,CREB3L3,ITPR1,MAPK1,MPRIP, NFATC1,PRKCZ,RHOA,ZAP70
NGF signaling	1.12E-04	AKT2,CREB3L3,MAPK1,PRKCZ,RHOA, RPS6KA2
IL-7 signaling pathway	1.29E-04	AKT2,CXCR5,MAPK1,MCL1,NFATC1
**Group 6 (48 CpG sites)**		
Ceramide signaling	7.88E-04	PIK3CD,TNFRSF1B
PD-1, PD-L1 cancer immunotherapy pathway	1.08E-03	PIK3CD,TNFRSF1B
IL-6 signaling	1.61E-03	PIK3CD,TNFRSF1B
Type II diabetes mellitus signaling	1.98E-03	PIK3CD,TNFRSF1B
HMGB1 signaling	2.48E-03	PIK3CD,TNFRSF1B
**Group 7 (995 CpG sites)**		
T helper cell differentiation	3.05E-05	IFNG,IL21,IL23R,IL5,IL6ST,TNF
Cardiac hypertrophy signaling (enhanced)	9.33E-05	EP300,FGFR1,GNG7,HDAC3,IFNG,IL21,IL5, IL6ST,IL7R,ITGB2,LEP,PLCH1,PTK2,TNF
Hepatic fibrosis/hepatic stellate cell activation	1.99E-04	BCL2,COL24A1,COL5A1,FGFR1,IFNG,LEP,MYH10,TNF
IL-7 signaling pathway	4.40E-04	BCL2,FYN,IFNG,IL7R,PTK2
Systemic lupus erythematosus in B cell signaling pathway	5.42E-04	BCL2,FYN,IFNG,IL21,IL5,IL6ST,LEP, SYK,TNF
**Group 8 (3,173 CpG sites)**		
TREM1 signaling	5.41E-04	IL18,MPO,NLRP3,NOD1,STAT5A,TLR5, TLR6,TREM1
Fcγ receptor-mediated phagocytosis in macrophages and monocytes	8.49E-04	FYB1,INPP5D,LYN,NCK1,NCK2,PRKCE, PRKCZ,SRC,VAV2
IL-15 production	1.25E-03	AATK,DSTYK,FES,IGF1R,LYN,MAP2K6, PRKCZ,SRC,STAT1,TNK2
Insulin secretion signaling pathway	2.00E-03	AGO1,AGO2,AGO3,CACNA1C,CHRM3, EIF4G3,ITPR1,KLF11,LYN,PRKCE, PRKCZ,SCNN1A,SRC,STAT1,STAT5A
Molecular mechanisms of cancer	2.17E-03	ARHGEF10,ARHGEF17,AXIN1,BMP1,CCND2, CTNND1,DAXX,E2F3,GNA12,HHAT,HIPK2, ITGA6,MAP2K6,NOTCH1,PRKCE,PRKCZ, RB1,SHC1,SRC,SYNGAP1,TFDP1
**Group 9 (8,639 CpG sites)**		
Molecular mechanisms of cancer	3.12E-08	ABL1,AKT1,APC,ARHGEF10,ARHGEF15, ARHGEF16,ARHGEF17,ARHGEF7,AXIN1,BMP6, CAMK2D,CASP8,CCND1,CCND2,CDK3, CDK5,CREBBP,CTNNB1,DAXX,DIABLO
		DIRAS3,E2F2,E2F5,FNBP1,FYN,FZD2,FZD5, FZD8,GAB1,GNA13,GNAO1,GNAT1,GRB2,ITGA6, ITGAL,JAK1,JAK3,KRAS,LRP5,MAP2K3,MAPK1, MYC,NAIP,NF1,NFKBIA,NOTCH1,PAK4, PIK3C2B,PIK3CD,PIK3R2,PIK3R5,PRKAG2, PRKAR1B,PRKCE,PRKCI,PRKCZ,PRKD3, PSENEN,RAF1,RALB,RAP1B,RAPGEF1,RASGRF1, RBPJ,RHOBTB2,RHOG,SMAD3,SMAD4,SMAD7, TCF3,TFDP1,TGFB2,TGFBR1,WNT4,WNT5B,WNT7A
c Epsilon RI signaling	4.64E-07	AKT1,CSF2,FCER1G,FYN,GAB1,GRAP2, GRB2,INPP5J,INPP5K,KRAS,LAT,LYN,MAP2K3, MAPK1,MS4A2,PDPK1,PIK3C2B,PIK3CD,PIK3R2, PIK3R5,PLA2G4F,PLA2G6,PRKCE,PRKCI,PRKCZ, PRKD3,RAF1,RALB,RAP1B,SYNJ2,TNF
Regulation of the epithelial-mesenchymal transition pathway	4.79E-07	AKT1,APC,AXIN1,BCL9,CTNNB1,FGF1,FGF11, FGF13,FGF3,FOXC2,FZD2,FZD5,FZD8,GAB1, GRB2,HGF,HMGA2,JAK1,JAK3,KRAS,MAP2K3, MAPK1,mir-8,NOTCH1,PDGFD,PIK3C2B,PIK3CD, PIK3R2,PIK3R5,PSENEN,RAF1,RALB,RAP1B,RBPJ, SMAD3,SMAD4,TCF3,TGFB2,TGFBR1,WNT4,WNT5B,WNT7A,ZEB1
IL-7 signaling pathway	5.11E-07	AKT1,BCL6,CCND1,CXCR5,EBF1,FOXO3,FYN, GRB2,HGF,JAK1,JAK3, LYN,MAPK1,MYC, NFATC1,PDPK1,PIK3C2B,PIK3CD,PIK3R2, PIK3R5,SLC2A1,STAT1,STAT5B
Glioblastoma multiforme signaling	2.04E-06	AKT1,APC,AXIN1,CCND1,CTNNB1,DIRAS3, E2F2,E2F5,FNBP1,FZD2,FZD5,FZD8,GRB2, IGF1R,ITPR1,ITPR2,KRAS,MAPK1,MYC, NF1,NOTUM,PDGFD,PIK3C2B,PIK3CD,PIK3R2, PIK3R5,PLCD1,PLCH2,RAF1,RALB,RAP1B, RHOBTB2,RHOG,TCF3,WNT4,WNT5B,WNT7A

## Discussion

In this study, we focused on investigating changes in DNA methylation during each trimester and after delivery. The findings provided supporting evidence that changes in DNA methylation during pregnancy may be important for maternal adaptation to pregnancy. Meanwhile, DNA methylation patterns during and after pregnancy were different, implying that puerperium repair may also act through DNA methylation mechanisms. In addition, different patterns of DNA methylation between the trimesters were identified in the present study. Therefore, based on our findings, studies related to DNA methylation during pregnancy should specify the time when the samples were obtained because gestational age is important for interpreting the results.

According to the IPA of the 14,018 identified CpG sites, the top canonical pathway involved is the “protein ubiquitination pathway,” which is a reversible process due to the presence of deubiquitinating enzymes that can cleave ubiquitin from modified proteins (Table 3) (23). Protein ubiquitination is essential for normal placental growth and development (24). In addition, the epigenetic regulatory role of long non-coding RNAs in the ubiquitin proteasome system and collagen remodeling may be related to spontaneous preterm labor and preterm premature rupture of membranes (25–27). TNFR2 signaling was also determined using IPA. TNF-α is also associated with inflammatory mechanisms related to implantation, placentation, and pregnancy outcomes. TNF-α is secreted by immune cells and binds TNFR1 and TNFR2. Elevated TNF-α is associated with recurrent pregnancy loss, early and severe preeclampsia, and recurrent implantation failure; all of these are “idiopathic” or related to antiphospholipid positivity, which implies the important role of TNF-α in maintaining normal pregnancy (28).

In this study, most CpG sites were in Group 9 (*N* = 8,639, 61.63%), indicating that although DNA methylation changes during pregnancy and after delivery, most of these changes were subtle during pregnancy. In Group 9, DNA methylation increased slightly after delivery. In addition, there were 4,168 (29.73%) CpG sites in Group 7 (995, 7.1%) and Group 8 (3,173, 22.64%), and significant DNA methylation changes were observed between the first and second trimesters. This is the stage of embryonic development and organogenesis (29). The results of the IPA of Groups 7 and 8 in the present study provided supporting evidence that hematologic system development and function, tissue development, tissue morphology, and hematopoiesis were the top systems and functions involved in the pregnancy. Some of these systems and functions overlap with those of Groups 1, 2, 4, and 6. In contrast, the IPA of Group 5 yielded very different results. The top five systems and functions involved were nervous system development and function, embryonic development, hair and skin development and function, humoral immune response, and urinary system development and function. Since Group 5 had a significant increase in DNA methylation between the second and third trimesters, further studies are needed to investigate the functional implications of DNA methylation changes during this period and the development and functions of these systems. The IPA results of canonical pathways involved in different groups are quite diverse. Pathways involved in glucose homeostasis were found in Groups 1, 2, 4, 6, and 8, whereas those involved in the immune system were identified in Groups 5, 6, 7, and 8. These findings suggested that changes in glucose homeostasis and the immune system during pregnancy are at least partly mediated by DNA methylation mechanisms. For example, our previous study showed that many genes had different methylation patterns in gestational diabetes mellitus (GDM) and non-GDM groups (18). In addition, hypomethylation of IL-10 in maternal blood and increased plasma concentrations of IL-10 before delivery were noted in women with gestational diabetes (30). A number of publications have identified the mechanisms through which maternal nutrition and environmental exposures, such as stress and toxic substances that alter the expression of imprinted genes during pregnancy, can influence fetal and neonatal phenotypes and susceptibility to disease development later in life (31–33). Understanding how maternal epigenomes change during different trimesters may help to investigate the pathophysiology of obstetric and fetal complications.

Pauwels et al. noted that the mean global DNA methylation percentage before pregnancy (6.89%) was significantly higher than that at 12 weeks (6.24%; *p* = 0.007), 30 weeks (6.36%; *p* = 0.04), and after delivery (6.35%; *p* = 0.04) (14). Gruzieval et al. also demonstrated that most CpG sites show a decrease in average methylation levels before, during, and immediately after pregnancy (34). In this study, the mean methylation degree of the top 1,000 CpG sites with significant changes was the highest in the postpartum period (mean β value = 0.432), followed by those in the first trimester (mean GA at 10 weeks, mean β value = 0.346, *p* < 0.001), second trimester (mean GA at 25 weeks, mean β value = 0.338, *p* < 0.001), and third trimester (mean GA at 38 weeks, mean β value = 0.338, *p* < 0.001). White et al. analyzed genome-wide differential methylation patterns in maternal leukocyte DNA in early pregnancy (*n* = 14), in the same women postpartum (*n* = 14), and age-and body mass index (BMI)-matched nulligravid women (*n* = 14) (35). White et al. identified 264 significant CpG sites, 122 (46.2%) of which were significantly different in this study (Supplementary Table 11). They identified nine genes with at least one CpG site differentially methylated in early pregnancy compared to both non-pregnancy groups (nulligravid and postpartum), which were significant at the 10% FDR(false discovery rate) level (q < 0.10). Seven of the nine genes (*NFE2L2, IL1R2, PTPRJ, SPAG4, HPR, CCIN*, and *PC*) were also differentially methylated between the mean β value of the three trimesters and the value after delivery in this study (*p* < 0.005). All seven genes showed more hypomethylation during pregnancy than after delivery, which was in agreement with White's findings. All studies showed that the degree of DNA methylation was much higher after delivery than that during pregnancy. These findings agreed with the concept that more genes are expressed during pregnancy for maternal changes and adaptation than after delivery. The findings of the present study suggested that puerperium repair may occur through DNA methylation mechanisms.

In the Born into Life study (36), Gruzieval et al. measured epigenome-wide DNA methylation in 34 women on four occasions: once before, twice during, and once 2–4 days after pregnancy (34). They identified 196 significant CpG sites, 133 (67.9%) of which showed significant methylation differences in this study (Supplementary Table 12). We compared the data from this study with the dataset GSE37722 from White et al. and the dataset GSE122408 from Gruzieva et al. There was one significant overlapping CpG site (cg24621042) in the *SERPINA1* gene, which was less methylated during pregnancy than during the non-pregnancy period in all three datasets. *SERPINA1* provides instructions for producing a protein called alpha-1 antitrypsin, which is a serine protease inhibitor (serpin). Serpins help to control several types of chemical reactions by inhibiting the activity of certain enzymes. This warrants further investigation of the physiological significance of hypomethylation in *SERPINA1* during pregnancy.

Pregnancy is an acquired risk factor of venous thromboembolism (VTE) (37). In 2020, Wang et al. first demonstrated that blood-based global DNA methylation is associated with venous thromboembolism (38). Global DNA methylation was significantly higher in patients with primary VTE than in healthy controls (median: 0.17 vs. 0.08%; *p* < 0.001). We also noted that global DNA methylation was significantly higher in pregnant patients than in postpartum patients (*p* < 0.001). VTE is a multifactorial disease influenced by both genetic and non-genetic risk factors (39). Epigenetic factors may play a role in VTE pathophysiology. Several genes in coagulation pathways, including genes for von Willebrand factors, factor VII, and factor VIII, are regulated by DNA methylation (40–42). Further studies are required to investigate the mechanisms underlying the role of DNA methylation in the pathophysiology of VTE during pregnancy.

This study has several limitations. The first is the small sample size, which makes it difficult to perform further detailed analyses. However, the present study demonstrated the concept of “DNA methylation changes during pregnancy” and provided insights on these changes. Further studies with more samples are required to present more detailed subgroup analyses. The second limitation is that all pregnant women included in the study were Han Chinese. Further studies on other ethnic groups are required to determine whether racial differences exist. Third, DNA methylation was measured in whole blood, without adjusting for leukocyte proportions. DNA from peripheral blood is a mixture of genetic substrates from various leukocyte subtypes, and variations in leukocyte proportions could confound the true epigenetic associations between phenotype and DNA methylation (34). EpiDISH software (R) was used to calculate the cell composition of each sample (Supplementary Table 13) (43). In the literature, most studies have used data on DNA methylation without adjusting for leukocyte proportion (14, 15, 44), which is similar to the present study; this makes comparisons between studies easier. In addition, we focused on dynamic changes during different trimesters, which included changes in leukocyte proportions. However, for mechanistic implications, further studies considering the role of different leukocyte ratios are required. Fourth, although we provided each pregnant woman with diet education at the first prenatal visit and recommended taking 400 to 1,000 μg of folate or folic acid per day, 1,000 mg of calcium per day and 70–90 g of protein per day throughout pregnancy, a diet diary was not maintained in this study. Further investigations of DNA methylation during pregnancy should consider diet and nutrition.

## Conclusion

In conclusion, the present study provides supporting evidence that DNA methylation patterns change in the three trimesters of pregnancy and after delivery.

## Data Availability Statement

The raw data supporting the conclusions of this article will be made available by the authors, without undue reservation.

## Ethics Statement

This study was approved by the Research Ethics Committee of the National Taiwan University Hospital (NTUH-REC No.: 201907038RINC). The patients/participants provided their written informed consent to participate in this study.

## Author Contributions

M-WL was responsible for the data collection and manuscript writing. C-YS and M-HT performed the statistical calculations. Y-YT also helped with the clinical data collection. C-NL provided his patients and planned the study. S-YL conceived the original idea and helped write the manuscript. All authors contributed to the article and approved the submitted version.

## Funding

This study was supported by a grant (109-S4466) from National Taiwan University Hospital.

## Conflict of Interest

The authors declare that the research was conducted in the absence of any commercial or financial relationships that could be construed as a potential conflict of interest.

## Publisher's Note

All claims expressed in this article are solely those of the authors and do not necessarily represent those of their affiliated organizations, or those of the publisher, the editors and the reviewers. Any product that may be evaluated in this article, or claim that may be made by its manufacturer, is not guaranteed or endorsed by the publisher.

## Supplementary Material

Supplementary Figure 1 The principle component analysis (PCA) of the 40 samples to exclude batch effect. Chip ID was shown. X-axis denotes that the first principal component explains the variability around 65% and Y-axis denotes that the second principal component explains the variability around 15.4%.

Supplementary Table 1 The plate layout of Illumina Human MethylationEPIC BeadChip Array.

Supplementary Table 2 The identified CpG cites in group 1.

Supplementary Table 3 The identified CpG cites in group 2.

Supplementary Table 4 The identified CpG cites in group 3.

Supplementary Table 5 The identified CpG cites in group 4.

Supplementary Table 6 The identified CpG cites in group 5.

Supplementary Table 7 The identified CpG cites in group 6.

Supplementary Table 8 The identified CpG cites in group 7.

Supplementary Table 9 The identified CpG cites in group 8.

Supplementary Table 10 The identified CpG cites in group 9.

Supplementary Table 11 The 122 CpG sites identified with significantly differentially methylated in pregnancy compared with non-pregnancy groups both in the White et al. and in this study.

Supplementary Table 12 The 133 CpG sites identified with significantly differentially methylated in pregnancy compared with non-pregnancy groups both in the Gruzieva et al. and in this study.

Supplementary Table 13 The estimated blood cells ratio in each sample.
