# Supplementary figures and images for: Comparison of DNA Methylation Changes Between the Gestation Period and the After-Delivery State: A Pilot Study of 10 Women
Source: Front Nutr. 2022 May 4;9:829915. doi: 10.3389/fnut.2022.829915 (PMC9116383; doi:10.3389/fnut.2022.829915)

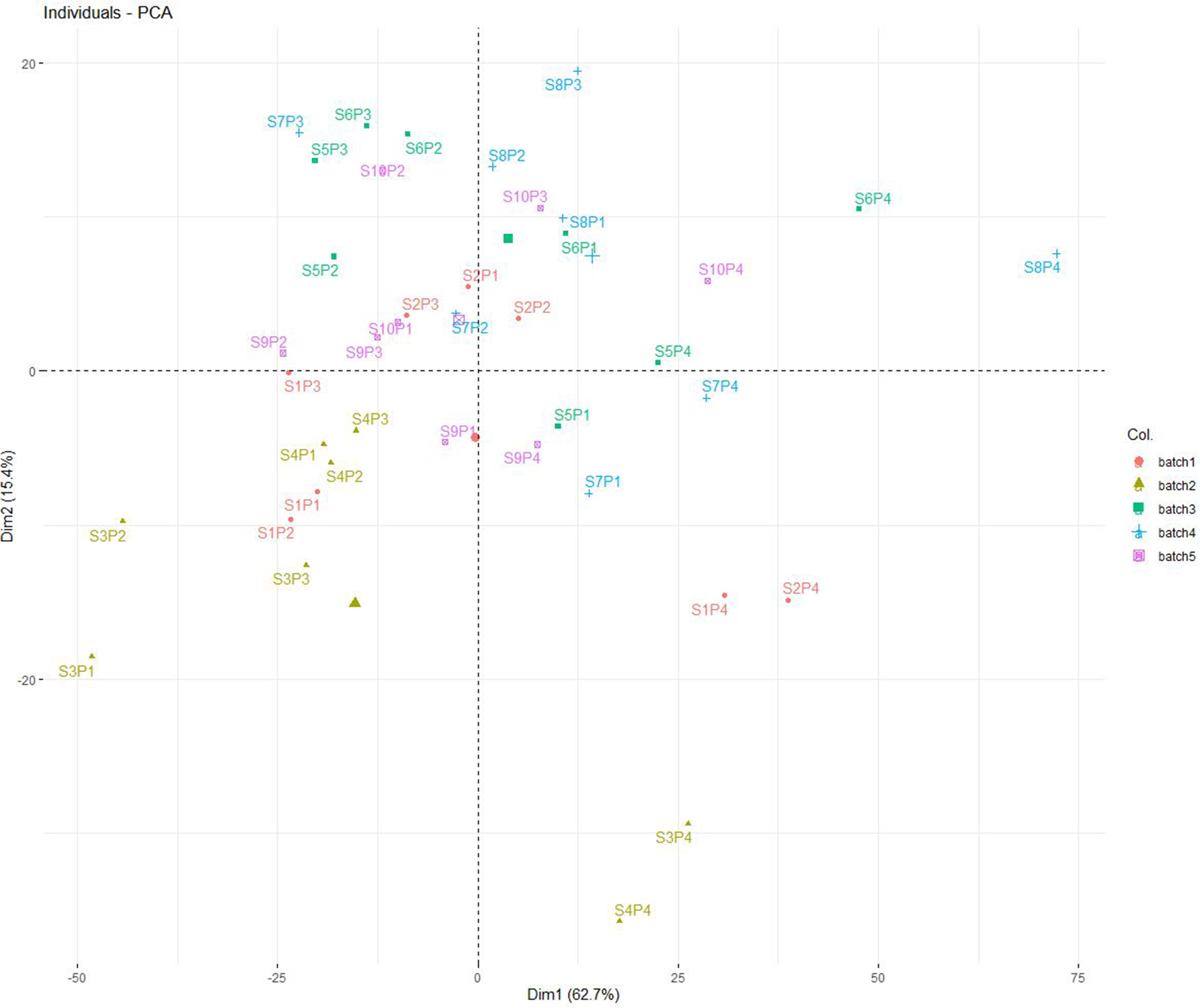

Supplement: Supplementary Figure 1 — The principle component analysis (PCA) of the 40 samples to exclude batch effect. Chip ID was shown. X-axis denotes that the first principal component explains the variability around 65% and Y-axis denotes that the second principal component explains the variability around 15.4%. [file Image_1.JPEG]
