# Supplementary material for: Comparison of DNA Methylation Changes Between the Gestation Period and the After-Delivery State: A Pilot Study of 10 Women
Source: Front Nutr. 2022 May 4;9:829915. doi: 10.3389/fnut.2022.829915 (PMC9116383; doi:10.3389/fnut.2022.829915)
Supplement: Supplementary Table 1 — The plate layout of Illumina Human MethylationEPIC BeadChip Array. [file Data_Sheet_1.docx]

# **Supplementary table 1. The plate layout of Illumina Human MethylationEPIC BeadChip Array.**

| Position | CHIP_ID | Sample ID |
| --- | --- | --- |
| 1 | 203723050004_R01C01 | 1-1 |
| 2 | 203723050004_R02C01 | 1-2 |
| 3 | 203723050004_R03C01 | 1-3 |
| 4 | 203723050004_R04C01 | 1-4 |
| 5 | 203723050004_R05C01 | 2-1 |
| 6 | 203723050004_R06C01 | 2-2 |
| 7 | 203723050004_R07C01 | 2-3 |
| 8 | 203723050004_R08C01 | 2-4 |
| 1 | 203723060026_R01C01 | 3-1 |
| 2 | 203723060026_R02C01 | 3-2 |
| 3 | 203723060026_R03C01 | 3-3 |
| 4 | 203723060026_R04C01 | 3-4 |
| 5 | 203723060026_R05C01 | 4-1 |
| 6 | 203723060026_R06C01 | 4-2 |
| 7 | 203723060026_R07C01 | 4-3 |
| 8 | 203723060026_R08C01 | 4-4 |
| 1 | 204391650104_R01C01 | 5-1 |
| 2 | 204391650104_R02C01 | 5-2 |
| 3 | 204391650104_R03C01 | 5-3 |
| 4 | 204391650104_R04C01 | 5-4 |
| 5 | 204391650104_R05C01 | 6-1 |
| 6 | 204391650104_R06C01 | 6-2 |
| 7 | 204391650104_R07C01 | 6-3 |
| 8 | 204391650104_R08C01 | 6-4 |
| 1 | 204391650142_R01C01 | 7-1 |
| 2 | 204391650142_R02C01 | 7-2 |
| 3 | 204391650142_R03C01 | 7-3 |
| 4 | 204391650142_R04C01 | 7-4 |
| 5 | 204391650142_R05C01 | 8-1 |
| 6 | 204391650142_R06C01 | 8-2 |
| 7 | 204391650142_R07C01 | 8-3 |
| 8 | 204391650142_R08C01 | 8-4 |
| 1 | 204391650166_R01C01 | 9-1 |
| 2 | 204391650166_R02C01 | 9-2 |
| 3 | 204391650166_R03C01 | 9-3 |
| 4 | 204391650166_R04C01 | 9-4 |
| 5 | 204391650166_R05C01 | 10-1 |
| 6 | 204391650166_R06C01 | 10-2 |
| 7 | 204391650166_R07C01 | 10-3 |
| 8 | 204391650166_R08C01 | 10-4 |

1-1 stands for sample 1 in the first trimester. 1-2 stands for sample 1 in the 2nd trimester. 1-3 stands for sample 1 in the 3rd trimester. 1-4 stands for sample 1 in the after-delivery status and so on.

# **Supplementary table 2.** **The identified CpG cites in group 1.**

| Target ID | p-value | Chromosome | SNP_ID | mean beta value in the 1st trimester | mean beta value in the 2nd trimester | mean beta value in the 3rd trimester | mean beta value in the after-delivery status |
| --- | --- | --- | --- | --- | --- | --- | --- |
| cg11099072 | 9.62E-04 | 1 | rs536459465 | 0.633156 | 0.666475 | 0.69025 | 0.611042 |
| cg12287469 | 9.35E-04 | 4 | rs113650083 | 0.617526 | 0.647542 | 0.672268 | 0.658115 |
| cg13628057 | 9.30E-04 | 9 | rs529680259; rs540190418; rs560371461 | 0.686294 | 0.722122 | 0.756158 | NA |
| cg13279847 | 8.40E-04 | 10 | rs192469505 | 0.617953 | 0.638339 | 0.659695 | 0.580701 |
| cg14381840 | 8.33E-04 | 4 | rs72987030 | 0.683211 | 0.721409 | 0.746085 | 0.644016 |
| cg21753092 | 8.05E-04 | 2 |  | 0.48459 | 0.509302 | 0.533415 | 0.460496 |
| cg12143417 | 6.93E-04 | 12 | rs117690785; rs78858857 | 0.649613 | 0.670912 | 0.700357 | 0.643256 |
| cg01887280 | 6.46E-04 | 7 | rs2299945; rs562650170 | 0.707196 | 0.734031 | 0.761753 | 0.695037 |
| cg08080122 | 6.45E-04 | 5 | rs538057335; rs150356263; rs578004203 | 0.601616 | 0.629972 | 0.658455 | 0.580499 |
| cg26917727 | 6.31E-04 | 3 | rs534911647 | 0.725917 | 0.75222 | 0.772788 | 0.726027 |
| cg26647607 | 5.91E-04 | 3 | rs570196702 | 0.669487 | 0.693216 | 0.715489 | 0.643159 |
| cg15207883 | 4.84E-04 | 20 | rs6076192; rs529171330;rs549323796; rs559702984; rs527649961; rs187421098 | 0.749946 | 0.77054 | 0.793684 | 0.738206 |
| cg06201228 | 4.66E-04 | 12 |  | 0.70059 | 0.722494 | 0.746749 | 0.710923 |
| cg06805844 | 4.44E-04 | 1 |  | 0.55912 | 0.593308 | 0.621032 | 0.557203 |
| cg04544473 | 4.39E-04 | 4 | rs530977815; rs4696131; rs535535577 | 0.433858 | 0.462196 | 0.487118 | 0.382346 |
| cg09870892 | 4.07E-04 | 14 | rs561990200; rs529436477; rs547519064 | 0.524902 | 0.549042 | 0.574852 | 0.52563 |
| cg26705250 | 3.93E-04 | 13 | rs563444532; rs77231984 | 0.678945 | 0.704802 | 0.72541 | 0.642962 |
| cg03364760 | 3.92E-04 | 1 | rs114684145 | 0.505547 | 0.536738 | 0.564178 | 0.47365 |
| cg11578977 | 3.80E-04 | 6 | rs569328945; rs148807339; rs188680611; rs574443781 | 0.704309 | 0.734784 | 0.756338 | 0.666819 |
| cg23242220 | 3.62E-04 | 19 | rs540608233; rs34883528; rs142087395 | 0.601591 | 0.625748 | 0.647015 | 0.569355 |
| cg16007153 | 3.33E-04 | 17 | rs371082950; rs572389860; rs542671454; rs561210737 | 0.612028 | 0.632618 | 0.654196 | 0.588797 |
| cg21380264 | 3.01E-04 | 21 | rs569661619; rs530505589; rs2243498 | 0.807137 | 0.829431 | 0.851191 | 0.842655 |
| cg24056580 | 2.93E-04 | 8 |  | 0.709728 | 0.733866 | 0.758066 | 0.667682 |
| cg04744660 | 2.52E-04 | 1 | rs587653040; rs587710076 | 0.629202 | 0.656236 | 0.677517 | 0.619414 |
| cg08284097 | 2.19E-04 | 12 | rs138568156 | 0.562926 | 0.585147 | 0.614668 | 0.534673 |
| cg08268031 | 1.87E-04 | 1 |  | 0.648694 | 0.673174 | 0.694443 | 0.6203 |
| cg20874031 | 1.82E-04 | 2 |  | 0.596261 | 0.62219 | 0.6526 | 0.572348 |
| cg22930549 | 1.78E-04 | 14 | rs562439904 | 0.740921 | 0.764559 | 0.787118 | 0.734363 |
| cg05390091 | 1.73E-04 | 4 | rs140365507; rs535967393; rs181779154 | 0.750614 | 0.77368 | 0.79987 | 0.738079 |
| cg18203393 | 1.51E-04 | 18 | rs189427294 | 0.757202 | 0.779915 | 0.801578 | 0.721002 |
| cg26196760 | 1.16E-04 | 18 | rs540523021; rs633215 | 0.748571 | 0.775194 | 0.796245 | 0.728245 |
| cg08309687 | 1.05E-04 | 21 |  | 0.493199 | 0.515352 | 0.536598 | 0.482342 |
| cg24310395 | 8.13E-05 | 3 | rs558041553; rs56373695; rs190102212 | 0.634377 | 0.664679 | 0.698175 | 0.594727 |
| cg25153678 | 7.55E-05 | 13 |  | 0.64729 | 0.669816 | 0.714581 | 0.607563 |
| cg18537456 | 5.80E-05 | 6 | rs527295488; rs542646072 | 0.656402 | 0.687111 | 0.710285 | 0.594503 |
| cg09586927 | 5.34E-05 | 18 | rs184540223 | 0.701771 | 0.727956 | 0.754424 | 0.660792 |
| cg06127316 | 3.84E-05 | 11 | rs550058463; rs143222443 | 0.711657 | 0.734113 | 0.772714 | 0.678783 |
| cg23489671 | 2.67E-05 | 10 | rs10796203 | 0.392669 | 0.421635 | 0.442035 | 0.390558 |
| cg02401786 | 2.58E-05 | 17 |  | 0.435773 | 0.463828 | 0.492686 | 0.414922 |
| cg02619834 | 1.64E-05 | 10 |  | 0.638929 | 0.659468 | 0.684299 | 0.7265 |
| cg26277237 | 7.61E-06 | 9 | rs191612664; rs558613543; rs183755306; rs534732306 | 0.470603 | 0.519146 | 0.550121 | 0.439218 |
| cg17820085 | 6.55E-06 | 2 | rs569544675 | 0.686634 | 0.721408 | 0.750607 | 0.66975 |
| cg07721409 | 2.62E-07 | 10 | rs140652578; rs182421356 | 0.447132 | 0.480203 | 0.507838 | 0.413158 |

# **Supplementary table 3. The identified CpG cites in group 2.**

| Target ID | p-value | Chromosome | SNP_ID | mean beta value in the 1st trimester | mean beta value in the 2nd trimester | mean beta value in the 3rd trimester | mean beta value in the after-delivery status |
| --- | --- | --- | --- | --- | --- | --- | --- |
| cg12903948 | 9.34E-04 | 9 | rs558899952; rs572344815 | 0.305566 | 0.275317 | 0.242009 | 0.392878 |
| cg06633438 | 7.74E-04 | 19 | rs113020498; rs560685148; rs529725398; rs546354365; rs371374375; rs532256699 | 0.331436 | 0.300033 | 0.269566 | 0.409849 |
| cg11845168 | 7.27E-04 | 13 | rs150550153; rs139931698; rs538987782 | 0.252096 | 0.228451 | 0.208297 | 0.314073 |
| cg03187568 | 7.07E-04 | 21 | rs115125512; rs188882210 | 0.272086 | 0.250404 | 0.229646 | 0.339549 |
| cg20740903 | 7.00E-04 | 17 | rs528150626 | 0.35304 | 0.327707 | 0.304255 | 0.411888 |
| cg20896049 | 6.91E-04 | 12 | rs116436032; rs190240750; rs541024841; rs560821837 | 0.320529 | 0.288792 | 0.251353 | 0.40408 |
| cg01879591 | 5.83E-04 | 2 |  | 0.369341 | 0.335406 | 0.315324 | 0.433664 |
| cg17374310 | 5.76E-04 | 2 | rs114763784; rs551392629 | 0.307663 | 0.279987 | 0.247193 | 0.389604 |
| cg01366462 | 5.31E-04 | 4 | rs567576700 | 0.258492 | 0.237716 | 0.217315 | 0.337484 |
| cg19394039 | 4.75E-04 | 6 | rs547347308; rs572139284 | 0.310639 | 0.287271 | 0.264101 | 0.383478 |
| cg04253214 | 4.57E-04 | 1 | rs189958707; rs78579376; rs140698185 | 0.480226 | 0.434891 | 0.394152 | 0.544318 |
| cg16005573 | 4.28E-04 | 3 | rs561406522; rs530636288 | 0.338479 | 0.313993 | 0.285677 | 0.420109 |
| cg00840791 | 3.95E-04 | 19 | rs143218423; rs7258756; rs185828119; rs569299490; rs538581004; rs558410479 | 0.411943 | 0.340218 | 0.316579 | 0.451653 |
| cg22550512 | 3.57E-04 | 11 | rs75172493;rs78551945 | 0.278612 | 0.243796 | 0.223053 | 0.316496 |
| cg06545761 | 3.53E-04 | 16 | rs552648487; rs118035774 | 0.487198 | 0.447131 | 0.424931 | 0.546667 |
| cg12691572 | 3.37E-04 | 10 | rs567741850; rs537901923 | 0.286704 | 0.253809 | 0.230556 | 0.333763 |
| cg21035962 | 3.20E-04 | 13 |  | 0.278787 | 0.253882 | 0.229459 | 0.348228 |
| cg13471188 | 3.17E-04 | 2 | rs570396905 | 0.264951 | 0.23952 | 0.218942 | 0.330088 |
| cg10357682 | 2.97E-04 | 20 | rs571666353; rs112936229; rs142368377; rs74848036; rs181207201; rs6107341 | 0.336394 | 0.306857 | 0.281492 | 0.407843 |
| cg19430303 | 2.81E-04 | 14 |  | 0.244529 | 0.223108 | 0.202618 | 0.338447 |
| cg10764214 | 2.70E-04 | 7 | rs138842016 | 0.261198 | 0.234655 | 0.212755 | 0.332889 |
| cg08998950 | 2.55E-04 | 3 | rs146028585; rs711631 | 0.379231 | 0.354027 | 0.332016 | 0.47057 |
| cg14287688 | 2.50E-04 | 2 | rs574895127; rs145250677 | 0.30686 | 0.28386 | 0.259464 | 0.385719 |
| cg07227049 | 1.49E-04 | 2 | rs547751361; rs536299562; rs554664305 | 0.342488 | 0.312716 | 0.291803 | 0.434638 |
| cg16713785 | 1.34E-04 | 12 | rs146986879; rs138360236; rs531759683 | 0.276743 | 0.255232 | 0.235113 | 0.365482 |
| cg13596415 | 1.30E-04 | 8 | rs530423508; rs548571309; rs567268353 | 0.262929 | 0.235991 | 0.215536 | 0.346738 |
| cg06066700 | 9.04E-05 | 2 | rs74855027; rs558020328; rs578130303; rs540535374 | 0.240121 | 0.212579 | 0.192272 | 0.303128 |
| cg25585899 | 5.77E-05 | 18 |  | 0.294411 | 0.264581 | 0.2415 | 0.381454 |
| cg01058360 | 4.52E-05 | 7 | rs537709008; rs563160114 | 0.307861 | 0.286645 | 0.2575 | 0.398516 |
| cg08429353 | 4.43E-05 | 3 | rs76913836; rs147799983 | 0.263613 | 0.241563 | 0.218219 | 0.340342 |
| cg26873329 | 3.70E-05 | 5 | rs548840673; rs146507600; rs187638431; rs550914410 | 0.38782 | 0.365726 | 0.343844 | 0.472123 |
| cg21027759 | 2.40E-05 | 1 |  | 0.281624 | 0.254802 | 0.229023 | 0.371302 |
| cg18320790 | 2.00E-05 | 15 |  | 0.304463 | 0.278562 | 0.254968 | 0.392256 |
| cg16390637 | 1.94E-05 | 20 | rs640415; rs549877347 | 0.290301 | 0.257558 | 0.23553 | 0.359533 |
| cg01179618 | 1.54E-05 | 8 | rs147791903; rs560832961; rs115407559; rs549532236; rs55843815 | 0.340265 | 0.312582 | 0.287805 | 0.417729 |
| cg05186879 | 1.29E-05 | 3 | rs144540136; rs552979786 | 0.559995 | 0.533532 | 0.510619 | 0.60706 |
| cg11224904 | 1.93E-06 | 13 | rs570943372 | 0.405102 | 0.378396 | 0.354929 | 0.465632 |
| cg16149992 | 3.79E-07 | 14 | rs115048907; rs78788859 | 0.366399 | 0.341539 | 0.318582 | 0.436622 |
| cg04014328 | 5.94E-08 | 17 | rs114420588 | 0.618673 | 0.573656 | 0.551766 | 0.688272 |

# **Supplementary table 4. The identified CpG cites in group 3.**

| Target ID | p-value | Chromosome | SNP_ID | mean beta value in the 1st trimester | mean beta value in the 2nd trimester | mean beta value in the 3rd trimester | mean beta value in the after-delivery status |
| --- | --- | --- | --- | --- | --- | --- | --- |
| cg07275068 | 9.02E-04 | 22 |  | 0.757185 | 0.784449 | 0.761662 | 0.716832 |
| cg07099303 | 7.82E-04 | 17 | rs567993923; rs150109122; rs376557911 | 0.614657 | 0.646554 | 0.615422 | 0.532035 |
| cg24018650 | 7.37E-04 | 2 | rs182834669; rs541217549 | 0.761072 | 0.793502 | 0.771489 | 0.748397 |
| cg09597756 | 6.92E-04 | 14 | rs8003105; rs151009200; rs6574138 | 0.64996 | 0.694552 | 0.672916 | 0.60788 |
| cg05314055 | 6.08E-04 | 5 | rs561556992; rs528706219 | 0.744717 | 0.780681 | 0.744633 | 0.691711 |
| cg22129220 | 5.38E-04 | 6 | rs576105731; rs546587732; rs114483550; rs111391270; rs541356700; rs564225133 | 0.487053 | 0.541328 | 0.51843 | 0.474237 |
| cg15831456 | 5.08E-04 | X |  | 0.396295 | 0.41634 | 0.386863 | 0.390643 |
| cg06196398 | 8.20E-05 | 2 | rs534214096; rs72976373; rs151320983 | 0.536691 | 0.561518 | 0.536341 | 0.474817 |
| cg14438279 | 6.22E-06 | 5 |  | 0.739411 | 0.78921 | 0.767129 | 0.707116 |

# **Supplementary table 5. The identified CpG cites in group 4.**

| Target ID | p-value | Chromosome | SNP_ID | mean beta value in the 1st trimester | mean beta value in the 2nd trimester | mean beta value in the 3rd trimester | mean beta value in the after-delivery status |
| --- | --- | --- | --- | --- | --- | --- | --- |
| cg24463437 | 9.70E-04 | 1 | rs560288541; rs532149581; rs545892234 | 0.375553 | 0.337518 | 0.361624 | 0.433195 |
| cg16961070 | 9.61E-04 | 7 | rs561335661; rs530467622; rs550837250; rs564408513 | 0.690422 | 0.670197 | 0.710189 | 0.74377 |
| cg22707582 | 9.20E-04 | 11 | rs550237991; rs117856605; rs201617739 | 0.67403 | 0.650271 | 0.676044 | 0.723533 |
| cg23208557 | 9.19E-04 | 4 | rs142044397 | 0.494352 | 0.463227 | 0.489155 | 0.570426 |
| cg09289001 | 9.10E-04 | 8 | rs28927984 | 0.178116 | 0.157159 | 0.17902 | 0.256232 |
| cg04966289 | 9.08E-04 | 14 | rs577130865; rs151282006 | 0.42836 | 0.393447 | 0.414653 | 0.493067 |
| cg06988251 | 8.78E-04 | 1 | rs180943997; rs371298884; rs540276050 | 0.474921 | 0.452758 | 0.476468 | 0.561433 |
| cg18693051 | 8.46E-04 | 19 | rs182211370 | 0.647671 | 0.612691 | 0.640562 | 0.709304 |
| cg24780185 | 8.09E-04 | 10 | rs556198321 | 0.455858 | 0.41145 | 0.457925 | 0.51302 |
| cg06581979 | 7.82E-04 | 4 | rs527368522; rs577630848 | 0.701372 | 0.675903 | 0.707413 | 0.754506 |
| cg01897901 | 7.54E-04 | 6 | rs187234721; rs191066394; rs182096428 | 0.735162 | 0.702597 | 0.736491 | 0.770805 |
| cg18892446 | 7.52E-04 | 5 |  | 0.419131 | 0.397456 | 0.4263 | 0.496696 |
| cg06068625 | 7.37E-04 | 2 | rs577962234; rs184231405 | 0.691191 | 0.657397 | 0.679248 | 0.717884 |
| cg13739468 | 7.05E-04 | 2 | rs577258487 | 0.401417 | 0.359049 | 0.380464 | 0.476417 |
| cg09838115 | 6.99E-04 | 7 | rs558111733 | 0.846937 | 0.821338 | 0.849384 | 0.827264 |
| cg18146370 | 6.99E-04 | 1 | rs556132978 | 0.482506 | 0.461457 | 0.488103 | 0.564353 |
| cg02769743 | 6.97E-04 | 1 | rs563273953 | 0.607363 | 0.58659 | 0.612502 | 0.66439 |
| cg18146146 | 6.53E-04 | 19 |  | 0.683527 | 0.644166 | 0.666767 | 0.744495 |
| cg01752594 | 6.41E-04 | 13 |  | 0.350229 | 0.317192 | 0.338801 | 0.423952 |
| cg03351508 | 6.30E-04 | 21 |  | 0.557025 | 0.528049 | 0.558056 | 0.642583 |
| cg26146808 | 6.29E-04 | 20 | rs540246943 | 0.439388 | 0.417198 | 0.441257 | 0.51415 |
| cg20253855 | 5.78E-04 | 7 |  | 0.69717 | 0.674278 | 0.699097 | 0.760853 |
| cg05673041 | 5.63E-04 | 17 | rs527265610 | 0.356315 | 0.310401 | 0.330601 | 0.425934 |
| cg25700339 | 5.60E-04 | 6 | rs763122; rs545741515; rs564326271 | 0.732719 | 0.708547 | 0.729862 | 0.772982 |
| cg03293239 | 5.59E-04 | 12 | rs201268842; rs113800499; rs547499854; rs373121727 | 0.594912 | 0.574906 | 0.598222 | 0.665322 |
| cg03754700 | 5.57E-04 | 12 | rs573796850; rs542443732; rs553278189; rs573043189; rs117087653; rs564541249; rs530393568; rs147746512 | 0.768336 | 0.746907 | 0.782102 | 0.811428 |
| cg20889490 | 5.57E-04 | 18 |  | 0.399056 | 0.366836 | 0.396659 | 0.49373 |
| cg08286700 | 5.54E-04 | 2 | rs141212440; rs150208592; rs2288100 | 0.487404 | 0.466238 | 0.493079 | 0.584584 |
| cg18084389 | 5.51E-04 | 5 | rs552471359; rs141075167; rs569155027; rs531773151 | 0.644165 | 0.610278 | 0.636081 | 0.704577 |
| cg12449837 | 4.95E-04 | 22 | rs566895923; rs544982381; rs549218742 | 0.703884 | 0.680082 | 0.709429 | 0.755095 |
| cg10438624 | 4.75E-04 | 2 | rs533912865; rs554258139 | 0.371249 | 0.341814 | 0.371063 | 0.460966 |
| cg19619956 | 4.70E-04 | 5 |  | 0.38236 | 0.351355 | 0.371609 | 0.467448 |
| cg18436808 | 3.99E-04 | 9 |  | 0.334221 | 0.292466 | 0.319702 | 0.427509 |
| cg16495735 | 3.96E-04 | 2 | rs547496971; rs7594031; rs539642990; rs141563179; rs570317185 | 0.406276 | 0.384989 | 0.418946 | 0.493199 |
| cg21964148 | 3.80E-04 | 12 | rs551718127; rs571957178; rs148314754 | 0.241158 | 0.219853 | 0.253187 | 0.341717 |
| cg11337628 | 3.65E-04 | 3 | rs114516853; rs530134399 | 0.500169 | 0.46638 | 0.490998 | 0.569175 |
| cg04502852 | 3.57E-04 | 8 | rs572434987; rs150742187 | 0.360896 | 0.339267 | 0.359959 | NA |
| cg01297684 | 3.57E-04 | 12 | rs577549467; rs372016539 | 0.561433 | 0.510522 | 0.534224 | 0.66211 |
| cg13276306 | 3.44E-04 | 8 | rs375128138; rs80058562 | 0.475103 | 0.447135 | 0.476523 | 0.541626 |
| cg23601597 | 3.43E-04 | 20 | rs569634493; rs188845307; rs555185942 | 0.720099 | 0.695589 | 0.72142 | 0.754638 |
| cg26256135 | 3.42E-04 | 10 | rs531245398; rs71485519 | 0.64174 | 0.620062 | 0.641313 | 0.699906 |
| cg02813721 | 3.41E-04 | 8 | rs551476100; rs571471039 | 0.410417 | 0.369136 | 0.392172 | 0.49968 |
| cg02962647 | 3.39E-04 | 6 | rs188190949; rs559412896; rs533103979 | 0.64521 | 0.618178 | 0.656006 | 0.686716 |
| cg11525409 | 3.30E-04 | 2 | rs574610861; rs141198629; rs563170532 | 0.345098 | 0.317532 | 0.339951 | 0.433308 |
| cg17515347 | 3.11E-04 | 1 | rs565259184; rs180763959; rs551058284; rs569092412 | 0.409197 | 0.372646 | 0.398779 | 0.506858 |
| cg05602501 | 3.00E-04 | 13 | rs544919764 | 0.755169 | 0.733909 | 0.756087 | 0.783295 |
| cg21668714 | 2.90E-04 | 16 | rs564026031; rs201005851; rs79752925; rs146746669; rs201639174 | 0.478081 | 0.456565 | 0.479743 | 0.563485 |
| cg17803713 | 2.89E-04 | 2 | rs368169104; rs550989213 | 0.47796 | 0.45014 | 0.471673 | 0.523043 |
| cg13258453 | 2.72E-04 | 6 | rs556678964 | 0.528831 | 0.487074 | 0.511714 | 0.633045 |
| cg09793892 | 2.66E-04 | 1 | rs150325233 | 0.438265 | 0.418028 | 0.438446 | 0.516333 |
| cg26729947 | 2.49E-04 | 16 | rs111333647; rs542504099 | 0.620551 | 0.600046 | 0.625603 | 0.662421 |
| cg04936951 | 2.36E-04 | 5 | rs62398528; rs557778429; rs575899327; rs537160793 | 0.408654 | 0.388098 | 0.412744 | 0.466253 |
| cg24229188 | 2.28E-04 | 19 | rs554162055; rs199835191 | 0.791512 | 0.763587 | 0.786754 | 0.814274 |
| cg03242834 | 2.21E-04 | 19 | rs117032202; rs538000101; rs556329313; rs575623312 | 0.606763 | 0.584214 | 0.60683 | 0.66033 |
| cg01771885 | 2.20E-04 | 20 | rs114556997; rs139296980; rs554858154 | 0.692368 | 0.670988 | 0.693287 | 0.71296 |
| cg09069499 | 2.16E-04 | 8 | rs577442532; rs547272796; rs144741713 | 0.417572 | 0.391242 | 0.411952 | 0.502635 |
| cg13731776 | 1.99E-04 | 9 | rs557165894; rs575642230 | 0.675326 | 0.650477 | 0.682004 | 0.722182 |
| cg22798984 | 1.89E-04 | 14 | rs559354370; rs28376820 | 0.287778 | 0.2623 | 0.283588 | 0.237812 |
| cg01573140 | 1.86E-04 | 7 | rs557866539; rs566433879; rs534039420 | 0.602988 | 0.582323 | 0.607455 | 0.665333 |
| cg17149984 | 1.83E-04 | 3 | rs550165931 | 0.375836 | 0.338049 | 0.365612 | 0.453485 |
| cg15805490 | 1.61E-04 | 19 | rs372903611; rs61648096; rs540237413; rs72974801 | 0.622118 | 0.600892 | 0.62798 | 0.669647 |
| cg10009284 | 1.56E-04 | 6 | rs575745377; rs544807834; rs564973285 | 0.606142 | 0.575115 | 0.606246 | 0.65282 |
| cg07769015 | 1.53E-04 | 8 | rs552993488; rs574517980; rs541886379 | 0.404245 | 0.373589 | 0.39788 | NA |
| cg00504274 | 1.50E-04 | 13 | rs540972325 | 0.469627 | 0.446469 | 0.469224 | 0.530371 |
| cg18050741 | 1.45E-04 | 19 | rs552383833; rs564355127 | 0.396835 | 0.356601 | 0.379398 | 0.469184 |
| cg25381253 | 1.38E-04 | 5 | rs79612977; rs186518025 | 0.575405 | 0.553643 | 0.575456 | 0.635807 |
| cg05140089 | 1.37E-04 | 21 | rs139504333 | 0.602334 | 0.578428 | 0.59936 | 0.659901 |
| cg08084228 | 1.36E-04 | 16 |  | 0.60904 | 0.579979 | 0.607765 | 0.676292 |
| cg20018806 | 1.19E-04 | 11 | rs535420517 | 0.526399 | 0.500106 | 0.537566 | 0.616416 |
| cg06266097 | 8.88E-05 | 11 | rs537765768; rs77301402; rs567919498; rs533793333 | 0.649196 | 0.618475 | 0.643913 | 0.684922 |
| cg26202404 | 8.76E-05 | 5 |  | 0.498175 | 0.466453 | 0.48832 | 0.555293 |
| cg25957332 | 8.07E-05 | 20 | rs541627200; rs568472599 | 0.611432 | 0.586483 | 0.610238 | 0.664459 |
| cg12375635 | 7.98E-05 | 11 | rs147248160; rs528477428; rs554015237; rs551839048 | 0.572781 | 0.547378 | 0.569138 | 0.650432 |
| cg11399195 | 7.86E-05 | 1 | rs553440851; rs41274462; rs542546734 | 0.646059 | 0.62099 | 0.642494 | 0.703288 |
| cg09450087 | 7.44E-05 | 20 |  | 0.665889 | 0.641491 | 0.66525 | 0.721886 |
| cg05967487 | 7.22E-05 | 11 | rs576619348 | 0.667204 | 0.627176 | 0.648116 | 0.696553 |
| cg05194248 | 6.72E-05 | 19 | rs114142682 | 0.552747 | 0.532383 | 0.565499 | 0.614108 |
| cg03074946 | 6.49E-05 | 12 | rs73415041; rs569658358 | 0.531464 | 0.500459 | 0.522337 | 0.571062 |
| cg13178755 | 6.41E-05 | 2 | rs543702996; rs562165703 | 0.429495 | 0.384447 | 0.413155 | 0.531008 |
| cg22608800 | 6.41E-05 | 8 | rs190081747; rs59795434 | 0.634675 | 0.61156 | 0.637902 | 0.688065 |
| cg04454114 | 6.01E-05 | 17 | rs550500774; rs570410308 | 0.68993 | 0.667083 | 0.692299 | 0.772083 |
| cg09984738 | 5.63E-05 | 10 | rs75672933 | 0.3684 | 0.343644 | 0.369428 | 0.43524 |
| cg03834968 | 5.32E-05 | 8 | rs10096078; rs142276729 | 0.385854 | 0.355232 | 0.386606 | 0.487212 |
| cg19169208 | 5.07E-05 | 10 |  | 0.528083 | 0.501841 | 0.523215 | 0.603463 |
| cg15559252 | 4.90E-05 | 1 | rs554149617 | 0.419864 | 0.396591 | 0.427389 | 0.495916 |
| cg04602396 | 4.74E-05 | 19 | rs191719246; rs150714690 | 0.485513 | 0.462217 | 0.491341 | 0.579325 |
| cg22421019 | 4.46E-05 | 1 | rs137900312 | 0.394748 | 0.369954 | 0.391181 | 0.493519 |
| cg16449835 | 4.42E-05 | 22 | rs184916331 | 0.759738 | 0.737736 | 0.758987 | 0.800612 |
| cg10027857 | 4.33E-05 | 17 | rs528408734; rs543751933; rs147504842; rs74576794 | 0.672422 | 0.644201 | 0.665459 | 0.71954 |
| cg19613776 | 4.15E-05 | 15 | rs569689026 | 0.554818 | 0.534645 | 0.560039 | 0.646515 |
| cg13751307 | 3.88E-05 | 10 | rs540406821 | 0.655789 | 0.633785 | 0.656906 | 0.714305 |
| cg04897607 | 3.25E-05 | 18 | rs188005387; rs145083458 | 0.456391 | 0.415332 | 0.444602 | 0.562646 |
| cg07851983 | 3.13E-05 | 8 |  | 0.602247 | 0.565594 | 0.593649 | 0.666554 |
| cg10850338 | 2.83E-05 | 9 |  | 0.651112 | 0.629558 | 0.650937 | 0.71463 |
| cg07172755 | 2.40E-05 | 10 | rs61862634; rs1530765 | 0.523822 | 0.489223 | 0.510371 | 0.594851 |
| cg07402683 | 2.38E-05 | 12 |  | 0.555254 | 0.534413 | 0.55682 | 0.612119 |
| cg12921549 | 2.23E-05 | 3 | rs540903488; rs559142283; rs140482943 | 0.618763 | 0.586194 | 0.615457 | 0.684057 |
| cg06092244 | 2.13E-05 | 2 | rs372018226; rs543260539 | 0.696656 | 0.668144 | 0.69328 | 0.796358 |
| cg21101086 | 2.03E-05 | 2 | rs181574804; rs548745742 | 0.489565 | 0.466038 | 0.488432 | 0.579133 |
| cg14651126 | 1.28E-05 | 15 | rs193174422; rs142333855 | 0.594545 | 0.570263 | 0.59388 | 0.672845 |
| cg23941688 | 1.10E-05 | 17 | rs2108948; rs531784335; rs61194086 | 0.663318 | 0.638807 | 0.660795 | 0.723862 |
| cg00898064 | 1.09E-05 | 3 | rs148397439 | 0.456018 | 0.423664 | 0.446903 | 0.566676 |
| cg04487595 | 9.65E-06 | 1 | rs541642624; rs553559272; rs578055175; rs113085184 | 0.615981 | 0.581651 | 0.60784 | 0.659082 |
| cg12644285 | 8.16E-06 | 15 | rs574039865; rs58542701; rs559846908 | 0.451996 | 0.428873 | 0.449515 | 0.561106 |
| cg11731283 | 7.91E-06 | 7 | rs192012630; rs139845616 | 0.682605 | 0.659743 | 0.684306 | 0.755223 |
| cg20497667 | 7.49E-06 | 3 | rs571453697; rs6443996 | 0.737207 | 0.710342 | 0.740241 | 0.772214 |
| cg05037877 | 6.52E-06 | 10 | rs74676734; rs567473451; rs112753268 | 0.44366 | 0.402758 | 0.428366 | 0.528887 |
| cg14546840 | 4.79E-06 | 3 | rs541339925 | 0.447183 | 0.423334 | 0.445895 | 0.535505 |
| cg21879479 | 4.76E-06 | 3 |  | 0.535201 | 0.511677 | 0.533864 | 0.621114 |
| cg02761839 | 3.74E-06 | 2 | rs377076951 | 0.46599 | 0.429587 | 0.45789 | 0.588508 |
| cg03986427 | 3.53E-06 | 19 | rs527932611 | 0.647795 | 0.621004 | 0.642546 | 0.72143 |
| cg22877129 | 3.20E-06 | 1 | rs183003901; rs1774902 | 0.547899 | 0.518352 | 0.548551 | 0.647953 |
| cg14887853 | 7.68E-07 | 6 |  | 0.532547 | 0.499452 | 0.526361 | 0.644684 |
| cg01471929 | 6.40E-07 | 16 | rs116902295; rs559797729 | 0.526122 | 0.500648 | 0.530999 | 0.656202 |
| cg10163234 | 2.42E-07 | 1 | rs555090603; rs151308916; rs536435837; rs140559734 | 0.649101 | 0.62562 | 0.65094 | 0.710587 |
| cg25583608 | 1.98E-08 | 6 | rs73550694; rs190190979; rs561755239; rs73550697 | 0.659396 | 0.629721 | 0.652586 | 0.764042 |
| cg09897881 | 1.76E-08 | 15 | rs553551555 | 0.599078 | 0.574531 | 0.59605 | 0.688877 |

# **Supplementary table 6. The identified CpG cites in group 5.**

| Target ID | p-value | Chromosome | SNP_ID | mean beta value in the 1st trimester | mean beta value in the 2nd trimester | mean beta value in the 3rd trimester | mean beta value in the after-delivery status |
| --- | --- | --- | --- | --- | --- | --- | --- |
| cg17915189 | 9.86E-04 | 7 | rs537539653 | 0.594497 | 0.597897 | 0.621641 | 0.653514 |
| cg16950658 | 9.84E-04 | 12 |  | 0.615765 | 0.618834 | 0.644824 | 0.678139 |
| cg20708305 | 9.79E-04 | 3 | rs567117876;rs534651498; rs9874107; rs149287508; rs41380946; rs557150738;rs575395912; rs546155883 | 0.681813 | 0.675346 | 0.700416 | 0.742418 |
| cg19544466 | 9.77E-04 | 20 | rs148773321;rs546009454; rs142379160 | 0.695307 | 0.711516 | 0.731889 | 0.669921 |
| cg19565738 | 9.75E-04 | 2 | rs73974109 | 0.847217 | 0.84351 | 0.864385 | 0.820671 |
| cg07484053 | 9.74E-04 | 13 | rs3813739;rs560924610 | 0.625056 | 0.611575 | 0.63469 | 0.691877 |
| cg23175311 | 9.74E-04 | 20 | rs552760606;rs3761245; rs114803159 | 0.764425 | 0.772681 | 0.796506 | 0.815172 |
| cg18712514 | 9.64E-04 | 1 | rs551942917 | 0.684072 | 0.69418 | 0.724907 | 0.644923 |
| cg11657056 | 9.64E-04 | 21 | rs549156428;rs113847674 | 0.779194 | 0.781076 | 0.811419 | 0.770743 |
| cg18476499 | 9.59E-04 | 4 | rs562033476 | 0.645812 | 0.626612 | 0.650044 | 0.692791 |
| cg10492240 | 9.50E-04 | 4 | rs531095480 | 0.688778 | 0.685342 | 0.707846 | 0.7321 |
| cg07840179 | 9.45E-04 | 5 |  | 0.596185 | 0.614524 | 0.635057 | 0.568028 |
| cg03417903 | 9.43E-04 | 20 | rs138341522 | 0.676887 | 0.672213 | 0.694471 | 0.715886 |
| cg18045685 | 9.41E-04 | 7 |  | 0.816643 | 0.80157 | 0.824128 | 0.845516 |
| cg24042578 | 9.38E-04 | 1 | rs139079224 | 0.753356 | 0.751923 | 0.786692 | 0.79782 |
| cg17908749 | 9.35E-04 | 5 | rs561734609;rs187407097 | 0.777658 | 0.794308 | 0.821205 | 0.787575 |
| cg19520512 | 9.33E-04 | 3 | rs141564470 | 0.555395 | 0.568448 | 0.590959 | 0.532965 |
| cg20697427 | 9.30E-04 | 3 | rs200615909 | 0.816712 | 0.80075 | 0.832355 | 0.835444 |
| cg22538328 | 9.27E-04 | 11 | rs7126744 | 0.621703 | 0.613209 | 0.633638 | 0.664591 |
| cg03268942 | 9.27E-04 | 2 | rs185499905;rs532172507; rs547124770 | 0.66251 | 0.64693 | 0.667715 | 0.687167 |
| cg02404349 | 9.26E-04 | 15 | rs568854276;rs529948329 | 0.754915 | 0.766805 | 0.788738 | 0.742954 |
| cg20997632 | 9.13E-04 | 2 | rs13401811 | 0.497333 | 0.506415 | 0.527774 | 0.463493 |
| cg14744841 | 9.11E-04 | 16 | rs534983736;rs553115477; rs574655971 | 0.646355 | 0.628396 | 0.648553 | 0.704316 |
| cg26135322 | 8.95E-04 | 1 | rs554774949 | 0.780456 | 0.774059 | 0.79751 | 0.8163 |
| cg12401398 | 8.95E-04 | 11 | rs539477245;rs181432191 | 0.704459 | 0.708693 | 0.733034 | 0.75549 |
| cg10933035 | 8.90E-04 | 19 | rs543326185 | 0.715877 | 0.713089 | 0.735805 | 0.776557 |
| cg01881193 | 8.87E-04 | 16 | rs370410469 | 0.519933 | 0.50264 | 0.525222 | 0.574712 |
| cg13033086 | 8.84E-04 | 15 | rs543382429 | 0.523511 | 0.509971 | 0.530078 | 0.589719 |
| cg11762511 | 8.79E-04 | 13 | rs140075769;rs548799840; rs191906636 | 0.762506 | 0.771646 | 0.801572 | 0.730241 |
| cg13017471 | 8.69E-04 | 3 | rs574448231;rs151317659 | 0.703555 | 0.710345 | 0.731514 | 0.763014 |
| cg15304012 | 8.67E-04 | 6 |  | 0.676559 | 0.663754 | 0.695489 | 0.727484 |
| cg16618482 | 8.62E-04 | 9 |  | 0.682888 | 0.678248 | 0.70209 | 0.742675 |
| cg00445377 | 8.62E-04 | 5 | rs190837473;rs368415533; rs145060390 | 0.631184 | 0.619983 | 0.644127 | 0.686683 |
| cg09010228 | 8.57E-04 | 9 | rs148819547;rs146940924; rs551295782 | 0.755846 | 0.750832 | 0.771094 | 0.793708 |
| cg04140297 | 8.55E-04 | 8 | rs142132672;rs374942762; rs374203286 | 0.786383 | 0.785415 | 0.816801 | 0.820104 |
| cg09539739 | 8.54E-04 | 16 | rs576185695 | 0.592569 | 0.592083 | 0.612861 | 0.664023 |
| cg11844042 | 8.54E-04 | 4 | rs113735600;rs554043428; rs147765219;rs575575035; rs564251596 | 0.666812 | 0.675956 | 0.703882 | 0.72853 |
| cg26234885 | 8.53E-04 | 7 | rs145093306;rs146851898; rs201598418 | 0.685108 | 0.665317 | 0.694204 | 0.722224 |
| cg07232609 | 8.53E-04 | 15 |  | 0.481057 | 0.49484 | 0.523867 | 0.471655 |
| cg03812546 | 8.52E-04 | 12 | rs577107302 | 0.768579 | 0.767034 | 0.789202 | 0.812237 |
| cg04893852 | 8.49E-04 | 3 | rs184868643;rs573605407 | 0.568985 | 0.560874 | 0.585147 | 0.637185 |
| cg06958535 | 8.47E-04 | 1 |  | 0.631673 | 0.647437 | 0.667929 | 0.59234 |
| cg23668190 | 8.43E-04 | 21 | rs578062624;rs545061393 | 0.682845 | 0.674271 | 0.695082 | 0.715206 |
| cg25193319 | 8.40E-04 | 15 | rs111540567 | 0.766035 | 0.756382 | 0.776699 | 0.799215 |
| cg23828146 | 8.37E-04 | 1 | rs572332337 | 0.682557 | 0.695456 | 0.718227 | 0.681664 |
| cg08552290 | 8.33E-04 | 19 | rs143691008;rs377731268; rs533276482;rs150508720; rs563570117 | 0.716263 | 0.710461 | 0.740452 | 0.767942 |
| cg06880963 | 8.31E-04 | 11 | rs536749054 | 0.6586 | 0.64364 | 0.666225 | 0.696599 |
| cg03258139 | 8.30E-04 | 9 | rs181809442;rs547481626; rs148374748;rs575038609; rs62534881;rs151009526; rs7040790 | 0.441946 | 0.423217 | 0.446833 | 0.500288 |
| cg17068070 | 8.27E-04 | 7 | rs541122411;rs113339151; rs533337092 | 0.686319 | 0.681619 | 0.703241 | 0.721275 |
| cg14129637 | 8.24E-04 | 10 | rs539654849 | 0.771971 | 0.780574 | 0.802151 | 0.761275 |
| cg24141339 | 8.22E-04 | 22 | rs554828900;rs576595577; rs543680128 | 0.661715 | 0.651656 | 0.674229 | 0.716027 |
| cg02314983 | 8.19E-04 | 11 | rs561635109;rs529036498; rs544022704 | 0.737253 | 0.738819 | 0.759252 | 0.778031 |
| cg04436713 | 8.14E-04 | 3 | rs144555878;rs185184230; rs142692871 | 0.754308 | 0.769971 | 0.791866 | 0.74818 |
| cg15397930 | 8.10E-04 | 12 | rs200979388;rs202014462 | 0.36003 | 0.3729 | 0.398254 | 0.444347 |
| cg16200257 | 8.01E-04 | 15 | rs543369815;rs537887131; rs557003679;rs75579948 | 0.674353 | 0.665763 | 0.688795 | 0.716616 |
| cg18753680 | 8.00E-04 | 14 | rs373035751;rs549424993; rs559953277;rs528684107; rs551609082 | 0.609743 | 0.605837 | 0.62635 | 0.657192 |
| cg16556656 | 7.99E-04 | 16 | rs574101099;rs146328689; rs139546058;rs533303064; rs184229055 | 0.785973 | 0.779884 | 0.80007 | 0.823462 |
| cg15512528 | 7.98E-04 | 1 | rs140435603 | 0.748635 | 0.742711 | 0.766116 | 0.711938 |
| cg19043679 | 7.95E-04 | 5 | rs187945927;rs4976688; rs4976689 | 0.587543 | 0.596356 | 0.617263 | 0.637331 |
| cg24681304 | 7.93E-04 | 2 | rs145457687;rs532072352; rs6705008 | 0.596399 | 0.609377 | 0.633072 | 0.573178 |
| cg03994184 | 7.93E-04 | 7 | rs565138509;rs527837739 | 0.813874 | 0.796481 | 0.826262 | 0.846546 |
| cg12875749 | 7.90E-04 | 6 | rs140871995;rs496790; rs186981612;rs558449729 | 0.563864 | 0.575462 | 0.601091 | 0.638766 |
| cg06827339 | 7.82E-04 | 12 | rs75448842 | 0.763083 | 0.774535 | 0.795937 | 0.735455 |
| cg11940690 | 7.81E-04 | 3 | rs149817415;rs13059224; rs113526199;rs749072; rs538598242 | 0.813088 | 0.81603 | 0.836301 | 0.796966 |
| cg06072259 | 7.80E-04 | 9 | rs559471605;rs577778718; rs561180842;rs563244865 | 0.740924 | 0.740646 | 0.775536 | 0.725054 |
| cg09261992 | 7.79E-04 | 1 | rs540309403;rs140791471; rs532100793;rs545506093; rs565528694 | 0.762259 | 0.768529 | 0.789064 | 0.80465 |
| cg20756954 | 7.78E-04 | 16 | rs150374710 | 0.745086 | 0.759783 | 0.785536 | 0.737003 |
| cg21797863 | 7.76E-04 | 19 | rs199910856 | 0.615833 | 0.608322 | 0.62923 | 0.663751 |
| cg24680758 | 7.72E-04 | 6 | rs566980289 | 0.716975 | 0.724084 | 0.748526 | 0.706734 |
| cg23472153 | 7.69E-04 | 17 |  | 0.730378 | 0.718051 | 0.745215 | 0.765094 |
| cg16232444 | 7.68E-04 | 6 | rs117261919;rs375252992; rs139394111 | 0.531439 | 0.535635 | 0.567056 | 0.606562 |
| cg08975357 | 7.66E-04 | 1 | rs554632349;rs373998131 | 0.755531 | 0.743077 | 0.764636 | 0.791806 |
| cg13582399 | 7.66E-04 | 19 | rs182066327;rs527518202 | 0.729885 | 0.720894 | 0.741369 | 0.758425 |
| cg10796068 | 7.61E-04 | 19 | rs149032368;rs11880504 | 0.789877 | 0.80587 | 0.826065 | 0.787001 |
| cg02923972 | 7.59E-04 | 1 |  | 0.649335 | 0.63443 | 0.659448 | 0.693482 |
| cg11184985 | 7.59E-04 | 2 | rs75688901 | 0.632042 | 0.625331 | 0.646986 | 0.725237 |
| cg20161145 | 7.55E-04 | 10 | rs555874486;rs572083934 | 0.731121 | 0.729724 | 0.750307 | 0.703355 |
| cg26628069 | 7.52E-04 | 17 | rs149998766 | 0.594288 | 0.58667 | 0.634059 | 0.664188 |
| cg06426818 | 7.46E-04 | 5 | rs189211511;rs547772380; rs141033192;rs150257913; rs191708028;rs543677503; rs571763020 | 0.785756 | 0.795571 | 0.820016 | 0.764858 |
| cg07593415 | 7.40E-04 | 19 | rs114695632;rs564612569 | 0.77129 | 0.758089 | 0.778882 | 0.805064 |
| cg11485049 | 7.39E-04 | 15 | rs561870165 | 0.663057 | 0.65295 | 0.673155 | 0.693426 |
| cg25517015 | 7.34E-04 | 19 | rs534591419;rs111382030; rs554633287 | 0.615826 | 0.613223 | 0.642004 | 0.68154 |
| cg20103352 | 7.33E-04 | 15 | rs535421086;rs554899573 | 0.606314 | 0.586329 | 0.608381 | 0.672214 |
| cg10496420 | 7.28E-04 | 10 |  | 0.786621 | 0.783684 | 0.810031 | 0.824547 |
| cg09184464 | 7.25E-04 | 8 | rs77412240;rs555086205; rs9650314;rs534346601 | 0.449155 | 0.45465 | 0.478633 | 0.45873 |
| cg09507782 | 7.23E-04 | 7 | rs531536944;rs575539380; rs549784606;rs568315141 | 0.67104 | 0.665809 | 0.68787 | 0.7123 |
| cg08352530 | 7.18E-04 | 7 | rs147913028;rs112408875; rs73676731;rs537219830 | 0.657572 | 0.660378 | 0.688039 | 0.736974 |
| cg08351489 | 7.16E-04 | 11 | rs150797068;rs1792364 | 0.359435 | 0.371346 | 0.39631 | 0.358823 |
| cg16531578 | 7.14E-04 | 6 | rs181360853 | 0.294157 | 0.277312 | 0.298892 | 0.404923 |
| cg03538383 | 7.12E-04 | 12 | rs200730218 | 0.684103 | 0.684524 | 0.713204 | 0.746759 |
| cg07299144 | 7.10E-04 | 16 | rs573522518;rs182842578 | 0.813615 | 0.808266 | 0.829321 | 0.843482 |
| cg16968894 | 7.05E-04 | 2 | rs544529609 | 0.68261 | 0.688016 | 0.708131 | 0.732588 |
| cg06531025 | 6.95E-04 | 19 | rs528788506 | 0.568943 | 0.564788 | 0.585646 | 0.54581 |
| cg00365945 | 6.95E-04 | 20 | rs574106118 | 0.706093 | 0.69516 | 0.719149 | 0.737173 |
| cg19885037 | 6.94E-04 | 3 | rs185645668;rs79379778 | 0.6848 | 0.668077 | 0.688155 | 0.719596 |
| cg07839071 | 6.94E-04 | 6 | rs138135097 | 0.535664 | 0.542866 | 0.571012 | 0.626326 |
| cg13762904 | 6.92E-04 | 6 | rs528156226;rs145223414; rs190885964 | 0.796448 | 0.806962 | 0.827225 | 0.780616 |
| cg17498424 | 6.85E-04 | 22 | rs59099247;rs78047908 | 0.737031 | 0.731897 | 0.753565 | 0.783514 |
| cg01105321 | 6.83E-04 | 15 | rs573718781;rs144852618 | 0.819801 | 0.802141 | 0.82329 | 0.842438 |
| cg14355654 | 6.76E-04 | 8 | rs138814750 | 0.622113 | 0.618469 | 0.640029 | 0.674702 |
| cg17907003 | 6.76E-04 | 1 |  | 0.57886 | 0.567865 | 0.605214 | 0.649905 |
| cg14261271 | 6.73E-04 | 2 | rs185187470;rs149999970; rs34974542 | 0.776271 | 0.770984 | 0.791377 | 0.754456 |
| cg19270512 | 6.66E-04 | 5 | rs549412894 | 0.536789 | 0.544085 | 0.567461 | 0.612402 |
| cg22684425 | 6.64E-04 | 21 | rs573366125;rs181149547 | 0.603755 | 0.608386 | 0.629134 | 0.675696 |
| cg23122901 | 6.58E-04 | 22 | rs562542338 | 0.50861 | 0.519795 | 0.544186 | 0.499217 |
| cg02673849 | 6.58E-04 | 12 | rs566696564;rs533840550; rs73288733;rs529255766 | 0.670912 | 0.675855 | 0.698406 | 0.640039 |
| cg05420685 | 6.57E-04 | 16 | rs34558384;rs57506432 | 0.494817 | 0.4831 | 0.504063 | 0.563132 |
| cg20064558 | 6.46E-04 | 10 | rs542041775;rs188061697 | 0.757232 | 0.764575 | 0.789975 | 0.744655 |
| cg12819393 | 6.40E-04 | 9 | rs578049213 | 0.648099 | 0.644223 | 0.669766 | 0.685395 |
| cg04728402 | 6.38E-04 | 11 | rs545261132;rs565257978 | 0.501659 | 0.482901 | 0.50732 | 0.56762 |
| cg09756115 | 6.36E-04 | 1 | rs202045502;rs199998341; rs142832714;rs558379044; rs199869504;rs139717535; rs150903674;rs150064100; rs563620164 | 0.746999 | 0.734389 | 0.757699 | 0.787772 |
| cg16336248 | 6.36E-04 | 8 | rs531220656;rs549743507 | 0.385479 | 0.3813 | 0.402539 | 0.363661 |
| cg23439460 | 6.34E-04 | 10 | rs61871512 | 0.251466 | 0.240901 | 0.261837 | 0.251558 |
| cg18306932 | 6.30E-04 | 14 | rs77889730;rs140364284 | 0.579266 | 0.565116 | 0.587743 | 0.638736 |
| cg25058463 | 6.30E-04 | 1 | rs566181939 | 0.683927 | 0.669071 | 0.702905 | 0.731555 |
| cg05339114 | 6.28E-04 | 10 |  | 0.354975 | 0.356849 | 0.380769 | 0.33148 |
| cg00391821 | 6.21E-04 | 1 |  | 0.603092 | 0.593887 | 0.614402 | 0.652989 |
| cg03603485 | 6.21E-04 | 1 | rs143357548 | 0.592383 | 0.604748 | 0.629055 | 0.568593 |
| cg04876597 | 6.18E-04 | 16 | rs547663244;rs566081962; rs113374957;rs565841321 | 0.791356 | 0.78634 | 0.810803 | 0.818038 |
| cg14244842 | 6.17E-04 | 4 | rs530638303;rs556806088 | 0.467549 | 0.478091 | 0.502418 | 0.458543 |
| cg03958078 | 6.15E-04 | 4 | rs566385975 | 0.231078 | 0.233866 | 0.255203 | 0.244651 |
| cg17461700 | 6.13E-04 | X |  | 0.601386 | 0.586087 | 0.607556 | 0.648828 |
| cg08911692 | 6.12E-04 | 7 | rs182949561 | 0.714474 | 0.707602 | 0.737505 | 0.77296 |
| cg23403797 | 6.05E-04 | 8 |  | 0.69574 | 0.712276 | 0.741524 | 0.643377 |
| cg14986215 | 5.98E-04 | 10 | rs573835324 | 0.728958 | 0.717008 | 0.742307 | 0.774619 |
| cg11648638 | 5.93E-04 | 19 | rs114139254;rs571562266 | 0.785437 | 0.783471 | 0.810435 | 0.834748 |
| cg03169691 | 5.87E-04 | 14 | rs556793793 | 0.839921 | 0.839618 | 0.860305 | 0.83974 |
| cg22197416 | 5.87E-04 | 17 |  | 0.632379 | 0.624986 | 0.645308 | 0.689163 |
| cg18344193 | 5.83E-04 | 9 | rs180884650;rs150065615 | 0.563722 | 0.577876 | 0.601451 | 0.558058 |
| cg16832091 | 5.81E-04 | 5 | rs574153756;rs541525283 | 0.711441 | 0.712646 | 0.733039 | 0.750095 |
| cg02735988 | 5.79E-04 | 20 | rs545202762;rs535811837; rs527722476;rs181227166 | 0.66947 | 0.685379 | 0.706069 | 0.656435 |
| cg02657797 | 5.77E-04 | 12 | rs143230696;rs558455305 | 0.827379 | 0.838153 | 0.861848 | 0.816597 |
| cg25683811 | 5.76E-04 | 6 | rs557093228;rs575869138 | 0.497836 | 0.499606 | 0.523024 | 0.473393 |
| cg07601456 | 5.73E-04 | 21 |  | 0.65752 | 0.655121 | 0.677469 | 0.727725 |
| cg09618291 | 5.71E-04 | 2 | rs571814766;rs534329935 | 0.361027 | 0.357931 | 0.384296 | 0.366863 |
| cg08425166 | 5.66E-04 | 11 | rs550296716;rs556187717 | 0.733691 | 0.731153 | 0.7547 | 0.773871 |
| cg17002772 | 5.66E-04 | 11 | rs185966000;rs540880065; rs560625251;rs577134798 | 0.755729 | 0.764105 | 0.78678 | 0.792436 |
| cg02479755 | 5.64E-04 | 16 | rs187606458;rs551685565 | 0.690481 | 0.685707 | 0.720232 | 0.755637 |
| cg04585778 | 5.64E-04 | 17 |  | 0.197689 | 0.198123 | 0.2246 | 0.198363 |
| cg07682434 | 5.57E-04 | 2 | rs181951918;rs553151511; rs566631715;rs895437 | 0.794697 | 0.809872 | 0.831382 | 0.795171 |
| cg18736474 | 5.53E-04 | 1 | rs181552909 | 0.719662 | 0.715739 | 0.739516 | 0.768506 |
| cg02053920 | 5.52E-04 | 6 | rs531695585;rs188146206 | 0.774115 | 0.762136 | 0.784614 | 0.80629 |
| cg15635786 | 5.49E-04 | 8 | rs547798591 | 0.486956 | 0.473147 | 0.493722 | 0.550909 |
| cg07455936 | 5.47E-04 | 17 | rs140577952;rs374361883; rs199784329 | 0.818438 | 0.829834 | 0.85053 | 0.851618 |
| cg18558028 | 5.44E-04 | 7 | rs182923525;rs144041332 | 0.640104 | 0.631148 | 0.651341 | 0.703699 |
| cg16665029 | 5.36E-04 | 3 | rs571608216 | 0.572717 | 0.560221 | 0.58423 | 0.631768 |
| cg21423741 | 5.35E-04 | 3 | rs573670360;rs544186374 | 0.707969 | 0.707174 | 0.730775 | 0.746387 |
| cg19056927 | 5.34E-04 | 1 | rs151308916;rs536435837; rs140559734;rs569845928 | 0.582194 | 0.567216 | 0.588051 | 0.641297 |
| cg09580922 | 5.32E-04 | 17 | rs147576707;rs371002023; rs558463283;rs187205530 | 0.694059 | 0.684806 | 0.707049 | 0.750538 |
| cg03411938 | 5.27E-04 | 11 | rs185255596 | 0.506418 | 0.491438 | 0.523162 | 0.554294 |
| cg22801747 | 5.27E-04 | 11 | rs552955310;rs577533572 | 0.727484 | 0.711052 | 0.739999 | 0.783099 |
| cg16972043 | 5.25E-04 | 16 | rs561272682 | 0.497567 | 0.487909 | 0.513478 | 0.467654 |
| cg15215578 | 5.24E-04 | 2 |  | 0.734998 | 0.734406 | 0.763769 | 0.725899 |
| cg13298528 | 5.23E-04 | 11 |  | 0.60829 | 0.624995 | 0.6471 | 0.588661 |
| cg01362225 | 5.21E-04 | 14 |  | 0.779988 | 0.764653 | 0.787017 | 0.813793 |
| cg05979170 | 5.16E-04 | 3 | rs531819543 | 0.819667 | 0.827294 | 0.84802 | 0.805439 |
| cg25273875 | 5.14E-04 | 14 | rs192538288 | 0.708772 | 0.712034 | 0.738413 | 0.657007 |
| cg04524040 | 5.13E-04 | 19 | rs350880;rs538469153; rs555160554 | 0.451867 | 0.462677 | 0.483664 | 0.44 |
| cg16374225 | 5.10E-04 | 7 | rs76113232;rs148693393 | 0.787742 | 0.798189 | 0.818718 | 0.76999 |
| cg05352027 | 5.02E-04 | 19 | rs557744807 | 0.724719 | 0.716889 | 0.742855 | 0.766175 |
| cg12321355 | 5.02E-04 | 3 | rs147528852;rs549674351 | 0.778143 | 0.779936 | 0.800979 | 0.828233 |
| cg14032964 | 4.99E-04 | 9 | rs577479855;rs543719910; rs563088861;rs528888579; rs142554949 | 0.660524 | 0.652877 | 0.675736 | 0.706044 |
| cg24736561 | 4.98E-04 | 20 | rs148160098;rs527381389 | 0.485747 | 0.472568 | 0.494997 | 0.568753 |
| cg24380489 | 4.97E-04 | 17 | rs558484190;rs576749816 | 0.624963 | 0.622423 | 0.64382 | 0.700192 |
| cg01547466 | 4.97E-04 | 18 | rs185272335 | 0.669216 | 0.665643 | 0.697133 | 0.713837 |
| cg17564205 | 4.97E-04 | 12 |  | 0.796776 | 0.785315 | 0.808966 | 0.82138 |
| cg02694800 | 4.96E-04 | 6 | rs144627048;rs556693854; rs191764332 | 0.58318 | 0.602923 | 0.623591 | 0.556144 |
| cg17098093 | 4.94E-04 | 1 | rs184203943;rs71666235 | 0.66346 | 0.655941 | 0.676208 | 0.713015 |
| cg11628858 | 4.93E-04 | 16 | rs189023150;rs180910132 | 0.789869 | 0.802802 | 0.822847 | 0.769607 |
| cg06527396 | 4.78E-04 | 3 |  | 0.629553 | 0.618481 | 0.643646 | 0.687984 |
| cg05272476 | 4.74E-04 | 17 | rs574246405 | 0.759213 | 0.74812 | 0.770225 | 0.808348 |
| cg08130814 | 4.73E-04 | 8 | rs140311752 | 0.832603 | 0.844977 | 0.86822 | 0.868932 |
| cg11425201 | 4.72E-04 | 19 |  | 0.800709 | 0.791469 | 0.813666 | 0.827409 |
| cg20567148 | 4.72E-04 | 7 | rs79126847;rs148427197; rs114761150 | 0.685655 | 0.668423 | 0.696544 | 0.727806 |
| cg08083041 | 4.71E-04 | 15 | rs185983934;rs74027902 | 0.571853 | 0.568714 | 0.588879 | 0.63642 |
| cg23140076 | 4.64E-04 | 10 | rs571259752;rs532308991 | 0.729045 | 0.717782 | 0.74346 | 0.776316 |
| cg01449198 | 4.63E-04 | X | rs143354573 | 0.717422 | 0.730728 | 0.757941 | 0.697457 |
| cg24528574 | 4.62E-04 | 1 | rs147411586;rs72896094 | 0.667468 | 0.660156 | 0.684372 | 0.720593 |
| cg08949143 | 4.61E-04 | 13 |  | 0.384474 | 0.394736 | 0.419381 | 0.368566 |
| cg22051370 | 4.55E-04 | 7 | rs577885347;rs113712408; rs564067316 | 0.609541 | 0.596768 | 0.618135 | 0.662865 |
| cg22626683 | 4.54E-04 | 1 | rs57440247;rs111276830; rs541641478;rs190428488 | 0.397431 | 0.394305 | 0.41713 | 0.499011 |
| cg14784833 | 4.41E-04 | 1 | rs376222551;rs59569634; rs77786147 | 0.759582 | 0.766447 | 0.787898 | 0.723788 |
| cg16546834 | 4.40E-04 | 20 | rs556513295;rs556535522; rs6115656;rs148734462; rs572174392 | 0.851661 | 0.841398 | 0.868663 | 0.882711 |
| cg10973967 | 4.40E-04 | 15 | rs148161696;rs557558067; rs141056670 | 0.601969 | 0.593285 | 0.61757 | 0.65956 |
| cg23395177 | 4.40E-04 | 14 | rs28673127;rs544130510 | 0.628559 | 0.621027 | 0.6417 | 0.675149 |
| cg09075114 | 4.39E-04 | 4 | rs566423785;rs552904061; rs528677642;rs149293393; rs10939039;rs539494200 | 0.6448 | 0.642416 | 0.663328 | 0.712979 |
| cg02374486 | 4.37E-04 | 10 | rs542536578 | 0.616453 | 0.633611 | 0.658574 | 0.579654 |
| cg18071806 | 4.32E-04 | 12 | rs533235888 | 0.625129 | 0.609851 | 0.631115 | 0.670337 |
| cg00089214 | 4.31E-04 | 12 | rs533484056 | 0.7575 | 0.758265 | 0.784314 | 0.798472 |
| cg11167508 | 4.31E-04 | 6 | rs9355687 | 0.667074 | 0.662205 | 0.682729 | 0.737041 |
| cg25050723 | 4.28E-04 | 11 |  | 0.702381 | 0.704888 | 0.728499 | 0.751977 |
| cg05339403 | 4.25E-04 | 18 |  | 0.822283 | 0.830471 | 0.85098 | 0.819431 |
| cg06446550 | 4.23E-04 | 12 | rs556320705;rs79864004 | 0.665164 | 0.664883 | 0.687412 | 0.717215 |
| cg10849662 | 4.22E-04 | 17 | rs534613802;rs553189914 | 0.780986 | 0.771882 | 0.796756 | 0.815781 |
| cg08178031 | 4.22E-04 | 3 | rs16861460;rs79953567; rs147721722;rs6764279; rs534339849;rs114012396 | 0.771881 | 0.758812 | 0.781291 | 0.810499 |
| cg14381944 | 4.18E-04 | 5 | rs554209839;rs187953858 | 0.586327 | 0.568508 | 0.594263 | 0.644954 |
| cg06026786 | 4.18E-04 | 11 | rs559713382;rs2239695; rs548307336;rs568215063 | 0.612885 | 0.627141 | 0.654809 | 0.578989 |
| cg13784559 | 4.18E-04 | 2 | rs201707177;rs139293378; rs546582094;rs568012132; rs201221506 | 0.595957 | 0.605946 | 0.627326 | 0.560536 |
| cg25513379 | 4.14E-04 | 5 | rs567135744;rs536166018 | 0.679944 | 0.667516 | 0.68935 | 0.736087 |
| cg03625953 | 4.13E-04 | 17 | rs529379422 | 0.703913 | 0.694888 | 0.722122 | 0.757334 |
| cg09689650 | 4.13E-04 | 15 |  | 0.838466 | 0.85363 | 0.875342 | 0.838102 |
| cg07038004 | 4.10E-04 | 3 | rs185740013;rs138533007 | 0.658918 | 0.66355 | 0.685556 | 0.707832 |
| cg23756763 | 4.03E-04 | 2 | rs551918195;rs143570969 | 0.819218 | 0.833932 | 0.858996 | 0.810883 |
| cg01770362 | 3.98E-04 | 8 |  | 0.528952 | 0.531702 | 0.552079 | 0.581549 |
| cg05990867 | 3.98E-04 | 21 | rs551478255;rs79760373; rs114683625 | 0.821125 | 0.811076 | 0.831889 | 0.85295 |
| cg23234774 | 3.98E-04 | 19 | rs534631522;rs557443629 | 0.691047 | 0.684553 | 0.710227 | 0.720947 |
| cg24529532 | 3.93E-04 | 3 | rs183822097 | 0.519441 | 0.507005 | 0.533386 | 0.578097 |
| cg15222485 | 3.93E-04 | 2 | rs186577020;rs560891591 | 0.738989 | 0.753001 | 0.774449 | 0.721176 |
| cg22366799 | 3.90E-04 | 11 | rs181452630;rs559233776; rs577442699 | 0.811729 | 0.826257 | 0.852633 | 0.823056 |
| cg07139190 | 3.86E-04 | 6 | rs557407314;rs187606698; rs543618862;rs544996408 | 0.734881 | 0.73252 | 0.753928 | 0.78996 |
| cg07906003 | 3.85E-04 | 3 |  | 0.605142 | 0.602767 | 0.651523 | 0.591359 |
| cg07633839 | 3.81E-04 | 2 | rs141987649;rs544164250 | 0.663233 | 0.655769 | 0.677546 | 0.695932 |
| cg07380942 | 3.79E-04 | 16 | rs564763825;rs188509637; rs544189979 | 0.716588 | 0.707631 | 0.728379 | 0.76646 |
| cg26693033 | 3.76E-04 | 12 | rs142437646;rs145961445 | 0.706384 | 0.722305 | 0.742693 | 0.688585 |
| cg09955928 | 3.75E-04 | 2 | rs148141594;rs141924324; rs369973599;rs540406547; rs150243535;rs115684336 | 0.482922 | 0.472329 | 0.496861 | 0.561492 |
| cg05457104 | 3.73E-04 | 13 | rs577649735 | 0.652068 | 0.648288 | 0.670404 | 0.711247 |
| cg13183539 | 3.69E-04 | 19 | rs546391702;rs139976530; rs145487632;rs544188593 | 0.812594 | 0.808531 | 0.832734 | 0.848462 |
| cg14747209 | 3.69E-04 | 16 | rs191520727;rs137981576; rs563610165;rs182538289 | 0.811863 | 0.79972 | 0.827578 | 0.838115 |
| cg18899797 | 3.62E-04 | 5 | rs74820434;rs118093183 | 0.69773 | 0.705518 | 0.731792 | 0.684898 |
| cg12842646 | 3.56E-04 | 13 | rs41275124;rs41275126; rs140474346;rs77771278 | 0.654573 | 0.648658 | 0.68426 | 0.697463 |
| cg02396325 | 3.54E-04 | 7 | rs549212264 | 0.63821 | 0.622001 | 0.646143 | 0.687216 |
| cg21892407 | 3.52E-04 | 2 | rs191082685;rs140975021; rs183169551 | 0.270928 | 0.278873 | 0.300855 | 0.261863 |
| cg24798995 | 3.50E-04 | 4 | rs143392200 | 0.74959 | 0.740722 | 0.770253 | 0.781108 |
| cg07693617 | 3.49E-04 | 1 | rs566907204 | 0.650678 | 0.650476 | 0.673408 | 0.703894 |
| cg24260500 | 3.48E-04 | 12 | rs187648065;rs142609275 | 0.651616 | 0.650931 | 0.683443 | 0.717358 |
| cg18645642 | 3.47E-04 | 1 | rs573530635 | 0.647132 | 0.630379 | 0.65302 | 0.701882 |
| cg18682028 | 3.46E-04 | 3 | rs555399617 | 0.722523 | 0.742101 | 0.767744 | 0.716112 |
| cg25020820 | 3.45E-04 | 6 | rs537260797 | 0.686442 | 0.689911 | 0.710651 | 0.748094 |
| cg22089942 | 3.42E-04 | 4 | rs533151554;rs551318975; rs566392302 | 0.64809 | 0.647073 | 0.667849 | 0.709164 |
| cg00585776 | 3.41E-04 | 16 | rs140011781;rs528282793 | 0.744695 | 0.740041 | 0.760159 | 0.798596 |
| cg10119383 | 3.34E-04 | 6 | rs189834749 | 0.640919 | 0.649373 | 0.670746 | 0.706857 |
| cg24172324 | 3.33E-04 | 2 | rs142501771 | 0.355893 | 0.364323 | 0.387333 | 0.344956 |
| cg10629085 | 3.31E-04 | X |  | 0.6544 | 0.638964 | 0.661449 | 0.727388 |
| cg06553089 | 3.29E-04 | 1 |  | 0.753979 | 0.743579 | 0.776687 | 0.730544 |
| cg18457394 | 3.27E-04 | 2 | rs79183148 | 0.656571 | 0.664951 | 0.685281 | 0.634138 |
| cg15548768 | 3.26E-04 | 1 | rs550602949;rs567235124; rs536374485 | 0.767052 | 0.774972 | 0.796542 | 0.80552 |
| cg12331108 | 3.26E-04 | 16 | rs554415324;rs565360207 | 0.697153 | 0.679951 | 0.706157 | 0.737022 |
| cg19523620 | 3.24E-04 | 10 | rs79734561 | 0.820784 | 0.820125 | 0.854959 | 0.858755 |
| cg07386086 | 3.22E-04 | 8 | rs183986754;rs8178070; rs575070006 | 0.821298 | 0.836089 | 0.861189 | 0.813714 |
| cg23200278 | 3.21E-04 | 6 | rs183855401 | 0.558715 | 0.549645 | 0.587776 | 0.627443 |
| cg03474687 | 3.21E-04 | 14 |  | 0.780668 | 0.774482 | 0.797006 | 0.81362 |
| cg13886338 | 3.20E-04 | 8 | rs538174700;rs537114861 | 0.720315 | 0.713566 | 0.734671 | 0.764208 |
| cg09176594 | 3.20E-04 | 3 | rs2606220;rs188414958 | 0.737966 | 0.727572 | 0.755372 | 0.778095 |
| cg22862205 | 3.18E-04 | 6 | rs557845022 | 0.283969 | 0.28614 | 0.309742 | 0.272365 |
| cg04108240 | 3.16E-04 | 7 | rs562663811;rs148754166 | 0.687155 | 0.681106 | 0.703384 | 0.741087 |
| cg07866798 | 3.16E-04 | 13 | rs199749872 | 0.511481 | 0.509415 | 0.53621 | 0.604727 |
| cg16220435 | 3.06E-04 | 12 |  | 0.651386 | 0.643747 | 0.668323 | 0.685867 |
| cg23449570 | 3.05E-04 | 6 | rs539959457 | 0.72848 | 0.746106 | 0.766608 | 0.699604 |
| cg25338515 | 3.03E-04 | 16 | rs115444624;rs148362793 | 0.63311 | 0.625762 | 0.647511 | 0.69218 |
| cg01534390 | 2.96E-04 | 14 | rs113678567 | 0.76024 | 0.771799 | 0.794323 | 0.747637 |
| cg03414898 | 2.95E-04 | 10 | rs569283959 | 0.642998 | 0.625204 | 0.650972 | 0.70502 |
| cg13759513 | 2.95E-04 | 20 |  | 0.756054 | 0.755679 | 0.780204 | 0.810712 |
| cg11260269 | 2.88E-04 | 12 | rs561559236;rs530104592 | 0.831497 | 0.837046 | 0.86213 | 0.819439 |
| cg02943710 | 2.88E-04 | 2 | rs541376756;rs559657572 | 0.75711 | 0.76364 | 0.788885 | 0.731211 |
| cg12256921 | 2.82E-04 | 7 | rs115359614;rs572954463; rs192876493 | 0.756449 | 0.77367 | 0.800524 | 0.743408 |
| cg17545652 | 2.82E-04 | 10 | rs146440898;rs562604501; rs533035975;rs545069665 | 0.727724 | 0.71217 | 0.735521 | 0.755826 |
| cg08403513 | 2.81E-04 | 5 | rs559223326 | 0.628148 | 0.642198 | 0.679246 | 0.607893 |
| cg14690467 | 2.78E-04 | 7 | rs76040180;rs149937983; rs186381076 | 0.547443 | 0.532973 | 0.554551 | 0.606641 |
| cg21779510 | 2.76E-04 | 1 | rs74090732;rs72686182; rs182691177;rs187523206; rs568871026 | 0.524447 | 0.509684 | 0.530621 | 0.589977 |
| cg03278407 | 2.73E-04 | 11 | rs564893985;rs532253961; rs191450251;rs182237942; rs529764196;rs186474373; rs570374805 | 0.634762 | 0.624054 | 0.656381 | 0.681465 |
| cg15429134 | 2.73E-04 | 12 | rs3803098;rs150147833; rs190769009;rs546519254 | 0.324818 | 0.333767 | 0.357759 | 0.32929 |
| cg05098381 | 2.71E-04 | 3 |  | 0.679813 | 0.669997 | 0.692894 | 0.734532 |
| cg15809077 | 2.68E-04 | 2 | rs111952968 | 0.820809 | 0.808043 | 0.830195 | 0.868809 |
| cg09379044 | 2.67E-04 | 4 | rs557531340;rs575689637 | 0.714784 | 0.716875 | 0.74173 | 0.786205 |
| cg14538218 | 2.67E-04 | 4 | rs139004723;rs557966984 | 0.654363 | 0.635606 | 0.664561 | 0.707401 |
| cg11521721 | 2.66E-04 | 3 | rs143987107;rs147305927; rs372781583;rs34010809 | 0.746421 | 0.734369 | 0.761701 | 0.785708 |
| cg13497315 | 2.59E-04 | 15 | rs552005257;rs571928429; rs5812122 | 0.795408 | 0.805049 | 0.828247 | 0.7849 |
| cg26834212 | 2.58E-04 | 3 | rs4522807 | 0.628364 | 0.624176 | 0.64892 | 0.670151 |
| cg12170419 | 2.58E-04 | 20 |  | 0.555209 | 0.558739 | 0.579673 | 0.640639 |
| cg07391417 | 2.57E-04 | 2 | rs188578849;rs6726145; rs529449849;rs191465085; rs182450829 | 0.453236 | 0.46791 | 0.491286 | 0.450783 |
| cg15110917 | 2.56E-04 | 7 |  | 0.805171 | 0.816377 | 0.836495 | 0.781502 |
| cg26118326 | 2.56E-04 | 1 |  | 0.7107 | 0.730021 | 0.751041 | 0.658046 |
| cg16164268 | 2.54E-04 | 4 |  | 0.580605 | 0.600576 | 0.633716 | 0.557996 |
| cg18105735 | 2.54E-04 | 17 | rs566465559;rs187668100; rs555903665 | 0.717515 | 0.708288 | 0.730431 | 0.768812 |
| cg24369233 | 2.51E-04 | 2 | rs11884053;rs141530117 | 0.716173 | 0.727747 | 0.752159 | 0.695383 |
| cg10080401 | 2.50E-04 | 22 | rs144633633;rs181868377; rs539922061;rs5999822 | 0.710003 | 0.705589 | 0.726946 | 0.758319 |
| cg22451067 | 2.47E-04 | 10 | rs547127644 | 0.403818 | 0.385548 | 0.417865 | 0.495622 |
| cg14497145 | 2.45E-04 | 10 | rs527529547;rs578131602 | 0.661678 | 0.657252 | 0.67839 | 0.724128 |
| cg23211059 | 2.45E-04 | 3 | rs534028590;rs552380625; rs570981604;rs538309948 | 0.713185 | 0.716451 | 0.742425 | 0.668543 |
| cg20402477 | 2.40E-04 | 19 |  | 0.221847 | 0.236312 | 0.258527 | 0.221344 |
| cg16692494 | 2.37E-04 | 7 | rs116043672;rs538013160 | 0.713063 | 0.71135 | 0.747281 | 0.764626 |
| cg07378255 | 2.33E-04 | 5 | rs367577189;rs199575086; rs138471015;rs201922521; rs200611264;rs570670105 | 0.788219 | 0.784063 | 0.806623 | 0.750634 |
| cg05758692 | 2.31E-04 | 6 | rs187693761 | 0.678602 | 0.684354 | 0.707094 | 0.657172 |
| cg23340957 | 2.25E-04 | 2 |  | 0.6265 | 0.63791 | 0.657923 | 0.593019 |
| cg05618183 | 2.24E-04 | 16 | rs77688126;rs554303770 | 0.32409 | 0.322115 | 0.345451 | 0.303042 |
| cg00085620 | 2.24E-04 | 5 |  | 0.677628 | 0.665866 | 0.690491 | 0.722967 |
| cg15843975 | 2.22E-04 | 11 | rs145958332;rs539016221; rs139867471;rs550182134; rs143163876;rs554821100 | 0.526093 | 0.540399 | 0.574299 | 0.508824 |
| cg15053740 | 2.19E-04 | 16 | rs150526221;rs114164670; rs12930458 | 0.3414 | 0.34787 | 0.368955 | 0.343671 |
| cg26501107 | 2.18E-04 | 11 | rs560899071 | 0.608491 | 0.606619 | 0.630899 | 0.69211 |
| cg11610004 | 2.15E-04 | 2 | rs533640100;rs553192688; rs573110910 | 0.529841 | 0.520813 | 0.547906 | 0.615871 |
| cg03930187 | 2.14E-04 | 18 | rs530213393;rs74982081; rs147338817;rs141024579 | 0.741456 | 0.748953 | 0.771145 | 0.730802 |
| cg16168570 | 2.08E-04 | 3 | rs144530075;rs75269918; rs141774220;rs184286649; rs371887204 | 0.70263 | 0.685083 | 0.706327 | 0.748625 |
| cg08078218 | 2.08E-04 | 15 | rs144400630;rs201149010; rs148786916;rs187392024 | 0.82054 | 0.823074 | 0.853938 | 0.801008 |
| cg23089177 | 2.07E-04 | 7 | rs577676158;rs544569775; rs562966133 | 0.467 | 0.461726 | 0.486785 | 0.44955 |
| cg04616571 | 2.07E-04 | 10 | rs41302703;rs7070816 | 0.605277 | 0.592131 | 0.626509 | 0.667722 |
| cg15490785 | 2.05E-04 | 3 | rs147165546 | 0.736623 | 0.726414 | 0.748475 | 0.763053 |
| cg08036148 | 2.04E-04 | 17 | rs142959828 | 0.76696 | 0.772717 | 0.792994 | 0.719318 |
| cg15544235 | 2.03E-04 | 4 | rs554242249;rs146320452; rs546564411;rs563028535; rs531988986;rs147878273 | 0.616661 | 0.625511 | 0.647302 | 0.695578 |
| cg02976574 | 2.02E-04 | 3 | rs539042799;rs141843418; rs146058540 | 0.771795 | 0.762918 | 0.787838 | 0.830177 |
| cg04546122 | 2.00E-04 | 6 | rs527856562 | 0.523322 | 0.511328 | 0.539615 | 0.582946 |
| cg16600142 | 1.99E-04 | 20 | rs182955545;rs41281230 | 0.565363 | 0.550523 | 0.573204 | 0.63747 |
| cg07893986 | 1.92E-04 | 2 |  | 0.632315 | 0.627566 | 0.654161 | 0.693096 |
| cg13657553 | 1.91E-04 | 18 |  | 0.802616 | 0.797749 | 0.817818 | 0.837811 |
| cg11046602 | 1.91E-04 | 17 | rs76309849;rs118012013 | 0.554827 | 0.537803 | 0.566334 | 0.618019 |
| cg17249227 | 1.90E-04 | 5 | rs537271393;rs141631309; rs164077 | 0.578023 | 0.566363 | 0.59005 | 0.651956 |
| cg24905248 | 1.89E-04 | 2 | rs554191470;rs4586678 | 0.712824 | 0.71153 | 0.735045 | 0.753899 |
| cg20997222 | 1.85E-04 | 4 | rs553092161 | 0.648072 | 0.62953 | 0.657539 | 0.708115 |
| cg06270206 | 1.84E-04 | 16 | rs534079651;rs192004280 | 0.715981 | 0.704079 | 0.731811 | 0.767946 |
| cg05092891 | 1.82E-04 | 21 | rs60372094;rs78460965; rs181634409 | 0.605715 | 0.599527 | 0.624789 | 0.689449 |
| cg12471814 | 1.82E-04 | 1 | rs548898395 | 0.670411 | 0.685875 | 0.712369 | 0.631961 |
| cg22548220 | 1.79E-04 | 6 | rs549116643;rs545121695; rs564628828;rs16897515 | 0.606637 | 0.597358 | 0.621065 | 0.661738 |
| cg18436024 | 1.78E-04 | 4 | rs11930446;rs5856012; rs370203020;rs144369179; rs185073242;rs533373173 | 0.701135 | 0.692197 | 0.71379 | NA |
| cg14399284 | 1.75E-04 | 3 | rs57794552;rs186515101; rs138680524 | 0.820889 | 0.809822 | 0.841207 | 0.815192 |
| cg07850488 | 1.75E-04 | 20 | rs568391561;rs535820243 | 0.682402 | 0.676104 | 0.700889 | 0.746619 |
| cg07105409 | 1.75E-04 | 18 | rs570757439;rs566331072; rs144905682;rs574403068 | 0.686027 | 0.682638 | 0.710358 | 0.748745 |
| cg26662727 | 1.73E-04 | 19 |  | 0.70178 | 0.68816 | 0.709342 | 0.739105 |
| cg08796342 | 1.67E-04 | 14 | rs554526029;rs186044833 | 0.59539 | 0.60792 | 0.631674 | 0.54621 |
| cg21264724 | 1.66E-04 | 16 |  | 0.756303 | 0.739958 | 0.765078 | 0.770367 |
| cg24252430 | 1.65E-04 | 6 | rs141664968;rs570309501 | 0.352781 | 0.362386 | 0.383707 | 0.343543 |
| cg14906103 | 1.57E-04 | 4 | rs530312924;rs111983197; rs79121617;rs532655385 | 0.538339 | 0.523507 | 0.545371 | 0.617897 |
| cg20906197 | 1.54E-04 | 2 | rs571021930;rs62157663 | 0.638844 | 0.62818 | 0.65114 | 0.700483 |
| cg13677741 | 1.53E-04 | 1 | rs575742574;rs542621949 | 0.280133 | 0.283026 | 0.306057 | 0.240322 |
| cg26342537 | 1.53E-04 | 11 | rs34339888 | 0.409385 | 0.413837 | 0.436347 | 0.398151 |
| cg04541343 | 1.52E-04 | 10 | rs12412698;rs561420685; rs140266084;rs80211275 | 0.66577 | 0.653588 | 0.673933 | 0.717688 |
| cg00696269 | 1.52E-04 | 11 | rs551348986;rs571282709 | 0.578478 | 0.590128 | 0.611988 | 0.668937 |
| cg07201742 | 1.52E-04 | 19 |  | 0.625597 | 0.617601 | 0.639292 | 0.699451 |
| cg06457150 | 1.51E-04 | 7 | rs528386515 | 0.683398 | 0.676809 | 0.697269 | 0.742791 |
| cg23370394 | 1.50E-04 | 17 | rs376358163 | 0.781336 | 0.776725 | 0.801136 | 0.814887 |
| cg13675015 | 1.49E-04 | 6 | rs559557965; rs573084677; rs188408890 | 0.600067 | 0.585249 | 0.612349 | 0.664073 |
| cg12120701 | 1.48E-04 | 5 | rs114280860; rs199794271; rs184783670; rs552553935 | 0.680783 | 0.668638 | 0.690321 | 0.731877 |
| cg20262587 | 1.47E-04 | 18 | rs535279106 | 0.746334 | 0.764268 | 0.784644 | 0.744656 |
| cg11697861 | 1.43E-04 | 19 | rs149411804 | 0.728737 | 0.719622 | 0.741259 | 0.757352 |
| cg24853857 | 1.42E-04 | 11 | rs188454122 | 0.627735 | 0.646002 | 0.68124 | 0.600191 |
| cg15217637 | 1.39E-04 | 4 | rs116501938; rs532023527 | 0.643419 | 0.623475 | 0.645468 | 0.704302 |
| cg16713490 | 1.38E-04 | 17 | rs537791535; rs146164863 | 0.764981 | 0.768002 | 0.788055 | 0.811566 |
| cg07284463 | 1.36E-04 | 14 | rs376848909; rs528366452; rs185471225 | 0.658113 | 0.641773 | 0.665061 | 0.700604 |
| cg08384574 | 1.34E-04 | 16 | rs549182296 | 0.660769 | 0.654723 | 0.675312 | 0.718172 |
| cg20330031 | 1.31E-04 | 12 | rs139732201; rs539222867 | 0.756819 | 0.76554 | 0.786191 | 0.725906 |
| cg11213983 | 1.28E-04 | 17 | rs552149247 | 0.667896 | 0.679039 | 0.704721 | 0.633835 |
| cg06424992 | 1.26E-04 | 2 | rs563748592; rs577630431; rs543528783 | 0.663677 | 0.664458 | 0.687431 | 0.71023 |
| cg19572986 | 1.24E-04 | 17 | rs558322072; rs537495720; rs556132741 | 0.627836 | 0.621876 | 0.642973 | 0.684183 |
| cg14398876 | 1.24E-04 | 22 | rs186726667; rs534063893; rs553920643; rs573772789; rs113856063 | 0.563667 | 0.564065 | 0.586382 | 0.542338 |
| cg21558485 | 1.21E-04 | 9 |  | 0.751995 | 0.737202 | 0.767412 | 0.789224 |
| cg01769521 | 1.20E-04 | 8 | rs530308145; rs548409112 | 0.771751 | 0.760968 | 0.786483 | 0.816066 |
| cg06706483 | 1.18E-04 | 10 | rs140214457; rs568453129 ;rs143920514 | 0.58588 | 0.584279 | 0.613777 | 0.657355 |
| cg21569045 | 1.17E-04 | 12 | rs147889462 | 0.595733 | 0.58938 | 0.615483 | 0.646914 |
| cg18119851 | 1.17E-04 | 14 | rs544429475; rs562631556 | 0.642348 | 0.624958 | 0.65425 | 0.714397 |
| cg23798189 | 1.13E-04 | 3 | rs151071248 ;rs113294292; rs529722606 | 0.681728 | 0.684452 | 0.706779 | 0.643523 |
| cg26799677 | 1.12E-04 | 12 | rs193170892 | 0.496817 | 0.484437 | 0.505305 | 0.594789 |
| cg01908398 | 1.11E-04 | 22 | rs183753329; rs140350358; rs550978126 | 0.582461 | 0.571806 | 0.596557 | 0.623112 |
| cg24188441 | 1.09E-04 | 3 | rs570477086 | 0.505646 | 0.522968 | 0.545207 | 0.4862 |
| cg16825404 | 1.09E-04 | 21 | rs554462523; rs147311932 | 0.751642 | 0.746052 | 0.767953 | 0.797889 |
| cg13407065 | 1.07E-04 | 7 | rs567498071 | 0.709092 | 0.708503 | 0.737681 | 0.763545 |
| cg25738176 | 1.07E-04 | 17 | rs371458005 | 0.731658 | 0.729935 | 0.750008 | 0.778734 |
| cg00613587 | 1.07E-04 | 12 | rs531295227; rs189286796; rs192341681; rs115557041 | 0.351718 | 0.334808 | 0.359016 | 0.462184 |
| cg06754987 | 1.06E-04 | 1 | rs552953440; rs139438022 | 0.696644 | 0.688343 | 0.708351 | 0.743912 |
| cg07814932 | 1.06E-04 | 1 | rs200823597 | 0.390573 | 0.376313 | 0.398683 | 0.491865 |
| cg13353542 | 1.06E-04 | 9 | rs73653045; rs571565843 | 0.550191 | 0.561084 | 0.582082 | 0.507843 |
| cg01574233 | 1.06E-04 | 12 |  | 0.70979 | 0.696541 | 0.71826 | 0.740497 |
| cg06868474 | 1.06E-04 | 11 | rs540339132; rs76583617 | 0.735801 | 0.726488 | 0.74792 | 0.781462 |
| cg02169201 | 1.05E-04 | 20 | rs143904671 | 0.740565 | 0.734551 | 0.75717 | 0.778428 |
| cg01424562 | 1.05E-04 | 14 | rs569750320 | 0.676786 | 0.681348 | 0.704091 | 0.629047 |
| cg01804450 | 1.05E-04 | 22 | rs200823316; rs148834064; rs545160614 | 0.641232 | 0.634097 | 0.656891 | 0.706565 |
| cg22330841 | 1.04E-04 | 2 | rs570572995; rs539681842; rs13019824; rs576083074 | 0.790142 | 0.79658 | 0.823601 | 0.775793 |
| cg14280027 | 1.04E-04 | 11 | rs72910075; rs181869303 | 0.660831 | 0.650107 | 0.671741 | 0.698154 |
| cg06896316 | 1.03E-04 | 1 | rs602513; rs77470287 | 0.54947 | 0.565582 | 0.588137 | 0.60707 |
| cg15528722 | 1.03E-04 | 5 | rs556789177 | 0.611835 | 0.601638 | 0.625425 | 0.708386 |
| cg19748086 | 1.02E-04 | 12 | rs200373611 | 0.360358 | 0.372305 | 0.392392 | 0.312072 |
| cg13837145 | 1.01E-04 | 8 | rs190632888; rs13281818; rs73186329; rs574124632; rs75890310; rs559700851 | 0.448545 | 0.457286 | 0.482759 | 0.43975 |
| cg16548532 | 9.97E-05 | 9 | rs558544933 | 0.776817 | 0.772527 | 0.804132 | 0.817307 |
| cg12081267 | 9.96E-05 | 2 | rs117902906; rs114532263; rs116707109; rs368326259 | 0.572206 | 0.564155 | 0.592372 | 0.639212 |
| cg11114141 | 9.78E-05 | 2 | rs79729570; rs148831883 | 0.719109 | 0.72834 | 0.752264 | 0.700127 |
| cg25424986 | 9.77E-05 | 8 | rs187430392; rs563396039 | 0.537977 | 0.520033 | 0.540678 | 0.593355 |
| cg25944311 | 8.93E-05 | 8 |  | 0.689285 | 0.68092 | 0.704558 | 0.748024 |
| cg24527466 | 8.78E-05 | 13 | rs567277529 | 0.752968 | 0.769633 | 0.792484 | 0.727722 |
| cg19621475 | 8.59E-05 | 10 | rs576622371 | 0.637199 | 0.656379 | 0.677167 | 0.612127 |
| cg06599528 | 8.42E-05 | 21 | rs180893408 | 0.568248 | 0.578667 | 0.599607 | 0.561263 |
| cg22061957 | 8.38E-05 | 2 |  | 0.743361 | 0.758249 | 0.784925 | 0.699437 |
| cg07917930 | 8.28E-05 | 10 | rs549926402 | 0.841701 | 0.839794 | 0.865539 | 0.832409 |
| cg20666808 | 8.26E-05 | 15 | rs537612424; rs190142875 | 0.709677 | 0.695707 | 0.717459 | 0.778142 |
| cg08298632 | 8.23E-05 | 12 | rs35104932; rs67175201; rs540128631; rs11180385; rs112076923 | 0.511763 | 0.511491 | 0.533321 | 0.474781 |
| cg06784563 | 8.22E-05 | 18 | rs28446281; rs56384153; rs368417580; rs561442068; rs528915525 | 0.590845 | 0.605945 | 0.62606 | 0.55841 |
| cg17552056 | 8.07E-05 | 14 |  | 0.484592 | 0.503615 | 0.543151 | 0.468901 |
| cg09087803 | 8.06E-05 | 11 | rs188173266; rs529827707; rs548276464 | 0.626584 | 0.620456 | 0.647472 | 0.715013 |
| cg15605124 | 8.02E-05 | 1 | rs569935580; rs112301226; rs554995727; rs151165708; rs542337661; rs554009513 | 0.658426 | 0.645063 | 0.667861 | 0.698367 |
| cg03460132 | 8.01E-05 | 1 |  | 0.659784 | 0.660277 | 0.688013 | 0.736323 |
| cg06896091 | 7.97E-05 | 16 | rs540906328; rs565611830; rs185225762 | 0.648603 | 0.643834 | 0.666653 | 0.694438 |
| cg16659071 | 7.96E-05 | 2 | rs559251680; rs192102179 | 0.682605 | 0.68298 | 0.705379 | 0.760213 |
| cg07153421 | 7.56E-05 | 5 |  | 0.73374 | 0.731942 | 0.754288 | 0.766217 |
| cg10942336 | 7.51E-05 | 5 | rs6859702; rs112974619; rs541548405 | 0.654723 | 0.64483 | 0.66881 | 0.711437 |
| cg05994140 | 7.48E-05 | 2 | rs115673699; rs150576804 | 0.539432 | 0.544208 | 0.569992 | 0.524327 |
| cg00420390 | 7.29E-05 | 7 |  | 0.711278 | 0.699488 | 0.733882 | 0.785227 |
| cg07942182 | 7.09E-05 | 6 | rs3176350; rs532026768 | 0.709138 | 0.694553 | 0.718796 | 0.768369 |
| cg23092798 | 6.86E-05 | 10 |  | 0.578908 | 0.56708 | 0.591946 | 0.630619 |
| cg12636775 | 6.61E-05 | 2 | rs6547632; rs573669498; rs116607751 | 0.592108 | 0.582503 | 0.605043 | 0.644708 |
| cg06721411 | 6.20E-05 | 2 | rs79846504 | 0.4837 | 0.47237 | 0.494419 | 0.558584 |
| cg18865207 | 5.89E-05 | 1 | rs535422256; rs113865465; rs555630237 | 0.390448 | 0.389281 | 0.416481 | 0.357838 |
| cg09320924 | 5.82E-05 | 5 | rs73133671 | 0.534027 | 0.533535 | 0.565084 | 0.595771 |
| cg26867393 | 5.81E-05 | 8 | rs559271319 | 0.676526 | 0.66179 | 0.682109 | 0.746686 |
| cg02900757 | 5.80E-05 | 10 | rs558033933; rs576635102; rs139293591; rs555513408 | 0.678053 | 0.658871 | 0.687037 | 0.728963 |
| cg13947735 | 5.63E-05 | 17 | rs142539867 | 0.383523 | 0.372071 | 0.392245 | 0.477918 |
| cg10687163 | 5.50E-05 | 16 | rs543364239; rs181597215; rs528903371 | 0.699871 | 0.691351 | 0.717614 | 0.746488 |
| cg25056811 | 5.45E-05 | 3 | rs558644513; rs568873814 | 0.758183 | 0.776135 | 0.796477 | 0.762329 |
| cg21445207 | 5.41E-05 | 3 | rs186364196; rs76918604; rs530582699 | 0.741466 | 0.760524 | 0.789773 | 0.724956 |
| cg07865630 | 5.38E-05 | 6 | rs138344024; rs143842437 | 0.663157 | 0.649107 | 0.675881 | 0.744764 |
| cg04372213 | 5.38E-05 | 22 | rs539016697; rs182953080; rs200942768; rs200344564; rs199538534;rs146953411; rs137925620; rs141934766; rs200772932 | 0.597502 | 0.588215 | 0.617485 | 0.647883 |
| cg04108706 | 5.31E-05 | 1 | rs560669984; rs73038403; rs147331007 | 0.631714 | 0.614808 | 0.635334 | 0.689508 |
| cg13725885 | 5.19E-05 | 20 | rs183650639; rs151335323 | 0.587292 | 0.600943 | 0.623253 | 0.546017 |
| cg00729043 | 5.17E-05 | 21 | rs535040903 | 0.347467 | 0.328119 | 0.349593 | 0.451279 |
| cg00672192 | 5.07E-05 | 17 | rs571622277; rs533904058; rs141994894; rs79018681 | 0.583605 | 0.570457 | 0.590638 | 0.627719 |
| cg26815958 | 5.00E-05 | 17 | rs563728350; rs201566361; rs199870152; rs182307901 | 0.660337 | 0.649318 | 0.673141 | 0.715319 |
| cg11721435 | 4.95E-05 | 2 | rs148127587; rs183592440 | 0.716791 | 0.711343 | 0.732523 | 0.767028 |
| cg17694094 | 4.91E-05 | 3 | rs181943044; rs142549115 | 0.356082 | 0.356516 | 0.391962 | 0.318764 |
| cg03313849 | 4.89E-05 | 18 | rs140403415 | 0.613648 | 0.6034 | 0.624309 | 0.687664 |
| cg03461431 | 4.86E-05 | 1 | rs4660468 | 0.725023 | 0.728651 | 0.75153 | 0.777196 |
| cg06405246 | 4.73E-05 | 10 | rs572550889 | 0.734427 | 0.737295 | 0.757762 | 0.788898 |
| cg07219384 | 4.72E-05 | 6 | rs74824373 | 0.411818 | 0.430703 | 0.458972 | 0.375497 |
| cg14946715 | 4.63E-05 | 6 | rs182303774 | 0.542667 | 0.562163 | 0.586276 | 0.506992 |
| cg06625886 | 4.59E-05 | 3 | rs565802799 | 0.732622 | 0.747748 | 0.769138 | 0.703875 |
| cg22787849 | 4.58E-05 | 19 |  | 0.537076 | 0.530636 | 0.551761 | 0.533348 |
| cg01756512 | 4.57E-05 | 8 | rs540246995; rs551759083; rs566131739; rs1078908; rs554997733 | 0.186417 | 0.180812 | 0.216247 | 0.173306 |
| cg08371497 | 4.56E-05 | 22 | rs539391309; rs61531006; rs557529119; rs572906700 | 0.463446 | 0.443503 | 0.464997 | 0.519714 |
| cg04487720 | 4.45E-05 | 17 | rs536782808; rs548599285; rs569985860 | 0.632179 | 0.640877 | 0.662851 | 0.632176 |
| cg12581474 | 4.41E-05 | 1 | rs576650803; rs11310494 | 0.630504 | 0.612137 | 0.639657 | 0.697456 |
| cg17460054 | 4.35E-05 | 19 | rs538995946 | 0.731813 | 0.717988 | 0.739481 | 0.771178 |
| cg12914461 | 4.25E-05 | 7 | rs539061410; rs531164856 | 0.712458 | 0.708969 | 0.728981 | 0.75654 |
| cg05222829 | 4.21E-05 | 4 | rs76227793 | 0.855419 | 0.864603 | 0.88563 | 0.846059 |
| cg06482136 | 4.09E-05 | 17 | rs536280860; rs548488548; rs186436719 | 0.708886 | 0.723252 | 0.752925 | 0.664801 |
| cg14728609 | 4.08E-05 | 9 |  | 0.552599 | 0.53878 | 0.559122 | 0.620571 |
| cg05336633 | 3.95E-05 | 7 | rs551427753 | 0.793523 | 0.800645 | 0.820753 | 0.775228 |
| cg11409519 | 3.93E-05 | 22 | rs553539195; rs570530807; rs375742538 | 0.77019 | 0.767364 | 0.793427 | 0.800823 |
| cg13772414 | 3.86E-05 | 2 | rs552622665 | 0.425062 | 0.434626 | 0.45883 | 0.404763 |
| cg13817332 | 3.79E-05 | 2 | rs558711895; rs572251798; rs2166421; rs7421852 | 0.616513 | 0.60506 | 0.633199 | 0.692943 |
| cg16256242 | 3.76E-05 | 19 | rs563910185 | 0.607823 | 0.604271 | 0.62583 | 0.665377 |
| cg04424420 | 3.70E-05 | 22 | rs564429438; rs375788010; rs543585416 | 0.582048 | 0.569246 | 0.598342 | 0.660948 |
| cg17771504 | 3.61E-05 | 11 | rs534949719 | 0.785472 | 0.783304 | 0.807043 | 0.746581 |
| cg18414488 | 3.51E-05 | 17 | rs181798907; rs528199166; rs546338293; rs571460189 | 0.466151 | 0.472936 | 0.494396 | 0.439627 |
| cg13316148 | 3.42E-05 | 2 | rs184249841; rs144245767 | 0.741596 | 0.723739 | 0.749237 | 0.804189 |
| cg12311936 | 3.37E-05 | 2 | rs540184428; rs185826278 | 0.767797 | 0.764628 | 0.785898 | 0.729179 |
| cg24078454 | 3.33E-05 | 7 |  | 0.761002 | 0.778944 | 0.80117 | 0.746902 |
| cg22030053 | 3.32E-05 | 1 | rs141338245 | 0.567704 | 0.555053 | 0.577078 | 0.641802 |
| cg02271878 | 3.25E-05 | 12 |  | 0.717545 | 0.717994 | 0.740884 | 0.772609 |
| cg18042268 | 3.23E-05 | 4 | rs545797832 | 0.516261 | 0.498343 | 0.521812 | 0.605281 |
| cg04389899 | 3.22E-05 | 1 | rs114711568; rs527269225 | 0.802421 | 0.797 | 0.821545 | 0.776802 |
| cg00714531 | 3.05E-05 | 21 | rs373276123; rs564603445; rs146745022 | 0.722451 | 0.70555 | 0.728884 | 0.779424 |
| cg26145258 | 3.03E-05 | 11 | rs577235327; rs539948083 | 0.379486 | 0.361641 | 0.382981 | 0.456552 |
| cg16911301 | 2.88E-05 | 18 | rs552344655 | 0.495997 | 0.486325 | 0.513764 | 0.578654 |
| cg11409308 | 2.71E-05 | 3 | rs150586065; rs148308900 | 0.720645 | 0.721242 | 0.743295 | 0.787313 |
| cg09361958 | 2.67E-05 | 3 | rs142494132 | 0.687607 | 0.672383 | 0.695716 | 0.752914 |
| cg08841430 | 2.50E-05 | X |  | 0.656809 | 0.645443 | 0.666188 | 0.712736 |
| cg02144152 | 2.45E-05 | 6 | rs148208479; rs575156613; rs544289856; rs59866762 | 0.495914 | 0.478141 | 0.506089 | 0.581382 |
| cg01656259 | 2.23E-05 | 9 | rs148714336 | 0.505077 | 0.500637 | 0.525451 | 0.585804 |
| cg10671830 | 2.19E-05 | 4 | rs539849106 | 0.241698 | 0.250661 | 0.273901 | 0.231753 |
| cg26167930 | 2.17E-05 | 15 | rs115139033; rs537717615 | 0.537811 | 0.531825 | 0.553213 | 0.631266 |
| cg26484813 | 2.13E-05 | 11 | rs541669738; rs560321309 | 0.827542 | 0.817861 | 0.840585 | 0.806997 |
| cg14078897 | 2.04E-05 | 15 | rs537748210; rs549549911; rs190540281 | 0.734141 | 0.725448 | 0.747589 | 0.770794 |
| cg22425699 | 2.02E-05 | 5 | rs528176385; rs546633118 | 0.57967 | 0.568669 | 0.596808 | 0.678528 |
| cg11665416 | 1.98E-05 | 21 | rs568617533 | 0.565015 | 0.567009 | 0.600538 | 0.549906 |
| cg16890402 | 1.95E-05 | 1 | rs549075942; rs565298872; rs534323677 | 0.670943 | 0.664327 | 0.684996 | 0.734798 |
| cg14391258 | 1.74E-05 | 6 | rs552647921 | 0.818059 | 0.813116 | 0.833624 | 0.855538 |
| cg23925175 | 1.69E-05 | 9 | rs560727177 | 0.697626 | 0.680002 | 0.713147 | 0.749169 |
| cg16980393 | 1.65E-05 | 10 |  | 0.5694 | 0.555087 | 0.575235 | 0.663801 |
| cg09530779 | 1.63E-05 | 3 | rs576439058 | 0.448983 | 0.467511 | 0.493497 | 0.423022 |
| cg21298091 | 1.51E-05 | 22 | rs553532627; rs149019275; rs542224768 | 0.70293 | 0.696934 | 0.720583 | 0.772006 |
| cg26407930 | 1.51E-05 | 1 | rs567905347; rs532041240 | 0.704163 | 0.705477 | 0.726722 | 0.75002 |
| cg20137750 | 1.49E-05 | 1 | rs144637211; rs180991361; rs373707439; rs187102434 | 0.676759 | 0.676172 | 0.700213 | 0.759591 |
| cg23418197 | 1.47E-05 | 12 | rs117342721 | 0.348455 | 0.346095 | 0.367944 | 0.337795 |
| cg23149707 | 1.43E-05 | 17 | rs550997565; rs569425627; rs57781684; rs115280339; rs374668259 | 0.650734 | 0.633739 | 0.653762 | 0.701702 |
| cg00277804 | 1.31E-05 | 18 | rs543395841 | 0.607738 | 0.62367 | 0.647315 | 0.704095 |
| cg00392001 | 1.21E-05 | 1 | rs187137109 | 0.720774 | 0.709981 | 0.731663 | 0.764411 |
| cg13470125 | 1.13E-05 | 19 | rs546056984; rs139115782; rs575821196 | 0.60755 | 0.599126 | 0.621309 | 0.68389 |
| cg08237164 | 1.11E-05 | 19 | rs200791594; rs550738307; rs202061567 | 0.746171 | 0.729482 | 0.756349 | 0.770544 |
| cg15551881 | 1.09E-05 | 9 |  | 0.294623 | 0.311468 | 0.338652 | 0.268213 |
| cg24634058 | 1.08E-05 | 4 | rs150222927 | 0.606603 | 0.601315 | 0.624592 | 0.68433 |
| cg03619256 | 1.04E-05 | 7 | rs535834652 | 0.532516 | 0.543807 | 0.566525 | 0.587706 |
| cg00825551 | 9.42E-06 | 20 | rs542563537 | 0.612427 | 0.602274 | 0.625787 | 0.672851 |
| cg03437540 | 9.18E-06 | 22 | rs557840822 | 0.586226 | 0.572037 | 0.595681 | 0.654151 |
| cg04185639 | 8.81E-06 | 5 | rs140730183; rs372504318 | 0.59661 | 0.588034 | 0.61775 | 0.678962 |
| cg05914060 | 8.66E-06 | 11 | rs569527723; rs535401510 | 0.548391 | 0.531244 | 0.555656 | 0.639001 |
| cg19915900 | 8.55E-06 | 4 | rs578206227; rs113492750; rs540890467; rs79922041 | 0.681101 | 0.676157 | 0.697369 | 0.738903 |
| cg15789095 | 8.39E-06 | 1 | rs530252843 | 0.657346 | 0.656465 | 0.677588 | 0.714504 |
| cg03706086 | 8.15E-06 | 8 | rs142276729 | 0.370615 | 0.356103 | 0.385153 | 0.491918 |
| cg10685628 | 7.70E-06 | 1 |  | 0.407175 | 0.423137 | 0.4477 | 0.353906 |
| cg00765623 | 7.54E-06 | 20 | rs575233498; rs540796093; rs554081568 | 0.520097 | 0.503529 | 0.524309 | 0.589981 |
| cg11637118 | 7.13E-06 | 16 |  | 0.457348 | 0.463223 | 0.487765 | 0.452889 |
| cg21565676 | 6.28E-06 | 7 | rs188029172; rs62440485; rs79490106 | 0.523196 | 0.512598 | 0.536182 | 0.623373 |
| cg00694431 | 5.84E-06 | 8 | rs570050248 | 0.598154 | 0.586698 | 0.607304 | 0.675048 |
| cg23821480 | 5.75E-06 | 2 | rs116050682 | 0.763009 | 0.772322 | 0.798698 | 0.723127 |
| cg16160077 | 4.84E-06 | 12 | rs192295577; rs183718436 | 0.661652 | 0.643097 | 0.670622 | 0.733959 |
| cg03652595 | 4.76E-06 | 7 | rs573916558; rs544615659 | 0.547804 | 0.528665 | 0.549428 | 0.599469 |
| cg20973735 | 4.41E-06 | 11 | rs190085354 | 0.449373 | 0.439724 | 0.464975 | 0.395154 |
| cg16953845 | 4.15E-06 | 10 | rs185311300; rs145299376 | 0.520138 | 0.505234 | 0.531402 | 0.589721 |
| cg05906386 | 3.98E-06 | 6 | rs73567948; rs75264448 | 0.572923 | 0.556204 | 0.580792 | 0.688223 |
| cg21608071 | 3.97E-06 | 13 | rs563044582 | 0.594694 | 0.579778 | 0.599846 | 0.646444 |
| cg22357390 | 3.62E-06 | 12 | rs528798886 | 0.744381 | 0.740648 | 0.764282 | 0.789807 |
| cg03909307 | 3.23E-06 | 9 | rs573329393 | 0.81938 | 0.826414 | 0.848584 | 0.812145 |
| cg24597578 | 3.00E-06 | 17 |  | 0.760483 | 0.741356 | 0.762567 | 0.80266 |
| cg20599852 | 2.97E-06 | 16 |  | 0.598092 | 0.590766 | 0.612277 | 0.685246 |
| cg23529129 | 2.78E-06 | 3 | rs181698448 | 0.649887 | 0.635546 | 0.661071 | 0.729561 |
| cg04987734 | 2.78E-06 | 14 | rs564817018; rs577651137 | 0.374433 | 0.388729 | 0.419805 | 0.356617 |
| cg01059421 | 2.74E-06 | 6 | rs148477725 | 0.717732 | 0.73707 | 0.769299 | 0.699933 |
| cg08782002 | 2.59E-06 | 17 | rs117082006 | 0.525311 | 0.507491 | 0.528594 | 0.621836 |
| cg17964758 | 2.53E-06 | 1 | rs77981239; rs35801972; rs572118727 | 0.622559 | 0.622512 | 0.648483 | 0.700733 |
| cg06489569 | 2.42E-06 | 16 |  | 0.703015 | 0.686194 | 0.706416 | 0.765071 |
| cg13993179 | 9.90E-07 | 18 |  | 0.707974 | 0.707747 | 0.72818 | 0.777916 |
| cg25169445 | 7.09E-07 | 11 | rs544590246; rs562913050; rs530448503 | 0.723004 | 0.717726 | 0.738655 | 0.783505 |
| cg01507280 | 3.94E-07 | 7 | rs138781885; rs141530948 | 0.661929 | 0.652481 | 0.685388 | 0.728389 |
| cg25304758 | 3.78E-07 | 10 | rs181847835; rs535202007 | 0.535048 | 0.530468 | 0.551488 | 0.616935 |
| cg14505131 | 1.66E-07 | 8 | rs528129513; rs540501834 | 0.648584 | 0.638311 | 0.660578 | 0.749907 |
| cg22161874 | 1.09E-07 | 9 | rs568185651; rs45623432 | 0.60448 | 0.591842 | 0.616092 | 0.685399 |
| cg14524764 | 3.85E-08 | 22 | rs112955183; rs536759788 | 0.550822 | 0.532304 | 0.552484 | NA |
| cg11795976 | 3.36E-09 | 11 | rs550660538 | 0.765383 | 0.773026 | 0.795033 | 0.727212 |
| cg26676765 | 6.31E-11 | 16 |  | 0.592293 | 0.572342 | 0.592759 | 0.698874 |

# **Supplementary table 7. The identified CpG cites in group 6.**

| Target ID | p-value | Chromosome | SNP_ID | mean beta value in the 1st trimester | mean beta value in the 2nd trimester | mean beta value in the 3rd trimester | mean beta value in the after-delivery status |
| --- | --- | --- | --- | --- | --- | --- | --- |
| cg24360998 | 9.83E-04 | 1 | rs79829230 | 0.362687 | 0.369151 | 0.338647 | NA |
| cg01181415 | 9.77E-04 | 12 | rs527528077 | 0.263843 | 0.273773 | 0.252554 | 0.247499 |
| cg10355022 | 9.68E-04 | 5 | rs532091800;rs551876874 | 0.303937 | 0.285543 | 0.264132 | 0.385494 |
| cg05143156 | 9.48E-04 | 9 |  | 0.231271 | 0.214787 | 0.192766 | 0.321759 |
| cg25056645 | 9.30E-04 | 12 | rs189050186 | 0.311986 | 0.293853 | 0.268575 | 0.399311 |
| cg17251968 | 8.58E-04 | 16 | rs76092138 | 0.32955 | 0.313648 | 0.292265 | 0.420521 |
| cg12870776 | 8.31E-04 | 1 | rs530860536;rs552068974 | 0.708668 | 0.726337 | 0.697475 | 0.626148 |
| cg00320217 | 8.19E-04 | 2 | rs533433122;rs546511957 | 0.367796 | 0.359634 | 0.33891 | 0.463562 |
| cg09933065 | 7.22E-04 | X |  | 0.374997 | 0.366735 | 0.345436 | 0.339573 |
| cg02625241 | 7.08E-04 | 4 | rs141762292 | 0.325153 | 0.30821 | 0.286807 | 0.414574 |
| cg22639028 | 6.16E-04 | 9 | rs145825818 | 0.314497 | 0.29828 | 0.275157 | 0.384556 |
| cg15690717 | 6.01E-04 | 6 | rs542122749 | 0.241014 | 0.22793 | 0.206591 | 0.304563 |
| cg02447446 | 5.98E-04 | 9 | rs555439994;rs148756128 | 0.294857 | 0.284589 | 0.263939 | 0.344497 |
| cg24420366 | 5.98E-04 | 10 | rs373140745;rs186615950; rs531340076;rs76401084; rs149827782;rs182518068 | 0.319248 | 0.301603 | 0.280615 | 0.397292 |
| cg10447327 | 5.89E-04 | 2 | rs567406191;rs114161477 | 0.245118 | 0.230847 | 0.209748 | 0.34358 |
| cg08382137 | 5.54E-04 | 12 | rs559314202 | 0.638913 | 0.653452 | 0.632173 | 0.563936 |
| cg19110902 | 5.30E-04 | 6 | rs373687291 | 0.281383 | 0.292696 | 0.271962 | 0.250787 |
| cg26477557 | 5.09E-04 | 12 | rs146113157 | 0.204651 | 0.193263 | 0.173087 | 0.241124 |
| cg01580228 | 4.92E-04 | 11 | rs116938419 | 0.252817 | 0.246362 | 0.223188 | 0.33992 |
| cg17744261 | 3.61E-04 | 17 | rs567385949 | 0.27602 | 0.292914 | 0.269687 | 0.248676 |
| cg05599723 | 3.54E-04 | 1 | rs111717282;rs182415948; rs532278049 | 0.332048 | 0.312885 | 0.291223 | 0.414833 |
| cg15357334 | 3.38E-04 | 17 |  | 0.260922 | 0.243434 | 0.220309 | 0.322835 |
| cg23777206 | 3.21E-04 | 1 |  | 0.282046 | 0.280037 | 0.252263 | 0.390597 |
| cg01246178 | 2.85E-04 | 3 | rs329920 | 0.284374 | 0.264922 | 0.242015 | 0.389359 |
| cg06442240 | 2.84E-04 | 16 | rs557770866 | 0.409223 | 0.389852 | 0.369698 | 0.458097 |
| cg25266888 | 2.74E-04 | 11 | rs565394926;rs2075873; rs555115815;rs576535385 | 0.763801 | 0.78295 | 0.760245 | 0.810386 |
| cg01619417 | 2.55E-04 | 17 |  | 0.22685 | 0.220455 | 0.188386 | 0.310752 |
| cg17090424 | 2.43E-04 | 3 | rs148304166;rs536469740 | 0.800555 | 0.818985 | 0.793517 | 0.815909 |
| cg02647748 | 2.28E-04 | 17 | rs553082954;rs571717442 | 0.683891 | 0.692533 | 0.665735 | 0.737185 |
| cg13895650 | 1.65E-04 | 9 | rs181437778;rs77547622; rs186727234;rs142098069; rs548822994;rs561749349; rs73468084 | 0.440004 | 0.43017 | 0.40942 | 0.521411 |
| cg17833627 | 1.59E-04 | 12 | rs73405732;rs35855506 | 0.29003 | 0.277413 | 0.250509 | 0.401319 |
| cg02689908 | 1.50E-04 | 12 | rs78790259 | 0.245808 | 0.235131 | 0.2105 | 0.304509 |
| cg10839272 | 1.38E-04 | 2 | rs555502637;rs567363251 | 0.207981 | 0.193601 | 0.170875 | 0.260968 |
| cg15370339 | 1.20E-04 | 1 | rs544556642 | 0.226791 | 0.208879 | 0.188858 | 0.289279 |
| cg07805542 | 1.00E-04 | 1 | rs137868136;rs12564733; rs569199155;rs533408651 | 0.374392 | 0.360985 | 0.3349 | 0.421952 |
| cg02606488 | 9.95E-05 | 15 | rs187961831;rs573804085 | 0.571816 | 0.569291 | 0.543488 | 0.61737 |
| cg10920240 | 9.53E-05 | 5 | rs574153825 | 0.367052 | 0.367269 | 0.343793 | 0.478356 |
| cg19939909 | 9.46E-05 | X |  | 0.349806 | 0.367445 | 0.345177 | 0.316086 |
| cg22742385 | 9.41E-05 | 3 | rs574864920;rs56378811; rs186128050 | 0.587981 | 0.58648 | 0.565354 | 0.643412 |
| cg22736930 | 8.21E-05 | 5 | rs74950628;rs532624496 | 0.234101 | 0.235193 | 0.212253 | 0.315374 |
| cg18737789 | 7.92E-05 | 11 | rs558781547;rs192667736; rs538120889 | 0.814409 | 0.821506 | 0.800309 | 0.824754 |
| cg12322549 | 3.65E-05 | 19 | rs543332326;rs561215641 | 0.365514 | 0.351484 | 0.33126 | 0.477563 |
| cg00444151 | 3.24E-05 | 17 | rs546289081 | 0.466521 | 0.46523 | 0.44395 | 0.546456 |
| cg08812032 | 2.26E-05 | 4 | rs550287800 | 0.649693 | 0.651081 | 0.629263 | 0.693888 |
| cg09101692 | 1.38E-05 | 3 | rs554450681 | 0.343629 | 0.325215 | 0.302937 | 0.442074 |
| cg05629820 | 1.80E-06 | 20 | rs182233662;rs569771139 | 0.591069 | 0.598525 | 0.576378 | 0.666032 |
| cg04904784 | 1.35E-06 | 12 | rs529459622;rs112865756; rs11048813 | 0.347924 | 0.333956 | 0.313787 | 0.444252 |
| cg01055591 | 1.23E-07 | 22 |  | 0.483562 | 0.469836 | 0.449002 | 0.571981 |

# **Supplementary table 8. The identified CpG cites in group 7.**

| Target ID | p-value | Chromosome | SNP_ID | mean beta value in the 1st trimester | mean beta value in the 2nd trimester | mean beta value in the 3rd trimester | mean beta value in the after-delivery status |
| --- | --- | --- | --- | --- | --- | --- | --- |
| cg12022651 | 9.99E-04 | 1 | rs147884204;rs6677882 | 0.796895 | 0.819563 | 0.821868 | 0.793463 |
| cg03815117 | 9.99E-04 | 1 | rs181673494 | 0.827689 | 0.852665 | 0.838886 | 0.831369 |
| cg12606400 | 9.98E-04 | 18 | rs578122866;rs545315712; rs74638085;rs73420308 | 0.813618 | 0.840183 | 0.835132 | 0.815413 |
| cg23558966 | 9.97E-04 | 2 | rs139555606 | 0.636195 | 0.66329 | 0.665054 | 0.687393 |
| cg19802368 | 9.96E-04 | 7 | rs201109912;rs570062746; rs532187532 | 0.705789 | 0.733554 | 0.743689 | 0.713339 |
| cg00393031 | 9.94E-04 | 2 | rs551552801;rs148497882 | 0.749041 | 0.777726 | 0.771074 | 0.745072 |
| cg17074310 | 9.93E-04 | 6 | rs573842412 | 0.782143 | 0.808451 | 0.802081 | 0.764682 |
| cg23858394 | 9.91E-04 | 5 | rs73090266;rs77589906; rs78643930 | 0.724923 | 0.753041 | 0.753348 | 0.704537 |
| cg15602298 | 9.90E-04 | 1 | rs77100987 | 0.801266 | 0.828421 | 0.823686 | NA |
| cg10903500 | 9.89E-04 | 8 |  | 0.69491 | 0.721433 | 0.726853 | 0.658812 |
| cg04717258 | 9.86E-04 | 5 | rs558075800 | 0.797246 | 0.819341 | 0.810012 | 0.781709 |
| cg00270616 | 9.85E-04 | 2 | rs142139262 | 0.741935 | 0.772633 | 0.77437 | 0.740149 |
| cg02189269 | 9.84E-04 | 15 | rs59577009;rs551149162; rs150594582;rs139999951; rs538018299;rs553714901 | 0.805049 | 0.826811 | 0.822675 | 0.786797 |
| cg18406570 | 9.82E-04 | 11 | rs116673277;rs187662584 | 0.735541 | 0.756823 | 0.752884 | 0.784106 |
| cg04684983 | 9.80E-04 | 2 |  | 0.736703 | 0.764332 | 0.767056 | 0.716361 |
| cg25280810 | 9.80E-04 | 19 |  | 0.61437 | 0.639761 | 0.653893 | 0.570647 |
| cg22085487 | 9.75E-04 | 12 | rs191843379;rs373127197; rs142112063 | 0.727027 | 0.758108 | 0.760028 | 0.697274 |
| cg01669721 | 9.70E-04 | 14 | rs567883270;rs149749263; rs557113933 | 0.705972 | 0.736393 | 0.745812 | 0.683559 |
| cg16087432 | 9.69E-04 | 13 | rs12860901;rs546548105 | 0.765345 | 0.7905 | 0.797641 | 0.75446 |
| cg10821341 | 9.69E-04 | 5 | rs567362999 | 0.451272 | 0.489286 | 0.49008 | 0.450848 |
| cg06207791 | 9.67E-04 | 14 | rs535652272;rs553740444; rs146517260 | 0.615898 | 0.646716 | 0.642002 | 0.622161 |
| cg02668248 | 9.64E-04 | 19 | rs149231822 | 0.612294 | 0.638799 | 0.646884 | 0.573326 |
| cg17184593 | 9.62E-04 | 2 | rs142623499;rs576858992 | 0.599638 | 0.622506 | 0.628962 | NA |
| cg14005120 | 9.62E-04 | 9 | rs531197497;rs375358361 | 0.785426 | 0.808496 | 0.811464 | 0.779277 |
| cg18008625 | 9.57E-04 | 12 | rs370809040;rs562979899 | 0.708815 | 0.731567 | 0.738236 | 0.691197 |
| cg15055133 | 9.57E-04 | 5 | rs191235439;rs548724183; rs565699337;rs534692137 | 0.727725 | 0.749727 | 0.737914 | 0.700715 |
| cg11812438 | 9.57E-04 | 4 | rs6447614 | 0.780678 | 0.808783 | 0.810594 | 0.760656 |
| cg08180716 | 9.57E-04 | 11 | rs10750933 | 0.765636 | 0.79474 | 0.803882 | 0.74631 |
| cg15280233 | 9.54E-04 | 1 | rs75743002 | 0.78173 | 0.805755 | 0.814838 | 0.766821 |
| cg04927910 | 9.51E-04 | 11 | rs141116917;rs550698401; rs544131075;rs3741183; rs574499486;rs541917464; rs145877182 | 0.725733 | 0.774713 | 0.767464 | 0.668842 |
| cg16495782 | 9.50E-04 | 17 | rs564967647 | 0.648778 | 0.673888 | 0.668867 | 0.612504 |
| cg06188602 | 9.50E-04 | 1 |  | 0.640996 | 0.674277 | 0.667457 | 0.615524 |
| cg10020767 | 9.50E-04 | 7 | rs190642821;rs11974936; rs547981939 | 0.649603 | 0.675427 | 0.678209 | 0.619231 |
| cg22637658 | 9.49E-04 | 21 | rs536503587;rs531259710; rs148695959 | 0.544496 | 0.567864 | 0.573835 | 0.511294 |
| cg26664155 | 9.47E-04 | 5 | rs75139050;rs372870743; rs374731669;rs75737951 | 0.671762 | 0.694155 | 0.711245 | 0.673528 |
| cg23381998 | 9.46E-04 | 1 | rs536846061 | 0.744163 | 0.771243 | 0.766095 | 0.727933 |
| cg05021029 | 9.33E-04 | 10 |  | 0.69559 | 0.725646 | 0.737509 | 0.676838 |
| cg17317834 | 9.33E-04 | 4 | rs13127274;rs181193712 | 0.793194 | 0.818311 | 0.821668 | 0.792116 |
| cg25456728 | 9.30E-04 | 17 |  | 0.700665 | 0.721405 | 0.724158 | 0.674878 |
| cg17389227 | 9.29E-04 | 6 | rs182462653;rs552266048 | 0.736243 | 0.75744 | 0.767298 | 0.708706 |
| cg13424484 | 9.29E-04 | 10 | rs374670492 | 0.756447 | 0.784203 | 0.790686 | 0.733846 |
| cg19698472 | 9.29E-04 | 5 | rs137954732 | 0.790913 | 0.812433 | 0.803118 | 0.830844 |
| cg05790041 | 9.26E-04 | 3 | rs148180174;rs191827930; rs183115499 | 0.714609 | 0.743231 | 0.737954 | 0.679165 |
| cg14282114 | 9.22E-04 | 9 | rs543861290 | 0.199952 | 0.222636 | 0.233641 | 0.200085 |
| cg13399478 | 9.19E-04 | 5 |  | 0.740311 | 0.78007 | 0.769224 | 0.717859 |
| cg22034315 | 9.19E-04 | 2 | rs149256562;rs146175607; rs545499491 | 0.747527 | 0.769484 | 0.776629 | 0.799687 |
| cg13968079 | 9.17E-04 | 11 | rs4128739 | 0.825318 | 0.851864 | 0.837943 | 0.820316 |
| cg05368896 | 9.16E-04 | 1 | rs144895134;rs554049884; rs570734668 | 0.660513 | 0.70326 | 0.692986 | 0.658657 |
| cg19248404 | 9.16E-04 | 11 | rs565025337 | 0.774184 | 0.802963 | 0.795369 | 0.749601 |
| cg03847123 | 9.14E-04 | 3 | rs148003784;rs550860599 | 0.749848 | 0.772862 | 0.780225 | 0.727226 |
| cg13292401 | 9.14E-04 | 9 | rs115129095;rs193262918; rs115404585 | 0.735192 | 0.769022 | 0.769704 | 0.729601 |
| cg11318551 | 9.13E-04 | 8 | rs552832780 | 0.722171 | 0.747645 | 0.754638 | 0.684509 |
| cg04876396 | 9.13E-04 | 6 | rs377644170;rs554148309 | 0.621045 | 0.641615 | 0.632297 | 0.588011 |
| cg06536806 | 9.12E-04 | 5 | rs553938131;rs145407880; rs79351912 | 0.685559 | 0.706282 | 0.711641 | 0.674385 |
| cg14842480 | 9.11E-04 | 11 |  | 0.808932 | 0.839302 | 0.820147 | 0.798848 |
| cg26119123 | 9.08E-04 | 12 | rs184822618;rs150578157; rs188959109 | 0.785411 | 0.807052 | 0.821056 | 0.77003 |
| cg18603538 | 9.02E-04 | 7 | rs17151919 | 0.66117 | 0.682099 | 0.678083 | 0.637491 |
| cg01819937 | 9.00E-04 | 10 | rs141524232;rs566605732; rs527927522 | 0.803684 | 0.841901 | 0.834965 | 0.805957 |
| cg23717593 | 9.00E-04 | 1 | rs529696100 | 0.728465 | 0.755283 | 0.773162 | 0.712051 |
| cg09307605 | 8.97E-04 | 21 |  | 0.700069 | 0.726983 | 0.731061 | 0.679191 |
| cg04165933 | 8.95E-04 | 16 | rs565075802 | 0.794514 | 0.825035 | 0.820527 | 0.795339 |
| cg10031643 | 8.93E-04 | 8 | rs139743582 | 0.775765 | 0.798653 | 0.818086 | 0.833847 |
| cg04147160 | 8.93E-04 | 8 | rs116560047 | 0.694405 | 0.726599 | 0.718583 | 0.65843 |
| cg16379091 | 8.92E-04 | 11 | rs551001460;rs77612739 | 0.751702 | 0.773726 | 0.775949 | 0.741007 |
| cg17444479 | 8.90E-04 | 10 | rs370009002;rs148319446; rs544783489;rs532599832 | 0.785489 | 0.827532 | 0.820448 | 0.732945 |
| cg14360023 | 8.87E-04 | 1 | rs11465756 | 0.786808 | 0.811716 | 0.79659 | 0.752168 |
| cg21281086 | 8.87E-04 | 5 | rs531128361 | 0.75554 | 0.802395 | 0.785361 | 0.721027 |
| cg01592682 | 8.86E-04 | 9 | rs192523551;rs567863984; rs7860512 | 0.713288 | 0.734228 | 0.723683 | 0.762929 |
| cg26284167 | 8.86E-04 | 15 | rs76558172;rs572484866; rs546413309;rs564717593 | 0.799065 | 0.829159 | 0.81431 | 0.796432 |
| cg14569771 | 8.85E-04 | 4 | rs149623550 | 0.538164 | 0.573614 | 0.569218 | 0.525845 |
| cg20639011 | 8.83E-04 | 5 |  | 0.759732 | 0.784945 | 0.785608 | 0.73794 |
| cg13438353 | 8.80E-04 | 19 | rs191543375;rs12972423; rs536917295 | 0.633188 | 0.664112 | 0.683582 | 0.605819 |
| cg25065000 | 8.80E-04 | 7 |  | 0.696826 | 0.721292 | 0.728889 | 0.67863 |
| cg22971407 | 8.75E-04 | 16 |  | 0.747164 | 0.783989 | 0.779909 | 0.738251 |
| cg01314670 | 8.72E-04 | 2 | rs557313265;rs575993604; rs540166074 | 0.752931 | 0.774581 | 0.782514 | 0.740276 |
| cg10589408 | 8.71E-04 | 1 | rs116363353 | 0.687045 | 0.719119 | 0.716664 | 0.666952 |
| cg24148874 | 8.68E-04 | 5 | rs193203720;rs556347450 | 0.784076 | 0.82059 | 0.828841 | 0.729432 |
| cg14872824 | 8.67E-04 | 10 | rs529804310 | 0.792681 | 0.814892 | 0.808191 | 0.777153 |
| cg11033495 | 8.66E-04 | 14 | rs566654324;rs371712886; rs558809793 | 0.816844 | 0.837095 | 0.848853 | 0.807643 |
| cg15849527 | 8.66E-04 | 5 | rs551769876 | 0.645556 | 0.672497 | 0.67794 | 0.625586 |
| cg20370507 | 8.66E-04 | 13 |  | 0.695159 | 0.740598 | 0.747676 | 0.672943 |
| cg15439110 | 8.65E-04 | 3 | rs147325604 | 0.72316 | 0.751832 | 0.750049 | 0.678427 |
| cg01884851 | 8.63E-04 | 3 | rs144091196;rs115943963; rs566927205 | 0.680095 | 0.715105 | 0.73302 | 0.644999 |
| cg04854462 | 8.62E-04 | 18 | rs12326181;rs528519; rs603764;rs549433516; rs570766773 | 0.774444 | 0.820297 | 0.820681 | 0.790935 |
| cg10978719 | 8.61E-04 | 15 | rs142746865;rs531289265 | 0.773128 | 0.802 | 0.808982 | 0.74319 |
| cg02428556 | 8.58E-04 | 5 | rs149026890;rs536037761; rs556355875 | 0.76222 | 0.790805 | 0.795075 | 0.747321 |
| cg19386337 | 8.58E-04 | 4 | rs532049534;rs78628528; rs184025105 | 0.718494 | 0.740876 | 0.744917 | 0.686378 |
| cg07076751 | 8.56E-04 | 6 | rs530783278;rs145951780; rs115557175 | 0.731903 | 0.753928 | 0.771884 | 0.696021 |
| cg16988611 | 8.56E-04 | 10 | rs569391396;rs76080317; rs554981407 | 0.567763 | 0.589718 | 0.587136 | 0.549917 |
| cg03062944 | 8.55E-04 | 10 | rs576132667;rs543524434; rs74939166 | 0.798963 | 0.827024 | 0.820957 | 0.750059 |
| cg18577239 | 8.54E-04 | 12 | rs186410729;rs546368116; rs112292163;rs558176501 | 0.802551 | 0.825709 | 0.823876 | 0.796407 |
| cg18714170 | 8.52E-04 | 13 | rs576411965;rs141806197; rs185712288;rs112520636 | 0.650833 | 0.675135 | 0.682323 | 0.609783 |
| cg23947175 | 8.51E-04 | 13 | rs533933015;rs116642723 | 0.75108 | 0.77192 | 0.790066 | 0.73282 |
| cg12525665 | 8.50E-04 | 1 | rs540807839;rs559096836; rs529953914;rs541681435 | 0.786111 | 0.80822 | 0.820884 | 0.787824 |
| cg17894779 | 8.49E-04 | 3 | rs9829623;rs113713943; rs192974039;rs151061082 | 0.72631 | 0.755699 | 0.758133 | 0.717359 |
| cg00718409 | 8.49E-04 | 6 | rs138222305;rs572210298; rs541024341 | 0.784012 | 0.806416 | 0.801067 | NA |
| cg01560057 | 8.48E-04 | 1 | rs189548003 | 0.802364 | 0.823082 | 0.842951 | 0.793675 |
| cg10022505 | 8.45E-04 | 10 | rs377174165;rs3097725; rs572645642;rs531796183; rs541971666 | 0.726855 | 0.764811 | 0.771638 | 0.714135 |
| cg26154042 | 8.45E-04 | 3 | rs145987312 | 0.637612 | 0.662469 | 0.669958 | 0.615058 |
| cg12445613 | 8.43E-04 | 6 | rs550930296;rs569414448; rs372176788 | 0.66158 | 0.701886 | 0.707114 | 0.616825 |
| cg06184813 | 8.41E-04 | 4 | rs143224769;rs545906811 | 0.850342 | 0.873863 | 0.860311 | 0.857666 |
| cg08156039 | 8.41E-04 | 1 | rs536923703 | 0.78098 | 0.810821 | 0.81484 | 0.774128 |
| cg20687262 | 8.40E-04 | 10 | rs548051945;rs561361196; rs138870446 | 0.620023 | 0.671183 | 0.667025 | 0.592487 |
| cg00789792 | 8.39E-04 | 2 | rs144769045;rs557633131 | 0.768484 | 0.798997 | 0.799081 | 0.756108 |
| cg01685380 | 8.37E-04 | 3 | rs181244406;rs117952165; rs183868253 | 0.637382 | 0.682305 | 0.681202 | 0.610687 |
| cg15622269 | 8.37E-04 | 7 | rs544686624 | 0.727032 | 0.755113 | 0.755104 | 0.71538 |
| cg11591520 | 8.37E-04 | 4 | rs146205184 | 0.746739 | 0.769565 | 0.769699 | 0.732643 |
| cg05587627 | 8.36E-04 | 11 | rs183548278;rs138589273 | 0.648481 | 0.67557 | 0.685559 | 0.620096 |
| cg06456255 | 8.35E-04 | 4 | rs564095312;rs187372143; rs189954637 | 0.812328 | 0.834251 | 0.824811 | 0.797833 |
| cg02131510 | 8.35E-04 | 1 | rs78993007 | 0.785857 | 0.817834 | 0.818602 | 0.779745 |
| cg06360629 | 8.33E-04 | 8 |  | 0.835604 | 0.856459 | 0.859248 | 0.819225 |
| cg10516573 | 8.31E-04 | 1 | rs541518532;rs78175542 | 0.727998 | 0.749909 | 0.757824 | 0.715668 |
| cg26717964 | 8.30E-04 | 9 | rs574005161 | 0.840898 | 0.864524 | 0.858596 | 0.822061 |
| cg03989598 | 8.29E-04 | 20 | rs569131936;rs533034069 | 0.616036 | 0.6501 | 0.66043 | 0.593944 |
| cg06333725 | 8.27E-04 | 10 | rs577339433;rs540053757; rs4312019 | 0.774799 | 0.803311 | 0.806763 | 0.758303 |
| cg08836972 | 8.23E-04 | 6 |  | 0.310241 | 0.335191 | 0.352361 | 0.307244 |
| cg15533189 | 8.22E-04 | 6 | rs557709138 | 0.635474 | 0.664254 | 0.678323 | 0.609881 |
| cg00753399 | 8.21E-04 | 2 | rs141268047 | 0.692427 | 0.733077 | 0.740812 | 0.689908 |
| cg07500118 | 8.21E-04 | 4 |  | 0.804673 | 0.82773 | 0.837531 | 0.794291 |
| cg20146177 | 8.15E-04 | 8 | rs538694048;rs549319120; rs567337364;rs534765154 | 0.65341 | 0.673549 | 0.681712 | 0.631933 |
| cg01557687 | 8.15E-04 | 3 | rs538366907 | 0.774936 | 0.797174 | 0.806421 | 0.771864 |
| cg07377701 | 8.14E-04 | 3 | rs185826205 | 0.79834 | 0.820388 | 0.822252 | 0.767966 |
| cg07642816 | 8.12E-04 | 2 | rs568472998;rs188148650 | 0.794668 | 0.821789 | 0.82944 | 0.791383 |
| cg26045457 | 8.10E-04 | 17 | rs533044884;rs73326898; rs139938380 | 0.754267 | 0.776386 | 0.784942 | 0.728494 |
| cg10269608 | 8.08E-04 | 4 | rs146988362 | 0.745135 | 0.769141 | 0.767914 | 0.727506 |
| cg06906951 | 8.07E-04 | 5 | rs566963705 | 0.726368 | 0.752076 | 0.76499 | 0.713986 |
| cg23453570 | 8.05E-04 | 2 |  | 0.748673 | 0.770857 | 0.783332 | 0.732179 |
| cg14885762 | 8.04E-04 | 17 | rs553289438 | 0.635174 | 0.655817 | 0.669746 | 0.607862 |
| cg16943704 | 8.03E-04 | 18 | rs187425350;rs538920609 | 0.679055 | 0.700826 | 0.712929 | 0.657596 |
| cg05566945 | 8.03E-04 | 6 | rs77753095 | 0.799838 | 0.821452 | 0.824651 | 0.792406 |
| cg14925847 | 8.01E-04 | 21 | rs548738664;rs567243816; rs537797719;rs532536474 | 0.765644 | 0.803965 | 0.791123 | 0.734162 |
| cg07938480 | 8.00E-04 | 16 | rs549791198;rs139471214; rs569863582;rs538454490 | 0.541118 | 0.571115 | 0.580214 | 0.531165 |
| cg15781898 | 8.00E-04 | 16 | rs577087293 | 0.652827 | 0.679013 | 0.696428 | 0.635065 |
| cg20469625 | 7.99E-04 | 8 | rs145219749 | 0.757241 | 0.777535 | 0.786784 | 0.748649 |
| cg20521963 | 7.98E-04 | 3 | rs377355509 | 0.80926 | 0.830514 | 0.821792 | 0.801551 |
| cg26288692 | 7.98E-04 | 1 |  | 0.736636 | 0.761628 | 0.776886 | 0.735966 |
| cg09727784 | 7.97E-04 | 1 | rs370549992;rs140780079; rs143100015;rs146140626 | 0.835863 | 0.85594 | 0.864031 | 0.826032 |
| cg00929270 | 7.97E-04 | 20 | rs2427402;rs138548150 | 0.584638 | 0.60593 | 0.619283 | 0.55671 |
| cg18054375 | 7.96E-04 | 10 | rs574412508;rs4980079; rs4980080;rs142874284; rs545624550 | 0.704317 | 0.741431 | 0.729793 | 0.656225 |
| cg16255827 | 7.96E-04 | 2 | rs548038713 | 0.692671 | 0.727746 | 0.74692 | 0.714691 |
| cg12696761 | 7.90E-04 | 18 | rs566459234;rs562198476; rs527812508 | 0.611554 | 0.641557 | 0.631444 | 0.577108 |
| cg05508558 | 7.89E-04 | 1 |  | 0.404513 | 0.425186 | 0.441108 | 0.405727 |
| cg20730477 | 7.89E-04 | 3 |  | 0.811712 | 0.83402 | 0.830439 | 0.810211 |
| cg09841150 | 7.88E-04 | 6 | rs34617466;rs543833159; rs115702813 | 0.806636 | 0.833909 | 0.83188 | 0.787973 |
| cg26950080 | 7.86E-04 | 16 | rs553364802;rs578083331 | 0.722926 | 0.743981 | 0.755019 | 0.768696 |
| cg00369659 | 7.86E-04 | 21 | rs16981806;rs182524750 | 0.733028 | 0.756899 | 0.753423 | 0.709427 |
| cg25518708 | 7.84E-04 | 12 |  | 0.739961 | 0.767332 | 0.772505 | 0.731886 |
| cg11190798 | 7.76E-04 | 8 | rs9298009;rs144235802 | 0.752359 | 0.781901 | 0.791585 | 0.740389 |
| cg04443690 | 7.75E-04 | 6 | rs533417671;rs551900975; rs73535161 | 0.816026 | 0.843785 | 0.851586 | 0.799438 |
| cg09568833 | 7.73E-04 | 20 | rs117719728 | 0.64642 | 0.666686 | 0.675583 | 0.625853 |
| cg11421768 | 7.69E-04 | 5 | rs144367885;rs373344877; rs551882467;rs190411650; rs531737166;rs114566324 | 0.882146 | 0.916509 | 0.902113 | 0.866901 |
| cg03369432 | 7.66E-04 | 5 | rs180849207 | 0.767528 | 0.79283 | 0.796413 | 0.760819 |
| cg26404422 | 7.65E-04 | 11 | rs541715727 | 0.636367 | 0.661085 | 0.662396 | 0.590798 |
| cg02561723 | 7.62E-04 | 7 | rs138650084 | 0.581314 | 0.601625 | 0.611261 | 0.564574 |
| cg22039071 | 7.62E-04 | 4 | rs562282590 | 0.735871 | 0.769419 | 0.757338 | 0.703912 |
| cg23213278 | 7.56E-04 | X |  | 0.548435 | 0.571297 | 0.583139 | 0.546481 |
| cg02432506 | 7.56E-04 | 7 | rs145098617;rs566009803 | 0.850983 | 0.872597 | 0.872915 | 0.881997 |
| cg02531439 | 7.56E-04 | 7 | rs539911558 | 0.78196 | 0.803673 | 0.808989 | 0.763715 |
| cg06695406 | 7.55E-04 | 12 | rs527247827;rs554363264 | 0.758916 | 0.785099 | 0.783629 | 0.747298 |
| cg06135309 | 7.55E-04 | 10 | rs145204778;rs185138167; rs137901240 | 0.797871 | 0.819784 | 0.825645 | 0.78254 |
| cg26783962 | 7.54E-04 | 6 | rs543898294;rs560599855; rs533530584 | 0.735101 | 0.759486 | 0.779083 | 0.729486 |
| cg11924124 | 7.53E-04 | 6 | rs534139336 | 0.747304 | 0.781583 | 0.789832 | 0.727379 |
| cg04450994 | 7.53E-04 | 6 |  | 0.614807 | 0.639232 | 0.647835 | 0.552699 |
| cg04950342 | 7.52E-04 | 3 | rs540254721 | 0.771301 | 0.792599 | 0.78452 | 0.737705 |
| cg08237959 | 7.48E-04 | 3 | rs193092638;rs576677010 | 0.708926 | 0.740178 | 0.741484 | 0.693322 |
| cg22730137 | 7.42E-04 | 14 |  | 0.362346 | 0.382435 | 0.38991 | 0.386659 |
| cg03578193 | 7.40E-04 | 2 | rs112123011;rs76117056 | 0.7822 | 0.8039 | 0.813957 | 0.75919 |
| cg04258067 | 7.39E-04 | 4 |  | 0.71215 | 0.735816 | 0.742996 | 0.698113 |
| cg14983293 | 7.38E-04 | 11 | rs185767251;rs2742509; rs576126016 | 0.799751 | 0.821237 | 0.81139 | 0.834956 |
| cg02198144 | 7.37E-04 | 16 | rs149056508;rs144722491; rs542441898;rs8051098; rs71392762;rs8049860 | 0.815275 | 0.839846 | 0.831427 | 0.793021 |
| cg08535727 | 7.35E-04 | 5 | rs530433365;rs189012235; rs150974097 | 0.824846 | 0.848567 | 0.846263 | 0.823118 |
| cg07132091 | 7.31E-04 | 11 | rs538637689;rs546544960; rs558792512 | 0.816301 | 0.836791 | 0.841241 | 0.857604 |
| cg01219306 | 7.31E-04 | 16 | rs72771643;rs74799807 | 0.774836 | 0.809149 | 0.803397 | 0.78074 |
| cg26646061 | 7.30E-04 | 1 | rs574698900;rs140292040; rs10537703 | 0.766712 | 0.796918 | 0.806527 | 0.744844 |
| cg08230983 | 7.30E-04 | 6 | rs34179446;rs151147851; rs532555127;rs547715985 | 0.830743 | 0.854785 | 0.86856 | 0.821389 |
| cg07676455 | 7.28E-04 | 10 |  | 0.819317 | 0.851949 | 0.852346 | 0.821241 |
| cg25373630 | 7.28E-04 | 18 | rs533310603 | 0.71537 | 0.748764 | 0.749657 | 0.680962 |
| cg24461742 | 7.25E-04 | 10 | rs564369291 | 0.801354 | 0.825102 | 0.832875 | 0.798777 |
| cg10135682 | 7.25E-04 | 10 | rs561931425;rs573809611; rs540940367 | 0.777187 | 0.805375 | 0.808279 | 0.775341 |
| cg12694452 | 7.24E-04 | 12 |  | 0.81239 | 0.833888 | 0.835373 | 0.798282 |
| cg23466166 | 7.22E-04 | 8 | rs532592394;rs559243486 | 0.77135 | 0.79388 | 0.790277 | 0.752159 |
| cg10223809 | 7.21E-04 | 12 |  | 0.414535 | 0.440539 | 0.438008 | 0.410814 |
| cg21807318 | 7.20E-04 | 20 | rs554341880 | 0.763051 | 0.783211 | 0.791847 | 0.766101 |
| cg20318281 | 7.20E-04 | 9 | rs180756124;rs139784832 | 0.811381 | 0.840916 | 0.845777 | 0.809646 |
| cg05363382 | 7.16E-04 | 17 |  | 0.717205 | 0.740922 | 0.741447 | 0.691753 |
| cg06578716 | 7.16E-04 | 19 |  | 0.664919 | 0.694638 | 0.684399 | 0.6581 |
| cg00671823 | 7.16E-04 | 16 |  | 0.774431 | 0.803312 | 0.805569 | 0.770826 |
| cg06414499 | 7.13E-04 | 10 | rs542722541 | 0.761541 | 0.788915 | 0.804048 | 0.745373 |
| cg02981078 | 7.12E-04 | 12 |  | 0.697087 | 0.723686 | 0.721437 | NA |
| cg10206601 | 7.12E-04 | 8 | rs182377960;rs17707988; rs187817824;rs528294956 | 0.791302 | 0.811672 | 0.811876 | 0.77106 |
| cg16740064 | 7.10E-04 | 3 | rs183462236;rs558103469 | 0.674696 | 0.704501 | 0.715103 | 0.649979 |
| cg01363618 | 7.08E-04 | 22 | rs188449694;rs372035309 | 0.716409 | 0.737651 | 0.740927 | 0.769095 |
| cg10992339 | 7.07E-04 | 4 | rs575718785;rs544483305 | 0.792943 | 0.816181 | 0.833172 | 0.790182 |
| cg09939441 | 7.06E-04 | 10 |  | 0.809059 | 0.830171 | 0.819437 | 0.782376 |
| cg25343204 | 7.06E-04 | 7 | rs199505514 | 0.820353 | 0.848413 | 0.848067 | 0.81877 |
| cg16519151 | 7.05E-04 | 16 | rs538998531;rs559013032; rs576467264 | 0.695792 | 0.718584 | 0.73143 | 0.684849 |
| cg26157600 | 7.04E-04 | 14 | rs4902047 | 0.777273 | 0.800586 | 0.805909 | 0.737933 |
| cg08622666 | 7.04E-04 | 6 |  | 0.673174 | 0.706997 | 0.715877 | 0.639136 |
| cg14411221 | 7.03E-04 | 14 | rs559879161 | 0.777086 | 0.801516 | 0.798668 | 0.753791 |
| cg24446385 | 7.03E-04 | 2 | rs536184911;rs187188758; rs569769241 | 0.697929 | 0.732537 | 0.739624 | 0.668836 |
| cg13828440 | 6.98E-04 | 12 | rs553045265;rs572884608 | 0.761499 | 0.785207 | 0.795665 | 0.742563 |
| cg17894948 | 6.98E-04 | 14 | rs572897962;rs540271858 | 0.782719 | 0.805245 | 0.798042 | 0.758005 |
| cg25704278 | 6.96E-04 | 6 | rs184699787 | 0.799383 | 0.820544 | 0.825104 | 0.779581 |
| cg20708282 | 6.88E-04 | 5 | rs114765241 | 0.742261 | 0.773068 | 0.754648 | 0.720853 |
| cg04593745 | 6.88E-04 | 2 | rs563867283;rs112751693 | 0.7478 | 0.775372 | 0.784306 | 0.710597 |
| cg13153329 | 6.87E-04 | 14 | rs141914360 | 0.811242 | 0.840294 | 0.835257 | 0.801752 |
| cg13876315 | 6.87E-04 | 9 | rs376457885;rs201041365; rs200098613;rs139051036 | 0.626959 | 0.649348 | 0.649045 | 0.609618 |
| cg23823076 | 6.86E-04 | 15 | rs144264634;rs200450083; rs111243334 | 0.80067 | 0.833133 | 0.840715 | 0.781494 |
| cg11635702 | 6.84E-04 | 2 |  | 0.766849 | 0.79159 | 0.808916 | NA |
| cg26713159 | 6.84E-04 | 11 | rs548907145;rs73439983; rs537686526 | 0.751528 | 0.774019 | 0.775849 | 0.732882 |
| cg12393108 | 6.83E-04 | 11 | rs186622048;rs115649534; rs533597650 | 0.779278 | 0.814421 | 0.813739 | 0.76851 |
| cg22374742 | 6.81E-04 | 2 | rs571373067;rs35847245 | 0.836184 | 0.870747 | 0.866747 | 0.816707 |
| cg08264747 | 6.80E-04 | 7 | rs547255028;rs567054638; rs1174985 | 0.789103 | 0.815479 | 0.823277 | 0.784715 |
| cg03867746 | 6.78E-04 | 14 | rs561746548;rs146464523; rs550710396;rs75194159 | 0.712206 | 0.750284 | 0.746671 | 0.697049 |
| cg17822177 | 6.78E-04 | 2 | rs573732732 | 0.792186 | 0.81542 | 0.805349 | 0.77493 |
| cg06880420 | 6.76E-04 | 1 | rs540558461;rs534532721; rs555989849 | 0.759582 | 0.790142 | 0.802563 | 0.748529 |
| cg21536828 | 6.70E-04 | 12 | rs532970047 | 0.544032 | 0.57868 | 0.574382 | 0.539037 |
| cg16801161 | 6.69E-04 | 12 | rs530320699 | 0.737118 | 0.762563 | 0.762149 | 0.690012 |
| cg25464235 | 6.68E-04 | 13 | rs532069941;rs551851994; rs72633945 | 0.701168 | 0.724727 | 0.718102 | 0.751946 |
| cg16929850 | 6.68E-04 | 10 | rs556458944 | 0.621165 | 0.644869 | 0.652905 | 0.598806 |
| cg20899997 | 6.67E-04 | 12 |  | 0.708425 | 0.737058 | 0.749368 | 0.696474 |
| cg07354209 | 6.66E-04 | 2 | rs144981704 | 0.671197 | 0.702986 | 0.707375 | 0.666689 |
| cg03062881 | 6.66E-04 | 21 | rs530567184 | 0.626577 | 0.650099 | 0.666806 | 0.615659 |
| cg14416443 | 6.66E-04 | 17 | rs542775482;rs561872813 | 0.738682 | 0.764555 | 0.751588 | 0.748676 |
| cg13376352 | 6.65E-04 | 6 | rs117860362;rs536564815; rs114174368 | 0.708526 | 0.755703 | 0.750549 | 0.678563 |
| cg14302428 | 6.65E-04 | 9 | rs570323625;rs184450558 | 0.769956 | 0.79048 | 0.79176 | 0.747199 |
| cg15601452 | 6.64E-04 | 1 |  | 0.713226 | 0.750573 | 0.759329 | 0.680433 |
| cg04857881 | 6.61E-04 | 16 | rs561304586;rs180713067; rs548593712;rs567090244 | 0.764983 | 0.78989 | 0.792993 | 0.737116 |
| cg05147995 | 6.57E-04 | 5 | rs192518112 | 0.695293 | 0.720952 | 0.737035 | 0.685811 |
| cg02790372 | 6.56E-04 | 11 | rs535149498;rs148655987; rs536387339 | 0.776411 | 0.814167 | 0.795433 | 0.752737 |
| cg04891815 | 6.56E-04 | 4 | rs115372996;rs190857545; rs575306814 | 0.594676 | 0.625391 | 0.623957 | 0.589152 |
| cg16320210 | 6.56E-04 | 12 | rs560093377;rs533656314; rs545431515;rs551097009 | 0.689416 | 0.722187 | 0.731008 | 0.633808 |
| cg09404380 | 6.52E-04 | 14 | rs537451712 | 0.663312 | 0.697454 | 0.703866 | 0.648242 |
| cg04442806 | 6.52E-04 | 3 | rs528443793 | 0.712235 | 0.736351 | 0.751578 | 0.694904 |
| cg24996659 | 6.50E-04 | 14 | rs532457294 | 0.54571 | 0.56639 | 0.575105 | 0.524861 |
| cg12197357 | 6.49E-04 | 1 | rs192672622;rs559939006 | 0.646564 | 0.677896 | 0.688841 | 0.625389 |
| cg10790672 | 6.49E-04 | 5 | rs60166823;rs551084921 | 0.52585 | 0.552649 | 0.557699 | 0.48208 |
| cg08746762 | 6.48E-04 | 4 |  | 0.676913 | 0.70145 | 0.70033 | 0.655061 |
| cg00849955 | 6.48E-04 | 6 | rs577852558 | 0.808295 | 0.837673 | 0.83724 | 0.790905 |
| cg04489577 | 6.47E-04 | 4 |  | 0.760448 | 0.789792 | 0.784098 | 0.763529 |
| cg25855415 | 6.46E-04 | 3 | rs145756646;rs550991643; rs569576468 | 0.809975 | 0.832151 | 0.822917 | 0.778529 |
| cg24854175 | 6.44E-04 | 5 | rs539515453;rs140103184 | 0.738596 | 0.774251 | 0.763483 | 0.718104 |
| cg00136413 | 6.44E-04 | 6 | rs114388040 | 0.743975 | 0.767783 | 0.780469 | 0.699769 |
| cg07198181 | 6.43E-04 | 5 | rs191723641;rs183957627 | 0.696795 | 0.72133 | 0.730524 | 0.661602 |
| cg03155097 | 6.41E-04 | 5 | rs80126809;rs150170379; rs138563237 | 0.734324 | 0.767433 | 0.769185 | 0.722537 |
| cg06595154 | 6.37E-04 | 11 | rs140873738;rs553239750 | 0.725095 | 0.752719 | 0.754268 | 0.708744 |
| cg16158905 | 6.33E-04 | 7 | rs141644709;rs553974748 | 0.764243 | 0.790041 | 0.773095 | 0.722907 |
| cg00887881 | 6.31E-04 | 2 | rs569492829 | 0.577703 | 0.599594 | 0.60665 | 0.561099 |
| cg00894555 | 6.30E-04 | 11 | rs184626255;rs74540766 | 0.789386 | 0.81339 | 0.816916 | 0.790806 |
| cg09905051 | 6.29E-04 | 5 | rs529803716 | 0.764609 | 0.788263 | 0.798065 | 0.748318 |
| cg12266591 | 6.28E-04 | 18 | rs577749418;rs545013324; rs560890160;rs544138416 | 0.688976 | 0.722941 | 0.735595 | 0.689652 |
| cg24414037 | 6.26E-04 | 3 | rs556306960 | 0.834371 | 0.857181 | 0.852066 | 0.832986 |
| cg01656396 | 6.20E-04 | 9 | rs200755526;rs76223779 | 0.7176 | 0.765399 | 0.754325 | 0.728837 |
| cg06242392 | 6.20E-04 | X | rs35609266 | 0.6546 | 0.694349 | 0.687979 | 0.628819 |
| cg08153812 | 6.19E-04 | 20 | rs193245035;rs184496314; rs559390829;rs533306403; rs551378159 | 0.69426 | 0.727713 | 0.733142 | 0.66619 |
| cg25639921 | 6.17E-04 | 16 |  | 0.773898 | 0.796214 | 0.797175 | 0.76557 |
| cg06676778 | 6.17E-04 | 5 | rs533515187;rs143067440 | 0.534734 | 0.566146 | 0.573618 | 0.530445 |
| cg18507290 | 6.16E-04 | 1 | rs10925156;rs545655994; rs184755598;rs116639252 | 0.751466 | 0.793447 | 0.791867 | 0.697118 |
| cg21737421 | 6.16E-04 | 18 | rs540670855 | 0.729962 | 0.755794 | 0.773037 | 0.703021 |
| cg24475948 | 6.15E-04 | 1 |  | 0.794598 | 0.819578 | 0.823386 | 0.780525 |
| cg00322767 | 6.13E-04 | 12 |  | 0.748027 | 0.77433 | 0.789026 | 0.704604 |
| cg21256616 | 6.12E-04 | 1 | rs555970471;rs74088022 | 0.476555 | 0.502166 | 0.508022 | 0.470801 |
| cg10749585 | 6.11E-04 | 7 | rs551345068;rs570418685; rs539441086 | 0.773281 | 0.798424 | 0.805913 | 0.760213 |
| cg07713288 | 6.11E-04 | 3 | rs575091941 | 0.688374 | 0.710457 | 0.718125 | 0.644546 |
| cg24037746 | 6.11E-04 | 3 | rs192393532;rs149633887; rs578174694;rs146128441 | 0.782478 | 0.813477 | 0.799166 | 0.761418 |
| cg19293468 | 6.10E-04 | 17 | rs570936914;rs187398682 | 0.779122 | 0.799323 | 0.813875 | 0.748279 |
| cg09468177 | 6.10E-04 | 9 | rs553930608;rs186900707 | 0.220334 | 0.242614 | 0.234651 | 0.235483 |
| cg20606891 | 6.10E-04 | 2 | rs544564178;rs138610916 | 0.806213 | 0.836018 | 0.838359 | 0.812653 |
| cg21584691 | 6.09E-04 | 1 | rs186468083 | 0.781155 | 0.803358 | 0.820492 | 0.790779 |
| cg04689890 | 6.08E-04 | 5 | rs113564644;rs2548984; rs10064992 | 0.783087 | 0.821167 | 0.813011 | 0.783891 |
| cg08461692 | 6.07E-04 | 17 | rs530964450 | 0.429093 | 0.4585 | 0.457579 | 0.431114 |
| cg01857925 | 6.06E-04 | 11 | rs147765427 | 0.789499 | 0.823262 | 0.818744 | 0.794295 |
| cg15976200 | 6.06E-04 | 13 | rs575777066 | 0.67126 | 0.69142 | 0.710722 | 0.628015 |
| cg00272070 | 6.05E-04 | 2 | rs560227231;rs3771174; rs552568012 | 0.769208 | 0.793995 | 0.793855 | 0.749902 |
| cg07053784 | 6.02E-04 | 3 | rs556805854 | 0.724385 | 0.751257 | 0.751682 | 0.70875 |
| cg17288647 | 5.99E-04 | 11 | rs151327217;rs138554059 | 0.812039 | 0.832382 | 0.837246 | 0.804951 |
| cg26580824 | 5.97E-04 | 5 | rs560461657;rs529127866; rs145060907 | 0.686288 | 0.710953 | 0.718278 | 0.664319 |
| cg07217653 | 5.96E-04 | 15 | rs4965777;rs113309741; rs141517028 | 0.777342 | 0.804657 | 0.806698 | 0.773633 |
| cg26713533 | 5.93E-04 | 12 |  | 0.709087 | 0.732279 | 0.722601 | 0.683557 |
| cg13450951 | 5.93E-04 | 3 | rs572822256 | 0.780638 | 0.803705 | 0.801804 | 0.751923 |
| cg19458350 | 5.90E-04 | 14 | rs546323258;rs568984002; rs192976306 | 0.365902 | 0.392373 | 0.401131 | 0.359373 |
| cg16236275 | 5.90E-04 | 6 | rs575599094;rs6904310; rs564875790 | 0.776125 | 0.804162 | 0.80934 | 0.761365 |
| cg11243173 | 5.90E-04 | 12 | rs143864592 | 0.766412 | 0.787421 | 0.787056 | 0.815756 |
| cg07995893 | 5.87E-04 | 7 | rs554768221 | 0.74742 | 0.773047 | 0.778357 | 0.721906 |
| cg06043208 | 5.86E-04 | 5 | rs190699249 | 0.681279 | 0.707208 | 0.722952 | 0.640243 |
| cg19276014 | 5.84E-04 | 10 | rs555701540;rs565724414; rs371343080;rs53485 3980; rs557928224;rs577693392; rs543489683;rs556747335; rs573325386;rs542171312 | 0.878017 | 0.901426 | 0.913611 | 0.845676 |
| cg18365377 | 5.81E-04 | 7 | rs534111289;rs538254881 | 0.77146 | 0.797632 | 0.807879 | 0.771927 |
| cg15218868 | 5.80E-04 | 6 | rs559257754 | 0.74966 | 0.771382 | 0.785406 | 0.716126 |
| cg11959746 | 5.80E-04 | 12 | rs116489822;rs140973119 | 0.781355 | 0.804612 | 0.805246 | 0.765624 |
| cg12668144 | 5.78E-04 | 2 | rs542873939 | 0.687157 | 0.714104 | 0.703586 | 0.675193 |
| cg14160339 | 5.78E-04 | 2 |  | 0.553295 | 0.576987 | 0.584848 | 0.530779 |
| cg00759807 | 5.72E-04 | 16 | rs115071747;rs574436595 | 0.781729 | 0.803259 | 0.810335 | NA |
| cg05995064 | 5.72E-04 | 7 |  | 0.614279 | 0.647075 | 0.635764 | 0.605077 |
| cg16039157 | 5.72E-04 | 15 | rs564444319 | 0.732819 | 0.76308 | 0.766934 | 0.702557 |
| cg19006766 | 5.70E-04 | 12 | rs11048271 | 0.742786 | 0.771512 | 0.76968 | 0.740617 |
| cg14053955 | 5.69E-04 | 6 | rs184740064 | 0.59922 | 0.635793 | 0.638682 | 0.577749 |
| cg25303871 | 5.69E-04 | 10 | rs542786338 | 0.771384 | 0.80855 | 0.817479 | 0.786629 |
| cg21441438 | 5.69E-04 | 2 | rs578150239 | 0.774299 | 0.796154 | 0.80713 | 0.746527 |
| cg24802001 | 5.67E-04 | 14 | rs186815945 | 0.775548 | 0.800655 | 0.808635 | 0.769738 |
| cg05824517 | 5.65E-04 | 20 | rs112684892;rs186789752 | 0.725094 | 0.750837 | 0.764625 | 0.703265 |
| cg06246967 | 5.64E-04 | 15 | rs571704272;rs539086572; rs143445185;rs544912179; rs28671139 | 0.69009 | 0.71478 | 0.722279 | 0.655098 |
| cg07820423 | 5.60E-04 | 4 |  | 0.786982 | 0.814229 | 0.809593 | 0.761149 |
| cg08750995 | 5.59E-04 | 15 | rs554941771;rs574787623 | 0.751581 | 0.7801 | 0.770578 | 0.80567 |
| cg00154920 | 5.58E-04 | 1 | rs576421397;rs474414; rs3820411 | 0.590447 | 0.621399 | 0.624576 | 0.573538 |
| cg10699171 | 5.58E-04 | 6 | rs564404552;rs142155402; rs528317437 | 0.595509 | 0.634754 | 0.644262 | NA |
| cg25952037 | 5.56E-04 | 3 |  | 0.691128 | 0.714735 | 0.709085 | 0.666263 |
| cg16321672 | 5.56E-04 | 7 | rs76956998 | 0.715439 | 0.744064 | 0.736744 | 0.674444 |
| cg07930673 | 5.55E-04 | 2 |  | 0.717259 | 0.740757 | 0.74977 | 0.695604 |
| cg21625514 | 5.53E-04 | 1 | rs191362961 | 0.797006 | 0.819058 | 0.808938 | 0.770005 |
| cg10941394 | 5.53E-04 | 8 | rs540937080 | 0.813264 | 0.836108 | 0.827551 | 0.79797 |
| cg14785557 | 5.51E-04 | 21 |  | 0.731981 | 0.764134 | 0.762521 | 0.714036 |
| cg19142373 | 5.50E-04 | 17 |  | 0.635949 | 0.667008 | 0.685113 | 0.626267 |
| cg23999078 | 5.49E-04 | 6 | rs572443276 | 0.718761 | 0.744678 | 0.764408 | 0.685667 |
| cg13414010 | 5.48E-04 | 22 | rs112630104;rs140858989 | 0.744117 | 0.769369 | 0.78375 | 0.729823 |
| cg16848730 | 5.48E-04 | 3 | rs536001905;rs75335114; rs188921999;rs150818012 | 0.650501 | 0.685619 | 0.703884 | 0.636327 |
| cg20973598 | 5.47E-04 | 21 | rs372413488 | 0.701594 | 0.748544 | 0.757375 | 0.68176 |
| cg17618942 | 5.47E-04 | 19 | rs191336265;rs183148249; rs561229814;rs187486488 | 0.786387 | 0.814358 | 0.811042 | 0.78302 |
| cg08209849 | 5.44E-04 | 1 | rs185880979;rs546908974 ; rs562024837;rs77434440; rs368293066 | 0.712225 | 0.747338 | 0.761052 | 0.687064 |
| cg11713660 | 5.43E-04 | 17 |  | 0.634188 | 0.668378 | 0.658989 | 0.599133 |
| cg06791489 | 5.43E-04 | 1 |  | 0.716928 | 0.741385 | 0.738089 | 0.697871 |
| cg25108994 | 5.42E-04 | 2 | rs148301641 | 0.768868 | 0.804595 | 0.80266 | 0.74876 |
| cg07457855 | 5.37E-04 | 10 | rs536040341 | 0.688006 | 0.713705 | 0.728366 | 0.680182 |
| cg26277641 | 5.37E-04 | 2 | rs16865064 | 0.7763 | 0.804558 | 0.79609 | 0.749985 |
| cg16768840 | 5.35E-04 | 18 | rs150517748 | 0.724851 | 0.745227 | 0.74211 | 0.689402 |
| cg07880098 | 5.34E-04 | 21 | rs574955204 | 0.737682 | 0.759405 | 0.759129 | 0.711329 |
| cg26706520 | 5.33E-04 | 17 | rs547334651;rs560955382; rs529615212 | 0.676586 | 0.702793 | 0.720116 | 0.658837 |
| cg05080322 | 5.33E-04 | 14 |  | 0.766387 | 0.787388 | 0.796692 | 0.748377 |
| cg18608673 | 5.32E-04 | 1 | rs540137134;rs192793438 | 0.788861 | 0.822728 | 0.822739 | 0.747963 |
| cg00811275 | 5.31E-04 | 1 | rs562824188;rs6700007 | 0.767447 | 0.787859 | 0.783752 | 0.760692 |
| cg08435557 | 5.29E-04 | 12 |  | 0.779238 | 0.822477 | 0.819259 | 0.785023 |
| cg26854588 | 5.29E-04 | 17 | rs548981716;rs567176633; rs187875514;rs190082799 | 0.715793 | 0.745288 | 0.748531 | 0.680231 |
| cg14075484 | 5.26E-04 | 18 | rs368348795;rs562462713; rs531545793;rs548459347; rs185308998; rs190412672; rs114300037 | 0.806857 | 0.838106 | 0.837356 | 0.821488 |
| cg13668053 | 5.26E-04 | 22 | rs187109448 | 0.713718 | 0.734001 | 0.748488 | 0.766954 |
| cg04165577 | 5.25E-04 | 2 | rs569845045 | 0.76954 | 0.790796 | 0.785057 | 0.749016 |
| cg24286098 | 5.24E-04 | 11 | rs118054884;rs560470069 | 0.677131 | 0.697822 | 0.691972 | 0.663456 |
| cg11079441 | 5.23E-04 | 8 | rs529308180;rs547437711; rs566046139;rs143053690 | 0.744714 | 0.767136 | 0.77695 | 0.719475 |
| cg01595702 | 5.23E-04 | 5 | rs531139952;rs147781306; rs572028043;rs542844469; rs554461298 | 0.781137 | 0.805769 | 0.804086 | 0.763089 |
| cg12126243 | 5.21E-04 | 14 | rs555003897;rs183130375; rs147703384 | 0.793452 | 0.822408 | 0.818868 | 0.781429 |
| cg21379388 | 5.20E-04 | 3 | rs533519940;rs184914622; rs567438917;rs190117621 | 0.540828 | 0.585032 | 0.577896 | 0.536372 |
| cg26895405 | 5.20E-04 | 11 | rs572471050;rs531641892; rs541568198 | 0.744346 | 0.769838 | 0.76994 | 0.727577 |
| cg23671795 | 5.19E-04 | 14 |  | 0.537235 | 0.558528 | 0.573357 | 0.49839 |
| cg17293195 | 5.18E-04 | 11 | rs76604776;rs534001085; rs74995203;rs181096970; rs578170269; rs545509474; rs185733489 | 0.684207 | 0.746774 | 0.751964 | 0.707105 |
| cg17644137 | 5.18E-04 | 11 | rs530233617;rs115640750 | 0.623228 | 0.646579 | 0.648769 | 0.605458 |
| cg08962640 | 5.18E-04 | 17 | rs151194489 | 0.616306 | 0.637045 | 0.640132 | 0.59076 |
| cg02737575 | 5.15E-04 | 10 | rs118002521;rs149263024; rs144461425 | 0.843481 | 0.865987 | 0.858929 | 0.846392 |
| cg05646531 | 5.15E-04 | 12 | rs186040741;rs190709178; rs542690310;rs560954950 | 0.633235 | 0.658862 | 0.658978 | 0.605702 |
| cg11727721 | 5.13E-04 | 18 | rs146553769;rs373078912; rs553600648 | 0.769219 | 0.794228 | 0.795616 | 0.742743 |
| cg04061878 | 5.12E-04 | 13 | rs145458275 | 0.758223 | 0.799605 | 0.804429 | 0.743918 |
| cg04923352 | 5.09E-04 | 9 | rs114031037;rs192159923; rs541043370;rs144367260 | 0.705299 | 0.732204 | 0.741973 | 0.697015 |
| cg20370799 | 5.08E-04 | 9 | rs375523035;rs557384783 | 0.592157 | 0.615063 | 0.633554 | 0.5659 |
| cg23720708 | 5.08E-04 | 17 | rs182774393 | 0.759284 | 0.797295 | 0.781584 | 0.73005 |
| cg13587802 | 5.08E-04 | 12 | rs542558589 | 0.801488 | 0.823762 | 0.825985 | 0.778947 |
| cg18855174 | 5.08E-04 | 5 |  | 0.743564 | 0.767977 | 0.781112 | 0.735301 |
| cg23489236 | 5.07E-04 | 5 | rs141613268;rs112054727 | 0.737472 | 0.76984 | 0.760736 | 0.692944 |
| cg00197431 | 5.06E-04 | 7 | rs552894124;rs572691035; rs535277286;rs62469963 | 0.757981 | 0.786216 | 0.789043 | 0.723145 |
| cg08588453 | 5.04E-04 | 11 | rs493315;rs75391788 | 0.669178 | 0.69816 | 0.704699 | 0.657658 |
| cg08655413 | 5.04E-04 | 20 | rs571970010;rs4810832; rs145224962 | 0.816934 | 0.838988 | 0.840517 | 0.785693 |
| cg04602414 | 5.03E-04 | 1 | rs113984758;rs193170082 | 0.803282 | 0.832191 | 0.825946 | 0.794938 |
| cg24464500 | 5.01E-04 | 1 | rs78915964 | 0.671397 | 0.702009 | 0.718393 | 0.652057 |
| cg25180759 | 5.00E-04 | 12 |  | 0.826698 | 0.850453 | 0.850445 | 0.820117 |
| cg02635500 | 4.98E-04 | 4 | rs571462804;rs535394453 | 0.820396 | 0.842195 | 0.844077 | 0.826277 |
| cg01355030 | 4.95E-04 | 5 |  | 0.786724 | 0.81615 | 0.821501 | 0.779326 |
| cg16581631 | 4.94E-04 | 8 | rs563523709;rs529591548; rs549271105;rs559955483 | 0.763932 | 0.78833 | 0.792968 | 0.752577 |
| cg12154406 | 4.94E-04 | 21 | rs186689446;rs534769320 | 0.695285 | 0.717873 | 0.707256 | 0.670042 |
| cg21894525 | 4.93E-04 | 1 | rs577316301 | 0.76819 | 0.799955 | 0.798997 | 0.746694 |
| cg08852619 | 4.90E-04 | 4 | rs533730682;rs545952130 | 0.77177 | 0.794452 | 0.801214 | 0.747401 |
| cg26003848 | 4.89E-04 | 5 |  | 0.798334 | 0.820954 | 0.83446 | 0.780106 |
| cg01823887 | 4.89E-04 | 1 | rs4657374 | 0.756731 | 0.785991 | 0.795863 | 0.745542 |
| cg23787700 | 4.88E-04 | 12 | rs559043965;rs181151952; rs541630537 | 0.752554 | 0.773867 | 0.785484 | 0.731632 |
| cg23195522 | 4.88E-04 | 11 | rs116926403;rs192363010; rs545128036 | 0.713428 | 0.73695 | 0.756799 | 0.712973 |
| cg04495317 | 4.85E-04 | 8 | rs73663132;rs540575173; rs183151050;rs574462406; rs541689117 | 0.662683 | 0.685126 | 0.704264 | 0.625377 |
| cg05963956 | 4.85E-04 | 3 | rs79046940;rs542576457; rs563951629 | 0.829267 | 0.851841 | 0.856726 | 0.804116 |
| cg15277915 | 4.84E-04 | 11 | rs545841361;rs185691340 | 0.769268 | 0.791139 | 0.793692 | 0.810513 |
| cg12407884 | 4.83E-04 | 6 | rs530444522;rs550603243; rs149438031 | 0.771517 | 0.792055 | 0.784455 | 0.741226 |
| cg22624902 | 4.82E-04 | 2 | rs550238184;rs571658284; rs538400115;rs553572707 | 0.680173 | 0.706266 | 0.7227 | 0.627341 |
| cg16770312 | 4.79E-04 | 20 | rs57781510 | 0.774002 | 0.79613 | 0.803901 | 0.753087 |
| cg16802888 | 4.79E-04 | 1 | rs558923304;rs115742088 | 0.768269 | 0.793206 | 0.801634 | 0.763311 |
| cg14535531 | 4.75E-04 | 18 | rs189713123;rs570911489; rs199996781 | 0.490128 | 0.511422 | 0.527421 | 0.482053 |
| cg15389191 | 4.70E-04 | 11 | rs554952943;rs77671502 | 0.718571 | 0.767558 | 0.763737 | NA |
| cg16852756 | 4.67E-04 | 7 | rs574923394;rs545478929 | 0.814521 | 0.835152 | 0.844369 | 0.805752 |
| cg13828397 | 4.67E-04 | 11 | rs140814048;rs537088660; rs200752813;rs555076254 | 0.795833 | 0.816571 | 0.802111 | 0.825552 |
| cg07543199 | 4.66E-04 | 10 | rs566643519;rs534027617; rs553495809;rs578152209; rs141167931;rs114094487 | 0.793163 | 0.825657 | 0.818862 | 0.792985 |
| cg03985195 | 4.65E-04 | 12 | rs185060832 | 0.794716 | 0.81576 | 0.805231 | 0.769716 |
| cg02745316 | 4.62E-04 | 6 | rs375669292;rs141976261; rs911532 | 0.77992 | 0.804073 | 0.816387 | 0.768886 |
| cg16629664 | 4.62E-04 | 12 | rs143065920 | 0.714585 | 0.742571 | 0.752622 | 0.711541 |
| cg19638572 | 4.59E-04 | 1 | rs562594761;rs531022269 | 0.430167 | 0.453434 | 0.452475 | 0.406716 |
| cg23867923 | 4.58E-04 | 17 | rs549293671;rs569517204; rs3935192;rs558325044; rs529232833 | 0.41297 | 0.437513 | 0.443219 | 0.404937 |
| cg24503369 | 4.57E-04 | 2 | rs541702863;rs553705004 | 0.744237 | 0.769105 | 0.769794 | 0.731816 |
| cg07842386 | 4.55E-04 | 1 | rs11465758;rs572214044; rs541128139 | 0.681239 | 0.722359 | 0.72515 | 0.671849 |
| cg13542764 | 4.55E-04 | 14 | rs371180876;rs141770037; rs368634706 | 0.752243 | 0.783622 | 0.786065 | 0.7169 |
| cg00357958 | 4.52E-04 | 12 |  | 0.593403 | 0.613445 | 0.613659 | 0.527199 |
| cg26498539 | 4.51E-04 | 2 | rs138170796;rs180881489; rs148897730 | 0.711347 | 0.735188 | 0.743642 | 0.704925 |
| cg19696012 | 4.51E-04 | 8 | rs575025724 | 0.765667 | 0.789972 | 0.801325 | 0.745913 |
| cg22966160 | 4.51E-04 | 11 | rs559727918;rs144843560 | 0.807571 | 0.828458 | 0.814288 | 0.780037 |
| cg06939970 | 4.48E-04 | 4 | rs559772930 | 0.773727 | 0.798438 | 0.80177 | 0.76057 |
| cg06134364 | 4.44E-04 | 3 | rs550619702;rs567338478; rs75554350;rs552823468 | 0.801133 | 0.826247 | 0.837532 | 0.797183 |
| cg23295464 | 4.44E-04 | 7 | rs533728464;rs7795239 | 0.739848 | 0.763157 | 0.767773 | 0.728693 |
| cg05728281 | 4.43E-04 | 15 | rs181929822 | 0.695375 | 0.732783 | 0.750338 | 0.663899 |
| cg12402620 | 4.43E-04 | 8 |  | 0.696919 | 0.732261 | 0.728501 | 0.649174 |
| cg14597804 | 4.41E-04 | 5 | rs538715510;rs191630895 | 0.800282 | 0.820288 | 0.832339 | 0.784655 |
| cg22726405 | 4.40E-04 | 22 |  | 0.685849 | 0.715158 | 0.719726 | 0.664437 |
| cg10274606 | 4.39E-04 | 14 | rs575493912;rs192936621; rs150084173 | 0.801597 | 0.825924 | 0.827889 | 0.792169 |
| cg23975251 | 4.38E-04 | 1 | rs144500348;rs549435732; rs534071771 | 0.499556 | 0.543938 | 0.553767 | 0.495009 |
| cg16008223 | 4.37E-04 | 20 | rs545313722;rs558918478; rs185769631 | 0.729618 | 0.756473 | 0.757531 | 0.716297 |
| cg05425286 | 4.36E-04 | 17 | rs572627431 | 0.789696 | 0.812033 | 0.809961 | 0.837298 |
| cg19727803 | 4.35E-04 | 11 | rs601154 | 0.819064 | 0.843673 | 0.843184 | 0.812178 |
| cg22371961 | 4.35E-04 | 1 | rs569989581;rs572367389 | 0.713438 | 0.742931 | 0.747819 | 0.692288 |
| cg21278731 | 4.34E-04 | 4 | rs147984056;rs143370475; rs56036270 | 0.80586 | 0.828782 | 0.834848 | 0.790908 |
| cg26176362 | 4.30E-04 | 1 | rs578151393 | 0.661788 | 0.69657 | 0.689995 | 0.636892 |
| cg02952984 | 4.30E-04 | 15 | rs531120319;rs551120316 | 0.770577 | 0.79809 | 0.806138 | 0.771311 |
| cg09802688 | 4.27E-04 | 1 | rs552263312;rs545413984 | 0.215844 | 0.2421 | 0.242737 | 0.205275 |
| cg11818644 | 4.27E-04 | 5 | rs559839293;rs534701692 | 0.836909 | 0.857871 | 0.855862 | 0.837865 |
| cg01294868 | 4.27E-04 | 12 | rs200943283;rs540175050; rs557462000;rs113672095; rs2302746 | 0.607121 | 0.629612 | 0.636761 | 0.576135 |
| cg21066155 | 4.26E-04 | 12 |  | 0.751463 | 0.778928 | 0.788461 | 0.693072 |
| cg04312209 | 4.26E-04 | 5 | rs138981776;rs182533042; rs1353252 | 0.465387 | 0.501327 | 0.519883 | 0.45513 |
| cg05999010 | 4.25E-04 | 3 | rs541495234;rs188374703 | 0.827409 | 0.84787 | 0.85303 | 0.808143 |
| cg12927730 | 4.20E-04 | 2 | rs568665808;rs534104798 | 0.494775 | 0.516561 | 0.518608 | 0.476263 |
| cg10190839 | 4.20E-04 | 3 | rs533976176;rs555239863; rs567916378 | 0.835719 | 0.857624 | 0.851658 | 0.828593 |
| cg07209385 | 4.16E-04 | 8 | rs181557994;rs143234628 | 0.831425 | 0.851657 | 0.844784 | 0.828093 |
| cg10625096 | 4.16E-04 | 6 | rs550826951;rs567598635; rs142972343 | 0.75886 | 0.787724 | 0.796074 | 0.762389 |
| cg24489634 | 4.13E-04 | 2 | rs563229757;rs575517895; rs542523589 | 0.692428 | 0.719457 | 0.732108 | 0.660945 |
| cg19138376 | 4.11E-04 | 2 | rs543528597 | 0.7074 | 0.738289 | 0.752858 | 0.683122 |
| cg22798362 | 4.11E-04 | 17 | rs142074018;rs183384687 | 0.747585 | 0.773418 | 0.780488 | 0.708102 |
| cg11434837 | 4.08E-04 | 9 | rs139682247;rs200591527; rs188600149 | 0.628432 | 0.649092 | 0.65857 | 0.61913 |
| cg21798574 | 4.07E-04 | 20 | rs181878113 | 0.814974 | 0.842326 | 0.855478 | 0.793843 |
| cg18700007 | 4.05E-04 | 12 |  | 0.657704 | 0.685021 | 0.694888 | 0.625487 |
| cg10895682 | 4.04E-04 | 1 | rs72647403 | 0.499611 | 0.520563 | 0.536052 | 0.468546 |
| cg03225787 | 4.03E-04 | 1 | rs75775105;rs141594995 | 0.772808 | 0.793991 | 0.80211 | 0.747334 |
| cg25462807 | 4.02E-04 | 4 | rs187144854;rs554629189 | 0.761325 | 0.785959 | 0.785902 | 0.753024 |
| cg14453876 | 4.01E-04 | 7 |  | 0.77636 | 0.812362 | 0.807575 | 0.756344 |
| cg03541025 | 4.01E-04 | 7 | rs544027657;rs116117858; rs140722808;rs542663837; rs559330184 | 0.794698 | 0.819132 | 0.818505 | 0.763238 |
| cg09815885 | 4.00E-04 | 12 | rs142852759;rs192748518; rs184699309;rs114997980; rs548156587 | 0.773906 | 0.79441 | 0.799535 | 0.743159 |
| cg09232985 | 3.99E-04 | 20 | rs182189241;rs540428233 | 0.810324 | 0.836213 | 0.836583 | 0.80849 |
| cg22014687 | 3.99E-04 | 2 | rs188135209 | 0.781393 | 0.801905 | 0.810225 | 0.768879 |
| cg21911724 | 3.99E-04 | 8 | rs373554546;rs181031078; rs529547820 | 0.782652 | 0.803166 | 0.810238 | 0.772617 |
| cg18409436 | 3.97E-04 | 7 | rs118098604;rs183732759; rs551083048;rs189169368; rs115527731 | 0.765987 | 0.811884 | 0.807102 | 0.740972 |
| cg08169020 | 3.96E-04 | 14 | rs150284721 | 0.481854 | 0.50716 | 0.507741 | 0.389609 |
| cg05415936 | 3.94E-04 | 7 | rs542812173;rs533314876 | 0.772497 | 0.804753 | 0.793223 | 0.750318 |
| cg06229075 | 3.93E-04 | 7 |  | 0.692742 | 0.7376 | 0.74281 | 0.656302 |
| cg08678698 | 3.92E-04 | 6 | rs79948144;rs115630891 | 0.749868 | 0.775841 | 0.77967 | 0.714629 |
| cg04001802 | 3.91E-04 | 10 | rs181328799;rs184048312; rs571012329;rs539591096; rs553502059 | 0.817273 | 0.840232 | 0.845895 | 0.809982 |
| cg19948308 | 3.91E-04 | 2 | rs570392382 | 0.702084 | 0.732384 | 0.74371 | 0.668192 |
| cg10214132 | 3.90E-04 | 19 | rs181944943;rs560641603; rs376961960;rs550969977 | 0.789313 | 0.809592 | 0.816884 | 0.763033 |
| cg03134792 | 3.89E-04 | 13 | rs182993404 | 0.675792 | 0.697461 | 0.705061 | 0.65856 |
| cg07113062 | 3.88E-04 | 22 | rs567241331 | 0.77119 | 0.792763 | 0.808133 | 0.754364 |
| cg10781514 | 3.87E-04 | 8 | rs145461726;rs138054525; rs548313553;rs566619704 | 0.77557 | 0.80982 | 0.824002 | 0.772453 |
| cg14339466 | 3.87E-04 | 13 | rs527415449 | 0.694352 | 0.729373 | 0.732822 | 0.665392 |
| cg10797874 | 3.85E-04 | 1 | rs182721423;rs553724458 | 0.827295 | 0.848274 | 0.847621 | 0.814986 |
| cg10412258 | 3.85E-04 | 3 | rs144165398;rs563893080; rs115404280;rs146521018 | 0.800854 | 0.829934 | 0.832226 | 0.78413 |
| cg14595291 | 3.84E-04 | 11 | rs556381061;rs570175012 | 0.720852 | 0.742971 | 0.735247 | 0.689751 |
| cg04723513 | 3.83E-04 | 17 | rs182540563;rs548467935 | 0.600805 | 0.621631 | 0.622225 | 0.577846 |
| cg03472084 | 3.80E-04 | 15 | rs35571607;rs147438391; rs190747717 | 0.796212 | 0.820054 | 0.806599 | 0.787848 |
| cg09257456 | 3.78E-04 | 11 |  | 0.812853 | 0.83348 | 0.834285 | 0.799796 |
| cg09383432 | 3.78E-04 | 8 | rs533334605;rs552017479; rs79195151 | 0.748405 | 0.777551 | 0.774297 | 0.736407 |
| cg00868206 | 3.77E-04 | 6 | rs144570872;rs564405 | 0.750295 | 0.7717 | 0.774125 | 0.719136 |
| cg06940716 | 3.77E-04 | 18 |  | 0.788741 | 0.80947 | 0.815128 | 0.761272 |
| cg03505186 | 3.76E-04 | 3 | rs141502923;rs543480609; rs563254859;rs113334312 | 0.733238 | 0.768343 | 0.769321 | 0.723134 |
| cg00625436 | 3.75E-04 | 11 | rs552505722;rs566081729; rs137978817 | 0.768181 | 0.791105 | 0.802753 | 0.751674 |
| cg07816095 | 3.75E-04 | 16 | rs200019229;rs552116287 | 0.792471 | 0.812778 | 0.820374 | 0.834979 |
| cg06397732 | 3.73E-04 | 13 | rs142301476;rs73158445; rs544851081 | 0.746547 | 0.769952 | 0.776523 | 0.717714 |
| cg17854734 | 3.72E-04 | 10 | rs568257539;rs576263167 | 0.739803 | 0.761739 | 0.776257 | 0.743637 |
| cg26159063 | 3.72E-04 | 12 | rs145606758;rs185988740; rs542536780 | 0.641185 | 0.670232 | 0.675669 | 0.589316 |
| cg06726019 | 3.70E-04 | 10 | rs148078143;rs141832740 | 0.74294 | 0.769462 | 0.769332 | 0.710425 |
| cg23799246 | 3.67E-04 | 2 | rs146148019;rs574427071; rs74996855;rs540291267; rs148807713;rs72821908 | 0.54915 | 0.570388 | 0.577451 | 0.53044 |
| cg25920590 | 3.67E-04 | 8 |  | 0.466609 | 0.491987 | 0.492663 | 0.439403 |
| cg08156142 | 3.66E-04 | 5 | rs574744599;rs569792651 | 0.778636 | 0.803873 | 0.810213 | 0.770576 |
| cg02749714 | 3.61E-04 | 1 | rs530598393;rs190047852; rs181718753;rs536486107 | 0.787286 | 0.820748 | 0.817889 | 0.793407 |
| cg06398609 | 3.58E-04 | 10 | rs140443592 | 0.821613 | 0.841757 | 0.837378 | 0.815023 |
| cg16113883 | 3.58E-04 | 6 |  | 0.705635 | 0.734383 | 0.744044 | 0.673215 |
| cg09463055 | 3.53E-04 | 13 | rs552404259 | 0.702147 | 0.727798 | 0.721942 | 0.692171 |
| cg04892766 | 3.53E-04 | 3 | rs541244110 | 0.759468 | 0.779675 | 0.787953 | 0.733691 |
| cg08526894 | 3.53E-04 | 17 | rs193147927;rs569724917; rs58349074 | 0.780267 | 0.802507 | 0.786044 | 0.81011 |
| cg09050775 | 3.51E-04 | 12 | rs142686906 | 0.586472 | 0.607616 | 0.613143 | 0.552718 |
| cg03348631 | 3.50E-04 | 15 | rs181302868;rs202040979 | 0.80018 | 0.823058 | 0.827951 | 0.78597 |
| cg24196318 | 3.50E-04 | 21 | rs549655211;rs571499081; rs538928221;rs188517440 | 0.769951 | 0.794167 | 0.802825 | 0.740471 |
| cg26707728 | 3.50E-04 | 7 | rs138143481;rs575038042 | 0.773139 | 0.807271 | 0.79753 | 0.743758 |
| cg08315771 | 3.47E-04 | 4 | rs529738130;rs148531443; rs188321356 | 0.845968 | 0.873368 | 0.864762 | 0.830542 |
| cg06764656 | 3.44E-04 | 2 |  | 0.770101 | 0.793904 | 0.811236 | 0.742927 |
| cg05708683 | 3.43E-04 | 2 | rs79530332 | 0.792568 | 0.815234 | 0.818618 | 0.762941 |
| cg13246941 | 3.40E-04 | 3 | rs543921367 | 0.778881 | 0.803898 | 0.79833 | 0.733086 |
| cg23208285 | 3.40E-04 | 5 |  | 0.747697 | 0.790058 | 0.77582 | 0.727644 |
| cg23706319 | 3.39E-04 | 5 |  | 0.748684 | 0.779729 | 0.782288 | 0.738956 |
| cg04906747 | 3.37E-04 | 5 | rs556563132 | 0.831818 | 0.856723 | 0.857492 | 0.831868 |
| cg23149098 | 3.37E-04 | 7 | rs557919392;rs578067248 | 0.662545 | 0.683245 | 0.694741 | 0.613845 |
| cg07790947 | 3.35E-04 | 6 | rs542664043 | 0.643293 | 0.666389 | 0.670177 | 0.618411 |
| cg13978337 | 3.34E-04 | 6 | rs148827956;rs576606148 | 0.771498 | 0.792515 | 0.801024 | 0.756075 |
| cg08560458 | 3.33E-04 | 19 | rs76810560;rs193118795; rs76318120;rs375421667 | 0.762397 | 0.785952 | 0.789298 | 0.726178 |
| cg05235104 | 3.31E-04 | 8 | rs527909099 | 0.646163 | 0.673168 | 0.688038 | 0.618704 |
| cg14758944 | 3.31E-04 | 5 | rs146479544;rs186386867 | 0.672526 | 0.697891 | 0.710003 | 0.646972 |
| cg04905274 | 3.31E-04 | 12 | rs527264370 | 0.797348 | 0.820458 | 0.810486 | 0.84502 |
| cg02641917 | 3.31E-04 | 2 | rs116195807;rs571723621 | 0.80441 | 0.825449 | 0.838735 | 0.797344 |
| cg13378625 | 3.30E-04 | 19 | rs148298161;rs185506627 | 0.671617 | 0.695846 | 0.70553 | 0.646787 |
| cg01658895 | 3.30E-04 | 5 | rs542787908 | 0.71711 | 0.760944 | 0.764694 | 0.683079 |
| cg02209488 | 3.30E-04 | 4 | rs4541559;rs565899007; rs145983593 | 0.755978 | 0.785058 | 0.794223 | 0.73756 |
| cg26335865 | 3.29E-04 | 10 | rs188749290 | 0.587072 | 0.618037 | 0.61484 | 0.569177 |
| cg11044270 | 3.28E-04 | 12 | rs73131048;rs142982926; rs118027344 | 0.64446 | 0.687345 | 0.688173 | 0.644401 |
| cg09845310 | 3.28E-04 | 3 | rs545842454 | 0.81245 | 0.834166 | 0.843591 | 0.805438 |
| cg02895724 | 3.27E-04 | 18 |  | 0.691814 | 0.725209 | 0.732535 | 0.68471 |
| cg03805787 | 3.26E-04 | 17 | rs556280869 | 0.625213 | 0.660208 | 0.675766 | 0.58967 |
| cg01748970 | 3.23E-04 | 14 | rs183481399;rs2255959 | 0.743966 | 0.77424 | 0.769278 | 0.715727 |
| cg10443044 | 3.22E-04 | 12 |  | 0.717907 | 0.743116 | 0.747967 | 0.680024 |
| cg10537253 | 3.20E-04 | 21 | rs577655262;rs150853670; rs187609246 | 0.678513 | 0.706808 | 0.72035 | 0.660972 |
| cg09701035 | 3.18E-04 | 4 | rs146631228 | 0.722961 | 0.751456 | 0.760679 | 0.713688 |
| cg07393778 | 3.18E-04 | 4 | rs116569303 | 0.765682 | 0.786979 | 0.790875 | 0.760352 |
| cg17209404 | 3.17E-04 | 10 | rs546060015;rs113947651 | 0.790466 | 0.816377 | 0.807949 | 0.770593 |
| cg09181738 | 3.17E-04 | 21 | rs146747087;rs76153162; rs548987913;rs73315657 | 0.806273 | 0.827218 | 0.831285 | 0.791164 |
| cg08631134 | 3.16E-04 | 9 | rs114116330 | 0.660213 | 0.687619 | 0.702522 | 0.636862 |
| cg11545402 | 3.15E-04 | 14 | rs112816492 | 0.70233 | 0.725599 | 0.741576 | 0.662936 |
| cg06206888 | 3.15E-04 | 2 | rs191364208;rs78822378; rs572174177 | 0.803862 | 0.826131 | 0.830745 | 0.79319 |
| cg26945107 | 3.12E-04 | 1 | rs536221520 | 0.711649 | 0.73919 | 0.755684 | 0.702536 |
| cg07823284 | 3.11E-04 | 19 | rs567038350;rs539365191; rs143591143 | 0.795849 | 0.82467 | 0.808759 | 0.769056 |
| cg04557631 | 3.10E-04 | 3 | rs538720038 | 0.779863 | 0.800295 | 0.79161 | 0.773543 |
| cg26183708 | 3.10E-04 | 6 | rs553333003;rs140567533 | 0.835572 | 0.862308 | 0.845495 | 0.804467 |
| cg21168034 | 3.10E-04 | 13 | rs539061960 | 0.758242 | 0.789154 | 0.79972 | 0.752487 |
| cg24791971 | 3.09E-04 | 3 | rs535728984 | 0.69158 | 0.714269 | 0.71621 | 0.685295 |
| cg25038311 | 3.09E-04 | 10 | rs143955195 | 0.765026 | 0.796389 | 0.795708 | 0.734215 |
| cg15257322 | 3.07E-04 | 7 | rs539229025 | 0.736164 | 0.771419 | 0.770502 | 0.693596 |
| cg17449964 | 3.05E-04 | 7 | rs3808106;rs180867272; rs183584856;rs189498172; rs560810139 | 0.742678 | 0.767622 | 0.757747 | 0.725506 |
| cg14328090 | 3.05E-04 | 12 |  | 0.812328 | 0.843757 | 0.832581 | 0.811709 |
| cg17381661 | 3.05E-04 | 4 | rs143203083 | 0.744916 | 0.765808 | 0.781239 | 0.723198 |
| cg03482710 | 3.04E-04 | 1 | rs573548597 | 0.771459 | 0.802192 | 0.810238 | 0.74758 |
| cg08798862 | 3.04E-04 | 16 | rs573769086;rs541030229; rs146564310;rs192832707 | 0.712504 | 0.737188 | 0.739584 | 0.683246 |
| cg07111500 | 3.04E-04 | 5 | rs576606584;rs2650940; rs185757098 | 0.772473 | 0.794439 | 0.788601 | 0.742029 |
| cg15165735 | 3.03E-04 | 3 | rs373072820 | 0.753539 | 0.782086 | 0.77641 | 0.727749 |
| cg14642709 | 3.00E-04 | 17 | rs568562125 | 0.793632 | 0.815164 | 0.820457 | 0.749134 |
| cg08156015 | 3.00E-04 | 7 | rs554988123;rs567138520 | 0.81397 | 0.838275 | 0.835395 | 0.811421 |
| cg03004374 | 3.00E-04 | 3 | rs114488648;rs573224836 | 0.729083 | 0.763812 | 0.782099 | 0.71589 |
| cg10798853 | 2.99E-04 | 18 |  | 0.769813 | 0.789975 | 0.800377 | 0.755768 |
| cg24341513 | 2.98E-04 | 1 | rs545728574 | 0.797784 | 0.821192 | 0.833652 | 0.791071 |
| cg13449535 | 2.98E-04 | 1 | rs80296038;rs531459645 | 0.789817 | 0.825047 | 0.826059 | 0.784499 |
| cg02677189 | 2.96E-04 | 8 | rs183393227 | 0.783533 | 0.808831 | 0.804024 | 0.769578 |
| cg07972624 | 2.96E-04 | 7 | rs545997767 | 0.743187 | 0.771247 | 0.766796 | 0.721612 |
| cg03681905 | 2.96E-04 | 4 |  | 0.778702 | 0.802613 | 0.811357 | 0.753108 |
| cg24111025 | 2.95E-04 | 6 | rs555493390;rs140245535 | 0.772131 | 0.80079 | 0.796883 | 0.738987 |
| cg06994787 | 2.95E-04 | 16 | rs187276466;rs191679415; rs567290136;rs117032079; rs546985996 | 0.739351 | 0.762089 | 0.754755 | 0.727303 |
| cg12214701 | 2.95E-04 | 10 |  | 0.745585 | 0.783518 | 0.791691 | 0.716261 |
| cg06696684 | 2.94E-04 | 7 | rs570230081;rs78302322 | 0.81818 | 0.84225 | 0.850215 | 0.814324 |
| cg18700420 | 2.92E-04 | 5 | rs116338481;rs144182041 | 0.749533 | 0.783599 | 0.776867 | 0.724786 |
| cg02576260 | 2.87E-04 | 1 |  | 0.802334 | 0.823341 | 0.835283 | 0.792862 |
| cg14544831 | 2.86E-04 | 18 | rs543688353 | 0.449689 | 0.471875 | 0.474442 | 0.426904 |
| cg00110666 | 2.84E-04 | 8 | rs138299728 | 0.742688 | 0.765972 | 0.776622 | 0.701706 |
| cg26590823 | 2.82E-04 | 20 | rs533314189 | 0.721461 | 0.753917 | 0.753553 | 0.705137 |
| cg04259892 | 2.81E-04 | 8 | rs10956470;rs142541331; rs535779887 | 0.703994 | 0.7387 | 0.729601 | 0.657074 |
| cg08960658 | 2.81E-04 | 6 | rs556319580;rs182530331 | 0.73732 | 0.759377 | 0.771332 | 0.712294 |
| cg02250646 | 2.81E-04 | 15 | rs138131548;rs149227500 | 0.730777 | 0.758618 | 0.748948 | 0.705055 |
| cg16562296 | 2.79E-04 | 2 | rs181688298;rs541110109 | 0.661935 | 0.683901 | 0.702563 | 0.622316 |
| cg02908509 | 2.79E-04 | 2 | rs560498987 | 0.739356 | 0.765149 | 0.771044 | 0.73496 |
| cg01204100 | 2.77E-04 | 19 | rs73043120;rs150314415; rs79447456 | 0.736126 | 0.763261 | 0.769806 | 0.721952 |
| cg22892718 | 2.76E-04 | 6 | rs528578473;rs546660101; rs184606857;rs532498241 | 0.773545 | 0.794929 | 0.804691 | 0.765899 |
| cg22755399 | 2.74E-04 | 12 | rs182558678;rs142936190; rs151113017 | 0.591523 | 0.61504 | 0.625766 | 0.555395 |
| cg19405124 | 2.74E-04 | 17 | rs200866660 | 0.552745 | 0.573381 | 0.585293 | 0.637569 |
| cg10451262 | 2.73E-04 | 8 | rs117507592;rs150011853 | 0.818197 | 0.839678 | 0.839365 | 0.812688 |
| cg12728309 | 2.71E-04 | 5 | rs572033432 | 0.847838 | 0.872155 | 0.871099 | 0.860859 |
| cg00896282 | 2.70E-04 | 7 | rs184475181;rs562344335; rs529401351;rs143527406 | 0.790124 | 0.812359 | 0.816062 | 0.765142 |
| cg12774189 | 2.69E-04 | 1 | rs112437910;rs570694893 | 0.651046 | 0.683163 | 0.687921 | 0.646053 |
| cg21570108 | 2.69E-04 | 1 | rs140713566;rs143218531; rs563980851 | 0.762611 | 0.794778 | 0.792809 | 0.746858 |
| cg24754439 | 2.67E-04 | 13 | rs570536775 | 0.684748 | 0.706988 | 0.703721 | 0.680625 |
| cg06951580 | 2.67E-04 | 5 | rs6896628 | 0.783198 | 0.806368 | 0.7977 | 0.746785 |
| cg17756730 | 2.66E-04 | 1 |  | 0.624143 | 0.650363 | 0.65744 | 0.581405 |
| cg22494876 | 2.66E-04 | 1 |  | 0.785316 | 0.816338 | 0.812049 | 0.765942 |
| cg24999823 | 2.64E-04 | 15 | rs568186485;rs533751939; rs553815837 | 0.629059 | 0.653923 | 0.661469 | 0.604652 |
| cg06571552 | 2.63E-04 | 13 |  | 0.625098 | 0.648892 | 0.655537 | 0.586735 |
| cg07136619 | 2.61E-04 | 2 | rs147276279 | 0.63602 | 0.65809 | 0.665099 | 0.617099 |
| cg15811964 | 2.61E-04 | 12 | rs138814410 | 0.577629 | 0.601269 | 0.614443 | 0.540897 |
| cg01284698 | 2.60E-04 | 2 |  | 0.701607 | 0.727467 | 0.716853 | 0.68783 |
| cg25441252 | 2.60E-04 | 6 |  | 0.796379 | 0.818231 | 0.823415 | 0.76285 |
| cg06180822 | 2.59E-04 | 8 | rs145956874;rs538854482 | 0.506703 | 0.527334 | 0.529728 | 0.496954 |
| cg22482473 | 2.58E-04 | 12 | rs142168748;rs550378043 | 0.665549 | 0.685656 | 0.698066 | 0.641165 |
| cg23445947 | 2.58E-04 | 9 | rs530983222;rs142377727 | 0.734408 | 0.761477 | 0.766734 | 0.729621 |
| cg02648014 | 2.55E-04 | 5 | rs114546562;rs189516417 | 0.747803 | 0.777531 | 0.793185 | 0.737943 |
| cg05022368 | 2.54E-04 | 8 |  | 0.784289 | 0.805412 | 0.812973 | 0.768623 |
| cg15548797 | 2.53E-04 | 18 |  | 0.731436 | 0.755542 | 0.757492 | 0.706538 |
| cg26088711 | 2.52E-04 | 12 | rs115379634;rs542241840 | 0.703091 | 0.725529 | 0.728571 | 0.688944 |
| cg01526578 | 2.52E-04 | 7 | rs539326673;rs10276142; rs564078653 | 0.752588 | 0.784198 | 0.782824 | 0.721765 |
| cg17747450 | 2.52E-04 | 10 | rs550678728;rs180754722 | 0.786906 | 0.812654 | 0.818922 | 0.791562 |
| cg16371214 | 2.48E-04 | 5 | rs148607960;rs576957288 | 0.79217 | 0.817154 | 0.823041 | 0.775347 |
| cg12966876 | 2.48E-04 | 2 | rs577113400 | 0.672724 | 0.699387 | 0.692792 | 0.644598 |
| cg16014491 | 2.47E-04 | 9 | rs550147702 | 0.783459 | 0.80685 | 0.802019 | 0.774716 |
| cg09069435 | 2.47E-04 | 5 | rs531895688;rs541963229 | 0.724948 | 0.755188 | 0.751798 | 0.662775 |
| cg18611065 | 2.47E-04 | 3 | rs145125070 | 0.766255 | 0.797228 | 0.795328 | 0.742754 |
| cg15737401 | 2.45E-04 | 13 | rs191545542;rs535381221; rs115837403 | 0.683093 | 0.720965 | 0.732214 | 0.675202 |
| cg19719207 | 2.44E-04 | 4 |  | 0.792481 | 0.816244 | 0.815655 | 0.795419 |
| cg24639703 | 2.43E-04 | 1 | rs529837449 | 0.665641 | 0.696257 | 0.704769 | 0.648458 |
| cg13233968 | 2.43E-04 | 1 |  | 0.779662 | 0.803758 | 0.81647 | 0.768853 |
| cg01725917 | 2.42E-04 | 18 | rs142406597 | 0.816478 | 0.840163 | 0.835032 | 0.822231 |
| cg15571933 | 2.42E-04 | 22 | rs9618506;rs565497053 | 0.711126 | 0.732912 | 0.740743 | 0.692251 |
| cg15509764 | 2.42E-04 | 3 | rs577357804;rs545626786; rs114562075 | 0.699794 | 0.752399 | 0.757707 | 0.674273 |
| cg21273772 | 2.41E-04 | 11 | rs587659337 | 0.733215 | 0.754386 | 0.768097 | 0.717531 |
| cg09939446 | 2.41E-04 | 10 | rs190985422;rs570366824; rs12253822 | 0.809385 | 0.838557 | 0.847009 | 0.789796 |
| cg07830156 | 2.39E-04 | 1 | rs139441759 | 0.722856 | 0.753651 | 0.754608 | 0.71236 |
| cg11745672 | 2.37E-04 | 10 |  | 0.693862 | 0.720638 | 0.722897 | 0.660334 |
| cg08146256 | 2.36E-04 | 14 | rs571772111;rs540834348 | 0.746368 | 0.78286 | 0.772185 | 0.691986 |
| cg06238667 | 2.35E-04 | 8 | rs529364077;rs549214812 | 0.665291 | 0.710143 | 0.704546 | 0.634256 |
| cg14276730 | 2.34E-04 | 20 | rs6102408 | 0.721754 | 0.750233 | 0.74934 | 0.713825 |
| cg14094409 | 2.32E-04 | 12 | rs573968192;rs78028061 | 0.716193 | 0.743695 | 0.761014 | 0.681722 |
| cg02250431 | 2.30E-04 | 5 | rs555056054 | 0.627745 | 0.65779 | 0.647906 | 0.706237 |
| cg25248319 | 2.27E-04 | 5 |  | 0.764859 | 0.79641 | 0.793108 | 0.761573 |
| cg01826492 | 2.27E-04 | 13 |  | 0.77165 | 0.800475 | 0.794158 | 0.757153 |
| cg08750473 | 2.25E-04 | 19 | rs576852127 | 0.767557 | 0.793476 | 0.798213 | 0.755103 |
| cg01659072 | 2.24E-04 | 4 | rs192096755;rs183038490 | 0.533985 | 0.560821 | 0.574881 | 0.519568 |
| cg00576494 | 2.24E-04 | 6 |  | 0.761915 | 0.790127 | 0.779863 | 0.75269 |
| cg14479377 | 2.23E-04 | 9 | rs115460772;rs571935762 | 0.633489 | 0.655823 | 0.674207 | 0.610481 |
| cg00889210 | 2.22E-04 | 16 | rs143898973 | 0.452099 | 0.473094 | 0.481175 | 0.436278 |
| cg26304979 | 2.22E-04 | 4 | rs573724156;rs541234798 | 0.757123 | 0.777712 | 0.792572 | 0.740236 |
| cg10956811 | 2.20E-04 | 1 | rs114330083 | 0.823292 | 0.844497 | 0.846639 | 0.826767 |
| cg25806677 | 2.19E-04 | 2 | rs114339931;rs544068321; rs555786370;rs140657793; rs541254240;rs559564781 | 0.67752 | 0.707977 | 0.707417 | 0.669947 |
| cg21476731 | 2.17E-04 | 8 | rs540732483;rs560509838; rs529286459;rs4986988; rs563016062 | 0.761486 | 0.7968 | 0.792297 | 0.746416 |
| cg26967115 | 2.17E-04 | 4 | rs113864857 | 0.798907 | 0.825217 | 0.817465 | 0.79387 |
| cg14069921 | 2.16E-04 | 14 | rs113725666;rs184835074 | 0.783202 | 0.809825 | 0.811181 | 0.769421 |
| cg20043176 | 2.15E-04 | 12 | rs536191722 | 0.640682 | 0.673131 | 0.669858 | 0.603856 |
| cg01262564 | 2.14E-04 | 9 | rs527492061;rs552487280 | 0.469895 | 0.499905 | 0.51579 | 0.463058 |
| cg25426982 | 2.13E-04 | 18 | rs151170101;rs140237039 | 0.488021 | 0.508478 | 0.500919 | 0.482286 |
| cg00848007 | 2.13E-04 | 12 | rs181407537;rs569749256; rs537209084 | 0.758176 | 0.784593 | 0.801947 | 0.744279 |
| cg26528744 | 2.13E-04 | 10 | rs534995442;rs553238191 | 0.780504 | 0.80285 | 0.803874 | 0.754128 |
| cg10359823 | 2.12E-04 | 17 | rs141500093;rs563618389; rs147037677 | 0.71915 | 0.742645 | 0.749248 | 0.705508 |
| cg22520850 | 2.10E-04 | 12 | rs192959388;rs114722682; rs548074444 | 0.216807 | 0.240526 | 0.236792 | 0.212139 |
| cg17355820 | 2.09E-04 | 14 | rs548222525;rs574971662; rs533929491;rs193282334 | 0.725671 | 0.751522 | 0.76777 | 0.708471 |
| cg18396789 | 2.08E-04 | 7 |  | 0.788746 | 0.817844 | 0.803688 | 0.771949 |
| cg02292788 | 2.08E-04 | 2 | rs73976197;rs187142860; rs562863674 | 0.720766 | 0.742847 | 0.760766 | 0.678674 |
| cg02889110 | 2.07E-04 | 5 |  | 0.790631 | 0.816898 | 0.818934 | 0.788122 |
| cg06381319 | 2.06E-04 | 6 | rs566499248;rs538225404 | 0.7044 | 0.736766 | 0.737655 | 0.64419 |
| cg01800986 | 2.03E-04 | 13 | rs565731105;rs534716839 | 0.816857 | 0.837543 | 0.841405 | 0.812447 |
| cg11906293 | 2.02E-04 | 18 | rs77707603;rs580504 | 0.763484 | 0.788754 | 0.791607 | 0.725438 |
| cg04911180 | 2.01E-04 | 3 | rs141136876;rs578078572; rs543794774;rs562794807 | 0.700416 | 0.726873 | 0.727404 | 0.666955 |
| cg19632186 | 1.99E-04 | 17 | rs577442955 | 0.791739 | 0.817822 | 0.814667 | 0.778387 |
| cg18759066 | 1.99E-04 | 12 | rs577651384;rs141747075; rs560088482 | 0.528411 | 0.551699 | 0.558808 | 0.526849 |
| cg04994855 | 1.99E-04 | 12 |  | 0.783798 | 0.804861 | 0.811408 | 0.765516 |
| cg16059068 | 1.98E-04 | 8 | rs569596548;rs6988101; rs555116648 | 0.601948 | 0.625742 | 0.626966 | 0.596429 |
| cg03135964 | 1.96E-04 | 12 | rs550303147;rs117788818 | 0.67874 | 0.709243 | 0.704639 | 0.635011 |
| cg12573049 | 1.95E-04 | 14 | rs186394481 | 0.719893 | 0.74025 | 0.75754 | 0.671444 |
| cg02792136 | 1.94E-04 | 10 | rs535262376;rs74980157 | 0.717753 | 0.745617 | 0.752147 | 0.687447 |
| cg16661042 | 1.94E-04 | 15 | rs149808233;rs532749490; rs146810137 | 0.772546 | 0.801507 | 0.790934 | 0.764758 |
| cg20566439 | 1.90E-04 | 7 | rs573791030 | 0.8255 | 0.847505 | 0.846248 | 0.797471 |
| cg15913931 | 1.89E-04 | 8 | rs540461414 | 0.800717 | 0.822317 | 0.825137 | 0.771896 |
| cg25594356 | 1.89E-04 | 8 | rs55824165;rs113143495 | 0.76205 | 0.78683 | 0.787143 | 0.732436 |
| cg04267686 | 1.89E-04 | 6 | rs186641350;rs56393247 | 0.817397 | 0.839776 | 0.845961 | 0.785809 |
| cg18154404 | 1.87E-04 | 7 | rs548095017;rs571203865; rs145524267 | 0.807918 | 0.837463 | 0.837491 | 0.808972 |
| cg01109380 | 1.86E-04 | 21 | rs181888456 | 0.720326 | 0.758172 | 0.770648 | 0.690587 |
| cg19818294 | 1.86E-04 | 9 | rs184039398;rs189452866 | 0.775813 | 0.801253 | 0.796158 | 0.751278 |
| cg09718063 | 1.85E-04 | 16 | rs181674443;rs557002143; rs186828557 | 0.787553 | 0.807994 | 0.816824 | 0.779551 |
| cg01925695 | 1.83E-04 | 7 |  | 0.778076 | 0.800645 | 0.805871 | 0.756192 |
| cg16133683 | 1.83E-04 | 9 | rs567523208 | 0.787563 | 0.809549 | 0.801328 | 0.748631 |
| cg01957599 | 1.83E-04 | 4 |  | 0.766111 | 0.788477 | 0.799395 | 0.73754 |
| cg10850293 | 1.82E-04 | 15 | rs546426659;rs564617388 | 0.77355 | 0.794163 | 0.789199 | 0.742395 |
| cg12505889 | 1.81E-04 | 2 | rs75069209 | 0.793313 | 0.829942 | 0.835144 | 0.810549 |
| cg26382697 | 1.81E-04 | 11 | rs538183424 | 0.689241 | 0.719404 | 0.729775 | 0.672831 |
| cg18783424 | 1.80E-04 | 15 | rs537356295 | 0.811143 | 0.832983 | 0.839867 | 0.854563 |
| cg24198209 | 1.78E-04 | 13 | rs526506;rs558113046 | 0.766322 | 0.797505 | 0.809233 | 0.755692 |
| cg15673861 | 1.77E-04 | 11 | rs568987058;rs193205047 | 0.653026 | 0.678012 | 0.683904 | 0.636746 |
| cg06633990 | 1.77E-04 | 2 |  | 0.539953 | 0.580761 | 0.581105 | 0.518227 |
| cg06872172 | 1.76E-04 | 1 | rs138749020;rs190240107; rs367564949;rs142742644 | 0.792799 | 0.815776 | 0.816049 | 0.779634 |
| cg02043791 | 1.75E-04 | 8 | rs552179661;rs72630656 | 0.614084 | 0.634205 | 0.645254 | 0.694159 |
| cg14966288 | 1.74E-04 | 21 | rs114784794;rs544691473 | 0.755281 | 0.788176 | 0.790869 | 0.74622 |
| cg14112356 | 1.73E-04 | 21 | rs373180970;rs528295707 | 0.548293 | 0.569014 | 0.581371 | 0.506709 |
| cg02948188 | 1.73E-04 | 10 | rs576228937 | 0.743613 | 0.77786 | 0.782682 | 0.73186 |
| cg00080483 | 1.72E-04 | 6 |  | 0.274596 | 0.300176 | 0.285981 | 0.299744 |
| cg19568380 | 1.71E-04 | 4 | rs547365165 | 0.79236 | 0.813006 | 0.811183 | 0.750534 |
| cg02484634 | 1.70E-04 | 7 | rs114628365 | 0.76395 | 0.804321 | 0.808637 | 0.73562 |
| cg10174359 | 1.70E-04 | 7 |  | 0.753279 | 0.773879 | 0.787531 | 0.743971 |
| cg16440429 | 1.69E-04 | 3 | rs9852490;rs537203418 | 0.718021 | 0.749418 | 0.741536 | 0.697547 |
| cg26202142 | 1.69E-04 | 17 | rs539504397;rs557908159 | 0.3417 | 0.374612 | 0.365619 | 0.356909 |
| cg25963419 | 1.69E-04 | 2 | rs4851526;rs72990754 | 0.700744 | 0.727587 | 0.737681 | 0.664109 |
| cg08719237 | 1.68E-04 | 1 | rs558435329;rs75782254; rs534746140 | 0.75123 | 0.791981 | 0.785623 | 0.735445 |
| cg19743778 | 1.68E-04 | 8 |  | 0.692217 | 0.712815 | 0.7294 | 0.649782 |
| cg10679542 | 1.68E-04 | 1 | rs560457562 | 0.766601 | 0.795798 | 0.797219 | 0.74743 |
| cg07845510 | 1.67E-04 | 8 | rs577408652 | 0.824542 | 0.853419 | 0.854935 | 0.819492 |
| cg11616262 | 1.66E-04 | 2 | rs537336641 | 0.660583 | 0.70912 | 0.721645 | 0.662899 |
| cg05841987 | 1.66E-04 | 20 | rs146763060 | 0.82399 | 0.845338 | 0.844188 | 0.820369 |
| cg02101403 | 1.66E-04 | 7 |  | 0.784446 | 0.810078 | 0.828653 | 0.811356 |
| cg26131463 | 1.66E-04 | 13 | rs544606448 | 0.756908 | 0.79573 | 0.786994 | 0.741031 |
| cg04894443 | 1.66E-04 | 10 | rs374145869;rs561113688; rs529902509;rs546774512 | 0.745001 | 0.784567 | 0.784812 | 0.698984 |
| cg23307893 | 1.66E-04 | 8 | rs573307497;rs540358307; rs78142429;rs529510008; rs184757603 | 0.640917 | 0.671728 | 0.668125 | 0.613674 |
| cg09957895 | 1.65E-04 | 3 |  | 0.790099 | 0.816743 | 0.820354 | 0.759198 |
| cg23390269 | 1.65E-04 | 19 | rs140364316 | 0.839729 | 0.85999 | 0.864184 | 0.840288 |
| cg12277366 | 1.64E-04 | 8 | rs191518259 | 0.644488 | 0.666988 | 0.672565 | 0.617706 |
| cg02330172 | 1.63E-04 | 2 |  | 0.677857 | 0.70458 | 0.717779 | 0.618822 |
| cg17904105 | 1.63E-04 | 22 | rs73394890;rs562006468; rs111587642;rs554163645 | 0.803373 | 0.828845 | 0.823189 | 0.800904 |
| cg02060515 | 1.62E-04 | 6 | rs545649311 | 0.70114 | 0.733635 | 0.744428 | 0.659175 |
| cg26613271 | 1.62E-04 | 8 | rs147248159;rs16875900; rs531674565 | 0.73293 | 0.771625 | 0.776977 | 0.738094 |
| cg15479589 | 1.61E-04 | 8 | rs369803616;rs573021942; rs11779660 | 0.754443 | 0.786976 | 0.776756 | 0.740325 |
| cg03204155 | 1.61E-04 | 22 |  | 0.633633 | 0.672483 | 0.670253 | 0.582209 |
| cg11304291 | 1.60E-04 | 15 | rs183887051;rs187599681 | 0.819483 | 0.840198 | 0.83558 | 0.801426 |
| cg07109649 | 1.58E-04 | 4 | rs115747130;rs186743412; rs548140157 | 0.806962 | 0.830859 | 0.829708 | 0.855594 |
| cg24229206 | 1.54E-04 | 10 | rs539603679;rs12098246; rs111418873 | 0.739337 | 0.767194 | 0.763275 | 0.707426 |
| cg13714407 | 1.53E-04 | 9 | rs547492306;rs182306309 | 0.767343 | 0.792965 | 0.805902 | 0.735855 |
| cg17804154 | 1.52E-04 | 11 |  | 0.72025 | 0.762214 | 0.766589 | 0.716017 |
| cg17084900 | 1.50E-04 | 13 |  | 0.651837 | 0.674816 | 0.682644 | 0.619794 |
| cg01294526 | 1.50E-04 | 5 | rs61757417;rs191539268; rs551279605;rs183782460; rs188719907 | 0.789288 | 0.810758 | 0.815847 | 0.762961 |
| cg03444838 | 1.48E-04 | 10 | rs141494514;rs150872377 | 0.727271 | 0.754522 | 0.753645 | 0.692289 |
| cg02881547 | 1.48E-04 | 2 | rs34857485 | 0.843796 | 0.870628 | 0.867609 | 0.851962 |
| cg00980904 | 1.46E-04 | 3 |  | 0.795215 | 0.82141 | 0.815233 | 0.795748 |
| cg10126552 | 1.46E-04 | 13 | rs541201336;rs552467317 | 0.783677 | 0.823093 | 0.813018 | 0.752328 |
| cg17013606 | 1.46E-04 | 15 | rs542312470;rs560380865 | 0.747092 | 0.777379 | 0.791134 | 0.752038 |
| cg24606065 | 1.46E-04 | 17 | rs566640439;rs535915495; rs185611781;rs73988204; rs9900489 | 0.704297 | 0.738167 | 0.749791 | 0.698946 |
| cg08705882 | 1.45E-04 | 10 | rs534864034 | 0.838843 | 0.865752 | 0.868196 | 0.83768 |
| cg02088086 | 1.45E-04 | 20 | rs114915637;rs538865116 | 0.712981 | 0.734212 | 0.734868 | 0.670333 |
| cg20509001 | 1.43E-04 | 9 | rs575098913;rs367592109; rs72741097 | 0.499661 | 0.531827 | 0.531179 | 0.472565 |
| cg15248592 | 1.41E-04 | 14 | rs568294510;rs79916637 | 0.747433 | 0.788195 | 0.778858 | 0.739314 |
| cg03942839 | 1.40E-04 | 5 | rs142460836 | 0.769143 | 0.79992 | 0.791621 | 0.738564 |
| cg13480043 | 1.40E-04 | 3 |  | 0.691672 | 0.730407 | 0.735369 | 0.646848 |
| cg10808874 | 1.39E-04 | 14 | rs1677993;rs367846067 | 0.788292 | 0.811752 | 0.814382 | 0.764573 |
| cg15029037 | 1.38E-04 | 4 | rs554070523;rs573988114; rs536599551 | 0.774234 | 0.802227 | 0.809906 | 0.74914 |
| cg08916862 | 1.35E-04 | 16 | rs294266;rs566598385; rs535652971 | 0.74704 | 0.77925 | 0.781608 | 0.744994 |
| cg21947565 | 1.33E-04 | 20 | rs367959565 | 0.797359 | 0.823082 | 0.818142 | 0.777102 |
| cg00757822 | 1.31E-04 | 3 | rs575642451 | 0.784048 | 0.80468 | 0.803152 | 0.754541 |
| cg25434203 | 1.29E-04 | 20 | rs568759973;rs532965438 | 0.801332 | 0.830683 | 0.817507 | 0.798181 |
| cg08474396 | 1.29E-04 | 16 |  | 0.780425 | 0.811736 | 0.808127 | 0.759687 |
| cg08945795 | 1.28E-04 | 13 | rs544754528 | 0.736746 | 0.763072 | 0.765822 | 0.705232 |
| cg14153437 | 1.26E-04 | 5 | rs76106112;rs537729880 | 0.758101 | 0.783452 | 0.776254 | 0.711652 |
| cg22209302 | 1.26E-04 | 2 | rs547071745;rs6752068 | 0.760965 | 0.794115 | 0.800752 | 0.744614 |
| cg13057898 | 1.24E-04 | 1 | rs200838010;rs550960745; rs150301384;rs200988380 | 0.819503 | 0.852718 | 0.847628 | 0.795958 |
| cg25491321 | 1.24E-04 | 5 | rs547198662;rs565638071; rs539098329 | 0.719277 | 0.74443 | 0.751389 | 0.697877 |
| cg24169332 | 1.24E-04 | 11 | rs73438922;rs375252314; rs138679299;rs369781099; rs112436013 | 0.750219 | 0.783557 | 0.774021 | 0.728554 |
| cg07110142 | 1.24E-04 | 5 | rs577652768 | 0.76894 | 0.80202 | 0.798076 | 0.733245 |
| cg05447290 | 1.24E-04 | 5 |  | 0.7693 | 0.804418 | 0.796663 | 0.749114 |
| cg17533118 | 1.24E-04 | 2 | rs115314350;rs554544719; rs138966646;rs555071664; rs564661079 | 0.719329 | 0.755342 | 0.751646 | 0.695388 |
| cg24416197 | 1.23E-04 | 1 | rs200038116;rs200568289 | 0.610004 | 0.645717 | 0.664632 | 0.606815 |
| cg18661884 | 1.23E-04 | 4 | rs540682982;rs564936773 | 0.748535 | 0.782328 | 0.782423 | 0.699407 |
| cg01482790 | 1.22E-04 | 19 | rs11666117 | 0.538167 | 0.566395 | 0.570039 | 0.527622 |
| cg05036909 | 1.22E-04 | 5 | rs535473414 | 0.778896 | 0.80859 | 0.818726 | 0.765007 |
| cg08008702 | 1.22E-04 | 15 |  | 0.77433 | 0.803366 | 0.790833 | 0.74648 |
| cg05215884 | 1.21E-04 | 15 | rs528128415;rs541466868; rs561579557 | 0.792174 | 0.824978 | 0.82795 | 0.78795 |
| cg14640661 | 1.21E-04 | 1 | rs576330809 | 0.817323 | 0.844606 | 0.841363 | 0.802098 |
| cg01154353 | 1.21E-04 | 13 | rs533110533;rs368974812; rs369317979;rs577938242 | 0.771585 | 0.795762 | 0.789734 | 0.753885 |
| cg25882090 | 1.21E-04 | 20 | rs572600011;rs541704161 | 0.800926 | 0.829117 | 0.826943 | 0.788656 |
| cg00826632 | 1.20E-04 | 19 | rs539211626;rs552261624 | 0.754129 | 0.777911 | 0.788571 | 0.725435 |
| cg09111892 | 1.19E-04 | 5 | rs538418835;rs550465169 | 0.732266 | 0.757405 | 0.766439 | 0.695869 |
| cg26781797 | 1.19E-04 | 2 | rs552353332 | 0.795203 | 0.820892 | 0.808594 | 0.774077 |
| cg22883426 | 1.17E-04 | 14 | rs551419670;rs57519209; rs536780333 | 0.715469 | 0.738314 | 0.743492 | 0.671025 |
| cg14010395 | 1.17E-04 | 1 |  | 0.45939 | 0.48626 | 0.474326 | 0.468894 |
| cg02253134 | 1.17E-04 | 5 | rs369314680;rs190252656 | 0.67074 | 0.704557 | 0.704249 | 0.632093 |
| cg00010672 | 1.14E-04 | 2 | rs141247431;rs150857639 | 0.73388 | 0.772617 | 0.776217 | 0.691587 |
| cg07115924 | 1.14E-04 | 7 | rs571336876;rs538329320 | 0.799495 | 0.828718 | 0.822145 | 0.786337 |
| cg12428334 | 1.14E-04 | 14 | rs150915065;rs111473472 | 0.832984 | 0.853048 | 0.853356 | 0.798742 |
| cg06649682 | 1.14E-04 | 8 | rs571365902;rs537339073; rs557004817 | 0.81198 | 0.846118 | 0.840292 | 0.81719 |
| cg02907236 | 1.08E-04 | 1 | rs550190489 | 0.740882 | 0.765147 | 0.773884 | 0.714991 |
| cg08017911 | 1.07E-04 | 4 |  | 0.792896 | 0.81857 | 0.809239 | 0.752434 |
| cg22916901 | 1.07E-04 | 21 | rs150566511;rs552516210 | 0.772175 | 0.799853 | 0.797018 | 0.755195 |
| cg21470464 | 1.07E-04 | 7 | rs184495584;rs528235954 | 0.732146 | 0.760353 | 0.760321 | 0.708769 |
| cg26881802 | 1.07E-04 | 11 | rs554202168 | 0.809775 | 0.832711 | 0.83518 | 0.799839 |
| cg13654305 | 1.07E-04 | 1 | rs7543742;rs576714572 | 0.788681 | 0.808977 | 0.822469 | 0.76995 |
| cg08141194 | 1.06E-04 | 1 | rs528954187;rs550176154; rs562102479;rs956350; rs141736931;rs150159731 | 0.693519 | 0.733144 | 0.740035 | 0.658268 |
| cg13197072 | 1.06E-04 | 8 | rs116144355 | 0.616194 | 0.638438 | 0.638365 | 0.596533 |
| cg19425289 | 1.05E-04 | 5 | rs41270663;rs78084789 | 0.66145 | 0.685667 | 0.691545 | 0.651571 |
| cg13519191 | 1.05E-04 | 14 | rs149026922;rs535378614; rs554870137;rs376686206 | 0.762199 | 0.784966 | 0.801538 | 0.742764 |
| cg20411384 | 1.04E-04 | 7 | rs548661800;rs568603702; rs116051454 | 0.746666 | 0.768098 | 0.766526 | 0.725785 |
| cg24603102 | 1.04E-04 | 8 | rs192367038;rs17505079; rs11562816 | 0.773837 | 0.801056 | 0.805812 | 0.749289 |
| cg09046370 | 1.02E-04 | 5 |  | 0.739658 | 0.762039 | 0.763947 | 0.716647 |
| cg20863321 | 1.02E-04 | 2 | rs561510410 | 0.738643 | 0.764019 | 0.772441 | 0.715319 |
| cg22297466 | 1.02E-04 | 3 | rs367933941;rs181692114; rs185278288 | 0.71038 | 0.758884 | 0.755732 | 0.672994 |
| cg04601331 | 1.01E-04 | 12 | rs542843825;rs561322354 | 0.752443 | 0.775606 | 0.783843 | 0.721052 |
| cg01706030 | 1.01E-04 | 13 |  | 0.683816 | 0.717363 | 0.722309 | 0.627868 |
| cg13480465 | 1.00E-04 | 4 | rs574642605;rs148630106; rs557287787 | 0.676794 | 0.72029 | 0.708322 | 0.672738 |
| cg12159583 | 1.00E-04 | 9 |  | 0.152002 | 0.172879 | 0.17983 | 0.148722 |
| cg22118416 | 9.99E-05 | 4 | rs191636961;rs539702776; rs377111878;rs566509174 | 0.79152 | 0.815901 | 0.834 | 0.76496 |
| cg11435874 | 9.86E-05 | 9 | rs557618899 | 0.591118 | 0.6137 | 0.614277 | 0.580981 |
| cg14536993 | 9.79E-05 | 1 | rs192332874;rs185613390; rs553803430 | 0.592519 | 0.624602 | 0.635552 | 0.582003 |
| cg15936889 | 9.78E-05 | 22 | rs138060729;rs545253891 | 0.646175 | 0.675163 | 0.682233 | 0.629937 |
| cg07127693 | 9.66E-05 | 14 | rs142591942 | 0.839474 | 0.861964 | 0.848476 | 0.836003 |
| cg02423817 | 9.46E-05 | 11 | rs200586869;rs536852844 | 0.57241 | 0.625207 | 0.630757 | 0.529954 |
| cg01480833 | 9.43E-05 | 3 |  | 0.747636 | 0.767785 | 0.767076 | 0.716628 |
| cg00439750 | 9.40E-05 | 11 | rs139908576;rs77998334 | 0.549934 | 0.571498 | 0.57784 | 0.530411 |
| cg21715619 | 9.29E-05 | 4 | rs572704435;rs552371103; rs73080328;rs538374499 | 0.676873 | 0.701724 | 0.702958 | 0.639784 |
| cg26460286 | 9.23E-05 | 3 |  | 0.745333 | 0.769172 | 0.789045 | 0.720486 |
| cg00886225 | 9.15E-05 | 9 |  | 0.768207 | 0.791082 | 0.799151 | 0.741803 |
| cg00041047 | 9.15E-05 | 19 |  | 0.860337 | 0.881552 | 0.892764 | 0.814426 |
| cg02941028 | 9.09E-05 | 6 | rs547981797;rs146933540; rs117484986 | 0.770338 | 0.80165 | 0.802697 | 0.732763 |
| cg09680926 | 9.05E-05 | 15 | rs575111441 | 0.548073 | 0.590237 | 0.607883 | 0.555117 |
| cg19213799 | 8.99E-05 | 15 | rs568138833 | 0.689875 | 0.71763 | 0.736148 | 0.676507 |
| cg16424396 | 8.95E-05 | 10 | rs144579357 | 0.736423 | 0.763616 | 0.770103 | 0.714249 |
| cg05284888 | 8.91E-05 | 3 |  | 0.661048 | 0.701168 | 0.711653 | 0.635966 |
| cg20335953 | 8.85E-05 | 18 | rs571761189 | 0.722168 | 0.774835 | 0.764354 | 0.699786 |
| cg19736179 | 8.81E-05 | 14 | rs549308060;rs150646777 | 0.681982 | 0.725979 | 0.72277 | 0.668864 |
| cg21608953 | 8.61E-05 | 21 | rs543357120 | 0.787768 | 0.817371 | 0.814375 | 0.751473 |
| cg07408290 | 8.60E-05 | 17 | rs577228588;rs541519487 | 0.784786 | 0.809748 | 0.812142 | 0.760254 |
| cg02561376 | 8.55E-05 | 4 |  | 0.198771 | 0.219887 | 0.224067 | 0.178778 |
| cg05280527 | 8.53E-05 | 14 | rs540157210;rs556553789 | 0.544598 | 0.578661 | 0.573895 | NA |
| cg20899661 | 8.51E-05 | 17 | rs80149203;rs555555058 | 0.759274 | 0.784287 | 0.789711 | 0.735901 |
| cg14420496 | 8.50E-05 | 2 |  | 0.81667 | 0.844951 | 0.855589 | 0.825704 |
| cg24452282 | 8.48E-05 | 6 | rs4248160 | 0.708664 | 0.735728 | 0.733726 | 0.676437 |
| cg11354101 | 8.47E-05 | 17 | rs548029092;rs186261553 | 0.610218 | 0.632659 | 0.635109 | 0.572455 |
| cg01306003 | 8.41E-05 | 2 | rs556648989;rs575203822 | 0.771131 | 0.802425 | 0.804807 | 0.749165 |
| cg10733051 | 8.32E-05 | 6 | rs531148090 | 0.747259 | 0.769617 | 0.777512 | 0.724571 |
| cg18059941 | 8.29E-05 | 21 | rs73163018 | 0.720745 | 0.748849 | 0.759307 | 0.694207 |
| cg09363032 | 8.26E-05 | 4 | rs58115679;rs542636012; rs572144412 | 0.709162 | 0.74923 | 0.757825 | 0.671918 |
| cg23433242 | 8.03E-05 | 2 | rs56170971;rs182171056 | 0.738911 | 0.774191 | 0.774571 | 0.72628 |
| cg20809067 | 7.99E-05 | 17 | rs4790601 | 0.332033 | 0.354893 | 0.349645 | 0.312147 |
| cg10455314 | 7.99E-05 | 5 | rs188306216;rs113431645; rs545490979 | 0.756717 | 0.781685 | 0.785001 | 0.714925 |
| cg03613942 | 7.94E-05 | 5 | rs534947318 | 0.722709 | 0.747964 | 0.753419 | 0.695023 |
| cg13727618 | 7.94E-05 | 19 |  | 0.757733 | 0.790511 | 0.78367 | 0.748715 |
| cg03943613 | 7.94E-05 | 3 | rs527262913;rs550739362 | 0.735271 | 0.755631 | 0.765118 | 0.710371 |
| cg26088179 | 7.93E-05 | 12 | rs141354879 | 0.693882 | 0.71595 | 0.723425 | NA |
| cg07535790 | 7.85E-05 | 3 | rs10608546 | 0.750377 | 0.78523 | 0.78872 | 0.707398 |
| cg04787138 | 7.81E-05 | 20 | rs528254951;rs150904323; rs182393038;rs78270673 | 0.803517 | 0.827714 | 0.825439 | 0.788908 |
| cg15948960 | 7.81E-05 | 14 | rs542313332 | 0.775334 | 0.803397 | 0.822193 | 0.751728 |
| cg20730595 | 7.61E-05 | 22 | rs5758235;rs546292445; rs564574458 | 0.801212 | 0.824271 | 0.828872 | 0.784485 |
| cg06331306 | 7.50E-05 | 6 | rs575290453;rs542277210 | 0.702005 | 0.732749 | 0.724344 | 0.643341 |
| cg00187889 | 7.42E-05 | 6 | rs566625628;rs559110812; rs139997029 | 0.670274 | 0.70225 | 0.69803 | 0.652843 |
| cg04570358 | 7.40E-05 | 7 | rs567929107;rs147648554 | 0.769279 | 0.789871 | 0.788839 | 0.76365 |
| cg01476124 | 7.40E-05 | 12 | rs529983795;rs547738224; rs556472191 | 0.712396 | 0.746632 | 0.758698 | 0.693329 |
| cg08993055 | 7.39E-05 | 12 | rs150519915;rs556735280; rs568854149 | 0.611105 | 0.647606 | 0.64609 | 0.572887 |
| cg22831517 | 7.38E-05 | 4 | rs537666014;rs371661805; rs115881614 | 0.800633 | 0.825695 | 0.842966 | 0.79353 |
| cg11610626 | 7.32E-05 | 6 | rs376099062;rs556127676; rs568474640 | 0.539163 | 0.578065 | 0.585818 | 0.526562 |
| cg18436544 | 7.27E-05 | 13 | rs369976660 | 0.470845 | 0.492813 | 0.487457 | 0.444271 |
| cg19039140 | 7.25E-05 | 16 | rs556900429 | 0.677898 | 0.703431 | 0.722995 | 0.648026 |
| cg21920171 | 7.22E-05 | 18 | rs575776668;rs544416342; rs149269332 | 0.791789 | 0.813891 | 0.816766 | 0.773174 |
| cg03566388 | 7.15E-05 | 14 | rs541622394;rs562035976; rs530692622 | 0.588601 | 0.617435 | 0.60877 | 0.675247 |
| cg04994519 | 7.06E-05 | 12 |  | 0.735364 | 0.780241 | 0.770819 | 0.694846 |
| cg20513976 | 6.97E-05 | 20 | rs531268197;rs190544433; rs563317252;rs532196196 | 0.58978 | 0.615769 | 0.625311 | 0.530314 |
| cg03408779 | 6.96E-05 | 2 | rs576639865;rs543893221 | 0.797675 | 0.824539 | 0.814222 | 0.764899 |
| cg20207650 | 6.94E-05 | 7 | rs536869634;rs181833532 | 0.801798 | 0.827959 | 0.840534 | 0.78095 |
| cg21908960 | 6.90E-05 | 1 | rs186046151 | 0.790281 | 0.822472 | 0.825713 | 0.772845 |
| cg26919551 | 6.90E-05 | 10 | rs114310519;rs193109622 | 0.545285 | 0.565882 | 0.574044 | 0.507066 |
| cg03431588 | 6.84E-05 | 13 | rs548675300 | 0.771878 | 0.807979 | 0.799633 | 0.756035 |
| cg03208041 | 6.77E-05 | 8 | rs112503975;rs548408212 | 0.758256 | 0.781625 | 0.794531 | 0.736984 |
| cg05933789 | 6.63E-05 | 2 | rs549302122;rs559891729; rs114423961 | 0.749672 | 0.778082 | 0.79193 | 0.726038 |
| cg18558395 | 6.59E-05 | 5 | rs539221960 | 0.618743 | 0.642615 | 0.656489 | 0.616631 |
| cg23463608 | 6.52E-05 | 19 | rs550039299 | 0.55962 | 0.584299 | 0.586473 | 0.494813 |
| cg16349527 | 6.37E-05 | 2 | rs376022143 | 0.591939 | 0.614759 | 0.618372 | 0.547792 |
| cg09744194 | 6.31E-05 | 8 | rs551941297;rs562488594 | 0.545112 | 0.566895 | 0.5768 | NA |
| cg00155799 | 6.27E-05 | 2 | rs9973784 | 0.742313 | 0.778676 | 0.787551 | 0.730439 |
| cg07641497 | 6.26E-05 | 22 | rs139513596 | 0.724455 | 0.748384 | 0.7605 | 0.678402 |
| cg25816160 | 6.25E-05 | 1 | rs182151947 | 0.746813 | 0.772272 | 0.779392 | 0.707436 |
| cg23413777 | 6.15E-05 | 1 |  | 0.746421 | 0.785356 | 0.796153 | 0.716932 |
| cg24814040 | 6.12E-05 | 1 | rs139942342;rs78016505 | 0.694439 | 0.738466 | 0.722381 | 0.682825 |
| cg08338739 | 6.06E-05 | 4 | rs141702955 | 0.785066 | 0.812112 | 0.810904 | 0.763451 |
| cg11479389 | 6.04E-05 | 8 | rs141194392;rs544411859; rs561026245 | 0.592356 | 0.623036 | 0.613152 | 0.568174 |
| cg15786497 | 6.01E-05 | 3 | rs112418607;rs544058215 | 0.797557 | 0.828752 | 0.830043 | 0.785971 |
| cg03930929 | 5.98E-05 | 3 | rs537307054;rs557201881 | 0.789714 | 0.81819 | 0.812463 | 0.768815 |
| cg04742453 | 5.88E-05 | 8 | rs190520795;rs548629519 | 0.80199 | 0.822317 | 0.82923 | 0.788481 |
| cg15526437 | 5.74E-05 | 5 | rs189567630 | 0.760708 | 0.785017 | 0.779054 | 0.742635 |
| cg20495370 | 5.67E-05 | 14 | rs142813338;rs560499321; rs577160197;rs546368355; rs562951440;rs2065046 | 0.681224 | 0.722688 | 0.721434 | 0.647589 |
| cg21664866 | 5.59E-05 | 2 |  | 0.808378 | 0.838068 | 0.833004 | 0.791308 |
| cg19813814 | 5.58E-05 | 4 | rs183828507;rs532376236; rs138232290 | 0.76673 | 0.798738 | 0.791111 | 0.740732 |
| cg16261511 | 5.55E-05 | 1 | rs116743924 | 0.676734 | 0.699292 | 0.716155 | 0.658568 |
| cg10621390 | 5.51E-05 | 2 | rs564122307 | 0.737558 | 0.760288 | 0.768477 | 0.719796 |
| cg07117463 | 5.49E-05 | 1 | rs566939330;rs534759964; rs552745357;rs188356157; rs538220608;rs200510390; rs575190201 | 0.722873 | 0.759584 | 0.763086 | 0.677897 |
| cg25933842 | 5.40E-05 | 2 | rs539909632 | 0.592113 | 0.6195 | 0.605491 | 0.560801 |
| cg23256675 | 5.36E-05 | 5 |  | 0.804485 | 0.841869 | 0.831005 | 0.770557 |
| cg00154232 | 5.28E-05 | 13 | rs116082194;rs540764995; rs562177280;rs574152542 | 0.826755 | 0.847992 | 0.844591 | 0.820841 |
| cg01756700 | 5.25E-05 | 8 | rs28461614;rs190938859 | 0.790042 | 0.812757 | 0.817601 | 0.767977 |
| cg21223656 | 5.21E-05 | 10 | rs115372518 | 0.677358 | 0.703296 | 0.711847 | 0.657632 |
| cg00059664 | 5.15E-05 | 10 | rs140281371;rs202217604; rs368524364;rs144004164 | 0.862455 | 0.892119 | 0.89562 | NA |
| cg24744014 | 5.15E-05 | 2 | rs549012022;rs76814434 | 0.657607 | 0.679047 | 0.685121 | 0.632778 |
| cg18833386 | 5.13E-05 | 1 | rs541645619 | 0.795128 | 0.816301 | 0.832553 | 0.781921 |
| cg19197463 | 5.08E-05 | 1 | rs117515114 | 0.705094 | 0.739715 | 0.752124 | 0.696108 |
| cg17815944 | 5.00E-05 | 11 | rs557985397;rs577784868 | 0.760974 | 0.784135 | 0.800036 | 0.726251 |
| cg23096806 | 4.99E-05 | 10 | rs111639381 | 0.423937 | 0.449394 | 0.459052 | 0.417751 |
| cg25501139 | 4.93E-05 | 12 |  | 0.654495 | 0.679884 | 0.685923 | 0.636879 |
| cg12609063 | 4.92E-05 | 1 | rs565716173;rs536224444 | 0.493819 | 0.516608 | 0.532074 | 0.469043 |
| cg17036641 | 4.82E-05 | 3 | rs76101717 | 0.735091 | 0.761953 | 0.755677 | 0.72049 |
| cg14953379 | 4.72E-05 | 7 | rs568545394;rs535021394 | 0.797679 | 0.828338 | 0.832724 | 0.77221 |
| cg06326016 | 4.69E-05 | 3 | rs75909225;rs554607129; rs566648262;rs534081039; rs72851530 | 0.795819 | 0.815857 | 0.821822 | 0.777555 |
| cg14249990 | 4.64E-05 | 14 | rs527768856 | 0.564879 | 0.587084 | 0.605049 | 0.521182 |
| cg02384491 | 4.63E-05 | 1 |  | 0.767355 | 0.792644 | 0.789074 | 0.745472 |
| cg13375187 | 4.61E-05 | 15 | rs112165137;rs185847032; rs201673048 | 0.766957 | 0.792361 | 0.800665 | 0.746821 |
| cg06730002 | 4.49E-05 | 3 | rs79091774;rs538482674; rs554221535 | 0.55331 | 0.601672 | 0.610194 | 0.504859 |
| cg25481253 | 4.44E-05 | 7 | rs141196803;rs144536521; rs148448145;rs554958923 | 0.74737 | 0.772344 | 0.788443 | 0.736113 |
| cg16785409 | 4.34E-05 | 7 | rs115296488;rs2718054; rs564117268;rs532909886 | 0.809848 | 0.829922 | 0.839411 | 0.806468 |
| cg22861778 | 4.25E-05 | 4 | rs181089587;rs116514933; rs544756796 | 0.770517 | 0.796065 | 0.793126 | 0.753825 |
| cg03065503 | 4.20E-05 | 4 |  | 0.737359 | 0.765115 | 0.761704 | 0.718927 |
| cg19659935 | 4.18E-05 | 13 | rs7993067;rs117698403; rs540658231;rs562081253 | 0.739974 | 0.765312 | 0.769861 | 0.70773 |
| cg18422371 | 4.01E-05 | 11 | rs4151018;rs571445276 | 0.662065 | 0.695639 | 0.712868 | 0.637826 |
| cg24335133 | 3.99E-05 | 22 | rs535962441;rs185162927; rs572372378;rs540501842 | 0.567246 | 0.594606 | 0.593226 | 0.56692 |
| cg15114510 | 3.97E-05 | 11 | rs182858793;rs77760551 | 0.832036 | 0.854558 | 0.856575 | 0.812132 |
| cg04263658 | 3.97E-05 | 19 | rs78058280 | 0.759239 | 0.787442 | 0.786593 | 0.725794 |
| cg23665564 | 3.94E-05 | 8 | rs148421319;rs577554281; rs142570463 | 0.708073 | 0.749585 | 0.742203 | 0.69129 |
| cg09086620 | 3.91E-05 | 18 |  | 0.782517 | 0.812033 | 0.81674 | 0.743599 |
| cg08889157 | 3.89E-05 | 6 | rs147564620 | 0.81035 | 0.831656 | 0.836864 | 0.796175 |
| cg06095802 | 3.79E-05 | 11 | rs545297977 | 0.757968 | 0.790549 | 0.792106 | 0.751765 |
| cg05425585 | 3.74E-05 | 2 | rs191353199 | 0.822543 | 0.850426 | 0.844103 | 0.816737 |
| cg02771997 | 3.73E-05 | 7 | rs17136090;rs558027118 | 0.728656 | 0.753315 | 0.759495 | 0.671401 |
| cg24139912 | 3.72E-05 | 11 | rs113426908;rs111627523; rs565358859;rs148534302 | 0.68135 | 0.707113 | 0.706237 | 0.649683 |
| cg07250007 | 3.59E-05 | 5 | rs189107792 | 0.804957 | 0.829557 | 0.832426 | 0.787297 |
| cg26249510 | 3.59E-05 | 10 | rs73360865;rs529562993; rs551026624 | 0.520841 | 0.547756 | 0.562543 | 0.507789 |
| cg25226152 | 3.52E-05 | 10 |  | 0.736126 | 0.770249 | 0.771973 | 0.706586 |
| cg20272209 | 3.48E-05 | 21 | rs539831267;rs558225677; rs576608438 | 0.666153 | 0.693318 | 0.696461 | 0.585173 |
| cg22792560 | 3.46E-05 | 4 | rs140516520 | 0.378874 | 0.404228 | 0.402456 | 0.352638 |
| cg03371783 | 3.42E-05 | 7 | rs529891369 | 0.757791 | 0.789282 | 0.788157 | 0.730888 |
| cg18760423 | 3.40E-05 | 9 | rs543043187;rs201545266; rs200717973 | 0.776283 | 0.803563 | 0.789815 | 0.769156 |
| cg03961551 | 3.36E-05 | 1 | rs368007244;rs742232; rs114356209 | 0.554911 | 0.582673 | 0.601011 | 0.513979 |
| cg00920892 | 3.34E-05 | 8 | rs201230734;rs141536416 | 0.628179 | 0.648888 | 0.668114 | 0.584755 |
| cg12159213 | 3.33E-05 | 13 | rs557597791 | 0.822598 | 0.848456 | 0.847285 | NA |
| cg12136258 | 3.30E-05 | 13 | rs542862620;rs562353388 | 0.343288 | 0.3827 | 0.386257 | 0.350427 |
| cg03962250 | 3.22E-05 | 13 | rs554525641 | 0.711508 | 0.745678 | 0.743906 | 0.682691 |
| cg21303980 | 3.16E-05 | 2 | rs10169024;rs539506839; rs557910191;rs145471085 | 0.759539 | 0.78243 | 0.79201 | 0.740973 |
| cg01145975 | 3.11E-05 | 6 | rs536636715;rs566166642 | 0.653264 | 0.681036 | 0.689131 | 0.618397 |
| cg10431989 | 3.08E-05 | 3 | rs185994901;rs561319442 | 0.7721 | 0.797202 | 0.797612 | 0.738571 |
| cg14374996 | 3.05E-05 | 6 | rs193194513 | 0.809214 | 0.832541 | 0.836012 | 0.781932 |
| cg17741993 | 2.94E-05 | 6 | rs4645844 | 0.641183 | 0.694693 | 0.697688 | 0.593183 |
| cg08079935 | 2.93E-05 | 20 | rs57321259;rs552825771 | 0.762923 | 0.79412 | 0.797868 | 0.749315 |
| cg01823925 | 2.93E-05 | 5 | rs544574364;rs72811330; rs530239968 | 0.641219 | 0.663428 | 0.67589 | 0.596229 |
| cg13707972 | 2.85E-05 | 11 | rs529979524;rs368866160 | 0.775435 | 0.812713 | 0.816323 | 0.756197 |
| cg17124440 | 2.79E-05 | 3 | rs375502569;rs144729913 | 0.661181 | 0.686987 | 0.705259 | 0.608125 |
| cg01725658 | 2.79E-05 | 6 | rs562776766;rs79319672 | 0.750311 | 0.782708 | 0.794355 | 0.745813 |
| cg09699830 | 2.68E-05 | 16 | rs560254589;rs541539238 | 0.673392 | 0.698153 | 0.708377 | 0.62397 |
| cg12486124 | 2.68E-05 | 8 | rs79577465 | 0.706137 | 0.743256 | 0.753868 | 0.685272 |
| cg12224431 | 2.65E-05 | 11 | rs190588991;rs527580534; rs547147042 | 0.773929 | 0.807586 | 0.80464 | 0.756805 |
| cg09391799 | 2.65E-05 | 14 | rs529883389;rs546493615 | 0.431161 | 0.455248 | 0.459243 | 0.530879 |
| cg26980729 | 2.65E-05 | 6 | rs146486648;rs547051772 | 0.685121 | 0.724307 | 0.711309 | 0.670049 |
| cg04281882 | 2.63E-05 | 12 | rs551668207 | 0.743972 | 0.780665 | 0.769772 | 0.718984 |
| cg04101711 | 2.62E-05 | 2 | rs577483203;rs182445816 | 0.713041 | 0.742679 | 0.752477 | 0.701149 |
| cg09889490 | 2.62E-05 | 2 |  | 0.718728 | 0.753582 | 0.738659 | 0.689266 |
| cg19281794 | 2.57E-05 | 3 | rs535940209;rs61109654 | 0.222872 | 0.247308 | 0.266772 | 0.219954 |
| cg24291237 | 2.54E-05 | 1 | rs563558054;rs530955819; rs185213497;rs190266022 | 0.693145 | 0.732883 | 0.745598 | 0.666862 |
| cg16370268 | 2.53E-05 | 14 | rs536739266 | 0.72437 | 0.766659 | 0.762073 | 0.704405 |
| cg16479102 | 2.47E-05 | 6 | rs66988573;rs559708824; rs76126761;rs564234063 | 0.426621 | 0.449048 | 0.453555 | 0.394061 |
| cg02164748 | 2.43E-05 | 6 | rs117218761 | 0.671082 | 0.698726 | 0.705743 | 0.632758 |
| cg01188753 | 2.40E-05 | 20 |  | 0.746647 | 0.768882 | 0.784937 | 0.727772 |
| cg11923734 | 2.39E-05 | 3 | rs555841757;rs147244367 | 0.809398 | 0.835509 | 0.832298 | 0.767175 |
| cg04010004 | 2.31E-05 | 1 | rs368081909;rs545081811 | 0.745185 | 0.771286 | 0.780788 | 0.720517 |
| cg04404259 | 2.29E-05 | 3 |  | 0.737254 | 0.768148 | 0.77173 | 0.700135 |
| cg25170017 | 2.26E-05 | 11 | rs529487538;rs683507; rs568919105 | 0.562811 | 0.583516 | 0.595215 | 0.504164 |
| cg24315843 | 2.24E-05 | 9 | rs186259429;rs533797426; rs527338845 | 0.753966 | 0.781226 | 0.783245 | 0.733755 |
| cg08441850 | 2.08E-05 | 17 | rs572289583;rs542583248 | 0.56992 | 0.591519 | 0.60506 | 0.54355 |
| cg07028680 | 2.04E-05 | 6 | rs142724817;rs9400646 | 0.81607 | 0.841566 | 0.843059 | 0.790642 |
| cg11813949 | 2.03E-05 | 11 | rs559637829;rs145184326 | 0.668934 | 0.704002 | 0.716638 | 0.648717 |
| cg01674623 | 2.00E-05 | 7 | rs536702731 | 0.768235 | 0.792677 | 0.789442 | 0.733011 |
| cg24869834 | 1.98E-05 | 4 | rs566311554;rs140899892 | 0.771433 | 0.807776 | 0.808335 | 0.739675 |
| cg05703000 | 1.98E-05 | 14 | rs560538912;rs532672688; rs552731616;rs139208641 | 0.445102 | 0.477208 | 0.482836 | 0.438949 |
| cg08976426 | 1.94E-05 | 4 | rs538037208 | 0.765338 | 0.791458 | 0.799007 | 0.750399 |
| cg14373410 | 1.93E-05 | 14 |  | 0.731717 | 0.755767 | 0.765212 | 0.674524 |
| cg11985492 | 1.85E-05 | 4 |  | 0.781201 | 0.808772 | 0.806933 | 0.76434 |
| cg20029869 | 1.84E-05 | 5 | rs138457962;rs112626686 | 0.821349 | 0.853357 | 0.869802 | 0.83458 |
| cg07235562 | 1.75E-05 | 10 | rs150657578;rs55959406; rs530338324 | 0.34959 | 0.396702 | 0.396777 | 0.36094 |
| cg02795870 | 1.73E-05 | 6 | rs528795874;rs1625781; rs41288669;rs9262380; rs112168055;rs41288679; rs144503906;rs548248760 | 0.647124 | 0.690845 | 0.682813 | 0.625047 |
| cg02632952 | 1.70E-05 | 3 | rs563842745;rs577429034 | 0.703716 | 0.735242 | 0.74775 | 0.684698 |
| cg13487614 | 1.69E-05 | 6 |  | 0.697314 | 0.733624 | 0.72834 | 0.65811 |
| cg26461919 | 1.68E-05 | 14 | rs534728153;rs554974079; rs145637820 | 0.770471 | 0.792979 | 0.802076 | 0.721457 |
| cg13521913 | 1.61E-05 | 12 | rs559153959;rs373948679 | 0.767537 | 0.78956 | 0.779584 | 0.753576 |
| cg00070529 | 1.61E-05 | 13 | rs555625713;rs111580400 | 0.689737 | 0.735132 | 0.735161 | 0.679342 |
| cg11205926 | 1.60E-05 | 6 | rs11965933 | 0.682667 | 0.734856 | 0.73712 | 0.677377 |
| cg17683940 | 1.59E-05 | 15 | rs555735445;rs572770848; rs540268271 | 0.769011 | 0.795827 | 0.814017 | 0.731505 |
| cg10075638 | 1.56E-05 | 15 | rs374879195 | 0.5966 | 0.623375 | 0.627172 | 0.579056 |
| cg25571136 | 1.49E-05 | 6 | rs530960902;rs368855996 | 0.778277 | 0.808269 | 0.806303 | 0.721612 |
| cg17427728 | 1.48E-05 | 2 | rs537368365;rs148091439; rs567931805 | 0.706996 | 0.731368 | 0.729171 | 0.677146 |
| cg24414363 | 1.46E-05 | 22 | rs73165147 | 0.564246 | 0.585872 | 0.589743 | 0.532698 |
| cg23765831 | 1.44E-05 | 7 | rs58389569;rs78013364 | 0.743792 | 0.775884 | 0.776258 | 0.733401 |
| cg17587997 | 1.41E-05 | 6 | rs80018833;rs544756997 | 0.701336 | 0.723433 | 0.736035 | 0.647868 |
| cg10307691 | 1.39E-05 | 4 |  | 0.764056 | 0.784114 | 0.796571 | 0.746534 |
| cg08920681 | 1.31E-05 | 13 | rs181386249;rs531060645 | 0.710524 | 0.757273 | 0.765228 | 0.737813 |
| cg21489565 | 1.27E-05 | 5 | rs189154120;rs540939396 | 0.77061 | 0.803776 | 0.814656 | 0.76123 |
| cg02240296 | 1.25E-05 | 17 |  | 0.759867 | 0.78905 | 0.786048 | 0.733313 |
| cg07200939 | 1.22E-05 | 12 | rs372041593;rs78460632 | 0.813572 | 0.847789 | 0.844081 | 0.809036 |
| cg13504554 | 1.20E-05 | 6 | rs2473581 | 0.55198 | 0.586135 | 0.587544 | 0.552743 |
| cg16288834 | 1.20E-05 | 17 | rs550129278 | 0.722546 | 0.745166 | 0.755023 | 0.676371 |
| cg20239766 | 1.15E-05 | 17 | rs561871589;rs71373921; rs529049882 | 0.629856 | 0.662268 | 0.679114 | 0.6004 |
| cg15761265 | 1.13E-05 | 11 | rs563642533;rs531027457 | 0.720269 | 0.752157 | 0.771149 | 0.694811 |
| cg08423443 | 1.11E-05 | 20 | rs143676298;rs547448949 | 0.732905 | 0.755504 | 0.770632 | 0.715498 |
| cg25055447 | 1.07E-05 | 5 | rs180752413;rs75433967 | 0.724311 | 0.753284 | 0.764143 | 0.69109 |
| cg05306123 | 1.07E-05 | 1 |  | 0.813913 | 0.836419 | 0.832896 | 0.78485 |
| cg01385870 | 1.03E-05 | 5 | rs73784341 | 0.735193 | 0.756707 | 0.762333 | 0.712716 |
| cg09046193 | 9.83E-06 | 10 | rs528546717;rs80034049; rs145442143 | 0.792247 | 0.818152 | 0.810509 | 0.759069 |
| cg08017268 | 9.56E-06 | 17 | rs560248951 | 0.732023 | 0.778279 | 0.784369 | 0.699662 |
| cg02150029 | 9.43E-06 | 1 | rs543750801 | 0.734144 | 0.758303 | 0.759516 | 0.774912 |
| cg08053787 | 9.34E-06 | 21 | rs372413488 | 0.64307 | 0.668393 | 0.681966 | 0.606735 |
| cg02711994 | 8.78E-06 | 4 | rs11573770;rs554519856 | 0.749495 | 0.772131 | 0.781384 | 0.698359 |
| cg11132334 | 8.77E-06 | 4 | rs151201878 | 0.397294 | 0.417675 | 0.423656 | 0.38299 |
| cg03876812 | 8.33E-06 | 1 |  | 0.785066 | 0.808369 | 0.805303 | 0.753493 |
| cg08452590 | 7.87E-06 | 2 | rs193148197;rs535117573 | 0.797153 | 0.827101 | 0.831467 | 0.776331 |
| cg12550767 | 7.29E-06 | 1 | rs149522642;rs201221938 | 0.813313 | 0.837135 | 0.832305 | 0.790821 |
| cg11589017 | 6.81E-06 | 3 | rs199954782;rs556407931 | 0.746628 | 0.77635 | 0.772268 | 0.712127 |
| cg16515745 | 6.33E-06 | 15 | rs150627090 | 0.792536 | 0.822507 | 0.831314 | 0.785096 |
| cg06616506 | 5.81E-06 | 7 | rs534322881 | 0.804296 | 0.833158 | 0.828269 | 0.804191 |
| cg26521730 | 5.53E-06 | 7 | rs371329848 | 0.788143 | 0.81644 | 0.810597 | 0.757079 |
| cg06290379 | 5.47E-06 | 5 | rs191796758 | 0.598309 | 0.618667 | 0.622521 | 0.549804 |
| cg02466892 | 5.25E-06 | 3 | rs190896398 | 0.736617 | 0.766911 | 0.778728 | 0.725951 |
| cg14764476 | 5.25E-06 | 5 | rs146959621;rs574009432 | 0.775632 | 0.79892 | 0.817911 | 0.758195 |
| cg10062091 | 5.13E-06 | 10 | rs1255482;rs553984477 | 0.75814 | 0.791913 | 0.789813 | 0.731381 |
| cg00603376 | 4.71E-06 | 1 | rs17025863 | 0.771991 | 0.79311 | 0.804577 | 0.756739 |
| cg18720622 | 4.64E-06 | 5 | rs575120009;rs542116540 | 0.707004 | 0.745194 | 0.750692 | NA |
| cg23277505 | 4.23E-06 | 3 | rs531542488;rs551655091 | 0.777524 | 0.801139 | 0.796646 | 0.739714 |
| cg05776323 | 3.88E-06 | 1 | rs115888337 | 0.321602 | 0.344495 | 0.354379 | 0.284503 |
| cg17685004 | 3.84E-06 | 2 | rs528162172;rs546493718; rs191405199;rs529203355 | 0.70064 | 0.744864 | 0.75876 | 0.675379 |
| cg01604152 | 3.39E-06 | 3 | rs541505948;rs73836169 | 0.774807 | 0.802612 | 0.799557 | 0.766245 |
| cg25845027 | 3.21E-06 | 8 | rs188910101;rs148822139; rs543642433 | 0.72415 | 0.752921 | 0.757793 | 0.717621 |
| cg20361664 | 3.17E-06 | 12 | rs539353540;rs558793167 | 0.798058 | 0.827638 | 0.828861 | 0.762585 |
| cg13223095 | 3.11E-06 | 7 |  | 0.792327 | 0.812958 | 0.82241 | 0.769056 |
| cg18356337 | 2.13E-06 | 2 | rs188945944;rs75898153; rs113147648;rs570047926 | 0.772691 | 0.792733 | 0.772801 | 0.820445 |
| cg02521291 | 1.70E-06 | 4 | rs116762586;rs114774496 | 0.809032 | 0.834511 | 0.846206 | 0.799297 |
| cg11217518 | 1.66E-06 | 2 |  | 0.805159 | 0.830143 | 0.823493 | 0.768117 |
| cg01420103 | 1.60E-06 | 5 | rs144006285;rs565075252 | 0.806726 | 0.829588 | 0.831225 | 0.77117 |
| cg12663303 | 1.51E-06 | 6 | rs577960207;rs149689932; rs553739174;rs565371613; rs145490088 | 0.729939 | 0.761763 | 0.763039 | 0.704801 |
| cg10662221 | 1.39E-06 | 2 | rs534669814 | 0.50039 | 0.551772 | 0.540147 | 0.453384 |
| cg24877190 | 1.36E-06 | 1 | rs116964657;rs143043876; rs192254983 | 0.251235 | 0.2717 | 0.263937 | 0.229565 |
| cg26678245 | 1.27E-06 | 4 | rs561373798;rs565669245 | 0.786766 | 0.809471 | 0.814035 | 0.753203 |
| cg08884324 | 1.16E-06 | 18 | rs531470498 | 0.788671 | 0.815989 | 0.82884 | 0.764474 |
| cg20377347 | 7.15E-07 | 10 | rs553551687;rs186125192 | 0.742982 | 0.782391 | 0.784931 | 0.714779 |
| cg17408993 | 7.01E-07 | 12 | rs190758540;rs540848251 | 0.34047 | 0.366235 | 0.381046 | 0.360518 |
| cg26213943 | 5.58E-07 | 7 | rs181933310 | 0.782732 | 0.807935 | 0.813475 | 0.764964 |
| cg26381611 | 4.76E-07 | 4 | rs192782179;rs573521978 | 0.714445 | 0.743531 | 0.762561 | 0.717861 |
| cg26703001 | 3.43E-07 | 15 | rs184995083;rs548058787; rs187566491 | 0.777671 | 0.804196 | 0.7958 | 0.747364 |
| cg13130683 | 2.71E-07 | 13 | rs117444444;rs575211795 | 0.752729 | 0.77835 | 0.794918 | 0.729526 |
| cg24785663 | 1.04E-07 | 2 | rs533283965 | 0.816497 | 0.842886 | 0.832549 | 0.792171 |
| cg13568659 | 3.22E-08 | 6 | rs183352464 | 0.601114 | 0.633017 | 0.625255 | 0.58224 |

# **Supplementary Table 11.** **The 122 CpG sites identified with significantly differentially methylated in pregnancy compared with non-pregnancy groups both in the White WM et al and in this study.**

| Target ID | Gene | P-value (this study) | P-value (White et al) |
| --- | --- | --- | --- |
| cg00138126 | TMEPAI | 5.98E-03 | 4.69E-06 |
| cg00350296 | CD248 | 8.70E-03 | 2.20E-04 |
| cg00415993 | F2RL2 | 5.26E-04 | 1.79E-04 |
| cg00443307 | KLRG1 | 2.71E-03 | 2.29E-05 |
| cg00546897 |  | 4.94E-03 | 2.79E-04 |
| cg00615241 | PRTN3 | 2.34E-04 | 1.43E-04 |
| cg00739120 | NIFIE14 | 5.44E-03 | 5.01E-04 |
| cg01031251 | RPS6KA1 | 3.28E-04 | 4.56E-05 |
| cg01129847 | C19orf35 | 5.91E-04 | 1.56E-04 |
| cg01274660 | TRIP6 | 7.93E-03 | 4.06E-04 |
| cg01402255 | GATAD2B | 1.22E-04 | 1.32E-04 |
| cg01441777 | CSNK1E | 1.44E-03 | 2.45E-04 |
| cg01578324 | CLIC1 | 6.39E-05 | 3.23E-04 |
| cg01636591 | CCL8 | 7.93E-03 | 8.51E-05 |
| cg01813965 | C16orf50 | 2.45E-03 | 2.41E-04 |
| cg01980222 | TREM2 | 4.16E-04 | 9.89E-06 |
| cg02357714 | DOK3 | 4.94E-03 | 3.42E-04 |
| cg02679745 | FUT7 | 1.44E-03 | 1.61E-04 |
| cg03165378 |  | 3.67E-03 | 4.87E-04 |
| cg03330678 |  | 7.43E-04 | 4.60E-04 |
| cg03924115 | P518 | 7.43E-04 | 3.54E-04 |
| cg04784315 | GPR21 | 1.07E-04 | 7.00E-05 |
| cg04988978 | MPO | 5.98E-03 | 8.54E-06 |
| cg05501357 | HIPK3 | 5.26E-04 | 1.43E-05 |
| cg05718253 |  | 8.70E-03 | 1.42E-04 |
| cg06172871 | HP | 9.45E-05 | 6.85E-05 |
| cg07239938 | ELA2 | 4.16E-04 | 3.41E-04 |
| cg07285167 | CSF3R | 2.02E-04 | 2.29E-04 |
| cg07685786 | S100B | 4.48E-03 | 2.50E-05 |
| cg07924575 | HPS4 | 8.70E-03 | 4.87E-04 |
| cg08130265 | C15orf5 | 3.70E-04 | 3.95E-04 |
| cg08176694 | PITPNM2 | 5.91E-04 | 1.15E-04 |
| cg08290628 | CORO2B | 4.16E-04 | 5.55E-05 |
| cg08399444 | GSG1 | 8.70E-03 | 7.07E-06 |
| cg08458487 | SFTPD | 2.55E-04 | 2.50E-04 |
| cg08700306 | LRP3 | 7.93E-03 | 4.25E-04 |
| cg08872742 | CDH5 | 5.98E-03 | 7.07E-05 |
| cg09001777 | FUT3 | 4.93E-03 | 1.56E-05 |
| cg09358725 | LMO2 | 4.68E-04 | 2.47E-04 |
| cg09421562 | MPO | 5.26E-04 | 2.87E-04 |
| cg09584711 | HPR | 1.39E-04 | 1.46E-04 |
| cg09624565 | NCF4 | 8.70E-03 | 3.75E-04 |
| cg10057295 | STK24 | 3.70E-04 | 9.05E-05 |
| cg10257049 | C5orf4 | 1.39E-04 | 2.67E-04 |
| cg10307548 | SOD3 | 1.29E-03 | 2.74E-05 |
| cg11024597 | ECRG4 | 1.14E-04 | 3.47E-05 |
| cg11471401 | KRT6A | 1.61E-03 | 1.58E-04 |
| cg11939496 | CD244 | 3.70E-04 | 1.06E-04 |
| cg12089698 | SPATC1 | 5.44E-03 | 5.65E-06 |
| cg12845808 | PCDH12 | 1.39E-04 | 7.24E-05 |
| cg12907644 | SAA2 | 1.22E-04 | 2.04E-06 |
| cg12949760 | KCNQ1 | 4.48E-03 | 4.06E-05 |
| cg13030582 | MFAP4 | 7.23E-03 | 3.23E-04 |
| cg13053608 | LGP1 | 9.45E-05 | 1.82E-04 |
| cg13703437 | FYB | 5.91E-04 | 7.36E-05 |
| cg14023451 | GPLD1 | 5.91E-04 | 9.85E-05 |
| cg14088811 | SPI1 | 5.91E-04 | 2.50E-04 |
| cg14511156 | OSCAR | 5.91E-04 | 9.23E-05 |
| cg14654385 | URP2 | 1.78E-04 | 1.71E-04 |
| cg14700707 | NOTCH4 | 2.71E-03 | 1.72E-04 |
| cg14870461 | AER61 | 2.58E-04 | 7.19E-05 |
| cg15227982 | C10orf26 | 2.71E-03 | 1.45E-04 |
| cg15248035 | CCIN | 4.55E-04 | 3.93E-04 |
| cg15337006 | ITGAM | 4.06E-03 | 4.31E-04 |
| cg15484375 | SAA1 | 2.91E-04 | 4.21E-05 |
| cg15528736 | FCGRT | 1.22E-04 | 3.73E-05 |
| cg15662251 | PAQR7 | 7.23E-03 | 1.39E-04 |
| cg15674432 | SLC26A8 | 7.93E-03 | 2.90E-06 |
| cg15880738 | CD3G | 5.91E-04 | 3.45E-04 |
| cg15937958 | UNQ473 | 1.78E-04 | 2.74E-05 |
| cg16003913 | MPG | 4.06E-03 | 2.22E-04 |
| cg16465939 | KCNQ1 | 3.32E-03 | 3.60E-05 |
| cg16504798 | MYO1F | 3.28E-04 | 3.08E-04 |
| cg16545105 | CRHBP | 2.85E-05 | 4.67E-05 |
| cg16745604 | CASP10 | 1.79E-03 | 1.86E-04 |
| cg17122311 | IL27 | 2.28E-04 | 2.28E-04 |
| cg17166812 | NDUFS2 | 7.95E-06 | 6.46E-05 |
| cg17386185 | GLYCTK | 3.67E-03 | 1.93E-04 |
| cg17740645 | GRB7 | 1.52E-03 | 5.12E-04 |
| cg17753124 | IER2 | 6.39E-05 | 1.10E-04 |
| cg17777592 | CAB39L | 1.44E-03 | 2.02E-04 |
| cg17823175 | AZU1 | 3.32E-03 | 1.97E-04 |
| cg17922226 | CLCN1 | 4.48E-03 | 1.23E-04 |
| cg18145683 | JAK3 | 5.26E-04 | 1.71E-04 |
| cg18638581 | HK2 | 8.30E-05 | 2.22E-04 |
| cg18854666 | SLC11A1 | 1.99E-03 | 6.87E-05 |
| cg18934187 | STARD6 | 2.28E-04 | 1.37E-04 |
| cg19304352 | DEFA4 | 6.58E-03 | 3.03E-05 |
| cg19399532 | FLJ35530 | 7.43E-04 | 1.88E-04 |
| cg19906550 | SLC22A18 | 1.29E-03 | 2.63E-05 |
| cg20018806 | TCN1 | 6.39E-05 | 3.53E-05 |
| cg20098659 | CLEC9A | 5.91E-04 | 3.77E-05 |
| cg20340242 | IL1R2 | 2.58E-04 | 3.97E-04 |
| cg20713492 | AQP10 | 7.93E-03 | 2.09E-04 |
| cg20748065 | POR | 7.01E-04 | 9.86E-05 |
| cg21019522 | SLC22A18 | 2.45E-03 | 1.55E-04 |
| cg21033855 | ZNF690 | 2.45E-03 | 3.59E-04 |
| cg21126943 | CEACAM6 | 2.21E-03 | 4.73E-04 |
| cg21237418 | RAB34 | 4.16E-04 | 2.02E-05 |
| cg21283680 | SH3BP5 | 3.34E-05 | 4.54E-04 |
| cg21685427 | SGK2 | 7.93E-03 | 1.36E-04 |
| cg21846903 | VTN | 1.79E-03 | 3.50E-04 |
| cg22045288 | C10orf91 | 5.26E-04 | 7.84E-05 |
| cg22242539 | SERPINF1 | 5.98E-03 | 4.42E-05 |
| cg22820108 | NCOR2 | 2.71E-03 | 4.65E-04 |
| cg23090046 | KNS2 | 2.02E-04 | 4.81E-04 |
| cg23506842 | PTPN7 | 7.93E-03 | 7.69E-06 |
| cg23713742 | SPAG4 | 2.91E-04 | 2.86E-06 |
| cg23889010 | SLPI | 3.09E-04 | 2.11E-04 |
| cg24211388 | AIF1 | 1.07E-04 | 2.63E-04 |
| cg24354652 | PTAFR | 1.78E-04 | 2.13E-04 |
| cg24427660 | PNPLA2 | 5.91E-04 | 4.90E-04 |
| cg24453664 | CD59 | 5.44E-03 | 4.30E-04 |
| cg24621042 | SERPINA1 | 3.00E-03 | 7.10E-05 |
| cg24777950 | CTSG | 1.29E-03 | 1.77E-04 |
| cg24821554 | GUCY1B2 | 8.70E-03 | 1.76E-04 |
| cg25059899 | BCL2 | 4.94E-03 | 3.30E-04 |
| cg25341726 | IL27 | 2.21E-03 | 3.64E-04 |
| cg25402049 | PRDM2 | 8.29E-05 | 2.19E-05 |
| cg25600606 | HIPK3 | 2.91E-04 | 3.93E-05 |
| cg26112639 | CIAS1 | 9.53E-03 | 4.35E-05 |
| cg26306976 | ITGB1BP1 | 2.14E-04 | 1.50E-04 |

# **Supplementary table 12. The 133 CpG sites identified with significantly differentially methylated in pregnancy compared with non-pregnancy groups both in the Gruzieva et al and in this study.**

| Target ID | Gene | P-value (this study) | P-value (Gruzieva et al) |
| --- | --- | --- | --- |
| cg00052692 |  | 5.91E-04 | 1.28E-09 |
| cg00085812 |  | 5.26E-04 | 1.29E-07 |
| cg00358010 |  | 3.70E-04 | 8.26E-07 |
| cg00782811 | RCAN2 | 7.93E-03 | 4.56E-07 |
| cg00840791 |  | 7.43E-04 | 5.50E-08 |
| cg00868074 | ZCCHC14 | 2.71E-03 | 7.86E-10 |
| cg00989806 | NDUFA10 | 2.85E-05 | 1.12E-06 |
| cg01059398 | TNFSF10 | 1.99E-03 | 2.60E-08 |
| cg01072106 | NACC2 | 1.63E-05 | 3.72E-10 |
| cg01304182 | ZNF48 | 1.22E-04 | 1.10E-06 |
| cg01572694 | MIR10A | 4.10E-04 | 4.71E-07 |
| cg01819502 | GGT6 | 1.29E-03 | 3.42E-07 |
| cg01826979 | NTRK3 | 1.61E-03 | 1.86E-08 |
| cg01936839 |  | 4.40E-06 | 8.50E-09 |
| cg02146895 |  | 2.16E-05 | 4.33E-07 |
| cg02266185 | TANC1 | 1.57E-04 | 5.07E-07 |
| cg02464519 |  | 7.43E-04 | 4.18E-09 |
| cg02571436 |  | 8.70E-03 | 1.36E-07 |
| cg02836478 | HOXB3 | 1.78E-04 | 2.07E-09 |
| cg03029755 |  | 5.91E-04 | 6.16E-07 |
| cg03044533 | MAML2 | 7.43E-04 | 1.38E-07 |
| cg03351508 |  | 9.83E-04 | 3.46E-07 |
| cg03450635 | FAM198B | 2.28E-04 | 2.03E-09 |
| cg03513101 |  | 4.16E-04 | 7.60E-08 |
| cg03550384 | NECAB1 | 2.46E-04 | 1.40E-08 |
| cg03746015 | CLEC16A | 8.31E-04 | 7.01E-07 |
| cg03910896 | NSMCE1 | 1.04E-03 | 1.17E-08 |
| cg03958883 | ARHGEF17 | 1.16E-03 | 1.67E-07 |
| cg04014328 | HOXB4 | 2.02E-04 | 2.18E-08 |
| cg04040227 | ZNF366 | 7.28E-05 | 8.13E-07 |
| cg04213565 |  | 1.29E-03 | 1.23E-09 |
| cg04253214 |  | 1.29E-03 | 1.58E-08 |
| cg04590659 | PFKFB3 | 2.45E-03 | 5.45E-07 |
| cg04629221 |  | 3.28E-04 | 2.96E-07 |
| cg04725636 | DNAJC5B | 4.28E-05 | 8.51E-10 |
| cg04753874 |  | 2.91E-04 | 1.18E-09 |
| cg04763377 |  | 1.57E-04 | 2.04E-07 |
| cg04816140 |  | 2.48E-05 | 1.60E-07 |
| cg05186879 | MAPKAPK3 | 1.61E-03 | 1.00E-09 |
| cg05230392 | CUEDC1 | 1.88E-05 | 1.46E-10 |
| cg05648752 | CHSY1 | 1.78E-04 | 3.59E-08 |
| cg05844798 |  | 9.45E-05 | 3.15E-11 |
| cg05850884 | TRIM26 | 1.23E-05 | 5.93E-07 |
| cg06072257 |  | 4.16E-04 | 2.75E-09 |
| cg06096446 | TULP4 | 2.91E-04 | 1.21E-07 |
| cg06186155 | HOXB3 | 9.53E-03 | 3.31E-08 |
| cg06203221 |  | 2.16E-05 | 1.83E-07 |
| cg06437891 |  | 2.48E-05 | 5.79E-07 |
| cg06528771 |  | 1.88E-05 | 1.00E-06 |
| cg06556981 | SLC6A6 | 1.07E-04 | 8.09E-07 |
| cg06633438 | MLLT1 | 5.26E-04 | 6.24E-07 |
| cg07252680 | SERPINA1 | 3.74E-05 | 4.73E-11 |
| cg07263393 | GATA2 | 5.44E-03 | 1.24E-09 |
| cg07573872 | SBNO2 | 4.40E-06 | 3.15E-07 |
| cg08122070 | NADK | 1.41E-05 | 3.12E-07 |
| cg08285980 | UBASH3B | 3.26E-05 | 4.75E-07 |
| cg08493167 |  | 1.61E-03 | 3.50E-07 |
| cg08894561 |  | 1.88E-05 | 8.00E-07 |
| cg09178900 | RNF146 | 2.48E-05 | 9.78E-07 |
| cg09324566 | ST3GAL3 | 2.58E-04 | 1.13E-08 |
| cg09349128 |  | 9.19E-06 | 1.24E-07 |
| cg09430344 | NSMCE1 | 1.44E-03 | 3.28E-08 |
| cg09981464 | ZCCHC14 | 3.32E-03 | 3.79E-09 |
| cg10045881 | CHI3L2 | 7.95E-06 | 4.92E-07 |
| cg10843276 | PCGF3 | 1.99E-03 | 9.54E-07 |
| cg11629527 | MIR3155B | 5.59E-05 | 2.67E-10 |
| cg11795922 | PLB1 | 2.28E-04 | 1.80E-08 |
| cg11902329 |  | 5.59E-05 | 8.16E-10 |
| cg12054453 | TMEM49 | 1.57E-04 | 1.12E-06 |
| cg12992827 |  | 3.79E-06 | 2.93E-08 |
| cg12999836 | GRB10 | 2.80E-06 | 2.26E-07 |
| cg13074526 |  | 9.45E-05 | 6.25E-09 |
| cg13178755 | ZAK | 9.45E-05 | 6.07E-07 |
| cg13562751 | PHACTR1 | 4.48E-03 | 9.27E-07 |
| cg13634839 | TREM1 | 6.39E-05 | 5.84E-08 |
| cg13652985 | MIR10A | 2.21E-03 | 1.03E-06 |
| cg13667088 |  | 2.16E-05 | 2.78E-11 |
| cg13781414 | NACC2 | 1.16E-03 | 1.89E-07 |
| cg13975855 | HOXB3 | 1.39E-04 | 7.23E-08 |
| cg14162417 |  | 5.91E-04 | 6.52E-08 |
| cg14380013 | UNC119B | 1.23E-05 | 6.18E-07 |
| cg14410964 | NOTCH1 | 4.16E-04 | 8.85E-09 |
| cg15262980 |  | 4.16E-04 | 8.21E-07 |
| cg15293367 |  | 3.67E-03 | 8.97E-07 |
| cg15321908 |  | 6.39E-05 | 1.18E-06 |
| cg15548624 | SPECC1L | 1.07E-04 | 9.47E-08 |
| cg15678744 | SH3PXD2B | 3.32E-03 | 7.30E-08 |
| cg15745450 | KDM4B | 3.32E-03 | 1.80E-07 |
| cg15839964 | IL22RA2 | 3.79E-06 | 5.40E-08 |
| cg16207944 |  | 1.79E-03 | 3.42E-07 |
| cg16936953 | TMEM49 | 1.07E-04 | 1.70E-07 |
| cg17610574 |  | 2.45E-03 | 4.65E-09 |
| cg17634927 | ADAM23 | 4.06E-03 | 3.84E-07 |
| cg17743555 |  | 2.21E-03 | 7.43E-09 |
| cg18146146 | STRN4 | 1.04E-03 | 1.40E-07 |
| cg18814049 | NCF2 | 2.45E-03 | 8.91E-08 |
| cg19418318 | MYO9B | 6.63E-04 | 9.59E-07 |
| cg19748455 |  | 5.91E-04 | 1.60E-11 |
| cg19750024 | BMP1 | 4.90E-05 | 5.83E-07 |
| cg19821297 |  | 4.40E-06 | 3.22E-08 |
| cg19893929 |  | 2.21E-03 | 4.94E-07 |
| cg20090157 |  | 9.30E-04 | 9.43E-07 |
| cg20112774 |  | 1.22E-04 | 6.55E-08 |
| cg20137868 | CUEDC1 | 1.61E-03 | 4.76E-07 |
| cg20361768 |  | 4.40E-06 | 3.98E-08 |
| cg20701457 | ICAM2 | 4.16E-04 | 1.07E-07 |
| cg20789677 | SUPT3H | 9.53E-03 | 6.53E-07 |
| cg20995564 | ZEB2 | 9.19E-06 | 2.89E-10 |
| cg21461811 |  | 2.84E-05 | 5.55E-07 |
| cg22260008 |  | 5.91E-04 | 1.04E-06 |
| cg22298224 | SART1 | 7.28E-05 | 9.46E-08 |
| cg22652934 | RUNX1 | 4.16E-04 | 8.85E-08 |
| cg22792560 |  | 3.32E-03 | 3.44E-07 |
| cg22994883 |  | 3.79E-06 | 3.76E-08 |
| cg23014425 | HOXB3 | 1.61E-03 | 2.74E-10 |
| cg23257987 |  | 7.93E-03 | 5.10E-09 |
| cg23821206 | RABGAP1 | 2.02E-04 | 1.36E-07 |
| cg23891487 | SOCS2 | 3.67E-03 | 1.45E-07 |
| cg24025566 | AVP | 6.58E-03 | 1.48E-07 |
| cg24356337 | KIAA0825 | 8.31E-04 | 7.92E-08 |
| cg24621042 | SERPINA1 | 3.00E-03 | 9.22E-09 |
| cg25112191 | RORC | 1.06E-05 | 9.97E-08 |
| cg25325512 | PIM1 | 6.63E-04 | 7.23E-07 |
| cg25436217 | MGAT3 | 1.39E-04 | 1.15E-09 |
| cg25437672 | PDLIM4 | 8.31E-04 | 3.37E-08 |
| cg25712984 |  | 7.43E-04 | 3.13E-07 |
| cg25824462 |  | 6.87E-06 | 1.70E-09 |
| cg26262644 |  | 2.48E-05 | 5.75E-08 |
| cg26277237 | KANK1 | 3.32E-03 | 3.65E-07 |
| cg26416615 | ARID5B | 6.63E-04 | 9.91E-07 |
| cg26562462 | TBC1D14 | 4.94E-03 | 2.09E-08 |
| cg26715273 |  | 4.94E-03 | 4.05E-09 |
| cg26916621 | MIR10A | 1.57E-04 | 2.59E-08 |

# **Supplementary table 13. The estimated blood cells ratio in each sample.**

| Sample | B cell | NK cell | CD4+ T cell | CD8+ T cell | Monocyte | Neutrophile | Eosinophile |
| --- | --- | --- | --- | --- | --- | --- | --- |
| 1-1 | 0.00% | 2.97% | 0.00% | 6.80% | 4.97% | 85.26% | 0.00% |
| 1-2 | 0.08% | 3.21% | 2.24% | 7.69% | 6.14% | 80.63% | 0.00% |
| 1-3 | 0.72% | 0.51% | 0.32% | 7.00% | 5.30% | 86.16% | 0.00% |
| 1-4 | 3.55% | 8.41% | 6.03% | 11.14% | 3.23% | 67.64% | 0.00% |
| 2-1 | 0.72% | 1.83% | 4.39% | 3.81% | 7.59% | 81.67% | 0.00% |
| 2-2 | 1.90% | 5.01% | 7.03% | 4.73% | 8.67% | 72.67% | 0.00% |
| 2-3 | 0.57% | 0.00% | 4.74% | 6.62% | 3.52% | 84.56% | 0.00% |
| 2-4 | 4.84% | 6.64% | 15.66% | 10.17% | 9.71% | 52.98% | 0.00% |
| 3-1 | 0.00% | 0.09% | 0.45% | 5.47% | 4.36% | 89.62% | 0.00% |
| 3-2 | 0.00% | 0.00% | 0.00% | 8.31% | 4.84% | 86.85% | 0.00% |
| 3-3 | 2.35% | 0.00% | 0.57% | 17.08% | 10.64% | 69.37% | 0.00% |
| 3-4 | 1.97% | 11.24% | 4.19% | 20.84% | 10.07% | 51.68% | 0.00% |
| 4-1 | 0.00% | 0.40% | 0.15% | 5.93% | 8.83% | 84.69% | 0.00% |
| 4-2 | 0.28% | 1.89% | 3.07% | 5.19% | 7.90% | 81.67% | 0.00% |
| 4-3 | 0.41% | 0.00% | 5.38% | 4.53% | 7.69% | 81.98% | 0.00% |
| 4-4 | 2.64% | 6.00% | 8.13% | 7.93% | 7.58% | 67.72% | 0.00% |
| 5-1 | 6.78% | 4.46% | 4.96% | 5.43% | 9.48% | 68.89% | 0.00% |
| 5-2 | 0.64% | 2.37% | 0.70% | 2.67% | 5.48% | 88.14% | 0.00% |
| 5-3 | 0.04% | 0.00% | 0.00% | 3.50% | 5.91% | 90.55% | 0.00% |
| 5-4 | 3.95% | 1.55% | 1.83% | 9.88% | 7.81% | 74.98% | 0.00% |
| 6-1 | 3.25% | 0.00% | 4.02% | 10.57% | 6.75% | 75.40% | 0.00% |
| 6-2 | 0.61% | 0.00% | 0.34% | 7.53% | 6.54% | 84.98% | 0.00% |
| 6-3 | 1.49% | 0.00% | 0.00% | 8.59% | 7.08% | 82.84% | 0.00% |
| 6-4 | 4.28% | 1.26% | 5.56% | 7.10% | 5.70% | 76.10% | 0.00% |
| 7-1 | 4.74% | 7.97% | 9.26% | 9.48% | 7.61% | 60.94% | 0.00% |
| 7-2 | 2.09% | 1.29% | 6.51% | 9.76% | 6.29% | 74.06% | 0.00% |
| 7-3 | 0.22% | 3.41% | 0.00% | 2.32% | 4.53% | 89.52% | 0.00% |
| 7-4 | 2.77% | 4.43% | 6.23% | 10.53% | 4.53% | 71.51% | 0.00% |
| 8-1 | 0.60% | 1.04% | 8.66% | 9.79% | 7.49% | 72.43% | 0.00% |
| 8-2 | 0.52% | 0.00% | 5.18% | 12.12% | 7.45% | 74.74% | 0.00% |
| 8-3 | 1.54% | 1.50% | 9.38% | 6.30% | 6.48% | 74.80% | 0.00% |
| 8-4 | 3.39% | 7.64% | 9.47% | 8.63% | 6.50% | 64.36% | 0.00% |
| 9-1 | 0.25% | 7.45% | 6.12% | 4.59% | 6.44% | 75.15% | 0.00% |
| 9-2 | 0.47% | 4.57% | 3.53% | 2.89% | 5.00% | 83.54% | 0.00% |
| 9-3 | 0.00% | 1.71% | 6.82% | 4.60% | 5.39% | 81.49% | 0.00% |
| 9-4 | 0.00% | 2.44% | 5.22% | 7.12% | 5.43% | 79.81% | 0.00% |
| 10-1 | 0.25% | 3.19% | 5.17% | 5.44% | 4.40% | 81.55% | 0.00% |
| 10-2 | 0.85% | 0.00% | 2.82% | 7.07% | 5.88% | 83.39% | 0.00% |
| 10-3 | 1.60% | 3.30% | 4.29% | 9.50% | 13.80% | 67.51% | 0.00% |
| 10-4 | 2.48% | 1.49% | 5.98% | 10.03% | 7.34% | 72.68% | 0.00% |
