# Supplementary material for: Comparison of DNA Methylation Changes Between the Gestation Period and the After-Delivery State: A Pilot Study of 10 Women
Source: Front Nutr. 2022 May 4;9:829915. doi: 10.3389/fnut.2022.829915 (PMC9116383; doi:10.3389/fnut.2022.829915)
Supplement: Supplementary Table 9 — The identified CpG cites in group 8. [file Table_1.docx]

Supplementary table 9. **The identified CpG cites in group 8.**

| Target ID | p-value | Chromosome | SNP_ID | mean beta value in the 1st trimester | mean beta value in the 2nd trimester | mean beta value in the 3rd trimester | mean beta value in the after-delivery status |
| --- | --- | --- | --- | --- | --- | --- | --- |
| cg17816847 | 1.00E-03 | 6 |  | 0.369352 | 0.349166 | 0.350136 | 0.440468 |
| cg16413677 | 9.99E-04 | 21 |  | 0.491989 | 0.46155 | 0.468924 | 0.55102 |
| cg07721499 | 9.98E-04 | 12 |  | 0.253143 | 0.230716 | 0.228516 | 0.305233 |
| cg12592772 | 9.98E-04 | 12 | rs565067070 | 0.281572 | 0.246122 | 0.238518 | 0.338033 |
| cg15352315 | 9.98E-04 | 2 | rs188337161 | 0.177776 | 0.148053 | 0.155766 | 0.211193 |
| cg04533831 | 9.96E-04 | 14 | rs143607761;rs200045532; rs201664105 | 0.407129 | 0.385582 | 0.383019 | 0.503345 |
| cg01910652 | 9.95E-04 | 1 | rs543354394;rs528560541 | 0.26578 | 0.2391 | 0.233297 | 0.320675 |
| cg08996243 | 9.95E-04 | 6 | rs190982569;rs145404232 | 0.281094 | 0.258427 | 0.250125 | 0.34525 |
| cg10250072 | 9.94E-04 | 5 | rs573656262;rs190944554 | 0.500158 | 0.474678 | 0.467818 | 0.547353 |
| cg08304487 | 9.93E-04 | 11 | rs569371975;rs530429961; rs548544049;rs143249678 | 0.278967 | 0.247925 | 0.242995 | 0.341752 |
| cg13119178 | 9.92E-04 | 1 |  | 0.258369 | 0.235404 | 0.231587 | 0.304212 |
| cg22720822 | 9.92E-04 | 1 | rs145206570;rs74097471; rs545784427;rs562765076 | 0.297493 | 0.27391 | 0.276369 | 0.369596 |
| cg16461781 | 9.91E-04 | 1 | rs566594848 | 0.231925 | 0.202511 | 0.193619 | 0.260904 |
| cg09855037 | 9.91E-04 | 1 | rs183432304;rs186576735; rs150309555;rs560953620 | 0.337655 | 0.313725 | 0.296608 | 0.429073 |
| cg07137108 | 9.90E-04 | 16 | rs553194904;rs573089159 | 0.183232 | 0.158845 | 0.15806 | 0.234631 |
| cg11790624 | 9.89E-04 | 8 | rs2931719;rs563862201 | 0.257003 | 0.229407 | 0.220672 | 0.35708 |
| cg12080952 | 9.89E-04 | 1 |  | 0.225691 | 0.197411 | 0.198392 | 0.28003 |
| cg08812862 | 9.87E-04 | 11 | rs144093481;rs368937798 | 0.305883 | 0.281851 | 0.289274 | 0.368837 |
| cg06903512 | 9.85E-04 | 20 | rs543641082;rs562350307 | 0.265408 | 0.236115 | 0.230474 | 0.32919 |
| cg13508436 | 9.85E-04 | 2 |  | 0.250378 | 0.224802 | 0.222404 | 0.336415 |
| cg11304191 | 9.84E-04 | 8 | rs545993073 | 0.14842 | 0.122793 | 0.126934 | 0.175841 |
| cg02172262 | 9.84E-04 | 12 | rs147348295 | 0.289042 | 0.267071 | 0.250896 | 0.362491 |
| cg17171019 | 9.83E-04 | 13 | rs533129179;rs545693853 | 0.176064 | 0.154157 | 0.159988 | 0.22757 |
| cg17042723 | 9.82E-04 | 2 | rs573717050;rs150497480; rs184238582;rs17750734 | 0.308116 | 0.287389 | 0.284675 | 0.394458 |
| cg19371916 | 9.81E-04 | 1 | rs553334931 | 0.215706 | 0.194892 | 0.195219 | 0.26827 |
| cg18892778 | 9.81E-04 | 7 | rs185061336;rs58825093 | 0.31252 | 0.285889 | 0.283669 | 0.399745 |
| cg16788865 | 9.81E-04 | 12 |  | 0.253518 | 0.223596 | 0.223756 | 0.348828 |
| cg15568049 | 9.78E-04 | 17 | rs16968857;rs114646510 | 0.471734 | 0.44684 | 0.455485 | 0.539436 |
| cg00255093 | 9.78E-04 | 13 | rs573175456;rs535794662; rs555466164;rs146571071 | 0.373084 | 0.343545 | 0.356525 | 0.457901 |
| cg04403684 | 9.77E-04 | 12 |  | 0.284131 | 0.255137 | 0.250914 | 0.376578 |
| cg17734802 | 9.76E-04 | 1 | rs543127146;rs72902425 | 0.26174 | 0.222114 | 0.217812 | 0.323119 |
| cg12698270 | 9.76E-04 | 14 | rs548454436;rs113170017; rs188971373 | 0.303097 | 0.282809 | 0.269289 | 0.36753 |
| cg15987945 | 9.76E-04 | 6 | rs539543194;rs557782154; rs533068049;rs186775899 | 0.277361 | 0.243184 | 0.243304 | 0.339296 |
| cg13407196 | 9.72E-04 | 22 | rs113479582 | 0.296668 | 0.268101 | 0.259228 | 0.378708 |
| cg09655835 | 9.70E-04 | 7 | rs151336879 | 0.237136 | 0.20557 | 0.209091 | 0.290587 |
| cg03471150 | 9.69E-04 | 1 | rs145926764 | 0.390476 | 0.369594 | 0.373111 | 0.467632 |
| cg22486242 | 9.69E-04 | 6 |  | 0.305958 | 0.274238 | 0.262777 | 0.379298 |
| cg13300613 | 9.67E-04 | 15 | rs146845755;rs78944551 | 0.286991 | 0.262493 | 0.256792 | 0.344088 |
| cg09606588 | 9.66E-04 | 7 |  | 0.295004 | 0.26561 | 0.25679 | 0.372561 |
| cg15259746 | 9.65E-04 | 5 | rs185974356;rs10070292; rs553411945 | 0.141159 | 0.120742 | 0.135626 | 0.174955 |
| cg26277620 | 9.65E-04 | 5 | rs139934727;rs11750888; rs565429725 | 0.248069 | 0.21878 | 0.222173 | 0.314356 |
| cg02061820 | 9.62E-04 | 3 | rs369568828 | 0.224955 | 0.191177 | 0.178734 | 0.263575 |
| cg08468449 | 9.62E-04 | 1 | rs554622808 | 0.327558 | 0.30622 | 0.309969 | 0.390188 |
| cg05420679 | 9.62E-04 | 10 | rs537805044 | 0.400784 | 0.37442 | 0.38546 | 0.47706 |
| cg05872923 | 9.61E-04 | 17 |  | 0.181564 | 0.159215 | 0.161718 | 0.257 |
| cg07259245 | 9.61E-04 | 10 | rs11596008 | 0.614701 | 0.593743 | 0.602879 | 0.669745 |
| cg10769442 | 9.60E-04 | 5 | rs536747284;rs13170937; rs576547911;rs537337145 | 0.272117 | 0.235653 | 0.23514 | 0.341759 |
| cg06160853 | 9.59E-04 | 3 |  | 0.219392 | 0.196709 | 0.189747 | 0.299375 |
| cg19073531 | 9.58E-04 | 5 | rs146105560;rs114852385; rs565483962;rs79509998 | 0.197948 | 0.175111 | 0.174156 | 0.240832 |
| cg09197274 | 9.57E-04 | 10 | rs550169203;rs563677176 | 0.261325 | 0.234019 | 0.21435 | 0.325045 |
| cg08084547 | 9.56E-04 | 8 | rs374841821;rs544837758 | 0.178785 | 0.152347 | 0.156863 | 0.206827 |
| cg20907614 | 9.55E-04 | 8 | rs547808524 | 0.491098 | 0.46962 | 0.478457 | 0.582261 |
| cg17582254 | 9.55E-04 | 7 | rs548866615;rs143564897 | 0.23201 | 0.204713 | 0.194575 | 0.285721 |
| cg08077048 | 9.55E-04 | 15 | rs150581350 | 0.61139 | 0.591187 | 0.606659 | 0.63908 |
| cg24408573 | 9.55E-04 | 3 | rs535506060;rs113735882; rs555787853 | 0.228199 | 0.202164 | 0.19006 | 0.300301 |
| cg24761958 | 9.55E-04 | 21 | rs568063379;rs527476073 | 0.223788 | 0.197115 | 0.19129 | 0.312224 |
| cg16639568 | 9.54E-04 | 9 | rs79583720 | 0.246797 | 0.216192 | 0.202771 | 0.297614 |
| cg24096902 | 9.53E-04 | 1 | rs532284470 | 0.18084 | 0.160295 | 0.159552 | 0.236782 |
| cg17839366 | 9.52E-04 | 2 |  | 0.209582 | 0.185231 | 0.186317 | 0.243367 |
| cg03589967 | 9.51E-04 | 2 | rs558132495;rs576588974 | 0.277396 | 0.248027 | 0.253224 | 0.346578 |
| cg15837172 | 9.48E-04 | 20 |  | 0.226101 | 0.193977 | 0.199296 | 0.269265 |
| cg07758947 | 9.48E-04 | 13 | rs534278933 | 0.297327 | 0.266014 | 0.26231 | 0.356862 |
| cg06868432 | 9.48E-04 | 5 | rs556315028 | 0.293135 | 0.258244 | 0.260939 | 0.354829 |
| cg03999208 | 9.48E-04 | 19 | rs533821842;rs547540970 | 0.20518 | 0.175179 | 0.172166 | 0.260063 |
| cg24257119 | 9.47E-04 | 12 | rs75116073 | 0.297488 | 0.250894 | 0.254755 | 0.378894 |
| cg12157761 | 9.45E-04 | 15 | rs534960786;rs558072119; rs577747883 | 0.326899 | 0.30482 | 0.303589 | 0.415036 |
| cg02530879 | 9.44E-04 | 1 |  | 0.32025 | 0.297603 | 0.28092 | 0.387977 |
| cg15635287 | 9.43E-04 | 12 | rs554435990;rs533123674 | 0.673102 | 0.648224 | 0.662278 | 0.702727 |
| cg05753799 | 9.43E-04 | 20 |  | 0.271837 | 0.249461 | 0.241435 | 0.341761 |
| cg24686551 | 9.42E-04 | 3 | rs565796069 | 0.255431 | 0.231111 | 0.219593 | 0.315108 |
| cg23852001 | 9.41E-04 | X |  | 0.350264 | 0.3246 | 0.316554 | 0.308186 |
| cg05028260 | 9.41E-04 | 20 |  | 0.547536 | 0.519957 | 0.537231 | 0.623386 |
| cg00874051 | 9.41E-04 | 21 | rs5843644 | 0.210305 | 0.181731 | 0.185331 | 0.280173 |
| cg04249873 | 9.41E-04 | 16 | rs149601213;rs563549339; rs117337883 | 0.30466 | 0.279503 | 0.2616 | 0.374009 |
| cg17476271 | 9.40E-04 | 11 |  | 0.16296 | 0.142722 | 0.143543 | 0.192676 |
| cg10750832 | 9.37E-04 | 17 | rs140473989 | 0.292222 | 0.26021 | 0.257096 | 0.344192 |
| cg17667573 | 9.36E-04 | 8 | rs142681665;rs72729584; rs189237174;rs549056991 | 0.495689 | 0.473104 | 0.483351 | 0.570738 |
| cg01053551 | 9.36E-04 | 2 | rs535355256;rs143210360; rs568643340;rs145466643; rs148573943 | 0.291668 | 0.263075 | 0.258417 | 0.357465 |
| cg23390360 | 9.36E-04 | 11 | rs553853347;rs144035420; rs180907024;rs141623893; rs549280561 | 0.382767 | 0.355102 | 0.353241 | 0.453056 |
| cg11744817 | 9.35E-04 | 6 | rs569793631;rs537183929; rs556290571 | 0.22005 | 0.188385 | 0.187919 | 0.250141 |
| cg02395016 | 9.35E-04 | 6 | rs13193634 | 0.441922 | 0.410058 | 0.427297 | 0.507196 |
| cg24719666 | 9.33E-04 | 6 | rs570540776;rs528329519 | 0.648667 | 0.627838 | 0.644899 | 0.697637 |
| cg16139316 | 9.33E-04 | 1 | rs199809903;rs141169559; rs112531265;rs140601192 | 0.245587 | 0.21682 | 0.221149 | 0.325034 |
| cg08001909 | 9.32E-04 | 2 | rs540543021 | 0.205559 | 0.180094 | 0.182546 | 0.275952 |
| cg04675844 | 9.31E-04 | 8 |  | 0.336154 | 0.299285 | 0.29184 | 0.422827 |
| cg08575894 | 9.31E-04 | 11 | rs537265975;rs182872997 | 0.777462 | 0.754199 | 0.766108 | 0.734537 |
| cg02508770 | 9.30E-04 | 16 | rs556198559;rs577670757; rs55995425;rs56064937; rs146223434 | 0.455031 | 0.428033 | 0.43729 | 0.500848 |
| cg12020957 | 9.30E-04 | 3 | rs183057377;rs185898457; rs564417277;rs190417016; rs549862422 | 0.339341 | 0.317066 | 0.307144 | 0.407638 |
| cg07080440 | 9.30E-04 | 19 | rs623700;rs139581608; rs539806780;rs546991183; rs377445721 | 0.300719 | 0.275246 | 0.260058 | 0.359207 |
| cg21341487 | 9.29E-04 | 20 | rs532687565;rs547649491; rs565942875 | 0.31659 | 0.273098 | 0.27578 | 0.366099 |
| cg02509011 | 9.28E-04 | 4 | rs533723653;rs1027984; rs570208677;rs145384504 | 0.390901 | 0.369201 | 0.366655 | 0.504644 |
| cg19939925 | 9.28E-04 | 14 |  | 0.176799 | 0.149035 | 0.15091 | 0.221545 |
| cg11945474 | 9.27E-04 | 2 | rs138443474 | 0.408612 | 0.381377 | 0.392766 | 0.47438 |
| cg11083848 | 9.27E-04 | 14 | rs527529097;rs4903352; rs557807503 | 0.262003 | 0.22754 | 0.22939 | 0.339843 |
| cg25179011 | 9.27E-04 | 18 | rs577942687 | 0.230686 | 0.207723 | 0.197193 | 0.281559 |
| cg15350233 | 9.27E-04 | 19 | rs142155110;rs528175526 | 0.283127 | 0.262847 | 0.251083 | 0.328412 |
| cg22732167 | 9.26E-04 | 18 | rs55938405;rs75080227; rs569307025 | 0.471736 | 0.448816 | 0.442213 | 0.531715 |
| cg06298740 | 9.26E-04 | 1 |  | 0.29437 | 0.26113 | 0.259831 | 0.36972 |
| cg21293813 | 9.26E-04 | 4 | rs151175968;rs561754429; rs140060743;rs150306381 | 0.371136 | 0.326458 | 0.320414 | 0.445134 |
| cg26898932 | 9.22E-04 | 17 | rs75589684;rs549248748 | 0.318269 | 0.289241 | 0.276264 | 0.423091 |
| cg17090611 | 9.19E-04 | 8 | rs137890748 | 0.326698 | 0.282444 | 0.267454 | 0.403321 |
| cg12693060 | 9.18E-04 | 1 | rs541929963 | 0.27182 | 0.238619 | 0.219406 | 0.332349 |
| cg06662488 | 9.15E-04 | 5 | rs78278034;rs116577445 | 0.19824 | 0.176492 | 0.17075 | 0.259805 |
| cg06418013 | 9.14E-04 | 2 | rs565793977;rs536598315 | 0.341859 | 0.311801 | 0.304266 | 0.423635 |
| cg25173269 | 9.14E-04 | 15 | rs561506684;rs570751751; rs138604070 | 0.184931 | 0.160584 | 0.1644 | 0.231078 |
| cg09953946 | 9.13E-04 | 7 | rs547329514 | 0.312535 | 0.279438 | 0.270496 | 0.39143 |
| cg17709911 | 9.13E-04 | 3 | rs549538983 | 0.294809 | 0.260964 | 0.259671 | 0.37857 |
| cg11752100 | 9.12E-04 | 7 | rs141805403 | 0.165582 | 0.144799 | 0.14057 | 0.189946 |
| cg00169401 | 9.11E-04 | 1 | rs147261968 | 0.220418 | 0.187199 | 0.185224 | 0.268243 |
| cg17326611 | 9.10E-04 | 14 | rs532606959;rs116448890; rs140847392 | 0.335788 | 0.311582 | 0.321483 | 0.424162 |
| cg06092168 | 9.10E-04 | 13 |  | 0.324548 | 0.298053 | 0.288071 | 0.407533 |
| cg12203985 | 9.09E-04 | 2 | rs185415641;rs538970487; rs201964322;rs138410566 | 0.452321 | 0.431103 | 0.426487 | NA |
| cg02769451 | 9.09E-04 | 1 | rs138390862;rs147189398; rs142917322;rs148404744; rs530204751 | 0.246195 | 0.21693 | 0.212752 | 0.337856 |
| cg17034901 | 9.09E-04 | 3 | rs546614672;rs192702934; rs532259928;rs185037505; rs371270748 | 0.208477 | 0.18732 | 0.183822 | 0.267878 |
| cg04998379 | 9.09E-04 | 4 | rs542977090;rs565547427; rs534252788 | 0.17026 | 0.148469 | 0.149449 | 0.199634 |
| cg16449084 | 9.09E-04 | 11 | rs369386211;rs538601004; rs557316920 | 0.300942 | 0.27075 | 0.267917 | 0.38015 |
| cg15125801 | 9.08E-04 | 12 | rs546155147;rs564357549; rs531747382 | 0.496869 | 0.474742 | 0.489658 | 0.567424 |
| cg12315900 | 9.07E-04 | 12 | rs141719612;rs150144144 | 0.258079 | 0.219791 | 0.214685 | 0.301813 |
| cg22581364 | 9.07E-04 | 7 | rs546278413 | 0.233488 | 0.203317 | 0.203693 | 0.295956 |
| cg23204699 | 9.06E-04 | 12 | rs559206897 | 0.249508 | 0.226671 | 0.215275 | 0.330947 |
| cg18926797 | 9.06E-04 | 14 |  | 0.32374 | 0.292873 | 0.304078 | 0.409008 |
| cg10807988 | 9.04E-04 | 15 | rs189657932;rs561142833 | 0.266831 | 0.231351 | 0.232162 | 0.354748 |
| cg21590352 | 9.04E-04 | 11 | rs535811431;rs554151428 | 0.362965 | 0.340414 | 0.339315 | 0.433978 |
| cg20829834 | 9.03E-04 | 3 | rs148458717;rs530395594 | 0.275698 | 0.251576 | 0.240568 | 0.349922 |
| cg13523125 | 9.03E-04 | 11 | rs529875980;rs549714738 | 0.23619 | 0.209756 | 0.207918 | 0.297468 |
| cg20090157 | 9.02E-04 | 17 | rs558740053;rs534976601; rs9909357 | 0.573933 | 0.53396 | 0.552126 | 0.633919 |
| cg07470503 | 9.01E-04 | 2 |  | 0.288626 | 0.26337 | 0.260096 | 0.360978 |
| cg04695882 | 9.01E-04 | 16 |  | 0.212192 | 0.1855 | 0.191766 | 0.274352 |
| cg17511274 | 9.01E-04 | 18 | rs143059616 | 0.260513 | 0.226124 | 0.222024 | 0.336762 |
| cg10000705 | 9.01E-04 | 1 | rs149507818 | 0.186606 | 0.165438 | 0.163118 | 0.25938 |
| cg13304825 | 9.00E-04 | 17 | rs62072874;rs572352679 | 0.346953 | 0.32191 | 0.312222 | 0.404644 |
| cg05927518 | 8.98E-04 | 8 | rs149836912 | 0.425385 | 0.400076 | 0.41359 | 0.488117 |
| cg17331920 | 8.98E-04 | 17 | rs17176393 | 0.364783 | 0.314379 | 0.328154 | 0.444426 |
| cg11325152 | 8.97E-04 | 7 | rs191434160 | 0.243518 | 0.209271 | 0.216128 | 0.298519 |
| cg07133930 | 8.96E-04 | 10 | rs533486956;rs546982801 | 0.230992 | 0.201574 | 0.202468 | 0.290919 |
| cg11420606 | 8.95E-04 | 16 | rs548686345;rs374287616; rs537083231 | 0.214291 | 0.178623 | 0.181028 | 0.276638 |
| cg05490745 | 8.94E-04 | 4 | rs150066610 | 0.28556 | 0.252814 | 0.243028 | 0.341538 |
| cg23818106 | 8.93E-04 | 17 | rs376852768;rs374080562; rs548290908;rs531623714 | 0.340699 | 0.316888 | 0.306021 | 0.397783 |
| cg21779122 | 8.92E-04 | 16 | rs116018081;rs189199046; rs553435357;rs74563708; rs80251194;rs12929017; rs12599369;rs140665429 | 0.287052 | 0.258285 | 0.252487 | 0.352728 |
| cg21905127 | 8.92E-04 | 6 | rs534988395 | 0.328191 | 0.294675 | 0.311072 | 0.412535 |
| cg13424393 | 8.91E-04 | 17 | rs190024333;rs550997484; rs569250620 | 0.310501 | 0.286862 | 0.279744 | 0.400675 |
| cg07867876 | 8.89E-04 | 5 |  | 0.214293 | 0.187917 | 0.181182 | 0.259654 |
| cg05957749 | 8.88E-04 | 1 | rs539854403;rs187768237; rs78977029 | 0.455615 | 0.435506 | 0.444955 | 0.507558 |
| cg26298914 | 8.86E-04 | 14 | rs551985987;rs183725904 | 0.312565 | 0.270022 | 0.266608 | 0.382441 |
| cg13823627 | 8.85E-04 | 2 | rs569637584;rs183309095 | 0.276256 | 0.232327 | 0.23602 | 0.347501 |
| cg20794855 | 8.83E-04 | 8 | rs575845839 | 0.338686 | 0.31731 | 0.306439 | 0.399039 |
| cg04835334 | 8.83E-04 | 22 | rs551821056;rs570133959; rs181812012 | 0.243673 | 0.214681 | 0.215365 | 0.322716 |
| cg13017848 | 8.82E-04 | 1 | rs566291658;rs141951051 | 0.392883 | 0.367872 | 0.374 | 0.47531 |
| cg26938302 | 8.82E-04 | 12 | rs17808651 | 0.320171 | 0.279211 | 0.288931 | 0.400784 |
| cg13972318 | 8.81E-04 | 3 | rs148335614;rs71327061; rs569457834 | 0.201133 | 0.171925 | 0.173211 | 0.262534 |
| cg06349838 | 8.80E-04 | 15 | rs28396088;rs566210113; rs141954256;rs548635410; rs150676483 | 0.200402 | 0.177007 | 0.167329 | 0.249597 |
| cg13394762 | 8.79E-04 | 14 | rs200657300;rs575340739; rs181004032;rs200852191 | 0.299635 | 0.267102 | 0.255616 | 0.38573 |
| cg06275503 | 8.79E-04 | 11 | rs113903603;rs193247963 | 0.274391 | 0.250334 | 0.240007 | 0.349241 |
| cg07372938 | 8.78E-04 | 7 | rs142248789 | 0.377262 | 0.352291 | 0.346877 | 0.472355 |
| cg19248536 | 8.77E-04 | 2 | rs188733350;rs536895096; rs116403862 | 0.702242 | 0.679426 | 0.699064 | 0.733605 |
| cg08636882 | 8.77E-04 | 2 | rs144933074;rs534987558; rs182106778;rs577921582; rs147946883;rs570965438 | 0.271221 | 0.245448 | 0.239603 | 0.349252 |
| cg08040565 | 8.76E-04 | 10 | rs538979717;rs557595055; rs140624564 | 0.274489 | 0.245884 | 0.240552 | 0.347164 |
| cg12149081 | 8.75E-04 | 2 | rs572188382;rs541204351 | 0.288919 | 0.26092 | 0.258925 | 0.346109 |
| cg18568335 | 8.74E-04 | 14 |  | 0.303595 | 0.272651 | 0.264905 | 0.367634 |
| cg14000467 | 8.73E-04 | 19 | rs564987225;rs532647995; rs186452259;rs566234036 | 0.710958 | 0.690568 | 0.689548 | 0.765258 |
| cg11225405 | 8.72E-04 | 1 | rs12025416;rs72697331 | 0.334483 | 0.310624 | 0.309218 | 0.399778 |
| cg15188623 | 8.72E-04 | 15 | rs549064542;rs567298340 | 0.22988 | 0.199489 | 0.183167 | 0.290339 |
| cg01639416 | 8.72E-04 | 9 | rs139574808;rs538365917; rs142903858;rs559073871 | 0.388576 | 0.367217 | 0.369117 | 0.46732 |
| cg20721433 | 8.71E-04 | 19 | rs191170949 | 0.220567 | 0.199079 | 0.194894 | 0.283481 |
| cg01271812 | 8.70E-04 | 2 | rs117775419;rs564514285 | 0.256022 | 0.221039 | 0.230883 | 0.296415 |
| cg07001359 | 8.68E-04 | 19 |  | 0.262618 | 0.229557 | 0.222026 | 0.298384 |
| cg04202620 | 8.68E-04 | 8 | rs112424374;rs563442265; rs532075001;rs546477040 | 0.354731 | 0.329517 | 0.327926 | 0.426602 |
| cg08253164 | 8.67E-04 | 12 | rs548238529;rs567990452 | 0.157066 | 0.13638 | 0.142968 | 0.21808 |
| cg12789173 | 8.67E-04 | 11 | rs536850463;rs139626764 | 0.315029 | 0.293415 | 0.309232 | 0.395855 |
| cg15005248 | 8.66E-04 | 4 | rs561802877;rs575392013; rs544466705 | 0.29037 | 0.239393 | 0.238936 | 0.352589 |
| cg06906406 | 8.66E-04 | 2 | rs547091192 | 0.161931 | 0.141216 | 0.134507 | 0.191507 |
| cg23454282 | 8.65E-04 | 11 | rs540580437;rs560625460 | 0.308048 | 0.286081 | 0.281084 | 0.374112 |
| cg21908805 | 8.65E-04 | 8 | rs138206469 | 0.294295 | 0.269868 | 0.262914 | 0.356931 |
| cg04407063 | 8.64E-04 | 11 | rs577287986;rs544591713 | 0.303238 | 0.270058 | 0.271237 | 0.367906 |
| cg20626587 | 8.64E-04 | 20 | rs542945764;rs562829750; rs576528385 | 0.377871 | 0.347726 | 0.338422 | 0.417243 |
| cg03669245 | 8.63E-04 | 5 |  | 0.45022 | 0.414192 | 0.399054 | 0.502645 |
| cg05200313 | 8.63E-04 | 14 | rs186010698 | 0.241887 | 0.21824 | 0.210489 | 0.29908 |
| cg06506094 | 8.61E-04 | 7 | rs576262586;rs191191640; rs142506413 | 0.283016 | 0.238556 | 0.248941 | 0.371837 |
| cg03278707 | 8.59E-04 | 5 | rs541974101;rs189620734 | 0.351922 | 0.317171 | 0.329256 | 0.444524 |
| cg06943590 | 8.59E-04 | 12 | rs191334991 | 0.284683 | 0.259375 | 0.249851 | 0.366589 |
| cg02913768 | 8.56E-04 | 3 | rs13325350;rs377511849; rs116700358 | 0.241486 | 0.214434 | 0.221218 | 0.323865 |
| cg06894709 | 8.55E-04 | 1 | rs111940062;rs530979685 | 0.261014 | 0.239217 | 0.239293 | 0.320814 |
| cg07276518 | 8.53E-04 | 1 | rs572718047;rs71567057; rs541640481 | 0.326626 | 0.30661 | 0.297859 | 0.401502 |
| cg22284398 | 8.51E-04 | 2 | rs112327713;rs555752021; rs184716484;rs10199068; rs557927718 | 0.234347 | 0.205703 | 0.204517 | 0.273833 |
| cg25363080 | 8.51E-04 | 5 | rs553023924 | 0.230744 | 0.209632 | 0.200249 | 0.281942 |
| cg12182708 | 8.50E-04 | 10 | rs554579813;rs141369833 | 0.29271 | 0.263433 | 0.25782 | 0.359478 |
| cg00907842 | 8.50E-04 | 5 | rs545334439;rs73072525; rs114227674 | 0.221436 | 0.198849 | 0.186956 | 0.280957 |
| cg03938562 | 8.49E-04 | 8 | rs557169502;rs147853567 | 0.422718 | 0.400291 | 0.384392 | 0.476189 |
| cg18855165 | 8.48E-04 | 9 | rs113475100;rs576759616; rs541003738 | 0.248832 | 0.219546 | 0.221581 | 0.328236 |
| cg09088633 | 8.47E-04 | 1 |  | 0.306813 | 0.281373 | 0.276419 | 0.390409 |
| cg24827600 | 8.47E-04 | 2 | rs114388308;rs536802013; rs141098689;rs146939491 | 0.328075 | 0.303289 | 0.297204 | 0.414481 |
| cg17407476 | 8.47E-04 | 2 | rs75658717;rs150646849 | 0.317929 | 0.279941 | 0.278824 | 0.406325 |
| cg06305891 | 8.47E-04 | 3 | rs530823301;rs185550954 | 0.287556 | 0.262622 | 0.255363 | 0.34099 |
| cg18233010 | 8.46E-04 | 10 | rs536661361 | 0.181774 | 0.160154 | 0.1555 | 0.235226 |
| cg06637170 | 8.45E-04 | 11 | rs527821442;rs552680727 | 0.26994 | 0.245511 | 0.245038 | 0.327791 |
| cg19279310 | 8.45E-04 | 11 | rs111698821 | 0.331084 | 0.30968 | 0.315332 | 0.37496 |
| cg00740718 | 8.45E-04 | 14 | rs527988858 | 0.244489 | 0.206271 | 0.218294 | 0.309492 |
| cg16729467 | 8.44E-04 | 3 | rs181352847;rs559455154; rs528273025 | 0.339196 | 0.317212 | 0.315239 | 0.415979 |
| cg13661827 | 8.43E-04 | 9 | rs540126490;rs558167215 | 0.322716 | 0.300865 | 0.305177 | 0.399886 |
| cg10239545 | 8.43E-04 | 20 | rs4813659 | 0.227717 | 0.198447 | 0.195318 | 0.2711 |
| cg21511321 | 8.42E-04 | 7 | rs539690949;rs10282027 | 0.279152 | 0.254022 | 0.25264 | 0.334578 |
| cg25557698 | 8.42E-04 | 6 | rs537780284 | 0.331575 | 0.297991 | 0.28121 | 0.389239 |
| cg02322400 | 8.42E-04 | 11 | rs373331753;rs533267959; rs115419411;rs142462689 | 0.174142 | 0.149768 | 0.168735 | 0.229479 |
| cg22666015 | 8.42E-04 | 2 | rs529216086 | 0.235665 | 0.213452 | 0.202005 | 0.301721 |
| cg17117981 | 8.41E-04 | 8 | rs577832022;rs549709892; rs35643226;rs572078674 | 0.515915 | 0.486157 | 0.474257 | 0.574965 |
| cg21027187 | 8.41E-04 | 15 | rs550095626;rs2445782 | 0.408613 | 0.388295 | 0.390618 | 0.485966 |
| cg26823930 | 8.40E-04 | 16 | rs560023705;rs56109774; rs542903627 | 0.28939 | 0.256106 | 0.248383 | 0.373144 |
| cg08848776 | 8.39E-04 | 19 | rs184981005;rs568783359 | 0.220291 | 0.193362 | 0.198064 | 0.282046 |
| cg17952474 | 8.37E-04 | 21 | rs146031935;rs13051086 | 0.266504 | 0.240807 | 0.230834 | 0.355033 |
| cg17161591 | 8.37E-04 | 11 | rs190239707 | 0.223903 | 0.199179 | 0.193076 | 0.288318 |
| cg17209390 | 8.37E-04 | 19 | rs4803493;rs534423664; rs185242117;rs73931745 | 0.331714 | 0.297558 | 0.287353 | 0.389175 |
| cg19453003 | 8.36E-04 | 21 | rs530855841 | 0.348283 | 0.316988 | 0.319306 | 0.45658 |
| cg15235987 | 8.36E-04 | 12 | rs572923829;rs144659960 | 0.273807 | 0.243539 | 0.228592 | 0.32859 |
| cg26337227 | 8.36E-04 | 2 | rs564516752;rs531725553 | 0.258357 | 0.234123 | 0.235946 | 0.339051 |
| cg00512674 | 8.35E-04 | 2 | rs563153067;rs572683912 | 0.335487 | 0.308823 | 0.316159 | 0.422149 |
| cg08441877 | 8.34E-04 | 10 | rs75351022;rs533275003 | 0.468334 | 0.434088 | 0.443636 | 0.564227 |
| cg19913861 | 8.33E-04 | 11 | rs543337734;rs561983142 | 0.291654 | 0.254846 | 0.24611 | 0.362107 |
| cg11881280 | 8.32E-04 | 15 | rs546387434;rs8042222; rs77811966;rs552093092; rs189374905 | 0.378439 | 0.350794 | 0.360575 | 0.451881 |
| cg06810647 | 8.31E-04 | 16 | rs2301458;rs527826310 | 0.176998 | 0.153258 | 0.163277 | 0.212845 |
| cg23251286 | 8.31E-04 | 20 | rs79170211 | 0.298594 | 0.268051 | 0.269239 | 0.407576 |
| cg04212786 | 8.30E-04 | 4 | rs146786122 | 0.353772 | 0.319375 | 0.316272 | 0.417614 |
| cg22712983 | 8.30E-04 | 2 | rs577748225 | 0.573863 | 0.494745 | 0.507525 | 0.664628 |
| cg21361368 | 8.29E-04 | 8 | rs28999672;rs145638912 | 0.32622 | 0.282185 | 0.281499 | 0.400495 |
| cg21553977 | 8.29E-04 | 6 | rs569474467;rs138911243 | 0.293211 | 0.269602 | 0.256091 | 0.347013 |
| cg19817104 | 8.27E-04 | 1 | rs144401368;rs148602312; rs555001525 | 0.233217 | 0.21246 | 0.222762 | 0.288411 |
| cg03934069 | 8.27E-04 | 1 | rs548933070 | 0.146081 | 0.120714 | 0.130433 | 0.173818 |
| cg13509638 | 8.26E-04 | 1 | rs527384440;rs547607134; rs186581022 | 0.321083 | 0.297981 | 0.291805 | 0.377937 |
| cg01351337 | 8.26E-04 | 10 | rs61873598;rs553452331; rs145777885;rs185009313; rs76568221 | 0.26251 | 0.226936 | 0.218614 | 0.340902 |
| cg03831756 | 8.26E-04 | 22 |  | 0.337736 | 0.315192 | 0.312155 | 0.422865 |
| cg25600606 | 8.25E-04 | 11 | rs187022690;rs551845395 | 0.373431 | 0.339661 | 0.345903 | 0.463261 |
| cg00025063 | 8.24E-04 | 5 | rs142117251 | 0.428566 | 0.404671 | 0.39715 | 0.502406 |
| cg08279546 | 8.24E-04 | 17 | rs185040613;rs555319835 | 0.537004 | 0.512219 | 0.526993 | 0.588582 |
| cg03800252 | 8.23E-04 | 12 | rs555893969 | 0.623853 | 0.6009 | 0.616851 | 0.687629 |
| cg12453623 | 8.23E-04 | 2 |  | 0.449921 | 0.426807 | 0.433699 | 0.516899 |
| cg13999433 | 8.22E-04 | 9 | rs113947204 | 0.300566 | 0.274451 | 0.263702 | 0.385639 |
| cg24504014 | 8.22E-04 | 11 | rs541516579 | 0.344451 | 0.321405 | 0.324098 | 0.449249 |
| cg12313149 | 8.20E-04 | 2 | rs541434710 | 0.310409 | 0.271562 | 0.277732 | 0.387123 |
| cg09874335 | 8.20E-04 | 10 | rs567272344;rs144985487; rs138906037 | 0.32811 | 0.290069 | 0.290445 | 0.389822 |
| cg04172660 | 8.18E-04 | 17 | rs144810783;rs75670228; rs369806898 | 0.329329 | 0.303773 | 0.312013 | 0.393131 |
| cg01210663 | 8.17E-04 | 9 | rs190622191 | 0.134296 | 0.110732 | 0.115993 | 0.158153 |
| cg21285555 | 8.17E-04 | 8 |  | 0.338381 | 0.31318 | 0.313106 | 0.407861 |
| cg07470763 | 8.17E-04 | 18 |  | 0.37889 | 0.352376 | 0.354912 | 0.444316 |
| cg23317319 | 8.15E-04 | 2 | rs75332799;rs4852383; rs4852384 | 0.306741 | 0.273795 | 0.25735 | 0.375126 |
| cg05750782 | 8.13E-04 | 6 | rs181565519;rs572936164 | 0.312988 | 0.286707 | 0.286055 | 0.415414 |
| cg01950511 | 8.13E-04 | 2 | rs144143431 | 0.283027 | 0.24943 | 0.245579 | 0.356999 |
| cg10231978 | 8.13E-04 | 1 | rs569383844;rs72677646; rs558070674 | 0.291869 | 0.260396 | 0.265874 | 0.382262 |
| cg02538681 | 8.12E-04 | 10 | rs142112176 | 0.341894 | 0.31256 | 0.321726 | 0.427033 |
| cg25010177 | 8.12E-04 | 10 | rs549782873;rs181766176; rs532196848 | 0.300828 | 0.26161 | 0.258458 | 0.350655 |
| cg10777631 | 8.11E-04 | 5 | rs139484997;rs187999804; rs142781518;rs566832831 | 0.152205 | 0.12919 | 0.129005 | 0.177134 |
| cg18169682 | 8.11E-04 | 3 | rs142165356;rs557334809; rs567527358 | 0.389806 | 0.356666 | 0.359883 | 0.487638 |
| cg09298190 | 8.10E-04 | 5 |  | 0.253974 | 0.227654 | 0.220872 | 0.307938 |
| cg12339328 | 8.09E-04 | 20 | rs190721221;rs139616572; rs121918222 | 0.361566 | 0.335724 | 0.330958 | 0.443124 |
| cg24310530 | 8.09E-04 | 11 | rs572303034 | 0.353188 | 0.327543 | 0.309214 | 0.451686 |
| cg07472373 | 8.08E-04 | 10 | rs536625927;rs554911449 | 0.313224 | 0.276473 | 0.266664 | 0.387169 |
| cg18222653 | 8.08E-04 | 1 | rs555717360 | 0.347945 | 0.327344 | 0.327539 | 0.435042 |
| cg04543224 | 8.07E-04 | 19 | rs187053519;rs28378712 | 0.25516 | 0.232208 | 0.213602 | 0.301432 |
| cg14352263 | 8.06E-04 | 3 | rs113645169;rs574265547 | 0.260843 | 0.235211 | 0.237101 | 0.329935 |
| cg02142483 | 8.04E-04 | 16 | rs149994756;rs144286255; rs558506975;rs572442937; rs189337099;rs73251466 | 0.300916 | 0.263387 | 0.263423 | 0.376262 |
| cg15792938 | 8.04E-04 | 4 | rs554022246;rs573761137; rs536378622 | 0.285642 | 0.260095 | 0.262723 | 0.343786 |
| cg01723892 | 8.03E-04 | 1 | rs374412946 | 0.249912 | 0.221073 | 0.214106 | 0.30959 |
| cg15252445 | 8.03E-04 | 12 | rs149549340 | 0.204006 | 0.18218 | 0.174271 | 0.26316 |
| cg01594740 | 8.02E-04 | 22 |  | 0.28929 | 0.262411 | 0.265038 | 0.351563 |
| cg09288789 | 8.01E-04 | 6 | rs191168595 | 0.158424 | 0.130908 | 0.148685 | 0.210798 |
| cg20636248 | 8.00E-04 | 10 | rs192539345 | 0.321825 | 0.297878 | 0.29405 | 0.393593 |
| cg19245151 | 7.99E-04 | 5 | rs553485308 | 0.252792 | 0.227976 | 0.225704 | 0.296724 |
| cg07659375 | 7.99E-04 | 17 | rs566200722;rs540140571; rs28583687 | 0.326412 | 0.296361 | 0.297737 | 0.381969 |
| cg01487368 | 7.98E-04 | 12 |  | 0.26843 | 0.235643 | 0.233158 | 0.349763 |
| cg22076474 | 7.98E-04 | 21 |  | 0.273919 | 0.246317 | 0.2558 | 0.376037 |
| cg09042678 | 7.98E-04 | 20 | rs560648937 | 0.253438 | 0.219489 | 0.211669 | 0.302892 |
| cg06926882 | 7.98E-04 | 5 |  | 0.205226 | 0.178269 | 0.165452 | 0.263367 |
| cg06813706 | 7.98E-04 | 2 | rs576403848 | 0.33306 | 0.309315 | 0.303572 | 0.419961 |
| cg24504349 | 7.97E-04 | 4 | rs554878971;rs574812742; rs543573181 | 0.296445 | 0.252132 | 0.241909 | NA |
| cg19730560 | 7.96E-04 | 3 | rs546488299;rs4855623; rs149466398 | 0.377352 | 0.350444 | 0.358159 | 0.440659 |
| cg00771685 | 7.95E-04 | 22 |  | 0.367167 | 0.346593 | 0.33941 | 0.431724 |
| cg07497120 | 7.94E-04 | 9 |  | 0.254487 | 0.232633 | 0.240911 | 0.314464 |
| cg19379599 | 7.94E-04 | 21 | rs2836219 | 0.370952 | 0.343219 | 0.340941 | 0.45479 |
| cg20752509 | 7.94E-04 | 3 | rs192831845;rs148403244; rs77937342 | 0.275116 | 0.239234 | 0.234335 | 0.342716 |
| cg11763797 | 7.94E-04 | 17 | rs552313073;rs16965775 | 0.324992 | 0.299028 | 0.289111 | 0.397724 |
| cg17344321 | 7.91E-04 | 7 | rs10265151;rs531084574; rs549700712 | 0.209686 | 0.187413 | 0.184836 | 0.253189 |
| cg24427660 | 7.91E-04 | 11 | rs548638618;rs565743944; rs528106473 | 0.309509 | 0.282768 | 0.283984 | 0.366248 |
| cg26132071 | 7.90E-04 | 18 | rs148744846;rs142383033 | 0.280213 | 0.241405 | 0.247169 | 0.353679 |
| cg01582683 | 7.90E-04 | 1 |  | 0.274052 | 0.244445 | 0.236392 | 0.34446 |
| cg24178686 | 7.89E-04 | 2 | rs143363439 | 0.300577 | 0.271852 | 0.265467 | 0.391927 |
| cg17941843 | 7.88E-04 | 2 | rs534230584;rs142141803; rs113799704;rs574287183 | 0.394754 | 0.350755 | 0.359915 | 0.495137 |
| cg12468966 | 7.87E-04 | 6 | rs182121832;rs187506217; rs543202144 | 0.24353 | 0.206441 | 0.207059 | 0.299757 |
| cg13267916 | 7.86E-04 | 2 | rs549921059;rs201797465; rs16859050 | 0.307863 | 0.278018 | 0.272577 | 0.383983 |
| cg09358112 | 7.86E-04 | 2 | rs192950942;rs74573714; rs184166382 | 0.202929 | 0.179512 | 0.176052 | 0.239599 |
| cg16535257 | 7.86E-04 | 1 | rs3131972;rs560420638 | 0.226329 | 0.200977 | 0.196877 | 0.289481 |
| cg01276321 | 7.84E-04 | 6 | rs532877088 | 0.26057 | 0.2346 | 0.239951 | 0.365751 |
| cg17591816 | 7.84E-04 | 1 | rs576961190;rs139664660; rs537020305 | 0.321231 | 0.295848 | 0.292863 | 0.409124 |
| cg09108940 | 7.83E-04 | 2 | rs553835416;rs11675048; rs542457162 | 0.308628 | 0.283729 | 0.280043 | 0.387392 |
| cg17461390 | 7.83E-04 | 19 | rs539240896;rs552898272 | 0.289127 | 0.263631 | 0.259908 | 0.362389 |
| cg15199181 | 7.82E-04 | 1 | rs560254973;rs574276071 | 0.23524 | 0.206351 | 0.213295 | 0.273792 |
| cg02582338 | 7.82E-04 | 14 | rs11624703;rs76516447; rs571328797 | 0.382754 | 0.361655 | 0.380588 | 0.442836 |
| cg03102920 | 7.82E-04 | 6 | rs570957261;rs555265722; rs572112697 | 0.272741 | 0.249714 | 0.235952 | 0.331151 |
| cg14685461 | 7.82E-04 | 3 | rs558568847;rs114447531; rs575612176 | 0.325216 | 0.291285 | 0.299706 | 0.397794 |
| cg12896170 | 7.81E-04 | 6 | rs140475176;rs185488569; rs550986291 | 0.62746 | 0.598503 | 0.611122 | 0.666936 |
| cg00510842 | 7.80E-04 | 12 | rs74059919 | 0.280501 | 0.25104 | 0.239121 | 0.358171 |
| cg14218917 | 7.79E-04 | 2 |  | 0.297021 | 0.266031 | 0.255827 | 0.375009 |
| cg08493063 | 7.79E-04 | 16 | rs79001082 | 0.294968 | 0.268899 | 0.258458 | 0.357664 |
| cg10588962 | 7.78E-04 | 17 | rs570950945;rs538871948 | 0.195809 | 0.174169 | 0.179331 | 0.252998 |
| cg06022862 | 7.78E-04 | 4 | rs571543637 | 0.31802 | 0.28396 | 0.281003 | 0.391362 |
| cg25384702 | 7.78E-04 | 12 | rs558982732;rs569173464; rs538274383;rs554645172; rs574839588 | 0.309162 | 0.275817 | 0.263375 | 0.377464 |
| cg09641213 | 7.78E-04 | 1 | rs534633403 | 0.310838 | 0.284772 | 0.278462 | 0.363018 |
| cg08023927 | 7.77E-04 | 10 | rs111852538;rs555333886 | 0.275852 | 0.254696 | 0.245547 | 0.347864 |
| cg09268449 | 7.77E-04 | 10 | rs562421923 | 0.34951 | 0.323259 | 0.319239 | 0.408989 |
| cg19597640 | 7.77E-04 | 3 | rs139955754;rs575835308; rs111455722 | 0.336223 | 0.313994 | 0.311204 | 0.407593 |
| cg22924721 | 7.74E-04 | 17 | rs184741907;rs965229; rs188625951;rs181400847 | 0.352166 | 0.328173 | 0.325175 | 0.425347 |
| cg10257049 | 7.74E-04 | 5 | rs529312515 | 0.293137 | 0.267837 | 0.265189 | 0.348891 |
| cg10713461 | 7.74E-04 | 17 | rs369108310;rs185620696 | 0.31108 | 0.26461 | 0.263338 | 0.383814 |
| cg02765696 | 7.74E-04 | 5 | rs544015126;rs181717111 | 0.30409 | 0.266972 | 0.262331 | 0.381301 |
| cg23067535 | 7.73E-04 | 8 | rs539364167 | 0.709221 | 0.680631 | 0.688188 | 0.72033 |
| cg26794999 | 7.73E-04 | 3 | rs192882159;rs183541253; rs539650654 | 0.168534 | 0.146296 | 0.152097 | 0.219058 |
| cg11902347 | 7.73E-04 | 17 | rs185620696 | 0.302644 | 0.273302 | 0.256211 | 0.377791 |
| cg21627955 | 7.72E-04 | 13 | rs372337120;rs564486445 | 0.317124 | 0.289537 | 0.296563 | 0.411475 |
| cg09277541 | 7.72E-04 | 21 |  | 0.255036 | 0.22295 | 0.216475 | 0.334664 |
| cg01110839 | 7.72E-04 | 11 | rs536078280;rs375762869; rs566254675 | 0.42725 | 0.404863 | 0.392331 | 0.502366 |
| cg05866193 | 7.70E-04 | 1 | rs191497195 | 0.25752 | 0.232891 | 0.217961 | 0.31816 |
| cg00188327 | 7.70E-04 | 11 | rs550610967;rs4757215 | 0.241194 | 0.206061 | 0.199376 | 0.293949 |
| cg15210205 | 7.69E-04 | 10 |  | 0.528687 | 0.498748 | 0.508706 | 0.602409 |
| cg20745590 | 7.69E-04 | 4 |  | 0.225156 | 0.20255 | 0.20998 | 0.286487 |
| cg00949332 | 7.68E-04 | 8 | rs570298353 | 0.340874 | 0.298619 | 0.307236 | 0.443246 |
| cg08843850 | 7.67E-04 | 1 | rs188171497;rs562830733 | 0.268257 | 0.234218 | 0.234796 | 0.319225 |
| cg18581998 | 7.67E-04 | 9 | rs587740698;rs112767008; rs587696631;rs45489391; rs587650684 | 0.628298 | 0.587598 | 0.588689 | 0.69203 |
| cg18875208 | 7.66E-04 | 1 | rs115345534;rs546870233 | 0.269856 | 0.245212 | 0.25471 | 0.350336 |
| cg08968229 | 7.66E-04 | 12 | rs573726571 | 0.204246 | 0.171657 | 0.179651 | 0.259791 |
| cg21046286 | 7.65E-04 | 19 |  | 0.293812 | 0.262202 | 0.264969 | 0.350553 |
| cg06740950 | 7.65E-04 | 3 |  | 0.239824 | 0.209393 | 0.218197 | 0.320149 |
| cg03726817 | 7.64E-04 | 12 | rs547636815;rs567336556 | 0.20085 | 0.180067 | 0.164706 | 0.236834 |
| cg16552042 | 7.64E-04 | 1 |  | 0.315985 | 0.273578 | 0.269786 | 0.383972 |
| cg01018533 | 7.61E-04 | 1 | rs561622574 | 0.293575 | 0.26806 | 0.261151 | 0.368943 |
| cg06430860 | 7.61E-04 | 3 | rs532099331;rs552284964; rs568683939;rs537664546; rs554400953;rs185140616 | 0.297124 | 0.272604 | 0.267772 | 0.383318 |
| cg18481322 | 7.60E-04 | 12 | rs560906124;rs529663449; rs74736636;rs566439427 | 0.240075 | 0.210724 | 0.210889 | 0.297744 |
| cg02181446 | 7.59E-04 | 4 | rs79502004;rs151102014 | 0.392829 | 0.363533 | 0.377021 | 0.475553 |
| cg09794343 | 7.59E-04 | 16 | rs553819997;rs143747467 | 0.277843 | 0.248159 | 0.244337 | 0.363974 |
| cg06407729 | 7.59E-04 | 11 |  | 0.317275 | 0.296255 | 0.301317 | 0.397252 |
| cg08251386 | 7.59E-04 | 9 | rs562941681;rs530463592 | 0.271282 | 0.251017 | 0.242958 | 0.334252 |
| cg03746015 | 7.58E-04 | 16 | rs532522439 | 0.600155 | 0.553836 | 0.550106 | 0.650246 |
| cg15763706 | 7.57E-04 | 3 | rs555737983 | 0.231371 | 0.205566 | 0.209154 | 0.296417 |
| cg06287548 | 7.57E-04 | 21 | rs568926923 | 0.332122 | 0.305993 | 0.295763 | 0.392041 |
| cg03432697 | 7.56E-04 | 14 |  | 0.24654 | 0.215349 | 0.21726 | 0.309365 |
| cg02098075 | 7.56E-04 | 8 | rs202243723;rs572033911; rs545899149;rs74697155; rs201706002 | 0.294329 | 0.255999 | 0.263219 | 0.390745 |
| cg22388897 | 7.55E-04 | 14 | rs371865358;rs546539229; rs566368310 | 0.307413 | 0.272431 | 0.285384 | 0.372239 |
| cg16708289 | 7.54E-04 | 2 | rs570497891;rs149922804 | 0.323866 | 0.29198 | 0.286899 | 0.381617 |
| cg09948818 | 7.53E-04 | 5 | rs558950796;rs73795967; rs181601981 | 0.363302 | 0.34141 | 0.330881 | 0.428667 |
| cg25161900 | 7.53E-04 | X |  | 0.809935 | 0.788379 | 0.806866 | 0.829591 |
| cg16612625 | 7.53E-04 | 2 | rs562398297;rs577676450; rs149003733;rs559762215; rs564085137 | 0.439784 | 0.412428 | 0.431203 | 0.515495 |
| cg10977731 | 7.52E-04 | 8 | rs542297355;rs73681118 | 0.279265 | 0.254617 | 0.250113 | 0.337385 |
| cg00098559 | 7.51E-04 | 20 | rs189246209;rs192233100; rs6088505;rs543417587 | 0.334187 | 0.299782 | 0.303046 | 0.407461 |
| cg23532832 | 7.51E-04 | 15 | rs563566551;rs8027434; rs543344971;rs144174496; rs528092488;rs146483746 | 0.246244 | 0.218455 | 0.211124 | 0.292952 |
| cg26779692 | 7.50E-04 | 11 | rs12224194;rs200939157; rs145860110;rs148209474 | 0.286741 | 0.265152 | 0.260834 | 0.356719 |
| cg05976544 | 7.50E-04 | 18 | rs573827060;rs541099070; rs117295654;rs78886048 | 0.28716 | 0.264286 | 0.254726 | 0.36642 |
| cg16378249 | 7.50E-04 | 10 | rs113350216;rs529719806 | 0.220854 | 0.200182 | 0.185635 | 0.275271 |
| cg14547461 | 7.47E-04 | 8 | rs570474604;rs375881977; rs549879528;rs536489226; rs535148692;rs116051814 | 0.324873 | 0.299444 | 0.299137 | 0.404004 |
| cg19205426 | 7.47E-04 | 8 | rs75999726;rs141406539 | 0.273999 | 0.252193 | 0.248599 | 0.340071 |
| cg26396370 | 7.46E-04 | 2 | rs192585504 | 0.409017 | 0.377646 | 0.379503 | 0.515476 |
| cg26034770 | 7.46E-04 | 7 |  | 0.225816 | 0.196713 | 0.19189 | 0.296558 |
| cg21556598 | 7.46E-04 | 5 |  | 0.311953 | 0.288511 | 0.276531 | 0.383054 |
| cg25647583 | 7.45E-04 | 15 | rs559959850;rs142687667; rs16944986 | 0.314141 | 0.291488 | 0.294421 | 0.379672 |
| cg04508114 | 7.45E-04 | 10 | rs143992485;rs537986604 | 0.565882 | 0.539777 | 0.558192 | 0.613591 |
| cg15866367 | 7.44E-04 | 13 | rs145245676;rs536339152 | 0.651004 | 0.630184 | 0.649233 | 0.708213 |
| cg05791656 | 7.44E-04 | 2 | rs555889144;rs181072066 | 0.409281 | 0.380544 | 0.390678 | 0.488053 |
| cg26437697 | 7.44E-04 | 14 |  | 0.362329 | 0.340907 | 0.341457 | 0.441611 |
| cg24238409 | 7.43E-04 | 10 | rs12266787 | 0.43415 | 0.406775 | 0.402929 | 0.517201 |
| cg03688134 | 7.43E-04 | 12 | rs551652280 | 0.211365 | 0.186397 | 0.184612 | 0.271807 |
| cg00840770 | 7.43E-04 | 1 | rs138275771;rs144135523 | 0.345567 | 0.312139 | 0.322863 | 0.448018 |
| cg12022722 | 7.43E-04 | 19 | rs530158460 | 0.250142 | 0.222902 | 0.229655 | 0.313463 |
| cg24393958 | 7.42E-04 | 3 | rs139772012;rs73884519; rs192052785;rs559289192 | 0.345806 | 0.313751 | 0.313188 | 0.421721 |
| cg16018533 | 7.41E-04 | 16 | rs189589278;rs544225328; rs182275906 | 0.34833 | 0.325288 | 0.327756 | 0.410285 |
| cg04525852 | 7.40E-04 | 4 | rs193060776 | 0.204359 | 0.182554 | 0.182732 | 0.26727 |
| cg20544006 | 7.40E-04 | 5 | rs549791457 | 0.307805 | 0.278647 | 0.274377 | 0.380519 |
| cg13589461 | 7.40E-04 | 21 | rs551585005 | 0.308137 | 0.286308 | 0.283464 | 0.372874 |
| cg05643579 | 7.40E-04 | 3 | rs561468231 | 0.265691 | 0.234191 | 0.223139 | 0.338309 |
| cg10337975 | 7.39E-04 | 7 |  | 0.31988 | 0.299244 | 0.298805 | 0.379083 |
| cg06875621 | 7.39E-04 | 2 | rs186868216;rs531667887; rs545507921;rs192067509 | 0.287815 | 0.257133 | 0.252535 | 0.346208 |
| cg05962003 | 7.39E-04 | 19 | rs564311657 | 0.631317 | 0.605934 | 0.602874 | 0.67944 |
| cg07481273 | 7.39E-04 | 14 |  | 0.292166 | 0.255488 | 0.250174 | 0.367049 |
| cg02692522 | 7.38E-04 | 18 | rs528742786;rs551447394 | 0.224658 | 0.190495 | 0.180244 | 0.288197 |
| cg09290120 | 7.36E-04 | 2 | rs552326397 | 0.369241 | 0.342342 | 0.348305 | 0.452521 |
| cg00200629 | 7.35E-04 | 9 | rs536266012 | 0.261594 | 0.235138 | 0.239268 | 0.359929 |
| cg08324725 | 7.35E-04 | 6 | rs118169916;rs535236282 | 0.383855 | 0.355724 | 0.36771 | 0.467684 |
| cg24149590 | 7.34E-04 | 14 | rs184360608 | 0.176861 | 0.155046 | 0.157367 | NA |
| cg23419841 | 7.34E-04 | 18 | rs530866369 | 0.192517 | 0.163482 | 0.172481 | 0.25294 |
| cg03337482 | 7.33E-04 | 1 | rs142214021;rs191876875; rs542931721 | 0.453283 | 0.428576 | 0.442145 | 0.498967 |
| cg25007705 | 7.33E-04 | 1 | rs566439867 | 0.240444 | 0.220428 | 0.221666 | 0.291292 |
| cg21602435 | 7.33E-04 | 6 | rs576818576;rs117140721 | 0.218371 | 0.19836 | 0.18802 | 0.273422 |
| cg24520560 | 7.33E-04 | 1 | rs144291611;rs569402980 | 0.255594 | 0.231921 | 0.218154 | 0.328176 |
| cg17889831 | 7.32E-04 | 1 | rs181159956;rs570501535; rs539177515 | 0.251274 | 0.223027 | 0.223018 | 0.307245 |
| cg02504313 | 7.32E-04 | 1 | rs548715070;rs563751624 | 0.304907 | 0.277245 | 0.274967 | 0.362586 |
| cg25814293 | 7.32E-04 | 7 | rs116982397 | 0.549574 | 0.517463 | 0.509763 | 0.651814 |
| cg16648827 | 7.30E-04 | 18 | rs75105296;rs576975572 | 0.261522 | 0.231277 | 0.233211 | 0.316467 |
| cg22664807 | 7.30E-04 | 12 | rs60622848;rs3752398 | 0.204603 | 0.173838 | 0.167127 | 0.247003 |
| cg14985891 | 7.29E-04 | 1 |  | 0.278665 | 0.241964 | 0.234112 | 0.350718 |
| cg16107927 | 7.29E-04 | 18 |  | 0.227968 | 0.203747 | 0.202919 | 0.283331 |
| cg13581015 | 7.29E-04 | 12 | rs551157755 | 0.276589 | 0.248434 | 0.248275 | 0.362694 |
| cg15787128 | 7.28E-04 | 9 | rs532388949;rs560037416 | 0.237422 | 0.210212 | 0.213904 | 0.304211 |
| cg10173751 | 7.28E-04 | 17 | rs147390324;rs564880583 | 0.338338 | 0.299712 | 0.312842 | 0.405934 |
| cg02060682 | 7.28E-04 | 7 |  | 0.433368 | 0.409069 | 0.416126 | 0.532036 |
| cg01819950 | 7.26E-04 | 6 |  | 0.417707 | 0.381495 | 0.393445 | 0.48496 |
| cg10891461 | 7.26E-04 | 5 | rs142452443 | 0.276157 | 0.246285 | 0.24897 | 0.362383 |
| cg25922284 | 7.25E-04 | 8 | rs555309837 | 0.251911 | 0.21514 | 0.214219 | 0.346899 |
| cg17007896 | 7.24E-04 | 11 | rs570327169 | 0.29154 | 0.267242 | 0.257435 | 0.344973 |
| cg06005892 | 7.24E-04 | 11 | rs189674991 | 0.316351 | 0.283711 | 0.279473 | 0.367153 |
| cg20981347 | 7.23E-04 | 18 | rs146937303;rs571135874 | 0.272702 | 0.241567 | 0.229659 | NA |
| cg09084464 | 7.23E-04 | 3 | rs148659606;rs141835839 | 0.251186 | 0.231148 | 0.225066 | 0.313936 |
| cg19552598 | 7.22E-04 | 16 | rs568682346 | 0.193404 | 0.172275 | 0.175764 | 0.228488 |
| cg12264517 | 7.21E-04 | 5 | rs547534601 | 0.358877 | 0.333869 | 0.335793 | 0.453133 |
| cg23369670 | 7.21E-04 | 14 | rs542335896;rs562161730 | 0.332531 | 0.306088 | 0.308088 | 0.413811 |
| cg22729539 | 7.20E-04 | 12 | rs114916899;rs73417656 | 0.160534 | 0.134413 | 0.135756 | 0.194775 |
| cg14572478 | 7.20E-04 | 3 | rs556961266 | 0.236978 | 0.21467 | 0.207081 | 0.305923 |
| cg05415606 | 7.20E-04 | 2 | rs569127270;rs191923224 | 0.459023 | 0.431589 | 0.438591 | 0.497789 |
| cg11293275 | 7.19E-04 | 5 | rs554381521;rs185388380 | 0.27931 | 0.249599 | 0.258491 | 0.324761 |
| cg26903130 | 7.19E-04 | 12 |  | 0.332152 | 0.31112 | 0.308542 | 0.394124 |
| cg11154534 | 7.19E-04 | 5 | rs577064423;rs6865287; rs556784174 | 0.461271 | 0.43919 | 0.457087 | 0.535102 |
| cg09950966 | 7.18E-04 | 1 | rs569879518 | 0.358255 | 0.33487 | 0.329474 | 0.431762 |
| cg11391543 | 7.17E-04 | 16 | rs183291640;rs146524806 | 0.188568 | 0.155244 | 0.162734 | 0.225843 |
| cg17807279 | 7.17E-04 | 11 | rs531510399;rs190279688 | 0.26484 | 0.22986 | 0.218995 | 0.327488 |
| cg23386939 | 7.14E-04 | 16 | rs533358182;rs550492963 | 0.246843 | 0.22422 | 0.213349 | 0.317078 |
| cg24999217 | 7.13E-04 | 2 | rs531410860 | 0.238982 | 0.212083 | 0.204645 | 0.28841 |
| cg13106758 | 7.13E-04 | 15 | rs546788254 | 0.116516 | 0.096265 | 0.097879 | 0.150749 |
| cg03723688 | 7.12E-04 | 10 |  | 0.283785 | 0.252259 | 0.247661 | 0.3576 |
| cg25539505 | 7.12E-04 | 5 | rs543082109 | 0.346154 | 0.325613 | 0.319505 | 0.431374 |
| cg18974455 | 7.12E-04 | 8 |  | 0.271869 | 0.251852 | 0.246588 | 0.348766 |
| cg00788653 | 7.12E-04 | 16 | rs7202045;rs12447405 | 0.295286 | 0.252743 | 0.268925 | 0.376538 |
| cg19268599 | 7.11E-04 | 12 | rs146786862;rs535515145; rs181642584;rs147677490 | 0.372696 | 0.33188 | 0.336822 | 0.418361 |
| cg15328937 | 7.11E-04 | 2 | rs550020683;rs150558494 | 0.324895 | 0.299006 | 0.299641 | 0.390638 |
| cg10543836 | 7.10E-04 | 3 | rs140481146;rs566718860; rs534260656 | 0.669756 | 0.649378 | 0.662345 | 0.706763 |
| cg20107506 | 7.10E-04 | 4 | rs189200787;rs567919564 | 0.315154 | 0.277093 | 0.280689 | 0.414655 |
| cg20436925 | 7.08E-04 | 16 | rs118089749;rs141209123; rs538153933 | 0.305452 | 0.270252 | 0.270656 | 0.38601 |
| cg23889010 | 7.08E-04 | 20 | rs572756901;rs142770286 | 0.295591 | 0.256261 | 0.25551 | 0.382489 |
| cg19158670 | 7.07E-04 | 16 | rs552726332;rs5467 | 0.308894 | 0.276679 | 0.268179 | 0.377704 |
| cg11052716 | 7.07E-04 | 2 | rs112821619;rs188798962 | 0.296422 | 0.262331 | 0.260615 | 0.364641 |
| cg16948651 | 7.07E-04 | 1 | rs549726397 | 0.246727 | 0.226635 | 0.210381 | 0.305878 |
| cg21875396 | 7.05E-04 | 6 | rs572877486;rs571589687 | 0.257128 | 0.236178 | 0.236595 | 0.342996 |
| cg02615435 | 7.05E-04 | 6 | rs76684321;rs7747710 | 0.238134 | 0.211169 | 0.200788 | 0.299524 |
| cg08917944 | 7.05E-04 | 16 | rs573458052;rs186180023 | 0.249739 | 0.227639 | 0.218884 | 0.329784 |
| cg12893796 | 7.05E-04 | 4 |  | 0.304606 | 0.274012 | 0.271215 | 0.381659 |
| cg25348888 | 7.04E-04 | 5 | rs148639404;rs191239718 | 0.258663 | 0.215762 | 0.224422 | 0.323132 |
| cg17417856 | 7.03E-04 | 19 | rs555686920 | 0.546654 | 0.525962 | 0.536998 | 0.591175 |
| cg06290889 | 7.03E-04 | 20 | rs533331566;rs549923999; rs563736839 | 0.242598 | 0.215755 | 0.207604 | 0.304642 |
| cg10093938 | 7.03E-04 | 1 | rs578174083 | 0.358206 | 0.324712 | 0.312744 | 0.418126 |
| cg10156382 | 7.02E-04 | 15 | rs541668149;rs559952708 ; rs533550039;rs552130040; rs563823958 | 0.250634 | 0.227987 | 0.20973 | 0.318046 |
| cg15607142 | 7.01E-04 | 3 | rs6439141;rs6439142 | 0.29022 | 0.26636 | 0.251872 | 0.366113 |
| cg00311702 | 7.01E-04 | 10 | rs537371477 | 0.290297 | 0.255475 | 0.246698 | 0.369599 |
| cg14398691 | 7.01E-04 | 17 | rs572088947;rs542607246 | 0.312539 | 0.282284 | 0.296569 | 0.381977 |
| cg11648875 | 7.01E-04 | 7 | rs181015870 | 0.308652 | 0.273273 | 0.271266 | 0.401976 |
| cg23523937 | 7.00E-04 | 20 | rs73622027;rs531777284 | 0.258141 | 0.234975 | 0.226361 | 0.332492 |
| cg11184120 | 6.99E-04 | 12 | rs553646698 | 0.339139 | 0.315849 | 0.311994 | 0.423148 |
| cg15528736 | 6.99E-04 | 19 | rs183415283 | 0.315824 | 0.293718 | 0.29211 | 0.376923 |
| cg17479424 | 6.99E-04 | 2 | rs145617976 | 0.293287 | 0.267968 | 0.273692 | 0.373386 |
| cg24981221 | 6.98E-04 | 8 |  | 0.252294 | 0.229452 | 0.217302 | 0.322729 |
| cg17176113 | 6.98E-04 | 16 | rs560548889;rs181963926; rs116712806;rs568988476; rs374648259 | 0.240057 | 0.215856 | 0.205266 | 0.298324 |
| cg20954870 | 6.97E-04 | 5 | rs191166786;rs570820159 | 0.342648 | 0.306254 | 0.302221 | 0.4211 |
| cg18006980 | 6.97E-04 | 13 | rs573143027;rs117986217 | 0.459622 | 0.435947 | 0.452039 | 0.514526 |
| cg18321881 | 6.95E-04 | 7 | rs530321100;rs191581308 | 0.286521 | 0.254163 | 0.245852 | 0.356866 |
| cg11829879 | 6.95E-04 | 19 |  | 0.183274 | 0.163219 | 0.156141 | 0.228009 |
| cg01564818 | 6.95E-04 | 3 | rs575178396 | 0.216033 | 0.188709 | 0.188064 | 0.260545 |
| cg25272883 | 6.95E-04 | 2 | rs575700425;rs112810815; rs539269824;rs556179616 | 0.257529 | 0.225869 | 0.213772 | 0.345437 |
| cg11147278 | 6.94E-04 | 10 | rs140768416 | 0.19665 | 0.171958 | 0.175145 | 0.250273 |
| cg23090824 | 6.94E-04 | 6 | rs199914481 | 0.155501 | 0.131785 | 0.135892 | 0.188544 |
| cg06465076 | 6.93E-04 | 5 | rs192108291 | 0.343714 | 0.311865 | 0.323103 | 0.455398 |
| cg21622139 | 6.92E-04 | 15 |  | 0.545704 | 0.522235 | 0.538388 | 0.603979 |
| cg00091349 | 6.92E-04 | 3 | rs186283323;rs556118486 | 0.430001 | 0.40976 | 0.426302 | 0.51599 |
| cg20105437 | 6.92E-04 | 1 | rs75185347;rs139457797; rs116073037 | 0.319855 | 0.28694 | 0.285012 | 0.416742 |
| cg08598221 | 6.91E-04 | 8 |  | 0.419349 | 0.39555 | 0.404836 | 0.470667 |
| cg07363330 | 6.91E-04 | 5 | rs200532608 | 0.26428 | 0.236511 | 0.241271 | 0.348458 |
| cg11461407 | 6.90E-04 | 2 | rs536361813 | 0.274842 | 0.240998 | 0.237821 | 0.344174 |
| cg17569496 | 6.90E-04 | 9 | rs187357721;rs11138391; rs148163071 | 0.193176 | 0.172358 | 0.18 | 0.260401 |
| cg20757010 | 6.90E-04 | 12 | rs189085680 | 0.416709 | 0.392586 | 0.396971 | 0.472049 |
| cg12259002 | 6.89E-04 | 4 | rs571464490 | 0.358731 | 0.328377 | 0.337538 | 0.427553 |
| cg13864101 | 6.89E-04 | 1 | rs76312219 | 0.289203 | 0.264156 | 0.248544 | 0.372835 |
| cg22013864 | 6.88E-04 | 6 | rs555048502 | 0.309898 | 0.282712 | 0.268287 | 0.371489 |
| cg23404097 | 6.87E-04 | 3 | rs542554288;rs149222014; rs143410159 | 0.375845 | 0.348161 | 0.357926 | 0.452613 |
| cg09622624 | 6.86E-04 | 2 | rs147770788;rs550276364 | 0.337439 | 0.314645 | 0.31212 | 0.434709 |
| cg20693213 | 6.86E-04 | 3 | rs201516099 | 0.281188 | 0.259781 | 0.25527 | 0.375007 |
| cg02246090 | 6.86E-04 | 16 | rs538105426;rs556436058; rs146424688 | 0.326481 | 0.287221 | 0.286077 | NA |
| cg05599099 | 6.85E-04 | 1 | rs535696362;rs145023246; rs575713025 | 0.292133 | 0.262626 | 0.250138 | 0.350694 |
| cg26605477 | 6.85E-04 | 20 | rs114265611;rs78582716; rs146041803 | 0.398737 | 0.375073 | 0.361795 | 0.461245 |
| cg08593157 | 6.85E-04 | 17 |  | 0.611315 | 0.589468 | 0.597231 | 0.649752 |
| cg07699323 | 6.84E-04 | 8 | rs76488698 | 0.283489 | 0.236983 | 0.240656 | 0.36942 |
| cg20222562 | 6.84E-04 | 6 | rs148037901;rs555304700 | 0.301274 | 0.26283 | 0.245964 | 0.373125 |
| cg20052632 | 6.84E-04 | 12 | rs112560223 | 0.330243 | 0.29979 | 0.314408 | 0.390097 |
| cg05867001 | 6.84E-04 | 14 | rs372573129 | 0.326714 | 0.298721 | 0.297321 | 0.38872 |
| cg03209720 | 6.83E-04 | 6 | rs587683108;rs138417344; rs587639382 | 0.335665 | 0.303111 | 0.310825 | NA |
| cg06782950 | 6.82E-04 | 18 | rs192669430;rs142832204 | 0.230446 | 0.205802 | 0.199926 | 0.292852 |
| cg10056026 | 6.81E-04 | 10 | rs535078422;rs9888114; rs574981515;rs545523325 | 0.297688 | 0.25928 | 0.253015 | 0.372325 |
| cg00760729 | 6.81E-04 | 2 | rs556143547;rs577883685; rs143634225;rs556555646 | 0.229295 | 0.204948 | 0.197611 | 0.299396 |
| cg05954120 | 6.81E-04 | 1 | rs184917390;rs572149665; rs189364553 | 0.331673 | 0.299035 | 0.299045 | 0.391371 |
| cg10183963 | 6.81E-04 | 9 | rs539871413;rs558486629; rs112915500 | 0.321955 | 0.297222 | 0.296319 | 0.376865 |
| cg17266935 | 6.80E-04 | 6 | rs115871624;rs376600733 | 0.371336 | 0.33502 | 0.322404 | 0.469065 |
| cg14874993 | 6.79E-04 | 6 | rs528443430 | 0.30375 | 0.282399 | 0.276895 | 0.374119 |
| cg26976492 | 6.79E-04 | 2 | rs189069445 | 0.279715 | 0.242975 | 0.238549 | 0.366172 |
| cg25653900 | 6.78E-04 | 14 | rs566712485 | 0.224694 | 0.202696 | 0.203409 | 0.294707 |
| cg03544445 | 6.77E-04 | 2 | rs192204679;rs183912491 | 0.316098 | 0.290602 | 0.277523 | 0.397252 |
| cg21932231 | 6.77E-04 | 2 | rs3770885;rs147940496; rs546166244 | 0.307339 | 0.280129 | 0.285519 | 0.378444 |
| cg25348655 | 6.75E-04 | 7 | rs147993914;rs73680660 | 0.220745 | 0.19158 | 0.190776 | 0.288136 |
| cg21561989 | 6.74E-04 | 12 | rs561684648 | 0.750424 | 0.726104 | 0.736464 | 0.785551 |
| cg16322681 | 6.72E-04 | 11 |  | 0.322613 | 0.285439 | 0.27875 | 0.401536 |
| cg19286414 | 6.71E-04 | 16 |  | 0.323706 | 0.294423 | 0.293692 | 0.39524 |
| cg26273312 | 6.71E-04 | 3 |  | 0.286737 | 0.240841 | 0.245693 | 0.357036 |
| cg09010707 | 6.71E-04 | 12 | rs556651024;rs186219062; rs531581898 | 0.299958 | 0.269131 | 0.264676 | 0.363957 |
| cg21032100 | 6.71E-04 | 13 | rs78976457 | 0.500839 | 0.477063 | 0.484749 | 0.547834 |
| cg25025727 | 6.70E-04 | 13 | rs141412081 | 0.356449 | 0.331863 | 0.340324 | 0.451916 |
| cg21315017 | 6.68E-04 | 20 | rs535767097 | 0.322621 | 0.292797 | 0.300768 | 0.405542 |
| cg03327154 | 6.68E-04 | 13 | rs576349494;rs572571572; rs545150459 | 0.258166 | 0.227707 | 0.221507 | 0.340982 |
| cg01087534 | 6.67E-04 | 8 | rs546270133;rs562990128; rs184268070;rs542227497; rs562139390;rs142788301; rs73521212 | 0.234143 | 0.207832 | 0.211204 | 0.319885 |
| cg20729889 | 6.67E-04 | 4 | rs115790093;rs4478160 | 0.241992 | 0.21986 | 0.209478 | 0.292802 |
| cg10277044 | 6.66E-04 | 20 | rs112708102;rs80219446 | 0.362129 | 0.336536 | 0.342588 | 0.464526 |
| cg13048113 | 6.66E-04 | 2 | rs59730981;rs148679654; rs192362314 | 0.319035 | 0.279036 | 0.280939 | 0.398887 |
| cg23999640 | 6.65E-04 | 11 | rs145322941;rs557879490; rs573010700 | 0.302294 | 0.278562 | 0.274975 | 0.358641 |
| cg10140227 | 6.65E-04 | 11 | rs116186803 | 0.260298 | 0.233936 | 0.230085 | 0.338609 |
| cg02038962 | 6.65E-04 | 22 | rs113640349 | 0.388489 | 0.361574 | 0.351919 | 0.461908 |
| cg25103337 | 6.64E-04 | 1 | rs115326259 | 0.25684 | 0.232112 | 0.220386 | 0.32288 |
| cg00429622 | 6.64E-04 | 19 | rs553703499;rs570382463; rs190320144;rs556095432 | 0.324231 | 0.290551 | 0.299024 | 0.41563 |
| cg07249182 | 6.64E-04 | 7 | rs6946097;rs183637484 | 0.317725 | 0.289738 | 0.285091 | 0.403893 |
| cg25159832 | 6.63E-04 | 1 | rs538231555 | 0.28555 | 0.243809 | 0.23891 | 0.357528 |
| cg24428555 | 6.63E-04 | 1 | rs557110122 | 0.28757 | 0.256832 | 0.256128 | 0.345015 |
| cg13995774 | 6.63E-04 | 5 |  | 0.296443 | 0.262527 | 0.258985 | 0.36613 |
| cg08032476 | 6.63E-04 | 17 | rs141123280 | 0.287744 | 0.261783 | 0.269587 | 0.337304 |
| cg24527044 | 6.62E-04 | 7 | rs372457145;rs183961465; rs568012038;rs528891661; rs547118974;rs111552863 | 0.351803 | 0.319032 | 0.304922 | 0.446017 |
| cg07986907 | 6.61E-04 | 2 | rs148054695;rs140855509 | 0.605924 | 0.580588 | 0.593743 | 0.656986 |
| cg06320175 | 6.61E-04 | 1 |  | 0.693155 | 0.667249 | 0.685641 | 0.730979 |
| cg24134472 | 6.61E-04 | 5 | rs544449037;rs373561315; rs560424742 | 0.241259 | 0.21565 | 0.20609 | 0.309317 |
| cg17491991 | 6.58E-04 | 6 | rs566069765;rs528056047; rs547756552 | 0.274276 | 0.250866 | 0.262081 | 0.35741 |
| cg19069360 | 6.57E-04 | 12 | rs535524485;rs34885511; rs11061983;rs182556671; rs557669511;rs573050792 | 0.30176 | 0.271038 | 0.27658 | 0.38293 |
| cg16045681 | 6.57E-04 | 1 | rs143351098 | 0.234601 | 0.204922 | 0.198694 | 0.302516 |
| cg14364151 | 6.57E-04 | 14 | rs533384208;rs73289843 | 0.258917 | 0.227626 | 0.224855 | 0.31776 |
| cg24400974 | 6.56E-04 | 2 | rs537417611;rs189979095 | 0.323543 | 0.29018 | 0.289419 | 0.388104 |
| cg19557480 | 6.56E-04 | 17 | rs182047490 | 0.192954 | 0.157468 | 0.169469 | 0.260469 |
| cg20440319 | 6.55E-04 | 17 | rs117251797 | 0.30073 | 0.267236 | 0.26263 | 0.372743 |
| cg09010699 | 6.55E-04 | 3 | rs555640655;rs193017084; rs143975313 | 0.347168 | 0.305338 | 0.316654 | 0.444401 |
| cg00334362 | 6.55E-04 | 6 | rs545420927;rs565322467 | 0.233653 | 0.203793 | 0.198011 | 0.300375 |
| cg19577136 | 6.55E-04 | 1 | rs80297741 | 0.689511 | 0.669253 | 0.675729 | 0.722987 |
| cg14131824 | 6.54E-04 | 17 | rs553175417;rs140466120 | 0.257814 | 0.235157 | 0.224481 | 0.307708 |
| cg05743375 | 6.53E-04 | 13 |  | 0.168618 | 0.148291 | 0.151276 | 0.210828 |
| cg22118474 | 6.52E-04 | 15 |  | 0.24268 | 0.21472 | 0.215217 | 0.297083 |
| cg04701026 | 6.52E-04 | 9 | rs560011172 | 0.353105 | 0.323179 | 0.315125 | 0.435039 |
| cg23924737 | 6.52E-04 | 17 | rs548998607 | 0.305898 | 0.280159 | 0.280857 | 0.386824 |
| cg06308984 | 6.51E-04 | 16 | rs183939087;rs188834177 | 0.288269 | 0.2623 | 0.249261 | 0.353891 |
| cg00532474 | 6.49E-04 | 18 | rs141416904;rs3745003; rs146878771 | 0.45195 | 0.417096 | 0.423725 | 0.504965 |
| cg05463966 | 6.47E-04 | 11 | rs535357407 | 0.234038 | 0.208394 | 0.211426 | 0.284546 |
| cg07315452 | 6.47E-04 | 2 | rs548384930;rs140628159 | 0.277959 | 0.257919 | 0.245617 | 0.348043 |
| cg17457254 | 6.47E-04 | 14 | rs547809464 | 0.315499 | 0.290732 | 0.291875 | 0.39883 |
| cg18107573 | 6.46E-04 | 10 | rs564756926;rs578194265 | 0.283008 | 0.253217 | 0.246134 | 0.368798 |
| cg04869336 | 6.45E-04 | 3 | rs6763124;rs552044558; rs529588721 | 0.284403 | 0.246491 | 0.229619 | 0.362265 |
| cg25589279 | 6.44E-04 | 17 | rs547267115;rs552280105; rs571929519 | 0.16947 | 0.147262 | 0.136949 | 0.20793 |
| cg20877250 | 6.44E-04 | 5 |  | 0.270952 | 0.239731 | 0.234831 | 0.350151 |
| cg06975018 | 6.43E-04 | 6 | rs17788484 | 0.30186 | 0.2795 | 0.292261 | 0.408832 |
| cg06223856 | 6.43E-04 | 1 | rs142236452 | 0.290205 | 0.264063 | 0.251831 | 0.380628 |
| cg02927448 | 6.42E-04 | 12 | rs187013509 | 0.250327 | 0.226389 | 0.239881 | 0.303578 |
| cg04654716 | 6.42E-04 | 5 | rs540148844 | 0.468229 | 0.442386 | 0.461123 | 0.526251 |
| cg19114248 | 6.42E-04 | 1 | rs555145460;rs572121941; rs541533018;rs117658136 | 0.233983 | 0.209376 | 0.202767 | 0.28727 |
| cg20807920 | 6.41E-04 | 13 | rs77989441;rs531805414 | 0.475295 | 0.443234 | 0.457169 | 0.535852 |
| cg21757790 | 6.40E-04 | 21 |  | 0.35866 | 0.331646 | 0.32233 | 0.435325 |
| cg04636584 | 6.39E-04 | 1 | rs142955221;rs150698058; rs554989225;rs573204090 | 0.306169 | 0.271589 | 0.257393 | 0.383974 |
| cg11427511 | 6.38E-04 | 2 | rs537019804;rs180982280; rs574368733 | 0.304852 | 0.260124 | 0.257555 | 0.384733 |
| cg25978130 | 6.37E-04 | 7 | rs117047975;rs112312542; rs536914858 | 0.173797 | 0.14582 | 0.153933 | 0.215314 |
| cg16498634 | 6.37E-04 | 16 | rs11649549;rs183457556; rs572730675 | 0.227317 | 0.194495 | 0.191023 | 0.28291 |
| cg04054604 | 6.37E-04 | 19 | rs181819888 | 0.31193 | 0.286403 | 0.286093 | 0.366806 |
| cg01007979 | 6.37E-04 | 1 |  | 0.257337 | 0.228429 | 0.234982 | 0.321516 |
| cg25683854 | 6.36E-04 | 1 | rs191270732 | 0.250327 | 0.22462 | 0.205008 | 0.311932 |
| cg02280693 | 6.36E-04 | 6 | rs73564275 | 0.358277 | 0.33297 | 0.334219 | 0.428437 |
| cg10287970 | 6.35E-04 | 6 | rs187269790;rs565060047 | 0.313094 | 0.286745 | 0.267932 | 0.385039 |
| cg11460824 | 6.34E-04 | 7 | rs568930716 | 0.251758 | 0.229802 | 0.228376 | 0.317085 |
| cg06948629 | 6.33E-04 | 16 | rs532916646;rs117432663 | 0.295169 | 0.264306 | 0.260237 | 0.359553 |
| cg24386592 | 6.33E-04 | 13 | rs545272539 | 0.308569 | 0.282301 | 0.274444 | 0.375515 |
| cg05055371 | 6.32E-04 | 12 | rs575887555 | 0.447675 | 0.425337 | 0.43236 | 0.502631 |
| cg02302574 | 6.32E-04 | 13 | rs151062908;rs577119058 | 0.300066 | 0.278249 | 0.269372 | 0.394383 |
| cg00579955 | 6.32E-04 | 4 | rs138012314;rs188663751; rs531034972 | 0.341047 | 0.319329 | 0.315101 | 0.416894 |
| cg12782754 | 6.32E-04 | 9 | rs188995619;rs542905061 | 0.310609 | 0.286365 | 0.287419 | 0.389451 |
| cg10816237 | 6.32E-04 | 19 | rs104894696;rs200488037; rs150121265 | 0.294663 | 0.26774 | 0.254862 | 0.344195 |
| cg24509697 | 6.32E-04 | 16 | rs141735764;rs573917027; rs79194929;rs542889012; rs572873441 | 0.426041 | 0.405037 | 0.403805 | 0.463891 |
| cg21948812 | 6.32E-04 | 2 | rs536073420 | 0.260642 | 0.234861 | 0.228345 | 0.329861 |
| cg06311422 | 6.32E-04 | 6 |  | 0.245002 | 0.219521 | 0.221058 | 0.302466 |
| cg20392780 | 6.31E-04 | 14 | rs143475270;rs186905097; rs533021813;rs74536787 | 0.283374 | 0.260729 | 0.251269 | 0.331951 |
| cg20748065 | 6.30E-04 | 7 | rs41295381;rs374350596 | 0.265462 | 0.239536 | 0.225356 | 0.306767 |
| cg11654133 | 6.30E-04 | 12 | rs565762493 | 0.195471 | 0.175034 | 0.164259 | NA |
| cg09213710 | 6.30E-04 | 9 | rs562071979;rs574296722; rs541329727 | 0.341914 | 0.303752 | 0.304139 | 0.423566 |
| cg09732070 | 6.29E-04 | 12 | rs368381845 | 0.330715 | 0.309551 | 0.30863 | 0.404034 |
| cg14963498 | 6.28E-04 | 8 | rs28999675;rs28999674; rs11777942;rs2853230 | 0.310675 | 0.272463 | 0.268906 | 0.378439 |
| cg24233170 | 6.28E-04 | 2 | rs572653218;rs114796625; rs140532832 | 0.392042 | 0.370929 | 0.379765 | 0.476689 |
| cg13856752 | 6.28E-04 | 10 | rs150224313;rs138996499; rs571392600;rs1109073 | 0.222592 | 0.195692 | 0.193451 | 0.268523 |
| cg11786988 | 6.28E-04 | 17 | rs184361998;rs547845784; rs565973053 | 0.457623 | 0.433542 | 0.437653 | 0.505248 |
| cg26019173 | 6.28E-04 | 11 |  | 0.267617 | 0.245056 | 0.231367 | 0.334203 |
| cg08934272 | 6.27E-04 | 8 | rs193174163 | 0.274476 | 0.253825 | 0.256084 | 0.378173 |
| cg20398719 | 6.27E-04 | 9 | rs139904423 | 0.291152 | 0.262992 | 0.26011 | 0.360915 |
| cg18542546 | 6.26E-04 | 7 | rs559906182;rs148318726; rs542338199 | 0.344417 | 0.318407 | 0.306538 | 0.455342 |
| cg26213131 | 6.26E-04 | 2 | rs569058452;rs150065517 | 0.301634 | 0.267011 | 0.265862 | 0.366554 |
| cg14834285 | 6.24E-04 | 17 | rs140031985 | 0.297913 | 0.275076 | 0.276544 | 0.374539 |
| cg12061066 | 6.24E-04 | 12 | rs2046687;rs145238923; rs567123320 | 0.277799 | 0.233032 | 0.231372 | 0.343094 |
| cg05383910 | 6.24E-04 | 7 | rs376865635;rs535173476; rs555420066 | 0.165979 | 0.141201 | 0.146482 | 0.220412 |
| cg17479790 | 6.24E-04 | 17 | rs565184182 | 0.236552 | 0.208274 | 0.201348 | 0.295478 |
| cg08622826 | 6.23E-04 | 8 | rs540237807;rs12541260 | 0.333285 | 0.304983 | 0.316319 | 0.414073 |
| cg00034338 | 6.23E-04 | 6 | rs577162066 | 0.38302 | 0.343133 | 0.343168 | 0.484036 |
| cg25178074 | 6.23E-04 | 20 | rs567846987;rs146376934; rs112951395 | 0.261291 | 0.239493 | 0.222021 | 0.319034 |
| cg10933827 | 6.22E-04 | 7 | rs568430141;rs77196411; rs112143869 | 0.221583 | 0.193164 | 0.18708 | 0.286115 |
| cg15144116 | 6.22E-04 | 12 | rs72645148;rs16932569; rs555160968;rs137916184 | 0.314273 | 0.291704 | 0.283548 | 0.365702 |
| cg26847100 | 6.22E-04 | 19 | rs541056166 | 0.243941 | 0.221956 | 0.211718 | 0.305243 |
| cg08181251 | 6.22E-04 | 10 | rs549421952;rs567827542; rs528825895 | 0.281593 | 0.252826 | 0.255895 | 0.352169 |
| cg25596082 | 6.21E-04 | 10 | rs77765286;rs577782542 | 0.210851 | 0.181133 | 0.177445 | 0.239946 |
| cg10471743 | 6.21E-04 | 5 | rs182892086;rs564395070; rs303226;rs114003667 | 0.238173 | 0.20726 | 0.199405 | 0.312684 |
| cg20775668 | 6.20E-04 | 5 | rs534264782;rs554795672 | 0.290666 | 0.26519 | 0.261408 | 0.352094 |
| cg07395331 | 6.19E-04 | 12 | rs74519445;rs550081893 | 0.316559 | 0.280083 | 0.287197 | 0.392368 |
| cg20708537 | 6.19E-04 | 19 | rs546307715;rs566446426; rs12151305;rs549656274; rs115997950;rs7255931 | 0.32916 | 0.297577 | 0.289012 | 0.41717 |
| cg24830503 | 6.18E-04 | 1 | rs12095441;rs148070477; rs565503817 | 0.271666 | 0.247127 | 0.244531 | 0.320229 |
| cg10001455 | 6.17E-04 | 5 | rs538397589;rs550260396 | 0.364762 | 0.323399 | 0.327315 | 0.435553 |
| cg23965230 | 6.17E-04 | 10 | rs561651502;rs115319447 | 0.251922 | 0.218145 | 0.212302 | 0.317887 |
| cg15033222 | 6.16E-04 | 2 |  | 0.271058 | 0.223506 | 0.225991 | 0.346795 |
| cg08792630 | 6.16E-04 | 6 | rs113486951 | 0.273895 | 0.236108 | 0.235652 | 0.347774 |
| cg13072055 | 6.16E-04 | 18 |  | 0.282951 | 0.25848 | 0.244708 | 0.361463 |
| cg01922918 | 6.15E-04 | 1 | rs536063237 | 0.286994 | 0.249293 | 0.244762 | 0.339083 |
| cg16026561 | 6.15E-04 | 20 | rs146256990;rs561762088 | 0.305301 | 0.270204 | 0.26979 | 0.376573 |
| cg07909786 | 6.14E-04 | 1 |  | 0.208596 | 0.177809 | 0.179406 | 0.289923 |
| cg22825487 | 6.13E-04 | 6 | rs372545245;rs567414387; rs6942306;rs184715473 | 0.321829 | 0.276629 | 0.272909 | 0.402248 |
| cg14268695 | 6.13E-04 | 20 | rs561865019;rs150413599 | 0.340999 | 0.313592 | 0.319627 | 0.393232 |
| cg21530469 | 6.13E-04 | 2 | rs142397295 | 0.409137 | 0.364282 | 0.363871 | 0.482732 |
| cg07393105 | 6.13E-04 | 7 | rs373887179;rs544971546; rs564962439;rs140481945 | 0.296491 | 0.266527 | 0.280576 | 0.398704 |
| cg04097923 | 6.13E-04 | 17 | rs8064385;rs563284740; rs532221424 | 0.36277 | 0.334237 | 0.328219 | 0.431397 |
| cg09575262 | 6.13E-04 | 3 | rs112998023;rs77218111; rs144368455;rs550979487; rs72994222;rs144954838; rs554701001 | 0.394273 | 0.361761 | 0.368686 | 0.46741 |
| cg21602257 | 6.11E-04 | 20 | rs200854507;rs557169130 | 0.317923 | 0.295439 | 0.293288 | 0.387934 |
| cg09503838 | 6.11E-04 | 15 | rs148460096;rs142596486; rs573242336 | 0.331995 | 0.308015 | 0.312532 | 0.380003 |
| cg18019234 | 6.11E-04 | 2 | rs77843365 | 0.266505 | 0.244487 | 0.233547 | 0.338687 |
| cg16970323 | 6.10E-04 | 20 | rs181082783;rs146249443 | 0.200129 | 0.177591 | 0.164126 | 0.239692 |
| cg03445932 | 6.09E-04 | 3 | rs200183090 | 0.277771 | 0.246073 | 0.247376 | 0.371287 |
| cg21737434 | 6.09E-04 | 12 |  | 0.230936 | 0.204691 | 0.199897 | 0.27385 |
| cg23489137 | 6.09E-04 | 2 | rs376811752;rs190820973 | 0.240531 | 0.200205 | 0.210547 | 0.301677 |
| cg18554789 | 6.09E-04 | 7 | rs189114199 | 0.251139 | 0.215347 | 0.214817 | 0.325075 |
| cg16768949 | 6.06E-04 | 6 | rs145730163;rs148954268 | 0.156496 | 0.130126 | 0.140843 | 0.188404 |
| cg20711480 | 6.06E-04 | 1 |  | 0.438906 | 0.407825 | 0.425871 | 0.511409 |
| cg18677212 | 6.06E-04 | 9 | rs144608302;rs113050665; rs139453121;rs576285650 | 0.3364 | 0.303535 | 0.290229 | 0.4234 |
| cg04224187 | 6.06E-04 | 3 | rs149350954;rs556378242; rs41308222 | 0.301042 | 0.277518 | 0.274887 | 0.356487 |
| cg08124984 | 6.05E-04 | 3 | rs141360935;rs146483233 | 0.223831 | 0.202573 | 0.191642 | 0.300442 |
| cg18766962 | 6.05E-04 | 3 |  | 0.238615 | 0.21013 | 0.228178 | 0.313071 |
| cg14135462 | 6.04E-04 | 11 | rs536679503;rs182513782; rs565680621 | 0.217767 | 0.179408 | 0.179162 | 0.27822 |
| cg11254053 | 6.04E-04 | 1 | rs577784575;rs115480196 | 0.292782 | 0.262707 | 0.263901 | 0.373376 |
| cg00044665 | 6.04E-04 | 16 | rs533099944;rs185275701 | 0.278417 | 0.250597 | 0.260843 | 0.327001 |
| cg09978533 | 6.03E-04 | 22 |  | 0.210196 | 0.189522 | 0.191553 | 0.266549 |
| cg17884069 | 6.03E-04 | 8 | rs529474839 | 0.296694 | 0.26916 | 0.24987 | 0.396947 |
| cg10772452 | 6.02E-04 | 16 | rs562806664;rs576062786; rs143561608 | 0.256922 | 0.218523 | 0.215734 | 0.336524 |
| cg09969488 | 6.02E-04 | 2 | rs555923167;rs568131476 | 0.269365 | 0.247821 | 0.247184 | 0.333244 |
| cg15148329 | 6.02E-04 | 4 | rs17006521 | 0.199596 | 0.179243 | 0.16979 | 0.262349 |
| cg05697766 | 6.02E-04 | 16 | rs151122134;rs527807979; rs117879661 | 0.291633 | 0.255138 | 0.255066 | 0.374286 |
| cg15142192 | 6.01E-04 | 1 | rs569965014 | 0.281491 | 0.21567 | 0.223692 | 0.335017 |
| cg06147190 | 6.01E-04 | 9 | rs564036245 | 0.293158 | 0.260854 | 0.244425 | 0.375606 |
| cg22558269 | 6.01E-04 | 2 | rs560916876;rs574754768 | 0.343459 | 0.320418 | 0.318215 | 0.432213 |
| cg25505880 | 5.99E-04 | 11 |  | 0.287809 | 0.257445 | 0.250075 | 0.342807 |
| cg17274673 | 5.99E-04 | 5 | rs9324653;rs138887482; rs142896672 | 0.317919 | 0.294421 | 0.298615 | 0.386084 |
| cg25596327 | 5.98E-04 | 1 | rs144635152 | 0.295116 | 0.249527 | 0.240895 | 0.366197 |
| cg13941783 | 5.97E-04 | 22 | rs555623722 | 0.312121 | 0.280948 | 0.270188 | 0.373117 |
| cg13844237 | 5.97E-04 | 9 | rs13302549;rs188881132 | 0.670582 | 0.645968 | 0.640279 | 0.720582 |
| cg15013199 | 5.96E-04 | 5 |  | 0.288816 | 0.266992 | 0.259459 | 0.356909 |
| cg04430911 | 5.95E-04 | 1 | rs540301812;rs537953626 | 0.314784 | 0.291888 | 0.288591 | 0.377924 |
| cg12595667 | 5.95E-04 | 2 | rs559686336 | 0.152732 | 0.130933 | 0.141406 | 0.219204 |
| cg21390137 | 5.94E-04 | 16 | rs538543085;rs186235484; rs147670192 | 0.2406 | 0.213928 | 0.219251 | 0.286426 |
| cg21424270 | 5.92E-04 | 3 | rs556375857;rs574560369 | 0.195721 | 0.174984 | 0.175919 | 0.254402 |
| cg12274272 | 5.91E-04 | 1 | rs553255898;rs139933682 | 0.282536 | 0.248305 | 0.241049 | 0.363959 |
| cg26877303 | 5.91E-04 | 17 | rs578153004;rs139585541; rs144395794;rs529475646 | 0.275456 | 0.237846 | 0.239191 | 0.337333 |
| cg09953526 | 5.90E-04 | 12 | rs75270533 | 0.248897 | 0.222205 | 0.210507 | 0.319974 |
| cg03714884 | 5.89E-04 | 4 | rs542368679 | 0.308139 | 0.277788 | 0.268257 | 0.376988 |
| cg08918523 | 5.88E-04 | 18 | rs528138092 | 0.261109 | 0.231722 | 0.227295 | 0.324131 |
| cg16354423 | 5.88E-04 | 19 | rs547000909 | 0.351203 | 0.319699 | 0.330252 | 0.429981 |
| cg08701420 | 5.87E-04 | 10 | rs570806585 | 0.333147 | 0.310183 | 0.299177 | 0.410283 |
| cg02251680 | 5.86E-04 | 12 | rs529236519 | 0.279593 | 0.240584 | 0.241165 | 0.371495 |
| cg19214942 | 5.86E-04 | 13 | rs540897390 | 0.255387 | 0.224473 | 0.221371 | 0.333393 |
| cg21248089 | 5.86E-04 | 6 | rs528140674;rs547682455 | 0.281997 | 0.24042 | 0.240321 | 0.371739 |
| cg17541002 | 5.86E-04 | 12 | rs1612131;rs11615578; rs551888066 | 0.2884 | 0.261623 | 0.245452 | 0.363396 |
| cg00400869 | 5.85E-04 | 19 | rs190116700;rs570465211 | 0.224155 | 0.199533 | 0.191805 | 0.268579 |
| cg00303252 | 5.85E-04 | 9 | rs147031741;rs55755225 | 0.254662 | 0.21549 | 0.218302 | 0.321089 |
| cg18905278 | 5.85E-04 | 6 |  | 0.280629 | 0.248085 | 0.238 | 0.353503 |
| cg12599598 | 5.85E-04 | 4 | rs563066779 | 0.310445 | 0.28285 | 0.283469 | 0.381889 |
| cg25938803 | 5.85E-04 | 2 | rs183844032;rs189228924; rs77520767 | 0.279895 | 0.230259 | 0.227154 | 0.350851 |
| cg11012463 | 5.84E-04 | 1 | rs180916393;rs537759737 | 0.304597 | 0.269356 | 0.25602 | 0.364702 |
| cg11125805 | 5.84E-04 | 5 | rs150945002;rs560248373 | 0.297015 | 0.264758 | 0.256772 | 0.367408 |
| cg16858025 | 5.84E-04 | 4 | rs544382831;rs140147835 | 0.260594 | 0.229402 | 0.217943 | 0.346308 |
| cg11998002 | 5.83E-04 | 12 | rs369385529;rs113542589; rs144022952 | 0.296267 | 0.258862 | 0.259725 | 0.383957 |
| cg11030829 | 5.83E-04 | 1 | rs573640528 | 0.295247 | 0.269908 | 0.254461 | 0.365254 |
| cg21492378 | 5.83E-04 | 9 |  | 0.312868 | 0.278052 | 0.271796 | 0.394044 |
| cg26637548 | 5.81E-04 | 5 | rs544574810;rs1644431 | 0.50765 | 0.486543 | 0.497629 | 0.570925 |
| cg05344747 | 5.81E-04 | 11 | rs187867191 | 0.338938 | 0.299508 | 0.318256 | 0.439774 |
| cg19179237 | 5.80E-04 | 7 |  | 0.237976 | 0.213173 | 0.204059 | 0.306676 |
| cg06340304 | 5.80E-04 | 2 | rs4675729;rs73983681; rs149956099 | 0.232468 | 0.200655 | 0.196298 | 0.28463 |
| cg01574116 | 5.80E-04 | 7 | rs78900159;rs150052117; rs543019480;rs12333877 | 0.260141 | 0.237718 | 0.234061 | 0.326901 |
| cg23915630 | 5.79E-04 | 4 | rs151321165;rs78798049; rs373838793;rs542846564 | 0.289535 | 0.260343 | 0.263921 | 0.353725 |
| cg07198620 | 5.79E-04 | 1 | rs839648 | 0.292036 | 0.264391 | 0.251182 | 0.343623 |
| cg02558361 | 5.77E-04 | 19 | rs534408824 | 0.254388 | 0.233646 | 0.21645 | 0.306198 |
| cg08472008 | 5.77E-04 | 4 | rs77903343 | 0.243806 | 0.213866 | 0.210188 | 0.318297 |
| cg13675859 | 5.77E-04 | 6 | rs532761407 | 0.420651 | 0.397187 | 0.400631 | 0.502022 |
| cg00172603 | 5.77E-04 | 1 | rs190462714;rs182242639; rs186382070 | 0.13229 | 0.103578 | 0.101983 | 0.156588 |
| cg01257083 | 5.77E-04 | 4 | rs531246661;rs548125768 | 0.328364 | 0.305757 | 0.314589 | 0.392862 |
| cg21060726 | 5.77E-04 | 14 | rs73326854 | 0.4536 | 0.433558 | 0.439382 | 0.520895 |
| cg26622740 | 5.77E-04 | 4 | rs558672355 | 0.205854 | 0.181897 | 0.180524 | 0.271225 |
| cg00817529 | 5.75E-04 | 16 | rs550290814 | 0.389061 | 0.350387 | 0.339746 | 0.475475 |
| cg10516613 | 5.75E-04 | 6 | rs143947084;rs576500753 | 0.481585 | 0.449082 | 0.45726 | NA |
| cg12209946 | 5.73E-04 | 3 | rs138241146;rs149587931; rs558873015;rs569479438 | 0.300787 | 0.277582 | 0.272137 | 0.396159 |
| cg05880730 | 5.73E-04 | 9 | rs2487912 | 0.283786 | 0.25562 | 0.251658 | 0.335045 |
| cg14520947 | 5.73E-04 | 1 | rs11577164 | 0.284743 | 0.239027 | 0.245997 | 0.360248 |
| cg16350446 | 5.72E-04 | 2 | rs181668017 | 0.239699 | 0.208618 | 0.208548 | 0.316206 |
| cg04954286 | 5.72E-04 | 3 | rs577311525 | 0.219622 | 0.190677 | 0.184032 | 0.279101 |
| cg05193189 | 5.71E-04 | 12 | rs373808043;rs5744827 | 0.334748 | 0.302394 | 0.296957 | 0.43797 |
| cg23700338 | 5.71E-04 | 1 | rs183734490;rs534364706 | 0.343612 | 0.314882 | 0.320098 | 0.450748 |
| cg13114711 | 5.71E-04 | 12 | rs550131299;rs186961607 | 0.345701 | 0.304337 | 0.298513 | 0.433232 |
| cg25165969 | 5.71E-04 | 16 | rs574582800;rs188977370 | 0.421213 | 0.393335 | 0.413083 | 0.469523 |
| cg25609803 | 5.71E-04 | 1 | rs565640871 | 0.330885 | 0.307369 | 0.295674 | 0.391841 |
| cg11481582 | 5.69E-04 | 10 | rs146097737 | 0.190252 | 0.162334 | 0.153774 | 0.218724 |
| cg03621504 | 5.69E-04 | 12 |  | 0.20937 | 0.175846 | 0.17892 | 0.258566 |
| cg09709227 | 5.69E-04 | 13 |  | 0.286386 | 0.261909 | 0.254906 | 0.374723 |
| cg01878805 | 5.68E-04 | 2 | rs570764911 | 0.282845 | 0.241421 | 0.238824 | 0.377991 |
| cg26434370 | 5.67E-04 | 16 | rs561984595 | 0.34007 | 0.312734 | 0.305032 | 0.411244 |
| cg07409383 | 5.67E-04 | 5 |  | 0.305871 | 0.271145 | 0.256714 | NA |
| cg14547936 | 5.67E-04 | 14 | rs532174900;rs35501816; rs532828948 | 0.399449 | 0.358204 | 0.360891 | 0.498287 |
| cg14131038 | 5.66E-04 | 2 | rs35678376;rs12992944; rs563607091;rs542002804 | 0.318735 | 0.282363 | 0.289266 | 0.376022 |
| cg26964724 | 5.66E-04 | 4 | rs567381081;rs1460018; rs553018143;rs571233585 | 0.177552 | 0.1561 | 0.161501 | 0.247538 |
| cg07123846 | 5.65E-04 | 11 | rs562981650;rs530256936 | 0.295048 | 0.265007 | 0.261183 | 0.377682 |
| cg03876564 | 5.65E-04 | 16 | rs181818444;rs371271151; rs77296863;rs566148451; rs539953157 | 0.389001 | 0.368174 | 0.376995 | 0.454391 |
| cg13155300 | 5.65E-04 | 21 | rs115385619;rs144033196 | 0.336432 | 0.309698 | 0.307824 | 0.411599 |
| cg03279442 | 5.64E-04 | 2 | rs78668276 | 0.284761 | 0.252299 | 0.236154 | 0.38648 |
| cg24949238 | 5.64E-04 | 20 |  | 0.353979 | 0.333178 | 0.333338 | 0.431176 |
| cg22311446 | 5.64E-04 | 11 | rs200339999;rs142871932; rs2271589;rs139576225 | 0.292508 | 0.272153 | 0.262253 | 0.369893 |
| cg08850922 | 5.64E-04 | 4 |  | 0.376282 | 0.355174 | 0.362936 | 0.415847 |
| cg08389123 | 5.64E-04 | 13 | rs189231275;rs540408926; rs560551119 | 0.286162 | 0.255272 | 0.245022 | 0.364781 |
| cg19389293 | 5.63E-04 | 18 | rs561677630 | 0.243396 | 0.211097 | 0.2098 | 0.289698 |
| cg06706156 | 5.63E-04 | 1 |  | 0.319417 | 0.297844 | 0.283581 | 0.386941 |
| cg03580624 | 5.62E-04 | 19 | rs189411674;rs542424732; rs112849508 | 0.324239 | 0.30161 | 0.292889 | 0.405042 |
| cg23393544 | 5.62E-04 | 11 |  | 0.229476 | 0.203604 | 0.203217 | 0.303672 |
| cg23087531 | 5.61E-04 | 1 | rs554755879;rs60564108 | 0.241915 | 0.204805 | 0.190081 | 0.309402 |
| cg09946714 | 5.60E-04 | 12 | rs548941079 | 0.28991 | 0.257247 | 0.259807 | 0.368499 |
| cg17512517 | 5.59E-04 | 3 | rs569407257 | 0.401454 | 0.352726 | 0.363025 | 0.460277 |
| cg06685556 | 5.59E-04 | 17 | rs548324723;rs569739638 | 0.272538 | 0.239094 | 0.236879 | 0.330955 |
| cg15230710 | 5.59E-04 | 4 | rs533453751;rs551935480; rs563647711;rs530942225; rs549656082 | 0.283834 | 0.262956 | 0.253351 | 0.345309 |
| cg17517296 | 5.58E-04 | 2 | rs143499079 | 0.311934 | 0.287927 | 0.285765 | 0.370583 |
| cg26408390 | 5.57E-04 | 17 | rs574090692;rs543201246 | 0.479659 | 0.458408 | 0.466861 | 0.537002 |
| cg26929261 | 5.57E-04 | 5 | rs539754571;rs558470389; rs576556271 | 0.236378 | 0.215081 | 0.227398 | 0.198265 |
| cg01537883 | 5.57E-04 | 14 |  | 0.260515 | 0.224722 | 0.229017 | 0.320259 |
| cg25989526 | 5.56E-04 | 13 | rs80026366;rs115287517; rs577460129;rs534957171; rs140715784 | 0.227859 | 0.206864 | 0.198862 | 0.286349 |
| cg09393287 | 5.56E-04 | 22 | rs536173594;rs189406492; rs77751661;rs535040149 | 0.164396 | 0.14402 | 0.140032 | 0.208302 |
| cg17178546 | 5.56E-04 | 17 | rs139240472;rs549152974 | 0.340204 | 0.313936 | 0.309625 | 0.403565 |
| cg02640484 | 5.55E-04 | 10 | rs147473337 | 0.314057 | 0.276103 | 0.269707 | 0.383208 |
| cg09939625 | 5.55E-04 | 7 | rs544839342;rs201846940 | 0.255245 | 0.230922 | 0.239712 | 0.309562 |
| cg05653176 | 5.54E-04 | 18 | rs150163886;rs555486908; rs138607699 | 0.248488 | 0.203231 | 0.216539 | 0.309774 |
| cg06820437 | 5.54E-04 | 3 | rs78868657;rs538797338; rs139534384;rs538533595 | 0.261756 | 0.234703 | 0.224628 | 0.332562 |
| cg26725604 | 5.53E-04 | 18 |  | 0.25947 | 0.221368 | 0.218604 | 0.336495 |
| cg13623137 | 5.53E-04 | 15 | rs536808190;rs548508888 | 0.239321 | 0.205837 | 0.200624 | 0.314666 |
| cg04188216 | 5.53E-04 | 20 | rs556870936;rs111729138 | 0.270108 | 0.243256 | 0.237157 | 0.346774 |
| cg07276867 | 5.52E-04 | 9 | rs75981550 | 0.422434 | 0.392928 | 0.383704 | 0.481849 |
| cg04114966 | 5.52E-04 | 5 | rs7710619;rs527264487; rs540930816;rs77973107 | 0.283088 | 0.243984 | 0.236048 | 0.346448 |
| cg20242427 | 5.52E-04 | 11 |  | 0.27935 | 0.245773 | 0.229709 | 0.354491 |
| cg10301072 | 5.52E-04 | 4 | rs570057856 | 0.229547 | 0.208202 | 0.200769 | 0.285284 |
| cg26852723 | 5.51E-04 | 10 | rs188762393;rs142252042; rs555450514 | 0.235017 | 0.208657 | 0.206824 | 0.288725 |
| cg16323245 | 5.51E-04 | 3 | rs147638811;rs541234710; rs559356369 | 0.256134 | 0.233022 | 0.230275 | 0.330001 |
| cg25830243 | 5.51E-04 | 1 |  | 0.218383 | 0.194676 | 0.194231 | 0.296958 |
| cg19679092 | 5.51E-04 | 7 | rs535693985;rs185497815 | 0.180451 | 0.157656 | 0.156316 | 0.233007 |
| cg24853628 | 5.51E-04 | 7 | rs188525130 | 0.256521 | 0.220244 | 0.221273 | 0.334671 |
| cg25006194 | 5.51E-04 | 12 | rs150108160;rs55794167; rs558651699 | 0.231544 | 0.20811 | 0.198667 | 0.272676 |
| cg16160311 | 5.50E-04 | 10 | rs11815111 | 0.226864 | 0.195874 | 0.196398 | 0.278913 |
| cg18148870 | 5.47E-04 | 14 |  | 0.286352 | 0.23959 | 0.248193 | 0.353306 |
| cg01229061 | 5.47E-04 | 11 |  | 0.292321 | 0.265531 | 0.257555 | 0.36295 |
| cg20208935 | 5.47E-04 | 19 | rs188229585;rs144888545; rs193163181 | 0.254895 | 0.223588 | 0.220664 | 0.31103 |
| cg09068039 | 5.46E-04 | 4 | rs568323454;rs533962656 | 0.321 | 0.294251 | 0.289771 | 0.411033 |
| cg25436217 | 5.44E-04 | 22 | rs34943118 | 0.569713 | 0.543492 | 0.549906 | 0.638146 |
| cg09060789 | 5.44E-04 | 11 | rs182701610;rs561619218 | 0.33498 | 0.306072 | 0.298058 | 0.377503 |
| cg15800212 | 5.43E-04 | 16 | rs543320455;rs182993298; rs142631602;rs532043395; rs139271589 | 0.386709 | 0.357445 | 0.367062 | 0.457284 |
| cg09453768 | 5.43E-04 | 10 | rs570030026 | 0.285669 | 0.258465 | 0.251942 | 0.359639 |
| cg04450104 | 5.43E-04 | 1 | rs200581940;rs530899566 | 0.282604 | 0.252265 | 0.243705 | 0.354265 |
| cg19423170 | 5.41E-04 | 21 | rs73360979;rs527835256 | 0.354676 | 0.296195 | 0.303763 | 0.448211 |
| cg04034093 | 5.41E-04 | 14 | rs577459303;rs527308790; rs548112163;rs148330967 | 0.329879 | 0.292207 | 0.291606 | 0.391102 |
| cg17645652 | 5.40E-04 | 2 | rs561876293 | 0.42255 | 0.389242 | 0.404335 | 0.506688 |
| cg13672625 | 5.40E-04 | 6 | rs183496956 | 0.220771 | 0.192643 | 0.192103 | 0.267518 |
| cg19074962 | 5.40E-04 | 11 | rs566145807 | 0.224582 | 0.200679 | 0.213392 | 0.26793 |
| cg18190776 | 5.40E-04 | 5 | rs546988346 | 0.302524 | 0.282325 | 0.280706 | 0.379786 |
| cg19927678 | 5.39E-04 | 17 | rs560767825 | 0.25014 | 0.2183 | 0.221759 | 0.34859 |
| cg23579490 | 5.39E-04 | 11 | rs535781012;rs555619108 | 0.250918 | 0.220367 | 0.215762 | 0.335833 |
| cg17381185 | 5.38E-04 | 9 |  | 0.305467 | 0.275548 | 0.279597 | 0.381875 |
| cg06664104 | 5.38E-04 | 10 | rs537108440;rs550093270; rs661674 | 0.251451 | 0.221352 | 0.21976 | 0.307237 |
| cg13392051 | 5.38E-04 | 11 | rs534536283;rs139244130; rs553833140 | 0.2148 | 0.190564 | 0.198519 | 0.268965 |
| cg19714737 | 5.37E-04 | 11 | rs573349154;rs144224475; rs72981727;rs532665617; rs148283105 | 0.353845 | 0.324729 | 0.338205 | 0.401831 |
| cg13728797 | 5.37E-04 | 8 | rs149754108;rs73344827 | 0.414385 | 0.385278 | 0.388445 | 0.503293 |
| cg11522251 | 5.34E-04 | 9 | rs562758231;rs530289722; rs548476344;rs567149925 | 0.259175 | 0.238408 | 0.247481 | 0.335264 |
| cg21308575 | 5.34E-04 | 19 | rs534804490;rs573733246; rs577590788 | 0.192494 | 0.166138 | 0.165894 | 0.232646 |
| cg12965020 | 5.34E-04 | 6 | rs541809008;rs556100437 | 0.277047 | 0.255141 | 0.243432 | 0.360786 |
| cg16457662 | 5.33E-04 | 11 | rs550146210 | 0.271797 | 0.238106 | 0.230722 | 0.345531 |
| cg00819078 | 5.33E-04 | 5 | rs574934165 | 0.504507 | 0.480506 | 0.486437 | 0.561233 |
| cg14685356 | 5.33E-04 | 6 | rs528130407;rs562224649 | 0.269224 | 0.239983 | 0.240078 | 0.355494 |
| cg14315807 | 5.32E-04 | 7 | rs191042343 | 0.224324 | 0.198832 | 0.195835 | 0.284709 |
| cg12247014 | 5.32E-04 | 2 | rs76254199 | 0.472344 | 0.447338 | 0.465978 | 0.558754 |
| cg00446046 | 5.31E-04 | 3 | rs547977258;rs561500242; rs530306646 | 0.327188 | 0.281575 | 0.290118 | 0.417551 |
| cg16416987 | 5.31E-04 | 1 | rs199961310;rs145573392; rs143538680 | 0.275569 | 0.251098 | 0.252984 | 0.345001 |
| cg14729980 | 5.31E-04 | 18 |  | 0.411816 | 0.391599 | 0.395849 | 0.471898 |
| cg05506665 | 5.30E-04 | 1 | rs546709577;rs79522782; rs558066282;rs557167449 | 0.282469 | 0.254462 | 0.24447 | 0.344832 |
| cg20697025 | 5.30E-04 | 7 | rs73270232;rs191960981; rs142039133;rs372665565 | 0.128932 | 0.105502 | 0.108706 | 0.162418 |
| cg09165919 | 5.30E-04 | 19 | rs144868650;rs550179848; rs570331375 | 0.34771 | 0.320413 | 0.334078 | 0.423631 |
| cg21124310 | 5.29E-04 | 5 | rs78213348;rs566753644 | 0.221738 | 0.194936 | 0.187089 | 0.284053 |
| cg10725937 | 5.29E-04 | 6 | rs569649036 | 0.290344 | 0.254301 | 0.25816 | 0.367898 |
| cg06777739 | 5.28E-04 | 1 | rs112896140;rs180774719; rs562414892 | 0.294771 | 0.268733 | 0.261789 | 0.345133 |
| cg02819287 | 5.28E-04 | 6 | rs531499747;rs564218515 | 0.326949 | 0.305568 | 0.304577 | 0.406226 |
| cg01059929 | 5.27E-04 | 1 |  | 0.231645 | 0.208637 | 0.214274 | 0.293587 |
| cg19210276 | 5.26E-04 | 11 | rs554573323;rs11570176 | 0.202221 | 0.181067 | 0.182387 | 0.284868 |
| cg00741295 | 5.25E-04 | 3 | rs558273175 | 0.226967 | 0.206257 | 0.218965 | 0.203269 |
| cg18940763 | 5.23E-04 | 22 | rs189418764 | 0.159046 | 0.130394 | 0.140672 | 0.18872 |
| cg15899136 | 5.23E-04 | 13 | rs142358851;rs73184431; rs151281571 | 0.309258 | 0.278766 | 0.277145 | 0.401527 |
| cg26817092 | 5.22E-04 | 5 |  | 0.149746 | 0.125761 | 0.125745 | 0.189543 |
| cg01438390 | 5.22E-04 | 1 |  | 0.298938 | 0.259911 | 0.256774 | 0.392022 |
| cg03765062 | 5.22E-04 | 12 | rs542325470 | 0.264471 | 0.239472 | 0.22927 | 0.350295 |
| cg07876665 | 5.21E-04 | 8 | rs546227240 | 0.315627 | 0.283327 | 0.280454 | 0.407615 |
| cg14835461 | 5.21E-04 | 22 | rs577168380;rs138508749 | 0.3161 | 0.28642 | 0.286014 | 0.39277 |
| cg13011390 | 5.21E-04 | 16 | rs184707741;rs1684611 | 0.351501 | 0.315699 | 0.312375 | 0.423237 |
| cg15344180 | 5.20E-04 | 3 | rs560943867;rs147825866 | 0.357087 | 0.336509 | 0.330149 | 0.417256 |
| cg07952576 | 5.20E-04 | 3 |  | 0.290622 | 0.255554 | 0.243101 | 0.362825 |
| cg16927415 | 5.20E-04 | 4 | rs988163 | 0.179589 | 0.155463 | 0.163662 | 0.233537 |
| cg18085030 | 5.19E-04 | 11 |  | 0.276001 | 0.248164 | 0.253517 | 0.326281 |
| cg09833738 | 5.19E-04 | 3 | rs188009834;rs540575287; rs561443596;rs114008071 | 0.294809 | 0.25635 | 0.239767 | 0.368635 |
| cg24165874 | 5.18E-04 | 18 | rs556422315 | 0.252827 | 0.224248 | 0.228639 | 0.331255 |
| cg16834726 | 5.18E-04 | 12 | rs142788814;rs531578657; rs549698414 | 0.329463 | 0.303158 | 0.314337 | 0.390164 |
| cg23445957 | 5.17E-04 | 17 | rs73283516 | 0.363365 | 0.334227 | 0.321127 | 0.47069 |
| cg16491701 | 5.17E-04 | 17 | rs142615869;rs181182738; rs150991777 | 0.236207 | 0.2113 | 0.217134 | 0.299615 |
| cg08215000 | 5.17E-04 | 12 |  | 0.312526 | 0.288932 | 0.291985 | 0.363654 |
| cg16207944 | 5.17E-04 | 14 | rs567750135;rs538387441; rs377029212;rs578092570; rs539313126;rs554466660; rs374897125;rs367952898; rs561370391;rs573390662; rs543935977 | 0.470919 | 0.443722 | 0.440646 | 0.511444 |
| cg11285173 | 5.17E-04 | 6 | rs566367003;rs565166675 | 0.218756 | 0.198417 | 0.185719 | 0.293005 |
| cg14352601 | 5.16E-04 | 7 | rs77273326;rs183399796 | 0.242971 | 0.221141 | 0.219262 | 0.333229 |
| cg13547913 | 5.16E-04 | 4 |  | 0.463183 | 0.437991 | 0.455586 | 0.535116 |
| cg07805500 | 5.16E-04 | 6 | rs564075478;rs147679501 | 0.205578 | 0.179046 | 0.188736 | 0.271044 |
| cg04395593 | 5.16E-04 | 9 | rs551207361;rs564727588 | 0.260074 | 0.23808 | 0.227754 | 0.317881 |
| cg02847356 | 5.16E-04 | 7 | rs528229480;rs546392235 | 0.263321 | 0.234442 | 0.233395 | 0.331782 |
| cg04753874 | 5.15E-04 | 12 | rs569876255 | 0.347744 | 0.295984 | 0.288502 | 0.386385 |
| cg17256506 | 5.14E-04 | 10 |  | 0.181879 | 0.159607 | 0.162181 | 0.232206 |
| cg16846069 | 5.14E-04 | 12 | rs553145312;rs146146516; rs148802796;rs182195643 | 0.311227 | 0.284478 | 0.286933 | 0.375877 |
| cg03998264 | 5.14E-04 | 12 | rs574334114 | 0.256265 | 0.216308 | 0.213966 | 0.323113 |
| cg06510708 | 5.14E-04 | 3 |  | 0.396186 | 0.368759 | 0.374481 | 0.45313 |
| cg14825905 | 5.14E-04 | 10 |  | 0.729555 | 0.69936 | 0.711351 | 0.764708 |
| cg23013850 | 5.13E-04 | 2 | rs74731790;rs201679865; rs147752707 | 0.226784 | 0.191661 | 0.193195 | 0.284108 |
| cg10946356 | 5.13E-04 | 1 | rs570636993;rs138972355; rs34466404 | 0.332882 | 0.294136 | 0.291059 | 0.390784 |
| cg22526531 | 5.13E-04 | 1 | rs147017356;rs142007809; rs200635190 | 0.393903 | 0.35485 | 0.362002 | 0.461976 |
| cg18217480 | 5.13E-04 | 17 | rs192071349 | 0.325066 | 0.299527 | 0.285258 | 0.388006 |
| cg25270225 | 5.13E-04 | 1 | rs186236919;rs536229966; rs556229007;rs569684643 | 0.333133 | 0.307523 | 0.292119 | 0.40158 |
| cg25084760 | 5.13E-04 | 13 |  | 0.298192 | 0.262413 | 0.260801 | 0.397343 |
| cg19055828 | 5.12E-04 | 12 | rs541165932 | 0.298185 | 0.272858 | 0.256552 | 0.395535 |
| cg09082427 | 5.12E-04 | 9 | rs569991051 | 0.69775 | 0.674082 | 0.689247 | 0.76953 |
| cg17099692 | 5.12E-04 | 8 | rs549345635;rs548220589 | 0.285227 | 0.256707 | 0.242404 | 0.358593 |
| cg10098414 | 5.12E-04 | 4 | rs529581015 | 0.334806 | 0.306255 | 0.307385 | 0.430809 |
| cg13520922 | 5.11E-04 | 11 | rs192652420;rs850285 | 0.346089 | 0.318302 | 0.30532 | 0.419599 |
| cg05696274 | 5.11E-04 | 13 | rs530151225;rs543912461; rs41502045 | 0.331884 | 0.297312 | 0.298328 | 0.39922 |
| cg04382540 | 5.11E-04 | 20 |  | 0.262687 | 0.236059 | 0.226354 | 0.327986 |
| cg02105211 | 5.11E-04 | 3 | rs77966080;rs530117140; rs370122488 | 0.401818 | 0.367911 | 0.370756 | 0.489773 |
| cg01627252 | 5.11E-04 | 2 | rs542626294;rs556844251 | 0.334502 | 0.30675 | 0.314752 | 0.411323 |
| cg05265936 | 5.09E-04 | 16 |  | 0.58987 | 0.566438 | 0.569422 | 0.625123 |
| cg05104574 | 5.08E-04 | 8 | rs115566613 | 0.270015 | 0.246376 | 0.24005 | 0.343425 |
| cg02646429 | 5.08E-04 | 9 | rs574558339 | 0.296111 | 0.271914 | 0.267579 | 0.350187 |
| cg22556909 | 5.07E-04 | 2 | rs539761900;rs553177231 | 0.321257 | 0.285254 | 0.283443 | 0.407474 |
| cg03361738 | 5.07E-04 | 9 | rs148634136;rs144441671 | 0.421052 | 0.383857 | 0.394313 | 0.495959 |
| cg26667508 | 5.06E-04 | 10 | rs796667;rs112718794 | 0.202289 | 0.176433 | 0.179197 | 0.271991 |
| cg15037823 | 5.06E-04 | 3 |  | 0.271532 | 0.238797 | 0.24794 | 0.338925 |
| cg22595920 | 5.06E-04 | 1 |  | 0.238737 | 0.197551 | 0.203318 | 0.305312 |
| cg04071967 | 5.05E-04 | 17 | rs560138131;rs191581276 | 0.287356 | 0.259886 | 0.260513 | 0.357205 |
| cg00235484 | 5.05E-04 | 6 | rs547842916;rs145123424; rs147601188 | 0.299556 | 0.272005 | 0.270758 | 0.392794 |
| cg02573357 | 5.02E-04 | 1 | rs4951180;rs4951181 | 0.236335 | 0.208719 | 0.209643 | 0.299808 |
| cg21207061 | 5.02E-04 | 8 | rs192996651;rs150741922 | 0.363784 | 0.33689 | 0.351993 | 0.425135 |
| cg15300024 | 5.02E-04 | 2 | rs190259661;rs558098184; rs577976329 | 0.234779 | 0.203109 | 0.198817 | 0.284805 |
| cg10831427 | 5.01E-04 | 2 | rs181295042;rs545603712 | 0.329706 | 0.287447 | 0.29585 | 0.392853 |
| cg26746309 | 5.00E-04 | 10 | rs111635996;rs372452324; rs199657565;rs112671013; rs201208911;rs544148786 | 0.232185 | 0.203889 | 0.198328 | 0.283526 |
| cg14632793 | 4.98E-04 | 2 | rs117310549;rs560173801 | 0.287151 | 0.260431 | 0.255355 | 0.365898 |
| cg13933685 | 4.98E-04 | 2 | rs187148874;rs528193348 | 0.419874 | 0.394145 | 0.384893 | 0.52102 |
| cg23025223 | 4.98E-04 | 5 | rs114226488;rs141257556 | 0.12025 | 0.09729 | 0.099644 | 0.134792 |
| cg24621453 | 4.97E-04 | 1 | rs78738300;rs544772852; rs536428869 | 0.283554 | 0.261703 | 0.253496 | 0.353943 |
| cg11361809 | 4.95E-04 | 20 | rs185861952;rs190033144; rs78157940 | 0.291561 | 0.259476 | 0.246573 | 0.349916 |
| cg05802929 | 4.95E-04 | 3 | rs539695333 | 0.346419 | 0.325155 | 0.31886 | 0.431829 |
| cg10217365 | 4.95E-04 | 19 | rs368552619 | 0.287694 | 0.260295 | 0.256722 | 0.33736 |
| cg18893685 | 4.95E-04 | 9 |  | 0.304208 | 0.277154 | 0.269973 | 0.399791 |
| cg10418276 | 4.94E-04 | 18 | rs138360865 | 0.34869 | 0.326053 | 0.333928 | 0.471331 |
| cg05257483 | 4.94E-04 | 21 | rs190789053;rs543264014; rs561956777;rs547739962 | 0.236306 | 0.209181 | 0.199072 | 0.314425 |
| cg00769356 | 4.93E-04 | 4 | rs144047138 | 0.302048 | 0.281346 | 0.278608 | 0.392947 |
| cg00431806 | 4.93E-04 | 1 | rs531573772;rs78273755 | 0.228023 | 0.194253 | 0.192521 | 0.299645 |
| cg07686393 | 4.90E-04 | 17 |  | 0.234582 | 0.210685 | 0.195276 | 0.29303 |
| cg20267218 | 4.90E-04 | 6 |  | 0.246328 | 0.224433 | 0.218097 | 0.289856 |
| cg12492273 | 4.89E-04 | 7 | rs191152361;rs181123170; rs62444921 | 0.286427 | 0.265693 | 0.277362 | 0.358201 |
| cg20661985 | 4.89E-04 | 20 | rs185248403;rs548049002 | 0.298452 | 0.255946 | 0.259862 | 0.382592 |
| cg08856354 | 4.88E-04 | 6 | rs538887550;rs553832747 | 0.257398 | 0.218132 | 0.2015 | 0.331844 |
| cg07438291 | 4.88E-04 | 21 | rs541570661 | 0.265879 | 0.243822 | 0.228626 | 0.338942 |
| cg24640736 | 4.88E-04 | 5 | rs551116020;rs188187229; rs4242043;rs145767301; rs180899565 | 0.269832 | 0.243016 | 0.247457 | 0.3362 |
| cg02876904 | 4.87E-04 | 16 |  | 0.723266 | 0.699674 | 0.701137 | NA |
| cg09126588 | 4.85E-04 | 5 | rs190604078;rs569406991 | 0.287311 | 0.257945 | 0.258601 | 0.354102 |
| cg15132282 | 4.85E-04 | 2 | rs566036294;rs146723570; rs551695501 | 0.251305 | 0.228823 | 0.229173 | 0.323232 |
| cg02372723 | 4.84E-04 | 11 | rs138121546;rs142872801; rs535872217 | 0.403778 | 0.370927 | 0.36514 | 0.492489 |
| cg09912228 | 4.84E-04 | 1 | rs559860378;rs79745684 | 0.318616 | 0.280993 | 0.264955 | 0.38375 |
| cg14882828 | 4.83E-04 | 5 | rs60927035 | 0.298603 | 0.258076 | 0.259014 | 0.385637 |
| cg24907772 | 4.83E-04 | 9 | rs7857411;rs113939123 | 0.235935 | 0.214978 | 0.211625 | 0.318483 |
| cg19242228 | 4.83E-04 | 20 | rs192191592;rs375269672 | 0.256804 | 0.226471 | 0.223857 | 0.313892 |
| cg07579732 | 4.82E-04 | 12 |  | 0.285697 | 0.247356 | 0.246815 | 0.360104 |
| cg00168694 | 4.82E-04 | 21 | rs535157069;rs139406249 | 0.274587 | 0.251424 | 0.240716 | 0.351211 |
| cg16636227 | 4.82E-04 | 10 | rs542071141;rs140079000; rs572406437;rs191875424; rs77791001;rs550279153 | 0.36255 | 0.342127 | 0.332607 | 0.424783 |
| cg20905910 | 4.81E-04 | 3 | rs78355824;rs559254604 | 0.280253 | 0.253387 | 0.246907 | 0.356901 |
| cg20891765 | 4.80E-04 | 2 | rs113322271 | 0.306267 | 0.28559 | 0.28703 | 0.403618 |
| cg11968956 | 4.80E-04 | 6 | rs533084725 | 0.287263 | 0.246028 | 0.247694 | 0.369488 |
| cg07607891 | 4.80E-04 | 5 | rs558764268;rs575656785; rs544724210;rs112927680; rs574928626 | 0.17919 | 0.149999 | 0.143644 | 0.211196 |
| cg22034801 | 4.80E-04 | 2 |  | 0.247433 | 0.220094 | 0.205659 | 0.325888 |
| cg09494673 | 4.78E-04 | 3 | rs548022091;rs73198819; rs77082730;rs577316612 | 0.289859 | 0.260512 | 0.260648 | 0.387008 |
| cg04331199 | 4.76E-04 | 1 | rs146378211;rs75300309; rs114297094;rs139746046 | 0.470797 | 0.4392 | 0.44404 | 0.556585 |
| cg13240557 | 4.76E-04 | 3 |  | 0.344377 | 0.313837 | 0.311127 | 0.420175 |
| cg08270236 | 4.75E-04 | 4 |  | 0.421575 | 0.379875 | 0.379955 | 0.499177 |
| cg14584535 | 4.75E-04 | 9 | rs34751912 | 0.261888 | 0.230304 | 0.223501 | 0.324006 |
| cg19014318 | 4.75E-04 | 11 | rs569537695;rs542770920; rs554808986 | 0.289167 | 0.258026 | 0.257121 | 0.369996 |
| cg16515952 | 4.75E-04 | 6 | rs186986442;rs561392290 | 0.225831 | 0.196131 | 0.191729 | 0.305148 |
| cg13158272 | 4.74E-04 | 14 | rs117397647;rs151096382; rs141060431 | 0.573673 | 0.54619 | 0.552381 | 0.62719 |
| cg06783396 | 4.74E-04 | 4 | rs151083192 | 0.268679 | 0.240883 | 0.240789 | 0.330303 |
| cg04304338 | 4.73E-04 | 5 | rs546805795 | 0.592753 | 0.561603 | 0.573629 | 0.648907 |
| cg10613822 | 4.73E-04 | 15 | rs182932584 | 0.260343 | 0.235235 | 0.232077 | 0.335815 |
| cg00274937 | 4.73E-04 | 5 | rs569279977 | 0.269393 | 0.242619 | 0.236278 | 0.328943 |
| cg24086675 | 4.72E-04 | 14 | rs191171851;rs546955260 | 0.317023 | 0.284248 | 0.281119 | 0.391404 |
| cg19530603 | 4.72E-04 | 6 | rs535950288;rs552998169 | 0.266527 | 0.241441 | 0.238319 | 0.347741 |
| cg13918640 | 4.71E-04 | 9 | rs542630269;rs180950564 | 0.321105 | 0.299432 | 0.285757 | 0.384584 |
| cg19233088 | 4.71E-04 | 2 | rs370904017;rs75272274; rs4309533;rs548510290; rs79631062 | 0.365288 | 0.33996 | 0.333009 | 0.444917 |
| cg00776942 | 4.70E-04 | 15 | rs534421688 | 0.318945 | 0.293315 | 0.281571 | 0.407963 |
| cg16628676 | 4.70E-04 | 22 | rs111503634 | 0.319323 | 0.295551 | 0.294452 | 0.365787 |
| cg01014262 | 4.70E-04 | 16 | rs568124734 | 0.188068 | 0.166681 | 0.168272 | 0.25062 |
| cg26613073 | 4.69E-04 | 13 | rs186429329 | 0.260756 | 0.235961 | 0.227385 | 0.310507 |
| cg18120029 | 4.69E-04 | 2 | rs559159507;rs80188710; rs545611384 | 0.23408 | 0.212922 | 0.213193 | 0.296502 |
| cg12412636 | 4.69E-04 | 14 | rs186516581 | 0.295501 | 0.265561 | 0.263307 | 0.362244 |
| cg07577228 | 4.69E-04 | 1 | rs112088860 | 0.318169 | 0.288264 | 0.283763 | 0.414824 |
| cg06120750 | 4.69E-04 | 21 |  | 0.239607 | 0.216013 | 0.210995 | 0.297726 |
| cg16154454 | 4.67E-04 | 13 | rs535414918;rs557002651; rs377065284 | 0.205478 | 0.180343 | 0.185212 | 0.266885 |
| cg06719445 | 4.67E-04 | 14 | rs150222710;rs555249992; rs575545097;rs557531002 | 0.262872 | 0.226715 | 0.221878 | 0.361333 |
| cg20922407 | 4.66E-04 | 2 |  | 0.279332 | 0.255129 | 0.242956 | 0.362697 |
| cg26438215 | 4.65E-04 | 6 | rs112094516;rs575838615 | 0.333121 | 0.300229 | 0.294237 | 0.41803 |
| cg00361393 | 4.65E-04 | 9 | rs369641323;rs559393102 | 0.291079 | 0.267489 | 0.254224 | 0.363237 |
| cg13893555 | 4.65E-04 | 9 | rs77747293 | 0.502193 | 0.481771 | 0.494307 | 0.583426 |
| cg18688188 | 4.65E-04 | 20 | rs570965722;rs112950899 | 0.357656 | 0.318764 | 0.321385 | 0.435016 |
| cg22000700 | 4.65E-04 | 8 | rs185879997;rs10100417; rs553017740;rs566506122; rs78755489;rs373814975; rs112230921 | 0.229016 | 0.204334 | 0.197337 | 0.284204 |
| cg00435244 | 4.64E-04 | 8 | rs190133231;rs113201227; rs117029632;rs932268; rs555653963 | 0.586586 | 0.556392 | 0.574552 | 0.637705 |
| cg23788468 | 4.63E-04 | 5 | rs576927600 | 0.28978 | 0.259836 | 0.265434 | 0.369795 |
| cg02871300 | 4.63E-04 | 3 |  | 0.261252 | 0.226037 | 0.221288 | 0.320729 |
| cg20932630 | 4.63E-04 | 4 | rs538826202;rs146297798 | 0.286076 | 0.257798 | 0.252271 | 0.364584 |
| cg00980622 | 4.62E-04 | 14 |  | 0.343792 | 0.300018 | 0.305218 | 0.404468 |
| cg04172297 | 4.62E-04 | 20 | rs145557040;rs555932093; rs187123987;rs150633655 | 0.806299 | 0.784252 | 0.79741 | 0.796136 |
| cg08924923 | 4.62E-04 | 13 |  | 0.277336 | 0.240391 | 0.238532 | 0.358688 |
| cg26265624 | 4.62E-04 | 20 | rs79813406 | 0.337952 | 0.298827 | 0.295495 | 0.413072 |
| cg12269320 | 4.61E-04 | 1 | rs571279931;rs377423170; rs557215728 | 0.648647 | 0.623039 | 0.626105 | 0.696701 |
| cg26300092 | 4.61E-04 | 2 | rs146286404;rs569672959; rs193105766 | 0.311673 | 0.272969 | 0.267884 | 0.404209 |
| cg01157024 | 4.61E-04 | 1 | rs574314545;rs550971784 | 0.249687 | 0.219456 | 0.206579 | 0.324472 |
| cg17591980 | 4.61E-04 | 9 | rs565548796 | 0.282181 | 0.247908 | 0.246134 | 0.366768 |
| cg01811355 | 4.60E-04 | 8 | rs183524928 | 0.346651 | 0.324457 | 0.31112 | 0.437393 |
| cg07547426 | 4.59E-04 | 15 | rs561031456;rs76997344; rs146647797;rs185408427; rs375287718;rs60294887 | 0.239101 | 0.215799 | 0.205991 | 0.288853 |
| cg16395183 | 4.59E-04 | 1 | rs535878761;rs558541368 | 0.381409 | 0.357498 | 0.369724 | 0.460236 |
| cg12326440 | 4.58E-04 | 10 | rs113043328;rs150202439 | 0.438551 | 0.408693 | 0.426033 | 0.500081 |
| cg18731680 | 4.57E-04 | 22 | rs576328138 | 0.267016 | 0.24322 | 0.238258 | 0.337729 |
| cg13229972 | 4.57E-04 | 1 | rs570392350;rs529533734; rs549368252 | 0.276973 | 0.249495 | 0.25223 | 0.339107 |
| cg25618372 | 4.57E-04 | 17 |  | 0.499471 | 0.468462 | 0.468984 | 0.540247 |
| cg11136040 | 4.57E-04 | 5 | rs74740551 | 0.284111 | 0.253841 | 0.244189 | 0.370538 |
| cg10260093 | 4.57E-04 | 22 | rs572994675;rs540287328 | 0.235729 | 0.200321 | 0.197533 | 0.334938 |
| cg13358038 | 4.56E-04 | 12 | rs201323764 | 0.279204 | 0.252713 | 0.258217 | 0.348567 |
| cg04748191 | 4.55E-04 | 6 | rs534600748;rs794791 | 0.278921 | 0.246576 | 0.25789 | 0.362103 |
| cg11519751 | 4.55E-04 | 8 | rs34869758;rs571505044 | 0.253236 | 0.228527 | 0.211477 | 0.330631 |
| cg09702791 | 4.55E-04 | 3 | rs556892909 | 0.211755 | 0.174404 | 0.171994 | 0.253361 |
| cg08789503 | 4.55E-04 | 17 | rs549160802;rs567987204; rs534963293;rs34017632 | 0.271705 | 0.23301 | 0.234117 | 0.359759 |
| cg13242717 | 4.54E-04 | 18 | rs550045262;rs568156688; rs535454077 | 0.332614 | 0.303932 | 0.290343 | 0.406482 |
| cg08507334 | 4.53E-04 | 13 | rs532076499;rs545063571 | 0.262409 | 0.238055 | 0.229685 | 0.335731 |
| cg23686408 | 4.52E-04 | 1 |  | 0.457386 | 0.432502 | 0.428326 | 0.523841 |
| cg02699562 | 4.52E-04 | 15 |  | 0.273619 | 0.246611 | 0.231868 | 0.326687 |
| cg18934187 | 4.51E-04 | 18 | rs551995440;rs570492909; rs537465006;rs549395336 | 0.390046 | 0.368856 | 0.372129 | 0.487878 |
| cg05261452 | 4.51E-04 | 3 | rs182416491;rs549768339 | 0.357987 | 0.31405 | 0.310094 | 0.448045 |
| cg08305295 | 4.51E-04 | 5 | rs537446597 | 0.728603 | 0.707032 | 0.726517 | 0.777899 |
| cg26766847 | 4.51E-04 | 10 | rs546682039;rs190868537; rs535658088;rs550348021 | 0.409221 | 0.381612 | 0.393027 | 0.501658 |
| cg18727350 | 4.51E-04 | 20 |  | 0.243951 | 0.216847 | 0.210831 | 0.289333 |
| cg21223535 | 4.51E-04 | 6 | rs560186339;rs186357649 | 0.283524 | 0.260408 | 0.244651 | 0.357777 |
| cg07878585 | 4.50E-04 | 6 | rs149293458;rs370643688 | 0.28696 | 0.265101 | 0.26515 | 0.363149 |
| cg12189405 | 4.50E-04 | 15 | rs368602982 | 0.223859 | 0.201927 | 0.191228 | 0.301246 |
| cg03337475 | 4.48E-04 | 8 | rs376066750;rs115883652; rs562462282 | 0.303162 | 0.278132 | 0.271293 | 0.382367 |
| cg16469266 | 4.48E-04 | 1 | rs77205476;rs543870620 | 0.335442 | 0.310314 | 0.310338 | 0.407684 |
| cg20589873 | 4.47E-04 | 22 | rs561468132 | 0.287014 | 0.254729 | 0.243032 | 0.339294 |
| cg02731880 | 4.46E-04 | 15 | rs571124908;rs11857157; rs553033408 | 0.280656 | 0.25371 | 0.24311 | 0.347221 |
| cg20077668 | 4.45E-04 | 12 |  | 0.366517 | 0.334616 | 0.345553 | 0.452888 |
| cg08257297 | 4.45E-04 | 15 | rs541924001;rs563787649 | 0.504831 | 0.482252 | 0.496018 | 0.570396 |
| cg09180788 | 4.44E-04 | 15 | rs535890928;rs554086972; rs148574508;rs190241540 | 0.285062 | 0.256433 | 0.260309 | 0.350514 |
| cg05583921 | 4.44E-04 | 3 | rs339723 | 0.458959 | 0.422552 | 0.403715 | 0.564804 |
| cg00855703 | 4.42E-04 | 2 | rs11684917;rs143632904 ;rs563251384 | 0.304564 | 0.275015 | 0.26817 | 0.395502 |
| cg00847359 | 4.42E-04 | 3 | rs59197998;rs60847280 | 0.269567 | 0.240736 | 0.230695 | 0.348029 |
| cg19261949 | 4.42E-04 | 22 | rs549336328;rs567411814 | 0.296188 | 0.26605 | 0.257916 | 0.359481 |
| cg10961050 | 4.42E-04 | 9 | rs147737473 | 0.230753 | 0.202978 | 0.212025 | 0.285098 |
| cg06763402 | 4.42E-04 | 1 | rs183456882;rs547176859 | 0.203794 | 0.181234 | 0.17781 | 0.257416 |
| cg12380169 | 4.41E-04 | 13 | rs143718904;rs571599547 | 0.277873 | 0.246186 | 0.251161 | 0.351557 |
| cg16673748 | 4.41E-04 | 8 | rs565470957 | 0.253912 | 0.225819 | 0.216553 | 0.31948 |
| cg04947907 | 4.40E-04 | 12 | rs7136989;rs75376654; rs540194562;rs560389613; rs532220879 | 0.180481 | 0.142232 | 0.138653 | 0.209042 |
| cg04977356 | 4.39E-04 | 11 |  | 0.298628 | 0.273127 | 0.260956 | 0.400845 |
| cg22687766 | 4.39E-04 | 1 | rs542519077 | 0.323879 | 0.298525 | 0.30236 | 0.373432 |
| cg07390377 | 4.39E-04 | 6 | rs143565908;rs186497286; rs147138804;rs201567851 | 0.257929 | 0.229383 | 0.238472 | 0.324195 |
| cg26238134 | 4.38E-04 | 20 |  | 0.333898 | 0.302199 | 0.298432 | 0.405332 |
| cg01651448 | 4.37E-04 | 17 | rs563216673 | 0.221484 | 0.19556 | 0.198142 | 0.284661 |
| cg26776551 | 4.37E-04 | 13 | rs187016941;rs574462168 | 0.309614 | 0.283637 | 0.285125 | 0.370417 |
| cg00290637 | 4.37E-04 | 12 |  | 0.328211 | 0.305402 | 0.299654 | 0.393021 |
| cg06755438 | 4.37E-04 | 2 | rs544777059;rs559849480 | 0.320463 | 0.299958 | 0.293707 | 0.394097 |
| cg15638279 | 4.36E-04 | 4 | rs564250222;rs533313048; rs549930002;rs147568780; rs529159175;rs1974991 | 0.443175 | 0.421535 | 0.423796 | 0.51404 |
| cg04238983 | 4.36E-04 | 11 | rs112898102 | 0.259354 | 0.234159 | 0.248004 | 0.333429 |
| cg20741644 | 4.36E-04 | 2 | rs73984347;rs140400345; rs147975645;rs149888279; rs149023402;rs566985824; rs200737130 | 0.247016 | 0.225771 | 0.21851 | 0.314214 |
| cg16905911 | 4.35E-04 | 20 | rs79796140;rs148522302; rs544339379;rs41308715 | 0.305001 | 0.274045 | 0.273629 | 0.354047 |
| cg14363249 | 4.35E-04 | 9 | rs138374100;rs550027074 | 0.289461 | 0.256345 | 0.257948 | 0.366471 |
| cg24811892 | 4.35E-04 | 15 | rs546064167;rs375563473 | 0.27741 | 0.252721 | 0.241002 | 0.340148 |
| cg21645341 | 4.34E-04 | 13 | rs553227637 | 0.314527 | 0.287501 | 0.284516 | 0.399401 |
| cg15920724 | 4.34E-04 | 9 | rs561925421 | 0.204082 | 0.175144 | 0.180957 | 0.240662 |
| cg01685428 | 4.34E-04 | 20 | rs564002430;rs182597796; rs111354845;rs566651924 | 0.295861 | 0.252442 | 0.247779 | 0.38229 |
| cg18478667 | 4.33E-04 | 4 | rs145686612;rs34691452; rs527314565 | 0.15484 | 0.129854 | 0.1368 | 0.200746 |
| cg05655915 | 4.33E-04 | 17 | rs185221963;rs577779673; rs544917720;rs59649937 | 0.259576 | 0.221045 | 0.21627 | 0.338085 |
| cg23639019 | 4.32E-04 | 18 | rs77084629 | 0.329836 | 0.295512 | 0.307381 | 0.391808 |
| cg10136859 | 4.31E-04 | 1 | rs572092479 | 0.240292 | 0.216895 | 0.205637 | 0.293797 |
| cg26058030 | 4.31E-04 | 12 | rs118184961 | 0.277624 | 0.257098 | 0.241228 | 0.34007 |
| cg05215498 | 4.31E-04 | 17 | rs548930281;rs143144242 | 0.340778 | 0.302422 | 0.302256 | 0.430003 |
| cg08900384 | 4.30E-04 | 11 | rs567402996;rs528300416 | 0.295707 | 0.266377 | 0.25848 | 0.348385 |
| cg09328789 | 4.30E-04 | 1 | rs185235173 | 0.318135 | 0.286787 | 0.291285 | 0.390167 |
| cg19819715 | 4.29E-04 | 2 | rs567197466 | 0.328626 | 0.305777 | 0.300474 | 0.40743 |
| cg11776255 | 4.28E-04 | 17 |  | 0.378487 | 0.348713 | 0.360783 | 0.461318 |
| cg18821474 | 4.28E-04 | 4 | rs568610662;rs575215811 | 0.406721 | 0.381474 | 0.376268 | 0.444539 |
| cg10133935 | 4.28E-04 | 19 | rs563843715 | 0.409471 | 0.383291 | 0.387817 | 0.472672 |
| cg03811164 | 4.28E-04 | 1 | rs150310907 | 0.379801 | 0.35354 | 0.367541 | 0.454936 |
| cg23470684 | 4.28E-04 | 15 | rs533114954 | 0.341958 | 0.310451 | 0.309358 | 0.411876 |
| cg22586569 | 4.27E-04 | 7 | rs549159222;rs567938578; rs182017757 | 0.676042 | 0.652791 | 0.662754 | 0.699326 |
| cg18997983 | 4.27E-04 | 5 | rs150079276;rs578087100; rs545697710 | 0.281217 | 0.255042 | 0.259936 | 0.347005 |
| cg19772215 | 4.27E-04 | 11 | rs201261198;rs368881649 | 0.308689 | 0.283151 | 0.277503 | 0.38543 |
| cg24297706 | 4.27E-04 | 11 |  | 0.307396 | 0.275049 | 0.272798 | 0.381194 |
| cg15010903 | 4.27E-04 | 17 |  | 0.357464 | 0.330654 | 0.330656 | 0.432155 |
| cg12929208 | 4.25E-04 | 10 | rs564468109;rs533543120; rs550430390 | 0.238667 | 0.217232 | 0.214537 | 0.307771 |
| cg25521400 | 4.25E-04 | 2 | rs138354282 | 0.286036 | 0.262509 | 0.2486 | 0.354064 |
| cg10493843 | 4.25E-04 | 20 | rs550619604 | 0.267482 | 0.234396 | 0.229717 | 0.324691 |
| cg02287007 | 4.24E-04 | 6 | rs566033047;rs530355058 | 0.22392 | 0.189888 | 0.188785 | 0.278439 |
| cg05480073 | 4.23E-04 | 5 | rs79043187;rs576317247; rs116326997 | 0.302348 | 0.269465 | 0.261173 | 0.374069 |
| cg19306296 | 4.23E-04 | 11 |  | 0.251608 | 0.230259 | 0.224222 | 0.346676 |
| cg10703154 | 4.22E-04 | 2 | rs183271784;rs564145235; rs75011328;rs187792474 | 0.341885 | 0.306659 | 0.291395 | 0.421967 |
| cg09086039 | 4.22E-04 | 3 | rs192330338;rs112227491 | 0.244541 | 0.209604 | 0.211001 | 0.321094 |
| cg00873414 | 4.22E-04 | 6 |  | 0.17645 | 0.150521 | 0.151645 | 0.220284 |
| cg20152841 | 4.22E-04 | 19 | rs568353329;rs535422301 | 0.438239 | 0.414869 | 0.421552 | 0.487137 |
| cg07125403 | 4.22E-04 | 2 | rs547487372 | 0.296581 | 0.261135 | 0.254086 | 0.362493 |
| cg26877678 | 4.21E-04 | 17 | rs527954698;rs552924026; rs571007837 | 0.286494 | 0.261144 | 0.256581 | 0.367125 |
| cg06419663 | 4.21E-04 | 12 | rs142505152;rs114040911 | 0.228187 | 0.195945 | 0.205733 | 0.285594 |
| cg05670158 | 4.20E-04 | 2 | rs573743648;rs143647656 | 0.273291 | 0.252909 | 0.235676 | 0.368125 |
| cg26175771 | 4.20E-04 | 19 | rs148495731 | 0.337328 | 0.312269 | 0.313614 | 0.405854 |
| cg16205262 | 4.20E-04 | 15 |  | 0.278862 | 0.249105 | 0.245666 | 0.37869 |
| cg00385640 | 4.20E-04 | 8 | rs528504521 | 0.287 | 0.264052 | 0.261721 | 0.359187 |
| cg13348907 | 4.19E-04 | 15 |  | 0.238903 | 0.216234 | 0.198626 | 0.298817 |
| cg25486993 | 4.18E-04 | 4 | rs566766332 | 0.213314 | 0.187153 | 0.190014 | 0.273377 |
| cg11638399 | 4.18E-04 | 8 | rs537644426;rs554879338 | 0.297957 | 0.27288 | 0.259902 | 0.363437 |
| cg06173720 | 4.17E-04 | 7 | rs542207382;rs547734045; rs531074627 | 0.253537 | 0.219788 | 0.221608 | 0.295874 |
| cg16163847 | 4.17E-04 | 13 | rs9591413 | 0.300924 | 0.264192 | 0.262639 | 0.369215 |
| cg15704662 | 4.17E-04 | 8 | rs5894881;rs113568837 | 0.355399 | 0.315316 | 0.327396 | 0.430794 |
| cg13183099 | 4.16E-04 | 1 | rs6670711;rs79576104 | 0.387251 | 0.362068 | 0.349645 | 0.479022 |
| cg11579421 | 4.16E-04 | 10 | rs550114338;rs570231812 | 0.271406 | 0.24338 | 0.239317 | 0.348446 |
| cg22265539 | 4.16E-04 | 20 |  | 0.243045 | 0.204676 | 0.199386 | 0.310899 |
| cg19925518 | 4.16E-04 | 10 | rs564159546;rs11202972 | 0.496087 | 0.46609 | 0.476622 | 0.573545 |
| cg06966738 | 4.15E-04 | 2 | rs556153709;rs576210573; rs545181571;rs10153736 | 0.325632 | 0.289938 | 0.302993 | 0.424334 |
| cg06852746 | 4.14E-04 | 10 | rs574862320;rs537762034 | 0.321864 | 0.282581 | 0.272612 | 0.403133 |
| cg09708200 | 4.14E-04 | 11 | rs553178030;rs577654820 | 0.488141 | 0.454145 | 0.463636 | 0.546117 |
| cg05702548 | 4.13E-04 | 15 | rs543413605;rs181360750; rs10438418 | 0.34534 | 0.325118 | 0.329533 | 0.418274 |
| cg10056106 | 4.12E-04 | 11 | rs531336982;rs12271419; rs562005356;rs527624359 | 0.2785 | 0.246475 | 0.227519 | 0.349704 |
| cg14898946 | 4.12E-04 | 1 | rs72973087;rs141800274; rs537033009;rs556812682 | 0.222113 | 0.195561 | 0.190586 | 0.272722 |
| cg03278108 | 4.11E-04 | 9 | rs551548176;rs193229338; rs527400313 | 0.392349 | 0.357673 | 0.359576 | 0.474722 |
| cg06278080 | 4.10E-04 | 15 | rs541926693;rs528412244 | 0.320665 | 0.30056 | 0.284061 | 0.409497 |
| cg02327530 | 4.10E-04 | 7 |  | 0.27472 | 0.244805 | 0.245821 | 0.337501 |
| cg10856032 | 4.08E-04 | 1 | rs147362560;rs545500537 | 0.208094 | 0.186661 | 0.176814 | 0.263316 |
| cg12906062 | 4.08E-04 | 13 | rs557012195;rs576999945 | 0.32304 | 0.297348 | 0.30277 | 0.399663 |
| cg14950616 | 4.08E-04 | 1 | rs573079838;rs540025446; rs564755709 | 0.310783 | 0.286379 | 0.27457 | 0.38359 |
| cg25497409 | 4.08E-04 | 14 | rs530046731;rs540153463 | 0.326254 | 0.284677 | 0.293664 | 0.410798 |
| cg04283041 | 4.06E-04 | 2 | rs534720663 | 0.217171 | 0.192692 | 0.191863 | 0.306789 |
| cg09644712 | 4.06E-04 | 2 | rs559861941 | 0.312984 | 0.283504 | 0.270284 | 0.398114 |
| cg06180354 | 4.06E-04 | 12 | rs570442132 | 0.473197 | 0.447913 | 0.451163 | 0.560571 |
| cg15850190 | 4.06E-04 | 6 | rs546276160;rs572044183 | 0.195776 | 0.168105 | 0.175317 | 0.251897 |
| cg03937508 | 4.05E-04 | 3 | rs202046650;rs528292587 | 0.310509 | 0.276201 | 0.279228 | 0.384008 |
| cg04945216 | 4.05E-04 | 7 | rs118073034;rs112520705; rs17133072;rs544076040 | 0.217824 | 0.197591 | 0.19663 | 0.282627 |
| cg06107228 | 4.04E-04 | 1 | rs543848996 | 0.259072 | 0.227642 | 0.221413 | 0.333743 |
| cg22176763 | 4.04E-04 | 5 |  | 0.383335 | 0.357711 | 0.349266 | 0.462508 |
| cg02659916 | 4.03E-04 | 21 | rs183582847;rs536674738; rs531729522 | 0.268558 | 0.247826 | 0.2307 | 0.331525 |
| cg26561681 | 4.02E-04 | 1 |  | 0.234617 | 0.207611 | 0.21386 | 0.318513 |
| cg03390370 | 4.02E-04 | 2 | rs371194983;rs574170961; rs544728295;rs143437740; rs577781044 | 0.266113 | 0.224904 | 0.235762 | 0.320644 |
| cg05569131 | 4.01E-04 | 6 | rs139292480;rs149991115; rs9394373;rs76894359 | 0.305746 | 0.279312 | 0.285411 | 0.363975 |
| cg21477382 | 4.01E-04 | 21 | rs73901294;rs561661735 | 0.159716 | 0.131429 | 0.121142 | 0.196391 |
| cg18706710 | 4.00E-04 | 12 | rs146183012;rs368867411;r s137907685 | 0.240258 | 0.218466 | 0.204554 | 0.297302 |
| cg13584052 | 4.00E-04 | 1 | rs570675870;rs187301894; rs549832079 | 0.38211 | 0.347075 | 0.339888 | 0.465167 |
| cg23221723 | 3.99E-04 | 8 | rs190053767;rs572007407 | 0.275836 | 0.23628 | 0.255916 | 0.342618 |
| cg16910401 | 3.99E-04 | 17 | rs75902298;rs112650220 | 0.292897 | 0.2634 | 0.24969 | 0.367098 |
| cg07248440 | 3.98E-04 | 16 | rs370130406;rs117296272; rs147827344 | 0.507942 | 0.484906 | 0.488015 | 0.595344 |
| cg08773226 | 3.98E-04 | 6 | rs189145257;rs574660866; rs540478348;rs560278264 | 0.337643 | 0.304825 | 0.291918 | 0.420004 |
| cg01555113 | 3.98E-04 | 21 |  | 0.14119 | 0.119834 | 0.116471 | 0.173866 |
| cg21578596 | 3.98E-04 | 6 | rs148084922;rs564449684; rs143112264 | 0.253017 | 0.231917 | 0.221666 | 0.312975 |
| cg15312943 | 3.98E-04 | 11 | rs149838260 | 0.320207 | 0.28534 | 0.278317 | 0.390752 |
| cg08112866 | 3.98E-04 | 5 |  | 0.334094 | 0.296887 | 0.289403 | 0.426509 |
| cg15927650 | 3.97E-04 | 11 | rs555816393;rs574335600; rs535345792 | 0.257604 | 0.234163 | 0.232632 | 0.345869 |
| cg13576175 | 3.97E-04 | 17 | rs373299523;rs150501103; rs373128347;rs377602671; rs201167971;rs61748635 | 0.336794 | 0.311059 | 0.301005 | 0.412078 |
| cg25448856 | 3.95E-04 | 12 | rs187712734;rs537330520; rs192639439 | 0.326988 | 0.299854 | 0.292713 | 0.414338 |
| cg10721734 | 3.94E-04 | 16 | rs139894764;rs576664866 | 0.201588 | 0.17598 | 0.17024 | 0.250259 |
| cg09004573 | 3.93E-04 | 1 | rs550604150;rs562627557 | 0.232133 | 0.198475 | 0.195026 | 0.303733 |
| cg15553418 | 3.93E-04 | 1 |  | 0.253868 | 0.220669 | 0.215575 | 0.337013 |
| cg05239225 | 3.92E-04 | 15 | rs16940287 | 0.330873 | 0.29682 | 0.31129 | 0.403158 |
| cg01241390 | 3.91E-04 | 5 | rs143763897;rs113063421; rs538114644 | 0.322188 | 0.291902 | 0.301435 | 0.412977 |
| cg00453717 | 3.91E-04 | 11 | rs546224211;rs114922788; rs189864190 | 0.286179 | 0.260913 | 0.254252 | 0.357717 |
| cg08930993 | 3.91E-04 | 14 | rs72675450;rs185664616; rs117015060 | 0.344058 | 0.319892 | 0.31379 | 0.448588 |
| cg15712243 | 3.91E-04 | 8 |  | 0.281527 | 0.245472 | 0.247707 | 0.343833 |
| cg06576634 | 3.90E-04 | 1 | rs561111538;rs116453326; rs147171315;rs560206380 | 0.248035 | 0.221724 | 0.220518 | 0.313059 |
| cg04902811 | 3.90E-04 | 10 | rs562408915;rs554435430 | 0.235478 | 0.209008 | 0.207786 | 0.274807 |
| cg11658419 | 3.90E-04 | 12 | rs572369048;rs375591022 | 0.248466 | 0.226391 | 0.230986 | 0.304365 |
| cg14889690 | 3.90E-04 | 10 | rs527695134;rs547655530; rs570811628;rs117460753 | 0.295165 | 0.263048 | 0.253437 | 0.355624 |
| cg21301642 | 3.89E-04 | 3 |  | 0.269837 | 0.247002 | 0.252479 | 0.332282 |
| cg08840010 | 3.89E-04 | 1 | rs573638536 | 0.262367 | 0.231263 | 0.235806 | 0.345292 |
| cg10880410 | 3.89E-04 | 19 | rs531650990;rs544571054 | 0.219036 | 0.197782 | 0.184922 | 0.274663 |
| cg26878709 | 3.89E-04 | 11 | rs531484046 | 0.446148 | 0.423513 | 0.423424 | 0.512528 |
| cg15720713 | 3.89E-04 | 16 | rs75000520;rs548655867 | 0.384629 | 0.360464 | 0.357053 | 0.465361 |
| cg25576997 | 3.88E-04 | 14 | rs141106280;rs527821568 | 0.271278 | 0.24669 | 0.23416 | 0.363131 |
| cg08126118 | 3.87E-04 | 13 | rs139694159;rs114132991; rs557845947;rs577533884; rs536673064 | 0.283497 | 0.253362 | 0.252023 | 0.362845 |
| cg23835405 | 3.87E-04 | 11 |  | 0.297908 | 0.272991 | 0.257172 | 0.34653 |
| cg08760493 | 3.87E-04 | 4 | rs184376597;rs563910511; rs529522164;rs551623847; rs565713396 | 0.299253 | 0.271348 | 0.26566 | 0.360392 |
| cg13994241 | 3.86E-04 | 17 | rs150257107;rs114686333 | 0.399612 | 0.373167 | 0.369436 | 0.466265 |
| cg08106108 | 3.86E-04 | 17 | rs75658007;rs80220493; rs201597165;rs146455824; rs140748645 | 0.500872 | 0.471427 | 0.475858 | 0.571752 |
| cg21862228 | 3.86E-04 | 12 | rs191483255;rs529231137; rs142091049 | 0.25827 | 0.230941 | 0.21362 | 0.320933 |
| cg22587211 | 3.86E-04 | 6 | rs141952083 | 0.279098 | 0.257113 | 0.249655 | 0.355147 |
| cg13623749 | 3.86E-04 | 6 | rs561730999 | 0.37534 | 0.352936 | 0.360002 | 0.449065 |
| cg10012517 | 3.86E-04 | 5 | rs547534601 | 0.384218 | 0.352985 | 0.35604 | 0.465451 |
| cg08290733 | 3.85E-04 | 6 | rs570307937;rs192771695 | 0.262392 | 0.229609 | 0.222723 | 0.34652 |
| cg16872030 | 3.85E-04 | 13 | rs573455533;rs540662690 | 0.305201 | 0.278983 | 0.271669 | 0.371516 |
| cg23295942 | 3.85E-04 | 5 | rs111990800;rs547643233; rs565936409;rs536419641; rs554781184 | 0.428303 | 0.382822 | 0.363294 | 0.518642 |
| cg21421707 | 3.85E-04 | 11 | rs558304952;rs578096145; rs186838010 | 0.293448 | 0.249037 | 0.238205 | 0.366776 |
| cg06817342 | 3.85E-04 | 16 | rs758194 | 0.375546 | 0.349008 | 0.343081 | 0.447365 |
| cg26059180 | 3.84E-04 | 6 | rs551120447;rs571307116 | 0.280829 | 0.248702 | 0.249317 | 0.343062 |
| cg09091429 | 3.84E-04 | 9 | rs577442260;rs143428015 | 0.261143 | 0.2337 | 0.215632 | 0.323902 |
| cg14030146 | 3.84E-04 | 7 | rs186333110 | 0.233109 | 0.1919 | 0.197677 | 0.308536 |
| cg03955252 | 3.84E-04 | 3 | rs543093162 | 0.362943 | 0.325295 | 0.326772 | 0.470438 |
| cg01956297 | 3.82E-04 | 18 | rs372435454 | 0.293905 | 0.26727 | 0.252237 | 0.375979 |
| cg05380759 | 3.82E-04 | 3 | rs186792502;rs190010972; rs6771173;rs59887906 | 0.293511 | 0.266458 | 0.263914 | 0.356155 |
| cg21994966 | 3.81E-04 | 15 |  | 0.29585 | 0.27125 | 0.263693 | 0.375276 |
| cg04936213 | 3.81E-04 | 9 | rs377190412;rs574461616; rs183891193;rs556925168 | 0.295185 | 0.270784 | 0.263491 | 0.378259 |
| cg11919271 | 3.81E-04 | 3 | rs189535205;rs61740424; rs558398849 | 0.37624 | 0.352379 | 0.357278 | 0.473362 |
| cg19912160 | 3.81E-04 | 21 | rs573566736;rs181203161; rs559171234;rs575947067 | 0.373973 | 0.349826 | 0.352755 | 0.44475 |
| cg24303944 | 3.81E-04 | 3 | rs565713565;rs191735102; rs183571060 | 0.199215 | 0.174912 | 0.185807 | 0.245277 |
| cg18276112 | 3.81E-04 | 7 | rs148643826 | 0.251892 | 0.219982 | 0.213313 | 0.311316 |
| cg08417022 | 3.80E-04 | 21 |  | 0.291398 | 0.251384 | 0.254162 | 0.362988 |
| cg11098259 | 3.79E-04 | 15 | rs146505777;rs547176007 | 0.260115 | 0.2301 | 0.21969 | 0.331466 |
| cg22820233 | 3.79E-04 | 19 | rs3816051;rs559946949; rs143210491 | 0.28609 | 0.235125 | 0.226659 | 0.348713 |
| cg22639364 | 3.79E-04 | 16 | rs149412731;rs200129673 | 0.276394 | 0.24575 | 0.237709 | 0.334643 |
| cg14999035 | 3.79E-04 | 3 | rs531501061;rs548499182; rs567300122 | 0.284263 | 0.256588 | 0.255633 | 0.383334 |
| cg16689243 | 3.78E-04 | 6 | rs74968708;rs576503207; rs2745346;rs555946300 | 0.214394 | 0.186849 | 0.174088 | 0.268105 |
| cg01752051 | 3.78E-04 | 3 | rs559413426;rs529720730; rs67129644;rs547958734 | 0.240433 | 0.218487 | 0.220545 | 0.318517 |
| cg12461560 | 3.78E-04 | 20 | rs376957755;rs17093805; rs548767298;rs144348771 | 0.366165 | 0.33127 | 0.345188 | 0.439774 |
| cg16413076 | 3.77E-04 | 1 | rs554872724 | 0.213018 | 0.185084 | 0.180779 | 0.284619 |
| cg16172955 | 3.76E-04 | 17 | rs566521048 | 0.318433 | 0.285772 | 0.281582 | 0.392354 |
| cg13596487 | 3.75E-04 | 4 | rs150433967;rs145279419 | 0.312053 | 0.28812 | 0.281787 | 0.393958 |
| cg11603796 | 3.75E-04 | 12 | rs549694300;rs193226064 | 0.316085 | 0.291375 | 0.279433 | 0.377499 |
| cg19989072 | 3.75E-04 | 1 | rs141658593;rs147073466; rs58175882;rs186375645; rs2205604 | 0.342771 | 0.311138 | 0.313883 | 0.439825 |
| cg01727180 | 3.73E-04 | 4 | rs111990618;rs145041843; rs78346024 | 0.276116 | 0.247996 | 0.245944 | 0.351299 |
| cg04131381 | 3.72E-04 | 12 |  | 0.273952 | 0.251142 | 0.249361 | 0.34773 |
| cg13818049 | 3.72E-04 | 19 | rs567197425;rs137885616; rs143456680;rs566284312 | 0.458998 | 0.435851 | 0.436065 | 0.552499 |
| cg18337432 | 3.72E-04 | 6 | rs551523016 | 0.309764 | 0.280634 | 0.276689 | 0.386826 |
| cg18958185 | 3.72E-04 | 4 | rs566333228 | 0.277512 | 0.249956 | 0.237837 | 0.375648 |
| cg08351617 | 3.71E-04 | X |  | 0.488207 | 0.46583 | 0.480792 | 0.540058 |
| cg06143901 | 3.71E-04 | 20 | rs529714922;rs41379048 | 0.648362 | 0.627681 | 0.646682 | 0.696806 |
| cg26620147 | 3.71E-04 | 12 | rs146850643;rs530131819; rs187437987 | 0.229844 | 0.20187 | 0.199246 | 0.292678 |
| cg06172871 | 3.71E-04 | 16 | rs5467 | 0.269166 | 0.245621 | 0.238919 | 0.337087 |
| cg10951959 | 3.71E-04 | 6 | rs555719429 | 0.352399 | 0.327466 | 0.315861 | 0.42002 |
| cg06005163 | 3.70E-04 | 17 |  | 0.337358 | 0.312091 | 0.323543 | 0.414078 |
| cg08370835 | 3.70E-04 | 12 | rs10846761;rs186208098 | 0.157412 | 0.134895 | 0.143358 | 0.196184 |
| cg10969568 | 3.70E-04 | 14 |  | 0.251512 | 0.215067 | 0.208364 | 0.309829 |
| cg10740324 | 3.70E-04 | 5 | rs529121138 | 0.318866 | 0.28584 | 0.280074 | 0.384252 |
| cg03819732 | 3.69E-04 | 5 | rs187580140;rs540214745 | 0.282346 | 0.249818 | 0.237514 | 0.381242 |
| cg12509499 | 3.69E-04 | 17 | rs559119237;rs572729769 | 0.352892 | 0.320425 | 0.322481 | 0.434855 |
| cg18026876 | 3.69E-04 | 13 |  | 0.307338 | 0.28387 | 0.284893 | 0.376048 |
| cg08060645 | 3.69E-04 | 6 | rs140026207;rs112593149 | 0.245238 | 0.222616 | 0.22394 | 0.299435 |
| cg13564880 | 3.68E-04 | 3 | rs570713867;rs77343814; rs10511018 | 0.400291 | 0.379439 | 0.38483 | 0.471165 |
| cg02456934 | 3.68E-04 | 16 | rs566725430;rs143739879; rs78025782 | 0.520172 | 0.488847 | 0.498141 | 0.574881 |
| cg19966540 | 3.68E-04 | 21 | rs183232338;rs41312975 | 0.301975 | 0.275925 | 0.273065 | 0.395149 |
| cg04926108 | 3.68E-04 | 1 | rs543735020 | 0.298544 | 0.266487 | 0.251965 | 0.374749 |
| cg05456810 | 3.67E-04 | 9 |  | 0.256356 | 0.22617 | 0.22237 | 0.332357 |
| cg06159877 | 3.66E-04 | 10 | rs115070618 | 0.306463 | 0.274118 | 0.267838 | 0.365324 |
| cg10689784 | 3.66E-04 | 9 |  | 0.490813 | 0.454176 | 0.47212 | 0.556411 |
| cg02995065 | 3.66E-04 | 17 |  | 0.1979 | 0.165102 | 0.157357 | 0.243281 |
| cg08014027 | 3.65E-04 | 16 | rs115407258;rs556707019 | 0.217194 | 0.194706 | 0.19734 | 0.262058 |
| cg18945137 | 3.65E-04 | 20 | rs572728645;rs539984374; rs142921534;rs528915740 | 0.254095 | 0.213473 | 0.20946 | 0.331364 |
| cg12412309 | 3.64E-04 | 19 | rs183415283 | 0.294129 | 0.271564 | 0.279752 | 0.355909 |
| cg09470277 | 3.63E-04 | 12 | rs532763723 | 0.311275 | 0.273449 | 0.264783 | 0.377303 |
| cg08425576 | 3.63E-04 | 13 | rs556785173;rs576675743 | 0.254642 | 0.232429 | 0.224337 | 0.300816 |
| cg06615010 | 3.63E-04 | 11 | rs539159418;rs57901637 | 0.251647 | 0.231312 | 0.224731 | 0.318541 |
| cg01167335 | 3.62E-04 | 16 |  | 0.284489 | 0.245089 | 0.24529 | 0.394063 |
| cg01925950 | 3.62E-04 | 13 | rs555417567;rs573840933; rs116766837 | 0.271318 | 0.242014 | 0.245132 | 0.327402 |
| cg06841902 | 3.61E-04 | 17 | rs185227595;rs74844701; rs112495541 | 0.465019 | 0.441911 | 0.438037 | 0.529138 |
| cg04342877 | 3.60E-04 | 10 | rs76009228 | 0.26617 | 0.236526 | 0.239949 | 0.338658 |
| cg05661278 | 3.60E-04 | 13 | rs112961928 | 0.300349 | 0.272354 | 0.264953 | 0.381904 |
| cg04869977 | 3.60E-04 | 6 |  | 0.299522 | 0.265244 | 0.254111 | 0.377722 |
| cg04628648 | 3.60E-04 | 12 |  | 0.267515 | 0.241278 | 0.231202 | 0.341815 |
| cg19233981 | 3.59E-04 | 8 |  | 0.320206 | 0.296697 | 0.296175 | 0.404012 |
| cg22541962 | 3.59E-04 | 10 | rs149910901 | 0.287869 | 0.259413 | 0.250598 | 0.365281 |
| cg00182040 | 3.59E-04 | 6 | rs149976653;rs145203784 | 0.231141 | 0.195314 | 0.195848 | 0.293563 |
| cg14382954 | 3.59E-04 | 8 | rs535458002;rs556737044; rs575421204;rs545960756 | 0.346367 | 0.319908 | 0.309461 | 0.440009 |
| cg07052737 | 3.58E-04 | 2 | rs182622461 | 0.310852 | 0.283071 | 0.278802 | 0.394186 |
| cg12175038 | 3.58E-04 | 3 | rs150963389;rs140759325 | 0.325219 | 0.296721 | 0.291848 | 0.3973 |
| cg18963652 | 3.58E-04 | 6 | rs368774252;rs143414155; rs151325067;rs564641199; rs530518749;rs550002671 | 0.290613 | 0.254916 | 0.253073 | 0.366684 |
| cg16664352 | 3.58E-04 | 20 | rs7269117;rs550382662 | 0.365234 | 0.343703 | 0.346239 | 0.437977 |
| cg13184651 | 3.58E-04 | 20 |  | 0.315952 | 0.290815 | 0.285699 | 0.39086 |
| cg09187338 | 3.58E-04 | 7 | rs149182443 | 0.477316 | 0.455633 | 0.458892 | 0.514485 |
| cg07896832 | 3.58E-04 | 8 |  | 0.280681 | 0.256616 | 0.244726 | 0.362355 |
| cg01242422 | 3.57E-04 | 1 |  | 0.44941 | 0.424789 | 0.4358 | 0.49486 |
| cg11948905 | 3.57E-04 | 5 | rs114709172;rs193055309 | 0.227033 | 0.198844 | 0.204007 | 0.284363 |
| cg24055846 | 3.57E-04 | 3 | rs551391850 | 0.38553 | 0.364645 | 0.374558 | 0.457113 |
| cg00528014 | 3.56E-04 | 17 | rs190183259 | 0.567353 | 0.54229 | 0.559365 | 0.621229 |
| cg02584257 | 3.56E-04 | 2 | rs527379408;rs548624577 | 0.31258 | 0.290098 | 0.277088 | 0.376732 |
| cg12455066 | 3.56E-04 | 3 | rs149904563 | 0.241372 | 0.218813 | 0.222038 | 0.310794 |
| cg10346784 | 3.55E-04 | 7 | rs572363972 | 0.258928 | 0.237953 | 0.233271 | 0.338161 |
| cg26306888 | 3.55E-04 | 7 | rs150806182;rs118035358; rs552516383 | 0.288635 | 0.266585 | 0.265713 | 0.349086 |
| cg08748811 | 3.55E-04 | 4 | rs556943468;rs75763792 | 0.264014 | 0.230008 | 0.223581 | 0.351972 |
| cg14690837 | 3.54E-04 | 12 | rs577128781;rs540146646 | 0.322935 | 0.283598 | 0.279474 | 0.40235 |
| cg11042653 | 3.54E-04 | 1 | rs188735460;rs76221373; rs114155106 | 0.483963 | 0.457397 | 0.461825 | 0.54956 |
| cg26390455 | 3.54E-04 | 2 | rs544893272 | 0.302073 | 0.281068 | 0.293087 | 0.393338 |
| cg18921653 | 3.53E-04 | 11 | rs570683427 | 0.271773 | 0.251693 | 0.256301 | 0.313819 |
| cg15000124 | 3.53E-04 | 7 | rs111693051 | 0.221495 | 0.192397 | 0.186755 | 0.264942 |
| cg17067696 | 3.53E-04 | 6 | rs569880364;rs537437074 | 0.297284 | 0.264014 | 0.253185 | 0.379934 |
| cg10933959 | 3.52E-04 | 15 |  | 0.358047 | 0.329199 | 0.326525 | 0.437945 |
| cg22479077 | 3.52E-04 | 3 | rs567556716;rs536129079 | 0.349571 | 0.318929 | 0.311777 | 0.443988 |
| cg24806778 | 3.52E-04 | 6 | rs184188057;rs573194688 | 0.227389 | 0.204105 | 0.198236 | 0.277459 |
| cg13609544 | 3.52E-04 | 17 | rs572191849;rs540081568; rs560316700 | 0.393138 | 0.370263 | 0.374161 | 0.45957 |
| cg14919455 | 3.51E-04 | 6 | rs552899455;rs570893955 | 0.367804 | 0.339961 | 0.334328 | 0.444001 |
| cg25305879 | 3.51E-04 | 2 | rs563498788;rs187071305; rs546102143 | 0.316841 | 0.287291 | 0.279908 | 0.398361 |
| cg03637988 | 3.51E-04 | 11 | rs543094678;rs193187949; rs531890295;rs544857259 | 0.24942 | 0.226681 | 0.226224 | 0.3086 |
| cg00849335 | 3.51E-04 | 1 | rs562659832;rs533451657 | 0.289706 | 0.250154 | 0.249716 | 0.391303 |
| cg03211843 | 3.51E-04 | 4 | rs112617406;rs149161484; rs117999581 | 0.514325 | 0.490392 | 0.492237 | 0.564966 |
| cg26438294 | 3.50E-04 | 16 | rs116478524;rs17207792 | 0.292726 | 0.261165 | 0.259255 | 0.347531 |
| cg09008259 | 3.50E-04 | 7 | rs78470022;rs529597440 | 0.30671 | 0.278086 | 0.271422 | 0.386986 |
| cg19733025 | 3.49E-04 | 17 | rs531808124 | 0.270019 | 0.234163 | 0.231301 | 0.336754 |
| cg20397849 | 3.49E-04 | 11 |  | 0.349457 | 0.31537 | 0.322652 | 0.424983 |
| cg18235479 | 3.49E-04 | 8 | rs186706889;rs552350296 | 0.238453 | 0.208985 | 0.20687 | 0.314033 |
| cg05641051 | 3.49E-04 | 2 |  | 0.264463 | 0.239693 | 0.224204 | 0.337791 |
| cg18038894 | 3.48E-04 | 17 | rs563353035;rs77838835; rs186013502 | 0.347667 | 0.323307 | 0.324406 | 0.41638 |
| cg18084554 | 3.48E-04 | 19 | rs577103752;rs142620877 | 0.292623 | 0.266465 | 0.261638 | 0.345344 |
| cg08622198 | 3.48E-04 | 1 | rs573634157;rs540935740; rs74149217;rs560553741 | 0.357216 | 0.327285 | 0.314091 | 0.425266 |
| cg23501193 | 3.48E-04 | 12 | rs191954181 | 0.177819 | 0.157105 | 0.156683 | 0.221276 |
| cg07687273 | 3.47E-04 | 2 |  | 0.287003 | 0.248827 | 0.25866 | 0.368425 |
| cg00405044 | 3.47E-04 | 2 | rs192931215;rs530891692; rs542693677 | 0.264459 | 0.239538 | 0.244299 | 0.337238 |
| cg12610557 | 3.47E-04 | 15 |  | 0.254744 | 0.228275 | 0.212516 | 0.313509 |
| cg15195816 | 3.47E-04 | 1 | rs76380398;rs566870087; rs180980661 | 0.267684 | 0.234821 | 0.238707 | 0.334506 |
| cg17000067 | 3.46E-04 | 1 | rs61778409;rs574859747 | 0.273929 | 0.248178 | 0.24264 | 0.334565 |
| cg06040856 | 3.46E-04 | 2 | rs564651421;rs533209485 | 0.247473 | 0.214237 | 0.196962 | 0.298731 |
| cg22535089 | 3.46E-04 | 13 | rs189937129;rs181095745 ;rs73575607 | 0.329886 | 0.285052 | 0.277449 | 0.405377 |
| cg18548520 | 3.45E-04 | 8 | rs190757329;rs150224683; rs564265152 | 0.227033 | 0.196801 | 0.197396 | 0.289475 |
| cg22481130 | 3.45E-04 | 6 | rs374946597 | 0.272619 | 0.247051 | 0.24888 | 0.341107 |
| cg04482356 | 3.45E-04 | 4 | rs186603625;rs572655059 | 0.601546 | 0.579183 | 0.578606 | 0.656454 |
| cg10347224 | 3.44E-04 | 1 | rs192287390 | 0.566172 | 0.54447 | 0.54843 | 0.619392 |
| cg09948350 | 3.44E-04 | 2 | rs529195418;rs77274135 | 0.356253 | 0.329713 | 0.33157 | 0.45882 |
| cg25174412 | 3.43E-04 | 12 | rs146713345 | 0.175264 | 0.153019 | 0.161277 | 0.215902 |
| cg02585938 | 3.43E-04 | 14 | rs151123899;rs541932758 | 0.297384 | 0.27028 | 0.265324 | 0.372108 |
| cg05211664 | 3.43E-04 | 3 | rs201576814;rs145303759 | 0.255847 | 0.225396 | 0.218314 | 0.315744 |
| cg00482119 | 3.43E-04 | 15 |  | 0.268061 | 0.24431 | 0.238807 | 0.329269 |
| cg01502428 | 3.43E-04 | 17 | rs145763670;rs559501561 | 0.342203 | 0.313385 | 0.314595 | 0.441295 |
| cg25010677 | 3.42E-04 | 17 | rs368126879;rs118026114 | 0.405226 | 0.36791 | 0.368239 | 0.508585 |
| cg19143716 | 3.42E-04 | 22 |  | 0.22721 | 0.185847 | 0.187504 | 0.284504 |
| cg10080942 | 3.41E-04 | 1 | rs547010972;rs570079776 | 0.259866 | 0.236123 | 0.224867 | 0.329895 |
| cg02432502 | 3.41E-04 | 1 |  | 0.26197 | 0.239499 | 0.244965 | 0.337521 |
| cg05493358 | 3.41E-04 | 11 | rs547456471;rs567435386; rs147690116 | 0.290233 | 0.260481 | 0.258415 | 0.352036 |
| cg15243027 | 3.41E-04 | 2 | rs186965572;rs577748092 | 0.30921 | 0.282017 | 0.265652 | 0.397475 |
| cg24339704 | 3.41E-04 | 19 | rs563395381;rs740054 | 0.377113 | 0.354514 | 0.339401 | 0.449387 |
| cg21074468 | 3.40E-04 | 6 | rs60494656;rs542439128; rs562128363;rs141176557 | 0.278921 | 0.248868 | 0.241614 | 0.350214 |
| cg05611939 | 3.40E-04 | 17 | rs372543612 | 0.275944 | 0.245454 | 0.246791 | 0.3748 |
| cg01157084 | 3.40E-04 | X |  | 0.252846 | 0.22261 | 0.238795 | 0.341009 |
| cg10429934 | 3.39E-04 | 7 | rs150823576;rs572795769; rs533763725;rs77395958 | 0.304207 | 0.268613 | 0.261986 | 0.374641 |
| cg16534503 | 3.39E-04 | 3 | rs574280314;rs541028102 | 0.26982 | 0.239874 | 0.233439 | 0.343915 |
| cg05759347 | 3.39E-04 | 1 |  | 0.149201 | 0.126659 | 0.126003 | 0.190898 |
| cg14584051 | 3.39E-04 | 6 | rs562222372;rs9476678; rs543825730 | 0.307845 | 0.284315 | 0.272645 | 0.368858 |
| cg07099969 | 3.39E-04 | 9 | rs570146489 | 0.306417 | 0.275751 | 0.277705 | 0.39467 |
| cg20099625 | 3.39E-04 | 3 | rs76328061 | 0.250938 | 0.218807 | 0.21138 | 0.313591 |
| cg00382999 | 3.38E-04 | 3 | rs536595577 | 0.283329 | 0.25057 | 0.246089 | 0.361633 |
| cg11874719 | 3.38E-04 | 16 | rs564978587;rs150511496 | 0.399346 | 0.370382 | 0.372132 | 0.462842 |
| cg04868129 | 3.36E-04 | 5 | rs139230083;rs143244977 | 0.24786 | 0.225974 | 0.215831 | 0.324099 |
| cg13692437 | 3.35E-04 | 4 | rs114724760 | 0.2374 | 0.211521 | 0.21535 | 0.284287 |
| cg23397030 | 3.35E-04 | 14 | rs575957190;rs544812008; rs558526022 | 0.236688 | 0.207486 | 0.194625 | 0.310645 |
| cg09144083 | 3.35E-04 | 4 | rs181081264;rs73014111 | 0.274022 | 0.245519 | 0.231745 | 0.339165 |
| cg06494464 | 3.34E-04 | 22 | rs560131107 | 0.471036 | 0.444511 | 0.447073 | 0.53157 |
| cg23364294 | 3.34E-04 | 2 | rs193248132;rs16823661 | 0.205578 | 0.177981 | 0.180237 | 0.25583 |
| cg09875747 | 3.34E-04 | 7 | rs137947484 | 0.325682 | 0.298577 | 0.292989 | 0.41031 |
| cg18493214 | 3.33E-04 | 6 | rs75469261;rs183885434 | 0.245415 | 0.224245 | 0.217984 | 0.310155 |
| cg00402156 | 3.33E-04 | 16 | rs190133718;rs539604547 | 0.35366 | 0.327211 | 0.332733 | 0.413161 |
| cg06489037 | 3.33E-04 | 3 | rs111356263;rs560618377; rs529409829 | 0.387411 | 0.366576 | 0.364659 | 0.469529 |
| cg11474748 | 3.32E-04 | 13 | rs143465124;rs546708202 | 0.447635 | 0.422576 | 0.409612 | 0.517368 |
| cg11415350 | 3.32E-04 | 22 | rs553022780 | 0.207537 | 0.184945 | 0.198281 | 0.268327 |
| cg14064277 | 3.32E-04 | 19 | rs550384575;rs553456624 | 0.232208 | 0.207566 | 0.210227 | 0.312259 |
| cg11003806 | 3.32E-04 | 15 | rs530521240;rs550345007; rs144970164 | 0.325697 | 0.298719 | 0.296431 | 0.415634 |
| cg25841625 | 3.31E-04 | 4 | rs149969547 | 0.537395 | 0.515836 | 0.501413 | 0.588287 |
| cg26950588 | 3.31E-04 | 9 | rs554883466;rs576248562 | 0.281814 | 0.252576 | 0.239021 | 0.357786 |
| cg03689552 | 3.31E-04 | 16 | rs549937754;rs114930858; rs538739787;rs558576646 | 0.310032 | 0.282938 | 0.265589 | 0.378831 |
| cg16834198 | 3.30E-04 | 9 | rs547887988;rs7024472 | 0.190631 | 0.169569 | 0.168707 | 0.231049 |
| cg01406317 | 3.30E-04 | 16 | rs370668160;rs538050615; rs181919098 | 0.273919 | 0.238585 | 0.229797 | 0.344705 |
| cg21290787 | 3.30E-04 | 6 | rs185312960;rs571189102 | 0.334155 | 0.31262 | 0.303784 | 0.390156 |
| cg16958707 | 3.30E-04 | 3 | rs556783425;rs574520348 | 0.382935 | 0.362754 | 0.358716 | 0.489187 |
| cg01399658 | 3.29E-04 | 16 |  | 0.251956 | 0.214487 | 0.211591 | 0.303354 |
| cg21161403 | 3.29E-04 | 8 | rs536865797;rs74486821; rs575780703;rs75470489; rs147984839;rs572023481 | 0.566322 | 0.543897 | 0.531721 | 0.634217 |
| cg18499684 | 3.29E-04 | 5 |  | 0.289467 | 0.257605 | 0.248473 | 0.354164 |
| cg03313180 | 3.29E-04 | 8 | rs13273645 | 0.381146 | 0.354467 | 0.352978 | 0.443629 |
| cg15365305 | 3.29E-04 | 9 |  | 0.342868 | 0.320336 | 0.304252 | 0.417332 |
| cg12919527 | 3.29E-04 | 1 | rs568767299;rs535883057 | 0.205001 | 0.178201 | 0.17526 | 0.245778 |
| cg02770745 | 3.28E-04 | 11 | rs142228593;rs531782725 | 0.296289 | 0.244689 | 0.247153 | 0.376142 |
| cg14480116 | 3.28E-04 | 2 | rs565363919;rs533027300; rs543190589 | 0.291262 | 0.254235 | 0.243433 | 0.372562 |
| cg12663684 | 3.28E-04 | 10 | rs564930624;rs532538095 | 0.222065 | 0.197033 | 0.188891 | 0.265317 |
| cg23588307 | 3.28E-04 | 17 | rs1849738;rs114125300; rs537618330;rs557390789; rs138754984;rs542224080 | 0.27373 | 0.246244 | 0.248154 | 0.343618 |
| cg08356841 | 3.28E-04 | 1 | rs190904683;rs140287362; rs587660138 | 0.308236 | 0.278409 | 0.279017 | 0.385599 |
| cg08288126 | 3.28E-04 | 3 | rs4135245;rs145806574 | 0.318846 | 0.283784 | 0.276807 | 0.405841 |
| cg14153055 | 3.27E-04 | 10 |  | 0.232131 | 0.205708 | 0.198454 | 0.284309 |
| cg01272182 | 3.27E-04 | 3 | rs546403940 | 0.286353 | 0.233175 | 0.246523 | 0.381906 |
| cg20449670 | 3.27E-04 | 1 | rs574296987;rs28780420; rs553414427;rs571974358; rs545467883 | 0.301434 | 0.277568 | 0.262818 | 0.374981 |
| cg16481332 | 3.27E-04 | 7 |  | 0.288103 | 0.256446 | 0.250152 | 0.361452 |
| cg12195054 | 3.27E-04 | 13 | rs559203692;rs573079821 | 0.223058 | 0.17943 | 0.198839 | 0.277365 |
| cg25828963 | 3.26E-04 | 13 | rs9568150;rs117034759; rs538798446 | 0.521454 | 0.498972 | 0.497814 | 0.561955 |
| cg14028598 | 3.26E-04 | 1 | rs144639987;rs12045870; rs541819855;rs141125653 | 0.383677 | 0.353264 | 0.35278 | 0.486785 |
| cg00759609 | 3.26E-04 | 3 | rs555304831 | 0.524088 | 0.488103 | 0.494069 | 0.560997 |
| cg16286874 | 3.26E-04 | 4 |  | 0.274952 | 0.251189 | 0.236733 | 0.350093 |
| cg08870098 | 3.25E-04 | 3 | rs118083550;rs192140251; rs188304519 | 0.429293 | 0.400802 | 0.405272 | 0.510393 |
| cg07195748 | 3.24E-04 | 8 |  | 0.364133 | 0.328187 | 0.345285 | 0.446504 |
| cg07285167 | 3.24E-04 | 1 | rs144956945;rs76171015 | 0.24764 | 0.213322 | 0.20574 | 0.317173 |
| cg05249836 | 3.24E-04 | 22 | rs577611837;rs75464288; rs78808751;rs184770359 | 0.324851 | 0.292849 | 0.28529 | 0.403216 |
| cg18245735 | 3.24E-04 | 2 | rs570691484;rs540163375; rs553359259 | 0.30026 | 0.266181 | 0.263548 | 0.379503 |
| cg15473346 | 3.23E-04 | 1 | rs151051919;rs16851240; rs551743009 | 0.097194 | 0.073006 | 0.080295 | 0.117295 |
| cg08132858 | 3.23E-04 | 7 | rs371512486;rs542475256; rs563708678;rs575938437 | 0.304017 | 0.272946 | 0.274152 | 0.36437 |
| cg06308633 | 3.23E-04 | 15 |  | 0.182556 | 0.160433 | 0.166635 | 0.230219 |
| cg08277638 | 3.22E-04 | 1 |  | 0.236359 | 0.206622 | 0.20377 | 0.288303 |
| cg26116166 | 3.22E-04 | 4 | rs192416033;rs148427906; rs55809695;rs142537537; rs76622042 | 0.368572 | 0.344231 | 0.34285 | 0.431362 |
| cg06480205 | 3.22E-04 | 11 |  | 0.274176 | 0.244573 | 0.243494 | 0.352484 |
| cg23863319 | 3.21E-04 | 7 | rs532388919;rs551236285; rs571479038;rs537235375 | 0.266231 | 0.243743 | 0.232421 | 0.366361 |
| cg04703221 | 3.21E-04 | 16 | rs111366342 | 0.267852 | 0.238174 | 0.237053 | 0.327191 |
| cg17518710 | 3.20E-04 | 11 | rs537193718;rs118050126 | 0.14534 | 0.122349 | 0.124038 | 0.173052 |
| cg02762945 | 3.20E-04 | 10 | rs553730648;rs565711223 | 0.314972 | 0.281097 | 0.280178 | 0.383215 |
| cg01188078 | 3.19E-04 | 19 |  | 0.330389 | 0.298697 | 0.29262 | 0.436436 |
| cg17109053 | 3.19E-04 | 2 | rs536377433;rs116035426 | 0.252277 | 0.226353 | 0.22018 | 0.322438 |
| cg20112193 | 3.19E-04 | 5 | rs113158081;rs184920094; rs75070487;rs59950488 | 0.398773 | 0.37526 | 0.384241 | 0.439838 |
| cg21665024 | 3.19E-04 | 5 | rs573732671 | 0.339091 | 0.312396 | 0.308943 | 0.419509 |
| cg17934470 | 3.19E-04 | 5 | rs192240188 | 0.289637 | 0.257182 | 0.255163 | 0.374772 |
| cg03346852 | 3.18E-04 | 11 | rs61751338;rs558735824; rs577074852;rs535003331; rs138493671 | 0.27185 | 0.244376 | 0.232514 | 0.361167 |
| cg22108563 | 3.18E-04 | 5 | rs185681451;rs540692576 | 0.156819 | 0.133123 | 0.137077 | 0.198461 |
| cg07370361 | 3.18E-04 | 6 | rs111321992 | 0.510269 | 0.483985 | 0.500419 | 0.596892 |
| cg13825379 | 3.18E-04 | 8 |  | 0.244098 | 0.21515 | 0.219949 | 0.319811 |
| cg24343508 | 3.17E-04 | 6 |  | 0.485606 | 0.453139 | 0.452582 | 0.559631 |
| cg15519279 | 3.17E-04 | 7 | rs76855813;rs551639190; rs566511115 | 0.501235 | 0.479843 | 0.48173 | 0.576006 |
| cg11411148 | 3.17E-04 | 16 | rs150727633;rs575121018; rs139073609;rs560310802 | 0.299136 | 0.268553 | 0.268768 | 0.37334 |
| cg26596580 | 3.16E-04 | 8 | rs532982958;rs4355753 | 0.270795 | 0.229388 | 0.24802 | 0.329161 |
| cg16526170 | 3.16E-04 | 4 | rs116051774;rs55787530; rs529588274 | 0.252365 | 0.230267 | 0.22533 | 0.320332 |
| cg01611017 | 3.16E-04 | 14 |  | 0.153428 | 0.131328 | 0.125195 | 0.18295 |
| cg19087116 | 3.16E-04 | 7 | rs193126935;rs115766377; rs531174704 | 0.276909 | 0.247018 | 0.248778 | 0.349318 |
| cg13020347 | 3.16E-04 | 1 | rs563626226;rs149714308; rs543205037 | 0.263832 | 0.232699 | 0.21557 | 0.333756 |
| cg20282967 | 3.16E-04 | 17 | rs114076767 | 0.244654 | 0.220899 | 0.220907 | 0.305192 |
| cg22240755 | 3.16E-04 | 12 | rs570834805 | 0.282513 | 0.253064 | 0.237618 | 0.38113 |
| cg08632810 | 3.14E-04 | 8 | rs540554949;rs114229536; rs573827153 | 0.211612 | 0.177859 | 0.188151 | 0.274076 |
| cg03719830 | 3.14E-04 | 20 | rs75546298 | 0.293238 | 0.269653 | 0.259504 | 0.356754 |
| cg24867425 | 3.14E-04 | 1 | rs186393043;rs12065948; rs539056487 | 0.248099 | 0.223038 | 0.240864 | 0.285183 |
| cg00473633 | 3.14E-04 | 17 | rs191489850;rs534112229; rs592461;rs1702940; rs1715485;rs8081559 | 0.367777 | 0.347522 | 0.348062 | 0.426393 |
| cg04728805 | 3.13E-04 | 5 |  | 0.170606 | 0.149146 | 0.15327 | 0.228401 |
| cg22345419 | 3.13E-04 | 10 | rs7910643 | 0.334613 | 0.296162 | 0.292581 | 0.422235 |
| cg25859553 | 3.13E-04 | 1 | rs541804983;rs563274991 | 0.316649 | 0.282413 | 0.289298 | 0.403537 |
| cg00353415 | 3.13E-04 | 7 | rs573741891 | 0.272522 | 0.242616 | 0.235755 | 0.341701 |
| cg04349497 | 3.13E-04 | 5 | rs72767317;rs562207491 | 0.288392 | 0.261895 | 0.250733 | 0.373593 |
| cg06084610 | 3.13E-04 | 3 | rs559455111;rs532979240 | 0.373922 | 0.349847 | 0.331119 | 0.45783 |
| cg20063095 | 3.12E-04 | 2 | rs139880606 | 0.323502 | 0.30037 | 0.300916 | 0.402105 |
| cg15134583 | 3.12E-04 | 1 | rs184656588;rs545583260; rs560564917 | 0.300381 | 0.274193 | 0.264376 | 0.351937 |
| cg17023096 | 3.12E-04 | 10 | rs140689466;rs145338150; rs539175542;rs137957753 | 0.176151 | 0.154888 | 0.161419 | 0.2404 |
| cg01910182 | 3.12E-04 | 7 | rs546217774;rs558327014 | 0.148877 | 0.128855 | 0.136091 | 0.200002 |
| cg25444339 | 3.12E-04 | 7 | rs112042631;rs138371020 | 0.273792 | 0.248168 | 0.238122 | 0.331906 |
| cg25517633 | 3.11E-04 | 17 | rs544942986 | 0.387672 | 0.355037 | 0.359726 | 0.469481 |
| cg00690498 | 3.11E-04 | 14 | rs112088328;rs527862728; rs541749819 | 0.294756 | 0.254708 | 0.246889 | 0.370311 |
| cg06052053 | 3.10E-04 | 6 |  | 0.270638 | 0.246285 | 0.243613 | 0.327878 |
| cg26291072 | 3.10E-04 | 17 | rs150195297;rs556231144; rs569614671 | 0.315203 | 0.285009 | 0.279576 | 0.400958 |
| cg04790775 | 3.09E-04 | 2 | rs192159269;rs575588922; rs147098308;rs536660797; rs555139604 | 0.341491 | 0.303498 | 0.295548 | 0.439069 |
| cg14192764 | 3.09E-04 | 19 | rs115505864;rs8113274 | 0.311027 | 0.276048 | 0.273705 | 0.389693 |
| cg01827633 | 3.09E-04 | 2 | rs575431922 | 0.266159 | 0.239572 | 0.230225 | 0.336162 |
| cg05651511 | 3.09E-04 | 17 | rs542484497 | 0.229823 | 0.209142 | 0.195985 | 0.303643 |
| cg17493310 | 3.09E-04 | 2 | rs185959273 | 0.28227 | 0.254552 | 0.247529 | 0.33999 |
| cg20034027 | 3.08E-04 | 3 | rs149751856 | 0.430922 | 0.407023 | 0.404542 | 0.506948 |
| cg24383718 | 3.07E-04 | 16 | rs139256667 | 0.541086 | 0.518839 | 0.53207 | 0.573744 |
| cg04468621 | 3.07E-04 | 4 | rs566577150 | 0.188242 | 0.153807 | 0.145772 | 0.239784 |
| cg26537335 | 3.06E-04 | 12 | rs184589481;rs544842300; rs145600388 | 0.578953 | 0.555468 | 0.540938 | 0.608377 |
| cg08322008 | 3.06E-04 | 8 | rs558917221;rs575818817; rs186447457 | 0.38158 | 0.342116 | 0.340832 | 0.432843 |
| cg08961793 | 3.06E-04 | 16 | rs565832370;rs6498090 | 0.241992 | 0.215889 | 0.212229 | 0.300746 |
| cg18109527 | 3.06E-04 | 16 | rs544625106;rs561602776; rs192005560 | 0.314437 | 0.285389 | 0.280443 | 0.357568 |
| cg16715360 | 3.06E-04 | 5 | rs184398517;rs532710316; rs113949493 | 0.236218 | 0.207803 | 0.210845 | 0.295139 |
| cg03011556 | 3.05E-04 | 5 | rs201378122 | 0.297612 | 0.274984 | 0.275011 | 0.386367 |
| cg16344577 | 3.05E-04 | 17 | rs569133591 | 0.273999 | 0.24952 | 0.237203 | 0.3362 |
| cg21165486 | 3.05E-04 | 1 | rs572309416 | 0.26155 | 0.23243 | 0.229942 | 0.333144 |
| cg05919238 | 3.04E-04 | 17 | rs547959861;rs137919500; rs567531997 | 0.429082 | 0.37641 | 0.381255 | 0.502757 |
| cg12886054 | 3.04E-04 | 1 | rs191993603;rs565752059 | 0.29378 | 0.272343 | 0.267802 | 0.343862 |
| cg22257514 | 3.04E-04 | 6 | rs555712606;rs185603259 | 0.305357 | 0.275022 | 0.268379 | 0.377147 |
| cg21108554 | 3.04E-04 | 7 | rs538974309;rs186573480; rs572903770;rs149401461 | 0.200889 | 0.176893 | 0.174175 | 0.249705 |
| cg07671185 | 3.04E-04 | 8 | rs547135001;rs559171917; rs529725588;rs116459888; rs375732516 | 0.362213 | 0.326526 | 0.318469 | 0.442987 |
| cg25437886 | 3.04E-04 | 3 | rs576516175 | 0.285487 | 0.241056 | 0.22782 | 0.377683 |
| cg08437251 | 3.04E-04 | 6 | rs552727257;rs577529222 | 0.191862 | 0.167581 | 0.165563 | 0.245224 |
| cg08860021 | 3.03E-04 | 17 | rs557696108;rs571341490 | 0.305538 | 0.278484 | 0.268548 | 0.37883 |
| cg17772342 | 3.03E-04 | 5 | rs565989498 | 0.310653 | 0.287364 | 0.290172 | 0.244178 |
| cg02411596 | 3.03E-04 | 22 | rs561045037 | 0.235717 | 0.205801 | 0.201554 | 0.283573 |
| cg21279677 | 3.03E-04 | 12 | rs149603155 | 0.291718 | 0.27121 | 0.255709 | 0.347635 |
| cg24823912 | 3.02E-04 | 12 | rs374137756 | 0.333471 | 0.308447 | 0.295398 | 0.429125 |
| cg22694271 | 3.02E-04 | 1 | rs115706488;rs146697802 | 0.292758 | 0.254877 | 0.25296 | 0.36374 |
| cg21052905 | 3.02E-04 | 21 |  | 0.284148 | 0.255378 | 0.25135 | 0.348976 |
| cg03508627 | 3.02E-04 | 17 | rs564581237 | 0.196143 | 0.174722 | 0.168181 | 0.255134 |
| cg15489208 | 2.99E-04 | 20 | rs570047258;rs6116196; rs35873660;rs541831915; rs6084608 | 0.25525 | 0.226754 | 0.225104 | 0.343847 |
| cg22567464 | 2.99E-04 | 2 | rs192311440 | 0.211798 | 0.185527 | 0.195425 | 0.288899 |
| cg23283609 | 2.98E-04 | 17 |  | 0.251612 | 0.230238 | 0.223071 | 0.323612 |
| cg23301353 | 2.98E-04 | 10 | rs569276263 | 0.292354 | 0.271559 | 0.264207 | 0.354786 |
| cg13665420 | 2.97E-04 | 20 | rs35861107;rs572515088; rs533640940;rs555485038 | 0.312312 | 0.28517 | 0.294396 | 0.407066 |
| cg15586392 | 2.97E-04 | 8 | rs193014579;rs534962539 | 0.31846 | 0.285016 | 0.296054 | 0.401635 |
| cg26959689 | 2.97E-04 | 1 | rs117353394;rs537001535 | 0.289568 | 0.263855 | 0.261112 | 0.400253 |
| cg01151886 | 2.96E-04 | 7 | rs556407541;rs544623191; rs76367112;rs530240228 | 0.175464 | 0.155437 | 0.14955 | 0.20688 |
| cg18096633 | 2.96E-04 | 2 | rs35074676;rs151239984 | 0.318238 | 0.291707 | 0.279644 | 0.382304 |
| cg01761758 | 2.96E-04 | 17 | rs145763670;rs559501561 | 0.373279 | 0.335482 | 0.336079 | 0.457708 |
| cg17501210 | 2.96E-04 | 6 | rs569992380 | 0.635681 | 0.597391 | 0.606114 | 0.703276 |
| cg08952488 | 2.96E-04 | 16 | rs144380632;rs115176574; rs12148990 | 0.346129 | 0.319834 | 0.313383 | 0.420215 |
| cg03683213 | 2.95E-04 | 1 | rs575513873;rs74374037 | 0.245221 | 0.209538 | 0.204874 | 0.302958 |
| cg22033758 | 2.95E-04 | 5 | rs7733948;rs1469387 | 0.293256 | 0.259634 | 0.257709 | 0.362041 |
| cg22130509 | 2.95E-04 | 2 | rs568242767;rs182963042 | 0.235508 | 0.204361 | 0.214481 | 0.300581 |
| cg01247459 | 2.94E-04 | 11 |  | 0.265128 | 0.233193 | 0.222726 | 0.341229 |
| cg22517841 | 2.94E-04 | 1 | rs149057742;rs143644339; rs559826953;rs141888329 | 0.283849 | 0.253481 | 0.238195 | 0.354459 |
| cg22455501 | 2.94E-04 | 3 |  | 0.426924 | 0.404248 | 0.390219 | 0.527318 |
| cg11597277 | 2.93E-04 | 20 |  | 0.373464 | 0.332078 | 0.334371 | 0.460104 |
| cg00992745 | 2.93E-04 | 10 |  | 0.333871 | 0.305984 | 0.297576 | 0.417064 |
| cg26478331 | 2.93E-04 | 12 | rs149772010;rs144584255 | 0.252856 | 0.221144 | 0.210288 | 0.342221 |
| cg01479187 | 2.93E-04 | 2 | rs552833229;rs377059085 | 0.268435 | 0.243303 | 0.237467 | 0.34319 |
| cg09740468 | 2.93E-04 | 6 | rs192135369 | 0.344371 | 0.314931 | 0.310183 | 0.407365 |
| cg10588135 | 2.92E-04 | 17 | rs545551051 | 0.22383 | 0.191786 | 0.201733 | 0.272848 |
| cg14510831 | 2.92E-04 | 4 |  | 0.212353 | 0.179561 | 0.183435 | 0.279913 |
| cg10702418 | 2.92E-04 | 7 | rs189265386;rs556713166 | 0.338758 | 0.298868 | 0.295944 | 0.425718 |
| cg11016450 | 2.91E-04 | 19 |  | 0.356209 | 0.333125 | 0.329761 | 0.396594 |
| cg25621692 | 2.91E-04 | 4 | rs554992424;rs190447853; rs80165698 | 0.249685 | 0.210972 | 0.21256 | 0.318775 |
| cg07211972 | 2.91E-04 | 21 | rs111386779 | 0.323516 | 0.285672 | 0.268717 | 0.407617 |
| cg02262553 | 2.91E-04 | 4 | rs72613149 | 0.254287 | 0.225595 | 0.215323 | 0.334412 |
| cg10132464 | 2.91E-04 | 3 | rs116364400;rs545823457 | 0.249006 | 0.209448 | 0.199618 | 0.317354 |
| cg20067200 | 2.91E-04 | 5 | rs116411109 | 0.305799 | 0.278663 | 0.276493 | 0.39632 |
| cg24263958 | 2.90E-04 | 20 | rs554726366;rs214750; rs139360210 | 0.472582 | 0.451851 | 0.468328 | 0.545315 |
| cg09023491 | 2.90E-04 | 3 | rs577546693;rs533799344 | 0.229011 | 0.19647 | 0.203615 | 0.290329 |
| cg20375030 | 2.90E-04 | 6 | rs557997914;rs577793511 | 0.286695 | 0.257472 | 0.249587 | 0.35219 |
| cg21991396 | 2.89E-04 | 1 | rs201896158;rs202204798; rs191304686 | 0.318316 | 0.280907 | 0.279163 | 0.390178 |
| cg26703997 | 2.89E-04 | 14 | rs537528171 | 0.287658 | 0.266159 | 0.253436 | 0.33227 |
| cg13277939 | 2.89E-04 | 14 | rs190212368 | 0.253001 | 0.221563 | 0.22131 | 0.334391 |
| cg00593814 | 2.89E-04 | 16 |  | 0.266615 | 0.236785 | 0.24113 | 0.351734 |
| cg22255432 | 2.88E-04 | 2 | rs549107727 | 0.256855 | 0.228544 | 0.225311 | 0.320573 |
| cg25145679 | 2.88E-04 | 2 | rs12999766;rs528456685 | 0.337975 | 0.313496 | 0.309395 | 0.411867 |
| cg24894783 | 2.87E-04 | 5 | rs187319945 | 0.415415 | 0.376497 | 0.387234 | 0.499973 |
| cg08986203 | 2.87E-04 | 18 | rs183094340;rs186876104; rs34154304 | 0.225845 | 0.198399 | 0.186227 | 0.284322 |
| cg17115737 | 2.87E-04 | 7 | rs558883183;rs192345718 | 0.546808 | 0.523933 | 0.53094 | 0.629901 |
| cg10239857 | 2.86E-04 | 1 | rs151055301;rs186871009 | 0.276966 | 0.254565 | 0.238587 | 0.337995 |
| cg03479114 | 2.86E-04 | 4 | rs561200021;rs112480618 | 0.423874 | 0.401479 | 0.401325 | 0.498186 |
| cg03914662 | 2.86E-04 | 11 |  | 0.293518 | 0.272608 | 0.26341 | 0.348499 |
| cg20427144 | 2.86E-04 | 7 | rs560198678;rs148041964; rs188751195 | 0.315873 | 0.291285 | 0.289029 | 0.38169 |
| cg07413155 | 2.85E-04 | 2 |  | 0.322864 | 0.285479 | 0.266921 | 0.401719 |
| cg11031945 | 2.85E-04 | 12 | rs192932714;rs527767452 | 0.285728 | 0.261201 | 0.256613 | 0.342055 |
| cg09117588 | 2.85E-04 | 8 |  | 0.349826 | 0.327386 | 0.322137 | 0.423214 |
| cg25228921 | 2.84E-04 | 7 | rs571958185 | 0.523528 | 0.501639 | 0.5088 | 0.598866 |
| cg02925118 | 2.84E-04 | 14 | rs560126082 | 0.271515 | 0.249125 | 0.238577 | 0.35255 |
| cg01425249 | 2.84E-04 | 1 | rs531974339;rs147576233 | 0.331585 | 0.300314 | 0.294216 | 0.422542 |
| cg04701118 | 2.84E-04 | 22 | rs141684342;rs147060589; rs147680141;rs149126501 | 0.317923 | 0.283908 | 0.27275 | 0.405816 |
| cg13007187 | 2.83E-04 | 11 | rs560075066;rs527695540 | 0.301796 | 0.279848 | 0.27104 | 0.371092 |
| cg19717347 | 2.82E-04 | 12 | rs555346990;rs143198071 | 0.313587 | 0.288345 | 0.286223 | 0.392267 |
| cg06603775 | 2.82E-04 | 9 | rs373450447;rs149970848 | 0.270833 | 0.24158 | 0.244853 | 0.348747 |
| cg23381867 | 2.81E-04 | 2 | rs181895872;rs538297031 | 0.339713 | 0.299226 | 0.302976 | 0.436825 |
| cg03822565 | 2.81E-04 | 1 | rs560301339 | 0.321927 | 0.280663 | 0.275506 | 0.402516 |
| cg19705285 | 2.81E-04 | 20 | rs576083902 | 0.348505 | 0.317438 | 0.31769 | 0.417125 |
| cg12122241 | 2.80E-04 | 20 | rs146683181 | 0.323672 | 0.297255 | 0.294725 | 0.394541 |
| cg00040825 | 2.80E-04 | 19 | rs564581345;rs571693996; rs374783695 | 0.263315 | 0.23344 | 0.229949 | 0.329554 |
| cg05254327 | 2.80E-04 | 12 | rs565326979;rs532874431 | 0.255019 | 0.224365 | 0.222573 | 0.321536 |
| cg00797554 | 2.80E-04 | 6 | rs543045876;rs7754938 | 0.157647 | 0.131098 | 0.130231 | 0.197282 |
| cg20964377 | 2.80E-04 | 2 | rs537769644 | 0.268365 | 0.247331 | 0.242298 | 0.318336 |
| cg19074903 | 2.80E-04 | 1 | rs557285678;rs191477869 | 0.281779 | 0.247327 | 0.240498 | 0.361208 |
| cg12237948 | 2.80E-04 | 20 | rs190773974;rs6035606 | 0.360563 | 0.336414 | 0.335634 | 0.438713 |
| cg17642700 | 2.79E-04 | 1 | rs5780023 | 0.330042 | 0.302951 | 0.300232 | 0.398774 |
| cg15867796 | 2.79E-04 | 5 | rs531831201 | 0.260961 | 0.231079 | 0.231225 | 0.30767 |
| cg10620195 | 2.79E-04 | 22 | rs575051875 | 0.299864 | 0.272074 | 0.271225 | 0.371092 |
| cg03126694 | 2.79E-04 | 13 | rs374863736;rs538342545 | 0.29755 | 0.271667 | 0.271696 | 0.35683 |
| cg14486847 | 2.79E-04 | 1 | rs149616646 | 0.300313 | 0.268377 | 0.260243 | 0.376616 |
| cg15072057 | 2.78E-04 | 11 | rs555336650;rs181599703; rs111376829 | 0.319846 | 0.293351 | 0.287689 | 0.397227 |
| cg03016083 | 2.78E-04 | 4 | rs577755315;rs373842468; rs73242642 | 0.255405 | 0.225685 | 0.225686 | 0.330103 |
| cg20958815 | 2.77E-04 | 1 | rs187574604 | 0.253162 | 0.23228 | 0.23288 | 0.328102 |
| cg25200484 | 2.77E-04 | X |  | 0.374419 | 0.353322 | 0.352485 | 0.334931 |
| cg14382194 | 2.76E-04 | 4 | rs543587486;rs148370552; rs190735290 | 0.270956 | 0.22304 | 0.223623 | 0.356016 |
| cg14539563 | 2.76E-04 | 12 | rs370678391 | 0.248756 | 0.220903 | 0.210562 | 0.324072 |
| cg01976472 | 2.76E-04 | 7 |  | 0.316915 | 0.289394 | 0.284879 | 0.365828 |
| cg01307861 | 2.76E-04 | 13 | rs144457905;rs74789192; rs566367259;rs147823414; rs111983846;rs574961695 | 0.379036 | 0.35653 | 0.340183 | 0.469832 |
| cg20571119 | 2.76E-04 | 1 | rs534433208;rs113018617; rs577691457 | 0.259617 | 0.232087 | 0.224172 | 0.343653 |
| cg26950866 | 2.75E-04 | 8 | rs528784555;rs186999064 | 0.251228 | 0.22672 | 0.227302 | 0.301782 |
| cg17122582 | 2.74E-04 | 2 | rs67268513;rs146892509 | 0.322144 | 0.282051 | 0.276215 | 0.380975 |
| cg05345476 | 2.74E-04 | 4 |  | 0.66646 | 0.643243 | 0.662064 | 0.711908 |
| cg04886849 | 2.73E-04 | 10 | rs3026050 | 0.318436 | 0.29396 | 0.28907 | 0.383024 |
| cg19742173 | 2.73E-04 | 15 | rs564953166;rs575144471 | 0.234645 | 0.202659 | 0.198537 | 0.301996 |
| cg16283604 | 2.73E-04 | 13 | rs559025962;rs566247796 | 0.251504 | 0.223 | 0.225048 | 0.322886 |
| cg21584353 | 2.73E-04 | 6 | rs112548920 | 0.252657 | 0.230711 | 0.217694 | 0.329301 |
| cg02544380 | 2.73E-04 | 1 | rs145184128;rs530459738 | 0.334472 | 0.310951 | 0.313469 | 0.393106 |
| cg23182756 | 2.71E-04 | 16 | rs557463816;rs577332603 | 0.239352 | 0.215785 | 0.208713 | 0.299712 |
| cg19787543 | 2.71E-04 | 16 | rs564165050;rs78477699; rs138472249;rs141473693; rs188483875 | 0.316964 | 0.274411 | 0.258949 | 0.383374 |
| cg15763020 | 2.71E-04 | 2 | rs183274303;rs368597999 | 0.227293 | 0.203893 | 0.209744 | 0.292554 |
| cg11080651 | 2.70E-04 | 5 |  | 0.181303 | 0.153627 | 0.14691 | 0.196567 |
| cg06517984 | 2.70E-04 | 8 | rs564573057;rs528677043; rs111647548;rs561825493; rs529322072;rs35301504; rs117037204 | 0.769249 | 0.742533 | 0.76227 | 0.832177 |
| cg14996692 | 2.70E-04 | 11 |  | 0.246226 | 0.225803 | 0.220026 | 0.307088 |
| cg13180519 | 2.69E-04 | 19 |  | 0.254207 | 0.226627 | 0.218706 | 0.331266 |
| cg07006935 | 2.69E-04 | 7 | rs530201906;rs186946604 | 0.254332 | 0.22294 | 0.222233 | 0.321804 |
| cg10914422 | 2.69E-04 | 8 | rs554766111;rs139133726; rs143012745;rs72607402 | 0.319823 | 0.285535 | 0.291653 | 0.401712 |
| cg17734624 | 2.69E-04 | 1 | rs73008251;rs1539571 | 0.277981 | 0.251398 | 0.253142 | 0.332095 |
| cg12622973 | 2.69E-04 | 1 | rs186236919;rs536229966; rs556229007;rs569684643 | 0.36286 | 0.33062 | 0.313031 | 0.436296 |
| cg17932662 | 2.68E-04 | 1 | rs545311586 | 0.342992 | 0.31918 | 0.320313 | 0.417405 |
| cg24934803 | 2.68E-04 | 20 | rs574370395 | 0.217414 | 0.193352 | 0.189513 | 0.282161 |
| cg24918667 | 2.68E-04 | 19 |  | 0.310048 | 0.27555 | 0.25596 | 0.392097 |
| cg10161434 | 2.66E-04 | 9 |  | 0.416764 | 0.39617 | 0.397994 | 0.508471 |
| cg13390284 | 2.66E-04 | 1 |  | 0.356541 | 0.336221 | 0.333847 | 0.4116 |
| cg12457834 | 2.66E-04 | 2 |  | 0.269544 | 0.240573 | 0.233828 | 0.338241 |
| cg07765820 | 2.66E-04 | 6 | rs551611245 | 0.471587 | 0.444456 | 0.442191 | 0.542673 |
| cg19526041 | 2.66E-04 | 15 | rs186845495;rs191324558; rs369652234;rs553525096; rs147567675 | 0.330761 | 0.301963 | 0.303488 | 0.395348 |
| cg19024476 | 2.66E-04 | 5 | rs186482331 | 0.251601 | 0.218768 | 0.22398 | 0.322042 |
| cg21085915 | 2.65E-04 | 6 | rs558781766;rs577010283 | 0.256019 | 0.222735 | 0.21818 | 0.31968 |
| cg20299925 | 2.65E-04 | 3 | rs140370500;rs543429728; rs184435500 | 0.266891 | 0.241031 | 0.240895 | 0.327641 |
| cg22854463 | 2.65E-04 | 1 | rs547320474 | 0.276389 | 0.250072 | 0.24163 | 0.354493 |
| cg26006714 | 2.65E-04 | 10 |  | 0.298834 | 0.271084 | 0.263364 | 0.393089 |
| cg08721324 | 2.65E-04 | 16 | rs190539509;rs118042009 | 0.385591 | 0.355536 | 0.373188 | 0.477839 |
| cg11675009 | 2.64E-04 | 3 | rs34601454;rs541579769; rs559589503;rs200023218; rs545061605 | 0.281981 | 0.253948 | 0.243937 | 0.356493 |
| cg06818377 | 2.64E-04 | 8 | rs6474026 | 0.157901 | 0.127941 | 0.135469 | 0.191703 |
| cg06075261 | 2.64E-04 | 9 | rs542235980;rs562023210 | 0.262694 | 0.238389 | 0.228288 | 0.346002 |
| cg25753291 | 2.64E-04 | 5 | rs148513653;rs558763963; rs185264940;rs146278774 | 0.509808 | 0.485268 | 0.502486 | 0.569572 |
| cg15441686 | 2.64E-04 | 9 | rs552159874;rs182001281; rs528211295 | 0.235277 | 0.213113 | 0.212436 | 0.306682 |
| cg01323964 | 2.64E-04 | 7 | rs568898547;rs376560258 | 0.303978 | 0.260604 | 0.26346 | 0.381731 |
| cg01215778 | 2.63E-04 | 8 | rs72681535;rs545981766; rs564473930;rs78498935 | 0.237672 | 0.213797 | 0.205766 | 0.286576 |
| cg26633899 | 2.63E-04 | 4 | rs561630287 | 0.657654 | 0.631921 | 0.649042 | 0.704425 |
| cg14782541 | 2.63E-04 | 7 | rs537479467;rs191000074 | 0.285195 | 0.250514 | 0.258345 | 0.390514 |
| cg23365971 | 2.63E-04 | 20 |  | 0.288987 | 0.259806 | 0.260647 | 0.372412 |
| cg19997577 | 2.63E-04 | 5 |  | 0.345092 | 0.308958 | 0.312688 | 0.413229 |
| cg04093633 | 2.63E-04 | 15 |  | 0.286194 | 0.249879 | 0.256512 | 0.352208 |
| cg04488111 | 2.62E-04 | 1 | rs189602506;rs200532153; rs183331301;rs542482741; rs112859764 | 0.321314 | 0.280121 | 0.294168 | NA |
| cg23548201 | 2.62E-04 | 3 |  | 0.406223 | 0.383315 | 0.373935 | 0.503938 |
| cg11081255 | 2.61E-04 | 1 |  | 0.714194 | 0.692538 | 0.710335 | 0.750959 |
| cg18296743 | 2.61E-04 | 20 | rs116716366;rs73299319 | 0.32475 | 0.304448 | 0.294958 | 0.3961 |
| cg15275312 | 2.61E-04 | 3 | rs72932983 | 0.234114 | 0.209822 | 0.196914 | 0.296212 |
| cg02334333 | 2.61E-04 | 2 | rs201706698;rs534998425 | 0.274246 | 0.24641 | 0.23352 | 0.334545 |
| cg11373212 | 2.61E-04 | 3 | rs147903964;rs551550573; rs571395308;rs537312109; rs114370608 | 0.33367 | 0.297221 | 0.28666 | 0.423232 |
| cg26419676 | 2.61E-04 | 1 | rs146047869 | 0.520032 | 0.499221 | 0.512033 | 0.606911 |
| cg19701828 | 2.60E-04 | 2 | rs551671468;rs566852487; rs183404482;rs555968357 | 0.272589 | 0.248016 | 0.238891 | 0.335529 |
| cg07532960 | 2.60E-04 | 1 | rs74055029 | 0.256395 | 0.235128 | 0.235197 | 0.315423 |
| cg14502521 | 2.60E-04 | 1 | rs142942930;rs545323011; rs182309865;rs60086105 | 0.352448 | 0.322 | 0.317594 | 0.422457 |
| cg04718896 | 2.60E-04 | 16 | rs535786179 | 0.147215 | 0.121907 | 0.124698 | 0.168698 |
| cg16436762 | 2.59E-04 | 11 | rs142326519 | 0.226297 | 0.205843 | 0.190783 | 0.299187 |
| cg10881749 | 2.58E-04 | 1 |  | 0.408647 | 0.382784 | 0.3972 | 0.461679 |
| cg06149863 | 2.57E-04 | 15 | rs569586583 | 0.209578 | 0.181342 | 0.187259 | 0.251328 |
| cg06897797 | 2.57E-04 | 9 | rs572283210;rs572511197 | 0.288095 | 0.260926 | 0.255531 | 0.343268 |
| cg01168584 | 2.57E-04 | 18 | rs539994603;rs540731299; rs71356079;rs75159164 | 0.271599 | 0.236563 | 0.233691 | 0.357898 |
| cg00353789 | 2.56E-04 | 16 | rs143280975;rs35342456; rs567370731 | 0.311679 | 0.288812 | 0.28242 | 0.379386 |
| cg05652126 | 2.56E-04 | 15 | rs534935340;rs555088635; rs571982850;rs7169298 | 0.250684 | 0.221874 | 0.220179 | 0.302529 |
| cg05382012 | 2.56E-04 | 16 | rs532143386 | 0.324622 | 0.295256 | 0.295215 | 0.391037 |
| cg01055075 | 2.55E-04 | 7 | rs3889797;rs548031736; rs117631538 | 0.726889 | 0.706761 | 0.717249 | 0.767425 |
| cg17484029 | 2.55E-04 | 2 | rs569627921;rs189441966; rs548812714 | 0.172139 | 0.14927 | 0.143164 | 0.219513 |
| cg08173439 | 2.55E-04 | 19 |  | 0.44168 | 0.406218 | 0.395193 | 0.5139 |
| cg18349527 | 2.55E-04 | 12 |  | 0.353664 | 0.332152 | 0.333912 | 0.424891 |
| cg08130912 | 2.54E-04 | 14 | rs376962725;rs565160117 | 0.215076 | 0.186067 | 0.190046 | 0.25936 |
| cg10604836 | 2.53E-04 | 1 | rs547014215;rs6694681; rs534600720;rs191531861 | 0.307985 | 0.281526 | 0.264817 | 0.390312 |
| cg13645963 | 2.53E-04 | 9 | rs117794296;rs562093440 | 0.340102 | 0.309441 | 0.30329 | 0.421566 |
| cg13932501 | 2.53E-04 | 9 | rs543711693 | 0.367145 | 0.341006 | 0.342869 | 0.453214 |
| cg05623986 | 2.53E-04 | 6 | rs536638637 | 0.554789 | 0.522837 | 0.527811 | 0.624584 |
| cg24954325 | 2.52E-04 | 17 | rs554842312;rs113600613; rs7212079;rs554042232 | 0.328463 | 0.298009 | 0.297625 | 0.388382 |
| cg11034147 | 2.52E-04 | 14 | rs567120104;rs17127736; rs554463240;rs188386703 | 0.32893 | 0.30802 | 0.296941 | 0.404461 |
| cg09641170 | 2.52E-04 | 11 | rs567031855 | 0.283171 | 0.261269 | 0.246352 | 0.350119 |
| cg13901842 | 2.52E-04 | 2 | rs573942933;rs183102457; rs148838603;rs4625887 | 0.283731 | 0.254375 | 0.245575 | 0.374315 |
| cg25150026 | 2.52E-04 | 4 | rs11939668;rs566955757 | 0.227297 | 0.197551 | 0.195467 | 0.274952 |
| cg23517697 | 2.52E-04 | 20 |  | 0.295642 | 0.269248 | 0.270542 | 0.358119 |
| cg11675448 | 2.51E-04 | 22 | rs114198095 | 0.213945 | 0.186846 | 0.190723 | 0.263396 |
| cg00519063 | 2.51E-04 | 1 | rs543869121;rs17879576; rs545408175 | 0.54977 | 0.526379 | 0.522029 | 0.598839 |
| cg05639399 | 2.51E-04 | 18 | rs75234040 | 0.260267 | 0.222198 | 0.214338 | 0.317379 |
| cg00592245 | 2.51E-04 | 7 | rs10243271 | 0.279035 | 0.256936 | 0.250118 | 0.362602 |
| cg21519701 | 2.51E-04 | 17 | rs142741186;rs181917994; rs575274084 | 0.269832 | 0.228291 | 0.221046 | 0.335152 |
| cg22747843 | 2.50E-04 | 1 | rs79798414 | 0.249384 | 0.219131 | 0.211767 | 0.326832 |
| cg13827496 | 2.50E-04 | 7 | rs570861747;rs531182244 | 0.258692 | 0.234035 | 0.232939 | 0.326549 |
| cg05053920 | 2.50E-04 | 13 |  | 0.252196 | 0.227777 | 0.213647 | 0.326048 |
| cg14120403 | 2.49E-04 | 10 | rs72810504;rs187892390; rs566313104;rs533667136; rs193143883 | 0.387071 | 0.357593 | 0.355433 | 0.454606 |
| cg15195477 | 2.48E-04 | 1 | rs548289089 | 0.342095 | 0.318546 | 0.307822 | 0.388292 |
| cg00560428 | 2.47E-04 | 22 |  | 0.438109 | 0.414305 | 0.415226 | 0.488642 |
| cg26173962 | 2.47E-04 | 1 | rs374875939 | 0.32404 | 0.282508 | 0.277986 | 0.425347 |
| cg05511431 | 2.47E-04 | 6 |  | 0.311701 | 0.283198 | 0.277253 | 0.375816 |
| cg04304036 | 2.47E-04 | 16 | rs111888218;rs186630157 | 0.262124 | 0.235529 | 0.228009 | 0.345257 |
| cg26783127 | 2.46E-04 | 17 | rs562079877;rs115697474 | 0.275148 | 0.247842 | 0.242366 | 0.369131 |
| cg19356022 | 2.46E-04 | 1 | rs540730736;rs565206784 | 0.281504 | 0.249043 | 0.262123 | 0.368058 |
| cg07677920 | 2.45E-04 | 17 | rs184589375;rs529501008; rs547693945;rs144216206 | 0.278022 | 0.25242 | 0.246544 | 0.353298 |
| cg13616508 | 2.45E-04 | 7 | rs112298062;rs143567403 | 0.14139 | 0.120123 | 0.127924 | 0.175905 |
| cg14227037 | 2.45E-04 | 8 | rs560007908;rs75223170; rs545689733;rs142538250; rs531229684 | 0.631376 | 0.609895 | 0.601948 | 0.70024 |
| cg02311193 | 2.45E-04 | 17 |  | 0.323308 | 0.29956 | 0.297407 | 0.388896 |
| cg24591627 | 2.45E-04 | 20 | rs577926635 | 0.18197 | 0.158175 | 0.160919 | 0.217148 |
| cg23091673 | 2.45E-04 | 7 | rs182467885;rs186798045 | 0.482061 | 0.459478 | 0.479213 | 0.549947 |
| cg21932814 | 2.44E-04 | 3 | rs553834864;rs191500425 | 0.272198 | 0.236915 | 0.230961 | 0.338741 |
| cg04202047 | 2.43E-04 | 17 | rs529400938;rs547231835; rs565562155 | 0.251725 | 0.221332 | 0.228922 | 0.327884 |
| cg19503687 | 2.43E-04 | 22 | rs564433623;rs528772345 | 0.307983 | 0.271056 | 0.256631 | 0.386104 |
| cg05065948 | 2.43E-04 | 4 | rs527340591;rs148407045; rs564697502;rs11933748 | 0.378549 | 0.35045 | 0.347434 | 0.457285 |
| cg24155358 | 2.43E-04 | 11 | rs545223885;rs181478877; rs186455857 | 0.293475 | 0.255141 | 0.248565 | 0.383136 |
| cg05642546 | 2.43E-04 | 7 | rs142772798;rs189550980 | 0.277606 | 0.238395 | 0.231129 | 0.343695 |
| cg07433407 | 2.42E-04 | 1 | rs151282416;rs112320314 | 0.254141 | 0.227882 | 0.209768 | 0.323765 |
| cg12391815 | 2.42E-04 | 10 | rs545616865;rs187081401; rs528125122 | 0.237454 | 0.206005 | 0.190374 | 0.290206 |
| cg20561863 | 2.42E-04 | 17 | rs143560576 | 0.16487 | 0.139824 | 0.135003 | 0.2064 |
| cg00842918 | 2.42E-04 | 17 | rs183966271 | 0.318431 | 0.284137 | 0.285174 | 0.406449 |
| cg21238327 | 2.42E-04 | 1 | rs187178503;rs191402152; rs76589933;rs532034006; rs578214332 | 0.313019 | 0.283025 | 0.277916 | 0.381598 |
| cg00916652 | 2.42E-04 | 3 | rs147168765 | 0.295793 | 0.258868 | 0.249219 | 0.366055 |
| cg14578677 | 2.41E-04 | 4 | rs76321546;rs201448861; rs147516165;rs140034778 | 0.320714 | 0.293251 | 0.286549 | 0.40201 |
| cg06500418 | 2.41E-04 | 2 | rs547191800 | 0.329424 | 0.291302 | 0.296865 | 0.423112 |
| cg09043230 | 2.41E-04 | 16 | rs115710854;rs138651905; rs547274735 | 0.289566 | 0.266617 | 0.253628 | 0.369514 |
| cg16206394 | 2.39E-04 | 12 | rs567623512;rs536545847; rs553184399 | 0.316801 | 0.27819 | 0.276962 | 0.386119 |
| cg19948478 | 2.39E-04 | 1 | rs567484372;rs146560341; rs546962791 | 0.315023 | 0.28778 | 0.270694 | 0.415469 |
| cg03634071 | 2.39E-04 | 12 |  | 0.233919 | 0.210532 | 0.211973 | 0.321694 |
| cg15645600 | 2.39E-04 | 1 | rs6662507;rs550773010 | 0.27501 | 0.247286 | 0.242534 | 0.360203 |
| cg10034788 | 2.39E-04 | 12 | rs563849597;rs575942993 | 0.336277 | 0.300364 | 0.299877 | 0.431938 |
| cg22716663 | 2.39E-04 | 22 |  | 0.296553 | 0.25078 | 0.243413 | 0.351726 |
| cg03948938 | 2.39E-04 | 9 | rs573544369;rs539871413; rs558486629 | 0.354883 | 0.325758 | 0.321771 | 0.425274 |
| cg00514723 | 2.39E-04 | 20 | rs183333312 | 0.253082 | 0.227921 | 0.231868 | 0.309133 |
| cg04814136 | 2.38E-04 | 12 | rs142889035;rs539370071; rs541399415;rs564796031 | 0.280725 | 0.248664 | 0.249809 | 0.373459 |
| cg16032803 | 2.38E-04 | 12 | rs188084135;rs118070948 | 0.322925 | 0.29256 | 0.293571 | 0.382107 |
| cg16814392 | 2.38E-04 | 13 | rs563393576;rs576634384; rs7332691;rs559622755; rs528406439 | 0.374816 | 0.353103 | 0.336381 | 0.456426 |
| cg08920032 | 2.38E-04 | 15 | rs547869150;rs117944139 | 0.333928 | 0.295713 | 0.295064 | 0.4201 |
| cg03440799 | 2.38E-04 | 1 | rs185292080;rs113915235 | 0.377387 | 0.347013 | 0.358532 | 0.443228 |
| cg16097772 | 2.37E-04 | 12 | rs513342;rs58131341; rs531119114 | 0.278858 | 0.258792 | 0.257324 | 0.364504 |
| cg02929855 | 2.37E-04 | 12 | rs535524485;rs34885511; rs11061983;rs182556671; rs557669511;rs573050792; rs533750504 | 0.313077 | 0.279462 | 0.277499 | 0.397638 |
| cg14688689 | 2.37E-04 | 21 | rs376636972 | 0.486861 | 0.459158 | 0.465358 | 0.551584 |
| cg25900192 | 2.37E-04 | 10 | rs541164427 | 0.315109 | 0.279642 | 0.278719 | 0.414047 |
| cg21768918 | 2.37E-04 | 10 | rs570763315;rs539618555; rs546495725 | 0.293436 | 0.260333 | 0.25085 | 0.379886 |
| cg10520672 | 2.37E-04 | 16 | rs2280381;rs146779937 | 0.21073 | 0.187081 | 0.1882 | 0.264166 |
| cg21335396 | 2.37E-04 | 9 | rs146440492;rs562167925 | 0.297851 | 0.245524 | 0.248034 | 0.38167 |
| cg21571073 | 2.37E-04 | 11 | rs527398739;rs541098301 | 0.658242 | 0.636523 | 0.648853 | 0.70197 |
| cg02764373 | 2.37E-04 | 1 | rs537243607 | 0.182776 | 0.159553 | 0.152947 | 0.232173 |
| cg20040976 | 2.36E-04 | 15 | rs186744734;rs571012443 | 0.313543 | 0.291504 | 0.297172 | 0.390314 |
| cg21489364 | 2.36E-04 | 16 | rs550193222;rs570242595 | 0.401375 | 0.366274 | 0.36433 | 0.454548 |
| cg13852385 | 2.36E-04 | 3 | rs147420971 | 0.233228 | 0.206586 | 0.198483 | 0.294547 |
| cg22898055 | 2.36E-04 | 7 |  | 0.250295 | 0.223264 | 0.211834 | 0.314448 |
| cg25205240 | 2.35E-04 | 19 | rs549975694;rs11668486; rs571228767;rs139654260; rs553754509;rs367883984; rs1541924 | 0.514174 | 0.492053 | 0.494011 | 0.574042 |
| cg04993279 | 2.35E-04 | 1 | rs565700311 | 0.292171 | 0.260609 | 0.255033 | 0.357774 |
| cg15210526 | 2.35E-04 | 1 | rs541672974 | 0.320026 | 0.287214 | 0.282254 | 0.406439 |
| cg23145714 | 2.35E-04 | 6 | rs373394966;rs575889965; rs143366995 | 0.26788 | 0.240917 | 0.228129 | 0.340124 |
| cg02468551 | 2.35E-04 | 19 |  | 0.372207 | 0.333209 | 0.337543 | 0.450598 |
| cg19584038 | 2.35E-04 | 19 | rs115928860;rs180678845 | 0.278438 | 0.246298 | 0.246816 | 0.317807 |
| cg17009294 | 2.35E-04 | 2 |  | 0.28615 | 0.256641 | 0.245709 | 0.369252 |
| cg26147268 | 2.35E-04 | 1 | rs529830003;rs540780247; rs560524991 | 0.360449 | 0.339657 | 0.338001 | 0.411885 |
| cg00145289 | 2.34E-04 | 12 |  | 0.360746 | 0.336629 | 0.329781 | 0.416068 |
| cg04012574 | 2.34E-04 | 10 | rs548835822;rs188739096 | 0.300443 | 0.26101 | 0.266949 | 0.371988 |
| cg06236715 | 2.34E-04 | 1 |  | 0.328649 | 0.295437 | 0.295743 | 0.430005 |
| cg18981616 | 2.32E-04 | 20 | rs577180085 | 0.295001 | 0.26851 | 0.263292 | 0.361843 |
| cg05867620 | 2.32E-04 | 5 | rs185417581;rs533153394 | 0.380191 | 0.347827 | 0.331895 | 0.474164 |
| cg24895173 | 2.32E-04 | 17 | rs530395593;rs41324748; rs567389237 | 0.223063 | 0.200446 | 0.205921 | 0.283963 |
| cg06030535 | 2.31E-04 | 5 |  | 0.246603 | 0.213753 | 0.205544 | 0.31495 |
| cg23598140 | 2.31E-04 | 10 | rs571280229 | 0.269711 | 0.245725 | 0.236321 | 0.359865 |
| cg22626169 | 2.31E-04 | 6 | rs543344177;rs187673504 | 0.280797 | 0.253818 | 0.245436 | 0.354304 |
| cg20354873 | 2.31E-04 | 14 | rs547358385;rs139483470 | 0.31345 | 0.281623 | 0.278684 | 0.377575 |
| cg13844341 | 2.31E-04 | 7 | rs10254998;rs138304523; rs373791503 | 0.447515 | 0.39812 | 0.399009 | 0.521438 |
| cg11173131 | 2.31E-04 | 20 | rs536454073;rs201982370; rs6017912;rs190560552 | 0.22687 | 0.201846 | 0.202246 | 0.285445 |
| cg23697633 | 2.31E-04 | 13 | rs545741639;rs191863678; rs61962689 | 0.298388 | 0.272742 | 0.265356 | 0.372285 |
| cg02823530 | 2.31E-04 | 15 | rs533387183;rs147452749 | 0.386512 | 0.360815 | 0.355974 | 0.46945 |
| cg09200921 | 2.30E-04 | 13 | rs533860175 | 0.195898 | 0.167288 | 0.177493 | 0.235886 |
| cg21104835 | 2.30E-04 | 1 | rs376688623;rs198357; rs184222829 | 0.31774 | 0.291719 | 0.274687 | 0.419842 |
| cg10151645 | 2.29E-04 | 22 | rs564840476 | 0.304053 | 0.276555 | 0.266952 | 0.371249 |
| cg24069172 | 2.29E-04 | 11 | rs143008941 | 0.236142 | 0.203008 | 0.199868 | 0.295329 |
| cg14990796 | 2.29E-04 | 7 | rs186866744;rs73048969; rs535982303;rs562432505; rs113188198 | 0.358537 | 0.336448 | 0.335562 | 0.419819 |
| cg16909536 | 2.28E-04 | 17 |  | 0.251211 | 0.229093 | 0.225076 | 0.30693 |
| cg00724332 | 2.27E-04 | 12 |  | 0.293045 | 0.256542 | 0.255029 | 0.360402 |
| cg12196294 | 2.27E-04 | 3 | rs562027244;rs146870239 | 0.291304 | 0.259663 | 0.246756 | 0.373804 |
| cg20092399 | 2.26E-04 | 7 | rs540751839 | 0.490236 | 0.469971 | 0.483096 | 0.561311 |
| cg00159243 | 2.26E-04 | 12 | rs189000816 | 0.319717 | 0.290932 | 0.285379 | 0.377945 |
| cg23453794 | 2.26E-04 | 2 | rs189883311;rs182450632 | 0.291878 | 0.254551 | 0.246371 | 0.355708 |
| cg04355430 | 2.26E-04 | 21 |  | 0.247519 | 0.217919 | 0.214004 | 0.316361 |
| cg24352985 | 2.25E-04 | 2 | rs140319726 | 0.249102 | 0.228128 | 0.211243 | 0.316594 |
| cg24529979 | 2.25E-04 | 19 | rs145400997;rs570041542 | 0.307203 | 0.273295 | 0.259911 | 0.378615 |
| cg13448633 | 2.25E-04 | 5 | rs557603404 | 0.299065 | 0.260552 | 0.249462 | 0.382398 |
| cg04211197 | 2.25E-04 | 17 | rs554460104 | 0.227373 | 0.20433 | 0.190232 | 0.317198 |
| cg07870177 | 2.24E-04 | 2 | rs145415390 | 0.332972 | 0.309295 | 0.313187 | 0.42197 |
| cg10954073 | 2.24E-04 | 7 | rs188299714;rs559155462 | 0.345595 | 0.317206 | 0.306837 | 0.440543 |
| cg12172984 | 2.23E-04 | 15 | rs567671329;rs537877574 | 0.373906 | 0.35355 | 0.348344 | 0.490494 |
| cg00456448 | 2.23E-04 | 1 | rs142672617 | 0.378083 | 0.353866 | 0.356192 | 0.440119 |
| cg05749408 | 2.23E-04 | 3 | rs186220087 | 0.30568 | 0.278745 | 0.274147 | 0.388657 |
| cg14186336 | 2.23E-04 | 9 | rs184163495 | 0.316776 | 0.292092 | 0.294426 | 0.375688 |
| cg00747928 | 2.23E-04 | 11 |  | 0.263433 | 0.240461 | 0.241055 | 0.340753 |
| cg19104015 | 2.23E-04 | 17 | rs147476652 | 0.172583 | 0.147782 | 0.153958 | 0.224612 |
| cg00778525 | 2.22E-04 | 5 | rs73816546;rs573590125; rs188272147;rs28523545 | 0.296395 | 0.240529 | 0.232908 | 0.375503 |
| cg22800543 | 2.22E-04 | 14 | rs572367218;rs7156523; rs560911360;rs114919062 | 0.421191 | 0.393002 | 0.400729 | 0.511964 |
| cg04281169 | 2.22E-04 | 15 | rs565958351 | 0.146909 | 0.121353 | 0.128229 | 0.202111 |
| cg06197624 | 2.22E-04 | 4 | rs537323324;rs191014348; rs567548214;rs536602482; rs142376956 | 0.229331 | 0.207045 | 0.205638 | 0.295393 |
| cg26538140 | 2.22E-04 | 12 |  | 0.270036 | 0.24538 | 0.236391 | 0.336314 |
| cg20370152 | 2.21E-04 | 2 |  | 0.146417 | 0.123732 | 0.124307 | 0.195406 |
| cg03879730 | 2.21E-04 | 1 | rs587633858 | 0.785398 | 0.764898 | 0.783014 | 0.810167 |
| cg16032884 | 2.21E-04 | 3 | rs147080770;rs549683573; rs567976472;rs183914176 | 0.290158 | 0.246562 | 0.249447 | 0.363199 |
| cg18266414 | 2.21E-04 | 4 | rs569986092;rs191843640; rs74367552;rs77953099 | 0.332066 | 0.290918 | 0.278492 | 0.421857 |
| cg16123939 | 2.21E-04 | 2 | rs559357820;rs181329286; rs537889741 | 0.30953 | 0.267191 | 0.26421 | 0.38314 |
| cg23895848 | 2.20E-04 | 15 | rs199588319;rs535096598; rs11371388 | 0.298687 | 0.269726 | 0.270508 | 0.370249 |
| cg22437405 | 2.20E-04 | 1 | rs115232311 | 0.330879 | 0.309586 | 0.299073 | 0.398552 |
| cg07774556 | 2.20E-04 | 5 | rs558578387 | 0.322244 | 0.28714 | 0.285635 | 0.398547 |
| cg10671063 | 2.20E-04 | 6 |  | 0.246942 | 0.226788 | 0.229724 | 0.308936 |
| cg07910010 | 2.20E-04 | 2 | rs112671761;rs531820297 | 0.367197 | 0.327924 | 0.331652 | 0.472166 |
| cg15299926 | 2.19E-04 | 8 | rs111674373 | 0.303169 | 0.270704 | 0.255453 | 0.374052 |
| cg25235344 | 2.19E-04 | 19 | rs145327239 | 0.268909 | 0.238494 | 0.232993 | 0.335939 |
| cg05244667 | 2.18E-04 | 20 | rs527884644 | 0.266849 | 0.243577 | 0.230323 | 0.317474 |
| cg10605434 | 2.18E-04 | 11 | rs541044234;rs188516795 | 0.293516 | 0.269687 | 0.254902 | 0.351419 |
| cg13578165 | 2.18E-04 | 17 | rs572888604 | 0.525868 | 0.505348 | 0.503087 | 0.588238 |
| cg08700083 | 2.18E-04 | 4 | rs534501046 | 0.243176 | 0.220028 | 0.22237 | 0.323314 |
| cg16991143 | 2.17E-04 | 17 | rs138301112;rs149616725; rs78739684;rs146112182; rs565810436 | 0.241379 | 0.210468 | 0.204228 | 0.289453 |
| cg11663015 | 2.17E-04 | 20 | rs184205028 | 0.38913 | 0.36899 | 0.37525 | 0.482724 |
| cg00274428 | 2.17E-04 | 1 | rs144715908;rs12122194 | 0.612984 | 0.585778 | 0.589325 | 0.682197 |
| cg05944352 | 2.15E-04 | 4 | rs566102184 | 0.307553 | 0.277768 | 0.278716 | 0.396139 |
| cg06777477 | 2.15E-04 | 15 | rs547827987;rs566732093; rs11857192 | 0.303641 | 0.282967 | 0.279209 | 0.371866 |
| cg12655112 | 2.14E-04 | 15 | rs375608678;rs146180999; rs189893570 | 0.663992 | 0.642216 | 0.643117 | 0.710113 |
| cg20203469 | 2.14E-04 | 11 |  | 0.423986 | 0.395649 | 0.396645 | 0.514391 |
| cg03448068 | 2.13E-04 | 1 | rs551826363;rs4512673; rs534344333;rs546273762 | 0.233854 | 0.205381 | 0.200721 | 0.295631 |
| cg14659184 | 2.13E-04 | 15 | rs191168955;rs117382057 | 0.238313 | 0.205864 | 0.198438 | 0.313665 |
| cg09860323 | 2.13E-04 | 6 |  | 0.383032 | 0.355725 | 0.350041 | 0.46169 |
| cg02904191 | 2.13E-04 | 2 | rs546498861;rs566483813; rs535527625 | 0.252814 | 0.214326 | 0.213503 | 0.334168 |
| cg05538508 | 2.13E-04 | 19 | rs112499596;rs538153141 | 0.381316 | 0.35833 | 0.351998 | 0.453032 |
| cg20164557 | 2.13E-04 | 4 |  | 0.295464 | 0.252116 | 0.249055 | 0.371225 |
| cg03052078 | 2.13E-04 | 6 | rs561912735 | 0.278014 | 0.245688 | 0.240058 | 0.357853 |
| cg04602039 | 2.12E-04 | 3 | rs371047371;rs370454555 | 0.170614 | 0.148869 | 0.154405 | 0.22047 |
| cg20780105 | 2.12E-04 | 5 | rs143222866;rs143525064 | 0.313368 | 0.254575 | 0.268828 | 0.385974 |
| cg01463294 | 2.12E-04 | 3 |  | 0.269851 | 0.24275 | 0.244019 | 0.333868 |
| cg11845002 | 2.12E-04 | 3 | rs11714512 | 0.553156 | 0.527654 | 0.529344 | 0.619596 |
| cg17916766 | 2.12E-04 | 6 | rs117151882;rs550085812 | 0.308982 | 0.268489 | 0.265012 | 0.389231 |
| cg14607332 | 2.11E-04 | 1 |  | 0.534998 | 0.514896 | 0.527288 | 0.617351 |
| cg22476055 | 2.11E-04 | 16 | rs181381517;rs540935048; rs4781031 | 0.346614 | 0.319636 | 0.323297 | 0.418273 |
| cg14703454 | 2.11E-04 | 3 | rs187808490 | 0.257299 | 0.206225 | 0.208338 | 0.329633 |
| cg17649852 | 2.11E-04 | 12 | rs187259027;rs371811010; rs577352066;rs190120098; rs11048530 | 0.244045 | 0.217576 | 0.210164 | 0.314488 |
| cg08943386 | 2.11E-04 | 20 | rs55679506;rs539677411; rs116097743 | 0.175399 | 0.153075 | 0.137112 | 0.217141 |
| cg26307396 | 2.11E-04 | 3 | rs527844170;rs552511284; rs571131689 | 0.291147 | 0.256608 | 0.251105 | 0.365306 |
| cg02387312 | 2.10E-04 | 2 | rs559249922 | 0.240663 | 0.216626 | 0.20519 | 0.306898 |
| cg26823336 | 2.10E-04 | 11 | rs552954012;rs537803696 | 0.304533 | 0.266872 | 0.258934 | 0.377804 |
| cg22957691 | 2.09E-04 | 2 | rs542957341 | 0.277383 | 0.248566 | 0.239055 | 0.376575 |
| cg11194061 | 2.09E-04 | 3 | rs149269787;rs565758056; rs539182612 | 0.354551 | 0.311963 | 0.325519 | 0.441218 |
| cg12195441 | 2.08E-04 | 15 | rs75310579 | 0.345242 | 0.317769 | 0.316929 | 0.411988 |
| cg03714676 | 2.08E-04 | 8 |  | 0.332299 | 0.309498 | 0.305096 | 0.428489 |
| cg01227199 | 2.08E-04 | 1 | rs113741245 | 0.283642 | 0.245957 | 0.251416 | 0.37235 |
| cg09255910 | 2.08E-04 | 1 | rs550926605;rs116339733 | 0.356569 | 0.334402 | 0.330413 | 0.410414 |
| cg10125918 | 2.08E-04 | 2 | rs115647254;rs148432325; rs567891705 | 0.256854 | 0.231266 | 0.222005 | 0.373849 |
| cg10327881 | 2.08E-04 | 15 |  | 0.267236 | 0.245254 | 0.235447 | 0.345578 |
| cg16395527 | 2.08E-04 | 3 | rs528541411 | 0.269296 | 0.242759 | 0.235082 | 0.343007 |
| cg21039723 | 2.07E-04 | 2 | rs189321568;rs565978948 | 0.402325 | 0.37781 | 0.386145 | 0.481371 |
| cg26402169 | 2.07E-04 | 7 | rs571447687;rs148702565; rs547327854 | 0.739208 | 0.715519 | 0.71239 | 0.842644 |
| cg04623471 | 2.06E-04 | 3 | rs578094344 | 0.301113 | 0.276927 | 0.286667 | 0.350986 |
| cg18377857 | 2.06E-04 | 5 |  | 0.299626 | 0.277344 | 0.259766 | 0.388183 |
| cg11629026 | 2.06E-04 | 7 | rs114981293;rs575081499 | 0.317359 | 0.291904 | 0.288905 | 0.377665 |
| cg15793246 | 2.05E-04 | 12 | rs182980412 | 0.338143 | 0.297907 | 0.279474 | 0.434504 |
| cg24731096 | 2.05E-04 | 9 |  | 0.561811 | 0.538551 | 0.542256 | 0.616265 |
| cg09145126 | 2.05E-04 | 13 | rs528221479;rs546262728 | 0.294382 | 0.256517 | 0.268341 | 0.370393 |
| cg08160072 | 2.04E-04 | 2 | rs74452415;rs145237872; rs563921571 | 0.306781 | 0.282893 | 0.269903 | 0.408986 |
| cg04525074 | 2.04E-04 | 15 | rs561506684;rs570751751; rs138604070 | 0.237163 | 0.212917 | 0.211371 | 0.295432 |
| cg14691324 | 2.04E-04 | 11 | rs575540875;rs543932550 | 0.29462 | 0.265533 | 0.258327 | 0.368036 |
| cg15510965 | 2.03E-04 | 1 | rs562608590;rs547003795 | 0.276361 | 0.249636 | 0.242705 | 0.355091 |
| cg13562751 | 2.03E-04 | 6 | rs4379290 | 0.665897 | 0.642046 | 0.646402 | 0.70347 |
| cg10228304 | 2.02E-04 | 11 |  | 0.297885 | 0.268283 | 0.263127 | 0.365656 |
| cg15678744 | 2.02E-04 | 5 | rs185599689;rs368491449 | 0.643318 | 0.610379 | 0.626515 | 0.684549 |
| cg03237699 | 2.02E-04 | 20 | rs565030847;rs138981917; rs545455146 | 0.294845 | 0.259601 | 0.242993 | 0.352281 |
| cg11308512 | 2.02E-04 | 19 | rs541988886 | 0.308869 | 0.287429 | 0.28971 | 0.359844 |
| cg12078492 | 2.02E-04 | 3 |  | 0.531118 | 0.494604 | 0.49793 | 0.597033 |
| cg17352215 | 2.02E-04 | 15 | rs541120524 | 0.403978 | 0.375926 | 0.374717 | 0.488119 |
| cg16356622 | 2.01E-04 | 1 | rs566651312;rs534707104; rs552914889 | 0.329857 | 0.308939 | 0.299931 | 0.390136 |
| cg11363999 | 2.01E-04 | 21 | rs7276825;rs563630600 | 0.272628 | 0.239253 | 0.223574 | 0.333587 |
| cg13971866 | 2.01E-04 | 2 | rs35313480;rs151184011; rs199624410;rs1054737 | 0.53571 | 0.515364 | 0.522679 | 0.585954 |
| cg01411400 | 2.01E-04 | 17 | rs143502014 | 0.302694 | 0.270623 | 0.267595 | 0.370686 |
| cg02331761 | 2.01E-04 | 3 | rs531780755 | 0.346902 | 0.312521 | 0.303761 | 0.431426 |
| cg14864966 | 2.01E-04 | 5 | rs551393655;rs560511829; rs527787943;rs549419462 | 0.340073 | 0.308122 | 0.315919 | 0.441011 |
| cg23884784 | 2.00E-04 | 14 | rs562393854 | 0.216875 | 0.193126 | 0.201293 | 0.263319 |
| cg07394019 | 2.00E-04 | 11 | rs561823860 | 0.292567 | 0.26262 | 0.251366 | 0.394109 |
| cg05083015 | 2.00E-04 | 1 | rs534757953 | 0.300943 | 0.275059 | 0.256137 | 0.381246 |
| cg08557347 | 1.99E-04 | 16 | rs576018761;rs533846286 | 0.401288 | 0.378251 | 0.386608 | 0.459238 |
| cg03149787 | 1.99E-04 | 5 | rs561294393;rs202061857 | 0.256852 | 0.219658 | 0.21307 | 0.343385 |
| cg15418499 | 1.99E-04 | 11 | rs143370581;rs538983532 | 0.276182 | 0.250604 | 0.246899 | 0.352331 |
| cg12057878 | 1.99E-04 | 2 | rs111794962 | 0.295379 | 0.256244 | 0.253652 | 0.375863 |
| cg23506375 | 1.98E-04 | 18 | rs146924020;rs532674601; rs147975978;rs187573930 | 0.25394 | 0.223972 | 0.214472 | 0.354947 |
| cg15885703 | 1.98E-04 | 11 | rs116103424 | 0.516077 | 0.463216 | 0.446009 | 0.510964 |
| cg16967583 | 1.98E-04 | 2 |  | 0.264867 | 0.240666 | 0.240017 | 0.326807 |
| cg13206224 | 1.98E-04 | 4 | rs193287298 | 0.225668 | 0.195849 | 0.199375 | 0.276122 |
| cg14138235 | 1.98E-04 | 14 | rs139860498;rs377141496; rs573052744 | 0.309137 | 0.284166 | 0.282381 | 0.386296 |
| cg26284800 | 1.98E-04 | 1 |  | 0.231417 | 0.210522 | 0.208851 | 0.330808 |
| cg15557438 | 1.98E-04 | 2 | rs562558149 | 0.369976 | 0.339095 | 0.341764 | 0.428302 |
| cg09244707 | 1.98E-04 | 7 | rs150445763;rs561539376; rs138275311 | 0.566202 | 0.538904 | 0.541811 | 0.622494 |
| cg05616969 | 1.97E-04 | 2 |  | 0.191448 | 0.166485 | 0.174708 | 0.241138 |
| cg05320933 | 1.97E-04 | 1 |  | 0.32176 | 0.298301 | 0.304731 | 0.396719 |
| cg24060856 | 1.97E-04 | 4 | rs142026559 | 0.334114 | 0.299006 | 0.297116 | 0.411552 |
| cg17831820 | 1.97E-04 | 15 | rs183940158;rs574037654 | 0.374742 | 0.346734 | 0.346156 | 0.443243 |
| cg21547649 | 1.96E-04 | 2 | rs181260419 | 0.271477 | 0.241213 | 0.241036 | 0.323347 |
| cg25648980 | 1.96E-04 | 12 |  | 0.313204 | 0.286309 | 0.27678 | 0.400425 |
| cg01203858 | 1.96E-04 | 6 | rs148313668 | 0.35465 | 0.331018 | 0.344816 | 0.418344 |
| cg04046364 | 1.95E-04 | 12 |  | 0.286501 | 0.254789 | 0.245953 | 0.347386 |
| cg17039883 | 1.95E-04 | 17 | rs78751881;rs114667899; rs541689929 | 0.307919 | 0.266285 | 0.262429 | 0.389716 |
| cg14020052 | 1.95E-04 | 2 | rs189667838;rs180914877 | 0.149241 | 0.124994 | 0.128775 | 0.180996 |
| cg04853319 | 1.95E-04 | 16 | rs553475289;rs151180121 | 0.356428 | 0.328247 | 0.331675 | 0.444026 |
| cg07677892 | 1.94E-04 | 2 | rs181100597;rs550616841; rs62133271;rs561197013 | 0.273524 | 0.244562 | 0.238733 | 0.356213 |
| cg00711496 | 1.94E-04 | 19 | rs188398414 | 0.879973 | 0.859497 | 0.854168 | 0.909484 |
| cg09981220 | 1.94E-04 | 1 | rs530789537 | 0.297503 | 0.27187 | 0.263246 | 0.380439 |
| cg05822888 | 1.93E-04 | 17 | rs527266181 | 0.350548 | 0.317967 | 0.301684 | 0.4103 |
| cg00448525 | 1.93E-04 | 1 | rs201896158;rs202204798; rs191304686 | 0.289176 | 0.257926 | 0.253002 | 0.365867 |
| cg14594044 | 1.93E-04 | 4 | rs370239507 | 0.453513 | 0.432417 | 0.43989 | 0.519125 |
| cg16449581 | 1.93E-04 | 5 | rs147879662;rs543247259 | 0.358904 | 0.323405 | 0.314996 | 0.456481 |
| cg06785325 | 1.93E-04 | 1 | rs575743333;rs544571729; rs555737206;rs572384618 | 0.173452 | 0.148785 | 0.149417 | 0.189425 |
| cg26288821 | 1.93E-04 | 7 | rs10237852 | 0.253815 | 0.22512 | 0.215188 | 0.317791 |
| cg05957717 | 1.93E-04 | 1 | rs547240987;rs182104853; rs573014829;rs532779558 | 0.297081 | 0.256578 | 0.263696 | 0.381436 |
| cg22698501 | 1.93E-04 | 19 | rs575328190;rs544346455 | 0.334034 | 0.305369 | 0.298017 | 0.406287 |
| cg21810778 | 1.92E-04 | 1 | rs563442513;rs142237557 | 0.404161 | 0.380336 | 0.38139 | 0.466635 |
| cg20000185 | 1.92E-04 | 6 | rs553409178;rs565653796 | 0.288953 | 0.253274 | 0.249698 | 0.362258 |
| cg23054181 | 1.92E-04 | 15 | rs537835904;rs556109281; rs577970719 | 0.312576 | 0.279736 | 0.274487 | 0.393659 |
| cg09098522 | 1.92E-04 | 10 | rs561412538;rs529100918; rs550780431 | 0.280061 | 0.248005 | 0.250842 | 0.338719 |
| cg13667516 | 1.92E-04 | 6 | rs530716504 | 0.216294 | 0.194074 | 0.195164 | 0.277488 |
| cg03219393 | 1.91E-04 | 15 | rs112804879;rs78889995; rs542114756 | 0.264038 | 0.239845 | 0.248127 | 0.332771 |
| cg03418002 | 1.91E-04 | 4 |  | 0.293985 | 0.267794 | 0.264495 | 0.3947 |
| cg19057874 | 1.91E-04 | 10 |  | 0.28294 | 0.254111 | 0.25005 | 0.337738 |
| cg03614362 | 1.91E-04 | 4 | rs188618128 | 0.294306 | 0.259529 | 0.253083 | 0.379037 |
| cg08428726 | 1.90E-04 | 8 | rs554784475;rs573091925 | 0.287566 | 0.259724 | 0.251039 | 0.361383 |
| cg00524900 | 1.90E-04 | 5 | rs73247096 | 0.202582 | 0.174936 | 0.160801 | 0.251842 |
| cg09129050 | 1.90E-04 | 11 | rs149540967;rs556705217 | 0.360621 | 0.334044 | 0.323396 | 0.450063 |
| cg13643186 | 1.90E-04 | 3 | rs565867589;rs146916660 ;rs79764003 | 0.333987 | 0.302861 | 0.307169 | 0.417938 |
| cg12578063 | 1.90E-04 | 4 | rs112013157;rs548760925; rs142845963;rs371484377; rs553875738 | 0.363316 | 0.34098 | 0.340433 | 0.462824 |
| cg20760467 | 1.89E-04 | 22 | rs559655227;rs530183251; rs73879095 | 0.627288 | 0.60715 | 0.609378 | 0.659934 |
| cg10338518 | 1.89E-04 | 4 | rs559180661;rs17012226 | 0.280394 | 0.245329 | 0.243045 | 0.354713 |
| cg03882759 | 1.89E-04 | 12 |  | 0.169375 | 0.147894 | 0.151273 | 0.226096 |
| cg08871657 | 1.89E-04 | 3 | rs116393136;rs529705490; rs146307294 | 0.277817 | 0.242184 | 0.233523 | 0.357865 |
| cg25964609 | 1.89E-04 | 12 | rs558660647 | 0.247604 | 0.219655 | 0.211459 | 0.318563 |
| cg20378408 | 1.88E-04 | 12 | rs577931572 | 0.313539 | 0.279679 | 0.278207 | 0.376795 |
| cg05808565 | 1.88E-04 | 12 | rs536510876 | 0.270241 | 0.24693 | 0.232889 | 0.355825 |
| cg00224686 | 1.87E-04 | 8 | rs2623809 | 0.264198 | 0.231428 | 0.228307 | 0.333711 |
| cg04107129 | 1.87E-04 | 5 | rs184860809 | 0.34034 | 0.308425 | 0.290485 | 0.432517 |
| cg19774357 | 1.87E-04 | 1 | rs142274304 | 0.291291 | 0.258545 | 0.259248 | 0.351946 |
| cg20705966 | 1.86E-04 | 15 | rs74036978;rs543449683 | 0.300245 | 0.27678 | 0.280627 | 0.359638 |
| cg24052470 | 1.86E-04 | 2 | rs573205304;rs189148317 | 0.580197 | 0.559487 | 0.570213 | 0.648209 |
| cg25499678 | 1.86E-04 | 12 | rs80158047;rs114811910 | 0.346613 | 0.325487 | 0.310795 | 0.424369 |
| cg25197500 | 1.86E-04 | 17 | rs562444392;rs111575195 | 0.348824 | 0.314013 | 0.300636 | 0.461283 |
| cg04795186 | 1.86E-04 | 5 | rs543230810 | 0.231682 | 0.208178 | 0.199245 | 0.298558 |
| cg23247275 | 1.85E-04 | 8 |  | 0.321633 | 0.29322 | 0.293201 | 0.401712 |
| cg19982066 | 1.85E-04 | 3 | rs570857127 | 0.34636 | 0.321612 | 0.325725 | 0.424886 |
| cg07283015 | 1.85E-04 | 18 | rs560907397;rs529415718; rs549204503 | 0.334994 | 0.311334 | 0.305658 | 0.450347 |
| cg12511021 | 1.84E-04 | 14 | rs79380422 | 0.344933 | 0.311778 | 0.327652 | 0.448501 |
| cg24650171 | 1.84E-04 | 3 | rs542997026;rs186870556 | 0.290626 | 0.263156 | 0.26195 | 0.376939 |
| cg25582158 | 1.84E-04 | 3 | rs533194682;rs76116892; rs560415821;rs145732846 | 0.325726 | 0.278208 | 0.279908 | 0.394811 |
| cg05794482 | 1.84E-04 | 17 |  | 0.228342 | 0.20043 | 0.192784 | 0.302342 |
| cg00879685 | 1.84E-04 | 5 | rs531225369;rs372592060; rs375580342 | 0.414095 | 0.369255 | 0.351573 | 0.490408 |
| cg11427907 | 1.84E-04 | 8 |  | 0.286972 | 0.248309 | 0.2443 | 0.364321 |
| cg10490533 | 1.83E-04 | 8 | rs548594399;rs76918256; rs149033441 | 0.218273 | 0.190185 | 0.197164 | 0.293458 |
| cg17942388 | 1.83E-04 | 11 | rs528171991;rs547885604; rs150440660;rs550834768 | 0.603365 | 0.579583 | 0.590836 | 0.658287 |
| cg04928930 | 1.83E-04 | 14 | rs534666352;rs117872376; rs114198089;rs538275669 | 0.334948 | 0.313208 | 0.320341 | 0.426605 |
| cg10507445 | 1.83E-04 | 19 | rs569305125;rs536623628 | 0.358665 | 0.319334 | 0.313886 | 0.438835 |
| cg10289324 | 1.82E-04 | 18 |  | 0.26671 | 0.239129 | 0.238462 | 0.356191 |
| cg06576519 | 1.82E-04 | 4 | rs544378882;rs562678160; rs139447689;rs149248980 | 0.280505 | 0.24888 | 0.244397 | 0.348347 |
| cg14137166 | 1.82E-04 | 22 | rs577223138;rs149968337 | 0.310367 | 0.289203 | 0.280676 | 0.386338 |
| cg17746383 | 1.81E-04 | 1 | rs536173096 | 0.161772 | 0.141006 | 0.139905 | 0.208853 |
| cg23800226 | 1.81E-04 | 22 | rs566005444 | 0.276686 | 0.238448 | 0.232015 | 0.359018 |
| cg20369140 | 1.81E-04 | 13 | rs4769851;rs116046790; rs76090147 | 0.25139 | 0.222474 | 0.222114 | 0.326473 |
| cg02365539 | 1.81E-04 | 7 | rs573756760 | 0.452471 | 0.430864 | 0.445283 | 0.553512 |
| cg22497455 | 1.81E-04 | 2 |  | 0.283062 | 0.257354 | 0.244614 | 0.352414 |
| cg06567290 | 1.80E-04 | 11 | rs186567090;rs150026242 | 0.280407 | 0.252568 | 0.2533 | 0.348028 |
| cg11004181 | 1.80E-04 | 17 | rs151332874 | 0.306244 | 0.282652 | 0.267428 | 0.362764 |
| cg07102397 | 1.80E-04 | 14 | rs564828384;rs138443258 | 0.376458 | 0.349173 | 0.341256 | 0.475575 |
| cg22061818 | 1.79E-04 | 9 | rs540056319;rs560253262 | 0.318715 | 0.284847 | 0.272222 | 0.41799 |
| cg13047929 | 1.79E-04 | 3 | rs60461375;rs550352526; rs114136483;rs532687299 | 0.385113 | 0.364871 | 0.363142 | 0.487075 |
| cg17349544 | 1.79E-04 | 20 | rs536454073;rs201982370; rs6017912;rs190560552; rs16991469 | 0.216356 | 0.19128 | 0.187787 | 0.268012 |
| cg09779454 | 1.78E-04 | 7 | rs78796027 | 0.296694 | 0.256635 | 0.25602 | 0.371612 |
| cg04748721 | 1.78E-04 | 2 | rs138187481;rs181941055 | 0.298709 | 0.272205 | 0.267639 | 0.377706 |
| cg11426774 | 1.78E-04 | 17 |  | 0.257611 | 0.221362 | 0.21888 | 0.330634 |
| cg10834213 | 1.78E-04 | 2 | rs200816451;rs562134659; rs143647600;rs142888260; rs138209707 | 0.27223 | 0.248534 | 0.237346 | 0.340998 |
| cg24333795 | 1.77E-04 | 5 | rs372467211;rs537705227; rs74715453 | 0.345237 | 0.315361 | 0.312536 | 0.419207 |
| cg15294391 | 1.77E-04 | 10 |  | 0.619463 | 0.59303 | 0.603503 | 0.666772 |
| cg06885823 | 1.77E-04 | 5 | rs570262518;rs537233489; rs556265797 | 0.321727 | 0.296766 | 0.277837 | 0.39875 |
| cg03420467 | 1.77E-04 | 7 | rs558214384;rs141221635; rs540244059 | 0.291724 | 0.263209 | 0.257887 | 0.377964 |
| cg16295975 | 1.77E-04 | 1 | rs576288581;rs537270239; rs77676154 | 0.230083 | 0.195503 | 0.196024 | 0.279742 |
| cg19282986 | 1.76E-04 | 3 | rs576962113;rs111704254 | 0.310085 | 0.283528 | 0.277399 | 0.393106 |
| cg14554468 | 1.76E-04 | 17 | rs545199533;rs563597258; rs531250154 | 0.257109 | 0.226629 | 0.215647 | 0.319873 |
| cg02414559 | 1.76E-04 | 14 | rs147668411;rs180740545; rs529208984;rs542813722 | 0.242019 | 0.214945 | 0.212807 | 0.333988 |
| cg06465011 | 1.76E-04 | 16 | rs139118513;rs117249060 | 0.312563 | 0.281965 | 0.280458 | 0.405246 |
| cg11600734 | 1.76E-04 | 3 | rs1866998;rs139277643; rs184722918;rs149980531; rs188502883 | 0.236332 | 0.199524 | 0.204257 | 0.29961 |
| cg11599718 | 1.76E-04 | 12 | rs556453857;rs535279740 | 0.43075 | 0.410733 | 0.421833 | 0.475974 |
| cg14663914 | 1.76E-04 | 19 | rs534152403 | 0.302765 | 0.272726 | 0.261356 | 0.33765 |
| cg15979672 | 1.76E-04 | 7 | rs10488016 | 0.26998 | 0.241198 | 0.229698 | 0.358697 |
| cg11557901 | 1.76E-04 | 9 |  | 0.308285 | 0.272615 | 0.270539 | 0.413377 |
| cg00750518 | 1.75E-04 | 12 | rs542787979 | 0.488765 | 0.460335 | 0.462995 | 0.545779 |
| cg06032386 | 1.75E-04 | 10 |  | 0.247549 | 0.214736 | 0.204302 | 0.329292 |
| cg02622254 | 1.74E-04 | 2 |  | 0.310389 | 0.288361 | 0.279546 | 0.370763 |
| cg14893902 | 1.74E-04 | 8 | rs539359998 | 0.32057 | 0.293113 | 0.299282 | 0.384935 |
| cg23973532 | 1.74E-04 | 8 | rs142917652 | 0.591391 | 0.566418 | 0.556615 | 0.642977 |
| cg14654385 | 1.74E-04 | 11 | rs189147546 | 0.331985 | 0.304898 | 0.303118 | 0.39464 |
| cg04089901 | 1.73E-04 | 7 | rs574246720 | 0.276147 | 0.252218 | 0.244649 | 0.353434 |
| cg14476266 | 1.73E-04 | 2 |  | 0.303952 | 0.278973 | 0.277529 | 0.381687 |
| cg11940332 | 1.73E-04 | 9 | rs140310919 | 0.207784 | 0.181457 | 0.172711 | 0.246676 |
| cg24967811 | 1.73E-04 | 12 | rs555841674 | 0.258371 | 0.231152 | 0.230028 | 0.336767 |
| cg08599366 | 1.73E-04 | 7 | rs77659875 | 0.335164 | 0.307458 | 0.305107 | 0.410863 |
| cg15001615 | 1.73E-04 | 19 | rs552196301;rs184493903; rs531588863 | 0.157421 | 0.132996 | 0.131557 | 0.187381 |
| cg23721031 | 1.72E-04 | 14 | rs561046255;rs549167719 | 0.867469 | 0.846305 | 0.863332 | 0.882641 |
| cg20940424 | 1.72E-04 | 7 | rs75166977 | 0.254183 | 0.226688 | 0.222482 | 0.335031 |
| cg10870160 | 1.72E-04 | 17 | rs554308365;rs568341066 | 0.618153 | 0.594519 | 0.606237 | 0.655078 |
| cg07838278 | 1.72E-04 | 5 | rs183035780;rs141352377 | 0.265783 | 0.226128 | 0.230653 | 0.346585 |
| cg19553654 | 1.72E-04 | 3 | rs139091115;rs57488183 | 0.726291 | 0.703271 | 0.719823 | 0.765297 |
| cg17207806 | 1.71E-04 | 7 | rs111333670;rs534466198; rs17135348;rs574712521; rs532083628 | 0.301916 | 0.280143 | 0.28594 | 0.358115 |
| cg21958120 | 1.71E-04 | 6 | rs531377555 | 0.28985 | 0.265124 | 0.264856 | 0.38683 |
| cg02550660 | 1.71E-04 | 13 | rs553885845;rs201875384 | 0.531444 | 0.502857 | 0.516012 | 0.577287 |
| cg01821022 | 1.71E-04 | 12 | rs567377535 | 0.23685 | 0.213904 | 0.200922 | 0.304953 |
| cg23923854 | 1.71E-04 | 11 | rs536840407;rs556440947 | 0.52203 | 0.499043 | 0.494864 | 0.607277 |
| cg18197962 | 1.71E-04 | 2 |  | 0.154093 | 0.128323 | 0.133682 | 0.188179 |
| cg16227731 | 1.71E-04 | 12 | rs375548162;rs576351649 | 0.282184 | 0.251045 | 0.24577 | 0.353427 |
| cg04992345 | 1.70E-04 | 1 | rs535626766 | 0.243628 | 0.210431 | 0.199014 | 0.312433 |
| cg20561536 | 1.69E-04 | 6 |  | 0.26836 | 0.248028 | 0.238369 | 0.324441 |
| cg23338668 | 1.69E-04 | 8 | rs574561578 | 0.347496 | 0.314868 | 0.321626 | 0.450068 |
| cg26314853 | 1.69E-04 | 14 | rs563511755 | 0.487827 | 0.443272 | 0.440612 | 0.558534 |
| cg10408283 | 1.68E-04 | 1 |  | 0.336369 | 0.308581 | 0.295465 | 0.432766 |
| cg04739880 | 1.68E-04 | 6 | rs184214796 | 0.269138 | 0.242385 | 0.25819 | 0.349406 |
| cg26434859 | 1.68E-04 | 6 | rs529659760;rs543037129 | 0.263117 | 0.226862 | 0.213183 | 0.357046 |
| cg14396932 | 1.68E-04 | 11 |  | 0.302471 | 0.272641 | 0.267904 | 0.372511 |
| cg03931397 | 1.68E-04 | 13 |  | 0.281404 | 0.253552 | 0.243278 | 0.355258 |
| cg08557970 | 1.68E-04 | 6 | rs191752532;rs184119176; rs554737449 | 0.319912 | 0.297128 | 0.294101 | 0.395913 |
| cg26416566 | 1.67E-04 | 14 | rs185838383 | 0.288654 | 0.265003 | 0.250494 | 0.370367 |
| cg03568673 | 1.67E-04 | 13 | rs367689408;rs182597970; rs577545882;rs545121504; rs560005043 | 0.322175 | 0.283506 | 0.277694 | 0.398956 |
| cg08322440 | 1.66E-04 | 3 | rs556456470;rs183479076; rs546748398 | 0.283191 | 0.260956 | 0.255841 | 0.35122 |
| cg23670353 | 1.66E-04 | 1 | rs547551697;rs188085102; rs75055449 | 0.240694 | 0.217731 | 0.211002 | 0.319689 |
| cg25084479 | 1.66E-04 | 3 | rs557334809;rs567527358 | 0.345677 | 0.316561 | 0.321501 | 0.456835 |
| cg11203361 | 1.66E-04 | 7 | rs533768063;rs555430815 | 0.325234 | 0.292188 | 0.276225 | 0.413464 |
| cg06674056 | 1.65E-04 | 11 | rs187850520;rs568310083; rs139537044 | 0.557163 | 0.533645 | 0.534326 | 0.619444 |
| cg02097152 | 1.65E-04 | 13 | rs576206165 | 0.3269 | 0.300629 | 0.308361 | 0.402101 |
| cg18854765 | 1.65E-04 | 1 | rs186399715;rs202113178; rs201158336;rs200157650; rs200281954 | 0.253065 | 0.216873 | 0.221467 | 0.322303 |
| cg01141721 | 1.65E-04 | 20 | rs547727197;rs5841263 | 0.345459 | 0.315942 | 0.32626 | 0.420688 |
| cg08287940 | 1.65E-04 | 2 | rs357029 | 0.349766 | 0.313485 | 0.297954 | 0.430466 |
| cg17725378 | 1.64E-04 | 5 |  | 0.452559 | 0.427397 | 0.419559 | 0.524449 |
| cg14156706 | 1.64E-04 | 17 | rs145763670 | 0.414586 | 0.382162 | 0.392075 | 0.510097 |
| cg06947431 | 1.64E-04 | 1 | rs548939574;rs183721805; rs534155143 | 0.311257 | 0.26759 | 0.283141 | 0.415352 |
| cg16506592 | 1.64E-04 | 17 | rs528557827;rs551787061 | 0.30819 | 0.275388 | 0.280129 | 0.386198 |
| cg03511624 | 1.64E-04 | 6 | rs9480901;rs560226509; rs576839652 | 0.420831 | 0.377664 | 0.371393 | 0.515912 |
| cg23689722 | 1.64E-04 | 1 | rs556057380;rs572768852 | 0.369803 | 0.33828 | 0.348939 | 0.430614 |
| cg08553606 | 1.64E-04 | 5 |  | 0.317008 | 0.294931 | 0.28312 | 0.389172 |
| cg15699693 | 1.64E-04 | 5 | rs147304933;rs144309015 | 0.389788 | 0.35362 | 0.344637 | 0.483705 |
| cg06959448 | 1.64E-04 | 1 | rs116496200;rs16841702 | 0.353986 | 0.320398 | 0.321038 | 0.416089 |
| cg13933891 | 1.62E-04 | 3 | rs75135019 | 0.316172 | 0.283203 | 0.277515 | 0.368637 |
| cg09456758 | 1.62E-04 | 14 | rs557304072;rs577163594; rs188554525;rs60175838 | 0.255999 | 0.227141 | 0.223164 | 0.337881 |
| cg14043822 | 1.62E-04 | 2 | rs145046751;rs564379773; rs531643830 | 0.629419 | 0.602502 | 0.609826 | 0.667451 |
| cg01099596 | 1.62E-04 | 2 | rs78003529;rs147371113 | 0.269994 | 0.240078 | 0.22924 | 0.34989 |
| cg21893651 | 1.62E-04 | 6 | rs147784207 | 0.273598 | 0.240292 | 0.230004 | 0.356046 |
| cg25569341 | 1.62E-04 | 2 | rs562191338;rs13395299; rs544301893 | 0.678372 | 0.65203 | 0.658577 | 0.754434 |
| cg25389087 | 1.61E-04 | 18 | rs537256014;rs557621714; rs577515826;rs539801776; rs375687103;rs74378787 | 0.342885 | 0.295804 | 0.303943 | 0.434679 |
| cg16871855 | 1.61E-04 | 8 | rs140683461;rs556979350 | 0.269806 | 0.243044 | 0.237461 | 0.35571 |
| cg10984569 | 1.61E-04 | 10 | rs535514587;rs148759605; rs190779435;rs114658928; rs373419281 | 0.354056 | 0.327644 | 0.310024 | 0.452208 |
| cg21269770 | 1.61E-04 | 2 | rs555866950;rs143635463 | 0.387143 | 0.350287 | 0.360674 | 0.457463 |
| cg00464852 | 1.61E-04 | 14 | rs557798582;rs189929728 | 0.294575 | 0.271388 | 0.273928 | 0.372681 |
| cg26305504 | 1.60E-04 | 19 | rs568653435;rs535730082; rs547732105 | 0.525885 | 0.49549 | 0.47785 | 0.56646 |
| cg11328838 | 1.60E-04 | 3 | rs572983080;rs556232109; rs568216931 | 0.310716 | 0.277397 | 0.278636 | 0.382747 |
| cg19967449 | 1.60E-04 | 3 | rs528142208;rs4362757 | 0.267346 | 0.246055 | 0.233827 | 0.334415 |
| cg07388563 | 1.59E-04 | 2 | rs150614530;rs34416635; rs759458;rs147559268; rs556018903 | 0.225399 | 0.192458 | 0.195658 | 0.269236 |
| cg10925991 | 1.59E-04 | 4 | rs545920942;rs189623344 | 0.249116 | 0.228 | 0.228643 | 0.309626 |
| cg02335176 | 1.58E-04 | 3 |  | 0.290787 | 0.255356 | 0.249857 | 0.365276 |
| cg17496921 | 1.57E-04 | 19 | rs142962581;rs374409; rs12461865;rs150354998 | 0.278571 | 0.250403 | 0.239453 | 0.345303 |
| cg11107451 | 1.57E-04 | 2 | rs554611638;rs141260156; rs185617016 | 0.287902 | 0.256918 | 0.239866 | 0.376681 |
| cg08983526 | 1.57E-04 | 4 | rs576666106 | 0.256457 | 0.219767 | 0.21674 | 0.335241 |
| cg17404534 | 1.56E-04 | 7 | rs143035142;rs55695740; rs190665181 | 0.409293 | 0.382437 | 0.375122 | 0.500834 |
| cg14664412 | 1.56E-04 | 3 | rs552778865;rs75762356 | 0.280677 | 0.253047 | 0.240539 | 0.37271 |
| cg17075758 | 1.56E-04 | X |  | 0.360512 | 0.315956 | 0.310571 | 0.419322 |
| cg05273171 | 1.56E-04 | 2 | rs529111250;rs550543346; rs992158;rs12469881 | 0.334683 | 0.306618 | 0.300843 | 0.378496 |
| cg11933664 | 1.56E-04 | 5 | rs574829748 | 0.309799 | 0.260601 | 0.267354 | 0.394806 |
| cg00211174 | 1.55E-04 | 3 | rs143376511;rs542103978 | 0.312511 | 0.280899 | 0.279222 | 0.399058 |
| cg25965774 | 1.55E-04 | 10 | rs192366394;rs1715861; rs552999833;rs571518022 | 0.178386 | 0.150791 | 0.149623 | 0.213497 |
| cg04179951 | 1.55E-04 | 15 | rs145243820;rs545701621; rs560514246;rs139961303 | 0.363514 | 0.332081 | 0.343069 | 0.46566 |
| cg09218531 | 1.55E-04 | 2 | rs562745183;rs576155207 | 0.357497 | 0.319426 | 0.310978 | 0.441713 |
| cg19245776 | 1.55E-04 | 12 | rs549712512 | 0.398443 | 0.365398 | 0.376669 | 0.4714 |
| cg25246246 | 1.55E-04 | 17 | rs143899460;rs533807573 | 0.382868 | 0.350046 | 0.365333 | 0.456697 |
| cg26344426 | 1.55E-04 | 17 | rs148539492 | 0.411448 | 0.388119 | 0.385713 | NA |
| cg04360434 | 1.55E-04 | 6 | rs528605738;rs190628267; rs457846 | 0.229062 | 0.207802 | 0.199499 | 0.280264 |
| cg06053909 | 1.55E-04 | 5 | rs563142408 | 0.328102 | 0.296206 | 0.303294 | 0.421612 |
| cg13900919 | 1.54E-04 | 20 | rs80069055;rs569744155; rs139742824;rs143035909 | 0.276622 | 0.243668 | 0.243612 | 0.344266 |
| cg15655274 | 1.54E-04 | 2 | rs114264465 | 0.257796 | 0.234698 | 0.22234 | 0.308536 |
| cg13270738 | 1.54E-04 | 6 | rs549396834;rs566172938 | 0.295739 | 0.272256 | 0.261534 | 0.391636 |
| cg05515347 | 1.53E-04 | 18 | rs529848911 | 0.227487 | 0.204238 | 0.197261 | 0.311313 |
| cg03569351 | 1.53E-04 | 1 |  | 0.273803 | 0.252218 | 0.253622 | 0.340301 |
| cg15395733 | 1.53E-04 | 6 | rs187521603;rs549778844 | 0.284846 | 0.262804 | 0.264913 | 0.369606 |
| cg04281750 | 1.53E-04 | 20 | rs544090904;rs568566265; rs59664353;rs547832036 | 0.321415 | 0.279303 | 0.275682 | 0.399386 |
| cg26511108 | 1.53E-04 | 10 | rs569248806;rs143156676; rs151178813 | 0.190401 | 0.160495 | 0.161907 | 0.225983 |
| cg12189187 | 1.52E-04 | 2 | rs151159976;rs116939658; rs569463976 | 0.417625 | 0.395552 | 0.404694 | 0.467114 |
| cg22713444 | 1.52E-04 | 1 | rs190665108 | 0.251786 | 0.226552 | 0.223753 | 0.341293 |
| cg21078344 | 1.52E-04 | 19 | rs553806531;rs572703005 | 0.324468 | 0.301385 | 0.294989 | 0.392727 |
| cg08352329 | 1.52E-04 | 8 | rs576448866;rs150133162; rs138598347;rs572422175 | 0.263189 | 0.235522 | 0.221849 | 0.332852 |
| cg19126615 | 1.52E-04 | 11 | rs151137631;rs139105594; rs149895806 | 0.494718 | 0.473197 | 0.478843 | 0.550548 |
| cg04945422 | 1.51E-04 | 3 | rs561440508;rs73142293; rs538743539;rs190326965 | 0.272552 | 0.249917 | 0.245726 | 0.33889 |
| cg20588986 | 1.51E-04 | 1 | rs551215690;rs370158455; rs576003991 | 0.28258 | 0.244697 | 0.246042 | 0.367363 |
| cg04972459 | 1.51E-04 | 1 | rs541060612;rs559887218; rs548720195 | 0.302551 | 0.279135 | 0.286202 | 0.361041 |
| cg04747180 | 1.51E-04 | 4 | rs184651371 | 0.302492 | 0.268949 | 0.267967 | 0.385423 |
| cg13451703 | 1.51E-04 | 2 | rs536081989;rs186432017; rs62149880 | 0.307903 | 0.275902 | 0.273472 | 0.385405 |
| cg13529789 | 1.51E-04 | 6 | rs3846852;rs192651546; rs11759285 | 0.327048 | 0.284475 | 0.27882 | 0.410406 |
| cg10148702 | 1.50E-04 | 6 | rs563651778;rs111484218; rs146622134;rs571002859; rs189519357 | 0.358428 | 0.32892 | 0.334067 | 0.468965 |
| cg06219576 | 1.50E-04 | 8 | rs145367699;rs149197244; rs10089237;rs143060572; rs147800744;rs185359664; rs113011792 | 0.497278 | 0.477024 | 0.485766 | 0.575323 |
| cg03974400 | 1.50E-04 | 2 | rs141742190 | 0.33907 | 0.299141 | 0.299873 | 0.427036 |
| cg01865231 | 1.50E-04 | 17 | rs73320784;rs552928294; rs572827719;rs76713189 | 0.23752 | 0.215983 | 0.217331 | 0.317592 |
| cg02799411 | 1.50E-04 | 3 | rs188502883;rs191822329; rs75364576;rs144290129; rs556064017 | 0.228397 | 0.198932 | 0.194314 | 0.305164 |
| cg20613972 | 1.49E-04 | 4 | rs79243118;rs573836177; rs117547213 | 0.403143 | 0.354341 | 0.343932 | 0.476124 |
| cg06320712 | 1.49E-04 | 19 | rs75380103 | 0.250521 | 0.225817 | 0.235481 | 0.309984 |
| cg13529355 | 1.49E-04 | 12 | rs542982388 | 0.248019 | 0.220725 | 0.215107 | 0.325588 |
| cg02455719 | 1.49E-04 | 8 | rs547636633;rs148378853 | 0.307551 | 0.28515 | 0.271889 | 0.38334 |
| cg18028160 | 1.49E-04 | 21 | rs558927477 | 0.199476 | 0.172112 | 0.182558 | 0.247163 |
| cg12323063 | 1.49E-04 | 17 | rs377018612;rs548861453 | 0.382295 | 0.362131 | 0.356335 | 0.457241 |
| cg10053235 | 1.49E-04 | 14 | rs187068268;rs190262917; rs149082747 | 0.283873 | 0.242885 | 0.234587 | 0.374218 |
| cg04266202 | 1.49E-04 | 17 | rs182011210;rs150146528; rs531757804 | 0.365662 | 0.332001 | 0.325685 | 0.436252 |
| cg23731826 | 1.48E-04 | 15 | rs148019238 | 0.407885 | 0.386536 | 0.38412 | 0.473194 |
| cg11745838 | 1.48E-04 | 1 | rs181358246;rs573009984 | 0.293973 | 0.263342 | 0.259695 | 0.370384 |
| cg13647878 | 1.48E-04 | 12 |  | 0.302485 | 0.280632 | 0.273346 | 0.384466 |
| cg26197008 | 1.48E-04 | 3 | rs142349123;rs563680079 | 0.29728 | 0.26027 | 0.24812 | 0.385106 |
| cg08594651 | 1.48E-04 | 11 | rs192468283;rs4752831; rs182088708;rs538071712; rs554713896 | 0.394703 | 0.368579 | 0.361195 | 0.486718 |
| cg20782117 | 1.48E-04 | 7 | rs559795997 | 0.426621 | 0.396425 | 0.399367 | 0.484533 |
| cg00808150 | 1.48E-04 | 11 | rs11823739 | 0.230279 | 0.203983 | 0.204703 | 0.312343 |
| cg26066315 | 1.48E-04 | 7 | rs181328057;rs542215332 | 0.331346 | 0.310146 | 0.297551 | 0.38364 |
| cg13176235 | 1.47E-04 | 14 | rs545101840 | 0.284385 | 0.246231 | 0.248409 | 0.359166 |
| cg12668854 | 1.47E-04 | 17 | rs182748216 | 0.299545 | 0.271895 | 0.263765 | 0.366481 |
| cg14872583 | 1.46E-04 | 1 | rs577012239 | 0.556704 | 0.526005 | 0.534208 | 0.630631 |
| cg15719355 | 1.46E-04 | 6 | rs188178384;rs192751415 | 0.427474 | 0.406383 | 0.404633 | 0.494372 |
| cg13338124 | 1.46E-04 | 4 | rs528375351;rs140923107 | 0.298277 | 0.271119 | 0.271898 | 0.404435 |
| cg19205376 | 1.46E-04 | 11 | rs546645672 | 0.216653 | 0.190959 | 0.179496 | 0.294052 |
| cg06151364 | 1.46E-04 | 6 | rs148392334 | 0.243506 | 0.221869 | 0.216874 | 0.301938 |
| cg23778422 | 1.46E-04 | 14 |  | 0.430321 | 0.408895 | 0.419741 | 0.519934 |
| cg16647844 | 1.46E-04 | 1 |  | 0.223572 | 0.193744 | 0.205569 | 0.305476 |
| cg20348647 | 1.45E-04 | 2 | rs552198934;rs571049661 | 0.31587 | 0.283246 | 0.271852 | 0.412472 |
| cg13500720 | 1.45E-04 | 6 | rs548416691;rs538180207 | 0.205409 | 0.182986 | 0.188396 | 0.261828 |
| cg03815962 | 1.45E-04 | 8 | rs116459888;rs375732516; rs145556771; rs183876265 | 0.383045 | 0.352858 | 0.3412 | 0.460667 |
| cg03267730 | 1.45E-04 | 6 | rs180813089 | 0.317458 | 0.292541 | 0.284697 | 0.397128 |
| cg02356039 | 1.45E-04 | 2 | rs183629361 | 0.318269 | 0.294034 | 0.290521 | 0.381333 |
| cg23320029 | 1.45E-04 | 3 | rs570245571;rs74631279 | 0.684314 | 0.65566 | 0.663747 | 0.7129 |
| cg05177498 | 1.45E-04 | 2 | rs143449333 | 0.294856 | 0.267321 | 0.260529 | 0.371874 |
| cg16786178 | 1.44E-04 | 14 | rs371919773 | 0.274647 | 0.237842 | 0.233492 | 0.328519 |
| cg01942558 | 1.44E-04 | 2 | rs542954956;rs76327981; rs186403041;rs144588762 | 0.326397 | 0.279373 | 0.266845 | 0.41264 |
| cg21772289 | 1.44E-04 | 15 | rs528305237 | 0.362914 | 0.331954 | 0.346005 | 0.449767 |
| cg08922737 | 1.43E-04 | 5 | rs548191209;rs550174489; rs533855473 | 0.414705 | 0.385901 | 0.373316 | 0.497243 |
| cg07903073 | 1.43E-04 | 17 |  | 0.321532 | 0.291159 | 0.281754 | 0.410262 |
| cg21328082 | 1.43E-04 | 6 |  | 0.268202 | 0.244934 | 0.232517 | 0.362929 |
| cg26391219 | 1.42E-04 | 11 | rs568571132 | 0.312904 | 0.290373 | 0.288854 | 0.381041 |
| cg08525575 | 1.42E-04 | 12 | rs557932122;rs146934390; rs540481731 | 0.277433 | 0.251903 | 0.245898 | 0.367959 |
| cg10780074 | 1.42E-04 | 3 | rs77415195;rs539852069; rs183401900;rs187645805; rs542342473 | 0.558516 | 0.533596 | 0.553375 | 0.630808 |
| cg16679797 | 1.42E-04 | 9 | rs146718601;rs116486180 | 0.235179 | 0.214429 | 0.227312 | 0.282084 |
| cg10117579 | 1.42E-04 | 2 | rs188569435 | 0.324548 | 0.295021 | 0.286585 | 0.397596 |
| cg18360734 | 1.41E-04 | 17 |  | 0.442324 | 0.418679 | 0.427675 | 0.514547 |
| cg24548817 | 1.41E-04 | 21 | rs113137036;rs572256266 | 0.331666 | 0.296793 | 0.286057 | 0.396749 |
| cg09799980 | 1.41E-04 | 12 | rs150509590;rs577069002; rs374987039;rs3217857 | 0.232847 | 0.209672 | 0.199519 | 0.286013 |
| cg23267277 | 1.41E-04 | 11 | rs183876603;rs189275690 | 0.203312 | 0.179484 | 0.170591 | 0.261344 |
| cg24441357 | 1.41E-04 | 12 | rs187198188;rs79264200; rs539580415 | 0.353647 | 0.32416 | 0.30691 | 0.437358 |
| cg24799894 | 1.40E-04 | 15 | rs192216645 | 0.262294 | 0.234041 | 0.222993 | 0.337656 |
| cg05403383 | 1.40E-04 | 7 | rs141809529;rs59928515; rs529338779 | 0.229522 | 0.208041 | 0.209209 | 0.293743 |
| cg00539399 | 1.40E-04 | 9 | rs572951754;rs539955672 | 0.191727 | 0.163443 | 0.168185 | 0.23924 |
| cg07641926 | 1.40E-04 | 3 | rs200813228;rs375663930; rs140290837;rs184098710; rs143034003 | 0.643059 | 0.620741 | 0.632913 | 0.693584 |
| cg26841048 | 1.40E-04 | 17 |  | 0.430132 | 0.398357 | 0.396003 | 0.493814 |
| cg23854572 | 1.40E-04 | 10 | rs182729967;rs117262906 | 0.313354 | 0.261044 | 0.245218 | NA |
| cg11313708 | 1.39E-04 | 19 | rs144198715;rs3786632; rs570065440 | 0.645734 | 0.616077 | 0.633745 | 0.703252 |
| cg04535525 | 1.39E-04 | 13 | rs11842229 | 0.203377 | 0.180053 | 0.181208 | 0.285484 |
| cg19870584 | 1.39E-04 | 16 | rs113542437;rs562549411; rs76020130 | 0.30935 | 0.281065 | 0.285189 | 0.398361 |
| cg23361127 | 1.39E-04 | 5 | rs16872141 | 0.350832 | 0.317318 | 0.3241 | 0.425387 |
| cg05402432 | 1.38E-04 | 14 |  | 0.242009 | 0.216995 | 0.213907 | 0.305254 |
| cg02052762 | 1.38E-04 | 17 | rs115736596;rs139120581; rs375727184 | 0.302466 | 0.281782 | 0.267518 | 0.388644 |
| cg09635667 | 1.38E-04 | 17 | rs552006133;rs571858644 | 0.322541 | 0.29945 | 0.298591 | 0.375341 |
| cg00349233 | 1.38E-04 | 8 | rs563284194 | 0.362902 | 0.337639 | 0.321723 | 0.457822 |
| cg09620193 | 1.38E-04 | 11 | rs531374560;rs545001111 | 0.394031 | 0.363792 | 0.363677 | 0.468477 |
| cg16447142 | 1.38E-04 | 2 | rs188693576;rs560109103 | 0.294406 | 0.26667 | 0.256971 | 0.357057 |
| cg23136457 | 1.37E-04 | 19 | rs556540105 | 0.349589 | 0.327466 | 0.328099 | 0.39661 |
| cg17571559 | 1.37E-04 | 3 | rs535839226;rs554182133; rs141610893 | 0.305949 | 0.26751 | 0.267373 | 0.366546 |
| cg05783185 | 1.37E-04 | 8 | rs553409924;rs73312352; rs190901871 | 0.200474 | 0.173159 | 0.188132 | 0.29033 |
| cg13399261 | 1.37E-04 | 2 | rs555092180;rs565417715; rs534352523 | 0.231684 | 0.208724 | 0.208513 | 0.282005 |
| cg19309227 | 1.37E-04 | 20 |  | 0.290362 | 0.266834 | 0.250664 | 0.358826 |
| cg14973445 | 1.36E-04 | 12 | rs192361841;rs141591366 | 0.217891 | 0.191922 | 0.198687 | 0.296244 |
| cg10826999 | 1.36E-04 | 1 | rs557313270;rs111562103 | 0.309569 | 0.268103 | 0.257055 | 0.398934 |
| cg09310966 | 1.36E-04 | 6 |  | 0.499302 | 0.475505 | 0.477074 | 0.564223 |
| cg08529481 | 1.36E-04 | 1 | rs564643958;rs188885467; rs547003418 | 0.172361 | 0.142572 | 0.158252 | 0.205703 |
| cg13396713 | 1.36E-04 | 17 | rs10521105;rs373299523; rs150501103;rs373128347; rs377602671;rs201167971 | 0.318306 | 0.287106 | 0.281471 | 0.405751 |
| cg11072837 | 1.34E-04 | 10 | rs551327370;rs571181125 | 0.390738 | 0.363383 | 0.353846 | 0.448181 |
| cg02863947 | 1.34E-04 | 3 | rs182329493 | 0.31206 | 0.280409 | 0.278867 | 0.373909 |
| cg05538887 | 1.34E-04 | 2 | rs577258487;rs567438471; rs187360439 | 0.58644 | 0.559733 | 0.579018 | 0.670795 |
| cg18072664 | 1.34E-04 | 14 | rs74390432 | 0.34388 | 0.307539 | 0.304163 | 0.421168 |
| cg08946713 | 1.33E-04 | 2 | rs549990538;rs374725609; rs41413547 | 0.214642 | 0.190632 | 0.19494 | 0.269565 |
| cg25887955 | 1.33E-04 | 11 | rs117685945 | 0.273238 | 0.245544 | 0.234571 | 0.32968 |
| cg14611767 | 1.33E-04 | 9 | rs370600165;rs373190724 | 0.358026 | 0.328552 | 0.319567 | 0.444892 |
| cg07917901 | 1.33E-04 | 14 | rs187949373;rs547294850; rs190861574 | 0.259285 | 0.231974 | 0.223959 | 0.338958 |
| cg13206941 | 1.33E-04 | 4 | rs568846904;rs140206898; rs564188551;rs142238598 | 0.341108 | 0.318817 | 0.304702 | 0.4315 |
| cg19308563 | 1.33E-04 | 3 | rs571376344;rs189523437 | 0.276443 | 0.253567 | 0.249772 | 0.366107 |
| cg13481904 | 1.32E-04 | 2 |  | 0.421125 | 0.395344 | 0.387274 | 0.507377 |
| cg11016519 | 1.32E-04 | 8 | rs550070520;rs568128166; rs141582192 | 0.449506 | 0.421032 | 0.428002 | 0.527269 |
| cg06766034 | 1.32E-04 | 22 | rs113076938;rs183275015 | 0.290154 | 0.263433 | 0.253815 | 0.375148 |
| cg09047229 | 1.32E-04 | 16 | rs79001082 | 0.297073 | 0.269898 | 0.269033 | 0.361368 |
| cg01948978 | 1.32E-04 | 1 |  | 0.446632 | 0.41783 | 0.417331 | 0.540335 |
| cg17079941 | 1.32E-04 | 15 | rs539405311;rs556904057; rs150006109 | 0.208065 | 0.184505 | 0.18226 | 0.278684 |
| cg24211388 | 1.32E-04 | 6 | rs531742356 | 0.306278 | 0.281529 | 0.266038 | 0.375809 |
| cg14272075 | 1.32E-04 | 17 | rs567586811;rs534338937; rs190961300;rs571020800; rs538718613 | 0.310209 | 0.278617 | 0.2793 | 0.390236 |
| cg19724256 | 1.32E-04 | 12 | rs532910425 | 0.331284 | 0.300798 | 0.300434 | 0.447961 |
| cg19855622 | 1.32E-04 | 14 |  | 0.28 | 0.253408 | 0.248156 | 0.378075 |
| cg18881546 | 1.32E-04 | 12 | rs552513782 | 0.41213 | 0.387246 | 0.38723 | 0.51575 |
| cg04763057 | 1.32E-04 | 5 | rs541128554;rs71617111; rs561005446;rs183584470; rs543498589 | 0.288155 | 0.254512 | 0.249456 | 0.369731 |
| cg20783780 | 1.32E-04 | 11 | rs559320195;rs113536845 | 0.512942 | 0.486187 | 0.504305 | 0.576782 |
| cg08244301 | 1.32E-04 | 19 | rs192463989 | 0.209903 | 0.181941 | 0.186765 | 0.264614 |
| cg14598116 | 1.32E-04 | 1 |  | 0.27449 | 0.25301 | 0.271683 | 0.320176 |
| cg15846307 | 1.31E-04 | 12 | rs375378956 | 0.313877 | 0.283777 | 0.285166 | 0.412537 |
| cg13120357 | 1.31E-04 | 22 | rs116137092 | 0.47882 | 0.454516 | 0.459384 | 0.581053 |
| cg25539555 | 1.31E-04 | 1 | rs12091636;rs113462645 | 0.343243 | 0.313181 | 0.304097 | 0.41487 |
| cg01563236 | 1.31E-04 | 10 | rs373054688 | 0.443506 | 0.408633 | 0.413759 | 0.547675 |
| cg04140624 | 1.31E-04 | 22 | rs150706974;rs533898488; rs61739203 | 0.781256 | 0.760171 | 0.775213 | 0.829122 |
| cg13373048 | 1.30E-04 | 3 | rs187631779 | 0.322563 | 0.293428 | 0.279194 | 0.400637 |
| cg05331340 | 1.30E-04 | 17 | rs559169097;rs575453157 | 0.384225 | 0.36096 | 0.356001 | 0.451403 |
| cg17640043 | 1.30E-04 | 13 |  | 0.163781 | 0.138945 | 0.13062 | 0.200375 |
| cg16152137 | 1.30E-04 | 17 | rs543635461;rs561792639 | 0.324423 | 0.297305 | 0.284259 | 0.430693 |
| cg02874930 | 1.30E-04 | 1 | rs117564359 | 0.231119 | 0.206906 | 0.203241 | 0.316052 |
| cg01867004 | 1.30E-04 | 22 | rs182865807 | 0.301806 | 0.273999 | 0.266611 | 0.368586 |
| cg14806614 | 1.29E-04 | 18 | rs545498776;rs57997738; rs576006832;rs75747086 | 0.333607 | 0.305962 | 0.313879 | 0.418138 |
| cg10323962 | 1.29E-04 | 16 | rs532007635;rs58800453; rs112673036 | 0.244538 | 0.21356 | 0.213274 | 0.312829 |
| cg04730794 | 1.29E-04 | 5 | rs35832136;rs145746510 | 0.341392 | 0.299418 | 0.306924 | 0.429186 |
| cg03958883 | 1.29E-04 | 11 | rs151012726;rs140805031 | 0.469783 | 0.434327 | 0.447197 | 0.516123 |
| cg00602811 | 1.29E-04 | 2 | rs146518085 | 0.396032 | 0.369522 | 0.374475 | 0.461934 |
| cg02796279 | 1.28E-04 | 2 | rs545276748;rs564966030 | 0.283766 | 0.252041 | 0.250416 | NA |
| cg14097859 | 1.28E-04 | 4 | rs574663910;rs535416222 | 0.263588 | 0.238715 | 0.227327 | 0.334608 |
| cg19112290 | 1.28E-04 | 13 | rs184270782;rs9582404 | 0.495538 | 0.467128 | 0.484961 | 0.594047 |
| cg11251063 | 1.28E-04 | 6 | rs113488667;rs371908209; rs9378479 | 0.37914 | 0.354923 | 0.348697 | 0.436933 |
| cg07920894 | 1.27E-04 | 5 | rs146203208;rs268728; rs193015695 | 0.388189 | 0.353923 | 0.371063 | 0.469533 |
| cg04195852 | 1.27E-04 | 1 | rs572304803;rs539688506 | 0.339893 | 0.312896 | 0.296162 | 0.413114 |
| cg04784315 | 1.27E-04 | 9 | rs141576954;rs144184058; rs151065550;rs140964923; rs150917003 | 0.331108 | 0.310919 | 0.309832 | 0.406717 |
| cg13905385 | 1.27E-04 | 17 |  | 0.286367 | 0.25757 | 0.248733 | 0.37229 |
| cg01450842 | 1.26E-04 | 2 | rs527835694 | 0.839154 | 0.813667 | 0.819789 | 0.868745 |
| cg16061354 | 1.26E-04 | 17 | rs529757240;rs554001669 | 0.264926 | 0.232528 | 0.230677 | 0.350668 |
| cg06100973 | 1.26E-04 | 19 | rs544272607;rs556244475; rs574938033;rs137896468 | 0.291049 | 0.263859 | 0.252051 | 0.367692 |
| cg19468440 | 1.26E-04 | 20 | rs115569429;rs141161016; rs180902017;rs147368753 | 0.296396 | 0.273543 | 0.267257 | 0.404266 |
| cg20199626 | 1.26E-04 | 1 | rs11289842 | 0.351612 | 0.329578 | 0.31812 | 0.400145 |
| cg12488187 | 1.26E-04 | 12 |  | 0.346288 | 0.308055 | 0.311193 | 0.439545 |
| cg10997258 | 1.26E-04 | 2 | rs114681115 | 0.346742 | 0.305775 | 0.305314 | 0.439563 |
| cg01351315 | 1.26E-04 | 17 | rs150257107;rs114686333; rs574096279 | 0.303794 | 0.265336 | 0.267354 | 0.359886 |
| cg07739927 | 1.26E-04 | 17 | rs573109638 | 0.385332 | 0.355823 | 0.36681 | 0.440897 |
| cg15505203 | 1.25E-04 | 12 | rs73217771;rs577450633 | 0.20104 | 0.17887 | 0.182888 | 0.25757 |
| cg25405284 | 1.25E-04 | 5 |  | 0.519708 | 0.484095 | 0.488114 | 0.57636 |
| cg12667294 | 1.25E-04 | 2 | rs551154078;rs571060213 | 0.290062 | 0.268369 | 0.257911 | 0.367354 |
| cg17657253 | 1.25E-04 | 1 | rs138554763;rs544838061; rs192419169 | 0.285488 | 0.254841 | 0.249393 | 0.368807 |
| cg22733946 | 1.24E-04 | 5 | rs557958804 | 0.311631 | 0.287372 | 0.275237 | 0.369788 |
| cg25199876 | 1.24E-04 | 22 | rs201378550;rs562588594; rs567481163 | 0.315194 | 0.287248 | 0.27967 | 0.372053 |
| cg17064161 | 1.24E-04 | 2 |  | 0.657942 | 0.627586 | 0.630225 | 0.694962 |
| cg21097733 | 1.24E-04 | 17 | rs559912377;rs543109125 | 0.506565 | 0.483499 | 0.484831 | 0.553309 |
| cg23283063 | 1.24E-04 | 12 | rs113296741 | 0.29834 | 0.271302 | 0.261028 | 0.375174 |
| cg18708700 | 1.24E-04 | 19 | rs116017355;rs140017075; rs537169353 | 0.508722 | 0.485484 | 0.499555 | 0.57136 |
| cg05829961 | 1.24E-04 | 9 | rs536820617 | 0.356925 | 0.326411 | 0.329086 | 0.420274 |
| cg21058963 | 1.23E-04 | 6 | rs561794083;rs184515246 | 0.163111 | 0.138678 | 0.147738 | 0.217766 |
| cg18702935 | 1.23E-04 | 13 | rs192757422 | 0.533831 | 0.484064 | 0.494671 | 0.593942 |
| cg11826104 | 1.23E-04 | 15 | rs7165149;rs569814360; rs151186013 | 0.423544 | 0.391259 | 0.392724 | 0.496669 |
| cg10914865 | 1.23E-04 | 17 | rs570583744;rs537795095; rs556204488;rs574390373; rs78666479 | 0.31952 | 0.296576 | 0.291176 | 0.369741 |
| cg08809635 | 1.23E-04 | 15 | rs544309769;rs561031456 | 0.34232 | 0.308243 | 0.307397 | 0.43061 |
| cg04756491 | 1.23E-04 | 14 |  | 0.272233 | 0.241454 | 0.243511 | 0.354346 |
| cg22133576 | 1.23E-04 | 4 | rs150443035;rs138283237; rs189427176;rs567178081; rs534111510 | 0.683656 | 0.651587 | 0.64734 | 0.718392 |
| cg01216276 | 1.22E-04 | 4 | rs534396781;rs76521816 | 0.298312 | 0.258104 | 0.271672 | 0.405483 |
| cg11900393 | 1.22E-04 | 12 | rs372699873;rs573478495 | 0.442756 | 0.414562 | 0.429545 | 0.515716 |
| cg09535443 | 1.21E-04 | 11 |  | 0.310639 | 0.289165 | 0.278563 | 0.391232 |
| cg09655571 | 1.21E-04 | 16 | rs192007564;rs574633688 | 0.568009 | 0.547421 | 0.545807 | 0.623746 |
| cg16314013 | 1.20E-04 | 5 |  | 0.323462 | 0.299831 | 0.293709 | 0.409062 |
| cg05271255 | 1.20E-04 | 17 |  | 0.163052 | 0.138554 | 0.136705 | 0.206832 |
| cg02990302 | 1.20E-04 | 16 |  | 0.252259 | 0.232075 | 0.220017 | 0.325782 |
| cg01787084 | 1.20E-04 | 16 | rs115260618;rs529292453; rs35759961;rs559911610 | 0.21014 | 0.179411 | 0.196799 | 0.282621 |
| cg14799927 | 1.20E-04 | 6 | rs539258932 | 0.363752 | 0.318008 | 0.333367 | 0.425685 |
| cg06458106 | 1.19E-04 | 22 | rs75870648 | 0.573557 | 0.54398 | 0.545937 | 0.614279 |
| cg19987129 | 1.19E-04 | 6 | rs151321391;rs6925308; rs182024837;rs72972917 | 0.301722 | 0.273408 | 0.257668 | 0.383854 |
| cg19749984 | 1.19E-04 | 21 |  | 0.49176 | 0.467557 | 0.465016 | 0.537407 |
| cg04768892 | 1.19E-04 | 5 | rs554718737;rs372044355 | 0.279494 | 0.250705 | 0.25033 | 0.354684 |
| cg07241090 | 1.19E-04 | 12 | rs570189274;rs537248908 | 0.295441 | 0.268961 | 0.255806 | 0.348684 |
| cg00135546 | 1.19E-04 | 21 | rs148548430;rs144306802; rs375581925;rs17766637 | 0.197726 | 0.176952 | 0.179546 | 0.275102 |
| cg23097039 | 1.19E-04 | 8 | rs562742703;rs531549138; rs551558792 | 0.316492 | 0.278923 | 0.281979 | 0.377316 |
| cg16762030 | 1.19E-04 | 15 | rs78895723 | 0.275905 | 0.251515 | 0.232333 | 0.369995 |
| cg15961811 | 1.19E-04 | 14 | rs536206582;rs547881898; rs567153776;rs75892139; rs142363302 | 0.310129 | 0.286731 | 0.268113 | 0.381278 |
| cg18455737 | 1.19E-04 | 4 | rs146464213;rs566993006; rs187759454 | 0.34493 | 0.301884 | 0.296815 | 0.428507 |
| cg05920921 | 1.18E-04 | 6 | rs561308996 | 0.290472 | 0.26259 | 0.261353 | 0.385203 |
| cg22938535 | 1.18E-04 | 4 | rs76668327 | 0.136505 | 0.115408 | 0.116638 | 0.172847 |
| cg07638261 | 1.18E-04 | 4 | rs567749437;rs183380291 | 0.325219 | 0.274843 | 0.271292 | 0.417413 |
| cg24102964 | 1.18E-04 | 20 |  | 0.317057 | 0.293677 | 0.288271 | 0.386961 |
| cg17800229 | 1.18E-04 | 1 | rs528659399;rs192048791; rs562212302 | 0.177401 | 0.155648 | 0.161376 | 0.214324 |
| cg03720119 | 1.18E-04 | 17 | rs144330164;rs200070541; rs147785768 | 0.567524 | 0.546108 | 0.562808 | 0.627464 |
| cg17426520 | 1.17E-04 | 1 | rs139232061;rs112188334 | 0.498305 | 0.475859 | 0.487267 | 0.571581 |
| cg19064200 | 1.17E-04 | 8 | rs377698492;rs535782048; rs144297908 | 0.289577 | 0.264657 | 0.26256 | 0.370224 |
| cg00622815 | 1.17E-04 | 20 | rs541321276 | 0.315115 | 0.271016 | 0.267748 | 0.413404 |
| cg10380094 | 1.16E-04 | 1 | rs148830015 | 0.244932 | 0.222166 | 0.210442 | 0.317836 |
| cg24941342 | 1.16E-04 | 11 |  | 0.265538 | 0.233432 | 0.22554 | 0.331046 |
| cg14044685 | 1.16E-04 | 11 | rs187634354;rs190432598; rs566599436;rs138945007 | 0.313575 | 0.272298 | 0.281789 | 0.411428 |
| cg19689587 | 1.16E-04 | 17 | rs78005919;rs545190347; rs4969069 | 0.257148 | 0.233261 | 0.224406 | 0.336463 |
| cg01986936 | 1.16E-04 | 5 | rs79935815;rs564900988; rs572218267 | 0.295639 | 0.266203 | 0.261717 | 0.363947 |
| cg24522361 | 1.16E-04 | 2 | rs544387563;rs191876569 | 0.324403 | 0.30218 | 0.298429 | 0.39711 |
| cg09416908 | 1.16E-04 | 11 | rs544714243 | 0.275198 | 0.242742 | 0.230874 | 0.347991 |
| cg09802818 | 1.15E-04 | 12 | rs144019949;rs112895480; rs142886037 | 0.300466 | 0.270965 | 0.257286 | 0.375853 |
| cg16815249 | 1.14E-04 | 6 | rs544265525;rs186474903 | 0.283719 | 0.253491 | 0.23509 | 0.364521 |
| cg07377519 | 1.14E-04 | 19 | rs58026265;rs137986638 | 0.239924 | 0.20731 | 0.210919 | 0.308507 |
| cg21834738 | 1.13E-04 | 5 | rs186205240 | 0.303835 | 0.263183 | 0.253053 | 0.374129 |
| cg12823039 | 1.13E-04 | 19 | rs192089705;rs529200462 | 0.464494 | 0.444282 | 0.462775 | 0.546787 |
| cg07006003 | 1.13E-04 | 19 | rs573054305 | 0.308274 | 0.275429 | 0.271133 | 0.385219 |
| cg17419815 | 1.12E-04 | 12 | rs61741740 | 0.357553 | 0.315786 | 0.313213 | 0.450765 |
| cg12592359 | 1.12E-04 | 20 | rs367588832 | 0.417748 | 0.387307 | 0.390949 | 0.483803 |
| cg09324566 | 1.12E-04 | 1 | rs550072643;rs569517830 | 0.388856 | 0.359267 | 0.355619 | 0.428969 |
| cg15137408 | 1.12E-04 | 15 | rs149999161 | 0.249648 | 0.222334 | 0.214462 | 0.324962 |
| cg05727422 | 1.12E-04 | 14 | rs528681216 | 0.302633 | 0.282536 | 0.275019 | NA |
| cg00789535 | 1.11E-04 | 5 | rs187474320;rs554120497; rs192419956;rs142135098; rs564030582;rs532550239 | 0.284309 | 0.244167 | 0.237849 | 0.371413 |
| cg01425393 | 1.11E-04 | 12 | rs577421595;rs546130103 | 0.228581 | 0.20701 | 0.199549 | 0.311892 |
| cg05644602 | 1.11E-04 | 2 | rs568431477;rs148164365; rs557013486 | 0.438911 | 0.417442 | 0.412281 | 0.510203 |
| cg07422843 | 1.11E-04 | 17 |  | 0.308477 | 0.279924 | 0.279867 | 0.393079 |
| cg24849637 | 1.10E-04 | 5 | rs532995437;rs79452598; rs562690291 | 0.37153 | 0.332758 | 0.339467 | 0.454263 |
| cg09068998 | 1.10E-04 | 5 | rs114464628;rs566154272; rs6580598;rs558135173 | 0.226562 | 0.202378 | 0.200473 | 0.311785 |
| cg25409929 | 1.10E-04 | 10 | rs545942171;rs564228454 | 0.310417 | 0.273051 | 0.268761 | 0.382927 |
| cg19189348 | 1.09E-04 | 10 | rs10884973;rs184152660; rs562639031 | 0.313243 | 0.280963 | 0.278153 | 0.387422 |
| cg18767115 | 1.09E-04 | 22 | rs138508749;rs562801884; rs576627434;rs542019465; rs561845378;rs191712136; rs547710973 | 0.385231 | 0.357187 | 0.36093 | 0.463632 |
| cg09664107 | 1.09E-04 | 12 | rs146770533 | 0.165722 | 0.142703 | 0.14403 | 0.208718 |
| cg06687184 | 1.09E-04 | 5 | rs191248431;rs139867473 | 0.329776 | 0.303642 | 0.294946 | 0.397905 |
| cg20337822 | 1.09E-04 | 5 | rs577574658 | 0.329886 | 0.300377 | 0.300662 | 0.418591 |
| cg18990915 | 1.08E-04 | 16 | rs567201255;rs574558570 | 0.352176 | 0.323926 | 0.335697 | 0.420527 |
| cg14742445 | 1.08E-04 | 22 | rs539209546 | 0.294692 | 0.263319 | 0.256909 | 0.357806 |
| cg15762892 | 1.08E-04 | 2 | rs540126093;rs74347024 | 0.285719 | 0.260111 | 0.259658 | 0.35583 |
| cg06644892 | 1.08E-04 | 3 | rs549160728;rs561048309; rs531652293 | 0.559536 | 0.533766 | 0.519456 | 0.631245 |
| cg03115308 | 1.07E-04 | 15 | rs538960368;rs562248109; rs529457482;rs544710282; rs562882136 | 0.347523 | 0.315535 | 0.300682 | 0.420125 |
| cg09470754 | 1.07E-04 | 19 | rs115606959 | 0.292245 | 0.266454 | 0.264936 | 0.353227 |
| cg17069928 | 1.07E-04 | 3 |  | 0.325376 | 0.283147 | 0.284834 | 0.417682 |
| cg18067134 | 1.07E-04 | 3 |  | 0.376112 | 0.347366 | 0.342425 | 0.451464 |
| cg21093326 | 1.07E-04 | 2 | rs192822888 | 0.201681 | 0.173617 | 0.165817 | 0.246087 |
| cg09284275 | 1.06E-04 | 16 | rs553269333 | 0.304923 | 0.270424 | 0.266599 | 0.372312 |
| cg21814550 | 1.06E-04 | 14 | rs566149646 | 0.302282 | 0.263778 | 0.255688 | 0.410923 |
| cg07484151 | 1.06E-04 | 11 | rs112190291 | 0.283559 | 0.25299 | 0.245594 | 0.370957 |
| cg21508192 | 1.06E-04 | 12 |  | 0.241007 | 0.202296 | 0.200061 | 0.326613 |
| cg13486441 | 1.06E-04 | 7 |  | 0.204098 | 0.182125 | 0.169153 | 0.267807 |
| cg06502279 | 1.05E-04 | 16 | rs149153937 | 0.332631 | 0.291735 | 0.296453 | 0.411234 |
| cg24165638 | 1.05E-04 | 19 | rs577410344 | 0.38763 | 0.353798 | 0.363191 | 0.457144 |
| cg23139521 | 1.05E-04 | 10 | rs537803625 | 0.236174 | 0.21176 | 0.210333 | 0.308766 |
| cg06680852 | 1.05E-04 | 10 |  | 0.340293 | 0.316605 | 0.329502 | 0.414121 |
| cg14512523 | 1.05E-04 | 11 | rs551899681 | 0.218805 | 0.189702 | 0.194803 | 0.282114 |
| cg10511346 | 1.05E-04 | 11 | rs553345434 | 0.563536 | 0.53696 | 0.54635 | 0.631853 |
| cg13277464 | 1.05E-04 | 2 | rs140189160;rs186214816; rs573447121; rs545691693 | 0.590131 | 0.569576 | 0.58245 | 0.640249 |
| cg08137827 | 1.05E-04 | 22 |  | 0.360057 | 0.338205 | 0.321481 | 0.447458 |
| cg24742525 | 1.05E-04 | 7 | rs62479794;rs531414735 | 0.320267 | 0.275 | 0.261807 | 0.390872 |
| cg16125725 | 1.05E-04 | 15 | rs116374074 | 0.319952 | 0.287334 | 0.270366 | 0.414391 |
| cg18798412 | 1.04E-04 | 6 | rs535846248;rs548282764 | 0.345157 | 0.308313 | 0.302395 | 0.435477 |
| cg06939451 | 1.04E-04 | 5 | rs556232289;rs116754553 | 0.331315 | 0.306981 | 0.32454 | 0.427288 |
| cg24154948 | 1.04E-04 | 12 | rs140380182;rs568644002 | 0.383884 | 0.362552 | 0.358045 | 0.438127 |
| cg21179618 | 1.04E-04 | 9 | rs560311569;rs575103819; rs552226550 | 0.321282 | 0.297087 | 0.306689 | 0.376282 |
| cg21609524 | 1.04E-04 | 14 |  | 0.27842 | 0.246011 | 0.247922 | 0.362324 |
| cg05132705 | 1.04E-04 | 1 | rs547010972;rs570079776 | 0.192127 | 0.158494 | 0.170865 | 0.235202 |
| cg26551026 | 1.04E-04 | 18 | rs538176432;rs7226820; rs577212966 | 0.379204 | 0.347606 | 0.350126 | 0.451444 |
| cg14723713 | 1.04E-04 | 6 |  | 0.293355 | 0.249091 | 0.243421 | 0.380481 |
| cg14645898 | 1.03E-04 | 20 | rs540247960 | 0.148428 | 0.126302 | 0.11949 | 0.188773 |
| cg08438525 | 1.03E-04 | 7 | rs192244593;rs149249854 | 0.527255 | 0.48484 | 0.495962 | 0.588025 |
| cg08803535 | 1.03E-04 | 12 | rs566770568 | 0.539704 | 0.517846 | 0.523816 | 0.604412 |
| cg16002523 | 1.03E-04 | 3 | rs547033138;rs565668645 | 0.214292 | 0.192176 | 0.183886 | 0.259161 |
| cg20474199 | 1.03E-04 | 21 | rs150358629;rs550068779 | 0.441223 | 0.403753 | 0.412424 | 0.479444 |
| cg10020520 | 1.02E-04 | 16 | rs549559281 | 0.377792 | 0.357575 | 0.345945 | 0.429824 |
| cg24163194 | 1.02E-04 | 7 | rs549381846;rs562898116; rs530823606 | 0.329393 | 0.299124 | 0.30034 | 0.425408 |
| cg20010135 | 1.02E-04 | 16 | rs546794537;rs12443808; rs12443627 | 0.604896 | 0.578117 | 0.584565 | 0.658138 |
| cg03948781 | 1.01E-04 | 1 | rs576635212;rs140659214 | 0.197792 | 0.17462 | 0.169244 | 0.266565 |
| cg01087254 | 1.01E-04 | 15 | rs138792376 | 0.2725 | 0.237805 | 0.228935 | 0.333514 |
| cg24405567 | 1.01E-04 | 15 | rs147375720 | 0.287549 | 0.26629 | 0.251597 | 0.363943 |
| cg04959747 | 1.01E-04 | 3 | rs558867284;rs570708462 | 0.232632 | 0.20933 | 0.211396 | 0.302762 |
| cg16652463 | 1.01E-04 | 14 | rs188824213;rs181251970; rs549102633 | 0.27714 | 0.243994 | 0.246575 | 0.369969 |
| cg02080811 | 1.01E-04 | 22 | rs372333047;rs375763634; rs201115873;rs111517609; rs551308146 | 0.482299 | 0.457518 | 0.464584 | 0.558048 |
| cg14768260 | 1.01E-04 | 8 | rs188760258 | 0.265902 | 0.243728 | 0.232229 | 0.338058 |
| cg14293747 | 1.01E-04 | 3 |  | 0.539268 | 0.513601 | 0.525349 | 0.606942 |
| cg18634760 | 1.00E-04 | 13 | rs17844150;rs191719850; rs17844149;rs183179288 | 0.336221 | 0.307165 | 0.30748 | 0.43834 |
| cg26532550 | 1.00E-04 | 7 | rs184560412;rs74855904 | 0.295675 | 0.272244 | 0.257478 | 0.362348 |
| cg20292908 | 1.00E-04 | 5 | rs558544310;rs112504939 | 0.615798 | 0.590684 | 0.595497 | 0.67312 |
| cg21932286 | 1.00E-04 | 3 | rs186602397;rs566273370; rs111297430;rs144933326; rs55792220;rs181872252; rs542103868;rs571661769 | 0.412851 | 0.385079 | 0.379234 | 0.459076 |
| cg09492975 | 1.00E-04 | 3 | rs535839226;rs554182133; rs141610893 | 0.262707 | 0.228032 | 0.219919 | 0.317056 |
| cg05928849 | 9.95E-05 | 11 | rs189403726 | 0.333132 | 0.292918 | 0.299266 | 0.419728 |
| cg05813673 | 9.93E-05 | 4 | rs186637051;rs77359243; rs537786165;rs554400659 | 0.5516 | 0.526862 | 0.520383 | 0.623708 |
| cg10440877 | 9.93E-05 | 2 | rs569272357 | 0.426455 | 0.401898 | 0.399021 | 0.490531 |
| cg12026822 | 9.85E-05 | 18 | rs182460260;rs535767170; rs146802437 | 0.24075 | 0.212599 | 0.19471 | 0.32021 |
| cg05941378 | 9.85E-05 | 15 | rs142543311 | 0.271842 | 0.246512 | 0.232462 | 0.365268 |
| cg00891657 | 9.82E-05 | 18 | rs566090518;rs528871515; rs149574607 | 0.27784 | 0.247789 | 0.241803 | 0.381379 |
| cg07925925 | 9.81E-05 | 5 |  | 0.358537 | 0.32092 | 0.317772 | 0.430337 |
| cg00191409 | 9.78E-05 | 12 | rs184163442 | 0.273351 | 0.234492 | 0.22848 | 0.350515 |
| cg26503179 | 9.76E-05 | 11 |  | 0.268018 | 0.230409 | 0.225001 | 0.354911 |
| cg07504763 | 9.74E-05 | 1 |  | 0.408762 | 0.382305 | 0.381456 | 0.461376 |
| cg18544696 | 9.74E-05 | 11 |  | 0.201596 | 0.181074 | 0.180936 | 0.272986 |
| cg23002660 | 9.73E-05 | 2 |  | 0.336965 | 0.302582 | 0.295307 | 0.415483 |
| cg11543559 | 9.73E-05 | 2 | rs189426493;rs3771202 | 0.324441 | 0.279645 | 0.266998 | 0.396903 |
| cg17964466 | 9.72E-05 | 11 | rs531893321;rs544138667 | 0.22567 | 0.201756 | 0.205389 | 0.272911 |
| cg22941573 | 9.69E-05 | 20 |  | 0.347121 | 0.313467 | 0.309556 | 0.430771 |
| cg12049550 | 9.65E-05 | 1 | rs188416861;rs544447560; rs180772624;rs145308835; rs552049113 | 0.292143 | 0.267189 | 0.263568 | 0.373982 |
| cg09969502 | 9.64E-05 | 10 | rs541674994 | 0.336899 | 0.310648 | 0.309211 | 0.400913 |
| cg12031641 | 9.64E-05 | 1 | rs527569986;rs146780455; rs564277234 | 0.403017 | 0.379007 | 0.381146 | 0.480583 |
| cg25071648 | 9.64E-05 | 16 |  | 0.309317 | 0.286313 | 0.283553 | 0.39661 |
| cg13474450 | 9.63E-05 | 8 | rs558892917;rs528274678; rs541739901;rs560132156 | 0.419914 | 0.383277 | 0.382001 | 0.49463 |
| cg04170334 | 9.60E-05 | 2 | rs559945043;rs139746448; rs142633276;rs192808116 | 0.28842 | 0.241553 | 0.236378 | 0.368456 |
| cg20338956 | 9.59E-05 | 13 | rs148944981 | 0.285509 | 0.251473 | 0.244402 | 0.366678 |
| cg21892720 | 9.58E-05 | 19 | rs143530097;rs112647644; rs542033606 | 0.295557 | 0.263599 | 0.270383 | 0.379304 |
| cg08425760 | 9.58E-05 | 1 | rs182671352;rs557554330 | 0.320764 | 0.296617 | 0.289128 | 0.386544 |
| cg20147755 | 9.56E-05 | 5 | rs144249233;rs115847254; rs567996121 | 0.29037 | 0.257492 | 0.255228 | 0.361352 |
| cg17133173 | 9.51E-05 | 14 | rs189467671;rs78357955; rs570088278 | 0.266667 | 0.244507 | 0.230815 | 0.335029 |
| cg20109867 | 9.50E-05 | 6 | rs138212381 | 0.300257 | 0.276267 | 0.270868 | 0.394224 |
| cg18326313 | 9.49E-05 | 13 |  | 0.432739 | 0.402296 | 0.399956 | 0.520102 |
| cg05465821 | 9.46E-05 | 20 | rs151212796;rs531638633 | 0.258123 | 0.232888 | 0.220993 | 0.314259 |
| cg06698971 | 9.42E-05 | 2 | rs115107642;rs2118305; rs192833017 | 0.323956 | 0.300086 | 0.300866 | 0.423359 |
| cg03253150 | 9.41E-05 | 9 | rs186267665;rs564712905; rs533532254 | 0.360747 | 0.321011 | 0.319073 | 0.427992 |
| cg19368946 | 9.39E-05 | 11 | rs527828198 | 0.385434 | 0.354475 | 0.35921 | 0.446155 |
| cg09654536 | 9.39E-05 | 4 |  | 0.432915 | 0.40287 | 0.398518 | 0.5392 |
| cg18888137 | 9.38E-05 | 6 | rs2066740;rs540735041 | 0.291121 | 0.268651 | 0.26072 | 0.372642 |
| cg18935575 | 9.33E-05 | 7 |  | 0.228039 | 0.198925 | 0.192142 | 0.292224 |
| cg05165907 | 9.31E-05 | 6 |  | 0.287032 | 0.260869 | 0.256074 | 0.34794 |
| cg16743940 | 9.28E-05 | 9 | rs146209359 | 0.247852 | 0.219646 | 0.204301 | 0.307756 |
| cg06661904 | 9.28E-05 | 12 | rs562066918;rs143184401 | 0.290866 | 0.270509 | 0.256605 | 0.365851 |
| cg24422977 | 9.28E-05 | 15 | rs537612956 | 0.144931 | 0.123584 | 0.132994 | 0.197813 |
| cg03626208 | 9.27E-05 | 12 | rs534038349 | 0.280544 | 0.260166 | 0.260848 | 0.368885 |
| cg12489775 | 9.27E-05 | 22 | rs568652871;rs5759190; rs117794580;rs547698608 | 0.224521 | 0.203724 | 0.198078 | 0.285947 |
| cg10269619 | 9.26E-05 | 5 | rs564521735;rs77829878 | 0.327058 | 0.303732 | 0.314396 | 0.418579 |
| cg01056126 | 9.25E-05 | 18 | rs529415718;rs549204503; rs9944755 | 0.307648 | 0.278774 | 0.268761 | 0.38262 |
| cg20263799 | 9.24E-05 | 17 | rs181366161 | 0.265643 | 0.238572 | 0.238651 | 0.310572 |
| cg19726712 | 9.22E-05 | 8 | rs536065013;rs576477659 | 0.508418 | 0.482125 | 0.482452 | 0.555309 |
| cg19384042 | 9.22E-05 | 5 | rs376843507;rs149325469 | 0.41421 | 0.39314 | 0.381802 | 0.498774 |
| cg15784590 | 9.22E-05 | 3 | rs528291673;rs546425011; rs565166468;rs76272584; rs373548156 | 0.342168 | 0.317424 | 0.321077 | 0.438246 |
| cg01048372 | 9.22E-05 | 8 | rs575919460;rs188760258 | 0.355772 | 0.332669 | 0.332026 | 0.443111 |
| cg12401918 | 9.18E-05 | 6 | rs374317137 | 0.196089 | 0.16864 | 0.171949 | 0.239779 |
| cg02497258 | 9.17E-05 | 7 | rs571863244 | 0.341487 | 0.309194 | 0.306302 | 0.397456 |
| cg24794625 | 9.16E-05 | 15 |  | 0.309039 | 0.275379 | 0.266358 | 0.376088 |
| cg11167160 | 9.15E-05 | 16 | rs566528480;rs533897541 | 0.377524 | 0.349735 | 0.339205 | 0.483797 |
| cg03964681 | 9.07E-05 | 13 | rs574201922;rs186697362 | 0.293498 | 0.253629 | 0.247056 | 0.392541 |
| cg06204101 | 9.06E-05 | 1 | rs548420924 | 0.655261 | 0.631206 | 0.644697 | 0.700195 |
| cg05913121 | 9.04E-05 | 10 | rs546507523;rs151305364 | 0.310494 | 0.288755 | 0.281952 | 0.392469 |
| cg14330209 | 9.03E-05 | 12 | rs184606520;rs563399251 | 0.275908 | 0.247767 | 0.246689 | 0.350116 |
| cg05303777 | 9.00E-05 | 3 | rs547879019;rs150706652 | 0.325359 | 0.296115 | 0.288089 | 0.400109 |
| cg18112953 | 8.97E-05 | 20 | rs545657605;rs73260268; rs146534938;rs368035631 | 0.310652 | 0.284743 | 0.285902 | 0.389362 |
| cg07168526 | 8.97E-05 | 7 | rs543816342 | 0.317323 | 0.28614 | 0.283624 | 0.391983 |
| cg17563088 | 8.95E-05 | 8 | rs549982360;rs113029886 | 0.615768 | 0.58872 | 0.594618 | 0.679006 |
| cg10272461 | 8.92E-05 | 7 | rs142772798;rs189550980 | 0.299357 | 0.258547 | 0.257337 | 0.371516 |
| cg17812664 | 8.91E-05 | 10 | rs538952203 | 0.388499 | 0.361644 | 0.372977 | 0.478717 |
| cg26446161 | 8.88E-05 | 6 | rs570159608;rs35767545; rs532646045;rs377165593 | 0.448439 | 0.426606 | 0.422609 | 0.514712 |
| cg00524291 | 8.88E-05 | 7 | rs556423601;rs574874816; rs574283082 | 0.370715 | 0.332791 | 0.335559 | 0.457942 |
| cg20701457 | 8.87E-05 | 17 | rs570875066;rs539541378; rs550004446 | 0.487122 | 0.460128 | 0.467364 | 0.538526 |
| cg16230352 | 8.85E-05 | 8 | rs542650705;rs192678237; rs531827960 | 0.303468 | 0.280749 | 0.283093 | 0.368544 |
| cg14136168 | 8.83E-05 | 9 |  | 0.188741 | 0.165867 | 0.16463 | 0.235762 |
| cg21470541 | 8.81E-05 | 2 | rs530165158;rs368461460; rs566971952 | 0.528888 | 0.497871 | 0.49647 | 0.611787 |
| cg05676915 | 8.78E-05 | 16 | rs545844647;rs564282774; rs573087010 | 0.289703 | 0.255646 | 0.252425 | 0.35385 |
| cg05161346 | 8.72E-05 | 11 | rs199907307 | 0.280485 | 0.253884 | 0.236284 | 0.359643 |
| cg04609694 | 8.72E-05 | 6 | rs554084505;rs572256406 | 0.30134 | 0.278839 | 0.271486 | 0.399009 |
| cg09166556 | 8.71E-05 | 1 | rs7411312 | 0.408565 | 0.36597 | 0.359893 | 0.516058 |
| cg17973115 | 8.70E-05 | 1 | rs12058996;rs544716752; rs11249236 | 0.220038 | 0.195793 | 0.196699 | 0.287505 |
| cg25077579 | 8.68E-05 | 19 | rs565881409;rs7251245 | 0.189369 | 0.163698 | 0.159921 | 0.249295 |
| cg08925954 | 8.68E-05 | 19 | rs11461038;rs575440857; rs541663763 | 0.291475 | 0.261885 | 0.251594 | 0.363423 |
| cg09128944 | 8.67E-05 | 2 | rs182591329;rs12474309 | 0.279729 | 0.243935 | 0.245115 | 0.335346 |
| cg14209922 | 8.66E-05 | 2 | rs553914964;rs34097579 | 0.270756 | 0.243201 | 0.223818 | 0.342106 |
| cg04204465 | 8.66E-05 | 9 | rs539757445;rs148184169 | 0.318112 | 0.292139 | 0.284643 | 0.417334 |
| cg02432022 | 8.65E-05 | 6 | rs148971434;rs66467133 | 0.291724 | 0.245273 | 0.241563 | 0.374932 |
| cg24532893 | 8.63E-05 | 10 | rs559515610 | 0.264523 | 0.232808 | 0.232267 | 0.324325 |
| cg15612947 | 8.63E-05 | 5 | rs553125250 | 0.298978 | 0.274966 | 0.266044 | 0.362223 |
| cg13618516 | 8.63E-05 | 17 |  | 0.349599 | 0.310695 | 0.307685 | 0.433651 |
| cg23031748 | 8.58E-05 | 14 | rs7150807 | 0.295801 | 0.259322 | 0.248493 | 0.357794 |
| cg04808349 | 8.57E-05 | 20 |  | 0.266189 | 0.243864 | 0.234972 | 0.342006 |
| cg04207436 | 8.55E-05 | 2 | rs542247823;rs556085285 | 0.403505 | 0.357449 | 0.360797 | 0.468245 |
| cg26690937 | 8.53E-05 | 8 | rs11777631;rs557075776 | 0.352593 | 0.306286 | 0.301341 | 0.421376 |
| cg03947738 | 8.52E-05 | 11 | rs549684151;rs117280875 | 0.301333 | 0.266521 | 0.254341 | 0.37147 |
| cg24475773 | 8.46E-05 | 5 | rs182068779 | 0.199684 | 0.173657 | 0.184953 | 0.270514 |
| cg01403010 | 8.45E-05 | 22 |  | 0.309491 | 0.28545 | 0.286145 | 0.359459 |
| cg18411994 | 8.44E-05 | 14 | rs570096850;rs537409257; rs558692539 | 0.337591 | 0.303324 | 0.310214 | 0.414299 |
| cg20956594 | 8.43E-05 | 13 | rs540172286;rs553693210 | 0.309372 | 0.280632 | 0.270696 | 0.393225 |
| cg14295935 | 8.42E-05 | 2 | rs558432582;rs576987324; rs534371033 | 0.276849 | 0.24796 | 0.241319 | 0.334621 |
| cg09359862 | 8.42E-05 | 10 | rs568630634;rs537424164; rs148741645;rs568648001 | 0.310725 | 0.283905 | 0.281712 | 0.38274 |
| cg22203678 | 8.41E-05 | 9 | rs140172204 | 0.402389 | 0.381686 | 0.387247 | 0.484733 |
| cg01910486 | 8.41E-05 | 12 |  | 0.359289 | 0.337732 | 0.335669 | 0.429338 |
| cg13412040 | 8.39E-05 | 14 | rs141994965 | 0.39026 | 0.352545 | 0.359552 | 0.477928 |
| cg12889391 | 8.36E-05 | 12 | rs539759872;rs11181376 | 0.317803 | 0.280815 | 0.278328 | 0.395324 |
| cg07550247 | 8.34E-05 | 20 | rs190566853;rs552229326 | 0.304395 | 0.267004 | 0.272911 | 0.361264 |
| cg09468055 | 8.34E-05 | 2 | rs140652694;rs146574472 | 0.319818 | 0.297757 | 0.291795 | 0.389813 |
| cg25274160 | 8.32E-05 | 5 |  | 0.156899 | 0.129879 | 0.131096 | 0.199793 |
| cg25255913 | 8.29E-05 | 1 |  | 0.238395 | 0.20266 | 0.201067 | 0.290672 |
| cg23173175 | 8.27E-05 | 13 | rs78089980;rs557968673; rs182487244 | 0.341051 | 0.30698 | 0.305214 | 0.413495 |
| cg08374619 | 8.26E-05 | 17 | rs543867643 | 0.3134 | 0.28463 | 0.287952 | 0.402823 |
| cg12621097 | 8.20E-05 | 9 | rs141902711;rs191535263; rs182168790;rs146269804 | 0.377037 | 0.341644 | 0.350074 | 0.456321 |
| cg00243880 | 8.18E-05 | 7 | rs189115419;rs572533307; rs73187816 | 0.355304 | 0.318407 | 0.315358 | 0.422813 |
| cg26284484 | 8.17E-05 | 11 | rs561298278;rs146432238; rs78377164 | 0.309419 | 0.279343 | 0.284022 | 0.375524 |
| cg19157948 | 8.16E-05 | 4 |  | 0.220322 | 0.196728 | 0.187372 | 0.284673 |
| cg13818121 | 8.13E-05 | 2 | rs181974532;rs148226209 | 0.508173 | 0.486557 | 0.501567 | 0.548683 |
| cg11253820 | 8.11E-05 | 17 | rs553694969;rs142980742; rs536037181;rs555964254 | 0.610474 | 0.58943 | 0.595158 | 0.67098 |
| cg04225088 | 8.06E-05 | 11 |  | 0.318373 | 0.279494 | 0.276205 | 0.411968 |
| cg19696103 | 8.06E-05 | 5 | rs143592008;rs550849347; rs192996544;rs530315056 | 0.387076 | 0.363266 | 0.344287 | 0.494951 |
| cg18790856 | 8.03E-05 | 2 | rs555280533;rs192858038 | 0.261427 | 0.211484 | 0.219874 | 0.336139 |
| cg01250398 | 8.02E-05 | 19 | rs575468505;rs73045696 | 0.326207 | 0.306174 | 0.289433 | 0.415956 |
| cg03214819 | 8.00E-05 | 17 | rs12936583;rs533828526 | 0.327135 | 0.293508 | 0.28474 | 0.421152 |
| cg02358983 | 7.99E-05 | 15 | rs4419010 | 0.28752 | 0.261555 | 0.247165 | 0.371024 |
| cg26073844 | 7.99E-05 | 8 | rs564810557 | 0.649022 | 0.627966 | 0.641774 | 0.70429 |
| cg26605164 | 7.96E-05 | 10 | rs573944064 | 0.311741 | 0.281394 | 0.28371 | 0.38014 |
| cg08918508 | 7.96E-05 | 13 | rs557221610;rs144701761; rs17553585 | 0.297323 | 0.26347 | 0.280009 | 0.379734 |
| cg25257256 | 7.96E-05 | 2 | rs566421326 | 0.279719 | 0.24387 | 0.238259 | 0.348746 |
| cg05026415 | 7.95E-05 | 4 | rs560428837;rs528049746 | 0.419549 | 0.398986 | 0.406441 | 0.463874 |
| cg19066932 | 7.93E-05 | 6 | rs566367003;rs565166675; rs139401986 | 0.238532 | 0.21027 | 0.20538 | 0.316803 |
| cg22559881 | 7.92E-05 | 12 | rs554335663 | 0.366065 | 0.335886 | 0.344268 | 0.435976 |
| cg10920722 | 7.92E-05 | 16 | rs537855513 | 0.287883 | 0.260912 | 0.258076 | 0.355186 |
| cg17491920 | 7.88E-05 | 9 | rs141676174;rs539510356; rs556222808;rs185640002 | 0.33912 | 0.315228 | 0.310609 | 0.415606 |
| cg12292594 | 7.88E-05 | 5 | rs541240869 | 0.386718 | 0.356829 | 0.373499 | 0.477285 |
| cg01498829 | 7.87E-05 | 14 | rs79850260;rs185720502; rs548307946;rs374511198; rs533794502 | 0.43273 | 0.411132 | 0.417648 | 0.483928 |
| cg08815499 | 7.85E-05 | 12 | rs149047434 | 0.322856 | 0.265383 | 0.260994 | 0.413675 |
| cg26873486 | 7.85E-05 | 1 | rs546953591 | 0.376147 | 0.350549 | 0.356632 | 0.432639 |
| cg16061152 | 7.85E-05 | 5 | rs572659269 | 0.264275 | 0.238937 | 0.249018 | 0.320758 |
| cg05116792 | 7.82E-05 | 5 | rs569439222;rs148344429; rs548346684 | 0.320151 | 0.283937 | 0.267347 | 0.392116 |
| cg14647416 | 7.74E-05 | 5 | rs146702597 | 0.283971 | 0.258694 | 0.251095 | 0.37082 |
| cg15549550 | 7.71E-05 | 2 | rs190270641 | 0.29429 | 0.268517 | 0.265559 | 0.367007 |
| cg16422316 | 7.67E-05 | 16 | rs539245070 | 0.318784 | 0.297409 | 0.280022 | 0.432528 |
| cg20720686 | 7.65E-05 | 7 | rs41295375 | 0.381907 | 0.341295 | 0.345351 | 0.458504 |
| cg02465404 | 7.63E-05 | 6 | rs9464307;rs549956196; rs570100801;rs73442197; rs549173914 | 0.383884 | 0.361712 | 0.355158 | 0.45135 |
| cg20373822 | 7.62E-05 | 5 |  | 0.290209 | 0.266784 | 0.270435 | 0.358581 |
| cg11016299 | 7.61E-05 | 1 |  | 0.157124 | 0.126339 | 0.135719 | 0.207583 |
| cg00621599 | 7.58E-05 | 1 | rs553382388 | 0.229394 | 0.207241 | 0.200633 | 0.324592 |
| cg18538723 | 7.57E-05 | 10 | rs141520373 | 0.39581 | 0.372351 | 0.380044 | 0.465715 |
| cg16402252 | 7.54E-05 | 18 | rs187392936;rs145020560 | 0.214782 | 0.190617 | 0.184437 | 0.271987 |
| cg13979622 | 7.49E-05 | 12 | rs140663953;rs527243582 | 0.261924 | 0.237922 | 0.226001 | 0.322468 |
| cg23476905 | 7.49E-05 | 4 | rs561565757;rs187015219; rs57306565 | 0.352326 | 0.324014 | 0.318655 | 0.423021 |
| cg17925976 | 7.49E-05 | 9 | rs7029837;rs150960012 | 0.267446 | 0.235175 | 0.21721 | 0.355303 |
| cg16894841 | 7.46E-05 | 10 | rs141125831;rs78189904 | 0.23005 | 0.203158 | 0.200565 | 0.299266 |
| cg05776899 | 7.46E-05 | 2 | rs545137412;rs184221674; rs549354588;rs560620460 | 0.444554 | 0.423979 | 0.437846 | 0.499449 |
| cg06857116 | 7.45E-05 | 17 | rs148461675;rs142633043 | 0.399579 | 0.377569 | 0.37703 | 0.486868 |
| cg06164037 | 7.44E-05 | 1 |  | 0.30118 | 0.278219 | 0.274205 | 0.369948 |
| cg22278294 | 7.44E-05 | 5 | rs533761090;rs544479289; rs144648246 | 0.302785 | 0.280092 | 0.263157 | 0.394184 |
| cg02459120 | 7.43E-05 | 18 |  | 0.430118 | 0.405276 | 0.409769 | 0.51815 |
| cg10456459 | 7.37E-05 | 12 | rs373083641;rs558762857 | 0.303199 | 0.259617 | 0.261712 | 0.399798 |
| cg06796035 | 7.37E-05 | 3 | rs114395051;rs111728124; rs530946805 | 0.545567 | 0.524056 | 0.52613 | 0.606037 |
| cg10924731 | 7.36E-05 | 16 | rs113068031;rs58627370 | 0.290081 | 0.262325 | 0.257516 | 0.357293 |
| cg00685236 | 7.35E-05 | 4 | rs180980870 | 0.68434 | 0.649321 | 0.641878 | 0.713294 |
| cg14190451 | 7.33E-05 | 11 | rs550358885;rs377038511; rs1108820 | 0.549778 | 0.528652 | 0.547349 | 0.608396 |
| cg04645556 | 7.33E-05 | 20 | rs554643272 | 0.317308 | 0.274134 | 0.281819 | 0.415008 |
| cg16464494 | 7.30E-05 | 14 | rs553648993 | 0.281864 | 0.252659 | 0.233071 | 0.357949 |
| cg03085538 | 7.29E-05 | 15 | rs191191050;rs552326105 | 0.272512 | 0.238241 | 0.232986 | 0.355434 |
| cg03653366 | 7.27E-05 | 13 | rs577690716;rs545225277 | 0.287462 | 0.260422 | 0.25564 | 0.365188 |
| cg04769861 | 7.26E-05 | 17 | rs547420838 | 0.338904 | 0.309597 | 0.310557 | 0.405589 |
| cg02193956 | 7.25E-05 | 4 | rs190261991;rs112630569; rs570122477;rs530950759 | 0.251314 | 0.214158 | 0.209559 | 0.34215 |
| cg16346844 | 7.23E-05 | 12 | rs3219275;rs538993598 | 0.567285 | 0.546165 | 0.557962 | 0.604193 |
| cg00780501 | 7.23E-05 | 3 | rs115739509;rs572813369; rs138073681 | 0.270806 | 0.242194 | 0.234019 | 0.332846 |
| cg26233331 | 7.22E-05 | 4 | rs552882005;rs572789605 | 0.256416 | 0.227248 | 0.230696 | 0.321735 |
| cg20627585 | 7.20E-05 | 7 | rs540519396;rs190691438 | 0.299923 | 0.27542 | 0.281302 | 0.353108 |
| cg12650721 | 7.19E-05 | 3 | rs537242878;rs557170901; rs567576513 | 0.309831 | 0.280737 | 0.264131 | 0.387412 |
| cg22716738 | 7.18E-05 | 7 | rs76103399;rs573337053; rs76232457 | 0.231329 | 0.198909 | 0.213318 | 0.303109 |
| cg10512006 | 7.17E-05 | 17 | rs577690879;rs528631620 | 0.545943 | 0.522997 | 0.531551 | 0.599837 |
| cg04353863 | 7.17E-05 | 8 | rs571569771 | 0.287277 | 0.253412 | 0.25466 | 0.374562 |
| cg19438764 | 7.15E-05 | 17 | rs138481031;rs534765015; rs192958871;rs565520939; rs536079418 | 0.320837 | 0.292657 | 0.281572 | 0.4032 |
| cg00112790 | 7.14E-05 | 13 | rs560861202 | 0.321604 | 0.27961 | 0.272218 | 0.399372 |
| cg20990473 | 7.12E-05 | 14 | rs139771425;rs547760994; rs568059444 | 0.303776 | 0.266813 | 0.261809 | 0.398513 |
| cg15958661 | 7.10E-05 | 1 | rs544899370;rs181943387; rs113109311 | 0.263361 | 0.24294 | 0.236278 | 0.329845 |
| cg02927618 | 7.07E-05 | 12 | rs183230470;rs543202566 | 0.667789 | 0.646844 | 0.666536 | 0.721077 |
| cg05425675 | 7.02E-05 | 6 | rs572496275;rs115822365; rs200941180;rs560391007 | 0.33415 | 0.313525 | 0.30884 | 0.413094 |
| cg07412002 | 7.02E-05 | 6 | rs115098925;rs189966116 | 0.3109 | 0.269879 | 0.268026 | 0.394424 |
| cg05473987 | 7.02E-05 | 5 | rs575168944 | 0.531149 | 0.509623 | 0.519091 | 0.577591 |
| cg08232510 | 7.01E-05 | 20 | rs192998426;rs139166727 | 0.281142 | 0.24452 | 0.231623 | 0.354478 |
| cg15726857 | 7.00E-05 | 12 | rs118135697 | 0.362378 | 0.341052 | 0.336062 | 0.416061 |
| cg11575996 | 6.98E-05 | 19 | rs188825125 | 0.377215 | 0.353824 | 0.346369 | 0.458807 |
| cg00500907 | 6.97E-05 | 18 | rs542617007;rs141810472 | 0.393322 | 0.360245 | 0.363177 | 0.492218 |
| cg01028226 | 6.96E-05 | 1 |  | 0.272398 | 0.245905 | 0.248337 | 0.3298 |
| cg17342301 | 6.95E-05 | 14 | rs537964605 | 0.451783 | 0.429091 | 0.430938 | 0.512836 |
| cg08732318 | 6.94E-05 | 12 | rs560994670;rs150030322; rs77911542 | 0.304904 | 0.271014 | 0.271909 | 0.377595 |
| cg17056618 | 6.92E-05 | 13 | rs566134445 | 0.498183 | 0.470869 | 0.479381 | 0.56354 |
| cg04031227 | 6.92E-05 | 6 | rs573574572;rs147010233 | 0.312021 | 0.275271 | 0.282026 | 0.38797 |
| cg01826979 | 6.91E-05 | 15 | rs540947693;rs565447559 | 0.562467 | 0.500534 | 0.49408 | 0.608778 |
| cg17833106 | 6.90E-05 | 1 | rs529922767 | 0.34234 | 0.313561 | 0.309336 | 0.412017 |
| cg02023149 | 6.89E-05 | 3 | rs556988651;rs573764049 | 0.331043 | 0.29514 | 0.296661 | 0.414603 |
| cg01763719 | 6.89E-05 | 2 | rs62194969;rs545982247 | 0.288307 | 0.259756 | 0.252031 | 0.384285 |
| cg21339473 | 6.86E-05 | 11 | rs532397964 | 0.298987 | 0.263191 | 0.254764 | 0.348168 |
| cg16812044 | 6.83E-05 | 1 | rs29000584 | 0.259992 | 0.239523 | 0.229741 | 0.350398 |
| cg06512115 | 6.83E-05 | 2 | rs140483585;rs150010414; rs563504900 | 0.300981 | 0.273783 | 0.266639 | 0.404251 |
| cg24629939 | 6.83E-05 | 10 | rs556881470;rs11198607; rs545418808 | 0.335498 | 0.311547 | 0.323621 | 0.414547 |
| cg14585083 | 6.79E-05 | 20 | rs544987432;rs114089259 | 0.299274 | 0.269974 | 0.289837 | 0.390392 |
| cg09674502 | 6.77E-05 | 1 |  | 0.392462 | 0.362466 | 0.350217 | 0.483727 |
| cg22970261 | 6.77E-05 | 22 | rs373115335;rs562976314 | 0.306101 | 0.27463 | 0.27225 | 0.370371 |
| cg22387323 | 6.76E-05 | 4 | rs144181135;rs556374607; rs570211229;rs535827899 | 0.407644 | 0.383607 | 0.388106 | 0.46393 |
| cg18938128 | 6.69E-05 | 16 |  | 0.289605 | 0.261009 | 0.262071 | 0.378742 |
| cg21247370 | 6.69E-05 | 3 |  | 0.206098 | 0.176932 | 0.175344 | 0.25399 |
| cg22718748 | 6.65E-05 | 10 | rs368568154;rs370644899 | 0.303787 | 0.273645 | 0.263485 | 0.390596 |
| cg01894508 | 6.63E-05 | 2 | rs529195418;rs77274135 | 0.50375 | 0.483139 | 0.494344 | 0.584947 |
| cg23546106 | 6.62E-05 | 2 | rs150256775;rs72944545; rs114065060 | 0.476077 | 0.451614 | 0.434899 | 0.545508 |
| cg15163119 | 6.61E-05 | 9 | rs370941866;rs527815579 | 0.296579 | 0.259935 | 0.248186 | 0.382736 |
| cg14565465 | 6.61E-05 | 9 |  | 0.24712 | 0.225045 | 0.225899 | 0.300174 |
| cg10289699 | 6.59E-05 | 6 |  | 0.285436 | 0.261064 | 0.247793 | 0.37406 |
| cg26876444 | 6.58E-05 | 1 | rs138691306;rs141121976 | 0.171608 | 0.142124 | 0.154747 | 0.220167 |
| cg26626205 | 6.57E-05 | 1 | rs550942870 | 0.219064 | 0.19697 | 0.199976 | 0.264762 |
| cg06937882 | 6.57E-05 | 20 | rs112016963;rs75726546; rs6114981 | 0.332334 | 0.306169 | 0.298735 | 0.407385 |
| cg07591278 | 6.56E-05 | 3 | rs193259807 | 0.179922 | 0.153171 | 0.172727 | 0.24306 |
| cg22888414 | 6.56E-05 | 10 |  | 0.504284 | 0.484272 | 0.486504 | 0.567635 |
| cg20312012 | 6.56E-05 | 2 | rs548975059;rs115642922; rs534675746 | 0.291407 | 0.253698 | 0.24566 | 0.390212 |
| cg06052655 | 6.51E-05 | 6 | rs142653954;rs576186132; rs543247592 | 0.254202 | 0.22511 | 0.205832 | 0.329321 |
| cg24651941 | 6.51E-05 | 20 | rs368967171;rs571361814; rs117228763;rs192432852 | 0.550635 | 0.52973 | 0.546171 | NA |
| cg24530587 | 6.49E-05 | X |  | 0.49707 | 0.466611 | 0.472799 | 0.555276 |
| cg17126294 | 6.46E-05 | 7 | rs550112371;rs184873554 | 0.356392 | 0.333749 | 0.331874 | 0.440024 |
| cg04201370 | 6.46E-05 | 5 | rs569061597 | 0.531244 | 0.510826 | 0.50544 | 0.603095 |
| cg00158770 | 6.45E-05 | 8 | rs11780388;rs577277886; rs546205013;rs181974457 | 0.377629 | 0.352712 | 0.348988 | 0.46027 |
| cg18911718 | 6.45E-05 | 5 | rs116589625;rs138521871; rs76264417 | 0.308449 | 0.278298 | 0.269799 | 0.376806 |
| cg13057273 | 6.44E-05 | 14 | rs574827610;rs542108640; rs559764229 | 0.277218 | 0.255056 | 0.241726 | 0.359033 |
| cg23969787 | 6.44E-05 | 19 | rs573592239;rs199824285; rs116046113 | 0.325542 | 0.289066 | 0.283449 | 0.391761 |
| cg04202597 | 6.39E-05 | 1 |  | 0.336219 | 0.314164 | 0.303269 | 0.422388 |
| cg09404747 | 6.37E-05 | 5 | rs147780765;rs563466153 | 0.399041 | 0.373523 | 0.378407 | 0.473848 |
| cg19830670 | 6.33E-05 | 4 | rs575440192;rs150918991; rs554447231 | 0.281947 | 0.260359 | 0.245445 | 0.37245 |
| cg08363114 | 6.31E-05 | 19 | rs149935834;rs2304523; rs191824521 | 0.479964 | 0.426485 | 0.43521 | 0.560799 |
| cg02786313 | 6.31E-05 | 10 | rs7085104;rs188214797; rs552228590;rs570579972; rs537654720 | 0.635755 | 0.613968 | 0.613102 | 0.680796 |
| cg03936701 | 6.28E-05 | 6 | rs568058452 | 0.289411 | 0.250883 | 0.241632 | 0.385753 |
| cg15033511 | 6.23E-05 | 17 | rs138168637;rs551908298; rs533475009;rs531266639 | 0.532024 | 0.507552 | 0.491225 | 0.633334 |
| cg15458313 | 6.20E-05 | 6 | rs558405706;rs575266868 | 0.275458 | 0.243207 | 0.223479 | 0.362082 |
| cg19408765 | 6.20E-05 | 3 | rs566791513;rs532172213; rs192558003;rs571603074 | 0.241488 | 0.216955 | 0.201655 | 0.293546 |
| cg12296597 | 6.20E-05 | 6 | rs544430438;rs1207170; rs575118344 | 0.488918 | 0.455076 | 0.467098 | 0.568319 |
| cg16407947 | 6.20E-05 | 10 | rs576852325;rs6602322 | 0.263845 | 0.23914 | 0.232719 | 0.342259 |
| cg06122230 | 6.20E-05 | 20 |  | 0.43935 | 0.403939 | 0.394311 | 0.539687 |
| cg19738122 | 6.18E-05 | 4 | rs550275905 | 0.232583 | 0.198922 | 0.206997 | 0.277878 |
| cg00397616 | 6.16E-05 | 3 | rs78793264 | 0.340408 | 0.315961 | 0.308164 | 0.416137 |
| cg11010932 | 6.16E-05 | 9 | rs561191850 | 0.372662 | 0.341456 | 0.349683 | 0.43819 |
| cg11097064 | 6.11E-05 | 2 | rs549247261 | 0.288727 | 0.260055 | 0.260363 | 0.357188 |
| cg04165922 | 6.10E-05 | 1 | rs181978896;rs547902696; rs566538006 | 0.332202 | 0.291777 | 0.277019 | 0.43885 |
| cg12330118 | 6.05E-05 | 2 | rs145360991;rs566037333; rs536409486;rs554760474 | 0.30801 | 0.275084 | 0.280668 | 0.37832 |
| cg14266770 | 6.04E-05 | 9 | rs558830335;rs576963220; rs117786874 | 0.351438 | 0.323918 | 0.308275 | 0.415291 |
| cg07026750 | 6.02E-05 | 4 | rs554279147;rs566154788 | 0.254672 | 0.214757 | 0.222367 | 0.326271 |
| cg02980737 | 6.01E-05 | 9 | rs573106841 | 0.38309 | 0.361638 | 0.377109 | 0.470837 |
| cg14632570 | 6.01E-05 | 20 | rs572288201;rs117610184 | 0.284942 | 0.253805 | 0.243123 | 0.348873 |
| cg07297964 | 6.01E-05 | 13 | rs528285110;rs113692325 | 0.426066 | 0.400443 | 0.409359 | 0.500211 |
| cg07520477 | 6.01E-05 | 17 | rs144208250;rs146537608; rs141309679;rs559328323; rs182100279 | 0.380683 | 0.349577 | 0.35597 | 0.463876 |
| cg04099543 | 6.01E-05 | 11 | rs112532953 | 0.327799 | 0.301814 | 0.297264 | 0.373213 |
| cg14727962 | 6.00E-05 | 5 | rs529423392;rs541488986; rs115369976;rs183118721 | 0.255306 | 0.229349 | 0.236595 | 0.312385 |
| cg19196676 | 6.00E-05 | 12 | rs574939165;rs535900703; rs144112576 | 0.289479 | 0.26946 | 0.25316 | 0.390875 |
| cg19100344 | 5.99E-05 | 1 | rs112801145 | 0.297604 | 0.274334 | 0.262741 | 0.388042 |
| cg20400045 | 5.98E-05 | 13 | rs147145112;rs193097818; rs115501759 | 0.298855 | 0.256455 | 0.264504 | 0.385451 |
| cg19470726 | 5.98E-05 | 1 | rs12039330 | 0.321166 | 0.292524 | 0.285101 | 0.413202 |
| cg10430168 | 5.98E-05 | 17 | rs573670105 | 0.599667 | 0.578311 | 0.59351 | 0.67058 |
| cg02979487 | 5.96E-05 | 4 | rs17012412 | 0.203791 | 0.172269 | 0.173189 | 0.251135 |
| cg01976035 | 5.96E-05 | 2 |  | 0.26278 | 0.231392 | 0.220276 | 0.355724 |
| cg17261827 | 5.96E-05 | 1 |  | 0.608151 | 0.582025 | 0.590816 | 0.662012 |
| cg04650604 | 5.95E-05 | 5 |  | 0.298386 | 0.272673 | 0.271031 | 0.386386 |
| cg17446956 | 5.92E-05 | 12 | rs181069951;rs555750265 | 0.388381 | 0.363707 | 0.37394 | 0.477708 |
| cg02916841 | 5.92E-05 | 16 | rs529497497;rs145798727; rs563098908;rs367860950 | 0.168972 | 0.147991 | 0.14768 | 0.208133 |
| cg07060794 | 5.86E-05 | 7 | rs147267580;rs36057757; rs202212306;rs145205704; rs151010616;rs573484529 | 0.307818 | 0.285191 | 0.275278 | NA |
| cg03537386 | 5.83E-05 | 1 | rs190330126;rs11589456 | 0.529114 | 0.503525 | 0.522874 | 0.597084 |
| cg14573371 | 5.83E-05 | 6 | rs189906998 | 0.308291 | 0.277482 | 0.260749 | 0.403744 |
| cg03500547 | 5.80E-05 | 9 | rs142536456;rs577919649; rs545427623;rs563629253 | 0.661666 | 0.635348 | 0.64069 | 0.686468 |
| cg26126031 | 5.79E-05 | 22 | rs186140569;rs116114915; rs529509908 | 0.296823 | 0.276182 | 0.279794 | 0.383801 |
| cg21704551 | 5.78E-05 | 8 |  | 0.397561 | 0.350697 | 0.358039 | 0.500592 |
| cg01239499 | 5.75E-05 | 7 | rs144948679 | 0.324215 | 0.286414 | 0.282286 | 0.424256 |
| cg01026915 | 5.73E-05 | 20 | rs375941679;rs553550102; rs574898504;rs542150995 | 0.309353 | 0.284491 | 0.279706 | 0.359658 |
| cg25025181 | 5.68E-05 | 1 | rs576150097;rs542164866 | 0.24075 | 0.194013 | 0.209764 | 0.31989 |
| cg01606650 | 5.67E-05 | 5 | rs541794433 | 0.264996 | 0.234855 | 0.232591 | 0.34135 |
| cg18790427 | 5.65E-05 | 2 | rs6761422;rs530242724; rs548357228 | 0.371521 | 0.345268 | 0.333872 | 0.453918 |
| cg07988171 | 5.65E-05 | 19 |  | 0.668268 | 0.630639 | 0.639147 | 0.723257 |
| cg01821345 | 5.65E-05 | 17 | rs538675585;rs547167791; rs75856527 | 0.324648 | 0.296445 | 0.289556 | 0.437548 |
| cg04244170 | 5.64E-05 | 3 | rs530955652;rs565054898; rs140181407 | 0.273969 | 0.246355 | 0.238344 | 0.370637 |
| cg25486883 | 5.63E-05 | 6 | rs7742053 | 0.353006 | 0.327007 | 0.309146 | 0.426953 |
| cg02609911 | 5.62E-05 | 8 | rs536270360 | 0.264621 | 0.237353 | 0.232859 | 0.354575 |
| cg25067419 | 5.62E-05 | 8 | rs75413393;rs574872881 | 0.332482 | 0.307023 | 0.296107 | 0.420593 |
| cg14428204 | 5.62E-05 | 12 | rs533388053;rs79388527; rs566959214 | 0.279559 | 0.243188 | 0.224563 | 0.3668 |
| cg26942829 | 5.61E-05 | 6 |  | 0.265321 | 0.24256 | 0.226568 | 0.344619 |
| cg02694449 | 5.55E-05 | 6 | rs531671104;rs551506214 | 0.638724 | 0.613627 | 0.622469 | 0.699266 |
| cg06144210 | 5.54E-05 | 6 | rs6939397 | 0.33262 | 0.306205 | 0.302402 | 0.410822 |
| cg08966997 | 5.53E-05 | 7 | rs572464929;rs546240340 | 0.302517 | 0.278235 | 0.277586 | 0.359807 |
| cg00677533 | 5.51E-05 | 4 | rs565432908;rs187346608 | 0.271652 | 0.241492 | 0.235407 | 0.350483 |
| cg14586711 | 5.48E-05 | 2 | rs568770529;rs537699310; rs557628158;rs577362852 | 0.291627 | 0.269271 | 0.253732 | 0.356565 |
| cg17901584 | 5.45E-05 | 1 | rs531880988;rs187474148 | 0.377868 | 0.349988 | 0.33139 | 0.474594 |
| cg05075327 | 5.45E-05 | 9 | rs190408299;rs112963862 | 0.334942 | 0.308929 | 0.290078 | 0.411292 |
| cg04015186 | 5.43E-05 | 3 | rs565830629 | 0.19198 | 0.167355 | 0.16342 | 0.249922 |
| cg08754446 | 5.41E-05 | 6 |  | 0.447185 | 0.419875 | 0.428728 | 0.513101 |
| cg01297352 | 5.40E-05 | 1 | rs139897310 | 0.317658 | 0.276554 | 0.282009 | 0.411049 |
| cg08801415 | 5.39E-05 | 20 | rs557674056;rs577379792 | 0.338159 | 0.316758 | 0.32882 | 0.430543 |
| cg20882551 | 5.33E-05 | 20 |  | 0.348633 | 0.325147 | 0.327477 | 0.435042 |
| cg19186970 | 5.33E-05 | 13 |  | 0.245821 | 0.218009 | 0.217174 | 0.352231 |
| cg22061832 | 5.30E-05 | 2 | rs574136534;rs78794133 | 0.280854 | 0.249957 | 0.245932 | 0.373867 |
| cg06204222 | 5.29E-05 | 12 | rs550413129;rs60950915 | 0.266158 | 0.238893 | 0.234863 | 0.348307 |
| cg24025566 | 5.29E-05 | 20 | rs549826382 | 0.73627 | 0.713716 | 0.725924 | 0.761804 |
| cg08688659 | 5.28E-05 | 11 | rs570322832;rs189527367; rs552540058 | 0.309346 | 0.26655 | 0.257285 | 0.397344 |
| cg14730017 | 5.26E-05 | 9 | rs547251301 | 0.28813 | 0.245516 | 0.241089 | 0.384735 |
| cg23304298 | 5.23E-05 | 14 | rs190051334;rs148659526; rs61987841 | 0.368689 | 0.34265 | 0.344649 | 0.45354 |
| cg20153863 | 5.23E-05 | 3 | rs561077918;rs531289422; rs13098911 | 0.273593 | 0.241658 | 0.233406 | 0.344394 |
| cg17910071 | 5.23E-05 | 11 | rs11309849;rs543880674 | 0.533109 | 0.511042 | 0.512766 | 0.584329 |
| cg05913590 | 5.20E-05 | 6 | rs552030619;rs565475105 | 0.235043 | 0.205003 | 0.2011 | 0.314532 |
| cg19930657 | 5.19E-05 | 3 |  | 0.267021 | 0.235302 | 0.222205 | 0.345679 |
| cg05117208 | 5.17E-05 | 17 | rs567817142;rs538070648 | 0.317336 | 0.293846 | 0.28695 | 0.396835 |
| cg19866404 | 5.17E-05 | 1 | rs188143663;rs180854352; rs532285081 | 0.285285 | 0.244323 | 0.241252 | 0.363535 |
| cg09589182 | 5.15E-05 | 14 | rs578237434;rs543767593 | 0.293245 | 0.267112 | 0.258136 | 0.375704 |
| cg02459648 | 5.14E-05 | 12 | rs537578316 | 0.326451 | 0.294717 | 0.298148 | 0.40275 |
| cg12079904 | 5.14E-05 | 2 | rs183822664;rs144564359 | 0.338738 | 0.316595 | 0.313993 | 0.399002 |
| cg03732627 | 5.12E-05 | 15 |  | 0.272014 | 0.246317 | 0.236113 | 0.367072 |
| cg13132858 | 5.12E-05 | 1 | rs566301152;rs145156659; rs139040249;rs1345529; rs571074838 | 0.305764 | 0.270821 | 0.263468 | 0.390243 |
| cg12594803 | 5.11E-05 | 3 | rs186387963 | 0.217051 | 0.192007 | 0.179638 | 0.281737 |
| cg13230994 | 5.11E-05 | 11 | rs575816805 | 0.305244 | 0.274959 | 0.267745 | 0.378196 |
| cg09524686 | 5.10E-05 | 11 | rs147272152;rs563265389 | 0.264673 | 0.240809 | 0.234638 | 0.319321 |
| cg05162166 | 5.08E-05 | 22 | rs1984584 | 0.345933 | 0.32246 | 0.321301 | 0.413887 |
| cg03519005 | 5.08E-05 | 9 | rs536863116;rs115442508 | 0.314301 | 0.285077 | 0.269938 | 0.378879 |
| cg04489053 | 5.06E-05 | 12 | rs189309753;rs117494432 | 0.351215 | 0.316719 | 0.313354 | 0.443913 |
| cg18853017 | 5.06E-05 | 17 | rs545011560 | 0.294704 | 0.268017 | 0.253988 | 0.376467 |
| cg25480460 | 5.06E-05 | 11 | rs564532349;rs533623952; rs189992045 | 0.287682 | 0.256967 | 0.264009 | 0.350866 |
| cg01182697 | 5.05E-05 | 1 |  | 0.24104 | 0.217923 | 0.209439 | 0.301252 |
| cg03122695 | 5.05E-05 | 14 | rs546469843;rs143881611; rs148578705;rs529159730; rs74082238 | 0.493623 | 0.472697 | 0.480374 | 0.551232 |
| cg26655028 | 5.03E-05 | 4 | rs186914561;rs72624135 | 0.268475 | 0.237506 | 0.228656 | 0.350063 |
| cg19974686 | 5.03E-05 | 3 | rs7651990;rs187542010; rs112925561 | 0.277483 | 0.251754 | 0.242535 | 0.36249 |
| cg03193657 | 5.01E-05 | 15 | rs533766017 | 0.270416 | 0.247008 | 0.23927 | 0.351081 |
| cg25498843 | 5.00E-05 | 15 | rs559426852;rs150768376 | 0.551877 | 0.531241 | 0.545813 | 0.627155 |
| cg02872054 | 5.00E-05 | 14 | rs186268773 | 0.126197 | 0.105999 | 0.121669 | 0.164775 |
| cg08710628 | 4.99E-05 | 7 | rs587762038;rs587636776; rs587716755;rs587746527; rs140401769 | 0.289142 | 0.253655 | 0.248561 | 0.382685 |
| cg12411992 | 4.98E-05 | 11 | rs144594301;rs12797891; rs138540266;rs141163669 | 0.352526 | 0.311729 | 0.303158 | 0.45095 |
| cg20574018 | 4.96E-05 | 15 | rs558306939;rs578088474; rs540735182 | 0.343413 | 0.305612 | 0.30765 | 0.441562 |
| cg13103915 | 4.96E-05 | 5 | rs557773980;rs578039857 | 0.50977 | 0.48964 | 0.502289 | 0.566198 |
| cg07172882 | 4.95E-05 | 4 | rs188688626;rs553544675 | 0.44929 | 0.418192 | 0.43091 | 0.517908 |
| cg23244697 | 4.94E-05 | 8 | rs139509424 | 0.446453 | 0.42539 | 0.434127 | 0.512996 |
| cg26869364 | 4.94E-05 | 17 | rs534688910 | 0.379604 | 0.355931 | 0.357071 | NA |
| cg19713330 | 4.94E-05 | 15 | rs534751852;rs116684278 | 0.29476 | 0.272655 | 0.257892 | 0.368505 |
| cg01469583 | 4.92E-05 | 2 | rs562234282 | 0.32911 | 0.306066 | 0.297358 | 0.411078 |
| cg22143387 | 4.90E-05 | 5 | rs147436495;rs560345996; rs572505982 | 0.50364 | 0.482749 | 0.497586 | 0.555264 |
| cg09509305 | 4.89E-05 | 3 | rs111428372 | 0.245843 | 0.215522 | 0.212702 | 0.320733 |
| cg00817490 | 4.87E-05 | 16 | rs573487082 | 0.526839 | 0.506609 | 0.513961 | 0.585089 |
| cg11561701 | 4.86E-05 | 22 | rs189304547;rs535357662; rs555648366 | 0.284019 | 0.254338 | 0.252168 | 0.363261 |
| cg01568243 | 4.86E-05 | 9 | rs552159874;rs182001281; rs528211295 | 0.243619 | 0.219355 | 0.218719 | 0.30612 |
| cg08036492 | 4.85E-05 | 17 | rs189492534;rs181765228; rs541640775;rs116204106; rs142153160 | 0.303834 | 0.278452 | 0.263098 | 0.395238 |
| cg10890106 | 4.83E-05 | 7 | rs532150028 | 0.315054 | 0.277442 | 0.271883 | 0.395674 |
| cg19499884 | 4.80E-05 | 10 | rs553176448;rs531006530 | 0.304512 | 0.280091 | 0.277719 | 0.377734 |
| cg03002688 | 4.79E-05 | 4 | rs545292091;rs77849807; rs530480750 | 0.407541 | 0.384877 | 0.389635 | 0.483197 |
| cg11335355 | 4.78E-05 | 2 | rs534957226;rs139081820 | 0.282925 | 0.261735 | 0.250348 | 0.391033 |
| cg17212314 | 4.77E-05 | 15 | rs72702744;rs530042937 | 0.267901 | 0.220906 | 0.216594 | 0.328172 |
| cg25282281 | 4.76E-05 | 4 | rs73856959;rs187438789 | 0.363178 | 0.338987 | 0.342639 | 0.445467 |
| cg25541103 | 4.75E-05 | 11 | rs149826059;rs191528650 | 0.689931 | 0.66806 | 0.674627 | 0.741286 |
| cg20223214 | 4.75E-05 | 14 | rs550368845 | 0.29112 | 0.255691 | 0.269853 | 0.364016 |
| cg21141827 | 4.74E-05 | 5 | rs570619096;rs534838703; rs553425220 | 0.29386 | 0.270022 | 0.272593 | 0.395858 |
| cg06084585 | 4.73E-05 | 13 | rs79596055 | 0.284519 | 0.254784 | 0.235566 | 0.35299 |
| cg24595580 | 4.71E-05 | 15 | rs189973573;rs116193392 | 0.302422 | 0.27414 | 0.254405 | 0.367506 |
| cg07115878 | 4.71E-05 | 1 | rs115967540;rs145685744; rs74366611 | 0.262096 | 0.227999 | 0.228823 | 0.354167 |
| cg24675644 | 4.70E-05 | 3 | rs181519981 | 0.30702 | 0.270345 | 0.260512 | 0.396166 |
| cg21563567 | 4.69E-05 | 3 |  | 0.357385 | 0.319655 | 0.308515 | 0.454525 |
| cg17230002 | 4.69E-05 | 12 | rs79991164 | 0.483169 | 0.461281 | 0.466578 | 0.557859 |
| cg00502509 | 4.67E-05 | 7 | rs559548176;rs528298601; rs548410495;rs375829161; rs190365730 | 0.892544 | 0.870599 | 0.863709 | 0.929003 |
| cg07078052 | 4.65E-05 | 5 | rs539164005;rs550946593; rs114867518;rs536908527 | 0.197808 | 0.175146 | 0.169603 | 0.234223 |
| cg17899674 | 4.65E-05 | 2 | rs184893759;rs574601610; rs6751295 | 0.306023 | 0.257177 | 0.250464 | 0.389057 |
| cg05067204 | 4.64E-05 | 19 | rs549628147;rs142155110 | 0.354746 | 0.326322 | 0.313752 | 0.430946 |
| cg09000622 | 4.64E-05 | 1 | rs138246702;rs550443882; rs113566235;rs529962010 | 0.292545 | 0.253172 | 0.247083 | 0.357276 |
| cg09599495 | 4.63E-05 | 4 | rs566846408 | 0.315198 | 0.292637 | 0.297857 | 0.396319 |
| cg01389727 | 4.62E-05 | 16 | rs150754966 | 0.540649 | 0.519184 | 0.517301 | 0.59592 |
| cg23585881 | 4.61E-05 | 16 | rs141194325;rs150778585 | 0.271913 | 0.238963 | 0.231628 | 0.336216 |
| cg10997193 | 4.59E-05 | 7 |  | 0.350259 | 0.329658 | 0.317309 | 0.417264 |
| cg15481493 | 4.59E-05 | 11 | rs547784661;rs566329830 | 0.412535 | 0.38902 | 0.396681 | 0.484809 |
| cg19750024 | 4.58E-05 | 8 | rs569677560;rs554274682; rs114314677;rs539867723 | 0.324417 | 0.302305 | 0.299837 | 0.375454 |
| cg20810503 | 4.57E-05 | 8 | rs558544544;rs576779457; rs28439079;rs552982485 | 0.35998 | 0.330364 | 0.336503 | 0.474217 |
| cg26827532 | 4.57E-05 | 11 | rs149071695 | 0.356598 | 0.333313 | 0.338986 | 0.417553 |
| cg07628474 | 4.55E-05 | 14 | rs185867624; rs535952636 | 0.284211 | 0.254157 | 0.255802 | 0.390627 |
| cg14082278 | 4.53E-05 | 3 |  | 0.294575 | 0.269607 | 0.26027 | 0.39767 |
| cg14850568 | 4.52E-05 | 5 | rs556189159;rs568221459; r535431893;rs553860911 | 0.375498 | 0.349975 | 0.349921 | 0.465305 |
| cg17743403 | 4.52E-05 | 2 | rs556374812;rs375393312 | 0.251209 | 0.218835 | 0.214315 | 0.313644 |
| cg02969426 | 4.50E-05 | 13 | rs574477659;rs182085758 | 0.456841 | 0.436733 | 0.454459 | 0.549622 |
| cg00772428 | 4.48E-05 | 15 | rs572804086 | 0.270432 | 0.242608 | 0.234529 | 0.358442 |
| cg20084702 | 4.47E-05 | 20 | rs574741166;rs541967056; rs563752904;rs371174206; rs551941446;rs144996202; rs149124579;rs546842346 | 0.358638 | 0.335821 | 0.328119 | 0.440566 |
| cg10453850 | 4.47E-05 | 6 | rs139997170;rs536612605; rs117660980 | 0.273389 | 0.236719 | 0.234159 | 0.325739 |
| cg19093019 | 4.46E-05 | 22 | rs146880230;rs62230495 | 0.289456 | 0.266172 | 0.257342 | 0.351093 |
| cg09767156 | 4.46E-05 | 1 | rs184824263;rs144024416; rs140657712;rs575659934; rs72769488;rs554887992 | 0.303022 | 0.278379 | 0.27787 | 0.379445 |
| cg00965449 | 4.46E-05 | 13 | rs187936632;rs558552811; rs565891163;rs543208597 | 0.460016 | 0.427813 | 0.433193 | 0.530179 |
| cg18897025 | 4.44E-05 | 5 | rs76271681 | 0.31417 | 0.276961 | 0.272601 | 0.413281 |
| cg10862709 | 4.44E-05 | 3 | rs149978649 | 0.210465 | 0.177394 | 0.175622 | 0.27438 |
| cg19854060 | 4.36E-05 | 17 | rs532270936 | 0.296261 | 0.268434 | 0.260645 | 0.37463 |
| cg15117322 | 4.34E-05 | 12 | rs115081286;rs191255254 | 0.340375 | 0.314385 | 0.318329 | 0.422472 |
| cg21724658 | 4.33E-05 | 5 | rs563074991 | 0.472391 | 0.440232 | 0.452605 | 0.554597 |
| cg08982816 | 4.28E-05 | 5 | rs185553645;rs190652193 | 0.286805 | 0.266072 | 0.252416 | 0.368281 |
| cg21626302 | 4.28E-05 | 9 | rs142590822;rs552260358 | 0.272861 | 0.249339 | 0.243418 | 0.363097 |
| cg10834070 | 4.27E-05 | 3 | rs567499191;rs529935692; rs2028874;rs566084363 | 0.282617 | 0.259752 | 0.242971 | 0.385369 |
| cg26342200 | 4.27E-05 | 2 | rs575305232 | 0.358092 | 0.332339 | 0.31529 | 0.446445 |
| cg23256108 | 4.27E-05 | 11 | rs144635353;rs146696759 | 0.355828 | 0.335214 | 0.334222 | 0.418964 |
| cg06374595 | 4.25E-05 | 2 | rs548099504;rs191233194 | 0.364888 | 0.333953 | 0.336671 | 0.43995 |
| cg13051649 | 4.22E-05 | 17 | rs539879571;rs187387695 | 0.400132 | 0.378314 | 0.371966 | 0.480985 |
| cg12507840 | 4.22E-05 | 13 | rs377433575;rs537666689; rs2149261 | 0.246675 | 0.220646 | 0.206341 | 0.323041 |
| cg25210609 | 4.21E-05 | 1 | rs536811271;rs2073099;r s181310745;rs575899610; rs185127420 | 0.228401 | 0.204759 | 0.206143 | 0.308442 |
| cg15262865 | 4.19E-05 | 5 | rs537970818;rs533470724 | 0.313159 | 0.287511 | 0.280679 | 0.389375 |
| cg11260662 | 4.18E-05 | 14 | rs534860028;rs548291123 | 0.34973 | 0.310234 | 0.305403 | 0.465571 |
| cg10679775 | 4.15E-05 | 18 | rs542603009 | 0.444978 | 0.419148 | 0.420817 | 0.537566 |
| cg26443127 | 4.10E-05 | 10 | rs577657603;rs10826979; rs370545502 | 0.361651 | 0.32211 | 0.317463 | 0.430506 |
| cg20757369 | 4.09E-05 | 2 | rs200816451;rs562134659; rs143647600;rs142888260 | 0.201484 | 0.176937 | 0.166948 | 0.253159 |
| cg10535895 | 4.07E-05 | 19 | rs191133599 | 0.260596 | 0.228981 | 0.21478 | 0.321772 |
| cg03562940 | 4.07E-05 | 3 | rs112568975;rs570916311; rs528569199;rs141939737 | 0.357215 | 0.309877 | 0.29926 | 0.455385 |
| cg18421604 | 4.06E-05 | 12 | rs555793233 | 0.315659 | 0.277595 | 0.277016 | 0.402128 |
| cg01944288 | 4.06E-05 | 9 | rs549944729 | 0.209498 | 0.18391 | 0.1767 | 0.263949 |
| cg22406187 | 4.02E-05 | 5 | rs76064750 | 0.307074 | 0.277495 | 0.267384 | 0.366251 |
| cg07500621 | 4.02E-05 | 5 | rs535511361;rs555203062; rs572563114 | 0.338959 | 0.314364 | 0.311637 | 0.416816 |
| cg07349477 | 4.00E-05 | 12 | rs140486002 | 0.287916 | 0.262721 | 0.246315 | 0.378891 |
| cg15931606 | 3.97E-05 | 3 | rs571222801;rs183941209 | 0.381524 | 0.350838 | 0.341907 | 0.469691 |
| cg19758817 | 3.97E-05 | 2 |  | 0.395592 | 0.355696 | 0.357985 | 0.480531 |
| cg05406863 | 3.97E-05 | 2 | rs77335736 | 0.291312 | 0.264518 | 0.254237 | 0.374779 |
| cg12865675 | 3.95E-05 | 5 | rs555502467;rs12054833; rs573515559 | 0.266552 | 0.232755 | 0.229825 | 0.364587 |
| cg08275159 | 3.94E-05 | 6 | rs113194955;rs531721117; rs188509087;rs17145857 | 0.382817 | 0.360548 | 0.354681 | 0.441298 |
| cg21664857 | 3.91E-05 | 20 | rs527537812;rs552097705; rs186227447;rs537620030 | 0.240967 | 0.219539 | 0.209383 | 0.304425 |
| cg15015109 | 3.90E-05 | 17 | rs147265471;rs182292054 | 0.343917 | 0.322254 | 0.308279 | 0.415859 |
| cg04655481 | 3.89E-05 | 9 | rs199876596 | 0.258263 | 0.237665 | 0.239885 | 0.312593 |
| cg00534516 | 3.89E-05 | 5 | rs145114434;rs540835146 | 0.361721 | 0.325368 | 0.330867 | 0.448963 |
| cg22079412 | 3.86E-05 | 12 | rs149487078;rs552587865; rs559527588 | 0.312071 | 0.275476 | 0.266181 | 0.388718 |
| cg21009562 | 3.84E-05 | 3 | rs541345970 | 0.388914 | 0.358013 | 0.369233 | 0.472313 |
| cg04052546 | 3.83E-05 | 2 | rs78793148;rs148330963; rs141503536 | 0.327605 | 0.29235 | 0.282253 | 0.406201 |
| cg01119452 | 3.83E-05 | 7 |  | 0.274207 | 0.249958 | 0.235563 | 0.352225 |
| cg06967020 | 3.82E-05 | 1 | rs145002995;rs147131844; rs61193942;rs115548884 | 0.373023 | 0.349829 | 0.346304 | 0.434626 |
| cg02622874 | 3.81E-05 | 4 | rs566974771;rs368454603 | 0.187068 | 0.160439 | 0.156631 | 0.242869 |
| cg02255986 | 3.80E-05 | 6 |  | 0.322064 | 0.290708 | 0.286988 | 0.408052 |
| cg14898111 | 3.78E-05 | 6 | rs555229449 | 0.290816 | 0.257699 | 0.248754 | 0.364239 |
| cg12529561 | 3.76E-05 | 3 | rs537504458;rs557194586; rs577134501;rs545911209; rs553229539;rs573249232 | 0.309765 | 0.278017 | 0.269362 | 0.412662 |
| cg25058201 | 3.76E-05 | 3 | rs538777742;rs11720228 | 0.366738 | 0.335012 | 0.332206 | 0.452222 |
| cg08850169 | 3.74E-05 | 1 | rs563239287;rs529151915 | 0.422439 | 0.397408 | 0.404354 | 0.506584 |
| cg15985115 | 3.74E-05 | 17 | rs575662202;rs191354181; rs564849894 | 0.252073 | 0.218491 | 0.214884 | 0.321668 |
| cg15100426 | 3.74E-05 | 2 | rs577976485 | 0.51604 | 0.472158 | 0.481603 | 0.592 |
| cg22954819 | 3.73E-05 | 7 | rs576623379;rs139345911; rs144314898 | 0.359395 | 0.338412 | 0.334398 | 0.407834 |
| cg05255811 | 3.73E-05 | 6 | rs139355049;rs184158937 | 0.336082 | 0.292645 | 0.288639 | 0.418286 |
| cg03910896 | 3.72E-05 | 16 | rs4499236;rs557225519 | 0.645108 | 0.610821 | 0.622347 | 0.701546 |
| cg13601309 | 3.71E-05 | 18 | rs552114487 | 0.383727 | 0.344498 | 0.353699 | 0.494059 |
| cg01431455 | 3.71E-05 | 14 | rs192844162 | 0.457181 | 0.434476 | 0.447946 | 0.526702 |
| cg18191116 | 3.70E-05 | 14 | rs149228290 | 0.622306 | 0.589491 | 0.585006 | 0.684476 |
| cg08853324 | 3.69E-05 | 19 | rs77714197;rs559397580 | 0.319527 | 0.297713 | 0.289661 | 0.385022 |
| cg20995304 | 3.69E-05 | 12 | rs540409457;rs560120680; rs532197316 | 0.385167 | 0.362042 | 0.368311 | 0.451719 |
| cg21964800 | 3.66E-05 | 11 | rs574182432 | 0.394915 | 0.324416 | 0.343416 | 0.490186 |
| cg20921414 | 3.66E-05 | 6 | rs116712669;rs17076665; rs190165957 | 0.412542 | 0.383834 | 0.378966 | 0.490311 |
| cg06536039 | 3.66E-05 | 19 |  | 0.311928 | 0.281002 | 0.274119 | 0.417266 |
| cg08628635 | 3.65E-05 | 6 |  | 0.295163 | 0.259907 | 0.254216 | 0.386394 |
| cg09669125 | 3.64E-05 | 5 | rs139475902;rs529788997; rs149655061 | 0.275539 | 0.247992 | 0.230117 | 0.344402 |
| cg10997669 | 3.64E-05 | 8 | rs73358655;rs559738536; rs533375203 | 0.289349 | 0.263455 | 0.257733 | 0.357164 |
| cg23007592 | 3.63E-05 | 8 | rs191604870 | 0.343659 | 0.31961 | 0.311821 | 0.420434 |
| cg14804531 | 3.63E-05 | 11 | rs572008810;rs540882996; rs563750567;rs577533086; rs543176241 | 0.521641 | 0.495079 | 0.50159 | 0.600977 |
| cg11925568 | 3.61E-05 | 21 |  | 0.28924 | 0.249123 | 0.240936 | 0.37801 |
| cg18159943 | 3.60E-05 | 17 | rs536943029;rs556790617 | 0.333804 | 0.299012 | 0.296788 | 0.412244 |
| cg10127227 | 3.59E-05 | 16 | rs530313354;rs550438675 | 0.166201 | 0.140519 | 0.138735 | 0.215524 |
| cg08324090 | 3.59E-05 | 17 | rs148196938 | 0.383002 | 0.334856 | 0.344802 | 0.475819 |
| cg06819277 | 3.59E-05 | 17 | rs149852502;rs199731812; rs200275851 | 0.311027 | 0.278771 | 0.278505 | 0.395159 |
| cg25800753 | 3.58E-05 | 10 |  | 0.396045 | 0.375999 | 0.377874 | 0.427332 |
| cg13779854 | 3.57E-05 | 3 | rs549049152 | 0.284561 | 0.252563 | 0.245089 | 0.370789 |
| cg19713585 | 3.56E-05 | 16 | rs549654721;rs181279086 | 0.529013 | 0.494179 | 0.503104 | 0.620605 |
| cg02118370 | 3.55E-05 | 6 | rs537200935;rs557680904; rs188281660 | 0.315172 | 0.286394 | 0.280526 | 0.407386 |
| cg06543116 | 3.55E-05 | 17 | rs374443107 | 0.196694 | 0.175332 | 0.181557 | 0.258074 |
| cg19972677 | 3.55E-05 | 12 | rs548676566;rs568787092; rs138431391 | 0.295499 | 0.270562 | 0.27196 | 0.350111 |
| cg06513217 | 3.53E-05 | 22 | rs737851;rs548166500 | 0.576898 | 0.549847 | 0.560678 | 0.642369 |
| cg26893705 | 3.51E-05 | 4 | rs566439723;rs566890861 | 0.34119 | 0.312987 | 0.302622 | 0.423351 |
| cg06095753 | 3.51E-05 | 13 |  | 0.267558 | 0.23757 | 0.22199 | 0.333948 |
| cg19687422 | 3.51E-05 | 5 | rs541700584;rs565273144 | 0.444612 | 0.41889 | 0.413138 | 0.521175 |
| cg03347022 | 3.50E-05 | 7 | rs192120874;rs73395934; rs550823385 | 0.34243 | 0.310393 | 0.304792 | 0.438878 |
| cg22673320 | 3.50E-05 | 22 | rs563094153;rs1883264; rs146476554;rs562340026; rs529423089;rs547531326 | 0.356107 | 0.333425 | 0.320943 | 0.411531 |
| cg09197509 | 3.50E-05 | 7 | rs111514170;rs537521565; rs9918732 | 0.506049 | 0.477904 | 0.476307 | 0.58745 |
| cg03971616 | 3.49E-05 | 17 |  | 0.513892 | 0.492719 | 0.502152 | 0.591204 |
| cg17948174 | 3.49E-05 | 2 | rs546469217 | 0.330036 | 0.289778 | 0.285649 | 0.416337 |
| cg22598533 | 3.48E-05 | 1 |  | 0.259416 | 0.233255 | 0.224614 | 0.325703 |
| cg24692378 | 3.48E-05 | 9 | rs186702756;rs374991237 | 0.457133 | 0.433276 | 0.436057 | 0.511305 |
| cg09597638 | 3.48E-05 | 17 | rs8075429 | 0.430337 | 0.396445 | 0.404975 | 0.495111 |
| cg02675179 | 3.47E-05 | 16 | rs191834843;rs546203470; rs200206868;rs163258; rs111414170;rs149028060 | 0.576856 | 0.549357 | 0.566689 | 0.632829 |
| cg23847017 | 3.46E-05 | 1 | rs537553880 | 0.222628 | 0.196537 | 0.192272 | 0.28311 |
| cg14175330 | 3.46E-05 | 9 | rs529664723;rs76205308; rs542132959 | 0.575559 | 0.551133 | 0.549471 | 0.651415 |
| cg09923296 | 3.45E-05 | 10 | rs11593273;rs548343333; rs567689112 | 0.29044 | 0.26636 | 0.25477 | 0.371918 |
| cg17588094 | 3.43E-05 | 8 | rs184886030;rs145209387; rs555727382 | 0.69734 | 0.676075 | 0.680354 | 0.749101 |
| cg07074766 | 3.43E-05 | 9 | rs138891880;rs61736901 | 0.3787 | 0.354162 | 0.35498 | 0.468608 |
| cg03404346 | 3.43E-05 | 1 | rs572787427 | 0.329374 | 0.302386 | 0.295421 | 0.432408 |
| cg09495519 | 3.42E-05 | 9 | rs561628243 | 0.269704 | 0.247541 | 0.237397 | 0.333144 |
| cg20334877 | 3.42E-05 | 17 |  | 0.28227 | 0.261886 | 0.249219 | 0.3525 |
| cg07914200 | 3.42E-05 | 6 | rs551410639;rs73388408; rs536626754;rs181268584 | 0.419779 | 0.398768 | 0.38867 | 0.472829 |
| cg15897845 | 3.40E-05 | 1 | rs564078470;rs539188677 | 0.183764 | 0.16119 | 0.162816 | 0.23555 |
| cg12401842 | 3.40E-05 | 12 |  | 0.300573 | 0.274191 | 0.272913 | 0.401663 |
| cg02499548 | 3.39E-05 | 1 | rs113274856 | 0.289293 | 0.263645 | 0.251887 | 0.383294 |
| cg03463948 | 3.39E-05 | 11 | rs117526054;rs539658142 | 0.325249 | 0.289136 | 0.28571 | 0.421381 |
| cg05270442 | 3.37E-05 | 2 |  | 0.283728 | 0.261988 | 0.260763 | 0.354434 |
| cg17586302 | 3.36E-05 | 6 | rs191622942;rs561528349 | 0.327504 | 0.282711 | 0.287824 | 0.421541 |
| cg00709073 | 3.33E-05 | 13 | rs76347213;rs183065617; rs543146770 | 0.388923 | 0.36473 | 0.361516 | 0.455026 |
| cg05554161 | 3.27E-05 | 9 | rs558854951;rs142271895; rs144639741 | 0.546856 | 0.522599 | 0.537463 | 0.608943 |
| cg16732654 | 3.24E-05 | 17 | rs547523786 | 0.311475 | 0.282212 | 0.267908 | 0.387228 |
| cg02364450 | 3.21E-05 | 6 | rs187724951;rs192232513 | 0.342926 | 0.314397 | 0.310938 | 0.416205 |
| cg11690666 | 3.18E-05 | 17 |  | 0.395788 | 0.365884 | 0.358389 | 0.511609 |
| cg11718162 | 3.17E-05 | 1 | rs116842438;rs549452838 | 0.473684 | 0.442772 | 0.443491 | 0.548477 |
| cg10472711 | 3.15E-05 | 7 |  | 0.529449 | 0.500169 | 0.517014 | 0.621597 |
| cg05815355 | 3.15E-05 | 5 | rs529468405 | 0.311344 | 0.273844 | 0.26005 | 0.372199 |
| cg08173915 | 3.14E-05 | 21 |  | 0.319335 | 0.291263 | 0.28154 | 0.394517 |
| cg17577640 | 3.11E-05 | 16 |  | 0.272521 | 0.247884 | 0.24281 | 0.337557 |
| cg26679428 | 3.10E-05 | 8 |  | 0.305994 | 0.270636 | 0.282231 | 0.372258 |
| cg09789874 | 3.09E-05 | 12 | rs141557389 | 0.269992 | 0.237458 | 0.239734 | 0.354747 |
| cg14645605 | 3.08E-05 | 4 | rs556794249;rs576904502; rs113237946;rs150320226 | 0.317617 | 0.287369 | 0.279159 | 0.402428 |
| cg24938761 | 3.05E-05 | 19 | rs369496324;rs55924931 | 0.266664 | 0.24086 | 0.228124 | 0.337622 |
| cg22894805 | 3.02E-05 | 15 | rs377390756 | 0.301678 | 0.275759 | 0.268164 | 0.3741 |
| cg16505891 | 3.00E-05 | 14 | rs145950769;rs529338563 | 0.665052 | 0.642437 | 0.652299 | 0.714295 |
| cg25272432 | 3.00E-05 | 4 | rs570057856 | 0.243412 | 0.212463 | 0.213777 | 0.32628 |
| cg17087974 | 3.00E-05 | 11 |  | 0.280236 | 0.25509 | 0.249397 | 0.339398 |
| cg10907086 | 2.97E-05 | 16 | rs556273728;rs576241256; rs544795255 | 0.359398 | 0.33227 | 0.335576 | 0.413946 |
| cg05600164 | 2.96E-05 | 8 | rs527664565;rs542643501; rs183140126 | 0.306324 | 0.270723 | 0.254774 | 0.380685 |
| cg03116466 | 2.96E-05 | 7 | rs186866744;rs73048969; rs535982303 | 0.377957 | 0.343834 | 0.342341 | 0.441878 |
| cg09981464 | 2.95E-05 | 16 | rs144846440;rs576839712 | 0.74876 | 0.714668 | 0.722769 | 0.789463 |
| cg24514600 | 2.95E-05 | 8 | rs4524749 | 0.391367 | 0.358147 | 0.345215 | 0.510577 |
| cg25643932 | 2.94E-05 | 10 | rs563763190 | 0.382369 | 0.360841 | 0.355277 | 0.461493 |
| cg02849689 | 2.90E-05 | 11 |  | 0.290638 | 0.266442 | 0.249097 | 0.389307 |
| cg11032810 | 2.90E-05 | 4 | rs185907990;rs543531962; rs150202884 | 0.643556 | 0.619537 | 0.626927 | 0.67803 |
| cg24598141 | 2.89E-05 | 7 | rs2710806 | 0.407744 | 0.387438 | 0.385346 | 0.472454 |
| cg24553417 | 2.88E-05 | 13 | rs541676169;rs115336807 | 0.337032 | 0.312948 | 0.305273 | 0.430547 |
| cg17825943 | 2.87E-05 | 19 |  | 0.327098 | 0.303124 | 0.29216 | 0.404247 |
| cg06019998 | 2.87E-05 | 17 | rs552363741;rs146380292 | 0.714971 | 0.693246 | 0.708457 | 0.78164 |
| cg03794433 | 2.86E-05 | 15 | rs192783887;rs182497171 | 0.23044 | 0.198085 | 0.193592 | 0.288978 |
| cg17054123 | 2.85E-05 | 14 | rs565458254;rs546745755; rs188823420 | 0.317433 | 0.289248 | 0.281824 | 0.419783 |
| cg00027232 | 2.83E-05 | 2 | rs560044762 | 0.375936 | 0.347256 | 0.348329 | 0.46003 |
| cg01488842 | 2.82E-05 | 1 | rs556199446 | 0.402857 | 0.375532 | 0.382062 | 0.493025 |
| cg24380149 | 2.81E-05 | 1 | rs115451892;rs540445177; rs114489917;rs368034; rs544369547;rs448831; rs147717692 | 0.621583 | 0.597015 | 0.605349 | 0.669117 |
| cg15656693 | 2.80E-05 | 3 |  | 0.382858 | 0.358376 | 0.360837 | 0.474228 |
| cg05710391 | 2.77E-05 | 4 | rs551294513;rs571458114; rs537240191 | 0.393588 | 0.364671 | 0.375821 | 0.472784 |
| cg16460860 | 2.76E-05 | 17 | rs540218419 | 0.428639 | 0.403181 | 0.408443 | 0.506998 |
| cg06816685 | 2.73E-05 | 17 |  | 0.401759 | 0.381018 | 0.387191 | 0.458346 |
| cg23586766 | 2.72E-05 | 2 |  | 0.309431 | 0.274447 | 0.276957 | 0.400585 |
| cg24678320 | 2.72E-05 | 19 | rs546697679;rs12976023; rs73593697 | 0.654434 | 0.632407 | 0.64311 | 0.729383 |
| cg16905966 | 2.71E-05 | 9 | rs535471732;rs555325706 | 0.34787 | 0.326898 | 0.333006 | 0.413957 |
| cg20948817 | 2.71E-05 | 2 |  | 0.277524 | 0.257116 | 0.243378 | 0.348232 |
| cg16998950 | 2.71E-05 | 5 | rs552921245 | 0.301733 | 0.255791 | 0.250885 | 0.385708 |
| cg06944141 | 2.70E-05 | 19 | rs116680652;rs114500766; rs529918357;rs4578780 | 0.329149 | 0.307263 | 0.308428 | 0.408887 |
| cg02579959 | 2.69E-05 | 15 | rs116193392 | 0.33798 | 0.305414 | 0.285972 | 0.430888 |
| cg05461268 | 2.69E-05 | 2 | rs376589784 | 0.351824 | 0.314385 | 0.303057 | 0.447809 |
| cg26387807 | 2.69E-05 | 7 | rs575049645 | 0.307575 | 0.285444 | 0.277182 | 0.391016 |
| cg25303556 | 2.68E-05 | 22 |  | 0.246727 | 0.220328 | 0.208857 | 0.316014 |
| cg03601962 | 2.68E-05 | 2 | rs78497933;rs555239562 | 0.285079 | 0.255737 | 0.239386 | 0.379663 |
| cg16463452 | 2.68E-05 | 13 | rs138779088 | 0.30883 | 0.281532 | 0.278939 | 0.391287 |
| cg23246961 | 2.67E-05 | 16 | rs147122919;rs192138129; rs184910103 | 0.570071 | 0.54539 | 0.545145 | 0.617076 |
| cg18138635 | 2.66E-05 | 8 | rs183286209;rs548911031 | 0.292044 | 0.263539 | 0.245571 | 0.367128 |
| cg14240440 | 2.65E-05 | 8 | rs564144353;rs528055397 | 0.274014 | 0.240074 | 0.244473 | 0.359947 |
| cg09857383 | 2.64E-05 | 13 |  | 0.300558 | 0.271271 | 0.266113 | 0.375852 |
| cg13814779 | 2.64E-05 | 16 | rs186074778;rs559937942; rs114088996 | 0.341666 | 0.315478 | 0.306305 | 0.427878 |
| cg24299175 | 2.63E-05 | 9 | rs142518974;rs537115742 | 0.274243 | 0.244564 | 0.250345 | 0.362414 |
| cg19230867 | 2.60E-05 | 3 | rs550893234;rs148895894; rs143246657 | 0.501643 | 0.471056 | 0.470008 | 0.566603 |
| cg13733708 | 2.59E-05 | 20 | rs543192143 | 0.351769 | 0.324755 | 0.323359 | 0.430802 |
| cg09306188 | 2.58E-05 | 16 | rs61734739;rs200609470; rs200074494;rs78699679; rs201334591;rs536507451 | 0.552392 | 0.531528 | 0.523368 | 0.646003 |
| cg25046649 | 2.57E-05 | 12 | rs528641294;rs542416887; rs191888279 | 0.360061 | 0.328584 | 0.342444 | 0.447961 |
| cg08959230 | 2.56E-05 | 16 | rs34843513;rs199713417; rs139673904 | 0.610555 | 0.581622 | 0.594102 | 0.656163 |
| cg04751885 | 2.55E-05 | 21 | rs527388414;rs73906728; rs567387953;rs150618458 | 0.299565 | 0.267691 | 0.262168 | 0.373579 |
| cg10908105 | 2.54E-05 | 10 | rs142980528;rs569386960 | 0.320575 | 0.288392 | 0.282069 | 0.394977 |
| cg17454247 | 2.52E-05 | 5 | rs140810920 | 0.377443 | 0.354178 | 0.362276 | 0.436769 |
| cg07460883 | 2.51E-05 | 11 |  | 0.310448 | 0.285768 | 0.281888 | 0.389618 |
| cg25002435 | 2.50E-05 | 5 | rs186208134 | 0.288999 | 0.261171 | 0.257939 | 0.363112 |
| cg09366951 | 2.49E-05 | 12 | rs16913870;rs576668971 | 0.23002 | 0.206969 | 0.199547 | 0.313352 |
| cg13876590 | 2.48E-05 | 11 | rs574562133;rs541991064 | 0.332121 | 0.303495 | 0.291195 | 0.413051 |
| cg02460791 | 2.46E-05 | 6 | rs529680637 | 0.336591 | 0.310255 | 0.310164 | 0.416931 |
| cg16851369 | 2.45E-05 | 9 | rs531977826;rs551874350; rs565388707 | 0.292692 | 0.260683 | 0.253028 | 0.395275 |
| cg05600851 | 2.45E-05 | 6 |  | 0.292138 | 0.262341 | 0.253048 | 0.392628 |
| cg21317441 | 2.41E-05 | 13 | rs572736187 | 0.343157 | 0.319232 | 0.318194 | 0.422699 |
| cg03618113 | 2.40E-05 | 12 | rs570381279;rs148643627 | 0.389365 | 0.357204 | 0.344756 | NA |
| cg22904406 | 2.40E-05 | 6 | rs1059231;rs61737694 | 0.539153 | 0.515389 | 0.530232 | 0.627211 |
| cg24244935 | 2.37E-05 | 1 | rs564204889;rs531451452; rs148809414 | 0.317453 | 0.296379 | 0.29371 | 0.380053 |
| cg00173498 | 2.36E-05 | 8 |  | 0.402348 | 0.375998 | 0.385116 | 0.450612 |
| cg01402665 | 2.36E-05 | 16 | rs555491051;rs538847379 | 0.294976 | 0.268252 | 0.258281 | 0.363119 |
| cg01618151 | 2.36E-05 | 6 | rs559284763;rs115290040 | 0.325628 | 0.303177 | 0.291234 | 0.387318 |
| cg09118771 | 2.35E-05 | 22 | rs540559278;rs35413610; rs528909959 | 0.302202 | 0.278622 | 0.260079 | 0.401587 |
| cg04765471 | 2.32E-05 | 10 | rs539874665;rs373430506 | 0.58896 | 0.565782 | 0.572245 | 0.660148 |
| cg00502926 | 2.31E-05 | 14 | rs574242545;rs543243191; rs185541436;rs79236586 | 0.316112 | 0.291713 | 0.300453 | 0.385765 |
| cg14716166 | 2.30E-05 | 15 | rs531690992;rs6492999; rs139152236 | 0.352558 | 0.32409 | 0.320178 | 0.465051 |
| cg08973191 | 2.28E-05 | 8 | rs555148335;rs200137501; rs543135484;rs558207698 | 0.413905 | 0.388485 | 0.397498 | 0.524203 |
| cg20001791 | 2.26E-05 | 6 | rs9383138;rs541486552; rs561240891 | 0.220296 | 0.199937 | 0.199754 | 0.307977 |
| cg16333793 | 2.26E-05 | 21 | rs191731609;rs181817692; rs561775117 | 0.241168 | 0.217588 | 0.220211 | 0.313684 |
| cg25990696 | 2.25E-05 | 13 | rs569074015;rs537995906 | 0.538731 | 0.501259 | 0.494099 | 0.633871 |
| cg25402049 | 2.25E-05 | 1 | rs557663139;rs559169011 | 0.658082 | 0.632391 | 0.650766 | 0.727763 |
| cg16478897 | 2.22E-05 | 16 |  | 0.328135 | 0.307981 | 0.293383 | 0.397379 |
| cg10815470 | 2.21E-05 | 6 | rs542651343;rs560816830 | 0.583351 | 0.558896 | 0.560245 | 0.649503 |
| cg18377866 | 2.19E-05 | 3 | rs543093162 | 0.722293 | 0.679886 | 0.69678 | 0.786618 |
| cg19636224 | 2.18E-05 | 1 |  | 0.315282 | 0.295259 | 0.292388 | 0.414666 |
| cg02464519 | 2.18E-05 | 15 | rs187141593;rs191636166; rs569048381 | 0.603025 | 0.571258 | 0.570952 | 0.67522 |
| cg20537531 | 2.16E-05 | 15 | rs191197283;rs569851075; rs150479031 | 0.308547 | 0.275484 | 0.273153 | 0.402304 |
| cg12993663 | 2.13E-05 | 11 | rs188946243;rs534395430 | 0.517286 | 0.476052 | 0.473306 | 0.578494 |
| cg01593812 | 2.12E-05 | 21 |  | 0.286644 | 0.256716 | 0.241552 | 0.336563 |
| cg11559388 | 2.11E-05 | 15 | rs142713129;rs551523944 | 0.304982 | 0.265745 | 0.264215 | 0.399731 |
| cg25949447 | 2.11E-05 | 2 | rs556637404;rs573307414; rs535805440 | 0.327919 | 0.289136 | 0.284167 | 0.412651 |
| cg06503571 | 2.10E-05 | 7 |  | 0.42905 | 0.406485 | 0.42278 | 0.504947 |
| cg18009691 | 2.09E-05 | 20 | rs535751462;rs113226127 | 0.227017 | 0.201649 | 0.2079 | 0.265694 |
| cg04311811 | 2.08E-05 | 20 | rs530236001;rs192334778; rs6095651 | 0.43159 | 0.410221 | 0.423758 | 0.525335 |
| cg21754524 | 2.08E-05 | 6 |  | 0.186632 | 0.163172 | 0.164545 | 0.243563 |
| cg00867317 | 2.07E-05 | 20 | rs557497245;rs11905493; rs75778408;rs558177369; rs6110189 | 0.259842 | 0.22363 | 0.224043 | 0.320267 |
| cg18803843 | 2.05E-05 | 2 | rs143365044;rs181741837; rs186400361 | 0.287493 | 0.259956 | 0.260586 | 0.356551 |
| cg21461811 | 2.05E-05 | 10 | rs562543259 | 0.522888 | 0.499142 | 0.507835 | 0.609072 |
| cg21881184 | 2.01E-05 | 19 | rs139660333;rs528659596 | 0.792637 | 0.764918 | 0.77852 | 0.842241 |
| cg01261013 | 1.98E-05 | 21 | rs529479252 | 0.350768 | 0.327012 | 0.338916 | 0.45301 |
| cg10907896 | 1.98E-05 | 2 | rs115167492;rs544994250 | 0.475736 | 0.454554 | 0.447856 | 0.583414 |
| cg01607346 | 1.98E-05 | 14 | rs73290660;rs192968963; rs113591217;rs75316294 | 0.322864 | 0.287185 | 0.276601 | 0.434404 |
| cg11462267 | 1.96E-05 | 9 | rs143643563;rs541669892; rs76531699;rs140545795; rs546659944;rs150044596 | 0.365484 | 0.321483 | 0.312674 | 0.449052 |
| cg00532633 | 1.96E-05 | 16 | rs536022898 | 0.342772 | 0.309236 | 0.317156 | 0.391277 |
| cg02355304 | 1.93E-05 | 7 | rs544503858;rs138023232; rs192049463 | 0.590009 | 0.557802 | 0.56961 | 0.643755 |
| cg10938213 | 1.93E-05 | 5 |  | 0.478525 | 0.440391 | 0.45037 | 0.542258 |
| cg00444239 | 1.92E-05 | 8 | rs140356799;rs558780330; rs368712012 | 0.422136 | 0.396072 | 0.405905 | 0.503773 |
| cg26706803 | 1.91E-05 | 16 |  | 0.205235 | 0.16521 | 0.182019 | 0.265728 |
| cg01804557 | 1.89E-05 | 15 | rs187402542;rs191824015; rs538424803 | 0.274684 | 0.246581 | 0.240225 | 0.370194 |
| cg08467103 | 1.88E-05 | 2 | rs117820224;rs577925628; rs181701528 | 0.326216 | 0.301869 | 0.301139 | 0.426496 |
| cg22695440 | 1.86E-05 | 4 |  | 0.496002 | 0.470727 | 0.488298 | 0.578592 |
| cg19409533 | 1.84E-05 | 7 | rs537826341;rs191530288; rs149842901 | 0.423948 | 0.398312 | 0.4106 | 0.497217 |
| cg22961402 | 1.80E-05 | 17 | rs148369444;rs3115094; rs555642557 | 0.489205 | 0.459434 | 0.464748 | 0.552774 |
| cg04256521 | 1.79E-05 | 22 |  | 0.233384 | 0.203941 | 0.190729 | 0.331018 |
| cg09024685 | 1.78E-05 | 15 | rs79528487;rs189480736; rs113237975;rs527966887 | 0.304373 | 0.283366 | 0.283126 | 0.390132 |
| cg11862131 | 1.76E-05 | 6 | rs4960263;rs546243519; rs149750518 | 0.294327 | 0.273245 | 0.265163 | 0.395778 |
| cg03655330 | 1.75E-05 | 3 | rs150976767;rs587729250; rs182987423 | 0.167115 | 0.142395 | 0.148152 | 0.209182 |
| cg14151003 | 1.75E-05 | 16 | rs555592115 | 0.196273 | 0.16931 | 0.17194 | 0.259468 |
| cg15951935 | 1.74E-05 | 10 | rs551349833;rs570957778; rs6480909 | 0.268258 | 0.23429 | 0.220095 | 0.34752 |
| cg24663338 | 1.72E-05 | 1 | rs79255428;rs528108420; rs546166480;rs567860442 | 0.313468 | 0.28279 | 0.272852 | 0.407435 |
| cg14750332 | 1.72E-05 | 2 | rs190897805 | 0.202684 | 0.173617 | 0.175144 | 0.251046 |
| cg20453370 | 1.71E-05 | 3 | rs571552489;rs539120508; rs557215026 | 0.327239 | 0.28393 | 0.290806 | 0.428153 |
| cg26296479 | 1.70E-05 | 8 | rs141093234;rs151293772; rs566248747 | 0.492377 | 0.471441 | 0.481109 | 0.541388 |
| cg23587176 | 1.69E-05 | 7 | rs187984435 | 0.401299 | 0.361375 | 0.373882 | 0.492539 |
| cg11909142 | 1.68E-05 | 20 | rs144542988;rs6045440; rs139419360;rs200836567; rs145142114;rs147576961 | 0.3213 | 0.287237 | 0.279005 | 0.392478 |
| cg20118717 | 1.68E-05 | 6 | rs544817923 | 0.319999 | 0.296689 | 0.310284 | 0.393164 |
| cg24908617 | 1.67E-05 | 2 | rs561329384;rs543890525 | 0.361466 | 0.334521 | 0.329768 | 0.446154 |
| cg01185443 | 1.66E-05 | 6 |  | 0.407526 | 0.38461 | 0.395849 | 0.4841 |
| cg02739429 | 1.65E-05 | 1 | rs548147872;rs183323298; rs72724161 | 0.324988 | 0.291627 | 0.278708 | 0.385582 |
| cg26308793 | 1.63E-05 | 1 | rs564269002 | 0.426031 | 0.394734 | 0.411258 | 0.509057 |
| cg01223600 | 1.60E-05 | 19 | rs544378837 | 0.398107 | 0.372136 | 0.367891 | 0.472591 |
| cg22282916 | 1.59E-05 | 22 | rs146870755;rs148300715 | 0.424708 | 0.397311 | 0.400871 | 0.525715 |
| cg06605331 | 1.58E-05 | 12 | rs113563079;rs576634677 | 0.594114 | 0.568561 | 0.579984 | 0.646834 |
| cg10741887 | 1.58E-05 | 17 | rs148715410;rs200152612 | 0.294023 | 0.261994 | 0.245273 | 0.367825 |
| cg19964040 | 1.57E-05 | 2 |  | 0.262625 | 0.237064 | 0.236777 | 0.330036 |
| cg01572694 | 1.57E-05 | 17 | rs545359920;rs150632354 | 0.435175 | 0.38274 | 0.368009 | NA |
| cg26343834 | 1.57E-05 | 2 | rs536299562;rs554664305; rs190790664;rs540857551; rs558965594 | 0.336737 | 0.301948 | 0.29448 | 0.428768 |
| cg01666088 | 1.55E-05 | 8 | rs564210072;rs76777587; rs147257596 | 0.537971 | 0.510638 | 0.528999 | 0.593636 |
| cg26465402 | 1.55E-05 | 5 | rs545459496;rs545689837; rs565479104;rs527284749; rs540651985 | 0.329259 | 0.297476 | 0.289526 | 0.431884 |
| cg20759375 | 1.55E-05 | 12 | rs556777581;rs73372845; rs538759579 | 0.273869 | 0.24686 | 0.239982 | 0.362756 |
| cg25401083 | 1.55E-05 | 16 |  | 0.220489 | 0.194138 | 0.199004 | 0.28092 |
| cg04188723 | 1.51E-05 | 15 | rs368299293 | 0.32566 | 0.29158 | 0.294878 | 0.403277 |
| cg16778107 | 1.51E-05 | 16 | rs570820154;rs534836199; rs117966223 | 0.27725 | 0.242491 | 0.243251 | 0.36397 |
| cg18849308 | 1.49E-05 | 3 | rs563523794;rs74433277 | 0.501811 | 0.480426 | 0.495957 | 0.571896 |
| cg19839282 | 1.49E-05 | 22 | rs544131426 | 0.316162 | 0.287911 | 0.28132 | 0.428387 |
| cg01339381 | 1.48E-05 | 11 | rs555954425 | 0.528966 | 0.492087 | 0.495412 | 0.596619 |
| cg03202617 | 1.48E-05 | 16 | rs149726924 | 0.293181 | 0.257285 | 0.254038 | 0.366063 |
| cg25365401 | 1.47E-05 | 5 | rs182444087;rs147568634; rs531605569;rs551408458; rs571221117;rs533874064 | 0.267257 | 0.238709 | 0.234411 | 0.318067 |
| cg20180364 | 1.46E-05 | 10 | rs185220612 | 0.243166 | 0.222484 | 0.210248 | 0.313483 |
| cg03577479 | 1.45E-05 | 5 |  | 0.31398 | 0.286208 | 0.285242 | 0.412046 |
| cg03211711 | 1.43E-05 | 2 | rs420225;rs557042044 | 0.292501 | 0.262975 | 0.254004 | 0.393603 |
| cg10981439 | 1.43E-05 | 6 | rs182934519;rs115951667; rs533482664 | 0.301766 | 0.255398 | 0.25957 | 0.375214 |
| cg02827175 | 1.41E-05 | 7 | rs185588798;rs534276086 | 0.445539 | 0.416152 | 0.413847 | 0.56022 |
| cg00754604 | 1.41E-05 | 2 | rs549820721 | 0.373027 | 0.347044 | 0.356197 | 0.460577 |
| cg12110801 | 1.40E-05 | 19 | rs528139307;rs551462559; rs571305916 | 0.31221 | 0.2738 | 0.281111 | 0.384848 |
| cg10458216 | 1.39E-05 | 13 | rs3809361;rs182891364 | 0.250912 | 0.223554 | 0.225363 | 0.329359 |
| cg03486096 | 1.38E-05 | 1 | rs543907811;rs192034498; rs115484949 | 0.344523 | 0.310569 | 0.308118 | 0.428074 |
| cg14123700 | 1.37E-05 | 3 |  | 0.358439 | 0.333315 | 0.335479 | 0.456777 |
| cg06441457 | 1.36E-05 | 1 | rs395859;rs395847 | 0.411432 | 0.369346 | 0.367932 | 0.525589 |
| cg15742245 | 1.35E-05 | 19 | rs541797815 | 0.588689 | 0.564608 | 0.582682 | 0.677248 |
| cg11644454 | 1.35E-05 | 1 | rs528199566;rs546655739 | 0.285608 | 0.250722 | 0.245852 | 0.360568 |
| cg14505810 | 1.34E-05 | 9 | rs547010157;rs566834416; rs535855157 | 0.300317 | 0.260587 | 0.257736 | 0.37973 |
| cg10052599 | 1.31E-05 | 2 | rs558520809 | 0.379177 | 0.349693 | 0.345617 | 0.454135 |
| cg21374307 | 1.31E-05 | 3 | rs532681676;rs13093643; rs558943995 | 0.292923 | 0.249835 | 0.248091 | 0.381948 |
| cg03650870 | 1.30E-05 | 19 | rs147680903;rs529324369; rs547565283 | 0.45689 | 0.429729 | 0.446023 | 0.527024 |
| cg24522277 | 1.30E-05 | 2 |  | 0.515009 | 0.493086 | 0.511636 | 0.563083 |
| cg14285150 | 1.29E-05 | 17 |  | 0.31738 | 0.28679 | 0.288162 | 0.369849 |
| cg02398240 | 1.29E-05 | 2 | rs143963309;rs186724689 | 0.374868 | 0.348943 | 0.355664 | 0.467293 |
| cg13074526 | 1.29E-05 | 17 | rs548218389;rs561520769 | 0.443368 | 0.403407 | 0.419916 | 0.514284 |
| cg19557860 | 1.28E-05 | 16 | rs12447543;rs548574000 | 0.267672 | 0.229181 | 0.226768 | 0.336773 |
| cg23348269 | 1.26E-05 | 20 | rs568584234;rs141820391 | 0.448557 | 0.427636 | 0.427975 | 0.540649 |
| cg16565031 | 1.26E-05 | 14 | rs575323088;rs542241405; rs560903290 | 0.291328 | 0.263451 | 0.258161 | 0.359151 |
| cg16276850 | 1.25E-05 | 17 |  | 0.319854 | 0.287117 | 0.288607 | 0.3812 |
| cg08888152 | 1.25E-05 | 17 | rs563216673 | 0.327576 | 0.293017 | 0.312461 | 0.448663 |
| cg10206388 | 1.25E-05 | 22 | rs537143049;rs557212759 | 0.259823 | 0.226913 | 0.222809 | 0.326759 |
| cg10905610 | 1.23E-05 | 6 | rs186007527;rs569327753 | 0.300323 | 0.267271 | 0.26699 | 0.368161 |
| cg01650347 | 1.22E-05 | 3 |  | 0.300579 | 0.266547 | 0.254101 | 0.397694 |
| cg08257722 | 1.21E-05 | 20 | rs549298408;rs6064392; rs6127743;rs370799989 | 0.287805 | 0.258335 | 0.249767 | 0.363035 |
| cg02663635 | 1.21E-05 | 12 |  | 0.285456 | 0.263736 | 0.245912 | 0.356716 |
| cg11061419 | 1.20E-05 | 20 | rs2904376;rs562551884 | 0.449463 | 0.418879 | 0.435243 | 0.559172 |
| cg22813992 | 1.18E-05 | 11 | rs564865606 | 0.403824 | 0.382535 | 0.385289 | 0.505681 |
| cg15177986 | 1.18E-05 | 12 | rs140924927 | 0.31338 | 0.280071 | 0.277183 | 0.405301 |
| cg12019801 | 1.18E-05 | 18 | rs556627902;rs576350325; rs543850383;rs117145635; rs73975413;rs141520495 | 0.275087 | 0.239919 | 0.234962 | 0.347982 |
| cg09224040 | 1.18E-05 | 6 | rs190936757;rs183205708 | 0.228316 | 0.202333 | 0.20938 | 0.320172 |
| cg13365436 | 1.18E-05 | 11 | rs539465457 | 0.52583 | 0.500637 | 0.518934 | 0.605059 |
| cg26262644 | 1.18E-05 | 15 | rs572138582 | 0.442338 | 0.412929 | 0.41609 | 0.503438 |
| cg21076170 | 1.17E-05 | 6 | rs560818786;rs11453604; rs529678955 | 0.321383 | 0.290475 | 0.298679 | 0.394766 |
| cg16332119 | 1.15E-05 | 3 | rs569149545;rs539687206 | 0.352398 | 0.31233 | 0.318171 | 0.426985 |
| cg06257058 | 1.15E-05 | 7 | rs539109497;rs143334598 | 0.308752 | 0.268791 | 0.2607 | 0.389217 |
| cg08900409 | 1.14E-05 | 19 | rs532612910;rs535442525; rs548771628 | 0.313544 | 0.292969 | 0.278561 | 0.388224 |
| cg16283183 | 1.14E-05 | 3 | rs575169974;rs545670504 | 0.259844 | 0.237479 | 0.219177 | 0.323745 |
| cg03510269 | 1.13E-05 | 5 | rs536135918;rs556040330; rs148785562;rs2731686; rs142457937;rs572085187; rs79967046 | 0.608019 | 0.581416 | 0.582187 | 0.670394 |
| cg24575128 | 1.12E-05 | 3 | rs537272477 | 0.298271 | 0.277365 | 0.270376 | 0.377013 |
| cg19028706 | 1.11E-05 | 3 | rs564073545;rs532775473 | 0.324667 | 0.291724 | 0.30235 | 0.384433 |
| cg15080495 | 1.11E-05 | 6 | rs151332685 | 0.296166 | 0.267192 | 0.254396 | 0.360315 |
| cg07487971 | 1.09E-05 | 19 | rs552931979;rs571142299; rs538456934;rs187211851 | 0.294982 | 0.266677 | 0.256728 | 0.372147 |
| cg01620416 | 1.08E-05 | 20 | rs555702985;rs574348355 | 0.306889 | 0.285891 | 0.27982 | 0.384465 |
| cg19683419 | 1.06E-05 | 3 |  | 0.361096 | 0.332879 | 0.337794 | 0.440273 |
| cg14628914 | 1.04E-05 | 6 | rs575204310;rs542681832; rs552985122 | 0.390036 | 0.3579 | 0.364698 | 0.471718 |
| cg18698096 | 1.04E-05 | 14 | rs140953418;rs554962529; rs535480819;rs75307401; rs544098495 | 0.190116 | 0.153782 | 0.166539 | 0.257515 |
| cg26126272 | 1.03E-05 | 16 |  | 0.324383 | 0.288184 | 0.290322 | 0.420767 |
| cg00733441 | 1.02E-05 | 11 |  | 0.534661 | 0.508027 | 0.512197 | 0.595644 |
| cg15430294 | 1.02E-05 | 19 | rs144173097;rs117339508; rs375921319 | 0.414976 | 0.386538 | 0.382325 | 0.455078 |
| cg07074527 | 1.02E-05 | 3 |  | 0.343367 | 0.318032 | 0.327147 | 0.402736 |
| cg22270609 | 1.02E-05 | 16 | rs575430501 | 0.476164 | 0.451025 | 0.466989 | 0.533945 |
| cg12771446 | 9.94E-06 | 1 | rs115162991;rs139332483 | 0.275405 | 0.253287 | 0.250628 | 0.332229 |
| cg24905779 | 9.65E-06 | 12 | rs551332740;rs73072465; rs114215438 | 0.450608 | 0.417276 | 0.422929 | 0.520547 |
| cg05575594 | 9.53E-06 | 20 | rs144542988;rs6045440; rs139419360;rs200836567; rs145142114;rs147576961 | 0.352448 | 0.327536 | 0.315488 | 0.442274 |
| cg05030067 | 9.15E-06 | 14 | rs541198858 | 0.264583 | 0.233798 | 0.226192 | 0.348458 |
| cg19232929 | 9.11E-06 | 12 | rs572224332;rs541112287; rs73111518;rs186720832; rs142200275;rs563444079 | 0.804731 | 0.770919 | 0.758281 | 0.843425 |
| cg00314247 | 8.98E-06 | 17 | rs565862397;rs534954603; rs554860268 | 0.348881 | 0.302438 | 0.317256 | 0.452049 |
| cg11814087 | 8.89E-06 | 5 | rs533569072;rs181063495; rs567396070;rs139499239 | 0.319127 | 0.294207 | 0.285566 | 0.414624 |
| cg02853638 | 8.89E-06 | 3 |  | 0.323451 | 0.287667 | 0.275897 | 0.409823 |
| cg23335299 | 8.85E-06 | 15 |  | 0.446522 | 0.407676 | 0.415463 | 0.51036 |
| cg02850815 | 8.84E-06 | 8 | rs539503501;rs557877222 | 0.449034 | 0.422055 | 0.428093 | 0.506233 |
| cg18887229 | 8.81E-06 | 5 | rs180711754 | 0.512811 | 0.483392 | 0.46682 | 0.583573 |
| cg04391950 | 8.81E-06 | 5 | rs201501612 | 0.336091 | 0.303689 | 0.313743 | 0.413656 |
| cg13175344 | 8.78E-06 | 20 | rs571943043;rs6053027 | 0.547415 | 0.524932 | 0.540382 | 0.612056 |
| cg17908151 | 8.64E-06 | 7 |  | 0.358352 | 0.33569 | 0.344726 | 0.434744 |
| cg02823975 | 8.57E-06 | 2 | rs188378781 | 0.413319 | 0.393202 | 0.396067 | 0.470024 |
| cg06408466 | 8.57E-06 | 19 | rs528254132;rs10409433 | 0.322055 | 0.300623 | 0.306135 | 0.374648 |
| cg20500453 | 8.54E-06 | 20 | rs73123219;rs145422399; rs538302406 | 0.563798 | 0.538392 | 0.547061 | 0.655119 |
| cg23991393 | 8.47E-06 | 8 | rs529551475 | 0.281285 | 0.257815 | 0.258728 | 0.338707 |
| cg16441262 | 8.46E-06 | 15 | rs138768257;rs77182755; rs74025645 | 0.451031 | 0.411578 | 0.431367 | 0.542653 |
| cg08130060 | 8.45E-06 | 17 | rs561786081;rs181261474; rs184936257 | 0.39921 | 0.365652 | 0.365779 | 0.488243 |
| cg10926282 | 8.33E-06 | 12 | rs190374959 | 0.256309 | 0.227041 | 0.219618 | 0.345759 |
| cg04481650 | 8.26E-06 | 17 | rs8067774 | 0.347265 | 0.317287 | 0.312493 | 0.4301 |
| cg18545100 | 8.20E-06 | 20 | rs562870699;rs117812664; rs13433178;rs548307709 | 0.2757 | 0.244565 | 0.232337 | 0.339664 |
| cg04903759 | 8.18E-06 | 3 |  | 0.302813 | 0.269079 | 0.262082 | 0.385824 |
| cg05488465 | 8.16E-06 | 11 | rs535309491;rs147853708 | 0.345331 | 0.319501 | 0.312089 | 0.430466 |
| cg06437891 | 8.12E-06 | 19 | rs536800600;rs150408410; rs112847472;rs79208384 | 0.505234 | 0.484277 | 0.489832 | 0.563993 |
| cg24211550 | 8.03E-06 | 17 | rs533585422;rs552078524 | 0.335554 | 0.281325 | 0.281905 | 0.3782 |
| cg07426937 | 7.98E-06 | 22 | rs536242345;rs554947211 | 0.698315 | 0.6667 | 0.684492 | 0.739258 |
| cg18998543 | 7.97E-06 | 7 |  | 0.337174 | 0.310212 | 0.319636 | 0.390894 |
| cg16814786 | 7.72E-06 | 10 | rs182379287;rs541789969; rs186700566 | 0.251237 | 0.221902 | 0.221012 | 0.327731 |
| cg08820841 | 7.08E-06 | 3 | rs182089148 | 0.28925 | 0.246695 | 0.254177 | 0.395229 |
| cg09031737 | 7.06E-06 | 6 |  | 0.211312 | 0.187437 | 0.18636 | 0.277653 |
| cg16125109 | 7.02E-06 | 20 | rs79297897 | 0.493023 | 0.460769 | 0.471109 | 0.554971 |
| cg16665098 | 6.99E-06 | 9 |  | 0.529024 | 0.499272 | 0.512439 | 0.589077 |
| cg19502359 | 6.96E-06 | 11 | rs548951584;rs565940992 | 0.308824 | 0.283945 | 0.277539 | 0.379405 |
| cg24010274 | 6.76E-06 | 10 | rs575405822 | 0.45653 | 0.431682 | 0.437812 | 0.513433 |
| cg23039250 | 6.67E-06 | 1 | rs5744147;rs575095022 | 0.549916 | 0.518178 | 0.528325 | 0.629581 |
| cg15128785 | 6.65E-06 | 22 | rs529300072;rs140644998 | 0.582167 | 0.558717 | 0.544422 | 0.63326 |
| cg12744859 | 6.65E-06 | 17 | rs113520215;rs377767191; rs57183284;rs547963863; rs568039277 | 0.209189 | 0.170635 | 0.1622 | 0.263898 |
| cg01819502 | 6.63E-06 | 17 | rs566047656;rs117614879; rs556713028;rs573532825; rs536054968 | 0.763895 | 0.737054 | 0.749019 | 0.802577 |
| cg01357222 | 6.63E-06 | 6 | rs555683154;rs572385230; rs78437912;rs557789652; rs191979972; rs543142504 | 0.300577 | 0.274065 | 0.269289 | 0.3743 |
| cg11338116 | 6.49E-06 | 1 | rs375232486 | 0.332401 | 0.312359 | 0.310498 | 0.423423 |
| cg18377381 | 6.47E-06 | 22 |  | 0.324489 | 0.301095 | 0.298326 | 0.376235 |
| cg25960038 | 6.37E-06 | 17 | rs536646514 | 0.239958 | 0.218273 | 0.219462 | 0.302149 |
| cg25107997 | 6.35E-06 | 3 | rs534920778;rs547426726 | 0.348961 | 0.317794 | 0.320663 | 0.416695 |
| cg07474797 | 6.30E-06 | 16 | rs569547495;rs28733667; rs531832862 | 0.326394 | 0.306124 | 0.315847 | 0.396834 |
| cg16315116 | 6.29E-06 | 3 | rs190373225;rs139446831 | 0.371391 | 0.347923 | 0.351982 | 0.430855 |
| cg00138407 | 6.24E-06 | 3 | rs547395055 | 0.480789 | 0.451342 | 0.452289 | 0.544289 |
| cg20766052 | 6.17E-06 | 6 | rs546686299 | 0.143827 | 0.122931 | 0.107598 | 0.17779 |
| cg18445680 | 6.17E-06 | 2 | rs142130997 | 0.451672 | 0.428279 | 0.427313 | 0.554962 |
| cg11610703 | 6.12E-06 | 8 | rs145209387;rs555727382 | 0.368144 | 0.337633 | 0.341084 | 0.440536 |
| cg26547816 | 6.02E-06 | 5 | rs77104676;rs543029100; rs142607553 | 0.413608 | 0.392596 | 0.39977 | 0.50136 |
| cg21148428 | 5.94E-06 | 20 | rs184674792 | 0.268914 | 0.243825 | 0.240101 | 0.337301 |
| cg23803230 | 5.92E-06 | 20 | rs144535608;rs574888891; rs139594013 | 0.261373 | 0.232539 | 0.23188 | 0.343041 |
| cg05348816 | 5.87E-06 | 22 | rs182637012;rs576049499 | 0.367714 | 0.333546 | 0.340961 | 0.462705 |
| cg23893833 | 5.87E-06 | 2 | rs113837852 | 0.784769 | 0.763519 | 0.778795 | 0.816897 |
| cg18635314 | 5.85E-06 | 2 | rs559228319 | 0.34069 | 0.303458 | 0.303544 | 0.423753 |
| cg12690186 | 5.75E-06 | 1 | rs568088850;rs527246781 | 0.325675 | 0.294792 | 0.283721 | 0.407952 |
| cg08909938 | 5.73E-06 | 12 | rs113313723;rs146991784; rs568215771 | 0.316717 | 0.286574 | 0.282635 | 0.418885 |
| cg04871472 | 5.73E-06 | 2 | rs111635818;rs184383585 | 0.304165 | 0.283909 | 0.273582 | 0.385632 |
| cg19023589 | 5.68E-06 | 13 | rs539364795;rs558503589 | 0.343632 | 0.306543 | 0.293294 | 0.459639 |
| cg07786372 | 5.61E-06 | 12 | rs552947874;rs529235556 | 0.286457 | 0.254693 | 0.245582 | 0.392136 |
| cg19145477 | 5.61E-06 | 17 | rs187915999 | 0.249045 | 0.214852 | 0.220247 | 0.322972 |
| cg17285259 | 5.60E-06 | 1 | rs142199307;rs546936456; rs566759142;rs534869727; rs144460481;rs148421744 | 0.371614 | 0.350804 | 0.345716 | 0.443715 |
| cg02774666 | 5.60E-06 | 8 | rs182953718;rs144708917 | 0.290454 | 0.264 | 0.25865 | 0.375063 |
| cg06918740 | 5.51E-06 | 19 | rs573911069;rs542865970 | 0.411378 | 0.375316 | 0.364317 | 0.488687 |
| cg14508198 | 5.44E-06 | 3 |  | 0.286938 | 0.259547 | 0.256778 | 0.378517 |
| cg12188928 | 5.34E-06 | 17 | rs73986768;rs184713236 | 0.548667 | 0.525128 | 0.529113 | 0.604486 |
| cg01379035 | 5.33E-06 | 11 | rs192258561 | 0.594496 | 0.564516 | 0.580439 | 0.641058 |
| cg08800144 | 5.29E-06 | 20 | rs141272370;rs927111 | 0.312448 | 0.291401 | 0.274217 | 0.391661 |
| cg16047291 | 5.27E-06 | 1 | rs550081613 | 0.541229 | 0.504323 | 0.517962 | 0.6123 |
| cg01693697 | 5.24E-06 | 15 | rs7182614 | 0.527243 | 0.494492 | 0.509923 | 0.626767 |
| cg06289919 | 5.24E-06 | 1 | rs367814926;rs552786781; rs186032208;rs538509279 | 0.34014 | 0.304069 | 0.306748 | 0.415074 |
| cg00675637 | 5.18E-06 | 10 | rs542315488;rs562067464 | 0.573516 | 0.5514 | 0.560143 | 0.649164 |
| cg01577632 | 5.17E-06 | 17 | rs368680687;rs530879469 | 0.50221 | 0.479736 | 0.485998 | 0.556544 |
| cg04478489 | 5.12E-06 | 3 | rs541265079 | 0.672902 | 0.651937 | 0.662879 | 0.747408 |
| cg26971210 | 5.07E-06 | 15 | rs544890184 | 0.331664 | 0.308725 | 0.308108 | 0.420919 |
| cg06926619 | 5.05E-06 | 17 | rs542393255 | 0.329414 | 0.305483 | 0.297476 | 0.390871 |
| cg03090145 | 5.05E-06 | 4 | rs558266903 | 0.370238 | 0.346538 | 0.346057 | 0.502003 |
| cg13509702 | 5.03E-06 | 8 | rs540592083;rs562385156 | 0.341836 | 0.312709 | 0.312992 | 0.406792 |
| cg14876940 | 4.93E-06 | 8 | rs569078853 | 0.323921 | 0.302554 | 0.315512 | 0.393212 |
| cg18335377 | 4.92E-06 | 1 | rs557934513 | 0.41391 | 0.393015 | 0.393154 | 0.478996 |
| cg08276755 | 4.89E-06 | 20 | rs151279021 | 0.502745 | 0.482089 | 0.499625 | 0.598645 |
| cg04571183 | 4.89E-06 | 16 | rs142521997 | 0.673132 | 0.65203 | 0.660523 | 0.710225 |
| cg24886006 | 4.85E-06 | 22 | rs138696621;rs141697530; rs146216738;rs532848243 | 0.350507 | 0.330166 | 0.31721 | 0.458188 |
| cg06562883 | 4.81E-06 | 22 | rs576767958;rs545728171 | 0.310539 | 0.282489 | 0.287566 | 0.366804 |
| cg07821355 | 4.72E-06 | 6 | rs542285621;rs57679449 | 0.562814 | 0.54099 | 0.549895 | 0.606729 |
| cg21249659 | 4.71E-06 | 12 | rs185281413 | 0.306768 | 0.280763 | 0.262155 | 0.382005 |
| cg03060184 | 4.57E-06 | 19 | rs543264297;rs3764542; rs565372698 | 0.353143 | 0.319817 | 0.309402 | 0.42767 |
| cg06623544 | 4.57E-06 | 7 | rs552046928;rs568645604; rs536094563 | 0.329259 | 0.306105 | 0.301993 | 0.392476 |
| cg07704771 | 4.54E-06 | 6 | rs73754845;rs560456615 | 0.412111 | 0.386028 | 0.386975 | 0.530342 |
| cg26225774 | 4.51E-06 | 3 | rs182447110 | 0.522061 | 0.469241 | 0.476947 | 0.613789 |
| cg25117333 | 4.45E-06 | 2 | rs560350568;rs112347275; rs192684944;rs139887193 | 0.289159 | 0.261074 | 0.24731 | 0.391965 |
| cg23637008 | 4.42E-06 | 17 | rs138602634 | 0.44042 | 0.417009 | 0.416048 | 0.508358 |
| cg00215011 | 4.24E-06 | 2 | rs189792550;rs534591170; rs184018783;rs3754935 | 0.394544 | 0.3582 | 0.345837 | 0.427998 |
| cg05287483 | 4.22E-06 | 20 | rs534135576 | 0.254672 | 0.215182 | 0.221235 | 0.344476 |
| cg02150868 | 4.17E-06 | 5 | rs571208032;rs538533462; rs117342134 | 0.331821 | 0.288417 | 0.279439 | 0.407161 |
| cg21858904 | 4.14E-06 | 1 | rs538056010 | 0.345172 | 0.309528 | 0.29397 | 0.417634 |
| cg24275611 | 4.12E-06 | 10 | rs146424267;rs545975845; rs140861871;rs150178321 | 0.112829 | 0.090363 | 0.100588 | 0.134915 |
| cg06186155 | 4.10E-06 | 17 |  | 0.835293 | 0.814656 | 0.807434 | 0.863209 |
| cg18447934 | 4.05E-06 | 12 | rs548348971;rs570078085; rs530892357 | 0.304077 | 0.282033 | 0.279293 | 0.36797 |
| cg26588061 | 4.03E-06 | 10 | rs543601092;rs140570235; rs532217563 | 0.432192 | 0.386061 | 0.399708 | 0.530023 |
| cg04128669 | 3.99E-06 | 2 |  | 0.441071 | 0.406112 | 0.42198 | 0.546131 |
| cg10578851 | 3.89E-06 | 2 |  | 0.38507 | 0.360535 | 0.363964 | 0.442666 |
| cg13223011 | 3.79E-06 | 8 | rs532610385;rs185114950 | 0.429266 | 0.408067 | 0.395903 | 0.543333 |
| cg21839974 | 3.74E-06 | 9 | rs113429895;rs546738030; rs147149721 | 0.391871 | 0.349663 | 0.350923 | 0.50101 |
| cg15931859 | 3.60E-06 | 22 | rs138105435;rs560542290; rs192318930;rs546133354; rs41537347 | 0.402782 | 0.377817 | 0.382882 | 0.467356 |
| cg09374015 | 3.57E-06 | 3 |  | 0.557345 | 0.530972 | 0.540044 | 0.641516 |
| cg13485503 | 3.48E-06 | 6 | rs570006566;rs560499084; rs78695435;rs568374448 | 0.419069 | 0.385899 | 0.398712 | 0.504904 |
| cg08876198 | 3.47E-06 | 16 | rs144950150;rs138955018 | 0.475472 | 0.449322 | 0.458398 | 0.547594 |
| cg07708637 | 3.45E-06 | 6 | rs55680618;rs533482210 | 0.202347 | 0.18015 | 0.172023 | 0.268183 |
| cg18134292 | 3.29E-06 | 7 |  | 0.568069 | 0.541449 | 0.54856 | 0.629319 |
| cg22237859 | 3.28E-06 | 4 | rs189083312;rs114145514; rs28820655 | 0.457805 | 0.407338 | 0.425981 | 0.562842 |
| cg06952027 | 3.26E-06 | X |  | 0.541758 | 0.521685 | 0.525009 | 0.59547 |
| cg13781414 | 3.16E-06 | 9 | rs531605131;rs150369320 | 0.663466 | 0.639908 | 0.642303 | 0.704955 |
| cg16246347 | 3.16E-06 | 21 | rs566905194;rs181994433; rs555541814 | 0.304713 | 0.270647 | 0.275435 | 0.384545 |
| cg19981226 | 3.16E-06 | 12 | rs539444095;rs557998606 | 0.286865 | 0.259767 | 0.268974 | 0.373977 |
| cg24417971 | 3.14E-06 | 7 | rs538688496;rs143146033; rs185683980 | 0.334733 | 0.310118 | 0.301142 | 0.406855 |
| cg20587151 | 3.14E-06 | 11 | rs542671649;rs560838342 | 0.589631 | 0.561344 | 0.573302 | 0.666033 |
| cg15893090 | 3.13E-06 | 1 | rs546952761;rs186982019 | 0.249454 | 0.218789 | 0.210763 | 0.327564 |
| cg09349128 | 3.11E-06 | 22 | rs28649194;rs75868168 | 0.246066 | 0.22221 | 0.229909 | 0.328405 |
| cg10331755 | 3.10E-06 | 8 |  | 0.675564 | 0.65372 | 0.670191 | 0.73422 |
| cg16176984 | 3.10E-06 | 15 |  | 0.310549 | 0.257284 | 0.26919 | 0.413574 |
| cg01615382 | 3.09E-06 | 6 | rs143572773;rs541773874; rs537123281 | 0.301724 | 0.279729 | 0.280485 | 0.366006 |
| cg04598292 | 3.03E-06 | 7 | rs368508425 | 0.208718 | 0.186574 | 0.18691 | 0.290072 |
| cg00208768 | 2.95E-06 | 17 | rs544456080;rs558088482 | 0.303839 | 0.271515 | 0.260351 | 0.375852 |
| cg17178175 | 2.89E-06 | 2 | rs13402068;rs568109385; rs373013655 | 0.411049 | 0.384821 | 0.403688 | 0.507104 |
| cg19563049 | 2.71E-06 | 6 |  | 0.309026 | 0.272441 | 0.272177 | 0.388051 |
| cg12523066 | 2.69E-06 | 8 | rs544708938;rs183718965; rs578218683 | 0.318674 | 0.272939 | 0.275508 | 0.413621 |
| cg06356163 | 2.64E-06 | 16 | rs576305794 | 0.557881 | 0.530995 | 0.529497 | 0.614191 |
| cg01539568 | 2.60E-06 | 13 | rs147272804;rs148705816; rs74078419;rs141421544 | 0.385981 | 0.362174 | 0.372575 | 0.434655 |
| cg21870229 | 2.60E-06 | 21 | rs545847214;rs564090819 | 0.211704 | 0.189078 | 0.1859 | 0.270578 |
| cg01509853 | 2.60E-06 | 7 | rs560335272;rs187780496; rs552434604;rs141722802 | 0.646881 | 0.619633 | 0.630027 | 0.705075 |
| cg05648752 | 2.58E-06 | 15 | rs534054564;rs144777333 | 0.495668 | 0.462991 | 0.462492 | 0.545709 |
| cg11621632 | 2.48E-06 | 10 | rs60839064;rs530238016 | 0.222812 | 0.200175 | 0.203373 | 0.279556 |
| cg00052692 | 2.47E-06 | 12 | rs569876255 | 0.486581 | 0.419216 | 0.406048 | 0.524437 |
| cg13215646 | 2.44E-06 | 20 | rs150549436 | 0.787892 | 0.763822 | 0.772246 | 0.835946 |
| cg16652288 | 2.43E-06 | 3 |  | 0.328881 | 0.307275 | 0.303553 | 0.398495 |
| cg05355216 | 2.38E-06 | 13 | rs566040116;rs116721996 | 0.691816 | 0.670228 | 0.679678 | 0.749335 |
| cg03583318 | 2.36E-06 | 6 | rs4145431;rs535732548; rs115311986 | 0.433 | 0.402907 | 0.39713 | 0.497866 |
| cg23564253 | 2.34E-06 | 21 | rs557145592;rs187627373 | 0.495308 | 0.471482 | 0.461533 | 0.559754 |
| cg02021984 | 2.32E-06 | 1 | rs559891766;rs114187621 | 0.428026 | 0.403741 | 0.402567 | 0.540325 |
| cg21380024 | 2.22E-06 | 17 |  | 0.471334 | 0.449693 | 0.467066 | 0.385826 |
| cg17543253 | 2.22E-06 | 12 | rs3026498;rs539858897; rs76824890 | 0.27766 | 0.255725 | 0.253556 | 0.331934 |
| cg11902329 | 2.05E-06 | 2 | rs562781364;rs4075150; rs548358138;rs148996505 | 0.5509 | 0.507091 | 0.499737 | 0.670255 |
| cg00814213 | 1.98E-06 | 17 | rs183076699;rs539769781 | 0.326501 | 0.285965 | 0.281324 | 0.425899 |
| cg22994883 | 1.97E-06 | 7 | rs531117697;rs563900394; rs531036850;rs552818520 | 0.375141 | 0.354688 | 0.360427 | 0.477404 |
| cg00527825 | 1.89E-06 | 15 | rs546934705;rs568404897; rs16958477 | 0.789362 | 0.76609 | 0.775752 | 0.830377 |
| cg23653454 | 1.88E-06 | 7 | rs112532702;rs539429241; rs138708976 | 0.28085 | 0.260287 | 0.252745 | 0.349239 |
| cg01952989 | 1.87E-06 | 7 | rs552644114;rs557396572; rs577350422;rs115019570; rs553475116 | 0.502105 | 0.462457 | 0.461749 | 0.645327 |
| cg23014425 | 1.78E-06 | 17 |  | 0.9166 | 0.894065 | 0.883817 | 0.935961 |
| cg18416097 | 1.70E-06 | 22 | rs536309559 | 0.252837 | 0.21747 | 0.207982 | 0.313849 |
| cg01643203 | 1.70E-06 | 1 | rs187948244;rs79158418 | 0.381913 | 0.346901 | 0.35837 | 0.463662 |
| cg02893324 | 1.69E-06 | 2 | rs193176570 | 0.466192 | 0.433461 | 0.437062 | 0.592007 |
| cg16993108 | 1.68E-06 | 7 | rs148791716;rs190652331; rs552644114;rs557396572 | 0.621347 | 0.583836 | 0.597993 | 0.772194 |
| cg06693170 | 1.63E-06 | 2 | rs116634293;rs117796085; rs10174818;rs529746930 | 0.488851 | 0.456688 | 0.464878 | 0.562793 |
| cg26957636 | 1.60E-06 | 20 | rs188936050 | 0.563731 | 0.537477 | 0.543397 | 0.616692 |
| cg05167074 | 1.53E-06 | 19 |  | 0.455691 | 0.429444 | 0.429706 | 0.573419 |
| cg16713119 | 1.51E-06 | 8 | rs542639951;rs574145090 | 0.395604 | 0.370157 | 0.366304 | 0.497867 |
| cg12244594 | 1.50E-06 | 8 | rs184732267;rs144757847 | 0.447157 | 0.402199 | 0.395862 | 0.538889 |
| cg13695027 | 1.46E-06 | 1 | rs181749961 | 0.312923 | 0.278996 | 0.272716 | 0.393928 |
| cg23720123 | 1.43E-06 | 21 | rs561916447 | 0.481397 | 0.442399 | 0.45754 | 0.549618 |
| cg07276358 | 1.40E-06 | 13 | rs552417972;rs186262298; rs141266684;rs112242825 | 0.758327 | 0.737896 | 0.73979 | 0.83591 |
| cg08721478 | 1.29E-06 | 1 | rs574698076;rs151263069 | 0.278903 | 0.257084 | 0.246823 | 0.33751 |
| cg19717326 | 1.24E-06 | 19 |  | 0.703142 | 0.677154 | 0.693695 | 0.739346 |
| cg16169513 | 1.23E-06 | 17 |  | 0.621156 | 0.597764 | 0.605797 | 0.67334 |
| cg02651683 | 1.19E-06 | 1 | rs534272790 | 0.515396 | 0.487744 | 0.503281 | 0.591895 |
| cg09674775 | 1.16E-06 | 2 | rs141300495;rs546757457 | 0.705953 | 0.661061 | 0.666187 | 0.778048 |
| cg25310867 | 1.07E-06 | 11 |  | 0.478046 | 0.452943 | 0.459138 | 0.569534 |
| cg19716697 | 1.05E-06 | 8 |  | 0.664885 | 0.640528 | 0.656228 | 0.704415 |
| cg14523221 | 1.03E-06 | 17 | rs578044209;rs80252475; rs61571884 | 0.600586 | 0.575681 | 0.574498 | 0.655307 |
| cg06528771 | 1.00E-06 | 2 | rs188886109;rs541105798 | 0.357533 | 0.332045 | 0.327183 | 0.466412 |
| cg04051206 | 9.38E-07 | 17 | rs564079321;rs146285322; rs540479331;rs137864824 | 0.650391 | 0.625817 | 0.620945 | 0.710216 |
| cg01776207 | 9.37E-07 | 5 | rs138215710;rs575035194 | 0.315454 | 0.283341 | 0.276425 | 0.422616 |
| cg21421087 | 9.34E-07 | 15 |  | 0.500594 | 0.468737 | 0.47442 | 0.591241 |
| cg25119028 | 8.90E-07 | 17 | rs183118250;rs575244058; rs544201541 | 0.580505 | 0.553669 | 0.560888 | 0.625154 |
| cg11262012 | 8.45E-07 | 9 | rs552067445;rs565314581; rs527920146 | 0.401827 | 0.37361 | 0.386073 | 0.520919 |
| cg11771151 | 8.29E-07 | 11 | rs537029028;rs556812882 | 0.394134 | 0.368466 | 0.361558 | 0.496142 |
| cg07252680 | 7.81E-07 | 14 |  | 0.437581 | 0.40825 | 0.414537 | 0.51786 |
| cg00401972 | 7.63E-07 | 16 | rs533099944 | 0.35146 | 0.318149 | 0.329056 | 0.418826 |
| cg01231543 | 7.33E-07 | 16 | rs542735272;rs554558785 | 0.643692 | 0.615586 | 0.619622 | NA |
| cg19240637 | 7.15E-07 | 2 | rs557691278 | 0.409259 | 0.378998 | 0.373453 | 0.460379 |
| cg19281948 | 6.85E-07 | 2 |  | 0.383131 | 0.362781 | 0.366118 | 0.443304 |
| cg01799015 | 6.67E-07 | 19 | rs150901074 | 0.496592 | 0.462002 | 0.459474 | 0.558321 |
| cg24213830 | 6.17E-07 | 12 | rs549292640 | 0.497195 | 0.471567 | 0.488752 | 0.556662 |
| cg04253213 | 6.01E-07 | 3 | rs148397439 | 0.339051 | 0.311242 | 0.310989 | 0.439538 |
| cg00797407 | 5.99E-07 | 20 |  | 0.464341 | 0.443761 | 0.443887 | 0.522376 |
| cg13413819 | 5.96E-07 | 2 | rs535772638 | 0.37587 | 0.355546 | 0.359788 | 0.44694 |
| cg11047885 | 5.75E-07 | 4 | rs536509346;rs563305691; rs146069332;rs542432608; rs78390043 | 0.391521 | 0.361993 | 0.345712 | 0.493097 |
| cg17025698 | 5.46E-07 | 17 | rs559618686 | 0.635285 | 0.610268 | 0.623915 | 0.708366 |
| cg00989806 | 5.42E-07 | 2 | rs542752055;rs554639663 | 0.438052 | 0.390069 | 0.400356 | 0.567383 |
| cg03482769 | 5.34E-07 | 19 | rs111617519;rs376351840 | 0.727732 | 0.707714 | 0.714099 | 0.783776 |
| cg20995564 | 4.65E-07 | 2 | rs549658388;rs139922284 | 0.483262 | 0.443386 | 0.461869 | 0.615027 |
| cg02111474 | 4.39E-07 | 11 | rs542133735;rs113935022 | 0.505248 | 0.474693 | 0.480634 | 0.586428 |
| cg16612503 | 4.34E-07 | 21 | rs149500451 | 0.286845 | 0.259403 | 0.265247 | 0.364703 |
| cg00483891 | 4.14E-07 | 2 | rs575187186 | 0.637407 | 0.616256 | 0.61904 | 0.72943 |
| cg21860329 | 3.61E-07 | 13 | rs200378367;rs184500543 | 0.360832 | 0.320827 | 0.313728 | 0.48503 |
| cg17164954 | 3.43E-07 | 6 | rs539524474;rs370490872 | 0.212306 | 0.190521 | 0.194773 | 0.289371 |
| cg20703530 | 3.31E-07 | 1 | rs557313238;rs575676948; rs390468 | 0.461401 | 0.433298 | 0.430695 | 0.564167 |
| cg06557644 | 3.30E-07 | 7 | rs572665238;rs138213714 | 0.455095 | 0.433075 | 0.43965 | 0.508023 |
| cg08122070 | 3.16E-07 | 1 | rs538913792;rs558850258 | 0.455485 | 0.40572 | 0.414398 | 0.599421 |
| cg13940723 | 2.87E-07 | 7 | rs539043556 | 0.597329 | 0.570929 | 0.575057 | 0.658579 |
| cg06981309 | 2.67E-07 | 3 | rs180690800;rs530012249; rs541795524;rs4434119; rs2738907 | 0.355392 | 0.320547 | 0.312895 | 0.434669 |
| cg18666552 | 2.65E-07 | 17 | rs74520623;rs115341498 | 0.589081 | 0.568354 | 0.584267 | 0.640677 |
| cg25437672 | 2.58E-07 | 5 | rs543273180;rs73787104; rs528829727 | 0.611025 | 0.586838 | 0.591371 | 0.651484 |
| cg15177103 | 2.48E-07 | 2 | rs117799400;rs539306298; rs558712881;rs575706402 | 0.438896 | 0.418615 | 0.434999 | 0.509713 |
| cg04196862 | 2.47E-07 | 17 | rs9299;rs113520215; rs377767191;rs57183284 | 0.335832 | 0.29974 | 0.301318 | 0.433153 |
| cg21735558 | 2.46E-07 | 15 | rs183849748;rs141294412 | 0.322301 | 0.288523 | 0.282754 | 0.42329 |
| cg10646607 | 2.44E-07 | 22 | rs191833634;rs557398587 | 0.631827 | 0.610307 | 0.611953 | 0.679469 |
| cg07651316 | 2.31E-07 | 16 | rs73505419;rs538060054; rs200012973;rs558401184 | 0.657031 | 0.629954 | 0.648137 | 0.753157 |
| cg11971423 | 2.19E-07 | 17 | rs544433055 | 0.579037 | 0.54851 | 0.54637 | 0.635219 |
| cg09430344 | 2.03E-07 | 16 | rs534758966;rs554651930; rs34561002 | 0.64116 | 0.590086 | 0.577779 | 0.67235 |
| cg06705017 | 2.01E-07 | 18 | rs532531723 | 0.342542 | 0.311572 | 0.318933 | 0.411582 |
| cg19526751 | 1.95E-07 | 20 |  | 0.463073 | 0.43444 | 0.445866 | 0.53101 |
| cg05631117 | 1.54E-07 | 11 | rs111257485;rs554186843 | 0.371759 | 0.341624 | 0.332952 | 0.466781 |
| cg10180440 | 1.53E-07 | 10 | rs182211982;rs148795942; rs187245531 | 0.513254 | 0.474713 | 0.481978 | 0.611713 |
| cg22298224 | 1.51E-07 | 11 | rs187226680 | 0.388205 | 0.355617 | 0.361802 | 0.503406 |
| cg15839964 | 1.43E-07 | 6 | rs185208638;rs140270270 | 0.382427 | 0.344713 | 0.361765 | 0.523233 |
| cg12126038 | 1.35E-07 | 14 | rs150038888;rs575557805 | 0.482302 | 0.461392 | 0.463307 | 0.553011 |
| cg12994228 | 1.35E-07 | 8 | rs113489988;rs115697442 | 0.377231 | 0.340317 | 0.342756 | 0.468493 |
| cg06203221 | 1.33E-07 | 4 | rs577832679;rs143827252 | 0.711894 | 0.678339 | 0.690764 | 0.764918 |
| cg01066175 | 1.32E-07 | 2 |  | 0.641029 | 0.618266 | 0.619669 | 0.706139 |
| cg17348029 | 1.27E-07 | 2 | rs530928238 | 0.354931 | 0.328515 | 0.331128 | 0.454948 |
| cg26204448 | 1.26E-07 | 7 | rs10268609;rs372983269; rs577645677;rs138880732; rs141400847 | 0.61507 | 0.563105 | 0.570886 | 0.752779 |
| cg07957619 | 1.13E-07 | 6 | rs183248493 | 0.375938 | 0.351816 | 0.353746 | 0.450592 |
| cg09424830 | 1.02E-07 | 9 | rs144517091;rs145586690; rs530063525 | 0.591826 | 0.541287 | 0.533731 | 0.639573 |
| cg07958818 | 1.00E-07 | 1 |  | 0.613797 | 0.591946 | 0.593512 | 0.662301 |
| cg06972745 | 8.86E-08 | 22 | rs575051875 | 0.24401 | 0.219464 | 0.209682 | 0.310583 |
| cg00156232 | 8.29E-08 | 7 | rs569610068;rs111749975; rs538436453;rs116333273 | 0.497582 | 0.475151 | 0.491498 | 0.612829 |
| cg26916621 | 7.94E-08 | 17 | rs554769879;rs574837970 | 0.627889 | 0.587171 | 0.583853 | 0.690325 |
| cg03741653 | 6.10E-08 | 8 | rs566510720 | 0.604003 | 0.56606 | 0.576724 | 0.705881 |
| cg24184014 | 4.84E-08 | 2 |  | 0.582014 | 0.544739 | 0.542232 | 0.63563 |
| cg14162417 | 4.84E-08 | 1 | rs535630793;rs139266598 | 0.514374 | 0.489192 | 0.499087 | 0.567065 |
| cg19437677 | 4.63E-08 | 1 | rs16835852 | 0.51826 | 0.483843 | 0.4925 | 0.596352 |
| cg01208778 | 4.22E-08 | 3 | rs553045911;rs150576079 | 0.407699 | 0.377098 | 0.387944 | 0.470623 |
| cg04498312 | 3.84E-08 | 13 | rs140377151;rs564421798 | 0.321193 | 0.281835 | 0.291128 | 0.366006 |
| cg15537305 | 3.47E-08 | 17 | rs555895830 | 0.490708 | 0.469961 | 0.471679 | 0.609268 |
| cg07573872 | 2.98E-08 | 19 | rs575569058;rs543149275; rs112410887 | 0.66066 | 0.635348 | 0.636954 | 0.748809 |
| cg19358608 | 2.32E-08 | 20 | rs6081325;rs572853755; rs572310690 | 0.496227 | 0.460926 | 0.45573 | 0.566407 |
| cg11099946 | 1.92E-08 | 4 | rs74664209;rs372798888 | 0.498153 | 0.465255 | 0.467499 | 0.553744 |
| cg11460520 | 9.72E-09 | 14 | rs187505166;rs141073831 | 0.518569 | 0.484512 | 0.487569 | 0.607644 |
| cg10561625 | 9.46E-09 | 15 |  | 0.399653 | 0.376259 | 0.392881 | 0.45709 |
| cg10771504 | 7.42E-09 | 8 |  | 0.50701 | 0.482306 | 0.489686 | 0.595324 |
| cg06258764 | 6.17E-09 | 10 | rs536470321;rs369798479; rs573090263;rs373037223 | 0.329671 | 0.305823 | 0.309758 | 0.417091 |
| cg20959703 | 5.65E-09 | 19 | rs562801283 | 0.491065 | 0.467396 | 0.475844 | 0.55657 |
| cg03067296 | 4.67E-09 | 17 | rs149970163 | 0.572279 | 0.52389 | 0.533267 | 0.641923 |
| cg02836478 | 4.04E-09 | 17 |  | 0.746374 | 0.712985 | 0.699049 | 0.788388 |
| cg08282375 | 3.97E-09 | 20 | rs185329255;rs113874691; rs78406311;rs536849584; rs555240922 | 0.339895 | 0.314532 | 0.31257 | 0.415456 |
| cg16969885 | 2.58E-09 | 17 | rs550788296;rs147508118 | 0.685814 | 0.665384 | 0.677411 | 0.755444 |
| cg03665051 | 1.96E-09 | 22 | rs540273863;rs565234415 | 0.44637 | 0.417197 | 0.428213 | 0.541287 |
| cg09342060 | 1.74E-09 | 7 | rs181742818;rs368820871 | 0.496917 | 0.467187 | 0.45913 | 0.576816 |
| cg19821297 | 1.25E-09 | 19 |  | 0.368737 | 0.34107 | 0.345666 | 0.477302 |
| cg05230392 | 1.01E-09 | 17 | rs543828572;rs143812597; rs565147296 | 0.707924 | 0.664026 | 0.662367 | 0.753784 |
| cg01839793 | 7.44E-10 | 15 | rs185686230;rs544926356 | 0.494881 | 0.472739 | 0.47678 | 0.581914 |
| cg12992827 | 6.81E-10 | 3 | rs190136083;rs181844988 | 0.521328 | 0.494836 | 0.491213 | 0.650262 |
| cg05844798 | 4.79E-10 | 20 | rs144778921;rs568383154; rs147930298 | 0.693459 | 0.669639 | 0.65362 | 0.742111 |
| cg06072257 | 3.51E-10 | 1 | rs552580160;rs72860204 | 0.62075 | 0.58653 | 0.58759 | 0.671528 |
| cg02491794 | 2.34E-10 | 2 | rs189445960;rs143444133 | 0.603914 | 0.575848 | 0.593292 | 0.692924 |
| cg01936839 | 2.23E-10 | 7 |  | 0.42597 | 0.390414 | 0.387538 | 0.486257 |
| cg25824462 | 1.36E-10 | 7 | rs547230889 | 0.598188 | 0.569669 | 0.57217 | 0.657623 |
| cg12999836 | 7.05E-11 | 7 | rs568500085 | 0.557259 | 0.510793 | 0.526908 | 0.659215 |
| cg12814730 | 4.26E-11 | 15 | rs563208617;rs141026185 | 0.746899 | 0.714241 | 0.718061 | 0.818618 |
| cg01072106 | 9.40E-14 | 9 | rs139577741;rs73668056 | 0.640472 | 0.619325 | 0.624478 | 0.729209 |
